# Symbiotic bacteria of the gall-inducing mite *Fragariocoptes setiger* (Eriophyoidea) and phylogenomic resolution of the eriophyoid position among Acari

**DOI:** 10.1038/s41598-022-07535-3

**Published:** 2022-03-09

**Authors:** Pavel B. Klimov, Philipp E. Chetverikov, Irina E. Dodueva, Andrey E. Vishnyakov, Samuel J. Bolton, Svetlana S. Paponova, Ljudmila A. Lutova, Andrey V. Tolstikov

**Affiliations:** 1 X-BIO Institute, Tyumen State University, Tyumen, Russia 625003; 2 Saint-Petersburg State University, St. Petersburg, Russia 199034; 3 Florida Department of Agriculture and Consumer Services, Gainesville, FL USA

**Keywords:** Entomology, Metagenomics

## Abstract

Eriophyoid mites represent a hyperdiverse, phytophagous lineage with an unclear phylogenetic position. These mites have succeeded in colonizing nearly every seed plant species, and this evolutionary success was in part due to the mites' ability to induce galls in plants. A gall is a unique niche that provides the inducer of this modification with vital resources. The exact mechanism of gall formation is still not understood, even as to whether it is endogenic (mites directly cause galls) or exogenic (symbiotic microorganisms are involved). Here we (i) investigate the phylogenetic affinities of eriophyoids and (ii) use comparative metagenomics to test the hypothesis that the endosymbionts of eriophyoid mites are involved in gall formation. Our phylogenomic analysis robustly inferred eriophyoids as closely related to Nematalycidae, a group of deep-soil mites belonging to Endeostigmata. Our comparative metagenomics, fluorescence in situ hybridization, and electron microscopy experiments identified two candidate endosymbiotic bacteria shared across samples, however, it is unlikely that they are gall inducers (morphotype1: novel *Wolbachia*, morphotype2: possibly *Agrobacterium tumefaciens*)*.* We also detected an array of plant pathogens associated with galls that may be vectored by the mites, and we determined a mite pathogenic virus (*Betabaculovirus*) that could be tested for using in biocontrol of agricultural pest mites.

## Introduction

Eriophyoid mites (four-legged mites, gall mites) represent an ancient lineage of common and widely distributed microscopic plant symbionts, with 4400 nominal species primarily associated with ferns, gymnosperms and angiosperms^1,2^. Some of these mites are of agricultural importance, damaging host plants through feeding, gall formation, and vectoring plant pathogens^3–6^. The ability to induce galls in their plant hosts is the most distinctive feature of eriophyoid mites. Galls create a unique niche that ensures the survival and sustainable population growth of mites through their manipulation of the host plant^2^. Many species of eriophyoid mites are gall-forming, indicating that gall formation may be a key innovation, enabling these mites to colonize many terrestrial seed plant species. Gall-forming ability is an evolutionarily labile trait and a possible driver of mite speciation^7^. Different mite species, including non-gall-formers and those that produce different types of galls, can co-exist on a single plant host. Therefore, both host specificity and habitat partitioning via gall formation effectively increase eriophyoid species richness^7^.

Molecular mechanisms of gall formation are relatively well known in bacteria^8^. However, in metazoan organisms, especially in mites and other arthropods, the exact nature of gall formation is not well understood^9–13^. In many phytopathogenic bacteria, gall formation ability is attributed to the production of cytokinins; these compounds, in the presence of auxin, lead to cell division and proliferation of plant tissue, resulting in the formation of galls or tumors^14–16^. Auxin-like and cytokinin-like activities have been detected in the salivary gland secretions of mites, and these phytohormones are delivered to the host plant during feeding^17^. High levels of auxins and cytokinins have been detected in mite-induced gall tissues^18^. It is likely that mites, like gall-forming insects, nematodes, and fungi, produce cytokinins endogenously via the regular *tRNA-ipt* pathway, which is present in all cellular organisms except for Archaea^10,19,20^. Gall-inducing organisms may also inject effector proteins (produced endogenously) such as *bicycle* proteins^21^ or CLE and CEP-peptides which are plant hormone mimics^22,23^. However, associated bacteria may also enhance the production of phytohormones by gall-inducing arthropods^24,25^. Here we test the latter hypothesis of an exogenous mechanism of gall formation^24,25^. Our assumption is that if bacteria play an important role in gall formation, a common bacterial species (gall-inducer) must exist across galls harboring the same mite species. Alternatively, if no common bacterial species is found across conspecific mite samples, then mite-specific bacteria is unlikely to cause galls or enhance their production. A key to this approach is accurate estimation of relative and absolute abundances of mite-associated microorganisms.

The phylogenetic position of Eriophyoidea on the tree of life is also a long-standing and contentious issue. One hypothesis places them near Nematalycidae (Acariformes: Endeostigmata)^26,27^, a deep-soil, basal acariform lineage, whereas another hypothesis places them within Eupodina (Trombidiformes)^26,28^, a relatively derived lineage that includes inhabitants of soil and plants. Definitive resolution of this question is key for understanding the basal relationships and early ecology of acariform mites, one of the oldest known terrestrial lineages^29,30^. Inferring an accurate position for Eriophyoidea is essential to ensure the stability of higher-level mite classification, since the two major mite divisions, Trombidiformes and Acariformes, are effectively undiagnosable without knowing the true position of Eriophyoidea. However, phylogenomic-scale datasets have only recently become available to answer this question.

**Figure 1:**
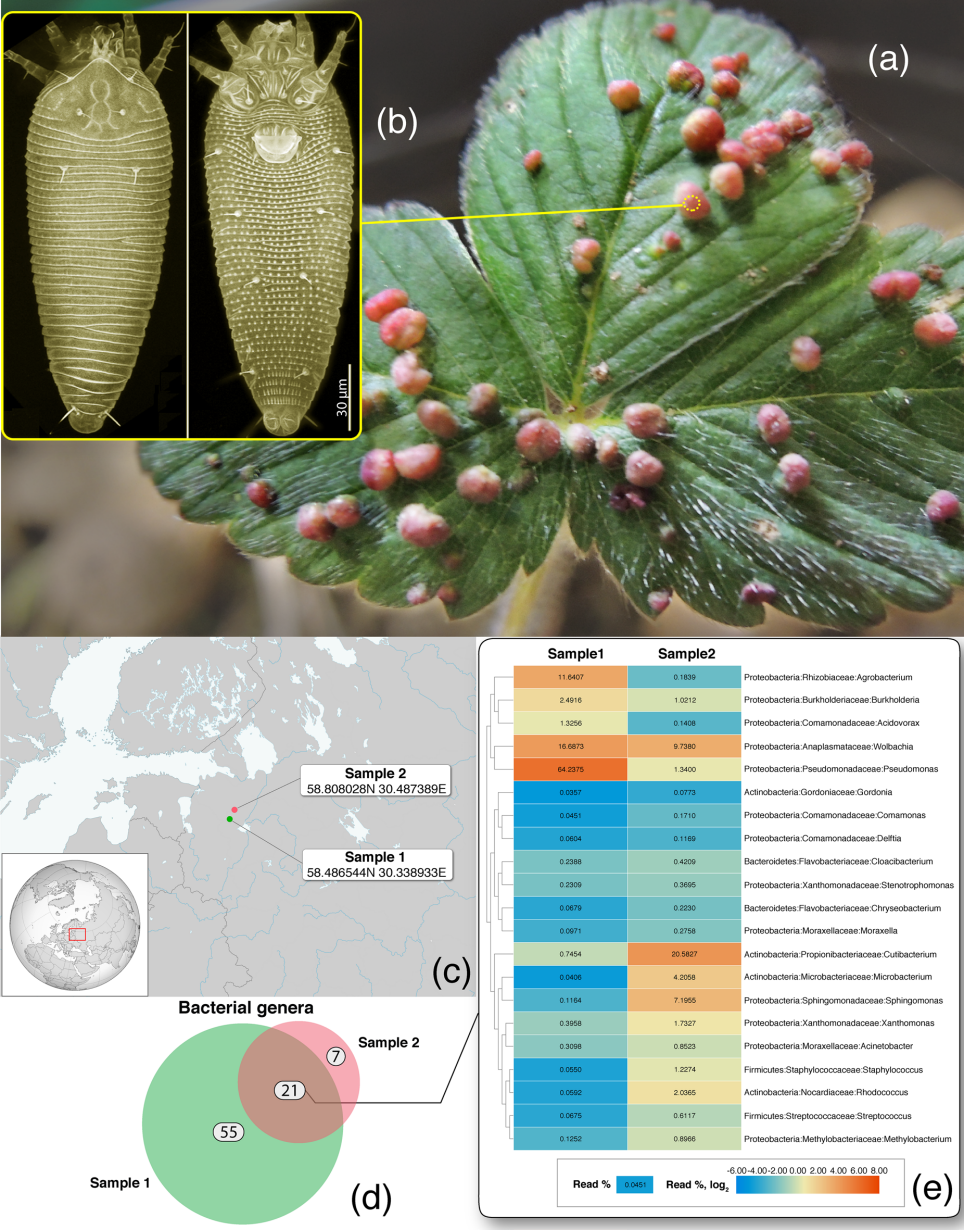
Galls on leaves of the creamy strawberry *Fragaria viridis* (**a**) induced by the mite *Fragariocoptes setiger* (**b**); mite collection localities, samples 1 and 2 (**c**); Bacterial taxonomic richness in samples 1 and 2, the number of unique bacterial genera in each sample and in their intersection is shown as a Venn diagram (**d**), k-mer-based taxonomic classification was done in Kraken using a normalized abundance threshold of ≥ 0.0338%; there was a total of 49,514,852 and 3,830,185 classified bacterial reads in samples 1 and 2, respectively; detailed taxonomic classification and abundance estimates are given in Supplementary Table S2 and visualized in Supplementary Fig. S1; abundance of bacterial genera in the intersection of samples 1 and 2 (**e**), abundance values are percentages of classified reads, while the heatmap colors are based on log_2_-transformed abundance values. Maps were generated by modifying public domain maps in Adobe Illustrator CS6 (https://commons.wikimedia.org/wiki/File:Outline_Map_of_Northwestern_Federal_District.svg, Finland (orthographic projection).svg).

Here we test the two aforementioned hypotheses on exogenous gall formation and the phylogenetic position of Eriophyoidea using deep, short read sequencing of the mite *Fragariocoptes setiger*, which causes distinctive galls on leaves of the green strawberry (*Fragaria viridis*) in Europe (Fig. 1)*.* First, we used several metagenomic (metatranscriptomic) analyses to characterize the microbiome composition of the mite from two independent, geographically isolated samples (sample 1 = genome, sample 2 = transcriptome) (Fig. 1). We then used these data to find a common bacterial species, a potential bacterial gall-inducer. We combined this metagenomic evidence with FISH-hybridization experiments and TEM microscopy. Second, we assembled a whole genome of *F. setiger* and inferred a phylogenomic tree of acariform mites, including two novel genomes of basal endeostigmatan taxa. With respect to the hypothesis on gall formation, other interesting research directions, like searching for genomic signatures of gall formation in mites, will be addressed in separate papers using additional evidence (for example, comparative genomics using a closely related non-gall inducing mite species, namely *Fragariocoptes ambulans*).

## Results

### Genome

The mite genomic assembly was 40,873,958 bp in size, had 3,581 contigs—N50 = 31,600 (Table 1)—and 3056 predicted and annotated genes. Metatranscriptomic assembly (39,862,892 bp, N50 = 1643 for contigs ≥ 500 bp only) had a total of 145,220 contigs; of these, 22,669 could be aligned back to the mite genomic assembly (Supplementary Table S1). BUSCO estimated that the mite genome is 58.7–77.0% complete. Specifically, BUSCO4 (arachnida_odb10, 2934 genes) identified 1724 (58.7%) complete, 56 (1.9%) fragmented, and 1154 (39.4%) missing genes. Of the genes in the former category 1673 (57.0%) were single-copy and 51 (1.7%) were duplicated. BUSCO3 (arthropoda_odb9, 1066 genes) identified the following: complete: 821 (77.0%) [single-copy: 805 (75.5%), duplicated: 16 (1.5%)], fragmented: 41 (3.8%), missing: 204 (19.2%) genes. The mitochondrial genome had the standard set of protein-coding genes, rDNAs, and tRNAs. Mitochondrial gene arrangement (GenBank accession JAIFTH000000000) was similar to that of other known eriophyoid mites, except the 16S-2S rDNA gene segment was inverted in *Fragariocoptes* (lineage Phytoptidae), which corresponds to the ancestral chelicerate order, while in other known eriophyoids (all belong to the lineage Eriophyidae s.l.) this segment is not inverted.

**Table 1:** Basic statistics for three assemblies of the mite *Fragariocoptes setiger* and its microbiome.

	Metagenome*	Metatranscriptome*	Mite genome
Contigs number (N)	70,345	31,089	3581
Total contig size (bp)	139,253,250	39,862,892	40,873,958
Longest contig (bp)	423,970	18,766	182,071
Mean contig length (bp)	1979.58	1282.22	11,414.12
Median contig length (bp)	757	826	3603
Standard deviation of contig length (bp)	7685.98	1167.73	18,445.63
Contig L50 (N)	2683	6522	370
Contig N50 (bp)	6218	1643	31,600
Contig L75 (N)	19,995	15,263	834
Contig N75 (bp)	1102	839	15,183
Contig L90 (N)	45,528	23,782	1445
Contig N90 (bp)	641	599	6110
Average coverage	265.81	192.21	4568.12
Median coverage	6.44	10.57	2480.21
Standard deviation of coverage	3185.71	4161.95	6778.21
Total GC content (%)	54.43%	47.38%	43.18%
Average contig GC content (%)	57.27%	47.66%	43.58%

*For contigs ≥ 500 bp only.

### Phylogenetic analyses

**Figure 2:**
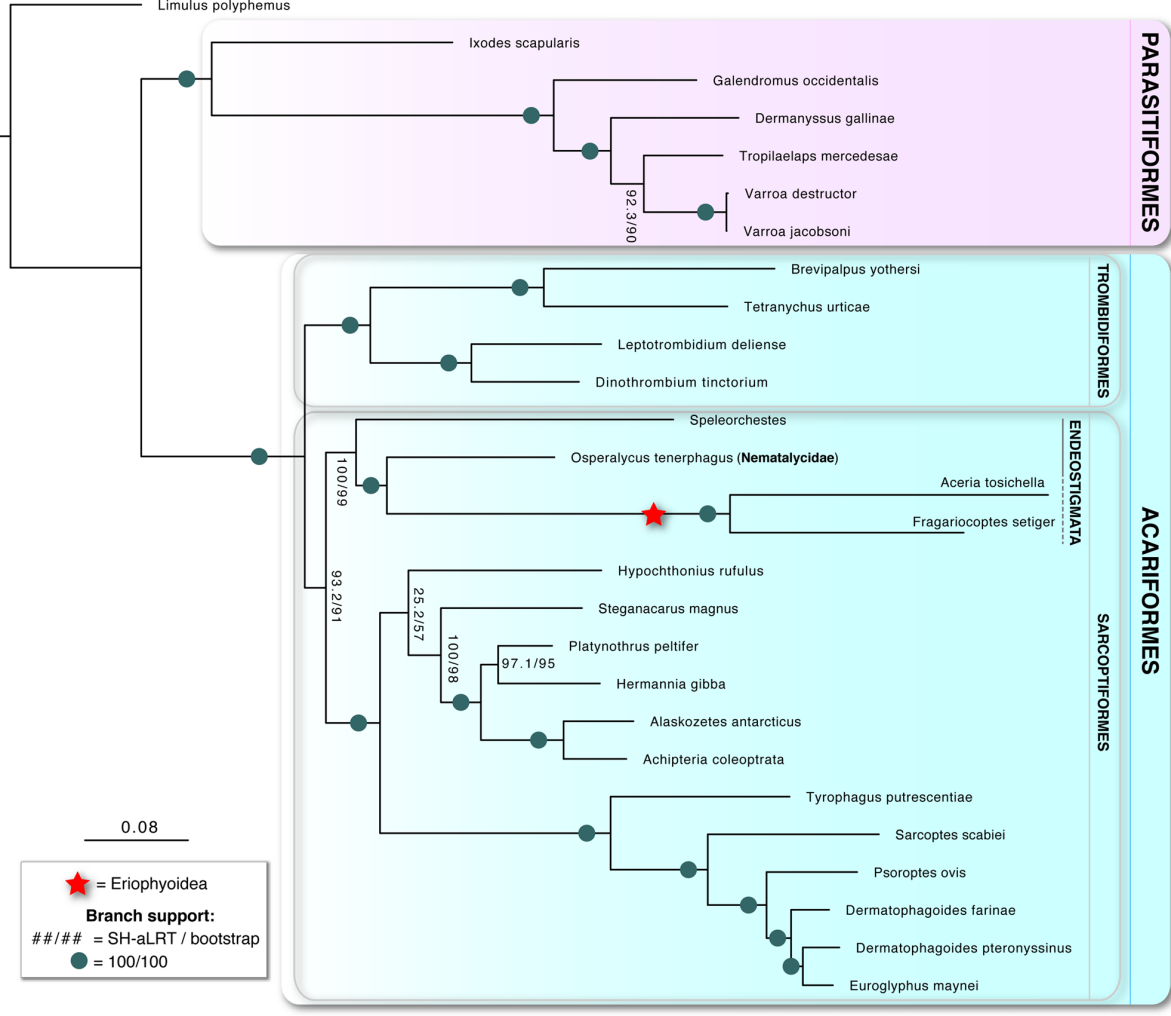
Relationships of parasitiform and acariform mites. Phylogenomic inference was undertaken using a Maximum likelihood framework in IQ-TREE based on 90 orthologous proteins.

**Figure 3:**
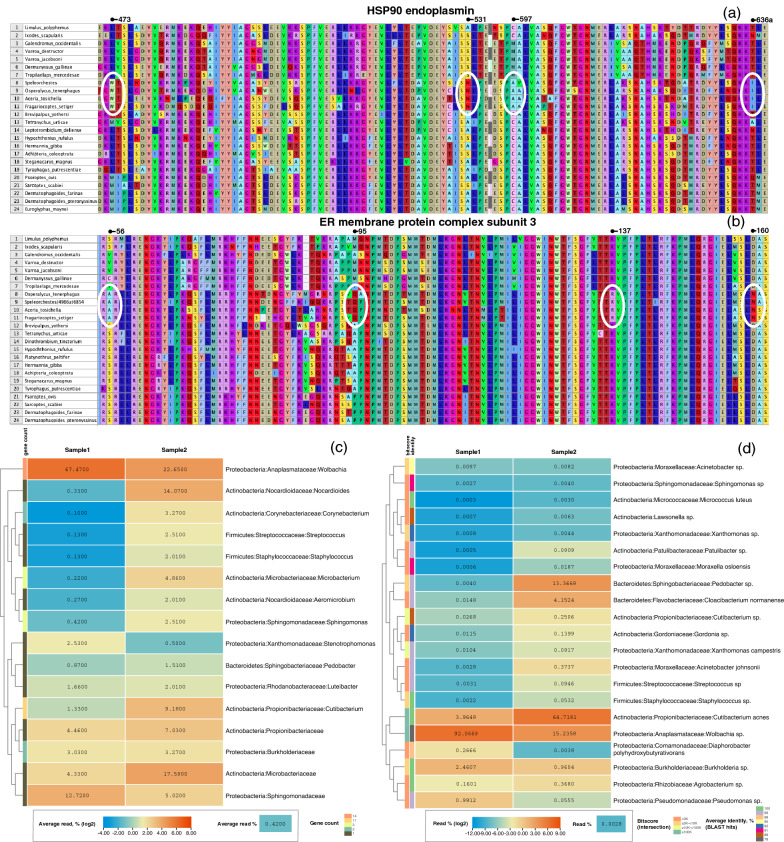
Select molecular autapomorphies for the Endeostigmata, including Eriophyoidea, in two proteins (**a**,** b**): HSP90 endoplasmin (**a**) and ER membrane protein complex subunit 3 (**b**); amino acid alignment coordinates are given for the *Limulus polyphemus* reference proteins (XP_013791125.1, XP_022251172.1). Bacterial OTUs shared between samples 1 and 2 as identified by mapping raw reads onto a set of 14 single-copy genes in singleM (**c**); for each OTU, a gene count returning matches in both samples is given. The heatmap gives read percentages in the intersection, while its colors are based on log_2_ of these values. Normalization was done only for OTUs present in the intersection. Bacterial OTUs shared among samples 1 and 2 as identified via intersection of two assemblies (**d**); for each OTU, intersection bitscore, and average sequence identity (BLAST using GenBank nucleotide database) are given. The heatmap gives normalized read percentages, while its colors are based on log_2_ of these values. Normalization was done only for OTUs present in the intersection. Only alignments having a bitscore ≥ 1500 are shown.

Our phylogenomic analysis shows strong support for Eriophyoidea being part of Endeostigmata (Fig. 2) (SH-aLRT support = 100%, bootstrap support = 99%) (Supplementary Methods: detailed mite systematics). Particularly, Eriophyoidea was sister to Nematalycidae (SH-aLRT support = 100%, bootstrap support = 100%). Inspecting the source gene alignment matrices revealed that the Endeostigmata + Eriophyoidea grouping was supported by many molecular synapomorphies (Fig. 3a,b). Endeostigmata (including Eriophyoidea) was inferred as sister to Sarcoptiformes (SH-aLRT support = 93.2%, bootstrap support = 91%). Topology based on the non-curated matrix was essentially the same (not reported further).

### Mapping reads on reference genes of known gall-inducers (Gall-ID)

A SRST2/Gall-ID analysis identifies gall-inducing bacteria by mapping reads to a reference database of select genes having close similarities or belonging to known gall-inducing bacteria. A single OTU of known gall-inducing bacterium (*Agrobacterium tumefaciens*, nucleotide identity = 99.5–100%) was found in both samples. However, a tumor-inducing Ti-plasmid, encoding loci responsible for the formation of galls, was only partially recovered. Some but not all of these genes were detected in sample 1, particularly the nopaline permease ATP-binding protein gene and a substantial portion of Type VI secretion system components (Table 2). Sample 2 lacked any loci encoded on Ti-plasmids (Table 2). In addition, *Rhodococcus fascians*, with the 16S gene having a 99.7% similarity to GenBank sequences, was detected in sample 2 only; in sample 1, a different *Rhodococcus* OTU was present at low abundance (Table 2). Other known gall-inducing bacteria (*Pseudomonas savastanoi* pv. *phaseolicola* & *glycinea*, *Rhizobium rubi*, *Erwinia herbicola*) were not detected; they only had distant matches to taxa from our datasets (Table 2). Validation of these results via read assembly followed by a BLAST search confirmed that these taxa actually belong to different species of mostly free-living bacteria (Table 2). In addition, these taxa were not present in both samples (Table 2).

**Table 2:** NGS read mapping onto known reference genes of gall-inducing bacteria in Gall-ID. Validation was done by BLAST searches of assembled contigs.

Sample	Gene	Gall-ID reference contig	len1	Depth	Diffs	Diverg	Ref.len	MAF	Read.prop	Cov	Validation (BLAST top hit)	% id	Aln.len
		**Sample 1 and 2**											
S1	16S	20_rRNA16s_rRNA16s_21 (*Rhodococcus fascians*)	99.7	1156.5	85 s,6 i	5.55	1532	0.50	0.1663	11.0	*Rh. olei* (MF405107.1)	97.7	749
S2	16S	20_rRNA16s_rRNA16s_21 (Rhodococcus fascians**)**	99.9	4807.8	39 s,2 i	2.55	1532	0.50	3.5878	484.0	Rh. fascians*	99.9	759
S1	16S	0_16S_Agrobacterium_tumefaciens_WRT31_198	100.0	1835.6	12 s	0.84	1437	0.49	0.2484	136.8	A. tumefaciens*	99.9	1686
S2	16S	0_16S_Agrobacterium_tumefaciens_strain_MEJ076_65	100.0	2,818,172.0	6 s	0.42	1437	0.49	1.9753	46.7	A. tumefaciens*	99.8	627
S1	16S	0_16S_Pseudomonas_savastanoi_pv._phaseolicola_1448A_1	100.0	4612.1	20 s	1.30	1539	0.46	0.6683	706.1	*P. yamanorum* (CP012400.2)	99.9	1621
S2	16S	0_16S_Pseudomonas_savastanoi_pv._glycinea_str._race_4_7	97.6	274.8	34 s,37 h	2.26	1539	0.47	0.2013	93.0	P. sp. DHXJ03 (JN244973.1)	100.0	1088
		**Sample 1 only**											
S1	tssC40	39_tssC40_Agrobacterium_tumefaciens_WRT31_19	100.0	43.8	13 s	0.93	1395	0.31	0.0058	8.5	**	99.4	310
S1	tagH	33_tagH_Agrobacterium_tumefaciens_WRT31_13	100.0	39.0	5 s	0.42	1200	0.05	0.0044	5.7	***	99.8	414
S1	tagJ	34_tagJ_Agrobacterium_tumefaciens_WRT31_15	100.0	35.8	10 s	1.22	822	0.11	0.0028	17.8	****	99.5	1035
S1	tssC41	40_tssC41_Agrobacterium_tumefaciens_WRT31_22	100.0	33.4	1 s	0.07	1482	0.10	0.0047	5.4	**	100.0	307
S1	tssG	44_tssG_Agrobacterium_tumefaciens_WRT31_22	100.0	29.2	3 s	0.30	1005	0.13	0.0028	4.1	***	99.7	363
S1	tagF	32_tagF_Agrobacterium_tumefaciens_WRT31_8	100.0	27.6	15 s	1.06	1410	0.36	0.0037	5.0	****	99.5	411
S1	tssE	42_tssE_Agrobacterium_tumefaciens_WRT31_15	100.0	24.4	1 s	0.20	510	0.07	0.0012	14.8	****	99.6	678
S1	nocP	14_nocP_Agrobacterium_tumefaciens_strain_S2_52	53.9	9.8	39 s,1 i,272 h	12.26	590	0.50	0.0003	5.8	*A. sp.* H13-3 (CP002248.1)	100.0	232
S1	tssF	43_tssF_Agrobacterium_tumefaciens_GW4_16	62.1	3.9	87 s,676 h	7.87	1782	0.00	0.0004	na	na	na	na
S1	tssK	48_tssK_Agrobacterium_sp._H13-3_3	100.0	40.2	1 s	0.08	1341	0.13	0.0051	6.9	***	100.0	404
S1	tssA	37_tssA_Agrobacterium_sp._H13-3_2	100.0	31.6	3 s	0.29	1040	0.11	0.0031	7.8	**	100.0	319
S1	tssD	41_tssD_Agrobacterium_sp._H13-3_2	100.0	31.6		0.00	477	0.08	0.0014	6.8	***	100.0	377
S1	tagE	31_tagE_Agrobacterium_sp._H13-3_3	100.0	28.6	4 s	0.49	810	0.09	0.0022	5.4	***	100.0	325
S1	tssL	49_tssL_Agrobacterium_sp._H13-3_3	99.9	27.7	7 s,1 i	0.46	1512	0.17	0.0039	4.2	****	99.7	337
S1	tssH	45_tssH_Agrobacterium_sp._H13-3_3	100.0	26.8	10 s	0.38	2670	0.27	0.0067	13.8	****	99.7	2876
S1	tssM	50_tssM_Agrobacterium_sp._10MFCol1.1_16	100.0	31.3	22 s	0.63	3480	0.36	0.0102	7.0	***	100.0	358
S1	tssB	38_tssB_Agrobacterium_sp._10MFCol1.1_19	100.0	31.2		0.00	510	0.42	0.0015	7.5	**	100.0	338
S1	tssI	47_2_Agrobacterium_sp._LC34_39	52.3	3.8	55 s,625 h	8.03	1310	0.50	0.0002	na	na	na	na
S1	tssI	46_1_Rhizobium_rubi_NBRC_13261_44	50.8	2.2	60 s,748 h	7.77	1520	0.50	0.0002	na	na	na	na
S1	CysT	7_CysT_Pseudomonas_savastanoi_pv._glycinea_str._B076_6	52.2	16.0	38 s,393 h	8.86	822	0.33	0.0006	na	na	na	na
S1	ISEhe3	16_ISEhe3_ISEhe3_17 (*Erwinia herbicola*)	50.2	6.7	35 s,3 i,754 h	4.59	1516	0.50	0.0005	1.5	*E. persicina* (CP022726.1)	98.5	330

*And other equivocal hits; **Agrobacterium tumefaciens (CP032922.1), *Agrobacterium* sp. H13-3 (CP002249.1); ***Agrobacterium sp. H13-3 (CP002249.1); ****Agrobacterium tumefaciens (CP032922.1); % id = percent identity (BLAST); aln. len = alignment length (BLAST); cov = coverage, k-mer based (assembled contig); diffs = differences between subject and reference (Gall-ID): s = snp, i = indel, h = hole; diverg = divergence; len1 = length coverage of reference (Gall-ID); MAF = Max MAF; na = assembly failed due to low read abundance; read.prop = read proportion*10^6^; ref.len = Reference length.

### K-mer-based short read identification (Kraken)

Kraken decomposes short reads into k-mers of size 35, which allows extremely fast read classification against a reference database. Our database included nearly all GenBank genomic sequences, except plants were represented by *Fragaria vesca* only. For the abundance threshold ≥ 0.0338% for all bacterial reads, 83 bacterial genera were identified in both samples, 28 in sample 1 and 76 in sample 2; 21 genera were shared across the two samples (Fig. 1d). Among the 10 most abundant bacterial genera in each sample, two were shared: *Cutibacterium* and *Wolbachia* (Fig. 1e). Detailed taxonomic classification and abundance estimates for this and other threshold and no-threshold analyses are given in Supplementary Tables S2, S3, S4 and Supplementary Figs. S1 and S2.

### Read mapping on marker genes

We identified OTUs shared across the two samples using mapping of raw reads onto 14 marker, single-copy genes in SingleM^31^ (Supplementary Table S5). This method is largely taxonomy-independent and does not suffer from issues related to copy-number variation in ribosomal genes (16S, 23S), plasmids, and transposable elements. This analysis identified 16 OTUs present in both samples. Of them, 3 OTUs were found at high abundances (percentages of reads are given in parentheses for samples 1 and 2, respectively): *Wolbachia* (67.47, 22.65%), Sphingomonadaceae (12.72, 5.02%), and Propionibacteriaceae (4.46, 7.03%) (Fig. 3c). Remarkably, SingleM did not find *Agrobacterium* and *Pseudomonas* in sample 2, and therefore these genera are not present among the 16 OTUs.

### Assembly intersection

To detect OTUs common to the two samples, the two NGS assemblies were intersected and shared contigs were classified using the BLAST nucleotide database (Fig. 3d). The following four most abundant species were shared between the two samples: *Wolbachia* sp. (92.07%, 15.24%), *Cutibacterium acnes* (3.96%, 64.71%), *Burkholderia* sp. (2.46%, 0.96%), *Agrobacterium* sp. (0.16%, 0.37%) (Fig. 3d). Two species (*Diaphorobacter polyhydroxybutyrativorans*, and *Pseudomonas* sp.) were only abundant in sample 1, and two other species (*Cloacibacterium normanense* and *Pedobacter* sp.) were only abundant in sample 2. All other species occurred at low abundances in both samples (Fig. 3d). In addition, a plant pathogenic bacterium, *Xanthomonas campestris*, was identified in both samples at a low abundance (0.01%, 0.09%).

### Non-bacterial taxa

Because non-bacterial taxa can also induce galls^8^, we conducted a brief exploratory survey of major viruses and fungi that can elicit gall symptoms in their plant hosts using raw Kraken results with the confidence score set to 0.1 (as in all analyses above) but without an abundance cutoff. In our samples, there were 62 genera of viruses, but *Phytoreovirus* (causes galls) was absent (Supplementary Table S4). The two most abundant viral genera were *Pahexavirus* (0.0002%, 0.0012%), which was probably a *Cutibacterium acnes* phage, and *Betabaculovirus* (0%, 0.0006%), which probably uses the mite as a host. Among gall-inducing Oomycota, we found *Albugo laibachii* at a perceptible abundance, especially in sample 2 (0.036%; for comparison, sample 1 = 0.003%) (Supplementary Table S4). The fungus *Ustilago maydis* (causing corn smut) was found at a very low abundance (0.00006% and 0.00416% in samples 1 and 2, respectively; Supplementary Table S4).

### Phytohormones and horizontal gene transfer

The annotated mite genomic assembly revealed no known bacterial/plant genes responsible for the production of phytohormones and enzymes involved in plant growth regulatory metabolism.

### TEM observations

**Figure 4:**
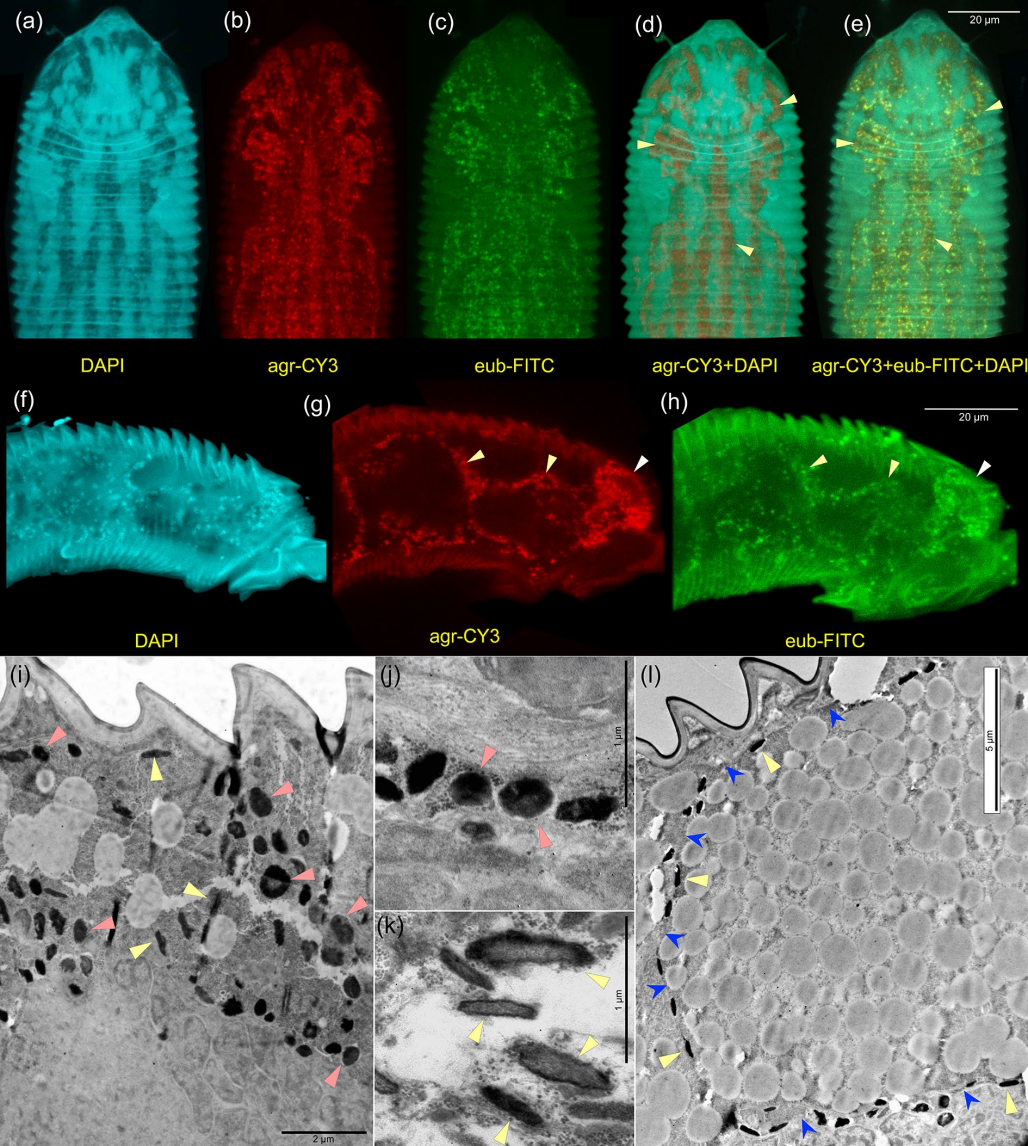
Endosymbiotic bacteria of the mite *Fragariocoptes setiger*, fluorescence in situ hybridization (FISH) with different fluorophores and oligonucleotide probes (**a–h**) and TEM microscopy (**i–l**). Mite anterodorsal (**a–e**) and anterolateral parts (**f–h**); intermuscular bacteria (**d-e**, yellow arrowheads), bacteria surrounding gigantic parenchymal cells (**g**,** h,** yellow arrowheads) and salivary glands (**g**, **h,** white arrowheads); DAPI + no probe (**a**, **f**); CY3 + 16S.1722F.Agr.tum (**b**, **g**); FITC + Eub338 (**c**, **g**); CY3 + 16S.1722F.Agr.tum, DAPI (**d**); CY3 + 16S.1722F.Agr.tum, FITC + eub338, DAPI (**e**). Bacterial morphotype 1 (*Wolbachia*) (**i**, **j**, red arrowheads), and bacterial morphotype 2 (yellow arrowheads) (**k**, **l**, yellow arrowheads) in various locations inside the mite: mid-lateral opisthosoma with saw-like cuticle and underlying tissues are visible (**i**), same as previous, a gigantic parenchymal cell (fat body) is shown and traced by blue arrows (**l**), spaces between the fat body and the gut (**j**, **k**).

We found two endosymbiotic bacterial morphotypes. Morphotype 1 was globular (Fig. 4i–j), which is consistent with the *Wolbachia* morphology. However, unlike all known *Wolbachia*, this bacterium was extracellular (Fig. 4i–j). Morphotype 2 was rod-shaped and also extracellular (Fig. 4i,k,l). Both morphotypes were most often closely associated with mite cell-plasma membranes. There were three distinct localizations: (i) around gigantic parenchymal cells (forming the fat body) filled with what is presumably lipid or glycogen vesicles (Fig. 4l); (ii) around and inside the salivary glands (in both cases surrounding the salivary gland cells, rather being inside these cells); (iii) under the mite epidermis, between the cells of underlying tissues (muscles and the fat body) (Fig. 4i). Bacteria were not found in mite oocytes, inside the gut or gut lumen.

### Fluorescence in situ hybridization

Abundant bacterial cells were detected inside mites using eubacterial (Fig. 4c,h), *Agrobacterium tumefaciens*-specific probes (Fig. 4b,d,g), and a combination of these probes (Fig. 4e). No-probe controls were also done (Fig. 4a,f). There was a substantial bacterial presence around the gigantic parenchymal cells (Fig. 4g,h), in intermuscular spaces of the body (Fig. 4d,e), and around the salivary glands (Fig. 4g,h).

## Discussion

Our phylogenomic analysis robustly inferred Eriophyoidea as sister to Nematalycidae (Fig. 2), a group of deep-soil, vermiform mites belonging to Endeostigmata. This result is well-supported and provides nearly decisive evidence for the long-standing controversy about the phylogenetic position of Eriophyoidea, which could not be confidently placed within a major mite lineage (see detailed discussion in^26,27^). Previous molecular studies were ambiguous, either because of incongruences among different data partitions^26^, unusual relationships involving Astigmata^32,33^, or the lack of sequence data for non-eriophyoid Endeostigmata^32,34–36^. A recent morphological analysis, however, identified several synapomorphies supporting the Eriophyoidea + Nematalycidae lineage^27^, which is in agreement with our result. By placing Eriophyoidea within Endeostigmata, our phylogenomic inference provides the stability for the high-level classification of acariform mites.

Using comparative metagenomics, we also tested whether gall formation in the *Fragariocoptes setiger* system is of a bacterial nature. To find a potential gall-inducer, two independent, geographically isolated samples (sample 1 and 2; Fig. 1) of the gall-inducing mite were analyzed. We conducted several metagenomic (metatranscriptomic) analyses, each using a different methodology: Gall-ID (comparison with known gall-inducers), Kraken (k-mer-based classification using nearly the entire GenBank nucleotide data as the reference database), SingleM (comparison with 14 single-copy bacterial genes), and BLAST (classification of the intersection of the two assemblies). Below we discuss the results of these analyses and then provide a synthesis of these data before giving concluding remarks.

Mapping reads of known gall-inducing bacteria in Gall-ID identified several potential candidates: *Agrobacterium tumefaciens* (99.8–100% match for 16S in both samples) and *Rhodococcus fascians* (16S match 99.7%, sample 2 only) (Table 2); there were also matches with *Pseudomonas savastanoi* and *Erwinia herbicola*, but these matches were not confirmed by validation analyses (Table 2). *Agrobacterium tumefaciens* is a common and widespread bacterium responsible for formation of crown galls in the rhizosphere of various plants, but only strains containing a tumor-inducing plasmid (Ti plasmid, pTi) are virulent. We found no evidence for the presence of a complete Ti plasmid, especially its functionally important virulence genes *Vir*, as well as auxin synthesis (*iaaH*, *iaaM*) and cytokinin synthesis (*ipt*) genes^37^. Other genes that may be associated with tumor-inducement pathways and encoded on the Ti plasmid^38^ were only found in sample 1: nopaline permease ATP-binding protein gene and a substantial portion of Type IV secretion system components (Table 2). However, these genes may be encoded on other plasmid types occurring in non-virulent bacterial strains^39^. Because crucial components of the Ti plasmid that are functionally important for tumor-formation (*Vir* genes) are lacking and *A. tumefaciens* is commonly found on healthy plants, either externally^40^ or internally^41^, this bacterium probably does not use the classical Ti-plasmid inducement pathway in our system, but its role in gall formation using a different pathway cannot be completely excluded (see also TEM microscopy and FISH experiments below). *Rhodococcus* OTUs with 97.7% (sample 1) and 99.9% (sample 2) identity to *Rhodococcus fascians* had unequal 16S abundances (0.18 × 10^–6^ and 3.58 × 10^–6^ of all reads in samples 1 and 2, respectively). Given the very low abundance of *Rhodococcus* in both samples (Fig. 1e) and a 2.1% difference in their 16S rDNA genes, we believe that it is unlikely that this bacterium has a biologically important role in our system. The genus *Pseudomonas* can colonize a wide range of ecological niches^42^; as a plant pathogen it can cause tumorous overgrowths (knots), cankers, foliar necrosis, and bacterial blight^43–46^. Knot-inducing pathovars encode genes related to indole acetic acid, cytokinins, rhizobitoxine, bacteriophytochrome, and others^43,47^. Our Gall-ID analyses identified the 16S gene of *Pseudomonas savastanoi* in both samples, albeit with mismatches with the reference sequences (Table 2). Validation of these data via a separate BLAST search did not confirm the presence of *Pseudomonas savastanoi*; instead, two different species were identified, *P. yamanorum* (CP012400.2) and *P.* sp. DHXJ03 (JN244973.1) in samples 1 and 2, respectively (Table 2). None of these species are known to induce galls. Gall-ID also identified the *ISEhe3* insertion element of *Erwinia herbicola* in sample 1 only (Table 2). This bacterium is a widespread epiphyte on many different plants, also occurring in other habitats, such as seeds, water, humans, and animals^48^. Several plant tumorigenic strains of *E. herbicola* have been identified; all carry a pPATH pathogenicity plasmid encoding virulence genes^49^. *ISEhe3* and other insertion elements are also present on the plasmid of plant-pathogenic strains, which suggests that these elements could participate in the evolution of the pPATH plasmid^49^. Validation of these data yielded a 98.5% match with *Erwinia persicina*, a bacterium which is known to be plant pathogenic but not gall-inducing^50^. The *ISEhe3* insertion element was not detected in sample 2, indicating that *Erwinia* is probably not responsible for gall formation in our system.

Among the 10 most abundant genera identified by Kraken in each sample, only two were shared: *Wolbachia* and *Cutibacterium*. The latter genus was represented by *Cutibacterium acnes*. This bacterium is associated with the human skin, and is a widespread contaminant of DNA extraction kits^51^; we consider its presence as a likely artefact. With respect to the well-known gall-inducers discussed above, our analysis showed highly uneven or low abundances in samples 1 and 2: *Agrobacterium* (11.64, 0.18%)*, Pseudomonas* (64.23, 1.34%), *Rhodococcus* (0.06, 2.04%), *Erwinia* (0.04, 0.05%) (Supplementary Table S2; Fig. 1e). These data, therefore, agree with our conclusion that these bacteria (except probably *Agrobacterium*) do not play an important role in gall formation in our system (see above). Below, we briefly discuss several other bacterial genera from our samples that have gall-inducing species. A novel species of *Wolbachia* was common among the two samples, occurring at 16.7% (sample 1) or 9.7% (sample 2) of all bacterial reads (Fig. 1e; Supplementary Table S2). It has been hypothesized that *Wolbachia* is used by caterpillars of a leaf-mining moth to produce green islands in yellowing leaves, which act as sinks for nutrients^52^. Manipulation of cytokinin levels by the endosymbiotic bacterium was suggested as the cause of green-island formation^25,52^. However, the exact molecular mechanism is not known and co-phylogenetic evidence indicates that the correlation of *Wolbachia* and the ‘green-island’ phenotype is high but not absolute^53^. *Wolbachia* associated with root-feeding insects can lower plant defenses^54^, and mites may use this property to invade new host plants. *Xanthomonas* was found at low abundances, 0.4 and 1.7% of all bacterial reads in samples 1 and 2, respectively. This bacterium interacts with the host plant by using a type III secretion system (*T3SS*) to secrete an array of effector proteins. Virulence factors include lytic enzymes that attack the plant's cell wall, in addition to proteases, amylases, cellulases and lipases that help lower the plant’s defense mechanisms^55^. It would therefore be interesting to further explore whether eriophyoid mites can use associated bacteria, such as *Xanthomonas* or *Pseudomonas*, to suppress host plant defenses at early stages of plant colonization^55,56^, while bacteria can use mites to penetrate through the plant cell walls at the mite feeding site. Furthermore, the following four OTUs were identified by Kraken at low abundances, 0.00001–0.15%: *Paraburkholderia*, *Rhizobacter*, *Frankia*, *Phytoplasma* (sample 2 only) (Supplementary Table S4). These bacterial genera include gall-inducing species^57–61^, with *Phytoplasma* being unique as it can replicate intracellularly both in plants and their insect vector hosts, while other bacteria can replicate only in plant cells^61–63^. Based on their extremely low and uneven abundances, it is unlikely that these OTUs are responsible for gall formation in our system.

In addition to the above Gall-ID and Kraken analyses, we also ran SingleM (Fig. 3c) and assembly intersection analyses (Fig. 3d). These analyses returned similar but not identical results (Fig. 3c,d). First, unlike Gall-ID and Kraken, these analyses largely do not rely on existing taxonomy to identify OTUs shared across samples and, therefore, may be more accurate with respect to organisms having no sequence data in GenBank. Second, the differences can also be attributed to disparate underlying methodologies used by these analyses, data complexity, and the uneven coverages of the two datasets. For example, in Bacteria, rDNA may have multiple copies per genome (e.g., *Agrobacterium*), resulting in higher coverages, and therefore affecting both k-mer-based and assembly-based methods. The assembly of rDNA reads may also be positively biased due to rDNA sequence conservatism also affecting assembly-based methods. These issues are exaggerated if closely related species are present (e.g., *Pseudomonas*, *Agrobacterium* in our samples). Furthermore, k-mer-based (Kraken) and assembly-based methods may be affected by the presence of plasmids and mobile elements, which may have multiple copies in the genome (thus a species abundance can be overestimated) and may be shared across species (thus creating spurious classifications when based on reference sequence databases). Our SingleM analyses, relying on single-copy protein-coding genes, did not detect low abundance taxa, such as *Agrobacterium* and *Pseudomonas*, in sample 2 (Fig. 3c), while other analyses, using rDNA among other sequence data, were able to detect these taxa (Figs. 1e, 3d). In other words, differences between various metagenomic analyses conducted here are expected, and we consider our results to be complementary to each other.

A comparison of our metagenomic results, FISH and TEM microscopy suggests that *Wolbachia* was the only abundant OTU shared across the two samples. The substantial abundance of this bacterium points to a functional importance for its mite host. Some *Wolbachia* are known to be beneficial to nematode or insect hosts^64^ and it is likely that this is also the case here. *Wolbachia* is not known to induce galls but was suspected of manipulating cytokinin levels^25,52^ (see above). The *Fragariocoptes* endosymbiotic *Wolbachia* is a novel and very divergent species, with a substantial average nucleotide difference (20.7%) with respect to other known *Wolbachia*. For this reason, it may have unexpected properties, including gall formation. Additional experiments would be required to confirm this hypothesis. Our FISH experiments and the metagenomic analyses (sample 1) suggested the presence of *Agrobacterium tumefaciens* (morphotype 2), which is a major inducer of crown galls (Fig. 4b,d,e,g). This is also an unexpected result since its intimate association with arthropod hosts has not been documented in the literature so far, except for a single study that provided experimental evidence that this bacterium can be vectored by an insect^65^. Both FISH and TEM microscopy identified a rod-shaped endosymbiotic, extracellular bacterium that characteristically surrounds gigantic parenchymatic mite cells and congregates in intermuscular spaces, especially around salivary gland cells (Fig. 4d,e,g,h,k,l). The abundance of this bacterium and its characteristic distribution inside the mite indicate a strong biological association with the mite. Gall formation by this bacterium cannot be excluded with data at hand, and further work is needed to evaluate this possibility. Given the incomplete Ti plasmid and the substantial abundance of *Agrobacterium tumefaciens* in sample 1 (see above), we cautiously suggest that a role of this bacterium in gall formation in our system is unlikely and needs to be further evaluated. A similar conclusion of no bacterial involvement in gall formation has been recently made for insect gall inducers^66^.

In conclusion, here we use comparative metagenomics to test the hypothesis suggesting that a bacterial symbiont can be involved in gall formation in eriophyoid mites using two independent samples from the mite *Fragariocoptes setiger*. We found a novel bacterial species of *Wolbachia* shared across all analyzed samples of the gall-inducing mite. Another endosymbiotic extracellular, rod-shaped bacterium (morphotype 2, possibly *Agrobacterium tumefaciens*) was also detected, and based on its distribution inside the mite, it appears to form a biologically important association with the mite. Although we were able to demonstrate the presence of the two potential candidates, we suggest that it is unlikely they play a role in gall formation. In addition, we detected an array of plant pathogens that are associated with galls and may be vectored by the mite: *Xanthomonas campestris*, *Rhodococcus fascians*, *Rhodococcus* nr. *olei, Erwinia* nr. *persicina*, *Clavibacter michiganensis* (bacteria), *Albugo laibachii* (Oomycota), and Erysiphaceae (powdery mildews). Some mite-associated microorganisms (*Xanthomonas*, *Pseudomonas*, and *Albugo laibachii*) can use their host to penetrate through the plant cell walls at the mite feeding site. In return, these microorganisms could potentially help the mite to suppress host plant defenses at early stages of plant colonization. Furthermore, we found a mite pathogenic virus, *Betabaculovirus*, which is a double-stranded DNA virus, that may have a potential use in the control of agricultural pests.

## Methods

### Samples

Sample 1. Russia: Novgorod Prov., right bank of Luga river, nr. Maluy Volochek village, 58.486544 N 30.338933 E, galls on leaves of *Fragaria viridis*, 14 Sept. 2018, about 80 adult mites. Sample 2. Russia: Leningrad Prov., Luzhsky District, 184 m E Beloye Ozero [Beloye Lake], 58.808028 N, 30.487389 E, galls on leaves of *Fragaria viridis*, Aug 10 2019, about 1500 specimens obtained by alcohol washing. As offspring of the mite founder inside the gall have the ability to form galls^4^, new galls occur throughout the season. Collection of plant material was done with relevant institutional, national, and international guidelines and legislation. This collecting was done as part of cooperative agreement № 075-15-2020-922 (Ministry of Science and Higher Education of the Russian Federation) for a non-endangered, non-psychoactive species growing on public land outside of protected areas; no additional permissions and/or licenses are required for these samples as per paragraph 11 of the Forest Code of the Russian Federation (No. 200-FZ).

### Nucleic acid extraction and sequencing

DNA/RNA extraction and Illumina next generation sequencing were performed as detailed in Supplementary Methods.

### Genomic assembly, decontamination, annotation, phylogenomic analyses

Mite metagenomic assembly was done in MetaSPAdes v. 3.13.0^67^. It was decontaminated using MetaBat v. 2.12.1^68^, BLAST, and Diamond^69^, and annotated in Maker v.2.31.10^70^ using the mite transcriptome and Ecdysozoa UniProtKB/Swiss-Prot proteins for gene prediction. See detail in Supplementary Methods. The phylogenomic tree was inferred using amino acid sequence data in a Maximum Likelihood framework in IQ-TREE^71^ with alignment matrices prepared based on the BUSCO v.4 arachnida_odb10 database^72^ and custom utility scripts. See detail in Supplementary Methods.

### Gall-ID

For identification of gall-inducing bacteria in samples 1 and 2 using raw reads, we used SRST2^73^ and Gall-ID databases^74^, with the minimum gene coverage parameter set to 50% and maximum divergence parameter set to 10%: srst2 –input_pe $input_file_reads_forward $input_file_reads_reversed –max_divergence 10 –min_coverage 50 –log –output $out –gene_db $input_file_gene_db –threads $proc –report_all_consensus. Gall-ID databases have either functional genes known to be part of gall formation pathways or house-keeping genes (16S rDNA) that can be used to identify gall-inducing bacteria. Since the use of a majority rule consensus sequence (the Gall-ID default) is unreliable in the presence of multiple similar bacterial species, we also conducted validation of our Gall-ID results: (i) reads mapped on target genes in the Gall-ID databases were extracted (samtools fastq –1 forward.fq –2 reverse.fq –s singletons.fq –0 other.fq in.bam), (ii) and assembled in SPAdes (for PE reads: spades.py –1 forward.fq –2 reverse.fq -s singletons.fq –t $proc; for SE reads: spades.py –s $extracted.reads.fq –t $proc –k 127), (iii) SPAdes contigs were then classified by BLAST.

### K-mer-based metagenomic profiling

Taxonomic classification of raw reads was made by Kraken2 v.2.0.8^75^. A custom 35-mer database was built from six standard Kraken databases (archaea, bacteria, fungi, human, protozoa, and viral) plus the genomes of *Fragaria vesca* (GenBank accession GCF_000184155.1), *Albugo laibachii* Nc14 (GenBank BioProject accession: PRJEA53219), *Fragariocoptes setiger* (JAIFTH000000000, assembled here), *Wolbachia* endosymbiont of *Fragariocoptes setiger* (JAHRAF000000000, assembled here) and the Illumina PhiX technical sequence. Taxonomic classification was done with a confidence scoring threshold value of 0.1, which performed well in identifying Illumina PhiX technical sequences (not reported). In addition, this approach also substantially decreases the number of false positive classifications^76^. Using Kraken utilities scripts (KrakenTools), we converted standard Kraken report files to MetaPhlAn format and then combined these converted files as follows: kreport2mpa.py –r $kraken_report –o $kraken_report.mpa; combine_mpa.py –i kraken_report1.mpa,kraken_report2.mpa –o kraken.mpas.combined.txt. To estimate relative abundances, we also tried Bracken^77^, using the read length value as appropriate, 150 bp (sample 1) and 250 bp (sample 2). However, this analysis produced spurious results, e.g., the read proportion for Enterobacteriaceae were seemingly overestimated: 310.6 times (*Salmonella*) and 299.2 times (*Escherichia*) higher in sample 1 as compared to the Kraken data. Abundances of these taxa were also substantially overestimated in sample 2. Since these unusually high abundances were not supported by any other analyses (Kraken, singleM, BLAST, see below), we do not report Bracken analyses here.

Our initial Kraken analysis yielded a large number of OTUs, suggesting that many reads were probably overclassified^76^. For example, there was a total of 1,124 genera, including 975 bacterial genera. We believe that such a large diversity is biologically unrealistic and we used a combination of Kraken confidence filtering (0.1, see above) and an abundance cutoff (≥ 0.0338%) as suggested in the literature^76^. For comparison, we also ran an analysis with a lower abundance cutoff (≥ 0.0005%).

To calculate the taxonomic intersection (shared OTUs in samples 1 and 2), bacterial genera with a fraction of reads ≥ 0.0338% at least in one sample were selected, and then these data were used to create Venn diagrams (OTU counts) and abundance heatmaps. For Bacteria, this analysis yielded a total of 83 genera in both samples and 21 genera in the sample intersection, which we consider biologically meaningful, and so we present this as our main result. These data were clustered based on Euclidean distances and visualized as heatmaps in TBTools^78^. For comparison, abundance heatmaps were also generated for all organisms using a lower abundance cutoff value (≥ 0.0005%), yielding 171 classified genera.

### Identifying common OTUs across samples: read mapping on marker genes

We identified OTUs shared across the two samples based on mapping of raw reads on 14 marker, single-copy genes in SingleM^31^. This is an assembly-free, largely taxonomy-independent approach. The use of a subset of single-copy genes eliminates issues associated with copy-number variation in ribosomal genes (16S, 23S), plasmids, and transposable elements. The following commands were used to create OTU tables from both samples, combine them and cluster OTUs: singlem pipe –forward $f1.fq –reverse $f2.fq –otu_table $dna_tbl –threads $proc; singlem summarise –input_otu_tables $dna_tbl $rna_tbl –output_otu_table dna_rna.combined.otu_table.csv; singlem summarise –input_otu_tables dna_rna.combined.otu_table.csv –cluster –clustered_output_otu_table clustered.otu_table.csv.

Unique representative sequences (used as "OTUs" in SingleM) shared across the two samples were filtered. This dataset was used to construct a heatmap (see the next subsection), where: (i) percentages of the average read counts across the 14 marker genes were used for each OTU in both samples; (ii) gene count, which is indicative of the data completeness, was recorded and visualized on the heatmap.

### Identifying common OTUs across samples: Assembly intersection

Common OTUs present in the two NGS samples could also be identified via read assembly for each sample followed by assembly intersection. Intersection was done by standalone BLAST where sample 1 contigs were the query and sample 2 contigs were the subject. Matches having ≥ 98% similarity and bitscore ≥ 500 were then classified by BLAST. OTUs having ≥ 96% similarity with GenBank nucleotide (nt) database were classified at the species level, while all other matches were classified at the genus level (top hits were reported in case of multiple matches). This approach generated a subset of contigs shared between the two assemblies, and these contigs were identified by their sequence similarity (not by taxonomic labels). This methodology effectively minimizes the effect of high misclassification rates due to incompleteness of the GenBank databases^79^. For each contig, read-based coverage was calculated in bbduk (bbmap.sh ref = $ref in1 = $f1 in2 = $f2 covstats = covstats.txt), and the number of mapped reads per classified OTUs were recorded. To minimize the influence of rDNA (which may have multiple copies per genome), plasmids, and mobile/transposable elements (which may occur in multiple species), BLAST results were checked and edited. Final read count data were normalized by calculating read percentages. It is important to emphasize that this procedure was done only for OTUs present in the intersection (so these data can only be interpreted in the context of the subset of OTUs present in both samples). Then these data were log_2_-transformed, and a heatmap was constructed in TBtools v.1.0^78^. In this heatmap, each OTU was labeled with (i) an intersection bitscore, which is indicative of the magnitude of common matches across the two samples for a given OTU, and (ii) average percent identity with the closest GenBank matches. Clustering was done using Euclidean distances. We did this analysis using only bacterial taxa. Among fungi, some of which also can cause galls, we found only a single dominant taxon, the family Erysiphaceae (powdery mildews, which do not produce galls). Best matches were *Cystotheca wrightii* AB120747.1 (18S identity = 100% bitscore = 813) followed by *Podosphaera pannosa* AB525937.2 (rDNA identity = 99.88% bitscore = 1578) and *Podosphaera leucotricha* JAATOF010000279.1 (mt-DNA identity = 99.9%, bitscore = 5723).

### Phytohormones and horizontal gene transfer

The annotated mite assembly was searched for the following major plant/bacterial genes responsible for the production of phytohormones and enzymes involved in growth regulatory metabolism: 1-aminocyclopropane-1-carboxylic acid (ACC) deaminase, 2,3-butanediol, abscisic acid, acetoin, auxin, brassinosteroids, cytokinin, ethylene, gibberellic acid, gibberellin, indole, indole-3-acetic acid (IAA), jasmonate, jasmonic acid, salicylic acid, strigolactone^80–82^.

### TEM microscopy

All mites (including adults and immatures) from a single gall were fixed in 2.5% glutaraldehyde and 0.1 M cacodilate buffer (Sigma C0250) for four hours, washed with the same buffer, treated with 1% tetroxide osmium for 1 h, washed twice in distillated water, dehydrated in increasing ethanol series (30, 50,70, 96, 100%) and embedded in SPURR resin (Sigma EM0300-1KT). Thin sections were prepared using a Leica EM UC7 ultramicrotome, contrasted with saturated solution of uranyl acetate 20 min and lead citrate for 5 min, and photographed using a Jeol JEM-1400 electron microscope.

### Fluorescence in situ hybridization (FISH)

About 300 adult and 50 immature mites were used. We used three oligonucleotide probes, each with a distinct fluorophore label: Eub 338 5′-GCTGCCTCC CGTAGGAGT-3′ specific to Bacterial 16S rDNA gene (except for Planctomycetales и Verrucomicrobia), and two probes specific to *Agrobacterium tumefaciens*: 16S.1722F.Agr.tum 5′-TGTCCTTCAGTTAGGCTGGC-3′ and 16S.907F.Agr.tum 5′-AATTAATACCGCATACGCCC-3′. Two fluorescent labels were used: CY3 (indocarocyanine 3) and FITC (5(6)-Carboxyfluorescein); DAPI (Invitrogen) was used for no probe control experiments. See additional detail in Supplementary Methods.

### Data submitted to GenBank

*Fragariocoptes setiger.* Genomic assembly. GenBank BioSample id: SAMN13972306, accession: JAIFTH000000000.

Mite-associated organisms, metagenomic assembly. GenBank BioSample id: SAMN13981716, accession: JAALJN000000000.

Mite and associated organisms, metagenome, Illumina short reads. Short read archive (SRA) BioSample id: SAMN13981716, run selector: SRR11015813.

Mite and associated organisms, metatranscriptome, Illumina short reads. Short read archive (SRA) BioSample id: SAMN13991554, run selector: SRR11026779.

*Osperalycus tenerphagus* AD1672, genomic assembly. DDBJ/ENA/GenBank accession JAGGCA000000000.

*Speleorchestes* sp. AD1671, genomic assembly. DDBJ/ENA/GenBank accession number JAGHQN000000000.

*Wolbachia* endosymbiont of *Fragariocoptes setiger.* Genomic assembly. GenBank accession: JAHRAF000000000, BioSample id: SAMN19370650.

## Supplementary Information

Supplementary Information 1.

Supplementary Figure S1.

Supplementary Figure S2.

Supplementary Information 2.

Supplementary Information 3.

Supplementary Information 4.

Supplementary Table S1.

Supplementary Table S2.

Supplementary Table S3.

Supplementary Table S4.

Supplementary Table S5.
