# Author Correction: Symbiotic bacteria of the gall-inducing mite *Fragariocoptes setiger* (Eriophyoidea) and phylogenomic resolution of the eriophyoid position among Acari

**DOI:** 10.1038/s41598-022-10467-7

**Published:** 2022-04-15

**Authors:** Pavel B. Klimov, Philipp E. Chetverikov, Irina E. Dodueva, Andrey E. Vishnyakov, Samuel J. Bolton, Svetlana S. Paponova, Ljudmila A. Lutova, Andrey V. Tolstikov

**Affiliations:** 1 X-BIO Institute, Tyumen State University, Tyumen, Russia 625003; 2 Saint-Petersburg State University, St. Petersburg, Russia 199034; 3 Florida Department of Agriculture and Consumer Services, Gainesville, FL USA

Correction to: *Scientific Reports* 10.1038/s41598-022-07535-3, published online 09 March 2022

The original version of this Article contained errors.

In Figure 2, the symbol for 100/100 branch support was omitted from the key. The original version of Figure 2 and its legend is included below.

**Figure 2:**
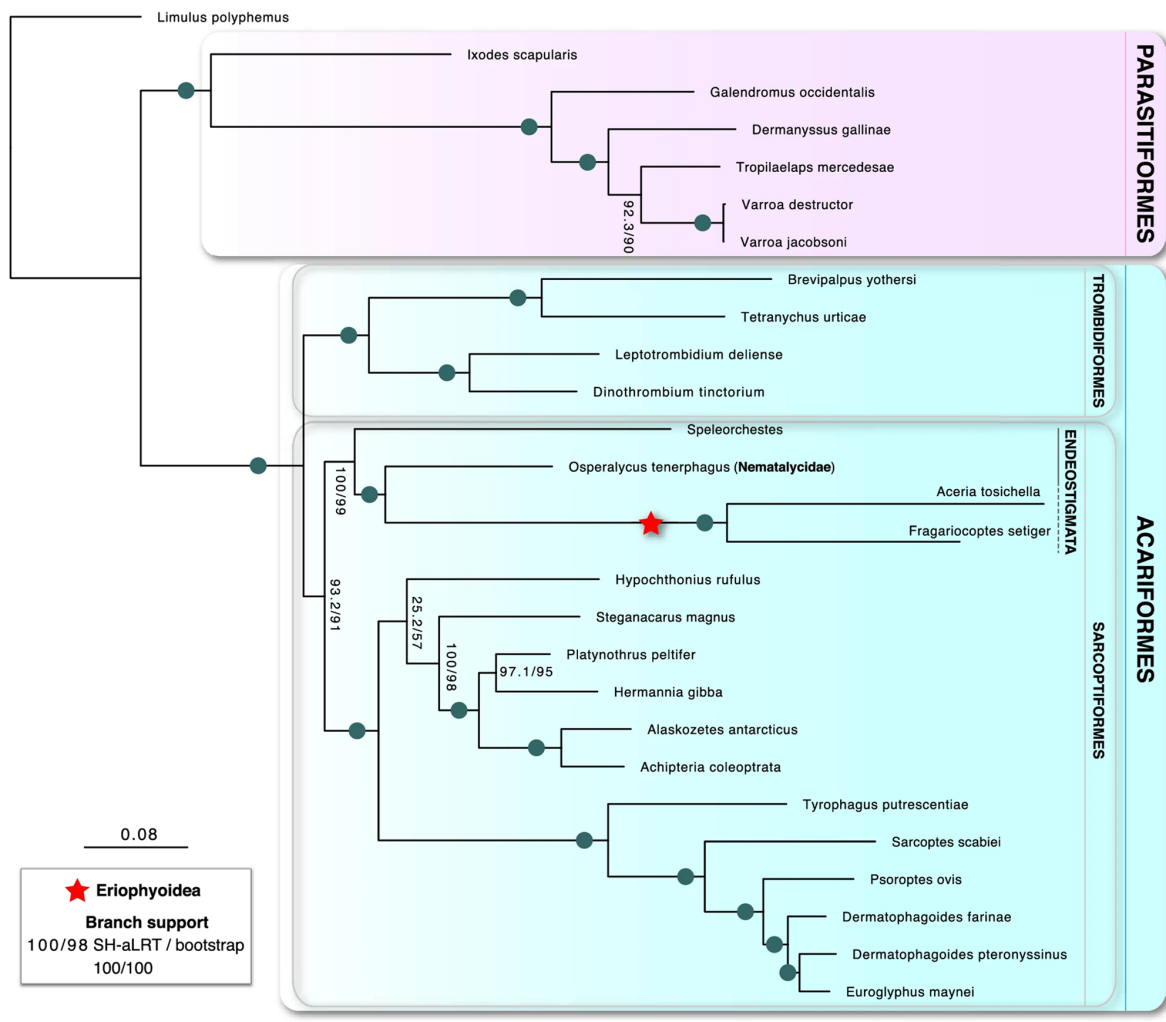
Relationships of parasitiform and acariform mites. Phylogenomic inference was undertaken using a Maximum likelihood framework in IQ-TREE based on 90 orthologous proteins.

In addition, the Acknowledgments,

“We thank Dr. Jeff Chang and Dr. Alexandra Weisberg (Oregon State University) for providing all databases used in the Vir-Search web service [http://gall-id.cgrb.oregonstate.edu/vir-finder.html], Sergey Diachkov (Tyumen State University, Russia) for help with the Gall-ID analysis and several GenBank submissions, and D. Hans Klompen (Ohio State University) who provided sequences of the two endeostigmatid mites.”

now reads:

“We thank Dr. Jeff Chang and Dr. Alexandra Weisberg (Oregon State University) for providing all databases used in the Vir-Search web service [http://gall-id.cgrb.oregonstate.edu/vir-finder.html], Sergey Diachkov (Tyumen State University, Russia) for help with the Gall-ID analysis and several GenBank submissions, and Dr. Hans Klompen (Ohio State University) who provided sequences of the two endeostigmatid mites.”

The original Article has been corrected.

