# Supplementary material for: Symbiotic bacteria of the gall-inducing mite Fragariocoptes setiger (Eriophyoidea) and phylogenomic resolution of the eriophyoid position among Acari
Source: Sci Rep. 2022 Mar 9;12:3811. doi: 10.1038/s41598-022-07535-3 (PMC8907322; doi:10.1038/s41598-022-07535-3)
Supplement: Supplementary file 1 — Supplementary Information 1. [file 41598_2022_7535_MOESM1_ESM.docx]

**Supplementary Methods**

**DNA extraction and sequencing.** Genomic DNA isolation was done from a pulled sample of 80 mite specimens, using a QIAamp DNA Mini Kit (QIAGEN) with the manufacturer's instructions for tissue samples. Whole genome library preparation for NGS sequencing was done by the University of Michigan Sequencing Core with a SMARTer® ThruPLEX® DNA-Seq Kit (Takara Bio). The average insert size was 542bp; the adapter sequences (with PCR primers and index sequences) were as follows: adapter1 AGATCGGAAGAGCACACGTCTGAACTCCAGTCACTCGACGTCATCTCGTATGCCGTCTTCTGCTTG; adapter2 AGATCGGAAGAGCGTCGTGTAGGGAAAGAGTGTTACTCCTTGTGTAGATCTCGGTGGTCGCCGTATCATT. Sequencing was done on an Illumina HiSeq-4000 instrument. Due to a technical issue, our first Illumina run ended 5 cycles earlier, generating 364,040,014 PE 2x145 bp reads; another run was complete, generating 346,816,650 2x150 bp reads. Both runs were used in the downstream analyses.

RNA extraction and sequencing: About 1500 specimens of mites were obtained by placing galls (opened with a sterile needle) in ethanol, agitating the sample, and then, after a few minutes, collecting the clean mite fraction at the bottom. Total RNA was extracted from pulled mite specimens using a Trizol kit (Thermo Fisher Scientific) according to the manufacturer's instructions. DNA quality was checked on a BioAnalyser instrument using an RNA 6000 Nano Kit (Agilent). PolyA RNA was purified with a Dynabeads® mRNA Purification Kit (Ambion). Illumina library was made using a polyA NEBNext® Ultra™ II RNA Library Prep (NEB) according to the manufacturer's instructions. The adapter sequence (with PCR primer and index) sequences was as follows: GATCGGAAGAGCACACGTCTGAACTCCAGTCACGGCTACATCTCGTATGCCGTCTTCTGCTTG. Sequencing was done on an Illumina MiSeq-1500 instrument, generating 82,596,682 SE 1x 250bp reads.

**Assembly.** For optical deduplication, we used BBtools v.38.34 (https://sourceforge.net/projects/bbmap/) (clumpify.sh dedupe optical), adapter and quality trimming (bbduk.sh ktrim=r k=23 mink=11 hdist=1 qtrim=rl trimq=10 tbo tpe minlen=70 ref=adapters.fa ftm=5). This procedure generated 344,846,696 metagenomic PE reads (average quality score Q = 37.18, total reads with ≥Q30 = 91.96%, average length = 148.94 bp) and 363,200,118 PE reads (Q=36.62, reads ≥Q30 = 92.33%, average length =144.19bp) for the complete and incomplete Illumina runs, respectively; singletons were removed. Read quality and adapter contents were evaluated in FastQC ^1^ before and after the deduplication / trimming procedure. Processed reads from the two Illumina runs were assembled in MetaSPAdes v. 3.13.0 ^2^ with three k-mer sizes: 21, 33, 55. Assembled metagenomic contigs were blasted against the NCBI nucleotide (nt) database using a local BLAST v. 2.7.1+ ^3^. Assembly quality statistics are given in Table 1. For metatranscriptomic assembly, deduplication, adapter and quality trimming, quality assessment, and BLAST searches were done as above. A total of 82,009,061 SE reads (Q = 37.11; reads with ≥Q30 = 97.49%, average length = 201.98 bp) were assembled in rnaSPAdes v. 3.13.0 ^4^ with k-mer sizes=55, 123). Quality assessment of the mite metatranscriptome (soft filtered contigs) was done in rnaQUAST v.1.5.1^5^ using the 'clean' mite genome as the reference (see below).

**Phylogenomic analyses.** Phylogenomic relationships of *Fragariocoptes setiger* among other mites were inferred as follows:

(i) A total of 27 chelicerate genomes were used (Table 2): 21 were assembled genomes from GenBank; 3 genomes were assembled *de novo* by us; and for 3 species, we assembled short transcriptomic reads from the SRA database in Trinity ^6^ using the following command: $Trinity --seqType fq --samples_file samples.txt --max_memory 128G --CPU $proc --trimmomatic --quality_trimming_params "ILLUMINACLIP:TruSeq3-PE.fa:2:30:10 SLIDINGWINDOW:4:5 LEADING:5 TRAILING:5 MINLEN:25 AVGQUAL:20".(ii) BUSCO v.4 with the arachnida_odb10 database ^7^ was used to recover a subset of 2934 orthologous genes from the 27 assemblies with the following command: $busco --in $assembly.fa --lineage arachnida_odb10 -o busco_out_$assembly.fa --mode geno --cpu $proc.

(iii) Existing phylogenetic scripts (https://github.com/JLSteenwyk/Phylogenetic_scripts) were used to extract and concatenate loci found by BUSCO. Because these scripts work only with the old BUSCO v.3 directory structure, we used the standard Unix symbolic link command (ln) to make our BUSCO v.4 output compatible with these scripts. Gene occupancy was set to 0.8. Amino acid sequence alignment was done in mafft v.7.429 ^8^ and alignment trimming was done with trimAl v.1.4 ^9^. All these commands were executed using a custom Unix shell script (Supplementary Data S1).

(iv) Concatenated matrix of BUSCO loci was manually checked. Divergent amino acid ends were trimmed and bacterial contaminants from GenBank sequences were removed. The raw and edited matrices are available (Supplementary Data S2, Supplementary Data S3).

(v) Inferring models of amino acid evolution, best partitioning scheme, and Maximum Likelihood phylogenetic analysis with branch support estimated by a Shimodaira and Hasegawa-like approximate likelihood ratio test (SH-aLRT) and ultrafast bootstrap (1000 replicates) were done in IQ-TREE ^10^: $iqtree -s partitioned_aa_matrix.nex -spp concat.partition -nt 16 -m MFP+MERGE -rcluster 10 -alrt 1000 -bb 1000 -safe. Two independent phylogenetic analyses were run, with the raw and curated matrix (see step iv).

**Mite genome decontamination.** MetaBat v. 2.12.1^11^ was used for draft metagenomic assembly binning (min contigs size 1500nt). Identification of the bins was done using BLAST. Bins with many mite hits, Eukaryote-only hits, or no hits were classified as mite (coverage 1,941-6,102). All other bins had a lower coverage, i.e *Wolbachia* with coverage of nearly 1,200). The mite genome was recovered as follows:

1. Nucleotide BLAST was done on the assembly. Hits on eukaryotic genomes with substantial bacterial contamination were removed. Hits with low identity (<96%) and bitscore (<500) were removed. Only contigs with a length ≥ 500 bp and coverage of >100 were considered.

2. Five bins were created: *Mammalia*, *Spermatophyta*, *Bacteria*+*Viruses*, *Fungi* and *unclassified (mite)*. For the former four bins, contigs was classified if all BLAST hits were exclusively within that lineage, otherwise contigs were placed into the *unclassified (mite)* bin. Contigs without any BLAST hits were tentatively placed into the latter bin as well.

3. For the *Mammalia*, *Spermatophyta*, *Bacteria*+*Viruses*, *Fungi* bins, all contigs with a coverage >1000x were verified by classification against a protein database in Diamond ^12^ using the following criteria: identity ≥50%, bitscore ≥150, alignment length ≥50% of the contig length.

4. Contigs from the *Unclassified (mite)* bin were verified by classification against a protein database in Diamond. Hits were considered reliable when the following criteria were met: identity ≥50, bitscore ≥150, alignment length ≥50% of the contig length.

**Mite genome annotation.** *Mitochondrial* genome. Three mitochondrial contigs were identified by their high coverage (136,933-161,465) and BLAST searches; these contigs were joined, and then annotated as follows: (i) MITOS server v.2 ^13^ was used to create a draft annotation; (ii) Gene boundaries for protein-coding were verified based on a comparative analysis in Mesquite ^14^ using sequences of *Tetranychus urticae* (EU345430.1) and three eriophyoid species: *Epitrimerus sabinae* (NC_029208.1), *Rhinotergum shaoguanense* (NC_034150.1), *Phyllocoptes taishanensis* (NC_029209.1); (iii) The presence of non-standard tRNAs, especially between putative protein-coding genes, was checked in ARWEN v1.2.3 ^15^ as follows: -l -seq -gcinvert -br -ps80.

*Nuclear genome*. The single ribosomal contig was identified by its high coverage (210,957) and BLAST hits and then annotated manually. All contigs which are not rDNA and mitochondrial DNA were annotated in Maker v.2.31.10 ^16^ bundled with Repeat Masker (RepBaseEdition-20181026) with the default parameters. Structural annotation was made in three steps: (i) Hidden Markov models were created based on our assembled transcriptome (RNA soft filtered transcripts) and UniProtKB / Swiss-Prot proteins for Ecdysozoa (est2genoime = 1; protein2genome = 1); (ii) imperfect gene models generated in step 1 were then used to train the gene prediction program SNAP ^17^ (est2genoime =0; protein2genome = 0; snaphmm=snap_1st_round.hmm); (iii) final SNAP annotation was done using gene models from the second step (est2genoime=0; protein2genome = 0; snaphmm=snap_2nd_round.hmm). Functional annotation was made in InterProScan v.5.38-76.0 ^18,19^ and BLAST v.2.7.1+ using the curated Swiss-Prot database. These results were then assigned to the maker annotation using Maker accessory functions.

**Fluorescence *in situ* hybridization (FISH).** Live mites were extracted from galls (sample 1, see above) with a sterile needle, washed to remove surface contaminants, placed in 96% ethanol for 2 hrs, and then fixed in 4% PFA in1х PBS for 12 hrs. After fixation, mites were washed in 1x PBS 3 times for 20 mins. DNA hybridization experiments were done in glass-covered, humidity-controlled chambers (Stender dish) in a thermal shaker Elmi ST-3L. We used three oligonucleotide probes with distinct fluorophore labels for each hybridization probe: Eub 338 5'-GCT GCC TCC CGT AGG AGT-3' specific for the domain Bacteria (except for Planctomycetales и Verrucomicrobia); 16S.1722F.Agr.tum 5'-TGT CCT TCA GTT AGG CTG GC-3' and 16S.907F.Agr.tum 5'-AAT TAA TAC CGC ATA CGC CC-3' both designed using the *Agrobacterium tumefaciens* 16S rDNA recovered by our metagenomic analysis and GenBank data. A BLAST search of 16S.1722F.Agr.tum and 16S.907F.Agr.tum confirmed their high specificity to *Agrobacterium*. Of these *Agrobacterium* probes, the former (16S.1722F.Agr.tum) yielded a stronger fluorescence and is therefore reported here.

For FISH hybridization experiments, we used two fluorescent labels, CY3 (indocarocyanine 3) and FITC (5(6)-Carboxyfluorescein), and the three probes described above. FISH hybridization was done in a 30% formamide solution (5М NaCl – 360 µL, 1М Tris/HCl – 40 µL, formamide – 600 µL, dH2 – 998 µL, 10% SDS – 2 µL; for a total volume of 2 mL) at 46C for 3 hrs. Washing was done in the wash buffer (5М NaCl – 41 µL, 1М Tris/HCl – 40 µl, dH2 – 1897 µL, 10% SDS – 2 µL; 0.5M EDTA – 20 µL for a total volume of 2 mL) 3 times, for 20 mins at 48C. For confocal microscope observations (control with no probes), samples were embedded in Prolong Diamond Antifade Mountant with DAPI (Invitrogen).

Confocal Laser Scanning Microscopy (CLSM) acquisition was done using a Spectral confocal & multiphoton system Leica TCS SP2 with an objective 63x N.A. 1.4–0.60 Oil lBL HCX PL APO at an excitation wavelength of 532 nm (green laser) at 90% intensity, 488 nm (light blue laser) at 85% intensity and 405 nm (blue laser) at 15% intensity. Acquisition resolution was 1024x1024 pixels, level of gain 600–950, frame average from 1 to 2, and the zoom range 1.5–2.5x. Recorded stacks of optical slices were processed in ImageJ. Maximum intensity projections (MIP) images were obtained to visualize stained bacteria.

**Sample 1 field note.** This sample had the following note: about 20% of galls had an unidentified, presumably phytopathogenic powdery mildew fungus (Erysiphaceae) inside, which mycelium gave the gall a fluffy appearance on the inside. Light microscopic observations identified articulate hyphae of probably a predatory/parasitic fungus in nearly 5% of mites. Singular barrel-shaped fungal spores and small prokaryotic cells were observed on the mite surface between the opisthosomal annuli.

**Supplementary Information**

Supplementary Table S1. Quality assessment of the mite metatranscriptome (soft filtered contigs) vs mite 'clean' genome in rnaQUAST.

Supplementary Table S2. Taxonomic classification of Bacteria in two metagenomic samples of the gall-inducing mite *Fragariocoptes setiger* in Kraken2. There was a total of 49,514,852 and 3,830,185 classified bacterial reads in samples 1 and 2, respectively. OTUs (genera) were filtered based on a normalized abundance threshold of ≥0.0338% in either sample, resulting in 83 OTUs (genera) represented by 46,906,755 and 1,895,438 reads (sample 1 and 2, respectively). Sample intersection (highlighted) is defined as OTUs with abundances satisfying the threshold in both samples. These data were visualized as abundance heatmaps: 83-OTU full dataset (Supplementary Fig. S1) and 21-OTUs present in the intersection (Fig. 1d,e).

Supplementary Table S3. Taxonomic classification of two metagenomic samples of the gall-inducing mite *Fragariocoptes setiger* in Kraken2. There was a total of 708,046,814 and 82,009,061 reads in samples 1 and 2, respectively. OTUs (genera) were filtered based on a normalized abundance threshold of ≥0.0005% in either sample, resulting in 171 OTUs represented by 670,717,361 and 72,439,919 reads (sample 1 and 2, respectively). These data were visualized as abundance heatmap in Supplementary Fig. S2.

Supplementary Table S4. Taxonomic classification and abundance estimate for two metagenomic samples of the gall-inducing mite *Fragariocoptes setiger* in Kraken2. There was a total of 708,046,814 and 82,009,061 reads in samples 1 and 2, respectively. These data were used to generate Supplementary Fig. S1 and Supplementary Fig. S2, Supplementary Table S2 and Supplementary Table S3.

Supplementary Table S5. Taxonomic profiling and read mapping statistics for two metagenomic samples of the gall-inducing mite *Fragariocoptes setiger* in SingleM. 14 standard conserved, single-copy genes (SingleM default) were used as mapping references. OTUs were treated as short representative sequences (rather than taxonomic labels) and then clustered. After the clustering step, taxonomic classification was assigned. These data were used to generate Fig. 3a.

Supplementary Fig. S1. Taxonomic classification of Bacteria in the two metagenomic samples of the gall-inducing mite *Fragariocoptes setiger* in Kraken2. There was a total of 49,514,852 and 3,830,185 classified bacterial reads in samples 1 and 2, respectively. OTUs (genera) were filtered based on a normalized abundance threshold of ≥0.0338% in either sample, resulting in 83 OTUs (genera) represented by 46,906,755 and 1,895,438 reads (sample 1 and 2, respectively). Data are given in Supplementary Table S2.

Supplementary Fig. S2. Taxonomic classification of two metagenomic samples of the gall-inducing mite *Fragariocoptes setiger* in Kraken2. There was a total of 708,046,814 and 82,009,061 classified reads in samples 1 and 2, respectively. OTUs (genera) were filtered based on a normalized abundance threshold of ≥0.0005% in either sample, resulting in 171 OTUs represented by 670,717,361 and 72,439,919 reads (sample 1 and 2, respectively). Data are given in Supplementary Table S3.

Supplementary Data S1. Unix shell script used to generate a phylogenomic matrix for 27 chelicerate genomes.

Supplementary Data S2. Concatenated phylogenomic matrix for 27 chelicerate genomes. Data are presented as amino acid alignments in fasta format; IQ-TREE-compatible partitions are given.

Supplementary Data S3. Curated concatenated phylogenomic matrix for 27 chelicerate genomes. Divergent amino acid ends were manually trimmed and bacterial contaminants from GenBank sequences were removed. This is a nexus file generated by Mesquite v.3.61(build 927). This file was used to infer the phylogenetic relationships of mites (Fig. 2).

**Supplementary References**

1 Andrews, S. FastQC: a quality control tool for high throughput sequence data. Available online at: <http://www.bioinformatics.babraham.ac.uk/projects/fastqc>. (2018).

2 Nurk, S., Meleshko, D., Korobeynikov, A. & Pevzner, P. A. metaSPAdes: a new versatile metagenomic assembler. *Genome Res.* **27**, 824-834, doi:10.1101/gr.213959.116 (2017).

3 Altschul, S. F. *et al.* Gapped BLAST and PSI-BLAST: a new generation of protein database search programs. *Nucleic Acids Res.* **25**, 3389-3402 (1997).

4 Bushmanova, E., Antipov, D., Lapidus, A. & Prjibelski, A. D. rnaSPAdes: a *de novo* transcriptome assembler and its application to RNA-Seq data. *Gigascience* **8**, doi:10.1093/gigascience/giz100 (2019).

5 Bushmanova, E., Antipov, D., Lapidus, A., Suvorov, V. & Prjibelski, A. D. rnaQUAST: a quality assessment tool for *de novo* transcriptome assemblies. *Bioinformatics* **32**, 2210-2212, doi:10.1093/bioinformatics/btw218 (2016).

6 Grabherr, M. G. *et al.* Full-length transcriptome assembly from RNA-Seq data without a reference genome. *Nat. Biotechnol.* **29**, 644-652, doi:10.1038/nbt.1883 (2011).

7 Seppey, M., Manni, M. & Zdobnov, E. M. BUSCO: assessing genome assembly and annotation completeness. *Methods Mol. Biol.* **1962**, 227-245, doi:10.1007/978-1-4939-9173-0_14 (2019).

8 Katoh, K. & Standley, D. M. MAFFT multiple sequence alignment software version 7: improvements in performance and usability. *Mol. Biol. Evol.* **30**, 772-780, doi:10.1093/molbev/mst010 (2013).

9 Capella-Gutierrez, S., Silla-Martinez, J. M. & Gabaldon, T. trimAl: a tool for automated alignment trimming in large-scale phylogenetic analyses. *Bioinformatics* **25**, 1972-1973, doi:10.1093/bioinformatics/btp348 (2009).

10 Nguyen, L. T., Schmidt, H. A., von Haeseler, A. & Minh, B. Q. IQ-TREE: a fast and effective stochastic algorithm for estimating maximum-likelihood phylogenies. *Mol. Biol. Evol.* **32**, 268-274, doi:10.1093/molbev/msu300 (2015).

11 Kang, D. D., Froula, J., Egan, R. & Wang, Z. MetaBAT, an efficient tool for accurately reconstructing single genomes from complex microbial communities. *PeerJ* **3**, e1165, doi:10.7717/peerj.1165 (2015).

12 Buchfink, B., Xie, C. & Huson, D. H. Fast and sensitive protein alignment using DIAMOND. *Nature Methods* **12**, 59-60, doi:10.1038/nmeth.3176 (2015).

13 Bernt, M. *et al.* MITOS: improved *de novo* metazoan mitochondrial genome annotation. *Mol. Phylogenet. Evol.* **69**, 313-319, doi:10.1016/j.ympev.2012.08.023 (2013).

14 Maddison, W. P. & Maddison, D. R. *Mesquite: a modular system for evolutionary analysis. Version 3.10* [*http://mesquiteproject.org*](http://mesquiteproject.org). (2016).

15 Laslett, D. & Canback, B. ARWEN: a program to detect tRNA genes in metazoan mitochondrial nucleotide sequences. *Bioinformatics* **24**, 172-175, doi:10.1093/bioinformatics/btm573 (2008).

16 Cantarel, B. L. *et al.* MAKER: an easy-to-use annotation pipeline designed for emerging model organism genomes. *Genome Res.* **18**, 188-196, doi:10.1101/gr.6743907 (2008).

17 Korf, I. Gene finding in novel genomes. *BMC Bioinformatics* **5**, doi:10.1186/1471-2105-5-59 (2004).

18 Zdobnov, E. M. & Apweiler, R. InterProScan--an integration platform for the signature-recognition methods in InterPro. *Bioinformatics* **17**, 847-848, doi:10.1093/bioinformatics/17.9.847 (2001).

19 Jones, P. *et al.* InterProScan 5: genome-scale protein function classification. *Bioinformatics* **30**, 1236-1240, doi:10.1093/bioinformatics/btu031 (2014).
