# Supplementary figures and images for: Symbiotic bacteria of the gall-inducing mite Fragariocoptes setiger (Eriophyoidea) and phylogenomic resolution of the eriophyoid position among Acari
Source: Sci Rep. 2022 Mar 9;12:3811. doi: 10.1038/s41598-022-07535-3 (PMC8907322; doi:10.1038/s41598-022-07535-3)

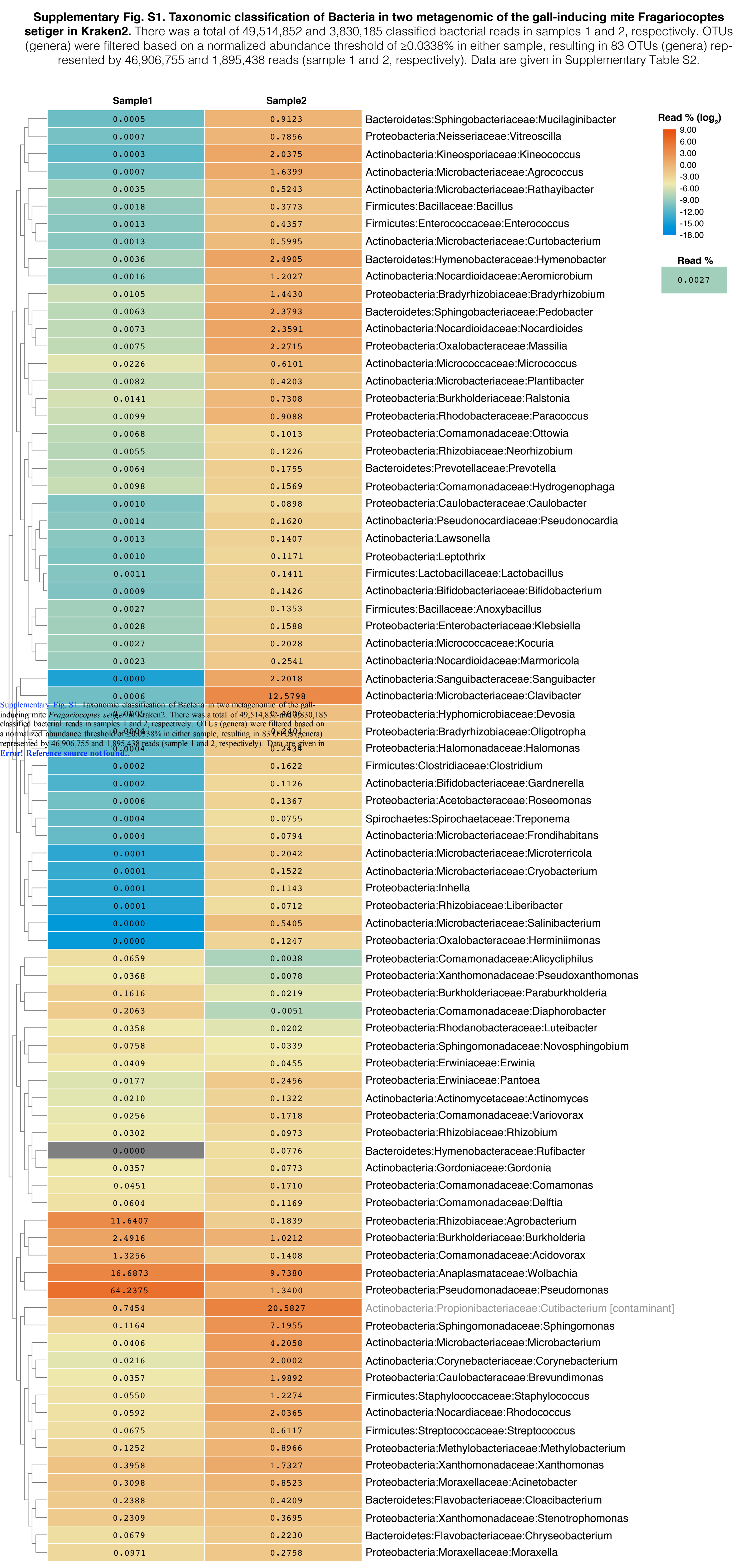

Supplement: Supplementary file 2 — Supplementary Figure S1. [file 41598_2022_7535_MOESM2_ESM.pdf]
