# Supplementary material for: Symbiotic bacteria of the gall-inducing mite Fragariocoptes setiger (Eriophyoidea) and phylogenomic resolution of the eriophyoid position among Acari
Source: Sci Rep. 2022 Mar 9;12:3811. doi: 10.1038/s41598-022-07535-3 (PMC8907322; doi:10.1038/s41598-022-07535-3)
Supplement: Supplementary file 3 — Supplementary Figure S2. [file 41598_2022_7535_MOESM3_ESM.pdf]

Supplementary Fig. S2. Taxonomic classification of two metagenomic samples of the gall-inducing mite Fragariocoptes setiger in Kraken2. There was a total of 708,046,814 and 82,009,061 classified reads in samples 1 and 2, respectively. OTUs (genera) were filtered based on a normalized abundance threshold of ≥0.0005% in either sample, resulting in 171 OTUs represented by 670,717,361 and 72,439,919 reads (sample 1 and 2, respectively). Data are given in Supplementary Table S3.

Read % (log_2): 10.00, 5.00, 0.00, -5.00, -10.00, -15.00, -20.00, -25.00

Read %: 0.000692

Sample1	Sample2	
0.000005	0.002990	Bacteria:Proteobacteria:Inhella
0.000005	0.005342	Bacteria:Actinobacteria:Microbacteriaceae:Microterricola
0.000006	0.003983	Bacteria:Actinobacteria:Microbacteriaceae:Cryobacterium
0.000012	0.006576	Eukaryota:Ascomycota:Mycosphaerellaceae:Cercospora
0.000013	0.002947	Bacteria:Actinobacteria:Bifidobacteriaceae:Gardnerella
0.000017	0.003555	Eukaryota:Ascomycota:Chaetomiaceae:Thielavia
0.000014	0.004244	Bacteria:Firmicutes:Clostridiaceae:Clostridium
0.000010	0.001699	Bacteria:Proteobacteria:Caulobacteraceae:Phenylobacterium
0.000012	0.001183	Bacteria:Actinobacteria:Microbacteriaceae:Leifsonia
0.000009	0.001144	Bacteria:Proteobacteria:Desulfovibrionaceae:Desulfovibrio
0.000016	0.000777	Bacteria:Firmicutes:Leuconostocaceae:Weissella
0.000014	0.000761	Bacteria:Cyanobacteria:Oscillatoriaceae:Oscillatoria
0.000013	0.000683	Bacteria:Proteobacteria:Methylobacteriaceae:Microvirga
0.000011	0.000578	Bacteria:Fusobacteria:Leptotrichiaceae:Leptotrichia
0.000009	0.000842	Bacteria:Firmicutes:Paenibacillaceae:Paenibacillus
0.000005	0.000581	Bacteria:Firmicutes:Bacillaceae:Aeribacillus
0.000003	0.000625	Bacteria:Firmicutes:Bacillaceae:Parageobacillus
0.000003	0.000605	Bacteria:Proteobacteria:Chromatiaceae:Rheinheimera
0.000007	0.001273	Bacteria:Proteobacteria:Alcaligenaceae:Pigmentiphaga
0.000006	0.001628	Bacteria:Proteobacteria:Phyllobacteriaceae:Hoeflea
0.000004	0.001154	Eukaryota:Ascomycota:Trichomonascaceae:Sugiyamaella
0.000005	0.001006	Bacteria:Actinobacteria:Beutenbergiaceae:Miniimonas
0.000005	0.000907	Bacteria:Actinobacteria:Cellulomonadaceae:Cellulomonas
0.000000	0.003676	Eukaryota:Ascomycota:Pichiaceae:Pichia
0.000001	0.007728	Eukaryota:Ascomycota:Schizosaccharomycetaceae:Schizosaccharomyces
0.000001	0.014143	Bacteria:Actinobacteria:Microbacteriaceae:Salinibacterium
0.000001	0.001241	Eukaryota:Bacillariophyta:Phaeodactylaceae:Phaeodactylum
0.000000	0.000584	Bacteria:Bacteroidetes:Flavobacteriaceae:Mariniflexile
0.000001	0.000580	Bacteria:Bacteroidetes:Chitinophagaceae:Flavisolibacter
0.000001	0.000816	Bacteria:Actinobacteria:Microbacteriaceae:Aurantimicrobium
0.000001	0.003262	Bacteria:Proteobacteria:Oxalobacteraceae:Herminiimonas
0.000002	0.001031	Bacteria:Bacteroidetes:Chitinophagaceae:Arachidicoccus
0.000002	0.001093	Bacteria:Proteobacteria:Alcaligenaceae:Advenella
0.000003	0.001630	Bacteria:Proteobacteria:Comamonadaceae:Candidatus_Symbiobacter
0.000004	0.001864	Bacteria:Proteobacteria:Rhizobiaceae:Liberibacter
0.000003	0.057612	Bacteria:Actinobacteria:Sanguibacteraceae:Sanguibacter
0.000006	0.025331	Eukaryota:Basidiomycota:Cryptococcaceae:Cryptococcus
0.000013	0.026676	Eukaryota:Ascomycota:Mycosphaerellaceae:Zymoseptoria
0.000042	0.329160	Bacteria:Actinobacteria:Microbacteriaceae:Clavibacter
0.000037	0.023872	Bacteria:Bacteroidetes:Sphingobacteriaceae:Mucilaginibacter
0.000050	0.020555	Bacteria:Proteobacteria:Neisseriaceae:Vitreoscilla
0.000024	0.053313	Bacteria:Actinobacteria:Kineosporiaceae:Kineococcus
0.000046	0.042909	Bacteria:Actinobacteria:Microbacteriaceae:Agrococcus
0.000247	0.013719	Bacteria:Actinobacteria:Microbacteriaceae:Rathayibacter
0.000128	0.009872	Bacteria:Firmicutes:Bacillaceae:Bacillus
0.000093	0.011401	Bacteria:Firmicutes:Enterococcaceae:Enterococcus
0.000088	0.015687	Bacteria:Actinobacteria:Microbacteriaceae:Curtobacterium
0.000033	0.012053	Bacteria:Proteobacteria:Hyphomicrobiaceae:Devosia
0.000028	0.006281	Bacteria:Proteobacteria:Bradyrhizobiaceae:Oligotropha
0.000026	0.006369	Bacteria:Proteobacteria:Halomonadaceae:Halomonas
0.000108	0.003578	Eukaryota:Apicomplexa:Plasmodiidae:Plasmodium
0.000095	0.004239	Bacteria:Actinobacteria:Pseudonocardiaceae:Pseudonocardia
0.000090	0.003680	Bacteria:Actinobacteria:Lawsonella
0.000063	0.004710	Eukaryota:Basidiomycota:Ustilaginaceae:Ustilago
0.000079	0.005646	Eukaryota:Ascomycota:Aspergillaceae:Aspergillus
0.000043	0.003578	Bacteria:Proteobacteria:Acetobacteraceae:Roseomonas
0.000068	0.003063	Bacteria:Proteobacteria:Leptothrix
0.000075	0.003691	Bacteria:Firmicutes:Lactobacillaceae:Lactobacillus
0.000066	0.003730	Bacteria:Actinobacteria:Bifidobacteriaceae:Bifidobacterium
0.000090	0.002751	Eukaryota:Apicomplexa:Babesiidae:Babesia
0.000072	0.002351	Bacteria:Proteobacteria:Caulobacteraceae:Caulobacter
0.000061	0.000650	Bacteria:Actinobacteria:Conexibacteraceae:Conexibacter
0.000067	0.000705	Bacteria:Proteobacteria:Comamonadaceae:Polaromonas
0.000038	0.000751	Bacteria:Firmicutes:Peptoniphilaceae:Peptoniphilus
0.000039	0.000663	Bacteria:Actinobacteria:Micrococcaceae:Arthrobacter
0.000043	0.000600	Eukaryota:Ascomycota:Glomerellaceae:Colletotrichum
0.000049	0.000737	Bacteria:Bacteroidetes:Cytophagaceae:Spirosoma
0.000045	0.000711	Bacteria:Proteobacteria:Oxalobacteraceae:Janthinobacterium
0.000027	0.001440	Bacteria:Bacteroidetes:Cytophagaceae:Dyadobacter
0.000031	0.001230	Eukaryota:Ascomycota:Sordariaceae:Neurospora
0.000029	0.001067	Bacteria:Proteobacteria:Yersiniaceae:Rahnella
0.000045	0.001677	Bacteria:Actinobacteria:Dermacoccaceae:Dermacoccus
0.000028	0.001975	Bacteria:Spirochaetes:Spirochaetaceae:Treponema
0.000030	0.002078	Bacteria:Actinobacteria:Microbacteriaceae:Frondihabitans
0.000090	0.001391	Bacteria:Actinobacteria:Geodermatophilaceae:Modestobacter
0.000120	0.001610	Bacteria:Proteobacteria:Neisseriaceae:Neisseria
0.000083	0.000692	Bacteria:Actinobacteria:Mycobacteriaceae:Mycobacteroides
0.000091	0.000675	Bacteria:Proteobacteria:Comamonadaceae:Limnohabitans
0.000100	0.000572	Bacteria:Actinobacteria:Actinomycetaceae:Schaalia
0.000114	0.000609	Bacteria:Fusobacteria:Fusobacteriaceae:Fusobacterium
0.000120	0.000649	Bacteria:Firmicutes:Streptococcaceae:Lactococcus
0.000183	0.000665	Bacteria:Firmicutes:Peptoniphilaceae:Finegoldia
0.000155	0.000694	Bacteria:Proteobacteria:Moraxellaceae:Psychrobacter
0.000222	0.000940	Bacteria:Proteobacteria:Enterobacteriaceae:Enterobacter
0.000228	0.000627	Bacteria:Bacteroidetes:Sphingobacteriaceae:Sphingobacterium
0.000264	0.000705	Bacteria:Deinococcus-Thermus:Deinococcaceae:Deinococcus
0.000000	0.000701	Viruses:Baculoviridae:Betabaculovirus
0.000202	0.001412	Viruses:Siphoviridae:Pahexavirus
0.000373	0.001470	Bacteria:Firmicutes:Veillonellaceae:Veillonella
0.000370	0.001478	Bacteria:Proteobacteria:Hydrogenophilaceae:Hydrogenophilus
0.004612	0.000099	Bacteria:Proteobacteria:Comamonadaceae:Alicycliphilus
0.002574	0.000204	Bacteria:Proteobacteria:Xanthomonadaceae:Pseudoxanthomonas
0.011300	0.000574	Bacteria:Proteobacteria:Burkholderiaceae:Paraburkholderia
0.014429	0.000134	Bacteria:Proteobacteria:Comamonadaceae:Diaphorobacter
0.000654	0.000046	Bacteria:Proteobacteria:Comamonadaceae:Melaminivora
0.000565	0.000157	Bacteria:Bacteroidetes:Flavobacteriaceae:Elizabethkingia
0.000535	0.000127	Bacteria:Proteobacteria:Rhizobiaceae:Sinorhizobium
0.001299	0.000094	Bacteria:Proteobacteria:Zoogloeaceae:Thauera
0.001357	0.000148	Eukaryota:Trypanosomatidae:Leishmania
0.001183	0.000126	Eukaryota:Ascomycota:Saccharomycetaceae:Naumovozyma
0.001249	0.000116	Bacteria:Proteobacteria:Burkholderiaceae:Pandoraea
0.005302	0.000888	Bacteria:Proteobacteria:Sphingomonadaceae:Novosphingobium
0.002859	0.001191	Bacteria:Proteobacteria:Erwiniaceae:Erwinia
0.000961	0.000657	Bacteria:Proteobacteria:Burkholderiaceae:Cupriavidus
0.000912	0.001064	Bacteria:Actinobacteria:Streptomycetaceae:Streptomyces
0.001391	0.000885	Bacteria:Proteobacteria:Phyllobacteriaceae:Mesorhizobium
0.000810	0.001708	Bacteria:Actinobacteria:Micrococcaceae:Rothia
0.001749	0.001590	Bacteria:Proteobacteria:Methylobacteriaceae:Methylorubrum
0.001372	0.001893	Eukaryota:Ascomycota:Nectriaceae:Fusarium
0.001416	0.001426	Bacteria:Actinobacteria:Intrasporangiaceae:Janibacter
0.001217	0.001466	Bacteria:Proteobacteria:Sphingomonadaceae:Sphingobium
0.000579	0.000340	Eukaryota:Ascomycota:Clavicipitaceae:Pochonia
0.000633	0.000594	Bacteria:Proteobacteria:Pasteurellaceae:Haemophilus
0.000795	0.000456	Bacteria:Proteobacteria:Alcaligenaceae:Bordetella
0.000676	0.000460	Bacteria:Proteobacteria:Bradyrhizobiaceae:Bosea
0.002503	0.000527	Bacteria:Proteobacteria:Rhodanobacteraceae:Luteibacter
0.001104	0.000453	Bacteria:Bacteroidetes:Flavobacteriaceae:Flavobacterium
0.001534	0.000541	Bacteria:Proteobacteria:Sphingomonadaceae:Sphingopyxis
0.000478	0.002652	Bacteria:Proteobacteria:Comamonadaceae:Ottowia
0.000383	0.003208	Bacteria:Proteobacteria:Rhizobiaceae:Neorhizobium
0.000447	0.004593	Bacteria:Bacteroidetes:Prevotellaceae:Prevotella
0.000689	0.004105	Bacteria:Proteobacteria:Comamonadaceae:Hydrogenophaga
0.000191	0.005306	Bacteria:Actinobacteria:Micrococcaceae:Kocuria
0.000160	0.006648	Bacteria:Actinobacteria:Nocardioidaceae:Marmoricola
0.000182	0.002626	Eukaryota:Apicomplexa:Sarcocystidae:Toxoplasma
0.000192	0.003541	Bacteria:Firmicutes:Bacillaceae:Anoxybacillus
0.000195	0.004155	Bacteria:Proteobacteria:Enterobacteriaceae:Klebsiella
0.000734	0.037757	Bacteria:Proteobacteria:Bradyrhizobiaceae:Bradyrhizobium
0.000437	0.062256	Bacteria:Bacteroidetes:Sphingobacteriaceae:Pedobacter
0.000510	0.061728	Bacteria:Actinobacteria:Nocardioidaceae:Nocardioides
0.000524	0.059435	Bacteria:Proteobacteria:Oxalobacteraceae:Massilia
0.000251	0.065166	Bacteria:Bacteroidetes:Hymenobacteraceae:Hymenobacter
0.000187	0.031172	Eukaryota:Ascomycota:Sclerotiniaceae:Botrytis
0.000113	0.031470	Bacteria:Actinobacteria:Nocardioidaceae:Aeromicrobium
0.016699	0.011013	Bacteria:Bacteroidetes:Flavobacteriaceae:Cloacibacterium
0.016150	0.009669	Bacteria:Proteobacteria:Xanthomonadaceae:Stenotrophomonas
0.004751	0.005834	Bacteria:Bacteroidetes:Flavobacteriaceae:Chryseobacterium
0.006792	0.007216	Bacteria:Proteobacteria:Moraxellaceae:Moraxella
0.001237	0.006426	Bacteria:Proteobacteria:Erwiniaceae:Pantoea
0.001465	0.003459	Bacteria:Actinobacteria:Actinomycetaceae:Actinomyces
0.001792	0.004495	Bacteria:Proteobacteria:Comamonadaceae:Variovorax
0.002112	0.002547	Bacteria:Proteobacteria:Rhizobiaceae:Rhizobium
0.000000	0.002029	Bacteria:Bacteroidetes:Hymenobacteraceae:Rufibacter
0.002498	0.002024	Bacteria:Actinobacteria:Gordoniaceae:Gordonia
0.003152	0.004475	Bacteria:Proteobacteria:Comamonadaceae:Comamonas
0.004224	0.003058	Bacteria:Proteobacteria:Comamonadaceae:Delftia
0.002356	0.025086	Eukaryota:Basidiomycota:Malasseziaceae:Malassezia
0.001578	0.015965	Bacteria:Actinobacteria:Micrococcaceae:Micrococcus
0.000573	0.010998	Bacteria:Actinobacteria:Microbacteriaceae:Plantibacter
0.000983	0.019121	Bacteria:Proteobacteria:Burkholderiaceae:Ralstonia
0.000694	0.023778	Bacteria:Proteobacteria:Rhodobacteraceae:Paracoccus
0.002840	0.110047	Bacteria:Actinobacteria:Microbacteriaceae:Microbacterium
0.001514	0.052336	Bacteria:Actinobacteria:Corynebacteriaceae:Corynebacterium
0.002495	0.052050	Bacteria:Proteobacteria:Caulobacteraceae:Brevundimonas
0.004140	0.053286	Bacteria:Actinobacteria:Nocardiaceae:Rhodococcus
0.003398	0.041241	Eukaryota:Albuginaceae:Albugo
0.003846	0.032116	Bacteria:Firmicutes:Staphylococcaceae:Staphylococcus
0.004718	0.016006	Bacteria:Firmicutes:Streptococcaceae:Streptococcus
0.008759	0.023461	Bacteria:Proteobacteria:Methylobacteriaceae:Methylobacterium
92.685587	90.633450	Eukaryota:Arthropoda:Phytoptidae:Fragariocoptes
0.814094	0.004811	Bacteria:Proteobacteria:Rhizobiaceae:Agrobacterium
0.174247	0.026721	Bacteria:Proteobacteria:Burkholderiaceae:Burkholderia
0.092704	0.003684	Bacteria:Proteobacteria:Comamonadaceae:Acidovorax
1.167029	0.254802	Bacteria:Proteobacteria:Anaplasmataceae:Wolbachia
4.492465	0.035061	Bacteria:Proteobacteria:Pseudomonadaceae:Pseudomonas
0.148333	4.821858	Eukaryota:Streptophyta:Rosaceae:Fragaria
0.140725	1.675884	Eukaryota:Chordata:Hominidae:Homo
0.027683	0.045337	Bacteria:Proteobacteria:Xanthomonadaceae:Xanthomonas
0.021667	0.022301	Bacteria:Proteobacteria:Moraxellaceae:Acinetobacter
0.052133	0.538561	Bacteria:Actinobacteria:Propionibacteriaceae:Cutibacterium
0.008142	0.188276	Bacteria:Proteobacteria:Sphingomonadaceae:Sphingomonas
