# Supplementary material for: Symbiotic bacteria of the gall-inducing mite Fragariocoptes setiger (Eriophyoidea) and phylogenomic resolution of the eriophyoid position among Acari
Source: Sci Rep. 2022 Mar 9;12:3811. doi: 10.1038/s41598-022-07535-3 (PMC8907322; doi:10.1038/s41598-022-07535-3)
Supplement: Supplementary file 4 — Supplementary Information 2. [file 41598_2022_7535_MOESM4_ESM.docx]

Supplementary Data S1. Unix shell script used to generate a phylogenomic matrix for 27 chelicerate genomes.

#!/bin/bash

#note, this script generates a PBS file to be run on a HPC cluster

wd=$(pwd)

pref=Mites_occ80.busco4_onArachnida_db_concatBUSCO_filter_raxml.partitioned

fila=fila64 #256 64

proc=2

pscript=/home/pavel/bin/Phylogenetic_scripts

occupancy=0.8

#make sure that busco_info.dat is populated (col1=species (eg Aus_bus), col2=busco dirs, MUST be in the script directory), see genomic_BUSCOs2uscofa.py manual

busco_pref='at6854' #make sure that BUSCOs names match in the ls commands below (eg EOG090X*)

#filtering criteria

mocup=0.8;mlen=200 #minumum taxon occupancy (after trimal) and min alignment length (>=, see awk below to modify)

occuppancy_file=fastas1_trimal.list

#these files will be deleted

rm -f *.fa *.fa.* concat* RAxML_info.m *.list $busco_pref

FILE="0-"$pref".pbs"

/bin/cat <<EOM >$FILE

#!/bin/sh

#Author pavelito

#PBS -N $FILE

#PBS -e $wd/pbs.err

#PBS -o $wd/pbs.log

#PBS -M pklimov@umich.edu

#PBS -q ${fila}

#PBS -l select=1:ncpus=$proc

cd $wd

###MAIN START

while read col1 col2

do

echo \$col1 >> taxon_names.list;

echo \$col2 >> busco_dirs.list

done < busco_info.dat #col1=taxon names, 2=busco folders (must be in the script dir)

. /home/pavel/bin/miniconda2/etc/profile.d/conda.sh

conda activate

source activate /home/pavel/bin/miniconda2/envs/biopython #conda activate biopython #headnode syntax

echo "Selecting buscos based on gene occupancy"; python $pscript/genomic_BUSCOs2uscofa.py -b busco_dirs.list -c prot -o $occupancy -t taxon_names.list

echo "running mafft..."; for i in \$(ls *$busco_pref.fa); do /home/pavel/bin/mafft-7.429-with-extensions/core/mafft --thread $proc --inputorder --bl 62 \$i > \$i.mafft ; done

echo "running trimal..."; for i in \$(ls *$busco_pref*mafft); do /home/pavel/bin/trimAl1.4/trimal -in \$i -out \$i.trimal -automated1 -resoverlap 0.75 -seqoverlap 80; done #added: -resoverlap 0.75 -seqoverlap 80

echo "standartizing fasta titles (species names only, eg Aus_bus|gene_id -> Aus_bus) across trimmed alignments..."; for i in \$(ls *.mafft.trimal); do sed -e 's/|.*//g' \$i > \$i.same_names.fa ; done

######filtering

rm -f $occuppancy_file $occuppancy_file.len $occuppancy_file.len.filtered

ls *.same_names.fa > $occuppancy_file

ntaxa=\`cat busco_info.dat | sed '/^\s*$/d' | wc -l\`

while read col1

do

nseq=\`grep "^>" \$col1 -c\`

aln_len=\`awk "/^>/ {n++} n>1 {exit} {print}" \$col1 | awk '/^>/ {if (seqlen){print seqlen};seqlen=0;next; } { seqlen = seqlen +length(\$0)}END{print seqlen}'\`

echo "\$col1 \$nseq \$((\$ntaxa - \$nseq)) \$(echo "\$nseq/\$ntaxa" | bc -l ) \$aln_len" >> $occuppancy_file.len

done < fastas1_trimal.list #col1=fasta file name (in the same dir), 2=taxa in fasta 3=taxa not in fasta, 4=occupancy 5=alignment length

#cat $occuppancy_file.len

awk 'BEGIN {FS=" "} ; \$4 >= oc && \$5 >= ml { print \$1}' oc="${mocup}" ml="${mlen}" $occuppancy_file.len > $occuppancy_file.len.filtered;

#cat $occuppancy_file.len.filtered

echo "lines before filtering"

wc -l $occuppancy_file

echo " "

echo "filtered lines:"

wc -l $occuppancy_file.len.filtered

#####end filtering

echo "concatenating matrices ..."

python $pscript/create_concat_matrix.py -a $occuppancy_file.len.filtered -c prot -t taxon_names.list -p concat

#source deactivate

conda deactivate

###MAIN END

mv ~/$FILE.o* ~/$FILE.e* $wd

mkdir run_$pref

mv concat* $busco_pref* fastas1_* RAxML_* busco_dirs.list taxon_names.list $FILE.* run_$pref

scp busco_dirs.list run_$pref

EOM

a=`qsub -k oe $FILE`

jobId=$(echo $a | awk -F"." '{print $1}')

/bin/cat <<EOM >$FILE".info."$jobId

Results will be saved here: $outDir

Cancel job: qdel $a

Job status: qstat $a

Monitor RUNNING job progress & errors: cat ~/*$jobId

Check FINISHED job progress & errors: cat $outDir/$FILE.o* $outDir/$FILE.e*

EOM

cat $FILE".info."$jobId
