# Supplementary material for: Symbiotic bacteria of the gall-inducing mite Fragariocoptes setiger (Eriophyoidea) and phylogenomic resolution of the eriophyoid position among Acari
Source: Sci Rep. 2022 Mar 9;12:3811. doi: 10.1038/s41598-022-07535-3 (PMC8907322; doi:10.1038/s41598-022-07535-3)
Supplement: Supplementary file 5 — Supplementary Information 3. [file 41598_2022_7535_MOESM5_ESM.docx]

#Supplementary Data S2. Concatenated phylogenomic matrix for 27 chelicerate genomes. Data are presented as amino acid alignments in fasta format; IQ-TREE-compatible partitions are given.

>Limulus_polyphemus

PGSKVNALSSEVMQGVVLMSSKPGCFIAGADIGMLEACQTEVQKLKPIVAAIMGSCLGGGLEVALACQYR-IAVKDKKTTLGLPEVMLGLLPGAGGTQRLPKLISIPSALDMMLTGRSIRPDKAKKMGLVDQLVMPLGPGLYLEEVAASKLARGEMKPRKRPLIERVIREQ-LFKKVQAQVIKQTQGLYPAPLKILEVVKTGLESMGFGELATPHSRALIGLYHGQTLCKKNRFGAPAKKVGVVGAGLMGAGIAQVSVDKGYEVILKDGLARGEHQIKKKKIEKERFLSNLEPVLNYSDIVIEAVFEDIEIKHIVLKEVEQDHCIFASNTSALPISKIAEASKRPEKVIGMHYFSPVDKMQLLEVITTDKTSSDTAAMAVDVGLKQGKVVITVKDGPGFYTTRILAPMLAEAMRILQEGTNVKELDSLKKFGFPVGAATLVDEVGIDVAAHIAEGVFGERKDLVAGCLGRKSGKGCFVYRDINPILKKYTTEELQMRLICRFVNESVLCLQEGILANPLEGDIGAVFGLGFPPFLGGPFRYIDSYGADKIVGMEKFTEFKPCELLIDHA-RDPTKNFHMQQPIILLKEGTENIQGKSQLISNINACQAVAEAVRTTLGPRGMDKLIVDNNGKATISNDGATIMKELDIVHPAAKTLVDIAKSQDAEVGDGTTTVVLLAAEFLKQCKSYIEEGVHPQVIIKSYRKAVQLAVDKINEISVKVKKEKHRAILEKCAMTTLSSKLVANQKTFFSKMVVDAVMQLDELLPLNMIGVKKVQGGALEESKLISGVAFKKTFSYAGFEMQPKKYVNPKIALLNVELELKAEKENAEVRVESVQEYQNIVDAEWNILYDKLKKIHDSGAKVVLSKLPIGDVATQYFADRDMFCAGRVQEEDLKRTMKACEFCYLTMVSPVRASSGCICRDIEYKVLFPYRYNLFTGCSNTKTVTIILRGGAEQFIEETERSLHDAIMIVRRAMKNDAVVAGGGAIEMELSKYLRDYSRTIPGKEQLLIGAMAKAFEIIPRQLCDNAGFDATNILNKLRQKHATGELWCGVDINNEDIANNFDACVWEPAIVKINALVAATEAACLILSVDETVKNPKSETDPSKGRPFMNEVNPAAANKQHQLAVTRDFISQPRLTYKTVSGVNGPLVILDDVKFPKFAEIVHLTLADGNVRTGQVLEVSGSKAVVQVFEGTSGIDAKHTVCEFTGDILRTPVSEDMLGRVFNGSGKPIDKGPHVLAEDFLDIQGQPINPWSRIYPEEMIQTGISAIDVMNSIARGQKIPIFSAAGLPHNEIAAQICRQGGLVKVPGKSLDDDNFAIVFAAMGVNMETARFFKQDFEENGSMENVCLFLNLANDPTIERIITPRLALTTAEFLAYQCEKHVLVILTDMSSYAEALREVSAAREEVPGRRGFPGYMYTDLATIYERAGRVEGRNGSITQIPILTMPNDDITHPIPDLTGYITEGQIYVDRQLHNRQVYPPINVLPSLSRLMKSAIGEGMTRRDHADVSNQLYACYAIGKDVQAMKAVVGEEALSSEDLLYLEFLGKFEKNFISQGNYENRSVFDSLDIGWQLLRIFPKEMLKRIPQKTLTEFYPRDSRHRS---------KGLFGEPDGFYLLKEEAMLETDKLVREATSRTRKMVQIFDELSDTLCKVADMAEFVRIGHPQNRFAKAAENASMAISGLVEKLNTNRELYNALKKVTQEG-DIVLTNVDEHVAELFLFDFEQSGIHVDDLMRERAVALNEYILHIGSHFVNGTNQPKSVLKSQLPENIILVTGLFADSENDLVREAAYKIYLFPDSHQAKLLDELLISRNELANLCGFPTYAHRAVRGSLAGDPQTVEFLDILSDGLRERAQNDYNDMLQLKPWDIPYYTPYFSLGICMEGLNNLFQSLFGTTFDVEGELWHKDVIKLSVHEEGSILGYIYCDFFERPGKPHQDCHFTIQGGRLENGTYQLPVVVLMLNLPPSLLTPGMVDNLFHEMGHAMHSMLARTPYQHVTGTRCATDLAEVPSILMEHFSSDPRVVLQFAKHYQTGAPIPHSLIEKWNASKQLYSASETQLQVFYAALDQVFHGEHPLSTTEILADVQNQYYGIPHVPNTAWQLRFGHLVGYGAKYYAYLMSRAVASCIWQVYFKDDPFSSSAGEYRRGVLAHGGSRPPRELIQDFLLAESLLNDIMPSDSKKKREAKKKEAAKQRAQKKTANGLASENELVKKLEHDMDLNAKARAVTGVQGLNPHSRDIKIENFSITFHGAEILADTKLELNCGQRYGLIGLNGSGKSSLLASIGRREIPIQDHIDIYHLTREIEPSNNTALQAVLDVDQERIRLEKLAEELAHYPDDDSQEQLMDIYERLDDIGADKAVSKAAYILHGLGFNKRMQEQKCKDFSGGWRMRIALAKALYIRPHLLLLDEPTNHLDLDACVWLEEELKSYKRILVIISHSQDFLNGVCTNIIHLHKKRLNYYGGNYDAFVRTRLELLENQMKRYNWEQSQISHMKDYIARFGHGSAKLARQAQSKEKTLSKMVSAGLTEKVMLDKNVQFYFPSCGTIPPPVIMVQHVSFRYTENGPWIYKDLEFGIDLDTRVALVGPNGAGKSTLLKLLCGELIPSDGLIRKHSHLRIARYHQHLHEQLDLDVSALEYMMRCFPDVKEKEDMRKIIGRYGLTGRQQTCPIRQLSDGQRCRVVFAWLAWQTPHLLLLDEPTNHLDMETIDALADAINDFDGGMVLVSHDFRLISQVAEEIWVCANQTISKWSGDILSYKEHLRKRIISEIQKMVLADLGRKITSALRSLSNATIINKEVLDSMLKEICAALLEADVNIKLVKQLRENVRSVIDFEDMAGGLNKRRMIQSAVFKELVKLVDPGVKAYQPTKAKSNIIMFVGLQGSGKTTTCTKLAYYYQKKGWKTSLVCADTFRAGAFDQLKQNATKARIPFYGSYTEVDPVVIAQDGVEKFKNEGFEIIIVDTSGRHKQEDSLFEEMLQVSIAVSPDNIIFVMDASIGQACEAQARAFKEKVDVGAVIVTKLDGHAKGGGALSAVAATHSPIIFIGTGEHIDDFEPFKVKPFVSKLLGMGDIEGLIDKVNELKLDDNEELIEKLKQGEFTLRDMYEQFQNIMKMGPFSQIMGMIPGFSSDFMTKGNEQESMSRLKKLMTIMDSMNDQELDHREGAKLFSRQSGRVTRVARGAGVTSREVQELLSQYTKFAAMVKKMGGIKGLFKGGDMGKNVNPAQMAKLNQHMAKMIDPKVLQQMGGMSGLQNMMRQLQGALLRDDPAFKKLQDYYDRNGSKINMLQLFNEDPDRFKKFSLNVKTAVGEILFDFSKNLINDEVLQLLLELAKSRKVEAARDAMFSGEIINFTENRAVLHIALRNMAGTSIIIDGQDVMPGVMRVLNHMRDFTDFVRNGEWKGYTGKPITDVVNIGIGGSDLGPLMVTEALKAYCCGPNVHFVSNIDGTHLAETLKHLNPETSLFIIASKTFTTQETITNAQSAKTWFLQHAKDDTAVAKHFVALSTNEPKVKEFGIDEKNMFEFWDWVGGRYSLWSAIGLSIALHIGMDNFEQLLTGAHFMDQHFKNMPLERNVPVLLALLGVWYGNFYGAESHALLPYDQYLHRFAAYFQQGDMESNGKYVTRSGAIVNYTTGPIVWGEPGTNGQHAFYQLIHQGTRLIPCDFIAPVKTLNPISDGLHHEILLANFLAQTEALMRGKTKEEAETELTKAGLDGEQLQKILPHKVFEGNKPTNSIMIPKLTPFNLGVLIAMYEHKIFTQGIIWDINSFDQWGIHAIFLFIKFGQLGLAGPDPISGNFSVTGYCLNFIIYSV-------------------------------------MDKMIQAGNGDVTITNDGATILKQMQVLHPAAKMLVELSKAQDVEAGDGTTTVVIIAGSLLDAASKLLAKGIHPTTISESFQKAAIKSVEVLSDMAVPVDLSDRESLLKSASTSLNSKVVSQHSSQLAPLAVNAVMKVIDPTNVDLRDIKIIKKLGGTVDDTELIEGLVFAQKTSGAGGPNKIEKAKIGLIQFCISPPKTDMDNQVIVSDYTQMDRVLREERAYILNIVKQIKKAGCNVLLIQKSILRDALSDLALHFLAKMKILVVKDIEREDIEFVCKSLVCKPIASLDHFVPEALGSADLVEEIHGSKFVKITGVANP---KTVSIFMRGSNKLVLEEAERSLHDALCVIRCLVKKRALIAGGGAPEIEISLRLSEHARSLTGMEAYCYRAFAEALEVVPYTLAENAGLSPISTVTELRNRHAQGEKTAGINVRKGAVTNILEENVIQPLLVSTSAVTLAAETVRSILKIDDIWL-----KKSVIYFWDPDVGNFHYGPGHPMKPHRLSVTHSLVLHYGLYKKVQVYRPYRASAHDMCRFHSDEYIDFLQRVTPQNIQSFTKSLSHFNVGDDCPVFDGLYDFCSMYTGASLEGAVKLNNMCCDIAINWSGGLHHAKKFEASGFCYVNDIVIAILELLKYHPRVLYVDIDIHHGDGVQEAFYLTDRVMTVSFHKYGNYFFPGTGDMYEIGAESGRYYSVNVPLKEGIDDQSYFQVFKPVIQHVVEFYQPTCIVLQCGSDSLAGDRLGCFNLSIRGHGECVKFVKELGLPLFVLGGGGYTVRNVARCWTYETSLLVEETISNEIPYNEYFEYFAPDFLLHPE-------ENANSRQYLEAIIKATTENLKCLIHAPSVQMQDVPPDTLNFDNNESEADAKAEPANEFFDKDQDKDNE??????????????????????????????????????????????????????????????????????????????????????????????????????????????????????????????????????????????????????????????????????????????????????????????????????????????????????????????????????????????????????????????????????????????????????????????????????????????????????????????????????????????????????????????????????????????????????????????????????????????????????????????????????????????????????????-MALSLNPVRILKGDAEEEKAEHARMSSFIGAIAIGDLVKSTLGPKGMDKILLCEGRQEGKVECTNDGATILRSVGVDNPAAKILIDISKVQDDEVGDGTTSVAVFASELLKEAEQLINMKLHPQTIIAGWRKAAIEARDALTSFARDNSGNQEKFYEDLINISRTTLSSKILSQSKDFFAKLAVDAVVRLKGSGNLQAIQVLKKLGGSLTDSFLDEGFLLDKKVGVNQPKRIEKARILIANTPMDTDKIKVFGSRVRVESVAKVAELELAEKEKMKDKVEMILKHNINVFINRQLIYNYPEQLFADVGVMAIEHADFDGIERLALVTGGEIVSTFGCPEKVKLGFCNLIEEVMIGEDKLLKFSGVPLGEACTIVLRGSTQQILDEAERSLHDALCVLSQTVKETRIVFGGGSAEMLMATAVGRLAERTPGKEAMAMESFAKALRQMATIIADNAGYDSAQLISELRAAHAEGKNTYGIDIRHHLLDSLQKLVILNHFRTSKTSIINIYQILDEIYKIENVMIVGLRRYN-DMNNMAAEIKPGGKPQLLNKLEGHQDTVNMAVIIPGEDGVISISDDKTVRIWLKRDTGQYWPSICHYMPSAATCMDYNSETKRLFVGMENGSISEFQVAEDYNRMAHQRNYLAHQAHVTGVVFSLITEWVLSVGRDKYFQWHCSESGRRFGGFHCNAWCTALQFDMQSKHAFIGDYSGQITMLKIESSGYKPVTTLKGHSGSICCLSWDAERQLLFSGSFDQSIVVWDIGGKQGTAYELQGHHNKVTALCYAGMSKLISGSEDSILVFWHMEAFPLDTPEWAESDCCQRCTRPFFWNVKAMIDQKTIGIRQHHCRRCGKAVCDACSTNQSSIPTMGYEFDVRVCDECHSVITDDDRIPMATFHDARHCIVDMDLDETRFHLLTVGSDRIMKLWDVSSLLM-------IVIVTCLIQVTKVAPKKKDVINKWTRNLKEGVNTEEDYSSGQYGKLPMNQSQKKIVRHLVEVKVLDLNFADQKVWVRGRLHTSRAKGKQCFFVLRQQHFTVQCLVSVSETISKAMVKFVALITKESILDVEGHVRTVFQKIESCTQKDVELHVEQLFVVSASEPRLPLQIEDASRPENE-----------------------------EAIYCLQAGVCELFREALKKMGFIEIHTPKIISAASEGGANVFEVSYFKGSAYLAQSPQLYKQMAIAADFDKVFTIGAVFRAEDSNTHRHLCEFVGLDLEMAFHYHYHEVITVIGMMFVEIFKGLRDRFSAEIAAVHRQYPAEPFKFLEPSLRLDYPEAVAMLRETGVDMGDEEDLSTPNEKLLGRLVKAKYDTDFFILDKFPLAVRPFYTMPDPHNSKYSNSYDMYMRGEEITSGAQRIHDPEFLAERAKNHGINIEHIKAYIDSFRYGAPPHAGCGIGLERVTMLYLGLDNIRKASMFPRDPRRLTPLKEKALLIRHVDRIVCKRIRLCYELSCKRS----REKFSIGNXXXXNARIEDIKDNSKEIESIWG---------SPVKMIKRVEIGEHLGASQILKGCDKSCIFLNIQCKHIPNRKKRVTTSEKPGDLEDYRRSRKLVKQESKRMYEKKLAENVKIIRMIKEGXIGFITFLYYFSV---------------------FNCSCVANMLRLVQEGLYSIQIKKYIGERFRWYPDERVTDFLLKQCICVHLDSNVHKFNLLVYMTRKLFALAKGECAIENPDNPMNQEVLLGGHLYLMVLKEKLESWLNTIKMILNRKMLQYIDISRPMEYLLATGNLVSRSGIGLMQFSGLTIVADKLNFFRYLSHFHSIHRGSFFAEMRTTAVRKLLPEAWGFLCPVHTPDGAPCGLLNHISAMCEIVVLLDGKFVGWVLRYMKVMQKVPSTLEIAFVEATTKASQYPGIYLFSTPARMLRPVVNTTNTIELIGTFEQVYLNICVVPEEATTHQELRETSMLSVLANMIPFSDFNQSPRNMYQCQMGKQTMGTPCYTYQYRSDNKMYCITPQSPLVRPVMYDFYHIDDYPLGTNAIVAVISYTGYDMEDAMILNKASLERGFKNGVYKSEFINLRIDMDGLPFIGTYGTPMCSYIETRVEKYKSTESAYVLYVKLCANLQKICITLWIRNPIIGDKFASRHGQKGVCSQQWPAENMPFTESGMTPDIVFNPHGFPSRMTIGMMIESMAGKAAAVHGLAFDATPFKFNEKKPAAEYFGELLQSAGYNYYGTERMYSGVDGRELEADIFFGVVYYQRLRHMVADKFQVRTTGPIDILTHQPVKGRKRAGGIRFGEMERDSLLAHGTSFLLQDRLFNCSDKSLCTRCGSLLSITVPYVFRYLVVE-LAAMNIKIWNDSWNLCYSRRRLAPQLAFFLTKHFLTNAIVYSFIPKIYKKQFLTTILNLDIL-FIHMHAKFHPLLVSGTRNSLVTIAHLHDSKAIRPISNSESTCRYIS-------VIYYDISHHLEPLFVTCGEKVDLWEERSEPLRSFW-GVDSLHVKFNPVESTASDRSIILYDIREAHPLRKVVLELRSNTICWNPMEAFIFTAANEDHNLYTFDMRKLKVPLQIHLDHVSAVIDVDYSPTGKEVVAGSYDKTVRIFRSRIVKKEGRSHVTTTDVTVTDDRTIPDGADEPHTVYWVKKTRYTL---EYCDLMLDIYSYIPFKKEISTFPHNVKIFRLKVFMMVSAVSRV--MDVIALHQIEDGISYYRLYVEKFSKDHLSHQMIGNVYQLLFKLKLTLNDLIGIVTDDYSFCYARLQRGNILLKQGRLEEAHIDYEWVLRLDPLNGDADLEPVKRDAIDVLTRLWDGKLREMRAGCYEALNDLVNAIGDLRSATKMRSDNTEGYLKLSQLHYKLGEVDESLTVIRECLKLDPDHKQCYKKVKKLARAMQDSCVEKIVQHIKGRCHCLNKGGEAISICSEALKLNAALCDRADAYLLNEDYDEDFQHAVNIDERAKEGLEKTQKLVKQAKKRDYYKILAGKREIMKAYRKLAMKWHPDNF-QGDE--KKTAEKKFIDIAAAKEVLTDPEKRQKFDNGEDPLDPDSQFTFKFHFMLVLFESPAGYAIFKLLDEKKLQETENLYKDFESSETASKVVKLKHFQKFQDMTEALSAATAAVEGKMSKGLKKLLKKLVAKEAQESLAVADAKLGNVIKEKLNVSCVYNSAIQELIRCIRMQIDNLIVGLPQKEITAMALGLAHSLSRYKLKFSPDKIDTMIIQAVSLLDDLDKELNNYIMRSKEWYGWHFPELSKIITDNNQYVHTVLAIGLRTNAAECDMSDFLPEELEHKVKEIAEVSMGTEVSEEDIMNIKHLCNEVLEMQNYRAQLYEYLKNRMMAIAPNLTVLVGELVGARLIAHAGSLLNLAKHPASTVQILGAEKAFFRAIKTKHDTPKYGLIYHAQLIGQCNPKLKGKMSRMLAAKSSLATRVDALGEEASQELGIEHRAKLETRLKQLEEGSLRKISGTGKARAKWEKYESKSEIYQYKPGLDSTIPTGQKRKIGEIEEDSFQPKKIELK--EETGEPE--VQEADTSEKKKKKKKKKEKSEVGPV-SQETEEVDQPEKKKKKVKQEEEHEVVKQEEVGECSTVVEKKKKKKKKKESECYLWPKDKPGIKGRVVVALGLLVGAKLLSVEVPFIFKYAVDFLNSHSGLMLGYGAARAGAALFNELRNAVFAKVAHNSIRRVARRVFLHLHDLDLSFHLSRHTGALSKVIDRGTRGINFVLSALVFNVVPTIFEVTLVSSILYYKFGKFALVTLGCIGTFAVTSWRTRFRLEMNKAETQAGAKAVDSLINYETVKYFNNEEYELTKYERASLKTTTSLALLNFGQNAIFSGAIMILASQMTIGDLVMVNGLLFQLSMPLNFLGSVYREVRQSLIDMQTMFELMAIETKLLQVTPTVTFEDVCFQTILNNLSFTVPTGKKVALVGGSGSGKSTIIRLLYRFFNPDKGRIMIAGKDIKDVSLRKAIAIVPQDTVLFHNTILHNLHYGDLTDEVYQAARLAELHDTIMRWYETQVGERGLMLSGGEKQRVAIARAALKDSPILVFDEATSNLDAITEYKIMNALRASKGRTSIFIAHRLSTVVDADDIIILEGGQVVERGHHSLLSSLYTHLWFKQHEFAVR--FQHTARPFAQTLLNVPETRVTTLTNGLRVATEDSGIPTCTVGLWIDTGSRYETDKNNGVAHFLEHMSFKGTSKRSQTDLELEIENMGAHLNAYTSREQTVYYAKSLSKDLPRAVEILADIIQNSKFGEQEIERERGVILREMQEVETNLQEVVFDHLHAVAYQGTPLGRTILGPTENIKSISRQDLVEYITNHYKGPRIVFAGAGGVGHEELVKLVDQHFGSLKTTYEGKTPCRFSGSEIRVRDDSMPFAHVAVAVEGCGWTNPDNIPLMVANTLIGSWDRSHGGGSNVASKLGQAATLGNLCHSFQSFNTCYKDTGLWGIYFVSEGLSLEDMLFNVQSEWMRLCTSVTEGEVSRAKNLLKTNMLLQLDGSTPICEDIGRQMLCYGRRIPLPELEARIEGIDIEIKNSI-------KCYNI-----VESKFNFITTDKQLLKHSVHTLVFRSLKRTHDMFLSDQANPPPPDETSEKIKLMVKAADEYKPVMHLIRNNERRSVSQALVLAGTQAIS-RKAPTMPKPQWHPPWKLYRVISGHLGWVRCIAVEPGNEWFCTGSNDRIIKIWDLASGKLKLSLTGHISGVRGLAVSPRQPYLFSCGEDKQVKCWDLEYNKVIRHYHGHLSGVYALALHPTIDVLITGGRDSVARVWDMRTKANIHTLAGHTNTVASVQSQATEPQVMTGSHDCTIRLWDLVAGKSKVTLTHHKKSVRALLIHPKLNMFASGAPDNIKQWKCPDGKFIQNLQGHNAIVNCLAMNVDNVLVSGGDNGTLFFWDWRTGYNFQRLQTPVQPGSIDSEAGIFAAIFDQSGTRLITAEADKTIKIFKEDDMATEDSHPINWKPEIAVLNKNSYVCGVSSSVRLDIIQDGLHLMYSIVYQGAFPVCICQFSKLRTVLQXSTSYVNDKRVEETHTKKEKLWKKSKILKFDEAVLLQHILQDSGARKLVVDFFNQD--LVYKFIKNSMYHTTLYSNKTSQNHRIEIELSKKRPAYIKAKERTAHMQKKLESAKKSLKAAKKVHEAHEGIKELENELKEVEEEFEEQDLTLEESQVQEYNSLKEEAGKLSSRYLQELDSVNREQKSDQDRHDNELRKKAEMEAKVKELEENVRRVEKLNELSDLMKQEEGFDAKKRINDELESIIELGDAKVDKHEDSRRKKKAEIVDHFKRLFPGVYDRLVNMCQPIHKRYNVAITKVLGRNMEAIVVDTERTARSCIKYLKEQMLEAETFLPLDYIDAKPLKERLRNIQSPRNVKLLYDVLQYDPPAIKRAVLYATNNALVSETAEDASKVAYELGDGKRYDAVALDGTYYQKNGFISGGSTDLAKRARRWDEKALHNLKYRKEKLTEELKEMVKKTRKESDLNTIQSQIRGLETRLKYSIIQSVEWRMKEREVQIAEVKEAMNTVEDRVFADFCHTIGVENIRQYEERELRATQERDRRLEFENQKNRIINRLEYER--SKDTNEVTDDEKELEKLKEAESKQMQLIDEEMQNLDRLKTSKITKKTEVDGMEDAMTEVRKRLTAMQKEITSVQKVVTGLETRLEQKKADRHSHLQACKLEDIIIPMKRGSMQRIYEKEKIQIDYIDLHLQKEISLQRIQAPNMRAMEKLDGVRERLRLTDSEFESARKKAKQVFEKVKRERSCFDHVSNKIDDIYKALTNNQSAQAFLGPENPEEPYLEGINYNCVAPGKRFQPMSNLSGGEKTVAALALLFAIHSYQPAPFFVLDEIDAALDNTNIGKVARFIREQTETSFQCVVISLKEEFYGHADALVGIVPDPGECTISRVLTLDLTVPEGVLVYLRKAIGEGNVYEIQDLYENSYVKLTERYFLILYKE--LYYHHLYADQRFESYYNYCDLFNYILSAGPVPLELPNQWLWEIIDEFIYQF--QSFSHYIWNVHSVLNVLHSLVDKSNINRQLEVYTSGGDPDSVAGEFGRHPLYKMLGYFSLVGLLRLHSLLGDYYQAIKVLENIELNKKSLSRVPACQITTYYYVGFAYMMMRRYADAIRTFADILLYVQRTRKTAQAEEISKQTDRMYHLLAICLVLHPQLIDESVLSQLKEKHMLRLQKGDLQEFENSPKFLSPVQLKVFMDEVQQQLTIRSYLKLYTTMPISKLAAFLLLCFKRKLLNV-FFTEVQSRSFLNVFFKPDMIHIADTKVARRYGDFFIRQIHRFDELYR------------------------------MGPPDPILGVTEAFKRDTNPKKINLGVGAYRDDQGKPFVLPSVRKAEELMMARHMDKEYAAIHGLQEFCQASASLAFSENSSVIKEGLNATIQGISGTGSLTMGAFFLRDFFKGNKEVYMPAPTWGNHIPLFKRAGFAVKQYKYYDPKTCGLDFKGLLEDISKIPENSVILLHACAHNPTGVDPKPEQWKEIQKPCKGKKLFPFFDMAYQGFATGDIDRDAMAVRMFVDNGHRIGLAQSFAKNMGLYGERVGAFTMVCNSKDEAQRVLSQLKIIIRPTYSNPPIHGARIAQTVLGEPELREQWLKDVKGMADRIISMRTRLRDGLKKEGSTRNWQHITDQIGMFCFTGMNPKEVEKLTAEFSVYLTKDGRISVAGISSNNVDYLAHAMHQATKILNSRTKHLLHCFLCFAVIITFEIFTFEFDNIDPIQKYGYIGSSILYLLRILTLLALPQCVFNFLGLVVYNAFPDKVQLKGSPLLAPFICVRTVTRGDYPELVKTNVTRNMNICFDIGMENFMIEVVSDKPVNLPKHPRIRELVVPSTYRSKSGALYKARALQYCLEDDVNILSDTDWIVHLDEETLLTENSLRGIMNFVFDGTHKFGQGLITYANENVVNWVTTLADCFRVADDMGKLRFQLQTYHRPLFGWKGSYVVTQAGAERKVSFDNGLDGSVAEDCFFAMMAYKEGYTFDFIQGEMWEKSPFSFWDFLQQRKRWMQGIFLVVHSPAIPFRNKIFLSLSLYSWITVPLSTSNLVLAGLYPIPCWQVLNVICAFVGAMNIYMYIFGVIKSFSLYRLGFFKFVFFVVGALCTIPFNIIIENIAVIWGFFGNKYRFYVVNKEIVMSILTTDCHKAARLQCPTCIKLGINDSYFCSQECFKGNWEIHKQVHKKARDQ---PWPGFQFTGKLRPFPKTPKREVPDLIQRPDYADHKKGWPLSEQAVKGSSHIKVLDDDEIEGMKVVCKLAREVLDVAADAAGVGVTTDEIDRLVHEASIERECYPSPLNYYGFPKSCCTSVNEVICHGIPDARPLEDGDLLNVDVTVYHRGFHGDLNETFLIGNVDEEGRRLVQVTHESLMKAIEIVKPGVKYREIGNVIQKHVQAHGFSVVRSFCGHGIHRLFHTAPSVPHYSKNKAVGIMKAGHCFTIEPMISEGTWRDLLWPDDWTAVTQDGKRSAQFEQTLLVTDSGCEILTQRREKGGQPHFMDKKNMLKVLKERSQKRRLLLAQQLGAGSVDNLSIVLGNQEDKYSRSSSKSNL---KSYSSSSKTGRLSTSYLLYNIYQEHFVHLIFYSSTFANFIYFEKAFEQSAYIQYLHRVKDHLIKGLREIWXYLKCDMETFELRELNCKFDVILVEPPLEEYQRSQGVSNSKFWTWDEIIKLEIEEVAAPRSFIFLWCGSSDGLDLGRQCLRRWGFRRCEDICWIKTNIKNSHSKNLEPRAIFQRTKEHCLMGIKGTVRRSTDGDFIHANVDIDLIISEETEYGSLDKPEEIFHIIEHFCLGRRRLHIFGRDTTIRPGWLTIGPELTNSNFNSETYNAYFNGPNEFLTGCTDRIEALRPKSPPPKGVGGTGSRG----???????????????????????????????????????????????????????????????????????????????????????????????????????????????????????????????????????????????????????????????????????????????????????????????????????????????????????????????????????????????????????????????????????????????????????????????????????????????????????????????????????????????????????????????????????????????????????????????????????????????????????????????MRTFGEKPTAFQLEEGGDYYYIGSEVGNYLRMFRGSLYKKYPSLWRRLVTVDERKKIASLGLGPHSLATNITLLRAVEVDEIFEGNDDRYKAVSISTEPPLPRETKSKRTTWMPALPNSSHHLDAVPCSTPINRNRSGHKKVRTFPHVYDDLDPSVIHENSSQPEVLVPIRLDMEIDGHKLRDTFTWNKNDSLITPEQFAEVLCDDLDLPPLSFVPAIAQSIRQQIDAFPTDNLLDEQTDQRVILKLNIHVGNISLVDQFEWDMSEKENSPEQFALKLCSELGLGGEFVTAIAYSIRGQLSWHQRTYAFSEAPLPTVDVPFRSQTEADQWCPFLETLTDAEMEKKIRDQDRNTRRMRRLANTAWYGANASQLEKEIGFPSNEHYFGLVNFGNTCYCNSVLQALYFCKPFREKVLDYKAKNKRTKETLLTCLADLFYNIATQKKKTGTIAPKKFIARLRKENDLFDNYMQQDAHEFLNYLLNTIADILQA---WVHDIFQGTLTNETRCLNCETVSSKDEDFLDLSVDVDQNTSITHCLRGFSNTETLCSEHKYYCENCCSKQEAQKRMRVKKLPMILALHLKRFKYMEQQSRHTKLSYRVVFPLELRLFNTSDDAYNPNRMYDLVAVVIHCGSGPNRGHYISIVKSHGLWLLFDDDIVDKIDASAIEDFYGLTSDTQKTSESGYILFYQSREMPDIDKWIDIAKECKYLPENDLKKLCDIVCDLLLEESNIQPVSTPVTVCGDIHGQFYDLEELFRTGGQVPDTNYIFLGDFVDRGYYSLETFTRLLTLKAKWPDRITLLRGNHESRQITQVFYIQNECQTKYGNANAWRYCCKVFDLLTVAAIIDEQVLCVHGGLSPEIRTLDQVRTIERNQEIPHKGAFCDILFVESIDVDTWAVSPRGAGWLFGSKVTHEFMHLNNLKLICRAHQLVHEGYKYMFEEKLVTVWSAPNYCYRCGNIAAVLAFTDVENRTAKLFHAVPDSERVIPPRNTPYFLMKNAEGETVKDTDLLTIEDLKEHARHIEKSVSSKEPRFIFRVLRALVTTRKKLNSKVLRKIICGFYTHSVEQRDSLLTFVEPMDTEQAVAGKAAQLPLLPELDVYFHLLVLLYLIDLQRIDQAVKCSNLLMQKVHGNNRRSLDLLASKCYFYHSRCYELADHLKDIRSFLHSRLRTATLRNDYEGQAVLLNCLLRNYLHYNLYEQAAKLVSKSVFPDMASNNEWARFLYYLGCIKAIQLEYSEAHKNLLQAIRKAPQHTAVGFKQTVNKLAITVELLLGDIPDRSIFRQPTLRRTLAPYFQLTQAVRTGNLGRFNEVLENFGAKFQADHTYTLIIRLRHNVIKTGVRMINLSYSRISLADIAQKLELDSSEDAEFIVAKAIRDGVIEATIDHEKGCMQSKENIDIYCTREPQAAFHQRISFCLDIHNHSVKAMRFPPKSYNKDLESAEEFSLKNELLLLHPKKEKLEMYIVLTQLCVALSAYIIHTVWPTAIADVAATVQPSHLILLELLTVLPEEFQTKGIIRHQVSELLLEVLSQDLHQMGLKCYYESLFRLSL-SVYNEEALEALSNVVTHPEARYPNLILKLLNSVIQLEDLLNKSLEEKDMDSIYGLFISFGEHHTKLLLDTLIEKPEFRIMKLIELIKQASATPGHYPVDELCSEQTFGFWYTLQDDIVASEP-PQFEATFNPVFHSLVDYLLKVQYPPDDLYLDDEKESFRCYRQDIGD--SFMYCYNILREAMLANLLGHLELA-QWQHLEASLFAFQSVAESVAFEEQHYL-PKFFNFLPKVP---RIFSAVMDAVGAYAEQVLGDVVPLLLLGLQNAP-ASTMALKDITRDCQACLKPFSEQILKYSQELKSREQVRLMNTIGQVLSMM-PNFILSYLDPVLTPMLQQLEHALTVTQKIMPVFNTVATVV-AQDEQVAEALCDALKRAVLTL-LEPIVEDILQLILRLYLDVTKQ-DLREHTSLLEIFYQTSQLIKKLDLSALFQCSPEKPTVKSLAEFINCSREVPLLNVVESLVVQVLRIIGSPRHVVEYMSDILMALNKKYRWMSTYLHSTEQKENFVRLILKERSNKRKLKETV--NEFTLRVKVKWKEVFPDVEVNTPMVFKAQLFALTGVQPDRQKVMLKGVTLKDESWGATVLLMGSKEELPGPTEKPVFMEDMTESELATALDLPAGLTNLGNTCYMNATVQCLRTVPELRDLRNFQGSITAALRDLYESMDTATIPPIILLQVLHMAFPRFAEKSEHGQQDANECWTEVMRMLQNFIDQYFGGTFVSLKCVESDDESKENFLQLSCFIS-QDVKYLHSGLKSRL-QETITKYSPTLNRDAQYKKSVISRLPAYLTIQFVRFYYKEKGSINAKILKDVKFPLRLDAFDLCSKELQQKLMPMRARFKEQEDKFSFPDDGSNNSGYYDLQAVLTHRGRSSSSGHYVSWIKREWFKCDDDKVVTSEEILKLSGGGTVHMFYCMVH---VF??????????????????????????????????????????????????????????????????????????????????????????????????????????????????????????????????????????????????????????????????????????????????????????????????????????????????????????????????????????????????????????????????????????????????????????????????????????????????????????????????????????????????????????????????????????????????????????????SSRHAKIFCPAHPDAPLIEDYRAGDMICPECGLVIGDRVVDVGTEWRTFNNEKSTNDPTRVGAAENPLLNGSDLSTVVGRTGDASDESGVAKYQNRRTMSSSDRALTNAFREINNMADRINLTKTIVRTTTLLFKQVHDGKTLKGRSNDAIASSCLYIACRQEGVPRTFKEICAVSKVSKKEIGRCFKLILKALETSVDLITTGDFMSRFCSNLGLPNTVQRAATHIARKAVELDIVPGRSPISVAAAAIYMASQASQDKKSQKEIGDIAGVADVTIRQSYKLMYPKAAELFPEDFRPSDGRPWIEKYRPVNFSDIVGNSETVSRLEIFAKEGNVPNIILAGPPGVGKTTTILCLARTMLGPSYKDGILELNASNDRGIDVVRNKIKMFAQQKVTLPKGKQKIIILDEADSMTEGAQQALRRTMEIYSKTTRFALACNTSDKIIEPIQSRCAMVRFGKLSDAQILSKLLAVCEKEDVSFTEDGMEAVVFTAQGDMRQALNNLQSTYSGFGHVNSDNVFKVCDEPHPLLIKDMLQHCVSGNFEDAYKIMAHLWKLGYASEDIISNIFRVCKNESIAEYLKLEYIKEIGYTHMRIVQGVSSLLQMSGLLAKLCQKVAVPDYLKKEHSLMKPYQGSGMTVPNWDFVGSTMVTSNYIRLTADEQSRIGAIWNKVPWELQVHFKVYGQGKDLFGDGLALWYAKEPLQIG-------HTFIKVFYISLIHQSN-HSHGHPYISAMVNNGTLHYDHDRDGTHTELAGCEAKFRGVDHETHISIRYEKDTVTVSTDIEGKNAWKECFKVSGVKLPTGYYFGASAATGELSDNHDIISMKLYELEEDRSNIEPSAASPRDHIDDPMSGTKLLLIMLCAIFGVFIVVFQKQQETSRKRFY--RGCVKDLR--NCLLSCAKRNGKTGDTVYYEIQLFSVGVYPVLANSSDSAHLLEVSIAVILFNSYILQKAPDEEPGFMYYFLSTIADVAANRFINSSAIYFAPNMSFTPSYKGFFNKTMPLFAPRAFRADDFNDPYHLEGTSTLNTIDAIDLGAISSNYTHEQYKINDWYFAWLPDQTRRHDSKTTYTVQITGTNETFVWHGPPAASDNPGPVKWTKPYFDCGRSNKWIVGASVPIPDIYPRHTGWRHIEIPTYVAVAVLELDFDRVDINQCPIGEGN-PRPNYFAGTARCKNRTTECEPIHGYGFRRGGYQCRCKPGFRLPRVVRNPYLGEIIERATQEEYNEGFHCKNINYLMVMTQNVESLMGFSTRLDPTLKGDIAFGKENQLENEARTAIRLANFISAMQQIIDPEELFAEFRVPDRPLNEDQVIGEVLANVIANEKIVGCGVFFNRNQFFAPY--AYRTKRNTQNFFVEDTTKIMIRYNSSGIRYDHYPLQYKAADIGYWTSPYFDCKGFHNEWLVTYAAPFFGWDKIKSRLEFKGAVTVMKLEELDINQCNAFKDTHKCDRKSSRCVPILGRGFMGGYKCECKQGYEYPFSDPVTYFDGQIVEAPNCLTSFRKFKLTNRAKYGGRFMVTMLPGDGIGPELMGHVKEVFRYAGVPVDFEEVHLESSQEDMKHVHKAITSIQRNGVALKGNIETRTNCPNFKSRNVELRLQLGLFANVLHCQSQPGVRTRHRDIDIVLIRQNTEGEYSCLEHENVKGVVESLKIITREKSEQIARYAFDYAQQHNRKKVTAVHKANIMKLTDGLFLQCCKEVAAEYSDIQFDNMIIDNCSMQLVANPHQFDVLLLPNLYGNILNNIACGLVGGPGLTSGRNYGNEYALFETGTRNTGKSIAGKNIANPIAMLNAGVDLLKHLGLTTHSKVISAAVDKTLNVDQIHTPDLGGQATTTDVMQNIIKEVVANTMKLQETPNEYKLENPPTDCVSSVKFGPNSNQFLLASSWDCSVRLYDVISNTMRLKYNHSGPVLDCCFQDAVHAWSGGLDCQVKVFDFNSSTESLAGNHDAPVRCVEFCPEVNMIITGSWDQTVKLWDPRTPCGAGTFSQPDKVYTMAVCGQKLIVGTAGRRVLVWDLRNMGYVQQRRESSLKYQTRCIRCFPNKQGYVLSSIEGRVAVEYLDPSPDVQKKKYAFKCHRIKDSSGMEYIYPVNAISFHNVYNTFATGGSDGYVNIWDGFNKKRLCQFHRYPTSISSLSFSTDGTVLAISSSFLYEQEDVKEIPPDAIYIRNVTDQETK-----------------------MKQYKPTDATTNPSLILQASSLPQYQTLIEKALKYGRGFDEQLSIAMDKLFVLFGCEILKIIPGRVSTEVDARLSFDKEGSIVKAKKIIQLYEEEGIGKERILIKLASTWEGIQAAKVLEEEDNIHCNMTLLFNFAQAVCCADAGVTLISPFVGRILDWYVQNTDKKSYEPEEDPGVLSVRKIYNYYKKYDYKTVVMGASFRNIGEVKALSGCDLLTISPKLLGELAESTEEVHAHLTEQSAKKEDLEKVEIDEKTFRWELNEDQMANDKLSEGIRKFAADARKLENILRERLKMGSLALRPLVTVYTEKNERSGASVALPAVFKAPIRPDVVNFVHMNMAKNRRHPYAVSEQAGHQTSAESWGTGRAVARIPRVRGGGTHRSGQGAFGNMCRGGRMFAPTKTWRRWHRKININQRRYAICSAIAATGIPALVMSKGHKIEETPEVPLVVSDKIQEYNKTKQAVQFLRKLKVWNDIQKVYKSKRFRAGKGKMRNRRRIQRLGPVIIYCSDNGLTRAFRNIPGIETLNVEKLNLLKLAPGGHVGRFVIWTESAFRKLDSLYGTWRKPSEKKKNFNLPMPKMSNTDLSRLLKSEEIRHAIRPVNRAIERRKLKKNPLKNIRVMLRLNPYAAVTRRNTILNAEKRKKMKILAKKRGIEIKPTRHQQAKKPKAKKV????????????????????????????????????????????????????????????????????????????????????????????????????????????????????????????????????????????????????????????????????????????????????????????????????????????????????????????????????????????????????????????---------MNNIFRVLHQ----YNTNNVKVAILGASGGIGQPMSLLLKNNPMITHLSLFDVAHTPGVAADLSHINTRARVTGHLGIEQLKESLDGAEVVVIPAGVPRKPGMTRDDLFNTNASIVRDLTDACAQTCPKAMICIIANPVNSTVPIASEVFKKRGVYDSNRIFGVTSLDVVRANTFIAEAKGLDPTQVNIPVIGGHSGVTIIPVISQATPAVSFKADELDAMTKRIQEAGTEVVKAKDGAGSATLSMAYAGARFTNSLLEAMKGKGVVECTFVKSSETEAAYFASPILLGPNGIGKNLGIGKLSPYETELVKTAMPELLSNIKKGEEFV--EFEWLLQEEVSAVLEQLILLECCHRLPV-PKTEKYFM-QVKVVVTLSGDNISHADINLRI-HKHSHRTIVQNDCQWKLQQIQDAGNHLMLALHLLKYNFRSAEEVTEMVNKLMSCLQRGRACLIIPKRKTIDELQKSRNMKSLQPPLPNDLAVSFYVQAHKLVFAVYHL-QKDQMKF-DAESSVPWLSEVLVLFTVSLQLCQQLKDKVDNIHYRGMVPAFIDIFKSLGADISDLQQIIGVCDVCFKDIESVL--NSIVS-LLVAFCEKLTKTPSVRICLR--VLQNLYEGLLFDVYYSLVRIAGDSISSVFTDVRKLKCWFSTEKAKKLLRLLHEVLSELASKVMIELLGTYTEDNASHARDDAHRCIVACLADPTMFLMDHLLALKPVRFLEGELIHDLLTIFVSEKLSSYLKFYNNNKDFL-NSLSLSHEQNMQKMRLLTFMQMGETKKEISFETIQGELQLKFEEIEGFVIDVLRTKLVRAKIDHVNKKVLVSSTMHRTFGKSQWQQLREVLNLSMVEPSMES????????????????????????????????????????????????????????????????????????????????????????????????????????????????????????????????????????????????????????????????????????????????????????????????????????????????????????????????????????????????????????????????????????????????????????????????????????????????????????????????----------MVLETAQDELVHYGVSVMELSHRSTTIMQGAKKDLRDLLNIPENYDILFLQGGGTGQFSAVPMNLCTADYLITGSWSAKATKEGEKYCAYNGIPDQSTWNLSPDAKYVYFCANETIHGVEFPIVTDMSSNILTKPVDVSKYGIIFAGAQKNIGIAGATVVIIRDDLVGHVPFCPSILDYKLNLQNNSVYNTPSTFSIYIMLVFKWIKKQGGAEAMEKSQVKSKLIYDVIDSSSGFYDRSRVNIPFRI-GGSEGSEYLEKKFLDEAHMVSLKGHRSVGGIRASLFNAITVDEVEVLYKDRPSWRDIEPIVQIAYSEKFRDVFDYFRAVLKDEKSERSFKLTEDAISLNPSNYTVWYFRRVLLQALKKDLHKELIYVQSIIEDNPKNYQVWHHRQVIVDWLEKQLTEAVLDPKNYHAWQHRQWVLEEFGIWEGEIEFVEQLLEDDIRNNSAWNHRWFCISQSKGFVL-YNIVYVLDCIKSNESSWNYLHGILSPYLLAFMVDRAIEIDKIRAEYWKFMARNLLRHIVGGLLLCLACTIAVSIFFALIVDRFEELLVFAPYMQNFLNEQKIKHKIFIINQVDQHRFNRASLINVGHLQSREECDYLAMHDIDLLPLNPALNYSYPEGGPFHIASPDLHPRYHYRTFVGGILLLTREDFELVNGLSNKYWGWGLEDDELYARMKKAKLNISRPKDINTGQKNTFKHIHDTKKRKRDTARLYNQREATRWRDHQTGLNTVQYRVVSKHKLMIDEASMTVINVELICNYTVTPWCDHKSVTPGDIITSDGFMRGHG-LVASVAGIVERVNKLVSVRPLKTRYNGEIGDVVVGRITEVQ--QRRWKVETNSRLDSVLLLSSVNLPGGELRRKTAEDELAMRKYLAEGDPISAEVQSVFSDGSLSLHTRSLKYGKLSQGTLVLVSPSLIKRRKHFHNLP-CGARIILGNNGYIWISVNEIWRETVARLRNCILALARHRLLLYDTTILYSYEAS-ISQLVRQKAQKVVGSWLMVCSGMVFGTVVLGGVTRLTKSGLSMIDWHPFKEFPPKTKQWEEEFHKYQQYPEYKLMNHEMTLQEFKKIWYMEYVHRMSGRSIGAIFFLPAAYFWYKGYFSKAMKPRVVAFAGLLAFQGLLGWYMVKSGLEEKPRVSHYRLAAHLGTAFVFYSLLLWSGLSHLLPTAQVRKFRMTKGVIFLAALSGALVAGLEAGLVYNSFPKFADRWIPSDILAYPKLRNVTENPTTVQFNHRILGETVVLGLWIYSRKVPLPPRARMAMNYVLVVALQVSLGIATLLFYVPKVLAASHQAGALTLLSTAIWLTHEMKLLRR-LAKWGEGDPRWIVEERPDATNVNNWHWTEKNASFWSQNLLKELLNDLTI-EGEVGRCITEITKVSGEAVANNRKGKLIFFYEWEITLHKDS-EIEGTAEIPNLSEENDPCEVDVNISV--QQLKDMMRTVGIQNIREKLAEYIKRLKDEFSQGMILPTKESNVLKVTPTKQRLDLTELNMESFKCKAEEFFRALTMVQAFTQGPCGGKFSLFDGNVHGIFVELKKIVQKWRFKTWPSGHYSDVLEITEKEDSTLTQKGVPRNELEQTREGWKNYYWQSMKRVFGFGAML????????????????????????????????????????????????????????????????????????????????????????????????????????????????????????????????????????????????????????????????????????????????????????????????????????????????????????????????????????????????????????????????????????????????????????????????????????????????????????????????????????????????????????????????????????????????????????????????????????????????????????????????????????????????????????????????????????????????????????????????????????????????????????????????????????????????????????????????????????????????????--------MCVLVGDGGTGKTTFVKRHLTGEFEKKYVATLGVEVHPLVFHTNRGPIRFNVWDTAGQEKFGGLRDGYYIQGQCAIIMFDVTSRVTYKNVPNWHRDLVRVCENIPIVLCGNKVDIKDRKVKAKSIVFHRKKNLQYYDISAKSNYNFERPFLWLARKLIGDPNLEFVAMPALAPPEVSMDPEWQAKLEKDMKEAQNTSLPDDDDDDLVAEFLTPILKESKFKETGVVTPEEFVICGDHLVHHCPTWQWASG-DKQYLPRDKQFLVTRNVPCYKRCKQMEEKLIETE-GGWVDTHSDEDDDEEAEDMDAFVDNEDKATEIVSTRTYDLNITYDKYYQTPRLWLYGYDENHKPLSIEEMYEDISQDHAKKTVTMETHPHLPGPPMASVHPCRHAEVMKKIIETVTEGGGLGVHMYLIVFLKFVQAVIPTIEYDYTQNFTM????????????????????????????????????????????????????????????????????????????????????????????????????????????????????????????????????????????????????????????????????????????????????????????????????????????????????????????????????????????????????????????????????????????????????????????????????????????????????????????????????????????????????????????????????????????????????????????????????????????????????????????????????????????????????????????????????????????????????????????????????????????????????????????????????????????????????????????????????????????????????????????????????????????????????????????????????????????????????????????????????????????????????????????????????????????????????????????------MFKNTFQSGFLSILYSIGSKPLQIWDKKVRNGHIKRITDNDIQSLVLEIVGTNVSTTYITCPADPKKTLGIKLPFLVMIIKNLKKYFTFEVQVLDDKNVRRRFRASNYQSTTRVKPFICTMPMRLDEGWNQIQFNLADFTRRAYGTNYIETLRVQIHANCRIRRVYFSDRLYSEDELPAEFKLYLPVQSKSKA---------------QIAI--CYQHQDLTKRDVLNAIHHYKNLIDRFVFNDGTTKDLMCLTGTIPVPFKYNIPVKIWLLDTHPVNSPMCYVTPTSDMKIKVSRNVDQNGRIYLPYLHEWNPNS-SDLIGLIQVMIIVFGETPPVYSK-PKPYP--NTGTISEEHIRASLLSAVEDKLKKRLREQTQAEIQVLKKTQDDLNNGKIKLDDIISKLEQEVELESEKNNEMKEVEIDDAVVTTAPLYKQLLNAFAEENATEDAIYFLGEALRKNVIDLDVFLKHVRELSRKQFMLRALMQRCRQKAGLPSNRKFFVGGNWKMNGSRSKIDDIVNFMVTGPVNTEVVVGCPSIYLDYSRKALPPTIGVAAQNCYKVASGAFTGEISPAMIKDVGVEWVILGHSERRNVFGENDQLVAEKVGHALAEGLKVIACVGELLEERESGKTEEVVFRQTKAIADKVSDWSKVVIAYEPVWAIGTGKTASPQQAQEVHAQLRKWLTDNVSADVASKTRLIYGGSVTAGNCKELAKESDVDGFLVGGASLKPDFVEIINAKAMAPEMERLRLFSHDKIRNFRTNNFLDRHPVCNGFALGKLGDTNTTLYITKMVTITNFVLGCCLILILNLLTEVETLLNNRVMTYELYAEKVAARGLCAIAQCESLRYKLIGGLAVRRACYGVLRFIMESEARGCEVVVSGKLRGQRAKSMKFVDGLMIHSGEPTNDYVETAVRHVLLRQGVLGIKVKIMLPWDRNGKIGPKRPLPDNVSIVNPKEETVPQQPYSEQK--AVSNDVVH-GGTPSPTSLPASGSKIDTLKQWSISTYKCTRQIISEKLGKGSRTVDSELECQIEQLRETQRKYANILRLARALASHFHVVQTQHALAESFSDLAHK-------ELQEEFLYNSETQRNLGKNGETLLGALNFFVSCLNTLCNKTIEDTLLTVKLYENARLEFDAYRSEARKNYKEKYEKLRSDVAVKMKFLEENKVKVMHKQLLLFHNAVSAY-FSGNQSMLESTLKQFNISWLEQ-MAELLLDSDIRLWVFLPIVILTFLIGIVRHYVSILISSTKKIELQQVQDSQALIRSRMLRENGKYIPKQAFLMRKHFFNNEESGYFK-TVKRAPAMQNPMTDPSMMTDMLKGNLTNVLPMILVGGWINWTFSGFVTTKVPFPLTLRFKPMLQRGIELSSLDASWVSSASWYFLNVFGLRSIYTLVLGENNAADQTRAMQDQMSGAAMAMPPDPKQAFKAEWEALEICEHQWTLDNVENEVCGL-----SVQQDVVDMALYKGAASEAGRAMQLKKKREKALEELEHQKKKIEEELKLSNIGNKFASHYDAVEQQLKSSTIGLVTLDQMRARQEDVVKERERQLAQKQEFLRQQEIEAKRRKKEQQKRQIASLSFKIDENEEEDDEEEEEEKESKTNDETDE------------------------------PVKKKKIGKNPNVDTSFLPDKEREEEEQRLREELRQQWEEKQQKLKSEEILITFSYWDGSGHRRSVKMKKGNSIYQFLQKCLELLRKEFNELRAVMADQLMYVKEDLIIPHHYTFYDFIVTKARGKSGPLFSFDVHDDIRLVSDATVEKDESHAGKVLLRSWYERNKHIFPASRWEPYDPTKNYEKYSISDKKK-LTISENKPL--LVSETLLKKRKKRAEQKAKAIQNAVRERKKQRQRKKGGFKSAEKLVKEYRRIERDNKRLKWAMRCPAKFDFPTEHRLGFIIRIRGSDGVCSRTSKILDLLKLRAVNIGVFVEMNEAMLQLLKVIEPFVTWGYPNLKTVRELIYKRGYGRAMGRRFPLSDNAFIEERLGKYGIVCLEDLLHQLYTIGPHFKKVANFLWKFKLNPPKNGWTKKSLRFTEGGDYGNRDIKINDLLRKMILTTTSLMVWWSKRYVA-----RARGARDSLSYSFDLSNRQLLCYIKISMDSPNVNRRLYQNAERIXIQHSLTSDLYEEIITFSTEQCLTLLRLWTMHINRGWIAMLIVGIISVTQVPYFIDTFDGFDTKVLEMKTVFNMKANFGRTRRMSALCVTGNKNGLAGFALGKSPEGRAAMRKTKKVAVLEIIF------PVFHDFYTCFGKTQIFVKKKPKGFGLVCHRAIKTICEIIGIKDLYAKVEGS-TNINNLTKAFFLGLQNQKTHQQLAQEKGLHVVEYREERDNFPQVIASPSKCRTMKEINPQEVIDYNEHINEGKVELIKTKRRPFYEYLDSWEYRVKKIERFRNHENVHVNLLVEHGKLTSFLNIEQVNTESNE--MDGQVRWTNKQRVLIFASRGITYRDRHLMQNLQSLLPHSRKESKME--KKDINEICEMKNCNKSIYFENKKKEDLYMWVSNVPRGPSAKFLVQNVHTMEELKLTGNCLKGSRPLLSFDKNFDKVPLLKELLVQVFGTPYHHPKSQPFTDHVFTFSILDNRIWFRNYQIMEE-DGSLVEIGPRFVLNPIKIFEGSFGGPVLYSNPKYISPNVHRAMLKKMSSGRYKLDEIFTTKKTFFRMAVGKNKGLVKGGKKGAKKKVVDPFTRKDWYDVKAPAMFSVRNIGKTLVNRTQGTRIASEGLKGRVFEVSLADLQNDEIAFRKFRLIAEEVQGRNVLTNFHGMNLTTDKLRSMVKKWQTLIEANVDIKTTDGYLLRVFCIGFTRKWPNQVKKTCYAQHTQVREIRKKMVEIMTREVSSSDLKEVVNKLIPDSVGKDIEKACQGIYPLHDVMIRKVKVLKKPKFELGKLLEIHGEGAGPTTTEGTVIERPEGYEPPIQEAVIKVGIVSGSGLD-DPDILQDETPYGKPSDA-LVTGKICGVETVLVARHGRKHSINPTEINSRANIWALK-QEGCTHILATTACGSLREEIHPGDFIPDQFLDRTRASTFYVCHITMHTPFTRQVLKDHESGTVITVEGPRFSSRAESNLFRCWGADIINMTTVPEVVLAKEAGLCYAAVAMATDYDCWH-DEQEAVHLEAVKVLKENGQKAIELLKKAVEIIVASCVL--------------------------MKILRHEEFEEGCKAACNGPYDGKWSKTMVGFGPEDNHFVAELTYNYGVNQYQLGNDFLGITIKSQTAIENAKNLNWPVKEDNNCYVIEAPGGYKFYLINDSQPQEKDPVQKVTLASSDLKKSVDFWHGLLDMKIYSQEEKTAVLGYSDNQCKLELCDIGTAVDHAKAFGRIAFSCPAEELQQIESKMKEEGQTILTPLVSLDTPGKATVQVVILADPDGHEICFVGNEAFCELSKVDPKADQLLEEAMAADKSDEWFAKKGISKAQARLPPLPTIKDIIRLYKLRALRQLSQNFLLDLLSDKIVKMAGHVCEVGPGPGNITRAILERGADRVTVIEKDRRFLPSLEFLASATN-GRLHIIIGDVLEYDIGHLF-NRELAKDWNDFHIIGNLPFNVSTPLIIKLLRQVSNREGPFAYGRTKMTLTFQEEVAERMIADQRCRLSVMCQNCKVEHGQAFLPKPKVNVGVVLLTPKQPLILPFEIVEKVARCLFHKYCKRDLVEETIRLADIDPTFHLSVEEVGQIAKAYDYICQQPKLKNYEFRMGK---MNRTLSWYFPLSLM-FHKSNENLLPLIPVVIEQTGRGERAYDIYSRLLKERIICVMGPITDELSSLVVAQLLFLQSESNKKPVHMYINSPGGSVTAGLGIYDTMQYILPPIATWCVGQACSMASLLLCAGAPAMRHSLPNSRIMMHQPSGHAAGQATDIQIHAEEILYLKKKLNSIYVKHTNQPIEKIETYIERDRFLSPEQAKEFGLIDFVLEHPPTHSEAHQAMRIDRPIGTWLLLWPAWSIGLATPAGHFPDLALFAAGALLMRGAGCTVNDMWDKDIDKQVARTKDRPLASGQLNMIDAWVLLGGQSLALFVLLQLNWYSIVLGASSLGLVVTYPLMKRFTYWPQVMLGLTFNWGAFLGWSSAVLPLYTACIFWTLIYDTIYAHQFKMFGLTRSMTQQKIRCENNNKNIIITVSTQHINYVTFFFYYAYSLKKHNILTILGICLKHSMFIYISRKPQTRKGKLYLENKEPKVQENAKAIFVRGSTASEVVLKAMKDFGNVHKPHGVFFNRKNPFEDPLSLEFFKKNDASLFMFGSHNKKRPHNLIIGRMYDYHVLDMLELGVESFQPLAEFEGPKIPVGTKPVLLFAGETFEDYQRLKNLFLDFFRGSRVRLQGLEHVIIFIAVEIYWRNYRILMKKSGTKIPR-IELVEMGPRMNLSVRRTKLASEDMFKLAKKQPSVLKPKKKKNVKQTALGSTVGRIHMKRQDFKLQTRKIKSLKVHLLPALSDNYMYLLVTKEAAIVDPVEPEKVTAAVNVKLTTVLTTHHHWDHAGGNEKLVVFGGDSRIGALTHHGD-EFKVGSLIRCLFTPCHTSGHICYFVVFTGDTMFVGGCGRFFEGSPEQMYQALLGALPDNTRVYCGHEYTVKNLKFAQTVEPENTIKEKLSWAEQEPTIPSTIGEEKTFNPFMRVAVQKHSDPIKTMAIIQLKNSNFVMNRLFGRSKPKEPPPNLTDCISNVDSRGESIDKKISKLDQELVKYKDQMKKMRDGPSKNVVKQKALRVLKQKRMYENQRENLMQQSFNMEQANYATQMLKDTKTTVEAMRTGVKEMKREYKKVNIDDIENLQDELEDMMGQAEEVQEALGRNYGMPEVDDDELEAELDALGDEIALDDTSYLDASKAPNAPTREPGSETINQEGVLVDDFRLPNITA???????????????????????????????????????????????????????????????????????????????????????????????????????????????????????????????????????????????????????????????????????????????????????????????????????????????----------------------------------------------------------------MEQMLRTFQEDLGSISHEILTLQEQSVSMNIRLKNRQAIRGELSQFVDDMVVPDVLINHILETPVVEKDFLEQIYVLDHKISFVKEQAFKEVHSCQDVRDILDKLKFKALSKIREYLLQRIYSFRKPMTNYQVTQDTMLKFKFFYQFLMTHEREVAREIRDEYVDTMSKIYFSYFKSYTTRLMKLQFEETADKDDLMGVEDTARKNILLKLKLKKNATIFTLGDRGNVLTTDLESPILVPHTAQKNETKYTFESLFRSQQYALVDNACREYLFLTEFFMVSGTGAQDLFHAVIGKTLTMFMKHVDVYIQDCYDSIAVFLCIHIIHRFQLLMHKRAVPALDKYWEALLQLFWPRFEYILQLNIQSVRDCDPQKFGSFDLRPHYITRRYAEFSAALVGINENFPSEQVTRLLAALQGEVENFILRMAAEFPGRKEQLVFLINNYDMMLSVLLERTREDSKESESFKELLNARTHEYVEEILSPHFGGLISFVKMCENYLERGQSEHLKKEEKKVPQLVRSFNSGWKGSLDDINKDIMKSFTNFKNGTNILQVALTQLIQYYHRFHKVLSQNPFKNLPIRSELINIHHLMVEVKKYKANFMIKPFFFLFISYI----SVHQQYQVWDACNEPCFSLRLTKKIIMSLYREDTRKDKYFVVLQITGPDGKVIYKGERETNGKYTFAAHMDGVYTYCFSNAMSTMTPKVVMFSMDVGNTPK---EQDNDAHQNKLEDMIKELSTSLTAVKHEQEYMAVRDRIHRAINENTNSRVVLWAFFEAIVLVCMTGGQIFYLKRFFEVRRVVMRLFGILRLPKDYSNFPKQYVERAMEQVGWRTPKGPQYLRKEIKKKYNMSRPWTEEFRKNEPGTHIFLEPVFRGDRVEVMVGKDKGKQGIVNYIVKERNWVVVEGLNCHEGPLLVTGQVSLVDPSDNKPSEVEWRYTDEGEKVRVSVRTGRIIPIPKAEETHDYKYLEQPKDTKSEEISKITFEP-KLMTFEQDIMNHLGIKEERQPHRTYWYMSDNQSS-VIGHMTSNKVSALLWMTRMTTMFLTFCYLIPNPYSCYYKALMSNAATSALRLHQRLPNFQFNREFLALLLIEDSCHYLFYSLIF-LPIT-LVLLPIFLFALLHSISILDKLGSLVG---QQINILRLVAFTEIFLMPLTLFMIFTGGSLLTPFVYYRFLTLRYASRRNPYTRNVFHELRLSIEMQANPRCPETLRRIAFVTRLAPTILVAIIVYYNFPELQEDEKLHIKLPKNIDDAKNLGKVLSRYTGDTVIGGYFLTYIFLQSFAIPGSIFLSILSGFLFPFPLALTLVCLCSALGASFCYILFYLVGRKLVLKYLPHLLNYIIFLRITPFLPNWFINITSPVIDVPLAPFFLGTFLGVAPPSFVAIQAGTTLHQLTSSFSWTSLTVLIVFALLSMIPVLFKLKEKF------MERVCVAHHENRTTDRISLYYKKTQLIVKITKIIFFWEKKDKRPTILTINFMKCSFCCYKCPFEAISIINLPSNLEKDTTHRYNQNSFKLHRLPTPRPGEVLGLVGTNGIGKSTALKILAGKLKPNLGRFNAPPDWTEILTYFRGSELQNYFTKILEDDLKAIIKPQYVDQIPKAVKGSVQQLLDKKDELRNQDTVCEMLDLKNVRDRNVENLSGGELQRFACAMVCIQKADIMIIIMCTMYLYLRQLFTCVRSQFFTVSVNIVIMMLMIDMRFVSYIQNFIKLLFIDRACYNYKCFYFFFTIGINIFLDGFVPTENLRFRETSLIFKVAETAEEEIKRMCRYEYPTMKKKMGNFSLSVEPGTFTDSEIVVMLGENGTGKTTFIQLLAGRLKPEGGCEIPSMLISYKPQKISPKSQGTVRQLLHEKIRDAYVHPQFVADVMKPLQIDNIIDQEVQNLSGGELQRVALVLCLGKPADVYLIDEPSAYLDSEQRLVAAKVIKRFILHSKKTGFVVEHDFIMATYLSDRVIVFEGQPSVKAVASSPQTLLAGMNRFLELLNITFRRDPNNFRPRINKLNSVKDTEQKKSGNFFFLDD?????????????????????????????????????????????????????????????????????????????????????????????????????????????????????????????????????????????????????????????????????????????????????????????????????????????????????????????????????????????????????????????????????????????????????????????????????????????????????????????????????????????????????????????????????????????????????????????????????????????????????????????????????????????????????????????????????????????????????????????????????????????????????????????????????????????????????????????????????????????????????????????????????????????????????????????????????????????????????????????????????????????????????????????????????????????????????????????????????????????????????????????????????????????????????????????????????????????????????????????????????????????????????????????????????????????????????????GLIIILIVLLIATGKVGAKKRKKLEMKAEKRAQREREEEEREERRQRNALEEERKKEEEKQKEEEQKREEEEKRVREEKEKQEYEEYLKMKEAFSVEEEGYDEAADEQESHNKLQEFVDYIKTQKVVLLEDLAAKFKLKTQEAVDRVQELLSQERLVGVIDDRGKFIHITREELESVAQFIHLRGRVSITELMESSNNLINLQPDVEHLEAEAKATAAKHVANMLQRPDQLEKVEQYKRRVIRKKASVEAMLKTAMQSQLDGVKTGLSHLETALSDIKEIKQRDIEDSFEIPRLEKLQDVREESLKHSQYAAAMENLKHIFNVPESVQKTQEWISDGKLLLAHQLADLENSRDDLLFEMHKLPNQSPTDRNMLKQYFSDVEKLSEDLGKQLWLILRRTLNSVRKEPQVIVTALRIIEREVRAAIARQQTT-GFMPTGRPKRWKKRCFEILEMSVQDRIEGNQFEERSQNKMWLVRHLEVTRQLILEDLKVVKTACVPCFPPSYDIVEKVKMYHDSLSKHLQDIISQLEGNEYITLLNWLNVYESSELMGHPDLMIDVSLLDNVVENLICRYVSMLETNYQDWMKNTVMDVKDWPEADSEGYFHTSLPVIMFQMIDQHIQVAKTVELVERVLMVSIEQVVSVTRLYKEAIVEYHFEDRSFTHYMIAIANNCLQFV-FESLKMDTLGYLEDELFLDLDKINDLLTDTICATVEDYCHDYIQNRVAKGYITAIL--QKKLSLKNYEERKEVADKKKLRESPFDALPLLAEVLKLKDGSLLSLEISG--------MLAGLCLADDTTTEEAEIKVDQDIGSSREGSRTDDEVVQREEEAIKLDGLNVAQVKELREKAEKFVFQAEVNRMMKLIINSLYRNKEIFLRELISNASDALDKIRLLSLTENEALSATEELSIKIKVFFDIHSEHSTDSKVFNTQ-----NLLKVCESFQSQFVKVIKK----KNMGHIWGQFGVGFYSAFLVADRVVVTSKHNDD-KQYIWESDSGSFSIVEDPRGDTLKRGTTVSLYLKEEARDFLEEDTLKQLIKKYSQFINFNIYLWTSKTETVEEPVEED--EEEKEKP-ETEEDGKVEEE---EKPKTKKVEKTTWDWELMNSSKPIWQRKPADINEDEYKDFYKSITKDTSDPLTKTHFIAEGEVTFKSVLYIPSSQPTETFNRYGSKVDHIKLYVRRVFITDDFQDMMPNYLNFIRGVVDSDDLPLNVSRETLQQHKLLKVIKKKLVRKTLDMIKKIPKEDYEKFWKEYSTNVKLGIIEDPTNRTRLAKLLRFISSN-DPEKLTSLAEYVERMKEKQEHIYYIAGSSLEEVKKSPFVERLLKKGYEVLYLTEPVDEYSVSALPEFEGKKFQNAAKDGLKLDESGKAKERLEDLEKEYEPLTKWLQDESLKDKILKAKISQRLHNSPCALVASQFGWTGNMERLARSNAHSKTYDTTRDYYLSQKKTLEINPRHPLIKELKKRIEDDKDDPTAKNMANIMFETATLRSGYILDDTFSFAERVETLLRKTLGVPEDAQIEDEPEEE---EAEKEEEDDEGETE-----???????????????????????????????????????????????????????????????????????????????????????????????????????????????????????????????????????????????????????????????????????????????????????????????????????????????????????????????????????????????????????????????????????????????????????????????????????????????????????????????????????????????????????????????????????????????????????????????????????????????????????????????????????????????????????????????????????????????????????????????????????????????????????????????????????????????????????????????

>Ixodes_scapularis

PDSRVNSLNAEVMESAVLISSKPGCFIAGADITMLDKVTSHASELKPIVAAIMGSCLGGGLEVAMACHYR-IAVKERKTVLGLPEVMLGILPGAGGTQRLPKLIQLPTALDMMLTGRNIRADQAKKMGLVDQTVELLGPGLYLEEVAAKGLASNTLTPRTRPLMERLARNY-VFEKAKGQVMKMTQGLYPAPLKILEVVRTGIEKEGFGELVTPQSKALIGLYFGQVTCKKNRFGEPVKTVGVLGAGLMGAGIAHVSVDKGYNVLLKDGLGRGEQQIKKKRLEKDRFMSALEPTLKYDDIVIEAVFEDIGIKHAVLKEVEQPHCVFASNTSALPIAKIAEASKRPEKVVGMHYFSPVDKMQLLEVITTDKTSQDTAAMAVDVGLKQGKVVITVKDAPGFYTTRILSAMMAEALRVLQEGTTVKELDSLKKFGFPVGAATLMDEVGIDVASHIAAKEFGARHDLVAGFHGRKSGKGCYLYRPTNTLLKKYTVEELQMRLAGRFVNEAILCLQEGVLANPVEGDIGAVFGLGFPPFLGGPFHYVDTYGADRLVQLDKFAQFEPCQLLRDHA-NDTAKKFHMQAPIILLKEGTENSQGKSQVISNINACQVIADAVRTTLGPRGMDKLIVDNSGKATISNDGATIMKLLDIVHPAAKTLTDIAKSQDSEVGDGTTSVVLLASEFLKQCKPYIEEGIHPQIIIRSLRKAAHLAVSRIREIAVAVEKNGQRELLEKCAMTTLSSKMIAGQKEFFARMVVDAVMQLDELLPLNMIGIKKVQGGALEESLLVSGVAFKKTFSYAGFEMQPKKYQNPKIAMLNVELELKAERDNAEVRVQNVQEYQNVVDAEWEVLYEKLRKIHESGARVVLSKLPIGDVATQFFADRDMFCAGRVVDEDLRRTAKACGGAILSTVFDLQESNLGRCELLEEIQIGGERYNLFTGCPETRTVTIILRGGAEQFIEETERSLHDAIMIVRRALKNDAVVAGGGAIEMALSKHLRDYSRSVAGKEQLLVAAVAKALEVIPRQLCDNAGLDATTLLNKLRQRHAAGDLWAGVDIQAGDVADNLAACVWEPAVVKTNALVAATEAACLVLSVDETIKAPKSQGDDERGRPFMPERSAAVASKEHALAVSRDYISQPRLVYKTVCGVNGPLVILDEVKFPKYAEIVHLVLADGATRTGQVLEVSGSKAVVQVFEGTSGIDAKNTVCEFTGDILRIPVSEDMLGRVFNGSGKPIDKGPPVLAEDFLDIQGQPINPWARIYPEEMIQTGISAIDVMNSIARGQKIPIFSAAGLPHNEIAAQICRQGGLVKLPGKSVLDDNFAIVFAAMGVNMETARFFKQDFEENGSMENVCLFLNLANDPTIERIITPRLALTTAEFLAYQCEKHVLVILTDMSSYAEALREVSAAREEVPGRRGFPGYMYTDLATIYERAGRVEGRNGSITQIPILTMPNDDITHPIPDLTGYITEGQVYVDRQLHNRQVYPPINVLPSLSRLMKSAIGEGMTRKDHADVSNQLASTASVGQGVYVHIGIVGTQCTHSEKVTGMPFCA---------GSYENRTIFESLDIGWQLLRIFPKEMLKRIPQALLQEFYPRDGRQRSLTTSCFNS-TGLFGEPNGFYLLKEQAIQDAETYVDEATDRSRKMVQVFDDLSDALCRIADMAEFVRIGHPENRFSSAALDASVSISNLVEKLNTNKNLYEALKRVTEHG-DVVPTTEDDYVSKLFLFDFEQSGIHLDAETRKKVVSLNDYTLHVGSYFANNALQARAVKKSELPENIVIVPGLYADANNELLREAAYRAYLYPDKHQSELLDELLAARHQLAVLCGFPTYAHRALRGSIAGSPDGVEFLGILSAQLKPRAEQDYKEMLNMKPWDVPYYTPYLSLGCCMDGLNEIFHSLYGISLEAEGEVWHPDVVKLAVKEENRLLGYIYCDFFERQEKANQDCHFTIQGGRLPDGSYQTPIVVLMLNLPPPLLTPSMMDNLFHEMGHAMHSMLARTRYQHVTGTRCATDLAEVPSILMEYFSSDPRVVSMFARHYQSGEPMPYQMALSLRHLRYHFAASETQLQVLYALLDQRYHSHHPLSTTQVLAELQDQHYGLPYVEDTAWQLRFGHLVGYGAKYYAYLVSRAVAAWTWQEVFKRDPFHKEAGNYRQKLLSHGGSVPAKQLVSDFLLATFLISDI????????????????????????????????????????????????????????????????????????????????????????????????????????????????????????????????????????????????????????????????????????????????????????????????????????????????????????????????????????????????????????????????????????????????????????????????????????????????????????????????????????????????????????????????????????????????????????????????????????????????????????????????????????????????????????????????????????????????????????????????????????????????????????????????????????????????????????????????????????????????????????????????????????????????????MVLADLGRKITNALRSLSNATIINKEVLDSMLKEICTALLEADINIKLVKQLRENVRSAIDIDEMGAGLNKRRMIQSAVFKELIKLVDPGVKAWQPTKGKNNIIMFVGLQGSGKTTTCTKLAYHYLKKGWKTCLVCADTFRAGAFDQLKQNATKARIPFYGSYTEVDPVVIAQEGVDKFKSEGFEIIVVDTSGRHKQEDSLFEEMLQVSNATNPDNIIFVMDASIGQACESQARAFKEKVDVGAVIVTKLDGHAKGGGALSAVAATHSPIIFIGTGEHIDDFEPFKVKPFISKLLGMGDIEGLIDKVNELKLDDNEELIEKLKHGEFTLRDMYEQFQNIMKMGPFSQIMGMIPGFSSDFMTKGNEQESMGRLKKLMTIMDSMNDQELDHREGAKLFSRQTGRVTRVARGAGVTTREVQELLAQYTKFAAMVKKMGGIKGLFKGGDMAKNVNPAQMNKLNQQMAKMMDPRVLHQMGGFSGLQNMMRQLQGAFLLDDPSYKKLQEYYNAKGKTLNMPALFKEDPERFRRYSVRLKTPVDGLLVDYSKNLINHDVMTRLFDLARSRKVEPARDAMFQGEKINFTEGRAVLHIALRNRSNNPIMVEGKDVMPSVNAVLKHMREFSDSVRNGVWKGYTGKSITDVVNIGIGGSDLGPLMVTEALQPFQKGPNVHFVSNIDGTHLAKTLKKLSAETTLFIIASKASSHKYNDIGAIFTKMRVLCSCQILISVARGNAPLESNIPVILALLESSYRMLLWSTWFAHCFSLFTTLTLTVILLKSCGIFLKVLTGTRFKRSHLFCCPQKATTELLMSSIRVHQPQAKKKTAYPVISPSEYLHRFAAYFQQGDMESNGKYVTRSGQRVAYTTGPIVWGEPGTNGQHAFYQLIHQGTRLIPCDFIAPVKTHNPISHGIHHEILLANFLAQTEALMKGKTKEEAEAELKKAGMTGEVLQKILPHKVFEGNKPTNSLMVDKMTPYSLGMLIAMYEHKIFTQGIIWDINSYDQWGVELGKQLAKVIQPELRGKSEVSTHDGSTNGLINFIKSYNEKATYKDKDKPSQVRQSNITAAKAVADAIRTSLGPRGMDKMIQAANGDVTITNDGATILKQMQVLHPAAKMLVELSKAQDVEAGDGTTSVVVICGSLLDAAAKLLLKGIHPTIISESFQRAASMAVEVLESMAHPLDLSDRDSLLKSATTALSSKVVSQHAGQLAPMAVDAVLRVVDPTNVDLRDIKVIRKLGGTVEDTELVDGLVFTQRLAGSGGPHRVEKARIGLIQFCISPPKTDMDHQVIVSDYTAMDRVLREERAYILNIVKQIKKAGCNVLLIQKSILRDALNDLALHFLAKMKILVVRDIEREEVEFVCRSLGCRPVASLDHFGPEALGSAELVEEVASAKYIRVTGVAHP---RTVTLLVRGSNKLVLEEAERSLHDALCVVRCLVKRRALIAGGGAPEMEISLRLAERARLLSGLDAYCVGAFAEALEVVPYTLAENAGLHPIATVTELRNRHAKGERDAGINVRKGSVTNILEENVVQPLLVSTSAVTLAAETVRSILKIDDIVQTVRMDKKNVMYFWDPDVGNFHYGPGHPMKPQRLSVTHSLVLHYGLYKKMQASQLSSSNSHDMCRFHSEEYIDFLERVTPQNIQTFTKSLSHFNVGDDCPVFDGLYDFCSMYTGASLEGAVKLNNECCDIAINWSGGLHHAKKFEASGFCYVNDIVIAILELLKYHPRVLYIDIDIHHGDGVQEAFYLTDRVMTVSFHKYGNYFFPGTGDMYELGAESGRYYSVNVPLKEGIDDASYFQVFKPVIQGVMEFFQPSCIVLQCGADSLAGDRLGCFNLSIRGHGECVRFIRELGLPLLVLGGGGYTVRNVARAWTYETALLVDEPVSSEIPYNEYFEYFAPDFTLHPE-------ENANSKQYLETIVRAVSENLKCLVHAPSVQMHHVPPDMLKPGST-SAVPTPVEPALSTTPTAAATVAEMSDEDDFMCDDEEDYDLEYSEDSNSEPDVDLENQYYNSKALKEDDPTAALASFQKVLDLEAGDKGEWGFKALKQMIKINFKLSKYEEMMARYKQLLTYIKSAVTRNYSEKSINSILDYISTLAEMELLQEFYETTLDALKDAKNDRLWFKTNTKLGKLYFDRSEFNKLAKILKQLHQSCQTDDGADDLKKGTQLLEIYALEIQMYTAQKNNKKLKKLYEQSLHIKSAIPHPLIMGVIRECGGKMHLREGEYEKAHTDFFEAFKNYDESGSPRRTTCLKYLVLANMLMKSGINPFDSQEAKPYKNDPEILAMTNLVSAYQNNDISEFELILKTNRRNIMDDPFIREHIEDLLRNIRTQVLIKLITPYTRIHIPFISRELNIDSNEVENLLVSCILDSTIQGRIDQVNQVLELDSKGQGAARYNALDKWTAQLGTLHQTISSFIANVGSLNAVRILKGEAEEEKAETARLSSFVGAIAIGDLMKSTLGPKGMDKILMCNNRGESKVEVTNDGATILKAIGIDNPAAKILVDISKVQDDEVGDGTTSVTVLASELLKEAEQLISKKLHPQTIVAGWRKATAVARQALEAFAADHSANAEQFREDVLNIARTTLGSKILSQHQEFFAKLSVDAVMRLKGSGNLDAIQIIKKLGGSLTDSYLEEGFLLDKKPGVHQPKRVEKARILIANTPMDADKVKVFGSRVRVESVAAIADMELAEKVKMKEKVDQILKHNISVFINRQLIYNYPEQLFADAGVMAIEHADFDGIERLALVTGGEIVSTFASPELVRLGTCDLIEEVMIGEDKLLKFSGVPLGEACTIVLRGATQQILDEAERSLHDALCVLSQVVKDNRVVFGGGSSEMLMASAVSKLAESTPGKESLAMEGFARALRQIPTIIADNAGFDSAQLVSELRAAHSEGKKTAGINMTEGCVDDMCKLGVTEAFVVKRQVLLSASEAAEMILRVDNIIKAAPRQRHPDRSHMAAEIKPGSKPQLINKLVGHQEAVNVAVIIPGEDGVISISDDRTVRVWLRRDTGQYWPSICHNMPSAASSMDYNSETRRLFIGMDNGSISEFLVADDFNKMTHQRNYLAHQGKVVGIVFSLIAEWVLSVARDKYFQWHCSETGRRLGGFQCNAWCTALQFDAQSKHAFIADYSGHITMVKVEETGYKPVTTLKGHSGSIQCLSWDAERKLLFSGGFDQTIIVWDIGGKQGTAYELQGHHNKVTALCYHSPGKLISASEDSTLVFWNMTTKRIETPEWGESDTCQRCSRPFFWNIKAMMDQKTIGIRQARRSLCGKAVCDKCSSNRSRIPIMGYEFDVRVCDECHVIITDDDRVSMATFHEARHPVVHMHLDVTRGHLLTTGSDRVIKIWDISHMLMAKSQPDGPLSKKALKKQAKEQEKAAKKSSRQSAESKQAVDTEVDYSEGHYGKMAMLQSSEKVSRELAKVKDLSSSIAGSYIWIRGRLHTSRSKGKQCFFVLRQQQYTVQCLVAVSEKVSKAMVKFCALVTKESLLDVYGIVQSSPQKVEGCTQQDIELHIEQAGAPSRFERMLYFQLDQNRTPAQD----EGLNIRVNQDTRLDNRILDLRTPANQAIYRLQAGVCSLFRESLAARGFVEIHTPKIISAASEGGANVFEVSYFKGSAYLAQSPQLYKQMAIASDFDKVFTVGAVFRAEDSNTHRHLCEFVGLDLEMAFNYHYHEVLDIIGQMFVDIFKGLRDRQATLICVMDFTYYCRFLHSRKQSLRLEFPEAVAMLRAAGVEMDDEEDLSTPNEKLLGRLVKAKYDTDFYILDKYPLKVRPFYTMPDPHNAKVSNSYDMFMRGEEILSGAQRIHDPTFLTERAKEHGSSVTLLQAYIDSFRYGVAPHAGGGIGLERVTMLYLGLDNIRKTSMFPRDPKRLTPMLKEGLSVADIPPSEFDRIKIIYPAECRNRGTTYRGKLDVTLWSLNGVQQDVIKKTAGEVPIMVKSQMCNLSKLSPKELVQRGEESEEFGGYFVVNGNEKIIRMLIMTRRNYPIAMMRNSWKNRGKMYSEYGVSLRSVKPDGTNMVLHYLTNGTVQVMFSYMKEIFFMPVMMLLKALCDVTDYHIYSELVAGKENDSFYKGCIINMLRQVQENLLTDEVRSYIGGKFRWYSDEQVALFLLRHCICIHLDSNFDKFNLILLMIKKLFALAKGECAIESSDNPMNHEVLLAGHLYLMVLKEKLGSFLFSIRQNIEKKFSKVLEVTGAMNYFLATGNVVTKSGLGLMQFSGTTVLAEKLNYWRYLSHFRCVHRGAFFAEMRTTTVRKLLPEAWGFLCPVHTPDGSPCGLLNHMTAMVEMVVVLDGRVLGYVLRTMKAHGKVPPTLEIGFVPKTSKASQYPGLFLFSTVARMMRPVLNATGTVEWIGTFEQVHLNISVIPDEATTHQELRQTSMLSVLGNMIPYSDFNQSPRNMYQCQMGKQTMGTPCQALRYRSDNKLYRITPQTPFVRPTAYDHYHMDDFPTGTNAIVAVISYTGYDMEDAMVLNKSSVERGFKHGVYKTELVNLRVDLDGLPYIGVQNDPVCSFVETKVHKYKSTESAYIHDVKLLGNLQNICITYWIRNPMIGDKFASRHGQKGVCSQLWPIENMPFTESGMTPDIIFNPHGFPSRMTIGMMIESMAGKSATLHGFVHDATPIEIEDISMFVQCKREFLLAAGYNYHGTERMYSGVDGREMEADIFFGVVYYQRLRHMVADKYQVRTTGPVDTLTHQPVKGRKRAGGIRFGEMERDSLLAHGSSFLLQDRLFNCSDRSLCRKCGSVLSISMPYVFRYLAAE-LAGINMGVLRETKHDIHKVQRNDPSLHPFEVCREYTRAMNAVKLDKIFAKPFLGSLGHRDGVQVLQKHPKSLSHLISGACDGEVKIWNLAERKCVRTIQAHRGMIRGITGDDKNIKTIITKISHHQENLFATCGEDVHLWEERSEPLRTLW-GVDTVYVRFNPIESASSDRSIVLYDTRESQPLRRVILEMRSNALCWNPMEAFIFTCANEDYNLYTFDMRRLKSPLSVHMDHVSAVMDVDYSPTGKEFVSGSYDKSVRIFHSREVYHTKRMQRLTCVLWSLDNKYIMTGSDEMNIRLWKACASEKLGMLQYQAKLKEKFAQHPQVKRIARHRHVPKHIYQEKRTMLEARKRKLGRQLLSKGQYADALSHYHAAVEGDPENYLNYYKRATVFLALGKSKPALEDLHEVIALKPDFLAARHQRGTVLLKQGNLDEAHIDFEWVLRLDPNNPDASIEPLKRDAIEVLTRVWDVTLREMRASCYENLGDVMNAIMDLRPTTKMVPDNTVGYLKLSKLYYKLAEAEESLNVIRECLKLDPDHKDCYKKVKKLARSIQDECVDKLVQHVRGRCHCHSKAGEALETCSEALKLDPALCDRAEAYLNDGQYDHDFQQAANADE-GPEGLKRAQRLERQSKKRDYYKILAAKREILKAYRKLAQKWHPDNY-QGDS--KKDAEKKFIDIAAAKEVLTDPEKRKRFDSGEDPLDPESQFTYKFVFMLVLFESPAGYAIFKVLDEKKLQQTDNLFKDFETPDKASRVVKLKHFEKFEDMTQALAAATSAIEGKMSKTLKKVLKKVVAKEAHESLAVADAKLGNVIKDKFDISCVANSSIQELMRCIRSQVDGLITGLPRKEMTAMALGLAHSLSRYKLKFSPDKVDTMIIQAVSLLDDLDKELNNYIMRCKEWYGWHFPELSKIVTDNLQYVRTVQKVGLRTNAIETDLSDILAEDLEAKVKEIAEVSMGTEVSDEDIRNILHLCEEVLQMSSYRSQLYEYLKNRMTAVAPNLTILVGELVGARLISHAGSLLNLAKHPASTVQILGAEKALFRALKTKHDTPKYGLIYHAQLVGQSTQKLKGKMSRMLAAKASLATRVDALGEDGGTELGVEHRAKLETRMRVLEEGGNRRISGLGRGRAAQDRYEHKSAVRQYQPGADSTLP---------------------------------------SASGGKKRKFKEEDDEAGPAPAEEVEEDTKPKKKKK--KKDESAEAAEEEQVTVKPEPVTPKKKKKKKKHAE???????????????????????????????????????????????????????????????????????????????????????????????????????????????????????????????????????????????????????????????????????????????????????????????????????????????????????????????????????????????????????????????????????????????????????????????????????????????????????????????????????????????????????????????????????????????????????????????????????????????????????????????????????????????????????????????????????????????????????????????????????????????????????????????????FKIVRRLHQLATTFSQTLLNVPETRVTTLDNGVRVATEDSGNPTCTVGVWIDAGSRYENEKNNGVAHFLEHMAFKGTSKRSQTDLELEVENMGAHLNAYTSREQTVYYAKCLSKDMPRAVEILSDILQNSKFGEAEIERERGVILREMQEVETNLQEVVFDHLHSVAFQGTPLGLTILGPTENIKSIQRQDLVDYISLHYKGPRIVLAGAGGVNHDELVKLASQHFGSMTTDYDAKLPCRFTGSEVRVRDDDMPYAHVAIAVESCGWADPDNIPLMVANTLIGNWDRSHGGGANVSSRLAEECVKDNACHSFQSFNTCYKDTGLWGIYFVSEGREMDFLVHAIQREWMRICMSATEGEVTRAKNLLKTNMLLQLDGTTPVCEDIGRQMLCYGRRIPLPELEARIDAVTAQTVRDVCTKYIYDRCPAVAGVGPVEALTDYANVRSNMYKHSVHTLVFRSLKRTHDMFLSDQANPPPQDETSEKLKVSIKTKDEYGLVMHLVKSNNRPNVEQMMVLAGQLAVAPRKPVTIPKPQWHPPWKLYRVISGHTGWVRCLAVEPGNQWFCTGSNDRIIKIWDLASGKLKLSLTGHISGVRGLAVSPRQPYLFSCGEDKQVKCWDLEYNKVIRHYHGHLSGVYALSLHPTIDVLVTGGRDATGRVWDMRTKASIHSLVGHSNTVASVQSQAAEPQVLTGSHDCTIRLWDLVSGKSRVTLTHHKKSVRALLVHPKLYAFASGGPDNIKQWKCPDGKFIQNLSGHNAIVNCLGMNDDGVLVSGGDNGTLFFWDWRTGYNFQRLQAPVQPGSIDSEAGIFACLFDMSGSRLITAEADKTVKIFREDDTATEETHPVNWKPDIVVHSSSEFRINVSLDEYLEDLSINVKAKNFLVFQGDVETIAMKNPKERTLLFEEISHSMIKAEEDTYQKKKGIAAEKKEARLEEADKYQRLKEDLQVVCQTFKLYHIQDEMSHKRRKEKVEEEVRDKRKIEQQIREDVELNKKKPAFIKAKERTAHMQKKLEAARKSYKAAKKVDETHQGISELEEELEEINREFEHQDFSLEDSQLREYHQLKEEAGRQASLHLQNLDSVRRDQKSDQDRHDNEVRKRQETQNKARELDENLRRVDKLGELEELRRQEQEVEAKARINRELEALNEMGDAKVDKHEDSRRRKKAEIVDHFKQLYSGVYDRLVNMCQPVHKKYNVAITKVLGKNMEAIVVDSEKTGRACIKYLKEQMLEAETFLPLDYIDAKPLKERLRTISQPKNVKLLYDVLQYDPPAIKRAVLYATNNALVCETADDASRVAYDLGDNKRYDAVALDGTYYQKNGFISGGSTDLAKRARRWDDKAFHTLKQKKEKLTEELKEMMKRTRKESDLTTIQSQIRGLETRIRYSVVKKIEEEMRKRELKIDELKQAQNGIEDRVFSDFCASIGVENIRQYEERELRATQERDKKLEFENQKNRIVNRLEYER--SKDTSEVEDDERELENLKEAEANQMQMIDDQMQSLEKLKSSKLGKKQDLDKAEEQMAEIRKRLAAVQKDIAVVQKSLTSLEARQEQRRLDRHSVLQTCKLEQIPLRLLDGTLTRLYEREQLRIDYSILRLSKEIALQRTQAPNMRAMEKLDGVKERLKETDTEFENARKKAKMAFEKIKRERTCFDRVSNRIDEIYKALTNNQSAQAFLGPENPEEPYLEGINYNCVAPGKRFQPMSNLSGGEKTVAALALLFAVHSFQPAPFFVLDEIDAALDNTNIGKVAHFIREQTETSFQCIVISLKEEFYSHADALVGIVPDPGECTVSRVLTMDLSLSEAMLLYLKKAIQEGNVFEIQNIYENNLAKLMEKYFMILYKE--LYYRQIYADERFESYYNYCALFNYILSTGPVALELPNQWLWELIDDLLQNF--QSFSQFIWNVHSVLNVLHSLVDKSNINLQLEVYTKGGDPDNVAGEFGRHPLYKMLGYFSLIGLLRLHSLLGDYYQAIKVLENLELNKKSLSRVPACQITTYYYVGFAYLMMRRYADAIRTFSNILVYIQRTKRSYQVDQINKQTEKMYALLAICLTLHPQRIDESILSQLKDKYMNKMQKGDKGEFGDCPKFLNPVQLNVFLSEVQGQITIRSFLKLYTTMPVSKLANFLLLSFKHKMKNVVLDGEFQSGSDVDFFIDGDMIHIADTKVARRYGDFYIRQYHKFEELYR???????????????????????????????????????????????????????????????????????????????????????????????????????????????????????????????????????????????????????????????????????????????????????????????????????????????????????????????????????????????????????????????????????????????????????????????????????????????????????????????????????????????????????????????????????????????????????????????????????????????????????????????????????QLSSRAKHLLHCCLCVGLILIVEVFTFSYDEVDPVRQYGWPWTIFLYLMRLLTVLALPQCICNCLGLLLYNAFPEKVRLKGSPLLAPFVCIRVVTRGDYADLVRGNVARNIETCADVGLENFIVEVVTDKALGLAKHPRIREVVVPTTYRPKSGALFKARALQYCLEDDVNILSDDDWIVHLDEETLLTEDSLRGILNFAFDGRHAFGQGLITYANERVVNWVTTLADSFRVADDMGKLRFQFWAFHRPLFGWKGSYVVARAGAERRVSFDHGLDGSVAEDCFFSMVAFREGYTFDFIPGEMWEKSPFSFWDFLQQRKRWMQGIFLVVHSGAIPIRHKFLLSLALYSWATIPLSTSNLVLAALWPIPCPTPLNFLCAFVGAMNIYMYVFGVIKSFSLYRLGPVRFLLCLLGAVSTIPFNVVIENIAVLWGCFGHKYRFYVVAKQLVMATGTLGCESSAKLQCPTCIKLGIKGSFFCSQACFKGSWDAHKQVHKDIKAN-FNPWPSYTFTGKLRPFPLSPKREVPEHIMRPDYAEHPDGIPLSEQAAKHSSVIKVLTEEEIEGVTLASKFSLEVLDVALGAAEVGVTTDELDRLVHEASIERDCYPSPLNYYKFPKSCCTSVNEVICHGIPDMRPLQDGDLLNVDITVYHNGFHGDLNETVFIGNVDKTARKLVEVTHECLSKAIEAVMPGVRYREIGNIIQKHAQSHGFSVVRSYCGHGIHRLFHTAPSVPHYAKNKAIGIMKAGHCFTIEPMISEGTWHDAVWPDSWTAVTADGKRSAQFEQTLLVTDTGCEILTRRRNKNGQPWFMDNNSVRQSLKERSQKRRALLAQQLGAGTASNLSQILGNEREAGPDDAARSPLEEVYTYRDSSTFLKGTQSANPHNDYCQHFVDTGQRPQNFIRDVGLADRFEEYPKLKELIRLKDELIRETAAPPMYLKCDLETFDFRTVKSKFDVILVEPPLEEYQRTCGLAHTKFWSWEEIMRLEIEEVAASRSFLFLWCGSSDGLDLGRQCLRKWGFRRCEDICWIKTNAKNVHLKNLEPRAVFQRTKEHCLMGIKGTVRRSTDGDFIHANVDIDLIISEEPQFGGTEKPEEIMHIVEHFCLGRRRLHVFGRDVSIRPGWLTIGPELTNSNFCSDAYNAHFNGTNDYLTGCTERIEALRPKSPPPKSATPGGARGARGR???????????????????????????????????????????????????????????????????????????????????????????????????????????????????????????????????????????????????????????????????????????????????????????????????????????????????????????????????????????????????????????????????????????????????????????????????????????????????????????????????????????????????????????????????????????????????????????????????????????????????????????????MKTFGDKPTAFQLEEDGEFYYIGSEVGNYLRMFRGSLYKKYPSLWRRLVTVDERKKIASLGLGPHSLATSISLLRAVEVDEIFDGKDDKYRAVSVSAEPPVQRETKSKRSTWMPTLPNSSHHLDAVPCSTPVNRNRIAHKKIRTFPLLYDDLDPATLHENSAQPEVLVPIRLDMEIEGHKLRDTFTWNKNESQITPEQFAEILCDDLDLPPLSFVPAISQSIRTQIEAFPTDNLLDDQTDQRVIIKLNIHVGNISLVDQFEWDMSEKENSPEQFALKLCSELGLGGEFVTAIAYSIRGQLSWHQRTYAFSEAPLPTVELPFRSQAEADQWCPFLETLTDAEMEKKIRDQDRNTRRMRRLANTTWFGANGSLLEKEIGFPSNEHYFGLVNFGNTCYCNSVLQALYFCKPFREKVLEYKARNKRTKETLLTCLADLFHSIANQKKKTGTIAPKKFIARLRKENDLFDNYMQQDAHEFLNYLLNTIADILQGAQGWVHDIFQGTLTNETRCLNCETVSSKDEDFLDLSVDVSQNTSITHCLRGFSNTETLCSEHKYYCENCCSKQEAQKRMRVKKLPMILALHLKRFKYTEQQNRHTKLSYRVVFPLELRLFNTSDDAFNPDRMYDLVAVVIHCGSGPNRGHYISIVKSHGLWLLFDDDIVDKIDAAAIEDFYGLTSDTPKTSESGYILFYQSREMLDVDKWIEIAKECKYLPENDLKKLCSMVCRLLLEECNIQPVSTPVTVCGDIHGQFYDLEELFRTGGQVPETNYIFLGDFVDRGYYSLETFTRLLTLKAKWPDRITLLRGNHESRQITQVYGFYDECQTKYGNANAWKYCCKVFDLLTVAAIIDEQVLCVHGGLSPQIKTLDQVRTIERNQEIPHKGAFCDIL-CPGTALHTYRIPPRGAGWLFGAKVTHEFMHLNNLKLICRAHQLVHEGYKYMFDDKLVTVWSAPNYCYRCGNIAAVLAFTDVNTRTAKLFHAVPDTERVIPPRNTPYFLMKSADGELESDPDTLTIEDIKEHARHIEKAVNSKEPRFILRILRALMPTRKRLNAKVMRKIVSGFYTHSNQSRDALLAFLEPMETEKEGRAKTSHLPLLPELDVYIHLLVLVHLIDSERLDQAVKCSDQLMAKIQAQNRRTLDLLASKCYFYHSRCYELTGQMSAIRSFLHGRLRTATLRSDYEGQAVLVNCLLRNYLHYNLYEQASKLVSKSAYPEAASNNEWARYLYYLGRIRAIQLDYSEARRHLLQAVRKAPQHAALGFKQTVHKLAITVDLLLGDIPDRSIFRQPPLRRTLAPYFQLTQAVRAGNLGRFNEVLENFGPKFQADHTFTLIIRLRHNVIKTGVRMINLSYLRISLADVAQKLQLDSPEDAEFIVAKAIRDGVIEASIDHDQGYMQSKENMDIYCTREPQAAFHQRICFCLDIHNQSVKAMRFPPKSYNKDLESAEERREREQQDLEYAKEEEDDGFLILTKILVAMASFVIHVIWSTAIPDLMSSIHPSVMLLLELLTVIPEELQARGPSRESVLQFLSRLVTL--SPSVLHCYHLQLLPRLV-QVENPEAAETLTNVATHPDARFPGFVMEVVSRIVGLEKTLAESLARGDRESIYSLLIEVGECHSHLLVESLLSKPEHKVLKLFRLVLQCSASPGHFPVDESWSRQALGFWYAVQDDVGAWEG-PQGESALHSLWQALLEFLRKARLPLDDTTTDEEKDSLRCYRQDISD--ALMYCYNMLRESLLATLAAHMELAKEWPFAEACVFALQAVAECVGVEQERYVAPLLAEALPALQ-HPRVVPTALSCLGAFGSSCLQATLPMLLRGLEDTAPAATLALKDISRDCSGALGPHGPHILAASQHLGNREKVRVMGLVGHILSSV-GDNARPWLSALVGPQLEALRLARPVFQTVVPLLN---------EQLVMTALCECVRRGAANL-EG---VKLLAVLVTLQLCSARAPNGKVHTVILEAFFQMYSIARKMDLFPVFACAPERSTVKALAEFILHSRPIPLMTVIELLVEQVLRATGSPRSVLDPMADILLALTKKYHWATVLIRTFEKKEHYLRLLLKERTNKRRIKEIV--SELSLKVKVKWKETFSDVEVDTPMVFKAQLFALTGVQPDRQKVMVKGAVLKDNDWGAVVLLMGSKEALPSPAEKPVFMEDMSESELATALDLPSGLTNLGNTCYMNATVQCLRTVPELKELAKFQGSVTAALRDLYGVMDSAVVPPIVMLQVLHMAFPRFSEKSEHGQQDANECWTEMVRMLQNFVDQYFGGIFVTLKCIESEDETTEHFLQLSCFIS-QDVKYMYAGLKSRL-LETIRKMSPTLGRDAEYKMSTISRLPAYLTIQFVRFFYKEKGAVNAKILKDIKFTMQLDMFELCSEALQQKLIPMRTKFKEEEDRFSGRDEGSNNSGYYELQAVLTHKGRSTSTGHYVAWIRREWFKCDDDKVVSADEILKLSGGGDWHTAYVLLYGPRLLEALINKKLPKELLLRIFSYLDVVSLCSCAQVSRLWHELALDGSNWQKIDLFDFQTDIEGPVVENISRRCGGFLKKLSLRGCQSVEDASLKTFAQNCNNIEDLLNGCKKLTDSTCQSLGRVLDLGSCCQVTDLSLRAIGQGCPNLEHLNISWCDQVSKYGVEALAQGCGRLRAFISKGCVNDEAVSQLANGLQTLNLHECTHITDAAVQCVSQHCPKLHFLCVSNCAQLTDASLVSLSQGCLCTLEVAGCTQLTDSGFQALSRSCHALEKMDLEECVLITDSTLLHLANGCPRLQQLSLSHCELVTDEGIRHLGAGVLELDNCPLITDASLEHLVPCLQRIELYDCQLITRAGIRKLRLDLKVHAYFAPVTPRPRYCRCCVVLTSGNARVFCPDHPDAALVEDYRAGDMICPQCGLVVGDRIVDVGTEWRVFPNEKSNNDPARVFLFKNPLLNGSDLATMIGKTGDASDESGTAKYQNRKTMSSSDRALVNAFREISNMADRINLTKTIVDRSNLLFKQVHDGKTLKGRSNDAIASACLYIACRQEGVPRTFKEICAVSKVSKKEIGRCFKLILKALETSVDLITTGDFMSRFCSNLGLPSSVQKAATHIARKAVELDVVSGRSPISVAAAAIYMASQASEDKKSQKEIGDIAGVADVTIRQSYKLMYPRAAELFPDDFKPKDCVPWVEKYRPAKFNEIVGNEETIARLEVFSREGNVPNVILSGPPGVGKTTTILCLARILLGPSFRDAVLELNASNDRGIDVVRNKIKMFAQKKVTLPPGKHKVIILDEADSMTEGAQQALRRTMEIYSKTTRFALACNTSDKIIEPIQSRCAVVRFSRLSDAQVLAKLLDICKQEDVSYAEDGLEALVFTAQGDMRQAINNLQSTFVGFSHVNSENVFKVCDEPHPLLIKDMLQHCVEGELDKAYKIMLHLWKLGYAAEDIISNVFRVCKSHTMPEYLKLEFIKEIGYTQMRTLQGVQSLLQMAGLLSRLCMKTKVIKYMKKEHSLVKPYQGAGMTIPNWDFLGNTMVTGQYIRLTPDRQSSKGAIWNNVPWEMHVHFKVHGSGKELFGDGFAIWYAKEALQLGPVFGSKDFSGLAIFFDTYANQNGPHNHGHPYISAMVNNGTLSYDHDRDGTHTELAGCEAKFRNLDHDTHIAVRYENDVLTVSTDIEGKNAWKECFTVKGVQLPTNYYFGASAVTGDLSDNHDIISMKLFEIDEDRSKIVPSAAPPRDHIEDAMSGTKLFLIVLCAILGLVLIVFQKQQETSRKRFYHVRDSFDEIRTRNCKLVDVNQLFLPNDTVTHVPNIKRLNIDPVFPNRTNLLHLHNMAISRAFFFSYILQKAADDEPGFMYYFMSVIADVAANRFLNSSAIYYAPNMSFTPSYKSFFNKTMPLFAPRAYRADDFNDPYHLEGTSTLNTIEAIDLGAISRNYSSDQYRINEWYHHWLPDLTKRQDSKTTYTVQITGTNETFTWHGPPAASDNPGPVKWTRPYFDCDRSNKWTYGATVPIPDIYPRHTQWRHIEIPKYVAVAVMEMDFERLDINQCPIGSGN-PRPNYFAGTSRCKNDTTECEPVNGYGFRRGGYQCRCRPGWRRPRIVRNPFHGELIERATDQEYKKGYNCEKIGYMMVQTQNLLNHMAIGAHFDPHLSGDVAYGRDQKLENEARSAVRLANFLSGFIQTVDPKELFAEFRVPDRSLTPDQVIAEALSLVIGNEKIQGVGVYFDHKQFFAPY--AYRKTRNALRFFVDDKAAESFRLPSKVVSNEVSPWVRVTPTSGHLFAETGTCGGMGKKAVKTIISWMFYFSSMSGRLYEHAIVERSKRKQKELKQVFYIKLTLKTDITSEACVPILGRGFSGGYKCECLQGYEYPYNDPITYFDGQIVEAPSKYDTL???????????????????????????????????????????????????????????????????????????????????????????????????????????????????????????????????????????????????????????????????????????????????????????????????????????????????????????????????????????????????????????????????????????????????????????????????????????????????????????????????????????????????????????MT--EAPNEFRLQQSPTDAISSLQFGPNSNQFLLVSSWDSNVRLYDVVSNTVRLKYSHKRPVLDCTFQDAVHAWSGGVDCQVKSFDFNCSAETVVGQHTAPVRCIEYCPDVNVVISGSWDSTVKLWDPRSPTNAGTFNQAERVYTMSVCGDKLVVGTALRKVLVWDLRNMGYVKQRRDSNLKYQTRCIRCFPNQQGFVLSSIEGRVAVEYLDPSAEVQKKKYAFKCHRTKDSAGIELIYPVNAIAFHNLYNTFATGGSDGHVNIWDGFNKKRLCQFHKYPSSIAALAFSHDGSLLAIGSSFLYEEDDVEVIPPDAIFVRRLVDGSRTMSSSLDQLKEITTVVADTGDFEVMKQYKPTDATTNPSLILAASKLPQYSHLVDEAVQYGKKPKEKLTNAMDKLVVLFGSEILKIIPGRVSTEVDARLSFNTEASIAKALRLIALYKEAGVDKKRILIKLASTWEGIQAAKVLESEHGIHCNMTLLFNFTQAVACAEAGATLISPFVGRILDWHVANTAIKSFQPLEDPGVLSVTRIYNYYKRFGHSTVVMGASFRNVGQVRALAGCDLLTISPALLSELAISIEPIVQYLSEDKAKKLDLEKVEVNEAIFRWELNEDQMATDKLSDGIRKFAADAVKLENILKEKLQMAYTIARPLVNVYNDKNELSGTHVALPAVFKAPIRPDIVNFVHVNMSKNNRQPYAVSKEAGHQTSAESWGTGRAVARIPRVRGGGTHRSGQGAFGNMCRGGRMFAPTKTYRRWHRRINVAQKRYAICSAVAATGVPGLVLSKGHKIEEIPEVPLVVSDKIQDLKKTKEAVLFLKKVKAWTDVEKVYKSRRLRAGKGKMRNRRRIQRLGPLVVFEADNGITRAFRNIPGVDTLRVDKLNLLKMAPGGHVGRFVIWTESAFRRLDKLYGTYRKPSSEKKNFSLPKPQMTNSDISRILKSDEIRQVIRAPCKKVVRRTQKKNPLKNINAMLRLNPYAAVTRRASVLLNQKQKLKKLLAEKRGVKLEKLQPARRAAAIKKKG????????????????????????????????????????????????????????????????????????????????????????????????????????????????????????????????????????????????????????????????????????????????????????????????????????????????????????????????????????????????????????????MFARAPKNLLNAVCISAHRNFSVTSKNHVKVAVLGASGGIGQPLSLLLKQHPAITYLSLYDIAHTPGVAADLSHINTRPQVKGFMGTDQLPESLKGMEIVVIPAGVPRKPGMTRDDLFNTNASIVRDLADACAQACPKAFLCIISNPVNSTVPIASEVFKKRGVYDPNRIFGVTSLDIVRANAFVAEAKGLDPATVNVPVVGGHSGVTIVPLLSQATPSVSFPQPELEALTKRIQEAGTEVVQAKAGAGSATLSMAFAGARFVFSLISALQGKGVVECAFVKSTETEATYFSTPLLLGKNGLAKNLGLGKLSPYESELVKTALPELKNNIKKGEDFSKKEFEWLLQEEVNIVLEQLVIMECSKRFPV-VKSEKFFM-QIKVVVTLTGDNISHADITLRI-PKHNLRTIVQNDCQWKLQQVQDAGNNLLQALSLLRFEFKSAEEVTQLMTTIMGCLQRGRASLIIPKKRTIEEILGSRNMKSLQPPLPSDIAASFYVQSHKLVFAVYHI-HKDSPKF-DAEASVPWLSEVLVLFTIALQLCQQLKDKVGVFQFRDFIPTFIDILKGFGADISNVQQIIGVCDVCFKDIEAVL--NSIVS-ILAAFCDKLSKAPSNRMCVR--VLQNLYEALRFDVYYNLVKVASDMMAAVFSDLNKVKAWLPVARVQKLLRLLHEALTELASKLMVELLSTYTEDNASQARDDAHRCIVSCLADPNTFLLDHLLPLKPVRFLEGELIHDLLTIFVSEKLSAYLQFYQANKDFV-SSLGLSHEQNLHKMRLLTLMQMAETQRELDFDVLQRELQL--QDVEGFVVEALRTRMLTAKINQTGRKVVVATTVHRTFGRHQWQQLKDILRLNLVENNMDRFLETRG-TK-RKKPIVELEEGKNAIEEMEERAMIEEDPRLKELISVLIEWINDELASHRII--VKDIEEDLYDGQVLQKLLESLTGKRLDVVEVTQSEEGQKQKLRTVVDCANQILGYKWSVESIHNKNIVSIIHLLVALARHFRAPV--RLPENVVVNVVVVQKR-EELTSTYDDLGMRVE-RDAFDTLFDHAPDKLQVVKKSLMTFVNKHLNKINLEDLD-TQFQDGIYLVLLMGLVEGYFVPLYFFPTP----------------------SSKVHNVAFAFSLMTDAGLKPKARPEDLVNADLKSTLRVLYNLFTKLSIN----PINFYTYANEQFLKI-----ELSHRSPGIVETAEADLRELMGIPSNYKVIFLQGGGTGQFSAVPLNLCPADYVVTGTWSAKAAKEAEAFIKHTCIPPQSEWKRSSDASYLYYCDNETIHGVEFPLVCDMSSNILTKPVDVSKYGLIFAGAQKNLGMAGVTVVIVREDLVGQAGCCPSVLSYKINAENKSLYNTPPTYAIYILLVMKWVKRNGGVEGMRRSAEKSRLIYSLFDASNNFYNRSRVNIPFRI-GGAEGDDELEKLFLKEATMIQLKGHRSVGGIRVSVFNAMSVEAIAVFYRDRPEWKDVTPVVRIAYSEQFQDIFDYFRAVLLNERSERALELVTDAVDINTSNYTVWHYRRALLKDLGKDLHEELTYIQKVIEDNPKNYQVWHHRRVLVEWLEKAFTESVLDAKNYHAWQHRQWAISEFDLWDGELDYVSTLLQDDVRNNSAWNQRFYIISNTTGFVLDREVAYTFECIRHNESPWNYLRGILVAYLLAFMVDRALEFDVIRKEYWNFVARGIRRNIELGLLASLLCTLLISFSIAVIVDRFDELLLFAPHMHKFLKAQRIQHRFLIVNQVDRLRFNRGSLINVGFLVAQSDCDYLVMHDVDLLPLNRELSYAYPANGGPHLAAPDLHPRYHYPTFVGGILLMSNARFRQLNGLSNKYWGWGLEDDEFYARMRDARLNVSRP-GLKTGIRNTFRHVHDKQHRPRDTARLHNQRAETRKRDRVTGLADVKYDLAALHRLVIDGAPVEVLDVRLRCNLTVTPWCQK--VAPGDVITTDGFMRGHG-LLSSVAGVVEKTNKLVTVRPLKTRYNGEVGDVVVGRIVEVQ--QRRWKIEMASRLNASLLLSSVNLPGGELRRKSAEDELLMRQYLSEGDLISAEVQSVFADGSLSLHTRSLKYGKLGQGTLVLVSPSLVKRCKHFHNLP-CGVHLILGNNGYIWIAGTKFVREAVARVRNCVLALARHHVMLFDTSVVYAYDVS-VADLTKQKSQKVVGGWLLVCSGMAGSNLNDAFAFRLTKSGLSMVHWHPFAEFPPSGKQWEQEFLKYQEYPEYKEYNQAMTLSEFKRIWYIEYLHRMWGRTIGAVFYTGASWLWWRGWLSRRAKGHVAVLGVGLAFQGALGWFMVRSGLHDQPLVGHMQKGYAIGQAFLLFTRILQTSEFIILKQTFSPRMSRVLGLVFVTAMSGALVAGLQAGLVYNSFPKMADRWVPSDILALPKLRNFTENPTTVQFDHRILGESVVTGLWLWGRKQPLPPRARKALHSHSGANMRATLGVSTLLTYVPVSLASSHQAGAVTLLSVALWLAHELKLLRR-ICCR-----------------------TEKNASQWSKDKLTELLTNLEVKD-GRGSCVVEMSKCDGEAVANNRKAKLIFFYEWAIELTDDS-TVEGKVEIPNLSEEHDPTDVDITVTV--ERLKELMRTKGEKLIRDQLETYISSLKKEFSQGMILPTKTDSINQTKSTGKALDTTTLTHETFKCTAQELYRALTMVQAFSQGPCGGRFELYGGNVTGTFTDLELISMRWRFSSWPQGHFSAVLELVQKEDCTLVQEHVPKAEADRTRDGWQRHYWDSLKRTFGFGAILMDPEMFLDVANQVTKLKMYPYFDIAHCVITCLYLREDLGPGSQLFSRKHPLSCWITSMFSIFAGGMLSSFLLGEPVLGIFKNNQQLLLATAVWYAIFYSPFDLVYKICKFFPCKLIIALMKEVTRCKKVHDGVTHAAKIYPNGYLIMVVIGVVKGNGSAFLKICERLLRGYWTPNAMEIMQPSFPTKACIVAAVIFVLDKKTEFISAPHALVYLGIVVFLLYFKLSAMLLGIHDPFLPFENLFCAIFLGGIWDALGRALM---SGRGGDGKSDSSKNGKSDATKKKDMLRKEGLVQRKNASSNDKSKDVDDSPRQESEDDSKETRLTLME----------EGYTSFWNDCISSGLRGCILVELALRGRIELERCGVRRRSLLLRKVLLRSDTPTGDVLLDEALRHLKETRPPETLQAWIDYLSGETWNPLKLRYQLRNVRERLAKNLVEKGVLTTEKQNFLLFDMTTHPLVDQASKGKLVRRVQEAVLGRWVNDPQRMDRRLLALLVLAHASDVLENAFAPLSDEDYELSMRRVRDLLDLDMEAEAAKPNACEVLWGVFAAFVKMQDCTPTFKCVLVGDGGTGKTTFVKRHLTGEFEKKYVATLGVEVHPLLFHTNRGPIRFNVWDTAGQEKFGGLRDGYYIQGQCAIVMFDVTSRVTYKNVPNWHRDLVRVCENIPIVLCGNKVDIKDRKVKAKSIVFHRKKNLQYYDISAKSNYNFEKPFLWLARKLIGDPNLEFVAMPALAPPEVTMDPEWQAKLENDMKEAQNTSLPDEDDDDLFADRLTPILKESKFKETGVITPEEFVLAGDHLVHHCPTWQWATG-EKSYLPPDKQFLVTRNVPCYKRCKHMEEKVLEEEDGGWVDTHDDDDDDEEAADMDDFVDADDPATGIVSTRTYDLNITYDNYYRTPRLWLYGYDENRQPLTIEEMYEDISQDHAKKTVTMEAHPHLPGPPMASVHPCRHAEVMKRIIQTVTEGGGLGVHMYLIVFLKFVQAVIPTIEYDYTQNFAMISKFEPNLPYDKLQQNLQVVRKRLNRPLTLSEKILYSHLDQPETQEIERGTSYLRLRPDRVAMQDATAQMAMLQFISSGLKRVAVPSTIHCDHLIEAQVGGAKDLARAKDLNKEVYNFLSTAGSKYGVGFWKPGSGIIHQIILENYAFPVLFFVSFTCSVQNKSGLGGLCIGVGGADAVDVMADIPWELKCPNVIGVNLTGKMSGWTSAKDVILKVAGILTVKGGTGAIVEYFGPGVESISCTGMGTICNMGAEIGATTSVFPYNSRMKDYLAATNRKDIAEAADQNKELLTPDSGCKYDQVIDINLSELEPHVNGPFTPDLAHPISQLGKNAKEAGWPMDIRVGLIGSCTNSSYEDMSRAAFLAKQALDHGVKAKCTFTVTPGSEQIRATIERDGQSKIFDEFGGMVLANACGPCIGQWDRKDIKKGDKNTIVTSYNRNFTSRNDGNPATHAFVTSPELVTALAIAGRLDFNPMTDELTGSKGEKFKLESPRGDELPQRGFDPGEDTYQGPPKDGTGVSVDVDVKSQRLQLLAPFDKWDGKDLEDMVILIKVKGKCTTDHISAAGPWLKYRGHLDNISNNMFIGAISEESGEANKVQHRQTGEWGGVPDTARKYKASSLRIVASGNEAFDEGPQTLHSRCERERRGPCA----------ETNLKKQGLLPLTFQNPADYDKIKSDDKLSLLGLKDLAPG------MFKNTFQSGFLSILYSIGSKPLQIWDKKVRNGHIKRITDNDIQSFVLEILGTNVSTTFITCPADPRKTLGIRLPYLIMIVKNMKKYFTFEVQILDDKNVRRRFRASNFQSTTRVKPFICTMPMRLDEGWNQIQFNLSDFTRRAYGTNYVETLRVQIHANCRIRRVYFADRLYSEDELPAEFKLYLPVQAKAKV---------------SLLT--KYHYPDQAKKDVSNALQHYRNLSSQFVFNDGTKKELFCLDGTIPVSYKYNIPVCVWLLDTHPYNSPMCYVKPTAYMQIKVSRHVDQTGRVFLPYLHEWNPNS-SDLLGLIQVMIIVFGETPPVFSK-PQPYPT-NTGTITQEHIRASLLTAVEDKVKGRLKEVAQAEMDVLKKTHDELNAGKTRLEDMINRMDREAELESERNEEMKEVDVDEAVVTTAPLYKQLVNAFAEENATEDAIYYLGEALRKGVIDLDVFLKHVRELSRKQFMLRALMQKCREKAALPSGRKFCVGGNWKMNGNKSSIKEICDMLKTAKPNTEVVLGCPAPYLDYVRRILPAAIAVSAQNCYKVEKGAFTGEISPAMIKDCGATWVILGHSERRNVFKESDELIGDKVHHALESGLNVIACIGELLEEREAGKTEEVVYRQTAAIAAKVTDWNRVVLAYEPVWAIGTGKTASPEQA-EVHAQLRQWLSKNVSPDVAKKVRIQYGGSVTAANCQELAKKPDVDGFLVGGASLKPEFVEIINARQ???????????????????????????????????????????????????????????????????????????????????????????????????????????????????????????????????????????????????????????????????????????????????????????????????????????????????????????????????????SRDAVHSGGAMPPTSFGTSASKIDYLRQWSVSTYKCTRQMLSEKLGKGTRTVDTELEAQIELLRDTQAKYLHVLRLARALASHFQVVQTQGLLAEAFQELAHR-------PLQEEFRYNAEAQRSLGRNGETLLGALNFFVSSLATLCHKTIDDTLLTVRLYESARLEYDAYRTEAQRNFKDKYEKLKADVTIKMKFLEENKVKVMHKQLLLFHNAVSAY-FSGNQASLEATLKQFNISWLEQ-MAELLLDPDIRIWVFLPIVVITFLIGIVRHYVSILISSTKKVELQQVQDSQALIRSRFLRENGKYIPKQSFLMRKNFFNHEETGYFK-TPKRAPVMQNPMTDPSMMTDMLKGNLTNVLPMIVIGGWINWTFSGFVTTKVPFPLTLRFKPMLQRGIELMSLDASWVSSASWYFLNVFGLRSIYALVLGENNAADQTRVMQDQMSGAALAMPPDPKQAFKNEWEALEICEHQLVS-----------------------?????????????????????????????????????????????????????????????????????????????????????????????????????????????????????????????????????????????????????????????????????????????????????????????????????????????????????????????????????????????????????????????????????????????????????????????????????????????????????????????????????????????????????????????????????????????????????????????????????????????????????????????????????????????????????????????????????????????????????????????????????????????????????????????????????????????????????????????????????????????????????????????????????????????????????????????????????????VKLTAEQLWKGVTSVSNAGRKRGRASGHSRKLSKDLNRGQVIGVGKVNMVWPGLNAPVVRGKEVVERVQLPPDKQREEKLLQLRNEMHGFRPLKLTPLERGWCGTRAPGRSIGPPDPVGDETFEKFDTIVVESRMVSRMTGNLGRTRRHKAVVVVGNKNGLIGFASGKAPEAKAALRMAKNRAIQRLRYIDRFEDTVYHNFFTQYGPMKMMVRKKPRGYGLVCHRIFKEVCKLAGITDIHVKMDCRAGSATVLLRAFILGLHNQKTHQQLADEKRLHLVEFKRETDNFPRVVASPKNCRTAEQIGTDEELDFDLVVSGGRLVKPPPKYVPWYIRTKGWQVHLRKVDPYRNAYNTRLQLTARYGKLCSFVNVSSPKDKDASRKSDEKTKWINKQRVMIFASRGITFRDRHLMLNLRTMLPHSKPESKME--KKDINEICEMKNCNKCIYFENKKRKDLYLWMSNVPNGPSVKFLVENVHTMEELKMTGNCLKGSRPLLSFDKAFNENPLLKELLCQVFGTPRHHPKSQPFVDHVFTFSLLDHRIWFRNYQIVEE-EGSLKEDSPRFVLNPIKVFEGSFGGPVIYSNPHYVTPSAYRRTLKQAAASKYKIDEIFETKLLFQRMAVGKNKGLSKGGKKGVKKKIVDPFTRKDWYDVKAPTMYTVRNIGKTFVNRTQGTKIASEGLKGRVFEVSQADLTNGEDAYRKFRLIAEEVQGRNVLTNFHGMDLTTDKLRSMVKKWQTLIEATVDVRTTDGYLLRMFCIGFTKKCANQLKKTCYAQHNQVRLIRKKMTEMMVVEVSSSNLKDVVNKLIPGSIGKDIEKSCQHIYPLHDVLIRKVKVLKKPKFELGKLLELHGEGKGAASTEGVKVDRPEGYEPPVLETVIKVGIIGGSGLD-DPDILEHTTPFGEPSDV-LVSGQIGHVDCVLLARHGRNHGIMPTNVNYRANIWALR-KMGCTHVLATTACGSLHEDFAPGHVFPDQFIDRQRATT--VCHLSMASPFLRKLLIQHEKGTVVTIEGPRFSSAAESIMFRSWGCHVINMTT---VVLAKEAGLLYAAIALPTDYDSWR-TDLAHVDVKQVETMKENSERALRILRAAIPRISRISLV--------------------------MKVLRHEEFEEGCRAACNGPYDLNWSKTMVGYGQEDNHFVVELTYNYGIGSYERGNDFLGIVIRSGDIVERARRHSWPVQEKGDVVVVMAPGGYPFLVCPKRNPLRQDPVERVILSSSDLARTTAYWRDILAMTLVHSSEKSAVLAYANDQCHLEFRYTAEPIRRGTAYGRIAFACPAAELQVIENRVKRAGHRILTPLTRLDTPGKATVTVVILADPDGHEICFVDAESFMLLSAVDPNANQLLSRAIEKDKSDEWFRDMAGGKKPS??????????????????????????????????????????????????????????????????????????????????????????????????????????????????????????????????????????????????????????????????????????????????????????????????????????????????????????????????????????????????????????????????????????????????????LRSQRSLTSLRQHTPR-IHTSSRRLNPLIPIVIEQTGRGERAYDIYSRLLKERIVCVMGPITDELSSLVVAQLLFLQSESNKKPVHMYINSPGGSVTAGLGIYDTMQYIMPPISTWCIGQACSAGSLLLCAGTAGMRHSLPHSRIMIHQPSGQASGQATDIQIHAEEILHLKRVINGMYAKHTKQPLETIESAMERDRFMSAEQAKEFGIIDVVLEHPPTF-EVPELMRADKPIGTWLLMWPAWSISLASQAGCLPDLALFGAGAFLMRGAGCTINDMWDRNIDSKVERTKYRPLAADQLSMFDALVFTGGQGLSTLILLQLNWYSIVLGASSVILVTLYPAMKRITYWPQLTLGLTINWGALLGWAAVVLPLYAASLCWTLIYDTIYAHQAKMDIGYNKLMSTALKFGPRTKRFLSLFSTMVSNLQCWPYYAAVTLKLNQIGFIMGILSSSLVRIPGVVKPRTQRGKRFLQNRESKLVENTKTIFIRGSNANSNVIKAMKNMCSVKRPHSVFFNKKNPFEDQTPIEFMKKSDASHFVFGSHSKKRPNNLVFGRTFNGHLLDMFELGMDSFKSLEDFKGPKVPVGTKPMLVFAGEQFEELERLKNYFIDFFKGEAVSLQGLEHVIMFTAVEVLLRSYRVLMKKSGTKLPR-VELEEMGPHLDFSLRRTKIASDDLFKTARRQPKQNKVTKKKNVEKTALGSTLGRIHMERQDFQLQTRKLKGLKIKVLNALQDNYMYLVVTKEAAVVDP-----VLEEVKVKLTTVLTTHHHWDHSGGNDKLVVYGGDDRVPKITRDGE-QLQVGQLVKCLHTPCHTSGHICYFFVFTGDTMFIAGCGKFFEGTADQMYKALLAKLPDATRVFCGHEYTINNLKFAAKVEPGNQIVDKMAWAKNEPTVPSTIAEEKTFNPFMRVAVHKHADPISTMAYLRKEKDHFRMRRSRRVPRAKEPPPNLTDCIAGVDGRAESVDKKISRLDAELKKYKDQMVKMRDGPAKNMVKQKAMRVLKQRKMYEQQRDNLMQQSFNMEQANFATQTLKDTKTTMEAMRLGVKEMKREYKKVNIDDIENLQDDLEDMLDQAGEVQEALGRSYGMPEVDDDELEAELNALGDEIALDDTSYLDEVTAPTIPTKEPGADSVTQGGVLVDEFGVLRRPPITSTRASSRAKHTLPDLPYDYGALEPVISGDLMRVHHQKHHAAYVNNLNAAEEKLADALAKNCVRSIVSCSMAIKFNGGGHLNHSIYWTNLSPNGGGEPTGDLLEAIKKDFGSFEALKAQMSARAVGVQGSGWAWLGYNPKTQRLQVATTGNQDSLWDTEGLVPLFTIDVWEHAYYLQYRNVRPDYVKAIWDIANWNNVAERFQKAHDVHIQDEFIKEALQSGMDLRQYSLQVEKDLKEVENASIQDSIEESVNIASLHNQIAACDEILERMENMLKTFQEDLGSISHEILTLQQQSVSMNVRLKNRQAVRGELSQFVDDIIVPEAMINNILEMPVTEKEFLEQLYVLDHKISFVKEQSFKDSRSCQDVRDILEKLKFKAIAKIREYLLQKVYSFRKPTTNYQVPQNAMLKHKFCYQFLMTHEREIAREIRDEYVDTMGKILFSYFKSYTNRLMKLQFEEVAEKDDLMGVEDTTKRNILSKPSLKNRSTVFTVGTRNNILTTELESPIIVPHAAQKNDARYPFESLFRSQQYALLDNACREYLFVTEFFMCSGSAAQDLFNLIMSKTLTMFLVRLTLQFAGCYNSLYLFVYIAIQYYCIMFLCSPTQSALTLYWESLLQFLYPRFEYILRLNIQSIRDCDPQKLGSIDMRPHYITRRYAEFSAAVVSINENFPSERVASLLAALQGEVENFILKMAAEFQGRKEQLIFLINNYDMMLSVLLERTKEDSKETESFKELLNARTQEYVEEILAPHFGGMISFVKECELLQERGQADVMQREEKKVTMLVRSFNTGWKKAIDDINHDIMQTFTNFKNGTNILQAALMQLIQYYHRFQKVLSQAPFKQLTVRTELLNIHHLMVEVKKYKPNF???????????????????????????????????????????????????????????????????????????????????????????????????????????????????????????????????????????????????????????????????????????????????????????????????????????MRLTNVLRLPKDYANLPESYVKRSMAMVEWRTPNAPQYQRKVIQRTYNMSRPWTDGFKSNMPGVRIYVEPIFRGDRVEVLVGRDKGKQGIVNYIVKERNWVVVEGLNCREAPLLVTTQVALLDPTDNKPTKVEWRYTEEGKKVRVSVRTGRILPIPLAEETMDYKYFEQPKDTKAKDLEKITYVP-KLMTFAQDIMEEMGIKEDRIPAKTYWYMGDE----VINHLSIHKIESLLWFTRMCTVVFTLLYIIPNPYTCYQRALISNAATSALRLHQRMPTIQLSRMFLSQLLIEDSCHYLFFSLIF-LPMT-LVLLPVFLFALLHSLTLLDKFGLLLE---HSNNILRLVAFTEIFLMPLTVFSLLSGSSLVTPFVYYRFLSLRYASRRSPYSRTVFHELRLVTEHYAAPGCPAFLSRIGLICQLSPFILAMILVYLNFPKLEPSEKQHIKLPRDIEDAKGLGRVLNRYTDRAVTSGFFVTYILLQSFAIPGSIFLSILSGFLFPFPMALFLVCLCSALGASFCYFFSYLVGRRLVLKYFPHLLNYMIFLRITPFLPNWFINIAAPVIDVPIAPFFLGTFVGVAPPSFVAIQAGTTLHQLSSSVSWWSVSVLAAFALLSLLPVVFKLKQKVMSSQEKLTRIAIVNTDKCKPKRCKQECKKSCPVVRLGKLCIEVTPNDKIAAISENLCIGCGICVKKCPFEAISIINLPSNLEKDTTHRYSQNSFKLHRLPTPRPGEVLGLVGTNGIGKSTALKILAGKLKPNLGRYGDPPDWTEILQYFRGSELQNYFTKILEDDLKAIIKPQYVDQIPKAVKGSVQQLMDKKDEMQNQEEVCRVLDLNNVRDRSVGDLSGGELQRFACAMVCIQKADIFMFDEPSSYLDVKQRLKCAEAIRALIHPQKYIIVVEHDLSVLDYLSDFICCLYGVPGCYGVVTMPFSVREGINIFLDGFVPTENLRFRDTSLVFKVAETAEEEVKRMCRYEYPNMRKCLGDFEMSIDAGTFTDSEIIVMLGENGTGKTTFIRMMAGLIMPALSGDVPSLNISYKPQKISPKSQGTVRMLLHEKIRDAYVHPQFIADVMKPLQIDAIIDQEVQNLSGGELQRVALALCLGKPADVYLIDEPSAYLDSEQRLVAAKVIKRFILHAKKTGFVVEHDFIMATYLSDRVIVFEGQPSVKTRANTPQTLLAGMNKFLELLNITFRRDPNNFRPRINKLNSVKDVDQKRSGNFFFLEDEEDGEELFGPALDTYDAVEDQLRQRDRGRMRRGLESIENLEDLKGHTVREWVTQGPKTEIYNRFKNFLRTYKEKIRQMEENKHSLEVNYTLAAAEQVLAYFLPEAPTEMLVILDEAAKDIVLGMFPQYERITGEIHVRITDLPLIEEIRSLRQLHLNQLVRTAGVVTSTTGVLPQLSLVKYDCGKCNYVLGPFVQSQELRPGSCPECQSLGPFTVNMEQTVYQNYQRISIQESPGKVTAGRLPRSKDAILLGDLCDSCKPGDEIELTGVYTNNYDGSLNTVNGFPVFATVVMANHVVKKDTDDDTRHILALAKDDIADRIIASIGPSIFGHENIKRAIALSLFGGEQKNPGQKHRVRGDINLLICGDPGTAKSQFLKYVQQVAPRAVFATGQGASAVGLTAYVSPVTKEWTLEAGALVLADKGVCLIDEFDKMNDADRTSIHEAMEQQSISISKAGIVTSLQARCAVIAAANPIGGRYDTFSENVRQSFFALSEAAAVFFLKDERLARFVVESHMRHHPIPQELLRKYILYAREKVHPKLDQDKIARMYSELRRESMATGSVPITVRHIESMIRLAEAHARLHLRQHVDVNMAIRVMLESFITQKYSVMRMGLLADCERIFGSTDIYKLLDVPKDASPTAIKKAYRRLSLLMHPDRVNAVQKQAATQKFQVLSKVYVLLSDSEKRAVYDETGCVDDDEDLSNNKDWDAYWRLVFPNITVADIDRFMLTYKGSPEEIEDLKKRYEEHEGDFDAISECLMGYEEDRYREILNDLIAKEEIEAYPKFTKEPKKKRNARRDRFLAEAKEVAD------GAMEDLAMALSKRQRASFNSLIDNIEARYCK----KKKKVTK-DLVMLLTIVLIALGKIGAKKRKKLEMKAEKRLQRERDLEDREEMKQRQAEEARKQLDEQKRKEEEAERAAEEERLKKEQEQRELEEYLRMKEAFSVEGEGFDAATQEQASPSFLHEFIEYIKTEKVVILEDLAAKFQLKTQDCIDRVQALLVDETLVGVIDDRGKFIYITRKELEDVAHFIRQRGRVSISELFENSNSLINLTPDVTALEAEAKATAIKHIITMLQLPEQLDKVQQYKRRVSRKKASVEAMLKTALQSQLDGFRTALTLQKSIPNDVLEVQRNDMQEIFDLPGLERLQEVKEESMRHSQYGAAIENLKHIFNVPGSVQKTQELIASGKLLHAHQLSDLENSRDDLLFELHKLPSQSVTDRNMLKQYFADVEKLSEDLGKQIWLVLKRTLNSVRKEPQVIVTALRIIEREERAARERERSSAGFLPPGRPKEWRRRCFEVLESAVEDRIEGNQFEERHENKMWLVRHLEVTRQIVLDDLRTVKTVCVPCFPPEYDIVERVKMYHNCLSRHLQNIVAQLEGNEYITVLGWLTVYSGEELMGHPDLALDVPLLGRDVQGLIQKYLGTLVANYQDWLKNALQDVKDWPDTDSRGCYHTSAPMIVFQMVDQHLQVAKTVDLVRKVLTISLEQTTKFVGAYMEAVTEFHFEDRSFTHYVIAVANNCLQFS-LDRLQERTLDALRDELFLDLGKLGDVLSDTVSATIEDYFRDYIQWQLARCYVQAIL--QRKITFKGYEERKVAAEKEQLRASPLDALPLLAEVLKMKDTSLLSLEVSGRRNLFISICLVGLSYAQETTA-DGTVKLDNDLGSSRDGSRTDDEVVQREEEAIKLDGLNVAQMKELREKAEKHVFQAEVSRMMKLIINSLYRNKEIFLRELISNASDALDKIRLLSLTDPDVLNTNPELTIRIKSDKDNGLLHITDSGIGMTKADLVNNLGTIAKSGTAEFLQKVTESDAPKELNDLIGQFGVGFYSAFLVADRVVVTSKHNDD-KQHVWESDSGEFTVAEDPRGNTLGRGTTVTLQLKEEARDFLEQDTLKKLIEKYSQFINFNIFLWTSKTETVEEPIEEP--TEEKEADTDKEEEDKVEEEEDDKKPKTKKVDKTTWDWELINSAKPIWTRKPAEIEEKEYEEFYKAITKDTQPPLMKTHFIAEGELTFKALLYVPAVQPTESFNRYGGKVDHIKLYVRRVFITDDFQDMMPSYLSFVRGVVDSDDLPLNVSREMLQQHKLLKVIKKKLVRKALDMMKRIPKEDYQRFWKEYSTNLKLGIIEDTTNRSRLAKLVRFHSSH-G-EELTSLSDYVTRMKDGQQFIYYIAGASLDEVKRSPFVERLIRKGYEVLLLTEPVDEYSISSLTEFEGKKFQNVAKEGLKVDE-GKARERHDALVKEFEPLTKWLEDDVFKGRILKAMVSERLATSPCALVANQFGWTGNMERLARSNAHAKSQDTMRDYYLSQKKNMELNPRHPLIKELLRRVKDDAKDSEARNMAELVYETATLRSGFMLEDTLAFATRVESLLRKNVGLPDDAAVEEEPEEEFATEASMAADEDEGETVERQQLMVKGE--RLSKEEVQMRVISEIIKELLVAQKEKRDVNLNSIKGSISSKYGLHSQPKIVDIIAAIPAQYKEVLLPKLKAKPVRTASGIAVVAVMCKPHRCPHINMTGNICVYCPGGPDSDFEYSTQSYTGYEPTSMRAIRARYDPFLQTRHRIQQLRQLGHSVDKVEFIVMGGTFMSLPEDYRDWFVRSLHDALSGHTSGSVGEAVLYGERGRTKCVGITIETRPDYCLKRHLGDMLLYGCTRLEIGVQSVHEDVARDTNRGHTVRAVCESFRLAKDAGFKVVAHMMPNLPNVDFERDIDQFVELFENPAFRMDGLKVYPTLVIRGTGLYELWKTGRYRSYPPGFLVDLIAKILALVPPWTRIYRVQRDIPMPLVTSGVEHGNLRELALARMRDFGTRCRDVRTREVGIQEIHHRVRPNQVELIRRDYVANGGWETFLSYEDPEQDILVGLLRLRRCSEETFRPELKGGASLVREHVYGSVVPVSVRDPTKFQHQGFGTLLMEEAERIALTEHGSSKIAVISGVGTRNYYRKLGYELDGPYMSK

>Galendromus_occidentalis

PGARVNVLSKELMLSVVLISGKKGCFIAGADITMLESCKSEAKKLKPVVAAIMGSCLGGGLETALACHYR-IAMNDSKTALGLPEVMLGVLPGGGGTQRLPKLVQLPTALDMMLTGKSLKANKAKKAGFVDVVIEPLGPGVYLEEVAARDLASQKLKVRTRPLSEKVVRDY-IFDKAKGQVMKLTNGLYPAPLRIIQVIRAGLEKQNFAELCTTESRGLMGLYHGQVQCKKTVIPCLSRNVAVLGAGLMGAGICEVSLKNFDKVIMKDGLVRGQNQIKRKRLEKDKLMSGLLPTLDYSDMVIEAVFEDLAVKHKVVKEVEAEHCVFASNTSALPITKIAEASKRPEKVVGMHYFSPVDKMQLLEVITTDKTSKDTAAAAVDVGLRQGKVVIVVKDGPGFYTTRILAPMMSEAMILLMEGCPVRDLDKLKDFGFPVGGATLLDEVGIDVGAHIAKTVFGEREEMVSNFLGRKSGKGCYIYRAVNPMQAKYTTEQMQYRLATRFINEAIMCLQEGILANPVEGDIGAVFGLGFPPNRGGPFHFVDTYGADKIVNMQQFQEFEPCQLLLDHA-KDPSKKFHMQRPIILLKDGTESHQGKPQVISNINACQVISDAVRTTLGPRGMDKLMVDSKGKTIISNDGATIMKQLDIVHPAARTLVDIAKSQDSEVGDGTTSVVLLAGEFLKQAKPYVEEHVHPQVIARSYRKASCMAIDKIREIAVTVDKGETRALLEKCAMTTLSSKLVASKKEFFAKMVVDAVMQLDELLPLNMIGIKKVSGGALEDSVLVSGVAFKKTFSYAGFEMQPKKYHNPKIALLNIELELKAERDNAEVRVDSVEEYQNIVDAEWNILYDKLAKIHASGAKVVLSKLPIGDVATQYFADRDMFCAGRVAEEDLRRTMKACGGCVLTTVQDLKDSNLGSCEKFEENQIGGERYNIFTGCPNSKTVTMILRGGAEQFIEETERSLHDAIMIVRRAVKNDAVVAGGGAIEMELSKYLRNYSRTVAGKEQLLIAAFAKALEVIPRQLCDNAGFDATNILNRLRERHAKGDKWSGVDINNEDIADNLEACVWEPAVVKINALTAATEAACLILSVDETIKAPQSNTDPTAGRPFAPTLNRVEAFREHGMAVTRDYISQPRMIYKTVCGVNGPLVILDQVKFPKYAEIVQLVLADGTPRTGQVLEVSGDRAVVQVFEGTSGIDAKHTVCEFTGDILRIPVSEDMLGRVFNGSGKPIDKGPAVLAEDFLDIQGQPINPWSRIYPEEMIQTGISAIDVMNSIARGQKIPIFSASGLPHNDIAAQICRQGGLVKRPGKSVMDDNFAIVFAAMGVNMETARFFKQDFEENGSMDNVCLFLNLANDPTIERIITPRLALTTAEFLAYQCEKHVLVILTDMSSYAEALREVSAAREEVPGRRGFPGYMYTDLATIYERAGRVEGRNGSITQIPILTMPNDDITHPIPDLTGYITEGQICVDRQLNNRLVYPPINVLPSLSRLMKSAIGEGFTRKDHADVSNQLYACYAIGKDVQAMKAVVGEEALNAEDMLYLEFLAKFEKNFISQGRYENRTIFESLDIGWNLLRIFPKEMLKRIQHKILAEYYPRGESKRRLNTAQFNS-TGLFESPHGFIILRDKAITEAENLIAEAVSRKRKMVQIFDDLSDCLCRVADLAEFVRTGHPDARFSSAAREASVAISSLVEQLNTNVALYSALRSVVEGE-DIVPTNIEKHVGRLFLFDFEQCGIHLDERTRKDVVALNDHILYTGSYFLQNSQKPRVVSNKVLPQNLFAVTGLNADSPQEAIRETTYKIFLLPDDEQMSLLDNLLESRQELAQLCGFKTHAHRVLKGSIADTPENVSFLSYLSKELRPRAEKDYQEMLRMKAWDIPFFSAYLSLSTCMEGLDMIFQSLYGVTIRVAGELWHPSVIKMIVKDSDDPIGIIYCDLFERAQKPHQDCHFTIQGGRKADGSYQIPKVVLMLNLPPPLLTPASMDNLFHEMGHAMHSMLARTEYQHVTGTRCATDLAEVPSILMEYFASDPRVVTRFARHYKTGEPMPAILASNLEASRVIFQASETQLQVFYAFVDHAYHSQYPRSTTEILREVQNEHYGVPFVENTAWQLRFGHLVGYGAKYYSYLMSRAVAASFWQKAFKADPLSREAGLYREKVLAHGGSLPPAELVRGKLLAEALIRDIMPSDAKKKRDAKKKEALKNRNMPDPTNGEDEMDEVTKKFEEDMRMNAAARAVTGVLSIHPRSRDVKIENLSITFHGWEVLQDTKLELNCGRRYGLIGMNGCGKSALLSAIGRRELPVQDCLDIYHLTRECPPSDKTALQMVLDVDKERLRLEKLAEELASAEDDTSQEQLMEIYERLDDMNAETALAKASYILHGLGFTLAMQHKKCRDFSGGWRMRIALARALYVKPHILLLDEPTNHLDLDACVWLEEELKTYNRILILISHSQDFLNGVCTNIIHMNQRKLEYYGGNYDQFVITRAEQLENQMKRYNWEQAQIADMKDYIARFGHGSAKLARQAQSKEKTLAKMVAGGLTEKVVQDKSVSFYFPSCGPIPPPVIMVQGVSFRYSDNTPLIYKNLEFGMDLDTRVALVGPNGAGKSTLLKLLCGALVPTDGIIRTHSHLKIARYHQHLHESLDVDLSALEYMMKSFPDVREKEEMRKIIGRYGLTGRQQICPIRQLSDGQKCRVVFAWLAWQVPHMLFLDEPTNHLDMETIDALADAINHFEGGMVLVSHDFRLISQVAKEIWICEHQTVTKWPGDIKTYKQHLKNKVMKELEKMVLADLGRRITGALRNLSTATVINQEVLDSMLKEICAALLESDINVRLVKQLRDNVRAAIDIEEMAVGLNRRKVVQSAVFKELVKLVDPGVKAWTPQKGRSNTIMFVGLQGSGKTTTCTKLAYHYMKKGWKTALVCADTFRAGAFDQLKQNATKARIPFYGSYTEVDPVVIAAEGVAKFKAEHFEIIIVDTSGRHKQEDSLFEEMLEVSNAVSPDNVIFVMDASIGQACELQARAFKEKVDVASVIITKLDGHAKGGGALSAVAATKSPVIFIGTGEHIDDFEPFRVKPFIQKLLGLGDIEGLIDKVNELKLDENHELIEKLKHGEFTLRDMYEQFQNIMKMGPFNQLMGMIPGFSADFMTKGNEQESMSRLKRLMTIMDSMTDDELDNREGARMFAKQQTRITRVARGAGCTTFEVHELLNQYTKFAGMVKKMGGMKGLFKGNDLAKNVNPAHMNKLSAEMAKMIDPRVLQQMGGFSGIQNMMRQMNGGILTEDPAFLKLRSL-SPITKPLRLKDLFDKDPSRAEKYTLKLQSGPETLLVDYSKNLINDEIMAGLLELAKNRGVEAMRDRMFKGEKINFTEDRAVLHVALRNRSNRPIEVDGKDVMPGVNAVLEHMRSFCHQVISGEWKGYTGKKITDVVNIGIGGSDLGPLMVTEALRPYQIGPNVHFISNVDGTHLFETLKRVDPETTLFIIASKTFTTQETITNAESAKIWFLGKAGDKSHVAKHFVALSTNAAKVTAFGIDKENMFEFWDWVGGRYSLWSAIGLSIALFIGMANFEKLLTGAHFMDEHFRTTPLEKNIPVILALIGSWYINFLGADTHCLLPYDQYLHRFAAYFQQGDMESNGKYVQRDGRIVEHETGPIVWGEPGTNGQHAFYQLIHQGTKLIPCDFIAPAKTHNPIQGGIHHKILLANFLAQTEALMKGKSSEEAKAELEASGLKGEALEKILPHKVFRGNRPTNSIMVQEVTPFTLGALIAMYEHKIFVQGIIWDINSYDQWGVELGKQLAKVIQPELEGKEPVSSHDQSTNQLINFIKSFNNNASYQDKDKPAQVRQSNIAAAKAVCDAVRTSLGPRGMDKMIQAVSGDVTITNDGATILQQMQVLHPAAKMLVELSKAQDVEAGDGTTSVVVIAGALLDAASKLLHRGIHPTIISEGFQAAAKECVDILSSLAIPVELSDRESLLKSATTSLCSKVVSQHSDLLAPIAVDAVLRVIDPTNVDLRDIKIIKKLGGTVEDSELIDGLVFTERLAGGNAPHRMEKAKIGLIQFCISPPKPNMDHQVIVSDYAAMDRVLREERAYLLNIVKQVKKTGCNVLLIQKSILRDAVSDLALHFLAKMKIMVVRDIEREDIEFVCKSIGCRPIASLDHFVPEALGTAELVEEVTSSKFVKVTGIANP---RTVSLLLRGSNKLVLEEAGRSIHDALCVVRCLVKKRALVAGGGAPEIEMSLKLAERARQLEGLHSYCYRAFADALEIIPYTLAENAGLNPIQTVTELRNKHAQGEKTFGINVRKGCVTNILDENVLQPLLVSTSAISLAAECVRSILKIDDIVQTVRMSKRDVVYLWDPDVGNFHYGPGHPMKPQRIAVTHSLVLNYGLHRKMRIYRPYRANPHDMCKFHSEEYVNFLQRITPQNMESFDKKETLFNVGDDCPVFSGLYDFCSLYTGASIDGAWRLNNRTCDIAINWSGGLHHAKKFEASGFCYINDIVVAILELLKTYARVLYIDIDVHHGDGVQEAFYLTDRVMTVSLHKYGAYFFPGTGDMYEVGAESGKYYALNVPLKEGIDDASYFQVFKTVISSVIDHYRPGAVVLQCGADSLAGDRLGCFNLSIKGHGDCVKFVRDLNIPLLVLGGGGYTLRNVARAWTNETAILVNEQVSPEIPYNEYLEFFAPDFSLFPD-------ENANSKQYLEAILKYTTENLRCLEHAPSVQMQDVPPDIVDLADDEPLIENSKEHPAEFYD--------MSD--DNMSDNEEDYDLEYSEDDDSQPDVDLENQYYNSKALKQDDPKAALQSFQKVLDLEGGQKGDYGFKALKQMVKINFRLGQYEEMMSRYKQLLSYIRTAVTRNYSEKSINSILDYISTSKRMQLLQEFYEVTLDALKDAKNDRLWFKTNTKLGKLYLDREEYPKLQRILKQLHLSCQNVDGSDDLRKGTQLLEIYALEIQMYTARKNNKELKKLYEASLQIKSAIAHPLIMGVIRECGGKMHLREGEYNFAHTDFFEAFKNYDESGSPRRTTCLKYLVLASMLMQKEINVLDSQEAKPYKDDPEIVALTDLVDAYQAHNISRFEAIVSPHKESIMKDAFIKEHIEQLLLNIRRQVLIRLIRPYTRIKIDFISCELNIPSQDVESLLVSCILDNTIQGRIDQVRQVLELSS-SSVQKRYSGMEQWALQIQNLQNTMTNPAAHLGNLNPIQILNHQAEEEKAENARLSSFVGAIAIGDLLKSTLGPKGMDKILLCETSRDSKVEVTNDGATILKAIGIDNPAAKVLVDISKTQDDEVGDGTTSVAVLASQLLQEAEKLVGMKIHPQTIISGWRRSVNASRAALEDFSQDRSNNEAQFKKDVMNIARTTLGSKILAQHKEFFAQLAVDAVMRLKGKCNLDAIHIIKKLGGSMLDSYLEAGFLLDKKPGLNQPKRVEKAQILIANTPMDSDKIKVFGSRMKCESIARVAELEEAEKLKMKRKVDSILAHKCNVFINRQLIYNYPEQLFADAGIMAIEHADFEGIERLALVTGGEIVSTFTSPETVKLGTCDLIEEVMIGEDKLLRFSGVPLGEACTIVLRGATQQILDEAERSLHDALCVLTSVVKEKKICYGGGSAEMLMAEAVDKLALTTPGKESLAIEAFARALRQLPTIIADNAGLDSAQLVSELRAAHATGKKSFGINIVDAKIDDMEKLGITEAFVVKRQVLLSASEAAEMILRVDNIIKAAPRKRVPDRSH---------KPQFLCKLDGHQETVNQAILIGAEDAVISISDDKTIRIWSRRDSGQYWPSICHTMPAAASAMDYEHGTRRLFVALDNGTITEFDLSDDLNKITLRRSYIAHQQRVASIKFSLATEWLLSAGKDKYFQWHSTETGRRLGAFQSSSWCTTVEFDAASRHAFVGDYSGNITMLKLTETNYQPVTTLRGHSGSIQTLLWDEKKSLLISGSFDQVIIVWDIGGGKGTAYELCGHKQRVTGLAIFG-QKLLSVSEDSTLVVWDMSAKRQGTPEWAERDCCERCARPFFWNVKAMLDQKQVGLRQHHCRRCGRALCDKCSSNRSPLPVLGYEFPVRICDECHLHVSDRDRQSLAEFHDLKTPVAGMSLAPNGKLMATVGSDRNMKIWDIATVIMGDIAAEGAVSKKALKKAQKDAEKAARKAQHKAEREAQNPNKDDDVSKGKYGSYGMIQSSERLERTLTPISSCTSKLVGQEVWVRGRIHVSRSKGKQCFLILRQACCSIQCVLAVGENVSREMLKFVSNIPKESIIDVCGKVMATPSKIDSCSQQDVELSTLQTWLVSASEARLPLQVDDAARPDVVKNEEDALNIRVNQDTRLDNRILDLRTPANQAIFRLEAGVCHLFRESLSKKGFVEIHTPKIISAASEGGANVFEVSYFKGSAYLAQSPQLYKQMAIAADFGKVYTIGAVFRAEDSNTHRHLTEFVGLDLEMAFEYHYHEVVDTIGHMFIDIFKGLERNYGAEIEAIRRQYPSEPFQFLEPSLVLKYSEGVEMLRSAGIEMEDDEDLSTPNEKLLGRLVKAKYNTDFYILDKFPLAVRPFYTMPDPENTKWSNSYDMFMRGEEIISGAQRIHDPEFLMQRAKQHGIDVSQIKSYIDSFRYGAPPHAGGGIGLERVVMLYLGLDNIRKTSMFPRDPKRLTPAMEEGLDLAEIEPLEFSRVKIIFPKECRIRRVTYRGRLELRFWWLNGLKQEPITRSCGEIPIMVKSRKCNLHGMSPDQLIERGEELEEFGGYFVVNGNEKVLRLLIMQRRNYPIAMARNGWKGRGSMFSEFGVSLRSCRKDSQNMVLHYLTNGTVQVMITNRKELYFVPIVLLLKALVDKSDYEIYKSLIKGFEDDSFYKGCITNMLRLVQENILTQDAKEFIGDKFRWATNSDVCDKLLDRCVCVHLDDNESKFNLMCFMVRKLFAAAKNKCALESADSTMNQEILLPGHLYLNVLKEKIEGLLLGVKLSTEKKMSNALDLTHAMNYFLSTGNLISKSGLSLQQVSGFTIIAEKLNFWRYIGHFRCVHRGSFFMQMRTTSVRKLLPEAWGFLCPVHTPDGGPCGLLLHLSAMCEIVVQLDGRCLGWILRYLKASG-IAPTLEICVIPRTEQNSLYPGIFLFSTPARMMRPVLNRTQAVEWIGTLEQVHMDICVVPEEATTHQELSETAMLSILANQIPWPDFNQSPRNMYQCQMGKQTMGSPMHSYRNRADNKLYRLNPQSALVRPTAYDHYKMDEYPSGTNAIVAVISYTGYDMEDAMVLNKMSVERGFKAGVYKTETVNLRVDYDGLPYIGSHGDPVCCYIQLKVVKYHSSEPATIVEVKILGNLQQVQFTYLIRTPMIGDKFASRAGQKGVCSTLWPTENMPFTESGMVPDIIFNPHGFPSRMTIGMMVESMAGKSAALHGYVHDASPFKFSEDDPSSEYFGELLQKAGFNHHGTERIYSGVDGREMDADIFFGVVYYQRLRHMVADKYQVRTTGPIDSLTRQPVKGRKRGGGIRFGEMERDSLLAHGTAFLLQDRLFNCSDKTLCAACGSVLSIPIPYVFRYLVAE-LASVNMKITRETSRDLHRHQRNEPELHPLATAREYTAAVNAAKIDRIFAKPFLCSLGHRDAVEIVAKHPEKISGVVSASVDGEVRWWDLTNRRCVGNVQAHDGPVRGLCGQDQSIKTILSKLAHHYSQMFATAGETVSLWEERNEPLRSLW-GVDTIYVTFSPIESLSSDRSIVLYDIREATPLRKVTLEMRSNALCFNPMQAMHFTVANENFNLYTFDIRHLKTALQAHTDHVGAVLSLDYSPTGTEFVSGSYDKSVRIFRSREVYHTKRMQKVTSVVYSMDAKYVISASDEMNLRLWKAKASEQLGITNYNETLLSRYGSHPTVKRIVKHRHIPKQLYKEKQIMLSSRKTKMGMQLLHRGAYQDALSHYHAAIEGDDSNYQSYYWRATVYLALGKSKLAVEDLNKVIDLKEDFIKAREQRGNILLKQGYLDEAHIDFEYVLRLDPHNLEAVIEQLKNDVISILQGVWNLRLRELRAECYEAIGDLNSAISDLRPAIRSVPDNTKGYLKLARLFHKHGEPEEGLTTIRECLKLDPDHKECYKSIKKLVKSMQDDCLEKIVTLVKTKCHCASKGGDAVNLCSEALRYDPILCDRAEAYLNADDFAQDFASARELDQRAAEGLKRAQKLEKAKGKRDYYKILASKGEITKAYRKLAAKWHPDQY-QGDD--KKGAEKMFIDIAAAKEVLTDPEKRAKFDRGEDPLDPDSGFHYTFHFMLVLFESPAGFAVFKVLDEKKIQKSENLFKEFEDASDASKILKLKHFQKFEDMTQALSAATGAIEGKLTKPLKKILKKL---EATETLAVADAKLGNIIKEKMDISCVANSSIQELMRCIRSQQEALITGLSQKEVTAMALGLAHSLSRYKLKFSPDKVDTMIIQAVSLLDDLDKELNNYVMRCKEWYGWHFPEMGKVVTDNMLYVKTVCKMGMRSNAITLDLSDILPEDQEAKVKELAEVSMGTEIAPDDVDNIKHLCEEVIQMTEYRATLHEYLKNRMAAVAPNLTILVGELVGARLIAHAGSLLNLSKQPASTVQILGAEKALFRALKTKHDTPKYGLIYHAQMVGQSSQKCKGKASRWLAAKAALAIRVDALGEDNDTDMSLKNRANLEARLKMLEEGKLTRISKGG-KSNKFDVYKHKSEVHEFKPANDSTI--GNKRKFGADEE--QEPKHFKAK------HFDEDEKAEAEETPAKKAKNKKKKSLAAADEGENSSVVEGKIKKKKKSKV--AEEVAEEEEEGA------PKKKKKKKSKAKRYIWPKDKPNIKRTVALALGLLVAAKLVNISVPFMFKHLIDFLNENTGLVVGYGLARAGAAGLNELRSAVFASAAQHSIRSMGRKLFSHLHDLDLGFHVGRQTGALSKAMDRGTRGINFVLSALVFNVAPTLLEVTLVSTILWYKCGQFAAVTLGCIGTLLVTQWRTQFRIDMNKADNKAGSRAVDSLINYETVKYFNNEKYELKDYEKASLKTTTSLAALNFGQNAIFSTVIMYLASEMTVGDLVMVNGLLFQLSLPLNFLGSVYREVRQAIIDMQTMFALTEIESSKLSISKEIEFENVTFQPILRNVSFKIPTGKKVALVGGSGSGKSTTVRLLYRFFDPIDGRILINGQDIKDVSLRRGIAVVPQDAVLFHNTIRFNLQYGDLQAEVEESARMAEIHDAITGWYETQVGERGLKLSGGEKQRVAIARAILKNSPILVFDEATSSLDSITEQKIMKALRAATGKTTLCIAHRLSTIADADEIFVLKDGTLIESGHQSLLRSFYAYLWNQQHL--MRTRMRAGATAFKSAVIDGISVRGRFVSSGGTSRAAAAAAPTHNLSRWHRTGSRDQSLRPAGLSRLLPHMAFKGTEKRSQTDLELEVENAGMHLNAYTSREQTVYYAKCLTKDVAKAVDIIADITQNPKLGEQEIERERSVILREMEEVEGNLQEVVFDHLHSVAYQGTPLGMTILGPTENIKSLKKQDLQTYIKEHYTGSRLVIAGAGGIDHDELVKLAEQNFGKVSNSMDQKMPCRYTGSDMRVRDDDMPFMHAAIAVEGAGWKNPDNIPLMIGNTMIGSWDRSHGGGNNATSRLAAAYAADQVVHSFQSFNTCYNDTGLWGIYFVATNGVVQRAVLQIQEQWMRLVTGATEADVTRAKNLLKTNLLLQLDGTTSICEDIGRQMLCYGRRIPLHELEARIDAVDAATLRKVCEEYLYDKCPVVAAVGPVEGLPDYTILRGHMWKHSVHTMVFRSLKRSHDMFLCDEGALPPIDEKAHKLRMDVKARDEYGRVMHLVNQNRRVPAPQALVLAPSQARAPLKKPSQPKPQWHPQWKLCRVISGHNGWVRCVAFDPSNEWFCTGSNDRIIKIWDLASGKLKLSLTGHIAGVRGLAVSQHHPYLFSCGEDKQVKCWDLEQNKVIRHYHGHLSGVYTIGLHPTIDVIVTGGRDSTARVWDMRSKSCIHTLTGHTNTVASVLVQATEPQIISGSHDSTIRLWDIVAGKTRVTLTYHKKSVRALALHPKLNMFASGAPDNIKQWMCPDGKFIQNLTGHNTIVNCLAVNHSNVLVSGGDNGSMQFWDWKSGYNFQKLTTPVQPGSIDSEAGIFQMAFDVSGSRLVTCEADKTIKIFKEDETATEEG------P--VTGSTSEYFINCSSEHYLEILGINVKAKNFLVFQGAVESIAMKNPKERTVLFEEISRSLLDSQEETYQKKKGIAAERKEAQMEEAEKYQKLKEDVQVNLHLFRLFHQEEDLEKRKKKERIENELREKKKVEQTFRDDVELNKRKPAYIRAKEKTAHTQKKLDAAKKSLDAATKTHRSHQGIEELEQELSQVEEAFDQEDVSLEESQVKEYNRLKEKAGKMASAALQEYDSVARDQKTDQDHLDNELRKRNECEAKLKELEENQRRVNKLVDLHDLKEEERKLEAKKRLSQKFEDVSSLGDAKVDKHEDARRKRKSEIVEHFKKLYPGVHDRLVNLCHPIHKKYNVALTKVLGRNMEAIVVDTEKTGRACIQYLKEQMLEAETFLPLDYIDFKPLKERLREFKDVPNVKLLYDVLKYEPLSIKKAVLYATNNALVCETAEDAAKVAFQAPDGKRYDAVALDGTYYQKNGFISGGSSDLAKRAKRWDDKDFHKLKDQKEKLQEDLREAMKTARKESDLTTIESQIKGLETRIKYSKIKQLEQSMREREVRINEIKAKQNTVEDDVFRDFCAQIGVANIREYEERELHASQEREQRADLENQKNRVASHLEYER--TKDTLAVELDQQELENLKEIEQKQKELIEQQVNLISQLKNERAVKKSKVDEIDEEVAEIRKRLTTQQKEVTAVQKGVTQAETKLEQKRSERHTFLQSCKLEGIRIPLILGSMQQMYEREALKIDYSQLRMEKEINLQRIQAPNFKAMEKLDSVKERLKDTDTEFEHARRKAKSNFELVKRERTCFEHVSNCIDDIYKSLTNNPSAQAFLGPENPEEPYLEGINYNCVAPGKRFQPMSNLSGGEKTVAALALLFAIHSYQPAPFFVLDEIDAALDNTNIGKVARFIREKTQTSFQCIVISLKEEFYGHADCLVGICPDPGECTISRIYTIDLSLTEAMLSFLRDAINKGDLNSMQDLYENSLTKLMDQYFLIIYKE--LYYRQIYADDRFNSYYNYVDFFNYILSSEPVTLELPNQWLWEIMDDFVYQF--QSYCVYMWNVHSVLNVLHSLVQKSNINQQLEVYSKGGNPEEVAGEFGRRPLYKMLGYFALIALLRLHSQLGDYYQAIKVLQHLELNRKGLSRVPSCQVSTYYYVGFAYMMMRRYEDAIRTFVNVLVYIMRTKRSYQLKQINKQMDKMYALLSICMALHPQRIDESVLQLLKSKQLQAMQSGDLDTFESCPRFLSPVQWNAFRGEIEAQLTIRSFLKLYTTMPVEKLAKFLLMSFKHLMSNMVLDGEFQTGSDMDFFIDKDMIHIADTKIARRYGEYFIRQYHKVEEAFE-MAMRS-AILIPRSVQVLGARSQSVFSHVEMGPPDAILGVTEAFKKDTNPKKMNLGVGAYRDDEGKPFVLPSVRQAEQKLAEQKHDKEYLPIGGLPAFCENAAKLALGKDSFVIKTGRNATVQGISGTGALRIGAAFLEKHLKGNKTVYMPNPTWGNHIPLFKHCNFEVKQYRYYQPKTCGLDLQGALEDISKIPEGSVILLHACAHNPTGVDPSASEWLEIEKVVRKRNLFPFLDMAYQGFATGDIDRDASAVRIFSQSG-PMCLAQSFAKNMGLYGERVGAFSLLCDSKEEAERCMSQIKILIRPMYSNPPVHGARIANLILSDPDLYAQWLADVKLMAGRIISMRERLQQGLKNEGSTRNWQHIVNQIGMFCFTGMKPHQVEKLTKDYSVYLTKDGRISVAGISSNNVDYLAHAIHNCTKRILRPVRHLAHCVLMAIVIYMFVESSTPASDVDPFAEYGVVLTILLYLFRLLPLLALPQSLTNFFGLTLYNAFPPKVKLKVDPLEAPFLCIRVVTRGGYPDLVKANVQRNLQTCLDLGMVNFVIEVATDKEVY---HPKIKQTVIPNDYQTSSGAMFKARALQYCLEDDVNVLDDDDFVLHLDEETIVTRDAMKGVLNFISQRRHPFGQGMITYANERIVNWVTTLADTYRVADDLGKLRFQFKFFHKPLFSWKGSYVCCRLGAERAVSFDNGPDGSVAEDCFFSMVAFSKGYSFEFIEGALWEKSPFTISDFLQQRKRWLQGIFLVVHSPRIPAKFKVWLACSLYAWATMPLSTSNLFLAPRYPLPCPQTFNILCAMIGALNLYMYFFGVLKSFSIKRQGLLKFLLCLGLVVVAIPINIFVENIAVVWGLLGDKHKFYIVDKSVVMEKQTEGCENAAKLQCPTCLKQGIEGSFFCNQQCFKSSWSVHKSVHKATPN-KYNPWPHYNFSGRLRPGRVGPMRDVPAHILRPDYADHPEGIAVSEQAMKGA-EIKVLSAAEIEGVRVASKLARECLDTALRAAKPGVTTDELDRLVHEAAIERNCYPSPLNYYLFPKSCCTSVNEVICHGIPDDRVLEDGDILNVDVTVYHKGFHGDLNETVFIGNVDDAAKNLVRVTYESLQKAIESCRPGVLYRDIGNVIQKHVQPHGYSVVKSYCGHGIHSLFHTAPSVPHYARNKAVGVMKPGHIFTIEPMISEGSYKDEVWPDNWTAVTIDGKRSAQFEQTLLITETGVEILTRRREKNGQPWFMDQSSIRRTLKAKSAKRRELLAQQLGAGCAENLGLLLGNEQDSASENKDQQNEDEVMAYRDSSTFLKGTQSANPHNDYCQHFVDTGQRPQNFIRDVGIQDRFEEYPKLKELIRLKDELIRETAKPPMYLKCDLMQYNLRDLNSKFDVVLIEPPLEEYQRSCGVTSSRFWAWEEIMKLEIEEIAAPRSFVFLWCGSSDGLDLGRQCLRKWGFRRCEDICWIKTNIDDSKVKNVEPRAVFQRTKEHCLMGIKGTVRRSTDGDFIHANVDIDLIISEEPQFGKMEKPEEIFHIIEHFCLGRRRLHLFGKDKAIRPGWLTVGPELMNSNFSSEAYNSYFNSPNEYLTGCTERIEALRPKSPPPKANLGGASRGGTGRGAFSKLGLDKLALSGKRVIIRVDFNVPIKEGKITNNQRIVAALPTIKHCLEKGAKAVILMSHLGRPDGNVNAKYSLAPVAEELKKLLSKEVTFLSDCVGPAVEKACENPAVGSVILLENLRFHIEEEGKGVDAAGNKQKADAEAVKNFRASLTRLGDIFVNDAFGTAHRAHSSMVGITLPERAAGFLMKKELDYFAKALDNPARPFLAILGGAKVKDKIQLIENLLDKVNEMIIGGGMAYTFLKINKGMKIGTSLFDEDGAAIVEKLLAKAKQNNVTIHLPVDFVTADKFDENATVGSATVETGIPDGWMGLDCGPKSIEQFKAAVGRAKTIVWNGPAGVFEFEKFAAGSKGIMDAVVEATKTGAVTIIGGGDTATCAAKWNTEDKVSHVSTGGGASLELLEGKDLPGVVALSEAMRTFGDRPIAFQLEDGGDYYYIGTEVGNYMRLFRGTLYKKYPSLWRRPVTVEERKKISQMNMSHHSAANFISLLKKVEVDDLIDGNEEKYRAAPVQESDGGAHGAKAARPSFMPAAPNNAHHLDAVPCSTPINRNRLQHKKNKSFPMLYDDLDPATLHENAAMPECLVPIRLDMEIEGNKLRDTFTWNKNEAQISPEQFAEILCDDLDLPPLLFVPQIAASMRQQIEAFPTESLLDEQTDQRVIIKLNIHVGNISLVDQFEWDMSEKLNSPEEFATKLCSDLGLGGEFVTAIAYSIRGQLAWHQRTYAFSEAPLSQLEMPFRPQSEAEQWCPFLETLTDQEMEKKIRDQDRNTRRMRRLANTGWMGANGSLLDREDAIPSTERYLGLVNFGNTCYCNSVLQALYYCKPFREKVLEYKAKNKRTRETLLTCLADLFHNIHSHKKKTGTLAPKKFIARLRKDNEVFDNYLQQDAHEFLNYLLNTIGDLLQAESIWVHEIFQGTLVNETRCLTCETISSKDEDFLDLSVDISPNTSISHCLRGFSSTETLRGEHKYHCEQCNSKQEAQKSLKVKKLPPILALHLKRFKYTEQQNRNTKLSWRVVFPLELRLFNTSDDAVNGDRLYDLVAIVVHCGTGPNRGHYISIVKSHGVWLLFDDDIVDKIDPTTIDDFFGLTQDTPKASESGYILFYQSKEGSDIDSWIKVAQECKYLPESDLKKLCNLVCNILIEENNVQPVSTPVTVCGDIHGQFYDLEELFRCGGQVPETNYVFMGDFVDRGYYSLETFTRLLTLKAKWPNKITLLRGNHESRQITQVYGFYDECQQKYGNANAWKYCCKVFDLLTLAAIIDGEIFCVHGGLSPEIKALDQIRTIQRNQEIPHKGAFCDLVWSDPDEVDSWCWSPRGAGWLFGAKATHEFMQYNGLSLICRAHQLVHEGYKYMFDDKLVTVWSAPNYCYRCGNVAAVLEISDPQNKNPKIFDAVPDNKRVIPERVTPYFLVD-VSAEESQDADATTIEELKEHTNHIQKAVANKESRFILRILRLLPATRKKLNSKLLRKIINGYYTHDKVHKDLLLSFV--EDTDTEAAQKSAHLALLPEVDVYLHLLLLVYIVDSKNMERAVKCSELLMAKVEGHSRRTIDLLAAKSYFYYSRVYELDGKLSNIRGFLLKRLRTATLRSDFEGQAVLINCLMRNYLHYSLFKQAAKLVSKVTFPEMASNNEWARYLYYLGRIKAIQLFYTDAHKNLLQAIRKAPQHTALGFKQTVYKLAVTVELLLGDIPDRATFRQPALRKSLAPYFQLTQAVRTGNLALFNKVLENYGTRFQADHTYTLIIRLRHNVIKTGVRMINLSYQRISLADVAAKLQLDSAESAEFIVAKAIRDNVIEATIDHDKSYMQSAENIDVYCTGEPHCQFDQRIQFCLDIHNQSIKAMRFPPKSYNKDLESAEERREREQQDMEYAKEEDDDAFQVSVKLAVTLSAFAARTLWKSAVHDMIANLKSSHPLLIEFLVALPDQNVK--CLTRDVLALCSSALADALREVSMRAIPPDLCITLL-NVPHNHACDALVNCLTHPDWRLPNTIAEILVQVSRCFIFRSTRSLARARNS--HRYSGVGETHSSVILQSL--QGEKRMEAFLQMLLECIGTPGYYPVDEILSRVPLTFWHLLLDDLSRLEV-SAKGRELHPVYEELVRLLTKSRLPDHGSMDADEMEDHRCYRQDIAD--CYVYVHTLLSKSMFRYLIFELKSAHNWKPIEACLFSLNAVGEMADGIEHGNVVHEVLDLLPQIPVNDEVMSQVMTAIGIFAEQQIGPLVHLLLRGLQEISFAASMALKDLARAHAEHLAPVANDILQAIGILKHRDRVRLVAIVGHVVSALSSDQALQSLTALMAPFVMQLSEMTNLVEQLAPLFSQIAAKY-PTNTKVVNGLAECLRRAVPVLETTPLLKQLLSLCCELQIECASSIDLHERTDLVEAFYTMASLLKKFAIETLLECTPETFTFRSLVQFVTASERIELRKTLEGIVATLIENMHVSRKLIEQEADVFLALNRSASWLNRFLQSQDSKTNFVRQILRERSNKRILTKVV--SDFTVKVKVKWKESY-DVDLDLPEVFKAQLFALTGVTPDRQKVMCKGAILKET-WGATILMMGSKGEIPQPAEKPTFLEDMDASQLATALKLPCGLYNLGNTCYLNAVVQCLRTVPELVKLATYQTQITCSLRDLFWNMEHAVIQPLFLVDFWRRTFPQFAEKGENGQQDANEAWTELVRVLDGFMKMLFGGELVTMKCSESESETTEEFFQLSCFIS-TEVRYLLAGLKLRM-QENITKMSPTLGRDAVYKTSKLSRLPGYLTVSIVRFFYKEKEAVNAKILKDVKFPMMLDVFELCSQDLQQSLIPQRDKFKVWEDEFAFPNDGSNNSGFYQLQAILTHKGRSSSSGHYVGWIRREWFKCDDETVIHEDDIMKLSGGGDWHVAYVLLYGPRLLENLINKMLPKELLLKVFSFLDIVTLCRCAQVSREWNLLAMDGSNWQNIDLFSYQKDINCDVVSYIAGRCGRFLTVISLRGCEDISGEALIQFSEHCPNIEKVLSCCRKITDDAIVALAKSLYIDSCVELTDRSIMSF----KNLRDVNISWCRKITQEGIGMLGS--EHLVRFTAKGCVTNEAMSRLASKLEALDLQCCPYVFDAAIIAVAQNCHELRNLCASGCSNLTDASTQALAQGCLHTLEMASCNRCGDAGFVPLVKACHELRRLDLEECVLITDSTLNSIALSCPFMDSLSLSHCDQITDQGVLKLSQNVIELDNCPFISDITLDCLVDCLQRVELYDCQLITQESIKKFKPGLRLHTYFAPTTPRQRYCRCCVIVSSKGARVFCPDHPEANLIEDYRAGDMICPQCGLVVGDRIVDVGTEWRVFQNEKSSNDPTRVGAAENPLLNGSDLSTIIGRTGDASDESGNAKYANRKTMSSSDRALIGAFREISAMGDRINLTRNIIDRSNALFKQVHDGRSLKGRSNDAIASACLYIACRQESVPRTFKEICAVSKVSKKEIGRCFKLILKALETSVELITTGDFMSRFCSNLALSTAVQKAATHIARKAVEMDIVAGRSPISVAAAAIYMASQASAEKKSQKEIGDIAGVAEVTIRQSYKQMYPRAAQLFPEDFKPANEVPWVEKYRPEKFTEIVGNEETVARLEVFSRQGNVPNIILCGPPGVGKTTTILCLARLLLGSSFREAVLELNASNDRGIDVVRNKIKMFAQTKVTLPPGRHKIIILDEADSMTEGAQQALRRTMENFSKTTRFALACNTSDKIIEPIQSRCAVIRFGKLSDAQVLAKIIDICRKENVSYAEDGLEALVYTAQGDMRQAIGNLQSTHVGFGHVNGKNVFKVCDEPHPLIIKEMIEYCAKGDIDEAYARMQTLYSLGYAAEDIVSNMFRVTKTNTLPEYLKLEFIKHIGLTHMTVLQGLGSLLQLTALLADLCQTIEIKSFVKKEHSLVKPYQGSGMSIPNWDFTGSTMVSSNYIRLTADVQSQQGSIWNKVPWEVQINFKVHGHGKDLFGDGMAIWYTKDPLQPGPVFGSKDFQGLAVFLDTYANQNGHHNHAHPYISAMVNNGSLSYDHDRDGTHTELAGCEAKFRNSEPETSISIRYEQDTLVVSTDILGKKEWKECFRVSGVRLPTKYHFGISAATGELSDNHDITSIKVFELDERREFITPQAAPHRDHVDDGMSGTKFFFVMLFSMLFLVCYYYKKHQENARKRFYRDRDAFDDIRDKNCKIQDINELFLPNDTVTHVPNIKRLNIDPVFPNRTNLLHIHNMAISKAFFLSFILQRAKDDEPGFMYYFMSVISDVAANRFINASAIYYAPNMSFTPSYKGFFNKTMPLFAPRAFRSDDFNDPYHLEGTSTLNTIEATDLGAISLNYSSDQYRINEWYSSWLPDLTKRQDSKTTYTVQITGNNDTFVWHGPPAANDNPGPVKWVRPYFDCGRSDKWVYGATSPIPDIYPRHTQWRHIEIPRYVAVSVMELDFERIDINQCPFGPGN-PRPNYFAGTSRCKNETTDCEPVHGYGFRRGGYQCRCKPGYRRPKIVRNPYHGELIEKASEYEYEHGYSCDKIGYIGVLTQNVNNYMAIGTRIDPLMGGDVVYGKEVQLENEARMAVRLANFISGFMQIVDPKDLFAEFRVPDKPLTADQMIGEVMSIVIGDQKVVGAGVYFDYKAFFGPY--AWRLGRNERKYFVDDTTKIQIRYNSSGIKYDHYPLQYKAADVGYWTSPYFDCGGYHNSWIVTYAVPFFGWDSLRARLQFKGVVAVIELDQLEINQCNAFENTHKCERKSSRCVPILGRGFQGGYKCECLQGYEYPYNDPITYFDGQIVEAPSRFERMTHVSGTDKSKYGGRYMVTALPGDGIGPELIGYVKEVFRYGGVPVDFEEVHLDSSRDDVDLLEQAITAVKRNGVAIKGNIETRHNDPNCKSRNVELRLRLGLFANIVHVTSQPGIETRHQDIDIVLIRQNTEGEYSSEEHTSIKGVVESLKVITKTRSDEIARYAFEWAKNNGRKKVTCVHKANIMKLSDGLFLSRCTEMAKEYPELEFDNIIIDNCSMQLVANPNQFDVLLLPNLYGNILTNLACGITGGPGIASGRNYGKDYAVFETGTRNTGKSIAGKNIANPIAMMNAGVDLLYHLGLTDHAQVIARAIDKTINVDKLHTPDLGGQATTTEVVQNIVKEVQKHARSEM-TQNEIALQDCPTDGISAVKFSNSSNQYLLASSWDEYVRVYDVQQDRCKHKFDHTSPVLDACFYNTTHIWSGGADKTVRLFDLHSGADLRAGTHDDAVRCVEYIPDVNQIVTGSWDGNIKLWDPRRPVGASTHAQDNKVYTLAVCGERLIVGTANRKILIWDLRNMAYVQQKRDSSLKFQTRAIRAFPDLTGYVLSSIEGRVAVEYLDPSPDVQKKKYAFKCHRTKE-NGIENIYPVNAIAFHSKYGTFATGGSDGFVNIWDGNNKKRLCQFHKFPSSIASLSFSPDGSMLAIASSFQHEYTLDPNPPPDQIFIRHHSDDDRSKMSSLENLKKLTVVVADTGDFEAMREYKPTDATTNPSLILQAAKLPQYATLIDEAVAYGKSPSQQLEEAMDKLFVLFGNEILKIVPGRVSTEVDARLSFDKEASITKALKLIGLYKELGVDKERILIKLASTWEGIEAARVLEKNHGIHCNMTLLFNFTQAVACAEAGATLISPFVGRILDWHVANTDKKSFEPLEDPGVKSVTKIYNYYKKFGHKTVVMGASFRNIGEVKALAGCDLLTISPKLLKELADSSDDVPQHLSADKAKNLDMEEVPADEKHFRWDMNEDKMANDKLSEGIRNFAADSRKLEALIQEKLKMGENVCRPLATVYAESNEASGTSAGMPAVFRAPIRPDLISFVHHQLLKNKRTPYAVSKEAGHQTSAESWGTGRAVARIPRVRGGGTHRSGQGAFGNMCRGGRMFAPTKTWRRWHRRVNVTQRRHAAASAVSASGVTALVMAKGHAVEQINELPLVVADKVQDYQKTKQAVNLLKSLKAWSDVEKVYQSKRLRPGKGKRRNRRYKKKCGPLVVYEKDNGIVRAFRNIPGVDTCNVNSLSIFKLAPGGHAGRFIIWTEAAFRKLNDIFGTFSKASKVKKAYKLPRAMMTITDIGRLQKSEEIRAAFRSAKSIIISKKEKPNPLKNPYLLDRLNPFAIVEKRSRILKLKEEAGKNLKKPRALEKMKATKRKRLSNVFPKNRKLLQGSVLKKTLDAIKDLINEGTWDCSAAGISLQAMDNSHVSLVALNLRADGFEKFRCDRNLSMGMNLSSMAKILKCAENNDVITMKAQDDADTVTFVFEANNQEKVSEFEMKLMNLDSEHLGIPDTDYSVVVKMPSSEFQRICRDLSQIGDSVQITCTKDGIRFAAAGDLGTGNISLSQTAEVDKEEEAVIIDMQEAVTLTFALKYLNSFTKATPLSGQVSLSMSADVPLVVEYDMGHLRFYLAPKIDDSEMSFARGS--IARLAA---RNFSTSQRNNVKVCVLGASGGIGQPLSLLLKQHPGISYLSLYDIAHTPGVAADLSHINTGAQVKGFVGQDQLKAALEGIQIVVIPAGVPRKPGMTRDDLFNTNAGIVRDLATACAQVCPKAMLAIISNPVNSTVPIASEAFKKAGVYDPNRIFGVTTLDVVRANTFIAEAKGLDPVSLSVPVVGGHAGVTIIPLISRASPKVDFPQDQLEKLTKRIQDAGTEVVQAKAGSGSATLSMAFAGARFVFSLVSAIKGKDVVECAYVKSDVGEAGFFSTPLLLGKNGLEKNLGLGKLSDFEAKLVAEAQDELKKSVQKGVEFANKEYEWLLKFEVNDIVEQLVIAECSKRFPL-ARSDKFVM-QIKVVATLTGDSISHADITLRL-PKHSQRTIVQNDAQWKLQQIQDAGNHLMQAMNLLKFEFISGQEVRNLMAAVMGCLGRGRACLVVPKKRTIEDIMQGRNM---VPPLPNDVAVSFYVQSYKLVFAVYHV-QKDSQKF-DAECSVPWLSEALVLFTVALQLCQQLKDKVEVFQFNDFLSMFIPVFLEYGAPLGEISDLIRVCDVCFKDAESVL--NGFVSILICGFCKCLEAAPTPKVAFN--VLYNFFEGLRVNVYMSLIKLAGKSVSEVFQDVAPLKKWLPVAEMREVYRALHKELSDMGLKVMVELLSTYSEQDATEARADAERCIAATLADPNTFLMDHLLPLKPIKALQGQPIHELLKIFIYEKVATYKEFYQHNKQLV-DGLGLDHERNVDKMRLLTFMMMAEKQREILFEDIARELDV--VDVEAFTISALKTKLVSAKINQMGRKIVVISTMHRTFERNEWEKLRETLRLDEVEQCTSQ-------------KLEDLIEESKAIDEGEERAVIEESASFQELVKILIEWINDELAPNRIL--VKSIEEDLYDGQVLHKLLETLSGARIDCVEMTQSNEGQREKLKVVLEKASQALGLKWSVDAIHSKNVVAIVHLLVSLARHFRAPVRARLPENVIVSVVSVTKR-EQLTTTYDEYGMKVE-RDAFDQLFDQAPEKLSVVKKSLLTFVNKHLSKINFEDLD-KQFHDGINLALLMGLLEGYFIPLHLSLTP----------------------EAKVNNVAFAFGLMKDAGLKPKARPEDIVNYDLKSTLRVLYNIFSQFSAGPSAVPLEVLKTAQREMLNYGCSVMELSHRSAKIIKSAEQDLRDILSIPPNYKVLFMQGGGTGQFAAVPLNLCPADYVVTGTWSQKAAKEAASFVKFDNIPPESGWKTSPDAAYLYYCDNETIHGVEFPIVCDMSSNILTRPVDVSKFGVIIAGAQKNLGPAGVTLVIVREDLIENASVCPSVLNYKITADNTSLYNTPPTYSIYLLLTLKWIKEHGGVEGMERSAKKSKALYDLIEASEGFYCRSRTTIPFRI----RCDEALEKKFLAEAEMIQLKGHRSVGGIRASMFNAMEVEQALVLYKDRSDWADVKPVINIAYSETFRDCYGYLRAVLSGELSERVFELTTTCADENPSCYTVWLLRRKLIAHLKKDLREELDFMVTQIQENQKNYQVWYHRQKMVEWLELEFIRNMLDAKNYHAWQYRQWILRKFNLWDGELAVCDEMLAKDCRNNSAWNQRYFVVLNSTGFVMDSEIEFTLDSVRYNESSWNYLRGIFSVFSLSILLDLALKADLLRANYWDFRARLLRQLFFAGIVLCVLCTLFLVCFLAVIVDRFEELIEFVPHIGRFLKAQGVAYRIIVVNQGDTYRFNRGALINIGYHVSKAQCDYLVMHDVDLLPMNSKLSYRYPQQEEVHLAAPHLHPKYHYATFVGGILMMRHETFARLDGLSNKYFGWGLEDDEFYVRIKEAEFTLERPVDIGTGINNTFKHMHDARRRPRDMARLGNQREESRRRDRVTGLHNVVYRLQSHHKMQIDGISADVFNVFLHCDVTRTPWCEKPKVSPGTTITTDGFMRGHGGLLSAVAGVVEKVNKLITVRPLKTRYNPEVGDVVVGRIVQVC--QKLWKVDVGGRLYAALHLHSVNLPGGELRRKSIEDELLMSQYLIDGDLVSAEVQNVGVDGAVSLHTRNLKYGKLGQGSLVQVSPSLVKRCKHFHNLP-NGVHLVIGLNGFLWITSSHFARESICRTRNVIISLSRYNIMLYDTTILYAYEIS-VAHLTRQGKDKLVGGWLLTCSGMAFGAVVLGGITRLTKSGLSMVDWHPFDEGRPRTQDWQREFEKYQQFPEFKITNRDMTLEQFKSIYWMEYIHRMWGRTIGAVYFIPAAAFWALGYLRGGMKQRVVLMGGLLAGQGLMGWYMVKSGLEEKPRVSNLRLAAHLGTAFVLYAFLFRSGLQLLLPTQALRKLIVSKGLVFFTVLSGALVAGIEAGLVYNSFPKMADRWIPTDVLAFPKWRNFFENPTTVQFDHRILGESVITALYLYSRKVPMPPRARFAANVVLAAWMQVALGITTLLNYVPTHLAATHQAGALTLFTTLLWLTHELKIDKK-LAKWGEGDPRWIVEERPDATNVNNWHWTEKDATQWSKDKLNALFTNLEIED-TILSVITEVSKFEGDAVVNNRKAKLIFIYDLNLELCAGS-VVKGKVEIPNLSEENDLDEIVIDVMLSDEKIKAMMRSKGVDVIRDKLSDYLNALRADYSQGLILPTKG--LNVTSTKGKKINTTNLTMEEFKCTAYELYRVFTMVQAFSQGPAGGKILMLDTNVSGKFLTLDRLEFSWRFKSWPAEHYSKVMTLDQTNDSTMIQEGVPEAELERTRAGWQRYYFDAIKRTFGFGAILMDPETFLEVANHVSKLKMYPYFEIAHCAVTLLYLREDLASGSHLFSRKHPLSCYISSMFSIFAGYMFSALLLGEPVLSAFKNNQSLILASAVWYLMFYSPFDVVYKFCKLLPVKLLLSLAKEITRAKKVHDGVHHAAKLYPNGYLIMVIIGVIKGNGTSFLKVYERLLRGYWTPNAIEIMQPSFATKICVIASIVFVVDKKTDLISAPHSLVYFGVVCFFLYFKLSSMLLGLHDPFLPFENLTCAIFFGGIWDAISRAL-----NKGGDAQSGGSKDAKDVAAKKKEMSRPDG-VRRRTPAVNAQNNAEADKRDDDKKDDDKETRMTLMEEVLLLGLKEKEGYTSFWNDCISSGLRGCILVELGLRGRIDLESAGMRRKSLLMRKVIVKNDAPVGDVILDEALKHIKETSPPDTLQNWVDYLSGETWNPLKLRYQLRNVRERLAKGLVEKGILTTEKQNFLLFDMTTHPLVDQNVKDKLIKRVQDSVLSKWANDIHRMERRQLSLLMLSHASDVLENAFNPLSDEDYEMAMRRVRDLLDMDFEAECAKSQSCDIMWGVFAAFVKMADNVPTFKCVLVGDGGTGKTTFVKRHLTGEFEKKYVATLGVEVHPIVFHTNRGAIRFNVWDTAGQEKFGGLRDGYYIQAHCAIMMFDVTARITYKNVPNWHRDLVRVCENIPIVLVGNKVDVKDRKVKAKAIVFHRKKNLQYYDISAKSNYNFEKPFLWLARKLIGDPNLEFVAMPALAPPEVQMDPEWQSKLENDMRDAQNTSLPDDDEDDLLAAQMTPVLKESRFRESGMLTPEEFVAAGDHLVATCPTWNWAKG-DKTYLPEDKQFLVTRNVPCSKRCRDMEEKVIEDG-EGWVDPYDNEDDDDGAVDMDAFLDEDPGAAGVLSTRTYDLNITYDNYYRTPRLWLTGYDEYHQPLRTKELYEDISQDFAKKTVTVEPHPHLDGPPQASVHPCKHAQAMKNLIQTVEDGGGLEVHMYLIVFLKFVQAVIPTIDYDYTTNFNMLSNLEKKLPYDKLSANIDVVKKRLNRPLTLSEKVLYSHLDQPASEEVVRGESYLKLRPDRVAMQDATAQMAMLQFISSGLPKVAVPSTIHCDHLIEAQHGGSQDLARAKDINKEVYNFLATAGAKYGVGFWKPGSGIIHQIILENYAFPGLLMIGTDSHTPNGGGLGGLCIGVGGADAVDVMAGIPWELKCPKVIGVHLTGKMSGWTSAKDVITKLAGILTVKGGTGAIVEYFGPGVQSISCTGMGTICNMGAEIGATTSVFPFNSRMADYLAATNRTAIADAASEVKDLLTADSGCKYDQVIEINLDTLEPHVNGPFTPDYAHPISQLGKVAKEKGWPLDVKVGLIGSCTNSSYEDMSRSAMLAQQALDHGLKSKSLFTVTPGSEQIRATIERDGQAKTLKQFGGMVLANACGPCIGQWDRQDAKKGDQNTIVTSYNRNFTGRNDANPQTHAFVTSPELVTALAIAGRLDFNPLQDELTAADGTKFKLQPPEGDELPRAGFDPGEDTYQGPPNDGTGVKVDVDPKSQRLQLLTPFAKWDGGDLQDLVILLKAKGKCTTDHISAAGPWLKYRGHLDNISNNMFIGAIPEESGEANKVQNRLTGEKGGVPDVARQYKAKGQGWVVIGDENYGEGSSREHAALEPRHLGGRAIIVKSFARIHETNLKKQGLLPLTFQNPSDYDKIKSDDKISIVGLKDFQPGMEARGGMFKNTFQSGFLSILYSLGSKPLQIWDKKVRNGHIKRITDNDIQSLVLEVMGSNVSTAFITCPADPRETLGIRLPFIILIIKNLKKYFTFEVQILDDKNIRRRFRASNFQSTTRVKPFICTMPMRLDEGWNQIQFNLADFTRRAYGTNYVQTLRVQIHANCRIRRVYFADRLYSEDELPAEFKLYLPVQHRTAAAALRER---------EKCLSSKYQYVHKARKDIQAASDHYKGLAETFIFNDGSSKELVCLDGTIPVRYKYYFPVRVWVLDTHPYHAPLCFVCPTPTMQIKVSRNVDESGRVYLPYLHDWNGNTGSDIVGVLKVMIMVFSETPPVFSK-PLPYPSSNTLSITDEHIRISLLSAVESRITDRALEKSKAEEEVLKKTNEELIQGKAKLQKFMSDMENDRELDSEKNEQLKLVDIDNAVTTTAPLYRQLVNAYAEESAVEDAIYYIGEGLRKEVIDLDTFLKHVRELSRKQFMLRALMQKCRQKAGLPMGRKFFVGGNWKMNGNKASIKEMCDRLKNAKFCVGVCVAVPAPYLMLCRESLPDSIRVAAQNCYKVASGAFTAELSVDMIKDCGCDTVILGHSERRNVFGEKDQLIAEKCAFALQNGLTVIACIGELLEEREGGKTEEVIFRQTKAYADLIKDWENVIIAYEPVWAIGTGKTASPKQAQEIHAKLREWLAKNVSEEVSRNTRIIYGGSVTAANCKELAQEPDVDGFLVGGASLKPEFVQIINANQMPGQISKKRRFVADGVFNAELNEFLRRELAENGYSGVEVRNGATKTDIIIMATRTQDVLGEKGRKIRELTAVVQKRFGFKEGTVNLFAEKVSARGLCAITQCESLRYKLIGGLAVRRACYSVLRCIMEAEAMGCEVVVSGKLRGQRAKSMKFVEGLMIHSGDPTNHYVETAVRHVLLKQGVLGIKVKIMHPHDPLGKRGPAQLLPDKVHVVDSPHEDDNVEISSDNKDTAI-------GGVA-PTTL----SKIEQIKQWSLSTYKCTRQILAEKMGKGIRTVDGELEANIELLRETHQKYLNILRLAKLLTSHFNTVATQAALGECFSDLAQK-------ELQQEFLYNAETQKNLSKNGETLLGALNFFVSSLSTLCNKTIEDTLITIRHYENARLEFDAYRCEAKASFRDKYERLRGDVQIKMKFLHENKVKVMHKQLLLLHNAVSAY-FSGNQSSLEATLKQFNISWLEQ-MVDLVLDRDIRIWVFLPIVLLTFLLGVVKHYVSILITTTRKPELQQVYDSQALIRVRYLRENGKFLPLKSFLMRKHYFNDEDSGWLK-TQNRPAPASNPMQDPGMMTEMLKGNLINVIPMIVIGGWINWTFSGFLTTKVPFPLTLRFKPMLQRGIELVSLDASWVSSASWYFLNLFGLRSIYALVLGEDNAADSTDAMQEQMTGAAMKI-TDPKAAFKAEWEALEVVEHKWMLANVEEDLCSKPLF----------MNMYKGAASEAGRAMQILKRRERQREEVELKKAKIEQEMKVT-MDDKFSSHFDAVEAQIKSATVGLVTLDEMKAKQENAVKEREKRLAQKEQEEKQKHEKRKRAQKEKQKKAIQALSFSMDDLDGESQDDNG-------------------------------SDSDTAETEAVEKT--RKMKKNPDVDTSFLPDREREEEERRIREELRQEWTDKQRKLKEEAIQITFSYWDGSGHRRVVEMKKGNSIYQFLQRCLETLRKDFHELRVVSADQLMYVKEDLIIQHHYTFYDFIVTKARGKSGPLFSFDAHEDVRMTSDASKEKEESHAGKVLLRSWYERNKHIFPASRWEPYDPTKCYDRYTVKDKKP-MAPA-KKPAKPTVPETLLKQRKNNAELRQQRILAVAAKKKADRARRVLAFQRAEKYVREYRQKEKAEKNNRLVAKLEGNFFVPDEPKVALVMRIRGINGVSPKPKKVMQLFRLRQINNAMFVRLNKATINMLRIAEPYLAWGYPNLRTVRDLIYKRGFARINGRRVPLVDNSIIEEKLGKYGLVCMEDLVHEIYTVGPNFKQAVNFLWHFKLNSPKGGWRKKTTHFVEGGDYGNREAFINRLVRKMISKVTAEHLWKGVTSVSNAGRKRGRASGSSRKMARDLNRGQVLGVGKINMVWPGLSAPAIRGQEIIRQQKLEPDPEREKRLTALRNK-HGVRRTKIAPLDRGWSGGKAGGRYIGPPDPVGDENFEGFKSCVVKMGMVFVMRGNTGRTRQHKSIVIVGNGNGLIGFAQGKAGDARSALRKAKNGAAKNLVYIERFENTVLHDFYTEYGSCKLIVKKKQRGYGLVCQRVVRELCKVIGIKDIFVKADARRMNTLSVIRAFLLGLHNQRSLQSVADEKRLHVVEFREEYGYFPKVVASPKDVRTAEEINPNEELDFNMIINGGRLVMMPKKKTPFYWNLPGYQIYLKKTDPLKNHRTTHLRILKRYGALKSFLTIREKQASLKSRNSDDRNRWTNRQRLLIFASRGITYRDRHLMNSFKGMLAHSKQECKFE--KKDINEIAEMKNCNRVMYFENRKKSDTYMWLANMETGPTLKFLVQNVHTMEELKFTGNCLRGSRPFLSFDPGFDEHPVVKEVLAQTFGTPAYHPKSQPFFDHVFTFRILDKRIWFRNYQVVEE-DGSLVEIGPRFCLNLVKIFDGPFSGAIIYTNPNYVAPNKARRLAKQD--NTYV-------------MAVGKNKGLSKGGKKGLKKKIVDPFTRKDWYDVKAPSMFQIRNVGKTLVNRTQGTKIASDGLKGRVYEVSQADLNNNEDAFRKFKLVCEEVQGRHCLTNFHGMDLTTDKLRSMVKKWQTLIEAQVDVRTTDGFVLRLFCIGFTKKTQSQVKKTCYAQHAQVRAIRRKMVEIIQREVSSCDLKDVVSKLIPESIGKDIEKVCNAIFPLHDVLIRKVKVLKKPKFDMSKLLELHGDSKGSSGTDGMAVDRPDDYEPPVLETV??????????????????????????????????????????????????????????????????????????????????????????????????????????????????????????????????????????????????????????????????????????????????????????????????????????????????????????????????????????????????????????????????????????????????????????????????????????????????????????????????????????????????????????????????????????????????????????????????????????????????????????????????????????????????????????????????????????????????????????????????????????????????????????????????????????RLPPLPTPAELLRLYRLKALKQMSQNFLLDPICRKLIRSAGHVIEVGPGPGNLTRPILELGA-TCSVIEKDLRFMPCLDLLAEAAE-GRLKVIHGDVLTYPIHSEI-PPELARPWESIHLIGNLPFAISTVLLVKWLKEISERSGPWQFGRVRMTLTFQKEVSTRITLMEKCRLSIISQGCEVEEGGSFVPPPLVDVGVVKLVPVKPVFQDFSLVEKVLRCMFNKTVEQDIKHDVLEKAELDPTLMLTTQEFSRLCDVYAEYCEKAGLYEYNYRGPRKMFSNRVGSLLR--SGLRLHRSVPALNPMVPIVIEQTGRGERAYDIYSRLLKERIICLMGPINDDIASLVVAQLLFLQSESSKKPIHLYINSPGGSVTAGLGIYDTMQYVLPPISTWCVGQACSAASLLLAAGEQGMRHSLPNSRIMVHQPSGGVSGQATDIQIHAEEILYLKKKVNRIYAKHTKQPIEAIDSIMERDRFMSPEQAKDFGLIDTVLEQPPAVKETRGFIRFERPVGTWLTLLPLWSLTMATPAGQLPDIALFGGGAFLMRGFGCTINDMWDKDIDKKVERTRARPLASGELSRWDALWFSAGQGMACLILLQLNWESVQLGAASVGLVVLYPLMKRFTYWPQAFLAVVFNWGVLLGFSAAALPLYAAAFSWTMVYDTIYAHQDKHDDLMIGAKSTALKFGSRTPLWLGAFTTMTTNLQTWPYYSAVTLDTSNVGLLIGCIAGTLLKVARVDRPKTRKGSRIVKAREPLTIENPKTSFVKAANINQRTAQILKDLYTLKKTESVFYQKKNPFEDVSHLEKLAKKDTSLFAFGSHNKKRPQNIVLGRTFDRMVSDQFEFGVENYKALDEFKVAKIGIMVKPVLIFAGEAWQEMKRLKNFLIDFFRGEYLGVSGLEHAISFTAMGILLRSYKIHLKRSGQKTPR-AEVEEIGPRMDLKLRRHKIASDDLFKQALRQPKGLKVKKKKNLEQDDLGTSLGRVHMERQDFRLQTRKMKGLKVTIIGALDDNYMYLITTKEAAIVDPVNPEKVLETVEVNLTTVLTTHHHWDHAGGNEKLLVYGGDHRIKQLTDRDEVEIKIGEYNDTRFTPCHTTGHVCFYIVFTGDTLFLAGCGKFFEGTAAHMQEAMLGQLPDDTLVYCGHEYTVNNLKFAQQVESDNQTQRKLEWAAHQPTIPSTIAEEKTYNPFMRTAVQKHADAVSTMDSLRCEKDNFRMNRLFGKAKAKEPPANLNDCISTVDQRANNMDEKIQKLDKELIKYKEQLSKMREGPAKNSVKQKALRLLKQKKTYESQRDNLLQQSFNMEQANFTTQQLKDTKVTVEAMKLGVKEMKNEYKKVNLNEIEDLQDDLEDMLEQANEVQETLGRSYNMPDMTDEELEAELAELNDELALDDNSYLDAVNTPAVPSKDPSAKEKPTDGIMVDEFGLPKIPQSVRHLSKSRGKATLPDLPYDYNALEPVICAEIMQLHHSKHHNAYVTNYNISSEKLQEAVSKGDVSAQIALQGAIKFNGGGHINHSIFWQNLCNPKSGEPSAELQAAITKDFGSLESLKEKVSAAAVAVQGSGWSWLAYNKATKSLQVAACANQDPLEATTGLVPLFGIDVWEHAYYIQYKNVRPDYVKAIWKVANWKDVSQRFANAKDGHIQHDVIKEALESGRDLREYSRSVDQQLKGTEDEAIKDYMENCKDIAALHNEIASCDGILQVMENILRGFQNDLGSISSEIQSLQRQSVAMNLQLKNRQAVKGELSQFVDDFIVPESTINVILDCPVTDEEFLAQLSLLDQKISFVKVQSFKEASSCQDVKDILDKLKVCAVTKIREWLLQKVFSFRKPNANFQLPQNAMLKHKLFFQFLATHEREVAKEVREEYVNTMSKVYYSYFKAYHSRLMKLQFDDVPDKDDLMGVDDTPKWGLFNKPSLKNRSTIFTLGNRNAVMSVELEAPVIVPHASAKNEKHYPFEQLFRSMQYALCDNAAREYLFISEFFLLTKSGAAEAFDSIMGKSMSMFAKYTETFVAECFDSIALFLCIHVVHKLRILMHQRNVPVLDSYWDVLVANIFPRFETILRLNINSIRDCDPSKLGSIDNRPHYITRRYAEFSAAIVSINENHPDERVSILLGQLQMEVENFILKMAAEFNGRKDQLIFLINNYDMMLGVLQQRTHEDSKETTNFRTLLTARQNEYVEQILTIHFGGMMTFIKECEFYIEKGQSEKLEKESHKVATLVRGFNSGWKKAIDDMNADFMKTFTNFKCGTNILQEALKQLLQYYHRFNKVVSQPPLNALPVRSELINIHHLMVDIKKYKATF---KSLLPLFLICLLFDGVLSYFIVVDAHAEECFHDRVAKGTKMGLTFEVVEGGFLDIDVKITGPDGKVVYNGERESSNKYTFAAYAEGMYAYCFSNAMSTMTPKTVMFSMDIGEEPKEEGKPAADAGDTKLEDMINELHTAMTGVKHEQEYMMIRDRIHRSISESTNSRVVIWAIFENLVIFAMTFGQVYYLKRIFEVRRLVMRLTRVLKLPKSWSNFPERYIKRSMEEIEYKTPVGRQYRRAVIKRPYGLDRPWTDGFKKNEPGKLQIIEPVFKGDRVQILVGKDKGKQGIVNYIVKERNWVCVSGLNCKEQPLVMFEQVALVDPTDERACKAQWRYTENNERVRISLRSGREIPIPLAEETYDYKYKNQPKDTSAADLVKITFLP-KLATFEMDIMEEHGIKDDRIPAEFYWYMADIAS--IFVDFVEKHFDSLLWISRMSTIMFTVLYFLG-PSSYYQKALMTNGVTSALRLHQRIPEVRLNVQFLARLVTEDSFHYLFYSFFFLLPVSVMVLVPPLLFAVLHSIKLLTKAGTIYL---VRVSLFQIIAMTEIMLMVILIIGVFTGMMLMAPFIYYRFLSQRYASNRNPYSRYVCRDLRVQLESAARRSCPPFISRTMFVSSRAPIAISAITVYLFFPEMEPEEKPYVTLPTSLESAKDLGRVLSNYTDDMVLLAFFCTYIFLQSFAIPGSIFLSFLSGFLFPFPLALLTVCLCSAIGASLCYLISYCVGRRLIMHYFPNMLYYIIFLRITPFLPNWLINVASPIVSVNLAPFFLGTFLGVAPPSILAIRAGISLQQLASAFTLENGLLLTGFAVLSMIPVVLRFKNKFEDLQDKLTRIAIVSTDKCKPKRCRQECKKSCPVVRMGKLCIEVTPNDKIAEISENLCIGCGICIKKCPFEAIMIINLPSNLEKDTTHRYSANSFKLHRLPTPRPGEVLGLVGTNGIGKSTALKILAGKLKPNLGRYNDPPDWTEILAYFRGSELQNYFTRILEDDLRAVIKPQYVDQIPKAVKGTVRQLLDKKDEMGKKTHLADILELNSVMDRQIGDLSGGELQRFATAMVCIQRGDIFMFDEPSSYLDVKQRLKAALAIRGQIEATKYVIVVEHDLSVLDYLSDFICCLYGTPGCYGVVTMPFSVREGINIFLDGFVPTENLRFRESSLVFKVSDNTDEDVKRICRYEYPTMVKIMGDFKLQVKGGSFTDSEIIVMLGENGTGKTTLIRMLAGKLAPDGGETVPALNISYKPQKISPKTQGTVRFLLHEKIRDAYQHPQFVADVMKPLLIDSIIDQEVQNLSGGELQRVALALALGKPADVYLIDEPSAYLDSEQRLAAAKVIKRFILHAKKTGFVVEHDFIMATYLADRVIVFEGVPSVDTVANAPQSLLVGMNRFLELLNITFRRDPNNFRPRINKLNSVKDSEQKRNGTYFFLEDEDEGEDLFGPELDRYDAAEEEMRRRDRGGMRRGLESIENLEDMKGHSIKDWVTQGPKTEIFNRFKNFLRTYKEKIRAMEQNKMSLEVTYNLAQSEQILAYFLPEAPAEVLPIFDEAAKDIVIGMFPHYERIHHEIRVRITELPILEEIRTLRKIHIDQLIRTSGVVTSTTGVLPQLRMVKYDCAKCKYVLGPFVQSQEVRPTSCPECQSTGPFILNVSQTIFQDYQRITIQEAPGKVSAGRLPRSKDAILLNDLCDSCKPGDEIEITGIYSNKFEGSLNKANGFPVFATVIIANHILRKDTDEDVKEVVKLSKEDLAERIMASIGPSIYGHDDIKRAIALSLFGGVSKNPGDKHRIRGDINVLLCGDPGTAKSQFLKYVQQIAPRAIYATGQGATAVGLTAYVSLVTRDWTLEAGALVLADKGVCLIDEFDKMNDADRTSIHEAMEQQTISIAKAGIVTSLRARCTIIAAANPIGGRYDTFHQNVNLSDPILSRFDVLCVVRDERLARFVVDSHARHHPIEQELLQKYILYAKDKIEPKLDQDKISQLYSDLRRESMVTGSMPITIRHLESIIRLAESHARMHLREHVDVNMAIRVMLDSFVTQKFSVMRMGLLSDIKEIFEVDCLYEVFDVEKTATTNDIKKAYRKKSLMCHPDKAPAEKKDEFTRKFQTLCKTYDLLQDEERRKVYDETGDV--DDAIDSNRNWDTYWRNLFPKVTLKCVDDFLKKYIGSELERKDLKKYYERFKGDMNKISQCHIGYNEDRLCSLLREMIESEEIKDYPAFSKETAASKRKRRENLEKEAEEARS------PENDELSMMILGNQRKDADKFIADLEAKYSS---KKRTKSKK-NVVLLTALVFLFLGKIGTKKLRKLEEKAEKRRLRELELQEREERKQKQAEDERRKKEDQKRDEEEKRQEELERKAKEDAERREHEEYLRMKAAFDIEEEGFDQEED---SPGRLTEFIKYIDEQKVVQLEDLAARFKLKTQDCIDRLHRMIEEESICGVIDDRGKFISITKAELEEVAKFIKLRGRVSIQELVENSNRLINLSVDILEQQEVAKSTGLKHVITMLQIPDQLDKVDQHRKRVQRKKASVEAMLKTAVQSQLDGVETGLSLLVSARDDIADCQKDEVEQIYDLAKLLQLQDVREESIKHSQTGTLMEHLKHIFNVPGSVARTQDLIQEGKFLLAHKLSDLEGSRDDLLYELHKQANNLPSDKAMLKQYFADVERLSDELGKQLWVILKRTLNTVRKEPQVIVTALRLITREEWAALRRQETT-GFLPPSRPKLWKKKAIETLEQSVAERLEANQIEGRQENKMWLVRHLEVTRQLIIDDLKTVKHHCTPCFPPSFDIFNEVRMIHNCLSQRLQTIISGLVDSEYIHILGWLNTYNSRELMQHPELNVDIALLPDTIKKLMQKYLAGLQVKFEEWLRNALTDHKDWPETDSDGYHRTEAPMLIYQMITQHIDVARTVTLISLVLNLAMEHMNNFLTSYIQLVTEYNFEDRSYTAYMIAVANNAVNMK-LKTLKENALSYLCEEVLMDIKATPNIMTDTVIVTLADYGNDYIEKQVASSYVRAIC--EKRISFKNYEERKTAAELNKLEKSELTVLKLMAEVLKMKDNSLLSLELSGMISPHHSCLIFHSRECRDPRRADAPTRVEDDIGKMAEGGRTDAEVVGREEEAIKIDSLSVSQLKEIRDKAEKQVFQAEVARMMKLIINSLYRNKEVFLRELISNASDALDKIRLLSLTNPDALKALQELSIRIMADKENNVLHITDTGIGMTKEDLVKNLGTIAKSGTAEFLQKVNDGEGSKDLNDLIGQFGVGFYSAFLVADRVAVASKNNDDDVQHVWESNASEFTVADDPRGNTLKRGTTVSLYMKDEAKDFLEHDTLKKLIEKYSQFINFNIYLWSSKT-VTEEVPEEEKDTTET----DEDDEAKVEEEKEA--PKMKKVEKTIWDWDLINSAKPIWTRKEKDVADEEYNEFYKAVTRDSQNPLARTHFTAEGELTFKSLLFVPVKQPQDSFNKYGQRTDHIKLYVRRVFITDDFQDMLPNYLSFLRGVVDSDDLPLNVSRENLQQHKLLKVIKKKLVRKALEMFRKISEEDFAKFWKEYSTNIKLGVIEDSANRSRLAKLLRFPSSIDSADKLVSLSDYVQRMKEKQSAIYYIAGGSMDEVKKSPFVERLLKRGYEVLFLTEAVDEYAISSLTEFEGKKFQNVAKEGLSIDE---NKEIREALEKEFEPLTKWLTETALKDKISKAIISERLVETPMALVASQFGWTGNMERIVSAQTHMKENDPQRQFYMSQKKTLEVNPRHPLIKELLRRVDDSPSDEMAKYFTEMMFDSATLRSGFQLSDNARFATNIEKMLRNMLGVSEEAQVDAEPEEAELPQVSKEDQEEDIESEPHDELGPRGEVKALSSEESKMIVISEIIQELVLAHNEKRDVNLNRVKCDASARHGMKSQPKLVDIIAAIPPQYKKILLPKLKAKPVRTASGIAVVAVMSKPHRCPHINYTGNICVYCPGGPDSDFEYSTQSYTGYEPTSMRAIRARYDPFLQTRHRVEQLKQLGHDVDKIEFIVMGGTFMSLPEDYRDYFIRSLHDALSGHTSSNVDEAVKYSERSKTKCIGITIETRPDYCLQRHLSDMLRYGCTRLEIGVQSVYEDVARDTNRGHTVASVCETFHMAKDSGFKVVTHMMPDLPNVDFERDILQFVELFKNPDFRMDGLKIYPTLVIRGTGLYELWKTGRYASYPPALLVDLIAQILSLVPPWVRIYRVQRDIPMPLVSSGVENGNLRELALARMKDLGLVCRDVRTREVGIQEIHNKVVPYHIELIRRDYVANGGWETFLAYEDPHQDILVGLLRLRKCSEQTFRPELMGQCSIVREHVYGSVVPVHSRDPTKFQHQGFGTLLMEEAERIAREEHNSTKIAVISGVGTRNYYRKLGYQLDGPYMSK

>Varroa_destructor

PGQRVNVLSKELMTSVVLISGKKGCFIAGADITMLEQCRSEAKNLKPIVAAIMGSCLGGGLETALACRYR-IAVEEPKTTLGLPEVMLGVLPGGGGTQRLPKLIQLPTALDMMLTGKSLHAKKAKKVGLIDAIVKPLGPGLYLEEVAARDLATGELKIRARPLTERIVRDM-IFDKARGQVMKLTNGLYPAPLKILDAVRAGLEKQNFAELCTKESKGLMGLYHGQVHCKKNAFGKPTENIAVLGAGLMGAGICQVSLKDFNRVVMKDGLVRGQNQIKKKKIQKDRLMSTLLPTLDYSDMIIEAVFEDIHVKHKVVKEVEAEHCVFASNTSALPIAKIAEVSKRPEKIIGMHYFSPVEKMQLLEVITTDKTSKDTAATAVDVGLRQGKVVIVVKDGPGFYTTRILAPMMSEAMVLLMEGCQVKELDKLKAFGFPVGAATLLDEVGIDVGAHIAEGVFGDRKEMVNNFLGRKSGKGCYIYRAVNPIQRKYTTEQVQFRLATRFMNEAVMCLQEGILANPVEGDIGAVFGLGFPPNRGGPFQFIDTYGADKIVNMRQFQEFEPCQLLLDHA-NDPLKKFHMQRPIILLQEGTESQQGKTHMMSNINACQTIGDAIRTTLGPRGMDKLMVDGKGKTVISNDGATIMKQLDVVHPAARTLVDIAKSQDSEVGDGTTSVVLLASEFLKQAKPYIEEGLHPQVIAKAYRKASKMAINKINEIAVKVDKGEMRALLEKCAMTSLSSKLVASKKQFFAKMVVDAVLQLDELLPLNMIGIKKVSGGALEDSVLVSGVAFKKTFSYAGFEMQPKQYSSPKIALLNIELELKAERDNAEIRVDNVEEYQKIVDAEWSILYDKLAKIHASGAKVVLSKLPIGDVATQYFADRDMFCAGRVVEEDLRRTMKACGGCVLTTVQDLKYSNLGSCERFEEVQIGGERYNIFKGCPNSKTVTMILRGGAEQFIDETERSLHDAIMIVRRAVKNDAVVAGGGAIEMELSKYLRDYSRTVAGKEQLLIAAFAKALEVIPRQLCDNAGFDATNILNRLRERHAKGEKWTGVDMNLEDIADNLSACVWEPAVVKMNAITAATEAACLVLSVDETIKAPQSNTDPSAGRPFAKVLNRLEAFREHEMAVTRDYISQPRMIYKTVCGVNGPLVILDQVKFPKFAEIVQLVLADGTPRTGQVLEVSGDRAVVQVFEGTSGIDAKNTVCEFTGDILRIPVSEDMLGRVFNGSGKPIDKGPPVLAEDFLDIQGQPINPWSRIYPEEMIQTGISAIDVMNSIARGQKIPIFSAAGLPHNDIAAQICRQGGLVKRPQKSVMDDNFAIVFAAMGVNMETARFFKQDFEENGSMDNVCLFLNLANDPTIERIITPRLALTTAEFLAYQCEKHVLVILTDMSSYAEALREVSAAREEVPGRRGFPGYMYTDLATIYERAGRVEGRNGSITQIPILTMPNDDITHPIPDLTGYITEGQIYVDRQLHNRQVYPPINVLPSLSRLMKSAIGEGFTRKDHADVSNQLYACYAIGKDVHAMKAVVGEEALSPEDMLYLEFLGKFEKNFISQGRYENRTIFESLDIGWNLLRIFPKEMLKRIQHSLLAEFYPRAEAKRVVTA------TGLFEDPNGFYLLKENAITQAEQLIAEAMYRKRKMVQIFDDLSDCLCRVADLAEFVKVGHPQGRYAQAAEHASLAISSLVEKLNTNRELYSALRSVIENG-DIVPTTAEQHVGRLFLFDFEQCGIHLDEERRQRVVALNDHILYVGGQFLQNSHRPRYVRQSGMPENLLVVSGLQADCSNELVREAAYRIYLYPDDHQLGLLDELLRSRHELARLCGFDTYAHRVLKGSIAETPENVTFLSYLSAELGPRAERDYQEMMGMKPWDVPYFTAYLSLASCMEGLDMIFNALYGINLEVVGELWHSSVVKLVVKNLASHMGVIYCDLFERPGKPHQDCHFTIQGGRRSDGSYQIPKVVLMLNLPPPLLTPSLMDNLFHEMGHAMHSMLARTEYQHVTGTRCATDLAEVPSILMEYFASDPRVVSKFARHYRTGELMPAEMAASLDASRVIFQASETQLQVFYAFVDHEYHSKYPLNTTEILRDVQNKHFGVKYVDNTAWQLRFGHLVGYGAKYYSYLMSRAVAATFWHRAFNADPFSRCVGTYREEVLAHGGALPPAQLIQNFLLAESLIRDIMPSDAKKKRDAKKKEALKNRNNPDATNGEDEMDEVTKKFEEDMKLNAAARAVTGVLSIHPRSRDIKIENLSITFHGWEVLQDTKLELNCCRRYGLIGLNGCGKSTLLSAIGRRELPIQECLDIYHLTRECPPSEKTALQMVLDVDKERARLEKLAEELAASDDDTSQEQLMDVYERLDAMSADTALAKASYILHGLGFTQSMMHKKCKDFSGGWRMRIALARALYVKPHILLLDEPTNHLDLDACVWLEEELKTYSRILILISHSQDFLNGVCTNIIHMNLRKLEYYGGNYDQFVITRNEMLENQMKRYNWEQAQMSHMKDYIARFGHGSAKLARQAQSKEKTLAKMVAGGLTDKVVYDKTVSFYFPSCGTIPPPVIMVQNVSFRYTDKTPFIYKNLEFGMDLDTRVALVGPNGAGKSTLLKLLCGALVPTDGIIRTHSHLKIARYHQHLHESLDVDLSALEYMMKSFPDVREKEEMRKIIGRYGLTGRQQVCPIRQLSDGQKCRVVFAWLAWQVPHMLFLDEPTNHLDMETIDALAEAINNFEGGMVLVSHDFRLISQVANEIWVCENQTVTKWRGDIKTYKQHLKNKVMKEMEKMVLADLGRRITSALRNLSTATVINQEVLDSMLKEICAALLESDINVRLVKQLRENVKAAIDIDEMAVGLNRRKVVQSAVFKELVKLVDPGVRAWQPSKGRSNVIMFVGLQGSGKTTTCTKLAYYYMKKGWKTALVCADTFRAGAFDQLKQNATKARIPFYGSYTEVDPVVIAADGVSKFKAEHFEIIIVDTSGRHKQEDSLFEEMLEVSNAVSPDNVIFVMDASIGQACELQARAFKEKVDVASVIITKLDGHAKGGGALSAVAATRSPVIFIGTGEHIDDFEPFRVKPFIQKLLGLGDIEGLIDKVNELKLDENHELIEKLKHGEFTLRDMYEQFQNIMKMGPFNQIMGMIPGFSADFMSKGNEQESMARLKRLMTMMDSMTDEELDDREGAKLFARQQTRITRVARGSGCSTFEVHELLNQYTKFAAMVKKMGGMKGLFKGNDLARNVNPAQMNKLSAEMAKMIDPRVLQQMGGFSGIQNMMRQMNASSLMQDGTFIKLKKLYSMKKGTLNINSLFENDPSRAEKYTIKLADGDETLLIDYSKNLIDDEILVNLVELAKNREVELMRAKLFSGEKINFTENRSVLHVALRNRSNRPITADGEDVMPKVNAVLEHMKAFCQQVISGEWKGYTGKKITDVVNIGIGGSDLGPLMVTEALKPFQVGPRVHFVSNVDGTHLFETLKKVDSETTLFIIASKTFTTQETITNAESTKQWFLDKAGDKAHVSKHFVALSTNKPKVEAFGIDAANMFEFWDWVGGRYSLWSAIGLPIALFIGMPNFEKLLAGAHFMDEHFRTTPLDKNVPVILAMVGVWYINFFGAESHCLLPYDQYLHRFAAYFQQVDMESNGKYVQRNGERVDYQTGPILWGEPGTNGQHAFYQLIHQGNRLIPCDFIAPVKTHNPIRGGVHHKILLANFLAQTEALMKGKSEQEAKSELKASGLSEEVLERILPHKVFLGNRPTNSIIVQQVTPFTLGALIAMYEHKIFVQGVIWNINSYDQWGVELGKQLAKRIELELNGKDPVSSHDPSTNQLINFINSYNTNVSYQDRDKPAQVRQSNITASKAVCDAVRTSLGPRGMDKMIQAVSGDVTITNDGATILQQMQVLHPAAKMLVELSKAQDIEAGDGTTSVVVIAGSLLDAASKLLLRGIHPTIISEAFQAAAKECVDILSCLAIPIELSDRESLLQSATTSLCSKVVSQHSDVLAPMAVDAVLKVIDPNNVDLRDIKIIKKLGGTVEDTELIDGLVFTEKLAGGNSPHRVEKAKIGLIQFCISPPKPNMDHQVIVSDYTVMDRVLREERAYLLNIVKVVKKAGCNVLLIQKSILRDAVSDLALHFLAKMKIMVIKDIERDDIEFISKSLGCRPIASLDHFVPEALGSAELVEEVTSAKYVKVTGVANP---KTVSLLLRGSNKLVLEEADRSIHDALCVVRCLVKKRALVPGGGAPEIELSLRLAERAREIEGLHSYCYRAFADALEIIPYTLAENAGLNPIQTVTELRNRHAQDKRTYGINVRRGCVTDILEENVLQPLLVSTSAITLAAECVRSILKIDDIVQTVR?????????????????????????????????????????????????????????????????????????????????????????????????????????????????????????????????????????????????????????????????????????????????????????????????????????????????????????????????????????????????????????????????????????????????????????????????????????????????????????????????????????????????????????????????????????????????????????????????????????????????????????????????-------MSDAEEDYELEYSEDDDSQPDVDLENQYYNSKALKEDDPQAALQSFQKVLDLEGGQKGDYGFKALKQMVKINFQLGEFKEMMSRYKQLLTYIRTAVTRNYSEKSINSILDYISTSKKMDLLQEFYEVTLEALRDAKNDRLWFKTNTKLGKLYLDREEWNRLARILRQLHLSCQNVDGSDDLRKGTQLLEIYALEIQMYTSQKNNKELKKLYEASLQIKSAIAHPLIMGVIRECGGKMHLREGEYNSAHTDFFEAFKNYDESGSPRRTTCLKYLVLASMLMQKEINVLDSQEAKPYKDDPEIVALTDLVDAYQAHDISRFESIVSPHKESIMKDAFIKEHIEQLLLNIRRQVLIRLIRPYTRITISFISRELNIPSAEVESLLVSCILDNTINGRIDQVKQVLELNP-SPTDRRYTAMEKWAAQVQLIQSTVVRKVAQLGTLNPVQILNQQAEEEKAENARLSSFVGAIAIGDLLKSTLGPKGMDKILLCETSRDSKVEVTNDGATILKAIGIDNPAAKVLVDISKTQDDEVGDGTTSVAVLAAQLLQEAEKLVGMRLHPQTIIAGWRKAVVAARAALEEFSQNRSNDEAQFRIDVLNIARTTLGSKILSQHKDFFAQLAVDAVMRLKGKSNLDAIHIIKKLGGSMLDSHLEQGFLLDKKPGLNQPKRVEKAQILIANTPMDSDKIKAGKLRDECFSRVVVAELEDAEKLKMKRKVDAILAHKCNVFINRQLIYNYPEQLFADAGVMAIEHADFDGIERLALVTGGEIVSTFTSPESVRLGTCDVIEEVMIGEDKLLKFSGVPLGEACTIVLRGATQQILDEAERSLHDALCVLAFVVKEKKICYGGGSAEMLMAAAVDSVAQTTPGKEALAIEAFARALRQLPTIIADNAGLDSAQLVSELRAAHANGQSTFGINIADAKIDDMEKLGVTEAFVVKRQVLLSASEAAEMILRVDSIIKDAPRKRVPDRSHMAASIKPSGKPQLLCRLDGHTDTVNQVVLIGDADAVISVSDDRTIRVWARRDTGQYWPSVCHTMPSLASAMDYDPQLRRLFVAMDNGSITEFELADDLNKITYRRSYIAHQQRVTSMKFSPVTEWLISAGKDKYFQWHCTETGRRLGAFLGSAWCTTVALDQASRHAFVGDYSGEITMLKLTETSYQPVTTLKGHSGSVQSLLWDERRRLLISGGFDQIIIVWDIGGGKGTAYELSGHRARITGLALYSASSLLSVSEDSTLVVWDVAAQRQETPEWTTRDCCERCARPFFWNLRARTSNSDVGSRQHHCRRCGRALCDACSENRSTLPRLGFEFPVRICNECHLHISDGDREPLAKFHDLKAPVTAMSLSAESKTMVTIGTDRSIKIWDLSKVLMGDQVKEGTASKKALKKAQKEAEKAARKAAHK---AAGKENGSDDGSQGLYGQYPMIQSTEKLQREMIEISQCTLERADQMVWLRGRLHTSRAKSKQCFFVLRQQHYTLQCLLDVSEGTSKQMLKFISAVPKETIVDVEGKLVKSPLKIESCSQQEVELRIYQFWIVSLSDTRLPLQVEDASRPEPAEGDEEALKIRVNQDTRLDHRVLDLRTPANQAIFRLQAGVCHLFRESLNRRHFIEIHTPKIISAASEGGANVFEVTYFKGKAYLAQSPQLYKQMAIAADFDRVYTIGAVFRAEDSNTHRHLTEFVGLDLEMAFKYHYHEVLDTIAEMFVDIFKGLRDRYQPEIDTINKQYPSEPFKFLEPSLRLEYSEGVAMLRAAGVEMADDEDLSTPNEKLLGRLVKAKYDTDFYVLDKYPLAVRPFYTMPDPNNQMLSNSYDIFMRGEEIMSGAQRIHDPEYLTQRAKAHGIDISTIQAYIDSFRYGAPPHAGGGIGLERVVMLYFGLNNIRKTSMYPRDPKRLTPAMEEGLQLNEINPLEFSRVKMVYPKECRIRRISYRGKLDLTLWWMNGIKQEPIKRTCGEIPIMVKSLKCNLYGLDPEQLVERGEEMEEFGGYFVVNGNEKVIRLLIMQRRNYPIAMARNGWKNRGSMFSEFGVSLRSCRRDAQNMVLHYLTNGTVQVMITYRKEVYFVPAVLLLKALVNKSDYDIYRTLTQGCENDSFYKGCITNMLRLVQENILTQDAKEFIGDKFRWSSNADVCDQLLKRCVCVHLDSNEDKFNLMCFMVRKLFAVAKNKCALESADSTMNQELLLPGHIYLHILKEKIEGLLVGVKISIDKKMSNALDVTNAMNYFLSTGNLVSKTGLSLQQTSGFTILAEKLNFWRYLAHFRCVHRGSFFMEMRTTTVRKLLPEAWGFICPVHTPDGGPCGLLLHLSAMCEVVVQLDGRCIGWVLRYLKATGNIPPTLEICVIPRTEQNSLFPGLYIFSTPARMMRPVLNRTQTVEWIGTLEQVHMDICVIAEEATTHQELRETAMLSVLANLIPWPDFNQSPRNMYQCQMGKQTMGSPMHTFRYRADNKLYRLYPQSALVRPTSYDHFQMDEYPSGTNAVVAVISYTGYDMEDAIVLNKMSVERGFKAGVYKTETINLRVDVDGLPYIGSQGDPVCAYIQLKTVRYYSTEPAIVHEVKILGNLQQIQLTYLIRTPMIGDKFASRAGQKGICSALWPTESMPFTDSGMVPDIIFNPHGFPSRMTIGMVVESMAGKSAALHGYVHDASPFKFSEEYPSSAYFGELLQRAGYNYYGTERMYSGVDGREMEADIFFGVIYYQRLRHMVADKYQVRTTGPVDSLTRQPVKGRKRGGGIRFGEMERDSLLAHGTAFLLHDRLFNCSDKTLCATCGSIISIPIPYVFRYLAAE-MVSINMKIIRDTSRDLHRHQRNAPELHPMTVVREYTAAVNAAKLDRIFAKPFLCSLGHRDAVELLAKHPDRISGAVSASADGELRWWDLSNRKCVRALQAHDGPIRGLVGQDQTIKTILSKLAHHYNNMFATAGETVSLWEERNEPLRSFW-GVDTIYVIFSPIESLSSDRSIVLYDIREASPLRKVILEMRSNALAFNPMQAMHFTVANENYNLYTFDMRHLKKALQSHTDHVGAVLSVDYSPTGTEFVSGSYDKSVRIYRSREVYHTKRMQRVTSVMYSLDSKYILSASDEMNIRLWKAKASEQLGIQNYNETLLQRFQHHPQVKRIVRHRHIPKTLYQEKQTMVTARKRKMGMQFLQRGAYQDALSHYHAAIEGDDTNYQSFYWRATVYLALGKSKLAVEDLNRVIELKDDFLKAREQRGNILLKQGHLDEAHIDYEFVLRLEPDNPEAMIEELKNDVIHILQRVWNLKLRELRASCYESIGDIQSAITDLRPAIRSVPDNTGGYLHLAQLYYKHGDPDDSLTTIRECLKLDPDHKECYKNVKKLAKSMQEECVEKMVGLIKTKCQCASKGGSAVQICTEALALDPILCDRGEAYINQDDFKQDFAAARELDQRAGEGLKRAQKLEKSRGKRDYYKILASKREIAKAYRKLAAEWHPDQY-QGID--KKNAEKKFIDIAAAKEVLTDPDKRAKFDRGEDPLDPESGFHYTFPFMLVLFESPAGYAVFKVLDEKKVQKTDNLFKEFEDASGAAKILKLKHFQKFQDMTQALSAATAAIEGKLCKPLKKVLKKLAVSDAHETLAVADAKLGNIIKEKMDILCVANSSIQELMRCIRSQQEALITGLSQREATAMALGLAHSLSRYKLKFSPDKVDTMIIQAVSLLDDLDKELNNYVMRCKEWYGWHFPEMSKVVTDNMLYVKTVRKMGMRSNAINLDLSDILPEDQEAKIKELAEVSMGTEIAPDDVANIMHLCDEVIQMTEYRGTLYEYLKNRMTAVAPNLTVLVGELVGARLIAHAGSLLNLSKQPASTVQILGAEKALFRALKTKHDTPKYGLIYHAQMVGQSSQKCKGKASRWLAAKSALAIRVDALGEDTDTEMSLRNRANLEARLKMLEDGKLTRISKTGSKSNKFDVYRHKSEILEYKPAKDSTL--GKKRKFADDDDDAHEPKQFKSK------HSD--VQQEQEEDQ-KVEQGSEEQ-----------SPVEKKKKKKKKSK------VAEEQEEVE------------------LFLWPKNSTKLQFSIFVCFGIMIVGRVCTPIAPIMQKKIVDGLVSSTGLLL---LFQGANSLMANVRSYLWLGVQQYTTKATQVSLYAHLHSLSISWHLSRKTGEVLKVLDRGTSGVQNLCSYLLFQIFPALTDIVIAFGYFTYAFNWFALVAFVCMGSIVLTEWRTKFRHEMNHLENAAYAKSVDALLNFETVKYYNAEEIEIAKYQNAEWKSQSSLALLNIIQGSTSSIIGALLCAYITAGDYVLFIAYNAQLYAPLTFLGTYYRMIQQSFTDMENMFELLDVVDHHLKL--EIEFRDVCFRTVLKHISFVVPHKHTVALVGHTGSGKSTILRLLLRFYDVQSGSILIDGQNISAVSLRSHIGVVPQDTVLFNMSIRENIRYGRPSEDVEAAAAAADLHHSIFQMYETVVGERGLKLSGGEKQRVAIARTILKAPSIIVLDEATSALDTQTERNVQKALNVLENRTSIVIAHRLSTIINANQIIVLEHGEIVEQGHEELLSKKYASMWRQQQAFLLRRQLTHLTRTFSQSLLNVPETRVTTLSNAVRVASEDTGAPTATVGIWIDAGSRYETEKTNGVAHFLEHMAFKGTGKRSQTDLELEVENAGMHLNAYTSREQTVYYAKCLKKDLARAVDIIADITQNPKLGEQEIERERGVILREMEEVEGNLQEVVFDHLHSIAYQGTPLGLTILGPTENIKSLQRQDLKDYIDTHYKGSRIVLAGAGGVDHDELVKIAEQTFGKVSNSMDSQAPCRYTGSDIRVRDDDMPFAHIAIAVEGAGWANADNIPLMVANTMIGSWDRSHGGGANASSRLAAWAQSVKSMHSFQSFNTCYKDTGLWGLYFVADGDELDDIMIAVQEEWMRICTEATDGDVTRAKNLLKTNLLLQLDGTTPLCEDIGRQMLCYGRRIPLHELEARIDAIDADTIRNVCQTYIYDRCPVVAAVGPVEGLTEYTRIRGQMYKHSVHTLVFRSLKRSHDMFICEEGALPPIDEKAHKLRVGTKGRDEYGPVMHLVSEGRRSVSSGPLVLAGQQLTAQLKKPSIPKPTWHPPWKLYRVISGHTGWVRCVAFDPTNEWFCTGSNDRIIKIWDLASGKLKLSLTGHISGVRGLAVSQHHPYLFSCGEDKQVKCWDLEQNKVIRHYHGHLSGVYTIGLHPTIDVIITGGRDSTARVWDMRTKANIHVLSGHTNTVASVLVQATEPQVVSGSHDSTIRLWDIVAGKTRVTLTHHKKSVRALVLHPKLNMFASGAPDNIKQWMCPDGKFIQNLSGHNTIVNCLAMNEDGVLVSGGDNGSLQFWDWKTGYNFQKLTTPVQPGSIDSEAGIFAMSFDLSGTRLVTCEADKTIKIFKEDETATEETHPINWRPEIVTGSTSEYLINCSAEHYLEILGINVKAKNFLVFQGAVESIAMKNPKERTVLFEEISRSMISAEEETYQKKKGIAAERKEAQIEEAEKYQKLKEDVQVNLHLFRLFHQEEELQKKRRKDKIEAELKEKKKVEQTHRDDVELNKRKPAYIKAKEKTAHMQKKLDAAKKSLEAATKTHKSHQGIEELEHELSQVEEAYEQEDVSLEESQVKEYNRLKEKAGKMASAALQEYDSVAREQKTDQDHLDNELRKRNECEAKLKELEENQRRINKLVDLQDLKEEEKKLEAKKRLTQKFEDVASLGDAKVDKHEDARRKRKSEIVEHFKKLYPGVHDRLVNLCHPIHKKYNVALTKVLGRNMEAIVVDTEKTGRACIQYLKEQMLEAETFLPLDYIDFKPLKERLREFKDVPNVKLLYDVLKYEPLSIKKAVLYATNNALVCETAEDAAKVAFQSPDGKRYDAVALDGTYYQKNGFISGGSSDLAKRAKRWDDKDFHKLKDQKEKLQEDLREAMKTARKESDLTTIESQIKGLETRIKYSKVKQLEQSMREREGRINEIKARQNTVEDDVFRDFCEQIGVANIREYEERELHASQEREQRAELENQKNRIASHLEYER--TKDTLAVEQDQQELGRLKEIEQKQKELIEQQMEAISTLKNERQSKKIRVDEIDEEVAEIRKRLTAQQKEVTGVQKTVTQAEAKLEQKRSERHTLLQSCKLEGIRIPLIRGSMSQMYAREAMEIDYNQLRMEKEINLQRIQAPNFKAMEKLDSVKERLKDTDTEFEHARRKAKSNFELVKRERTCFEHVSNCIDEIYKSLTNNPSAQAFLGPENPEEPYLEGINYNCVAPGKRFQPMSNLSGGEKTVAALALLFAIHSYQPAPFFVLDEIDAALDNTNIGKVARFIREKTQTSFQCIVISLKEEFYGHADCLVGICPDPGECTISRIYTIDLSMSEAMLLYLGQAIREGNLAEMQNIYERSLVNLMDQYLIIIYKE--LYYRQIYADDRFSSYYNYVNLFNYILSSGPVSLDLPNQWLWEIMDDFVYQF--QSFSTFMWNVHSVLNVLHSLVQKSNINQQLEVFSKGGNPDEVAGEYGSRPLYKMLGYFSLIALLRLHSQLGDYYQAIKVLQHLELNRKGLSRVPACQVSTYYYVGFAYMMMRRYEDAIRTFSDVLVYIGRTKRNYQLRQMNNQMEKMYALLSICMVLHPQRMDESVLQQLKLKHMMKMSQGDLDTFERCPRFLSPVQWRAFRGEVVAQMNMRSFLKLYTTMPVEKLTKFVLMCFKHLMSNVVLDGEFQTGSDMDFFIDKDMIHIADTKLARKYGEFFAKQYNRFEEWYEMAALRNGSLLIPRLVQALRERNQSFFAHVEMGPPDAILGVTEAYKKDPNPKKMNLGVGAYRDDDGKPFVLPSVRAAERQLMSKNLDKEYLPIGGLNDFCKNAAILALGDNSAVIKEDRNATVQGISGTGSLRIGAMFLDEFLKGNKTVYMPNPTWGNHIPLFKRCNFQVKQYRYYDPKTCGLDFQGALEDISSIPEGSVILLHACAHNPTGVDPRPEQWTEIEKVVRERNLFPFLDMAYQGFATGDIDRDASAVRLFAGSG-PMCLAQSFAKNMGLYGERVGAFSLICSSAEEQARCMSQIKILIRPLYSNPAVNGARIANLILSDPQLRAQWLKDVKGMADRIITMRSRLRSGLKREGSTHDWKHITEQIGMFCFTGMTAEQVTRLIKDYSVYLTKDGRISVAGISSHNVDYLARAMHEVTKHIRPSFYHALHCILMAVLMYVFVEFSKPPDQVDPFSEYGVIFTIILYLFRLLPLLALPQSLTNLFGLTLYNAFPPRVRLKVKPHEAPFLCIRVVTRGDYPGLVRENVKRNLATCLDTGIDNFVIEVVTDKEVYVTANSKIRQTVVPKSYNTTTGAMFKARALQYCLEDNVNLLADGDYILHLDEETLLTKDALRGVLNFISAGRCSFGQGLITYANERIVNWFTTSADMYRVADDLGKLRFQFNFFHKPLFSWKGSYVCTRVGAEREVSFDHGPDGSVAEDCYFSMVAFSKGYSFEFIEGALWEKSPFTISDLIQQRKRWMQGIYLVVHSAKIPWRYKVWLSCSLYAWATMPLSTSNLVLAPNFPLPCPQTFNIICAFIGALNIYMYIFGLIKSFSISRYGFFGFWLCFMLVVIAIPLNIVVENVAVVWGLLGNKHKFYIVDKNIVMETQTEGCGEPAKLQCPTCLKLGIKGSYFCNQVCFKGSWNTHKAIHKTAKNSPYNPWPHYHFSGKLRPGRISTKRTVPAHIKRPDYADHPEGIPVSEQAMKGA-EIKVLNEAEQEAVRKASLLARECLDVALAAAKPGVTTDELDRLVHEAAIARNCYPSPLNYYKFPKSCCTSVNEVICHGIPDDRPLKDGDILNVDVTVYHNGYHGDLNETIFIGKVDEAAKKLVRVTYESLQKAIECCRPGVLYREIGKIIQKHVQQNGFSVVKSYCGHGIHSLFHTAPSVPHYAKNKAVGVMKAGHCFTIEPMISEGVWQDEVWPDNWTAVTTDGKRSAQFEQTLLVTDTGVDILTRRRKKNGQPWFMDQSTLRECLKERSAKRRELLARQLGAGCAENLGLLLGNDKTTATEQGVTLDDEEVMAYRDSSTFLKGTQSANPHNDYCQHFVDTGQRPQNFIRDVGIQDRFEEYPKLKELIKLKDELIHETATPPMYLKCDLLQYNLRELNGKFDVILIEPPLEEYQRSCGVTNTRFWSWEEIMKLEIEEVAAPRSFVFLWCGSSDGLDLGRQCLRKWGFRRCEDICWIKTNINNSKVKNVEPRAVFQRTKEHCLMGIKGTVRRSTDGDFIHANVDIDLIISEEPPFGMMEKPEEIFHIIEHFCLGRRRLHLFGRDLTIRPGWLTLGPELTNSNLNTEAYNAHFNTANDYLTGCTERIEALRPKSPPPKSALGGPSRGQRGRTSLNKLNLLALDVAGKRVVMRVDFNVPLKDGKITNNQRILAALPSIKYCLDKGAKSVVLMSHLGRPDGQSNSKYSLAVVAEELNQLLGKKVIFLNDCCGAQIEAACADPTPGSVILLENLRFHIEEEGKGVDTAGNKIKADASKVKEFRASLTRLGDVYVNDAFGTAHRAHSSMVGVELPRRAAGFLMKKELDYFSKALDQPARPFLAILGGAKVKDKIQLIENLLDKVNEMIIGGGMAYTFLKVTRGMKIGDSLFDDDGAAIVEKLMAKAASNNVQIHLPSDFVIADKFHEDATTGTADISNGIPDGWMGLDCGPKSVELFAGAVSRAKTILWNGPAGVFEFEKFAVGTKGLMDVVVAATDRGAVTIICGGDTATCAAKWGTEDKVSHVSTGGGASLELLEGKILPGVAALSDAMRTFGDRPVSFQLEDGGDYYYIGTEVGNYLRLFRGTLYKKYPSLWRRAVTVDERKKISQMNMSQHSAANFISLLKKSEVDDLIDGNEEKYRAAPVQENEGGGHGAKNARPSFMPAAPNNAHHLDAVPCSTPINRNRLQHKKNKSFPMLYDDLDPAMLHESAALPECLVPIRLDMEIEGSKLRDTFTWNRHEAHISPEQFAELLCDDLDLPPLLFVPQIAASMRQQIEAFPSESLLDEQTDQRVLIKLNIHVGNISLVDQFEWDMSERANSPEEFATKLCSDLGLGGEFVTAIAYSIRGQLAWHQRTYAFSEAPLAQLEMPFRAQSEAEQWCSFICAIPKQQKPHIIRNFQRLFRRNNRSADCS-MGANGSTLDRDDALPSSERYLGLVNFGNTCYCNSVLQALYYCKPFREKVLEYKAKNKRTRETLLTCLADLFHNIHSHKKKTGTLAPKKFIARLRKDNEVFDNYLQQDAHEFLNYLLNTIGDLLQAESSWVHDIFQGTLVNETRCLTCETVSSKDEDFLDLSVDISPNTSISHCLRGFSSTETLRGEHKYHCEQCNSKQEAQKSLKVKKLPPILALHLKRFKYTEQQNRNTKLSWRVVFPLELRLFNTSDDALNGDRLYDLVAIVVHCGTGPNRGHYISIVKSHGLWLLFDDDMVDKIDPSTIDDFFGLTQDTPKSSESGYILFYQSKENADIDNWIELAKQCKYLPEADLKKLCNMVCQILIEENNVQPVSSPVTVCGDIHGQFYDLEELFRCGGHVPDTNYVFMGDFVDRGYYSLETFTRLLTLKAKYPKKMTLLRGNHESRQITQVYGFYDECQQKYGNANAWKYCCKVFDLLTLAAIIDGEIFCVHGGLSPEIKALDQIRTIQRNQEIPHKGAFCDLVWSDPDEVETWSCSPRGAGWLFGAKATHEFMTYNSLSLICRAHQLVHEGYKYMFEDKLVTVWSAPNYCYRCGNVAAVLEISNDQKKNPKIFNAVPDHERVIPERHAPYFLVDSASPEETQDAEAATIDELKEHTAHIQKAVAQKESRFILRILRLLPATRKKLNSKLLRKTINGFYTHDKMHREVLLSFVDAEDADTDAAQKSAHLALLPEVDVYLHLLLLVHMIDAANMERAIRCAELLKGKVEAHSRRSMDLLAAKTYFYYSRVYELDGNLSSIRGFLLKRLRTATLRSDFEGQAVLINCLMRNYLHYSLFKQAAKLVSKVTFPEMASNNEWARYLYYLGCIKAIQLYYTDAHKNLLQAIRKAPQHSALGFKQTVYKLAVTVELLLGDIPDRTTFRQPALRKSLAPYFQLTQAVRTGNLGLFNQVLESYGARFQADHTYTLIIRLRHNVIKTGVRMINLSYQRISLADVAAKLQLGSAEDAEFIVAKAIRDGVIEATIDHDKGYVQSAENIDVYCTGEPQSQFDQRISFCLDIHNQSIKAMRFPPKSYNKDLESAEERREREQQDMEYAKEEDDDTFQVAVRLTLALAAMAARTLWQSAVTDMIENFRDSQPLLFEFLARLPEEATAGYIYYTSVLSLCQSALASNLRITAMRTVPANLCITLL-DLTDDHACDAILSFLQHPDGHYPKLMGDLLEQVVKCGPIVETKRACGDQDSIYSLLTGMGEMHTNLILASLLPDSSNRTEMLLKMLLDCVGTPGQYPSEEIISRIPITFWHILLDELARVEPSTQMAKQLQPVYEQLVKMLRKSQLPDPGTMDLDEKEDLRCYRQDIAD--CYMYIATMLPAPVFYFFISALDTAKNAKVIEACLFALNAIGDMADSEDDSPVVGAVLALLPRIPAGDEVLSQVMTAVGIFAEENIGPLVHLLLRGLQQTSASASMALKDLARTHGDRLAPAANDILQAIAVLKHRDRVRLVAIVGHVVSALSSEQALTSLSALMAPFVQQLNEITNLVEQLMPLFKLIAAKY-SCDAEVVSNLAECIRKAVPVLELEVVLSELLSMCGALLLGAATSVDLHGCTDAVESFYKLAVLFKKFSLDDLIQLCPEQFTYRALTQFVNASERSPVKEALENIIAQLVQNIRVSRNFIEAEADVFLALNKTAAALQRFVSSPESRQNFARQILRERSNKRMLCKVI--IDFTLKVKVKWKEVY-DVEVHLPEVFRAQLFALTGVLPERQKVMFKGAILKDS-WGATILMMGTKEELPQPTEKTVFMEDMDDSEISTALKLPTGLNNLGNTCYMNAVVQCFKTVPELTDLAKFTGTITGALRDLYRSMESYSTAPIVLLQALHTMFPRFAEKGEHGQQDANECWTEMMRMLQNLIDQLFGGKLVALQCTESEEESTEDFLQLSCFIS-NEVKYLIAGLKLRM-QETITKMSPTLNRDASYKTSKISRLPAYLTINLVRFYYKERESVNAKILKNVVFPMMLDVYELCSTDLQQKLSPQREKFKKWDNEFSFSEDGSNNSGFYQLQAVLTHKGRSSSSGHYVGWVRREWFKCDDDVVVSEEEILKLSGGGDWHVAYVLLYGPRVLAGLINEKLPKELLLKIFSFLDIVSLCRCAQVSKEWNVLAMDGSNWQNIDLFSFQRDVTYDVVSYIAQRCGGFLRRISLRGCQNVPDQALSVFAQYCHNIEQVLTNCHKLSDDSVVSLSMSLHVDSCVELTDRSLRFF----NRLRVIDISWCRKITGQGIGTIAG--DQLLRFTAKGCLDNEAIIKLATKLQVLNLQCCSFLTDSAVIAVAQNCPDLRHLCVSGCSLLTDASPQALAGGCLHTLEMANCQRCGDAGLAPLLKACHDLRRLDLEECNLITDSTLNHVAAFCPLMEQLTLSHCDQITDQGVHKLAVQCIEIDNCPFISDTSLEYLADHLRRVELYDCQLITQDAIGKFQPEVRLHTYFAPATPRQRYCRCCVIVSSKAARVFCPDHPGANLIEDYRAGDMICPQCGLVVGDRIVDVGTEWRVFQNEKSSNDPTRVGAAENPLLGGSDLSTIIGRTGDASDESGNAKYANRKTMSASDRALIGAFREISAMGDRINLPKTIMDRSNLLFKQVHDGRSLKGRSNDAIASACLYIACRQEGVPRTFKEICAVSKVSKKEIGRCFKLILKALETSVELITTGDFMSRFCSNLALPPSVQKAATHIARKAVEMDIVAGRSPISVAAAAIYMASQASAEKKSQKDIGDIAGVAEVTIRQSYKQMYPKAAQLFPEDFKPANESPWVEKYRPEKFTEIVGNEETVARLEVFSRQGNMPNIILCGPPGVGKTTTILCLARLLLGNSFKDAVLELNASNDRGIDVVRNKIKMFAQTKI---------IILDEADSMTEGAQQALRRTMENYSKTTRFALACNTSDKIIEPIQSRCAVVRFGKLNDAQILAKVIDVCRKENISYTEDGLEAIVYTAQGDMRQAIGNLQSTHVGFGHVNAKNVFKVCDEPHPLIIKEIIEFCSKGDIDEAYTRMKTLYSLGYAAEDIISNMFRVTKSHGLAEFVKLEFVKQIGLTHMTILQGLGSLLQLSSLLANLCLIVQDKKYMKKEHSLVKPYQGSGMNMPNWDFTGTTMVSSNYIRLTRDSQSQQGSIWNKVPWEIQIQFKVYGSGKDLYGDGFAIWYTKDPLQPGPVFGSRDFQGLGIFLDTYANQNGHHNHGHPYISAMVNNGSLSYDHDRDGTHTELAGCEAKFRNSEYDTSVSIRYEHDTLVVSTDIMGKKEWKECFRVSGVRLPTKYHFGVSAATGDLSDNHDVIGIKVFELDEAREFILPQAAPHRDHIDDAMSGTKFFFVVLFSMLFLMFYFYQKHQENARKRFYRDRDAFDEIRDKNCRIQDINELFLPNDTVTHVPNIKQLNIDPVFPNRTNLLHIHNMAISKAFFFSFILQRAKDDEPGFMYYFMSVIADVAANRFINASAIYYAPNMSFTPSYKGFFNKTMPLFAPRAFRSDDFNDPYHLEGTSTLNTIEAIDLGAISLNYSSDQYRINEWYSAWLPDLTKRQDSKTTYTVQITGNNDTFVWHGPPAGNDNPGPVKWVRPYFDCTRSDKWVYGATSPIPDIYPRHTQWRHIEIPRYVAVSVMELDFERIDINQCPFGPGN-PRPNYFAGTSRCKNDTTDCEPVHGYGFRRGGYQCRCKPGFRRPRVVRNPYHGELIERASKHEYENGYQCDKIGYIAVLTQTLNNYMAIGTRIDPLLGGDVVYGKEVQLENEARMAVRLANFVSGFLQIVDPKDLFAEFRVPDKSLTADQMIGEVMSIVIGDQKVVGAGIYFDYKAFFGPY--AWRKGRNERKYFVDDTTKIRIRYNSTGIKYDHFPLQYKAADVGYWTSPYFDCGGYHNSWMVTYAAPFFGWDSLRARLQFKGVVAVIELEQLEINQCNAFENTHKCDRRSSRCVPILGRGFQGGYKCECNQGYEYPYNDPITYFDGQIVEAPSRFERMTHVSGTDKSKYGGRFMVTALPGDGIGPELIGYVKEVFRYGGVPVDFEEVHLDSSRDDVDLLEQAIIAVKRNGVALKGNIETRHNDPNCKSRNVELRLRLGLFANIVHVTSQPGIETRHSGIDIVLIRQNTEGEYSCEEHMSIKGVVESLKVITQSKSDEIARYAFEWAKNNGRKKITCVHKANIMKLSDGLFLRCCTEISKEYPELEFDNIIIDNCSMQLVSNPKQFDVLLLPNLYGNILTNLACGITGGPGIASGRNYGREYAVFETGTRNTGKSIAGKNIANPIAMMNAGVDLLYHLNLREHAEVIATAIDKTINVDKIHTPDLGGQATTTDVVQNIIKEVQKHAMTYSGSSNEVILRDPPTDGISAVKFGQTSNQFLVASSWDGFIRLYDIQGERCRAKFDLGGPVLDTCFQGSSHVWSAGVHRSVRLFDINQGTELKAGSHEDTVRCIEYASDVSQIVSGGWDGAVKLWDPRKPIGPSSHSQDNKVYAIAIAGERIIVGTANRKVLIWDLRNMAFVLQKRDSSLKFQTRAIKAFPDKTGYVLSSIEGRVAVEYLDPSPEAQKKKYAFKCHRMKDSTKIEHIYPVNAIAFHTVHGTFATGGSDGFVNVWDGRNKKRLCQFHKFPTSISSLAFSPDGSALAIAASFQHEYRLEQNPPADQIYIRHSLDRHQQTMSCLDQLKELTIVVADTGDFEAMKEYKPTDATTNPSLILQAAKLTQYQALIDEAVSYGRGPCEQLEEAMDKLFVLFGNEILKIIPGRVSTEVDARLSFDKDASINKAVKLISLYKELGVPKERVLIKLASTWEGIEAARILEKEHGIHCNMTLLFNFTQAIACAEAGVTLISPFVGRILDWYTANTDKKAFEPLEDPGVKSVTRIYNYYKKFGYKTVVMGASFRNTGEVKALAGCDLLTISPGLLKELANSNELVPHYLKAENATSIELEKISVDEKRFRWDMNEDQMATDKLSDGIRKFAADARKLEALIQEKLKMGDCVCRPLATVYAEDNEQSGTNVSMPAVFRVPIRPDLISFVHHQLLKNKRTPYAVSKEAGHQTSAESWGTGRAVARIPRVRGGGTHRAGQGAFGNMCRGGRMFAPTKTWRRWHRRVNVTQRRHAAASAISASGVTALVMAKGHAIERVNEVPLVVSDKVQEYKKTKQAVALLKSVKAWDDVEKVYKSKRQRPGKGKKRNRRYKKKCGPLVIYEKDNGIVRAFRNIPGVDTCDVNALSLFKLAPGGHAGRFIVWTESAFRKLNDIFGTFTKTSKVKKGYKLPRPMMTITDLGRLFKSEEIRGALRPKKSIIISKKNKPNPLKKIHLLGRLNPYALVEKRKTILAQQEQKSQRIQKKAALEKVAALKKKRLQSPFPKNRKLNQGAVLKKMLDAIKDLINEGNYDDIVSIYLHQAMDNSHVSLVALNLRADGFDKFRCDRNLSMGMNLTSMAKILKCAENNDIITLKAQDDADTVTFVFESQNQDKVSDFEMKLMNLDSEHLGIPETDYSVVVKMPSAEFQRICRDLSQIGDSVQLTCTKDGIRFSASGDLGTGNIQLSQTADVEKEEEAVIIDMQEAVTLTFALKYLNSFTKATPLSTQVCLSMSADVPLVVEYNMGHLRFYLAPKIDDSEMFARNGSK-LAGIAL---RNFSTSQRNNVKVAVLGASGGIGQPLSLLLKQHPGISYLSLYDIAHTPGVAADLSHINTGSKVKGFVGNDQLKAALEGIEIVVIPAGVPRKPGMTRDDLFNTNASIVRDLADACAQTCPKAMLAIISNPVNSTVPIASETFKKRGVYDPKRIFGVTTLDVVRANTFIAEAKGLDPVSMSVPVVGGHAGITIIPLVSQASPKVDFPQDQLEKLTKRIQEAGTEVVQAKAGAGSATLSMAFAGARFVFSLVSAIQGKNIVECAYVKSDIGDAGFFSTPLLLGKNGMEKNLGLGKLSKFEEKMVTDAMDELKKSVKKGIDFANKEYEWLLKFEVNDIVEQLVIAECSKRFPL-ARSDKFIM-QSKVVATLTGDSISHADISLRL-PKHSQRTIVQNDAQWKLQQIQDAGNHLMQAMNLLRFKFTSGQEVRNLMSDVMTCVGRGRACLVVPKKRTIEEIMQSRNMKSLQPPLPNDVAVSFYIQSYKLVFAVYHV-QKDPQKF-DAECSVPWLSEVLVLFTVALQLCQQLKDKVEVFQYNDFLPMFIPLFLEFNAPISEISDLLGVCDVCFKDAESVL--NGFVSILVCLFCERLESATVQPVAFN--VLYNFYEGLRVDVFCSLLRVAAGSVPEVFQDVSVTKKWLPVDKARQVYRNIHSALSDMPLRVMVELLSTYQDHDAAEANQDAIKCIAFAISDPNTYLMDHLIPLKPIKALENQPINELLKIFVYGKLSEYREFYRKHKEVV-EQLGLDHEKNVEKMRYLTFMYLAEKNQEISFDDIKREVEI--DDVEGFTINVLRTKLVTAKVNQPNQKVIVVSTMHRSFMKNEWEQLREILRLEKVEQSTQQFLEYLG-TR-RKKEVEELAEEAKAIDEGEERAVIEESPQFKELVNILVEWINDELAKHRII--VKNIEEDLYDGQILHKLLEQLTNSRIDVVEMTQNEEGQREKLKVVLERASQALGLKWSVDAVHSKNVVAIVHLLVALARHFRAPVRARLPENVVVSVVSVTKR-EQLTRTYDEYGMKVE-RDAFDQLFDHAPDKLTVVKKSLLTFVNKHLNKINIKDLD-KQFHDGFYLALLMGLLEGYFIPLYLTLTP----------------------NNKVNNVAFAFGLMKDAGLKPKARPEDIVNYDLKSTLRVLYNIFMQ-------------------MLNYGCSVMELSHRSTRIIKDAERDLRELLSVPPTYKVLFMQGGGTGQFAAIPLNLCPADYLVTGTWSRKAAEEAKQYVKYTRVPPASEWNLSPDAAYFYYCDNETIHGVEFPIVCDMSSNILTRPVDISKFGVIFAGAQKNLGPAGVTIVIVREDLVAVASVCPSILAYKVFAENDSLYHTPPTYAIYLLLVLKWIKSEGGVSGMARSAAKSQAIYDLIDQSGGFYCRSRTNIPFRI----RSNDALEKKFLKEAEMIQLKGHRSIGGIRASVFNAMDVVQAMKLYRERPEWQDVTPVLKISYTNTFRDCFGYLRAVISGELSERVFELTTTCADENPACYTVWVLRRRLLEHLKKDLNEEMEFMSRQIFDNQKNYQVWYHRQRLVQLRELDFIERVLDAKNYHAWQYRQWLLKTFNLWSKELNFCSSMLNEDIRNNSAWNQRYFVLKNTTGFVVEEEIKFTLDKISCNESAWNYLGGILNIYLYATLLSEALEIDIPRSPYWTFKQKLLRQLFCAGIALSLTCTLCLVFWLAIIIDRFEELLEFVPHLSRFLTAQGVAYRFIVINQGDRFRFNRGALINIGYLVSRAQCDYMVMHDVDLLPLNPKLSYRFPQGDNVHIAAPHLHPKYHYSTFVGGILLMRHAVFARLNGLSNKYWGWGLEDDEFYVRAKEANVRFERPTDIGTGINDTFRHIHDARRRPRDMVRIGSQREESRKRDRVTGLNNVVFQHQGLYQVRIGEVPVDVHNVHLHCDLTITPWCEKPRMSPGETITTDGYMRGHGGLLSAVAGVVEKVNKLITVRPLKTRYNPEVGDVVVGRIVQVC--QKLWKVDVGARLHAALHLHSVNLPGGELRRKSIEDELMMSQYFIDGDLVSAEVQNVGVDGAVSLHTRNLKYGKLGQGALVTVSPSLIKRCKHLHNLA-NGVHLIIGLNGFIWVTSSKFTREAICRTRNVILCLAIHNIMLYDTSIVHAYDIS-VAQLTRHRADKLVGSWLLTCSGMALGAVFLGGLTRLTKSGLSMVDWHPLNEGRPRTAEWEAEFAKYQQFPEYKVRNKDMTLEQFKSIYWMEYIHRMWGRTVGAAFYIPAAAFWARGYFNRGMKKQIIYLGTLLAAQGLMGWYMVRSGLEEKPRVSNLRLAVHLGAAFFFYGLLFRAALHRLIPSKPLVRFSWATGLVFTTALSGALVAGIEAGLVYNSFPKMADSWIPSDILAFPKWKNFVENPTTVQFDHRLLGETVLTALYIYSRKVPLPPRARLATHAMLAAWLQVGLGITTLLTYVPTPVAVSHQVGALTLLTTLLWLTHELKLIRR-LERYGIDD------------------VTEKNACQWSKDKLHSLFSDLEIND-SIMSVIKELKKCEGEATANNRKAKLIFFYEWELELCAGS-VVKGRVEIPNLSDENDIHEVSVNVTLVDEKIKGMMRSKGTEVIRSKLDEYVSSLKMDFSQGLILPTKDSGANSTATKGKKISTKELIMEDFKCRAEELYRAFTMVQAFTKGAAGGHFQMLDTNVSGKFLKLNELEFEWRFKSWPAEHYSFVITIEEVNADIITYTKVPPTSVKEISRLFFC-------GVMRTSQPLMDPETFLEIANHVSKLKMYPYFELAHCIVTLLYLREDLGTGSQLFSRKHPLSCWVSSMFSIYAGGIFAALLLGEPVLAVLKSNQSLILATACWYLIFYSPFDIVYKFCKILPIKLAIALAKEVTRAKKVHDGVHHAAKIYPSAYIIMVIIGVVKGNGTSFLKVFERLLRGFWTPQAMEIMQPSFATKACVIASLVFVVDKKTDLISAPHSLVYFGVVVFFVYFKLSSVVLGLHDPFIPFENLACAIFFGGIWDAISRAI-----NKGGDAAGSGLKDNKDTAAKKKEMFRPDGIVQRRTAAVIARDREEADRKDGDGCDKDKETRLTLMEEVLLLGLKEKEGYTSFWNDCISSGLRGCILVELGIRGRIDLEAAGMRRKSLLMRKVVVKNDAPVGDVILDEALKHIKETATPETLQNWVDYLSGETWNPLKLRYQLRNVRERLAKGLVEKGILTTEQQNFLLFNMTTHPLVDQNVKDKLIKRVQDSVLAKWVNDVHRMERRHLALLLLSHASDVLENAFNPLSDEEYEMAMRRVRDLLDMDFEAECAKSQTCDIMWGVFAAFVKMADGVPTFKCVLVGDGGTGKTTFVKRHLTGEFEKKYVATLGVEVHPIVFHTNRGAIRFNVWDTAGQEKFGGLRDGYYIQAHCAIMMFDVTSRITYKNVPNWHRDLVRVCENIPIVLVGNKVDVKDRKVKAKSIVFHRKKNLQYYDISAKSNYNFEKPFLWLARKLIGDANLEFVAMPALAPPEVQMDPEWQSKLENDMKEAQNIVLPDEEDDDLLAAQITPILRESKFRESGMLTPEEFVLAGDHLVATCPTWAWAKG-DKSYLPEDKQFLVTKNVPCSKRCRDMEEKIIEGD-EGWVDTHDEDNEDDEGVDMDDFLDEDLETAEILATRTYDLNITYDNYYRTPRLWLTGYDEKMKPLTTEQIYEDISQDFVKKTVTVENHPHIEGVPQASVHPCRHAQAMKNLIQTVEEGGGLEVHMYLIVFLKFVQAVIPTIEYDYTANFNMLSNLERKLPYDKLSANIDIVKKRLNRPFTLSEKVLYSHLDQPQSEEIVRGTSYLKLRPDRVAMQDATAQMAMLQFISSGLPKVAVPSTIHCDHLIEAQLGGDKDLSRAKDLNKEVYNFLATAGSKYGVGFWKPGSGIIHQIILENYAFPGLLMIGTDSHTPNGGGLGGLCVGVGGADAVDVMAGLPWELKCPKVIGVHLTGKMSGWTSSKDVITKLAGILTVKGGTGAIVEYFGPGVQSISCTGMGTICNMGAEIGATTSVFPFNSRMADYLASTNRRAIADAAEQVKDLLSADAGCKYDQVIEINLDSLEPHVNGPFTPDAAHPISKLGQTAKEKGWPLDVKVGLIGSCTNSSYEDMSRSAMLAKQALDHGVKSKSLFTVTPGSEQIRATIERDGQAKVLKEFGGMVLANACGPCIGQWDRKDIKKGEKNTIVTSYNRNFTSRNDANPQTHAFVTSPEMVTALAIAGRLDFNPLTDELTGSDGKKFKLKAPVGDELPRAGFDPGQDTYQAPPADGSNVKVDVDPKSQRLQLLSPFSKWDGKDLIDMVVLLKAKGKCTTDHISAAGPWLKYRGHLDNISNNMFIGAIPEESGEANKIQNRVSGSWGTVPEIARDYKAKGQPWVVIGDENYGEGSSREHAALEPRHLGGRAVIVKSFARIHETNLKKQGLLPLTFADPSDYDKIKSDDKISIVGLNGFAPGMESRGGMFKNTFQSGFLSILYSLGSKPLQIWDKKVRNGHIKRITDNDIQSLVLEIVGSNVSTTFITCPADPRETLGIRLPYIILIVKNLKKYFTFEVQILDDKNIKRRFRASNFQSTTRVKPFICTMPMRLDEGWNQIQFNLADFTRRAYGTNYVQTLRVQIHANCRLRRVYFADRLYAEDELPAEFKLYLPVQHRTAAQALRER---------------SYQYMQKARADIKRVTQQYRGLAETFIFNNGSSKELVCLDGTIPVRYKYNIPIRIWVLDIHPYHAPFCYVCPTPTMQIKTSQYVDESGRVYLPYLHDWNRNS-SDLIGVIQVMIMIFGEQPPVFSKSPNPYPSSNTLSITDQHIRISLLSAVESRITDRALEKSKAEEEVLRKTNEELQAGKQKLDRYMSDMERDREMESEKSEQLKQLDVDNAVTPTAPLYRQLLQAYAEESAVEDAIYYLGEGLRKGVIDLDTFLKHVREQSRKQFMLRALMQKCRQKAGLPMGRKFYVGGNWKLNGTKQSIQVICDRLKTSQSETEVCVGVPAPYIQFVRDLLPPSIHVAGQNCYKASSGAFTGELSVDMIRDCGGDSVILGHSERRNVFGENDQLIAEKCAFALQGGLVVIACIGELLEEREAGKTEEVVFRQTKAYADLIKDWKNVVIAYEPVWAIGTGKTATPEQAQEVHAKLREWLSKNVSEEVGLNIRIIYGGSVTAANCKELAQKPDVDGFLVGGASLKPEFVDIVNAKKMATALSKKRRFVADGVFNAELNEFLRRELAENGYSGVEVRNGAMKTDIIIMATRTQDVLGEKGRKIRELTAVVQKRFNFKEGTVNLFAEKVSARGLCAITQCESLRYKLIGGLAVRRACYSVLRCIMEAEAMGCEVVVSGKLRGQRAKSMKFVEGLMIHSGDPTNHYVETAVRHVLLKQGVLGIKVKIMHPFDAQGKRGPALMLPDKVTVMVPKYEDDNVETRSDNKDNAA--------GVAEPTTL----SKIEQLKQWSLSTYKCTRQILAEKMGKGTRTVDGELEANIELLRETHQKYLNILRLAKLLTTHFNTVATQAALGECFSDLAQK-------ELQQEFLYNAETQKNLSKNGDTLLGALNFFVSSLSTLCNKTIEDTLITIRHYENARLEFDAYRCEARANFKEKYERLRGDVQIKMKFLHENKVKVMHKQLLLLHNAVSAY-FSGNQSSLEATLKQFNISWLEQ-MVDLVLDRDIRIWVFLPIVVITFLVGIVRHYVSILLTSSRKAELQQVYDSQALIRVRYLRENGKYLPARGFFMRKHFFNDEETGWLK-TQKRAPPMNNPMSDPSMMSEMLKGNLTNVLPMIVIGGWINWTFSGFLTTKVPFPLTLRFKPMLQRGIELVSLDASWVSSASWYFLNVFGLRSIYTLVLGEDNAADSTRAMQDSMVPQAAAMPQDPKAAFKAEWEALEVVDHKWALTGIEEDLCSKPLQF---------MAQYKGAASEAGRAMQILKRRERQKEEVELKRQKIEQEMRVS-MGDKFSSHFDAVEAQIKSATVGLVTLNEMKAKQQDAVKEREKRLAQKELEEKQRAEDQKKAQKEKQKKAIQALSFNLDED-DEVEEENGEVYDKCKSDGASKCNSSSSAAGSNQSSGDDSHDNDAGLKDSRESK--IKVKKNPDVDTSFLPDREREEKERMIREELRQEWKDRQRALKEENIQITFSYWDGSGHRRVVDMKKGNSIYQFLQRCLDSLRKEFYELRVVSADQLMYIKEDLIIPHHYTFYDFIVTKARGKSGPLFAFDANEDIRMTSDASKEKEESHAGKVLLRSWYERNKHIFPASRWEPYDPTKCYDKYTIKDKKKNMSTATKKFVKPMVPETLLKQRKHNAELRQQRLLAAAAKKKAARARRVLAFRRAEQYVREYRRIETSEKNNRLVAKVNGNFFVPDEPKVAIVIRIRGITGVSPKPRKVMQLFRLRQINNAMFVRLNKATINMLRLAEPYLAWGYPNLKTVRDLIYKRGFGRVNGRRVPLIDNSIIEEKLGKYGIICMEDLVHEIYTVGPNFKQVVNFLWHFKLNNPKGGWRKKTTHFVEGGDYGNRETLINSLLRKMIVRLGAEDLWKSVTSVSNAGRKRGRASGHSKKASKDLNRGQKIGSGRINMLWPGLNAPIFRGREVVERQQLPPDPEREKRLIELRDKQQNFRRIRLTPLERGWSGTRAPGRSLGPPDTPEDIDIKDFDSVVLDLRLVSNMTSHLGRVRRHKAVVAVGNGKGLLGFATGRGPDGKTALRKAKNRALLRLCHYPMFEETVLHDFFSEYGCTRIVVKQKERGYGLKCHRAIMSLCKLIGIKNHVLFVAYSNIYINAIEKSFIV-LRYSRSKFDFADEKRLHVVEFRPEQLYFPRIVASPKTVRKEDEIDPYEELDINMIVSEGRMIYARKKAEPFYTRLPGWEVHLKKTDNLKNQRQVRLRLMKKYGALKSFLNIRKPLNQIGPRKSDEKYKWTNKQRLLIFASRGITYRDRHLMNSFRTMLAHSKEECKFE--KKDINEVAEMKNCNKVMYMENRRRSDTYMWLANMQTGPTLKFLVQNIHTMEELKFSGNCLRGSRPFLSFDPAFDSHPLVKEVLAQTFGTPAYHPRSQPFFDHVFVFRLLDKRIWFRNYQIVEE-DGSLVEIGPRFCLNLVKIFDGPFSGAVIYTNPHYVAPNKMRRLAQKE--NKYMYMDVFETKNTFYRMAVGKNKGLSKGGKKGLKKKIVDPFSRKDWYDVKAPSMFSIRNVGKTLVNRTQGTKIASDGLKGRVYEVSQADLQTGEDAFRKFKLVCEEVQGRHCLTNFHGMDLTTDKLRSMVKKWQTLIEAQVDVRTTDGFVLRLFCIGFTKKAQNQVKKTCYAQHAQVRAIRRKMVEIMHREVSSSDLKEVVNKLIPEAIGKDIEKNCIMIYPLHDVHIRKVKVLKKPKFDMGKLLEMHGEGKGSGGGDGMAVDRPDNYEPPVLEDVVKIGIIGGSGLE-DPELLKEDTPYGKPSDA-LISGKIDGIDVVILSRHGRRHTINPSNVNYRANLFALK-QEGCSHILVTTACGSLKEEVRPGNFTPNTFIDRTRIQTFYVCHLPMTKPFLSKLLSEHPKGTVVCIEGPRFSTVAESNVFRQWGADLVNMTVVPEVVLAHELGIPYAALAITTDYDCWR-E--ETVDVEKVSTLKLAADGACRILRATLPKVLKIAVM-------------------------VMRVLRHEEFEKGCLAACNGRYDGFWSKTMVGYGSEDDHFAMELIYNYGVDKYKKGNEFKGIVIQMSNVLKRAQENRWTVQQENDKSYVEAPGGFKFYVEEDSS-ERKDPVRQVIYSCTNVEKTRRFWVTMLGCEVVESGDDFLEVAYDKSKTSLRFEKIFEPIDRGEAYGRVAFACPRNQLPEIEAQVKNADGTVITPLVSLETPGKASVEVVILGDPDGHEICFVGDEGFHELSKEDPEASQVLQEKMK--EYDAYVEKVQRRDRRFRLPPLPTPADLLRFYRLRATKQLSQNFLLDPICSKFVRSSGHVIEVGPGPGCLTRPIFEQGAESVVVIEKDKRFMAALELLANATN-NKLKIVHGDVLNSKLEDLI-PAEKAKPWEGIHLIGNLPFSISTILIIKWLHMISRKSSAWQYGRVRMTLSFQKEVAERITSPERCRLSVIAQGCYVKNGGAFVPPPEVDVNIVRFVPVQPIFQPFDLVEKVLRCLFNKTLKIELAQELLQRSDVDPPVLISVEDYSKICDAYAGLCKEPGLYEYDFRSPRIVLLNRVAKTLRLAVGVRLHTSCRLNMPMVPIVIEQTGRGERAYDIYSRLLKERIICLMGSIDDDIASLVVAQLLFLQSESSKKPIHMYINSPGGSVTAGLGIYDTMQYVLPPIATWCVGQAASAASLLLAAGEPGMRHSLPNSRIMVHQPSGGVSGQATDIQIHAEEILYLKRTVNLLYAKHTNQALETIESAMERDRFMNPEQAKEFGLIDTVLEQPP-VREN---MRIERPVGTWLTLLPLWALTTAAPAGSLPDAALFCTGALLMRGFGCTINDMWDRDIDRQVERTRQRPLAAGRLSRWDALWFAGGQGLACLVLLHFNWNTVMLGLASVGLVVIYPVMKRFTYWPQAILAVVFNWGALLGFSATALPMYAGAFSWTLIYDTIYAHQDKRDDLLVGMKSTALRFGARTPLWLGAFSTMTTGLMGWPYYAAITLKIDNIGLLVGCIGGSLLKAAGSERQSVGRRSKILKAREPQVIENPKTSFVKGNNINQRTTQILKDIYALKKSESVFYQQRNPFEDPSPLEKLGKKDTSLFAFGSHNKKRPQNIVLGRTFDRMIIDQFEFGVENFKSLQEFKVPKIGVMLKPILVFSGESWQEMKRLKNFFIDFFKGDYIAVTGLEHVISFTILDILLRSYKVQLKKSGQKSPR-VEIEEIGPRMDLKIRRNKIASDDLFKQALRRPRELKVKRKKNMEQNDLGTSLGRVHMERQDFKLQTRKMKGLKVQILEALDDNYMYLVITREAAVVDPVNPEKVLRAVKVKLTTVLVTHHHWDHAGGNEKLLVFGGDDRIKELTDKDNVNITIGSLAQSLFTPCHTTGHVCYFIVFTGDTLFTSGCGKFFEGTAQQMQAAMLGSLPPETRVYNGHEYTVNNLKYAQHVEPDNQVKEKLLWAQKKPTIPSTIAEEKSINPFMRTSVQKHTDPVSTMNALRQEKDRFRMNRLFGKGKAKEPPPNLSNCIEVLDQRANNMDEKIKKLDAELFKYKEQMSKMREGPPKDLVKQKALRILKQKKMYESQRDNLMQQSFNMEQANFATQQLKDTKTTMEAMRLGVKEMKHEYKKVNLNEIEDLQDDLEDMLEQANEVQEALGRSYGMPEMDDEELEAELTMLNDEIALEDTSYLEDVTAPKVPSKGPSQKQKAEGGIMVDEFGLPKKEAQVRMVSRTRGKATLPDLPYDYNALEPVICAEIMTLHHSKHHNAYVTNYNIAAEKLQEAIQKNDTNAQIALQNAIRFNGGGHINHSIFWQNLCNPKSGEPSAELLAAIKKDFGSFEAMKDKMSAAAVAVQGSGWAWLGLNKANKQLQVAVCPNQDPLEASTGLVPLFGIDVWEHAYYIQYKNVRPDYVKAIWKVANWKDISQRFIKA-DDHIQHDVIKEALESGRDLREYSRSVEQQLKGSEDEAIRDYMHNCRDIAALHGEIASCDAILQRMESILRGFQNDLGSISSEIQSLQRQSVAMNLQLKNRQAVRGELSQFVDDFIVPEATINVILDCPVIDEDFLTQLALLDQKISFVKVQSFKEASSCQDVKDILDKLKVHAVTKIREWLLQKVFSFRKANANFQLAQNTMLKHRLFFQFLATHEREVAKEIREEYVDTISKVYLSYFKAYQTRLMKLQFEDVPDKDDLMGVDDTPKWGLFNKPSLKNRSTIFTLGSRNAVLGADLEAPVIVPHASAKSEKHYPFEQLFRSTQYALCDNAAREYLFVSEFFLLTKQGAVDMFDNILEKSMSLFAKHTESFVAECFDSIALFLCIHIIRKLKVVMHSRNVPVMDRYWDLLVKIIFPRFETILRMNIASIRDCDPSKLGSIDNRPHYITRRYAEFSAAIVSINENHPDERVTQLLGQLQIEVENFVLRMAAEFNGRKDQLIFLINNYDMMLGVLQQRTHEDSKETTNFRTLLTARQSEYVEQILTVHFGGMMTFIKESEYYVEKGQINELEKESHKVATLVRNFNSGWKKAIDDMNGDIMKTFTNFKCGTSILQEALKQLLQYYHRFNKVISQPPLCSLPVRSELINIHHLMVDIKKYKATFMNLAVTAVCLLVNVVVD---GYFIVVDAHSEECFHDRVVKGTKMGLTFEVAEGGFLDIDVKITGPDEKVVYNGERESSNKYTFAAYMDGVYKYCFSNAMSTMTPKTVMFTMDIGEEPKADGK---DGGENKLEDMISELHTAMTGVKHEQEYMMIRDRIHRSISESTNSRVVIWAFFENLIIIAMTLGQVYYLKRIFEVRRVVMRLSHILKLPKDYANLPKSYVKRAMAQVEWRTPNGRQFRRAVVQKPYTMNRPWTNEFMTNPPGVAIPVQVMFRGDRVEVMVGDDKGKQGTVNYIVPERNWVTVEGLNTEELPLLMGTQVKLVDPVDEKATDVEWRYTESGERVRVSVRTGRIIPIPLAYETIDYKYVDKAKDTSATELEKITFVP-KLATFEMDIMEQHGIQDDRVPHRTFWYMADNSE--IIVDLVEQKMELLLWLSRLACTMFTVSYLLGNPYSLYQKALICNGVTSALRLHQRAPTVRLNAEFLTTILVEDSCHYLIYSLIF-LPIS-MVLLPPMLFAVLHSLQLLERAGILLQ---SRVRVFRLIAMTEIFLMPTVIIGLFCGVSLMAPFMYYRFLKLRYASMRNPYNRNVFYELKLHANQLAAPNCPMIIQRVRFIESRAGLAIAVITVYLSFPEMRPEEQPYVKLPTSLEDAKNLGRVLSRYTDDAVVLAFFCTYIFLQSFAIPGSIFLSFLSGFLFPFLLAIFLVCLCSAIGASLSYLISYSVGSRLILAWIPNMVYYIIFLRITPFLPNWFINLASPIVDVHIVPFFIGTFIGVAPPSILAIRAGISLQQLASALTWENIGLLTGFAAFSLLPVLLKLKNKFEEVQDKLTRIAIVSIDKCKPKRCRQECKKSCPVVRMGKLCIEVTPNDRIATISENLCIGCGICTKKCPFEAIQIINLPSNLERDTTHRYSANSFKLHRLPTPRPGEVLGLVGTNGIGKSTALKILAGKLKPNLGRYNDPPDWTEILGYFRGSELQNYFTRILEDHLKAVIKPQYVDQIPKAVKGTVRELLDRRNDLGKEDYLCELLELVNVMDRQIADLSGGELQRFATAMVCIQKGDIFMFDEPSSYLDVKQRLKSAQAIRGQIEATKYVIVVEHDLSVLDYLSDFICCLYGTPGCYGVVTMPFSVREGINIFLDGFVPTENLRFRESSLVFKVSDNTDEDVKRLARYEYPSMVKKMGDFELKVQGGSFTDSEIIVMLGENGTGKTTLIRMLAGRLKPDGDEDVPTLNISYKPQKISPKSTGTVRFLLHEKIRDAYQHPQFVADVMKPLLIDNIIDQEVQNLSGGELQRVALALCLGKPADVYLIDEPSAYLDSEQRLAAAKVIKRFILHAKKTGFVVEHDFIMATYLSDRVIVFDGKPSVSTTANMPQSLLVGMNRFLELLNITFRRDPNNFRPRINKLNSVKDSEQKKNGTFFFLEDEEDGEDLFGPELDRYDAAEEEMRRRDRGRLPRGLESIENLEDMKGHTVKEWVTQGPKTEIFNRFKNFLRTYKEKIRAMEQNRMSLEVDYTLAQSEQVLAFFLPEAPAEVLPIFDEAAKDIVVGMFPHYGRIHHEIRVRITDLPILEEIRTLRKIHIDQLIRTSGVVTSTTGVLPQLRMVKYDCVKCKYILGPFVQSQEVKPSSCPECQSTGPFSVNVAQTIFQDYQRVTIQESPGKVNAGRLPRSKDAILLNDLCDSCKPGDEIEITGIYSNKFEGSLNKANGFPVFATVIIANHILKKDTDEDVKEVVKLSKEELAERIIASIGPSIYGHDDIKRAIALSLFGGVSKNPGQKHRIRGDINVLLCGDPGTAKSQFLKYVQQIAPRAVYTTGQGATAVGLTAYVSPVTRDWTLEAGALVLADKGVCLIDEFDKMNDADRTSIHEAMEQQTISIAKAGIVTSLRARCTIIAAANPIGGRYDTFHQNVNLSDPILSRFDVLCVVRDERLARFVVDSHSRHHPISQDLLQKYILYAREKIEPKLDQDKISQLYSDLRRESMATGSMPITIRHLESIIRLAESHARMHLREHVDVNTAIRVMLDSFVTQKFSVMRMGLLDDVKEIFGADTLYEVLGVKKSVRSDLLKKAYRQKSLLCHPDKANEENKEEFTKKFQILCKCYEILCDEEKRKIYDETGSV--DDSFDEKKDWKSFWRTLFPTVTDSQIQDFFDKYHGSEQEREDLKRAYEKTKGDMNKIAECFIGYDEDRLCSLLQKMIDDEEIPEYRAFTKETASSKAKRQKKFAREAKEAEK------NEMSSLELIL----KREGDDFLAALEAKYSKPK-KKLARSKK-TLLLAIAIGFLLLGKVGTKKLRKLEEKAEKRRLRELELQEREERAQKQAEDEYRKKVEQKKEDEQKQKEEEERRQKEEKERREHEEYLKMKAAFAVEEEGFDQEDDP-NSSQKLHEFITYINEHKVVQLENLAAQFRMKTQDCIDRVQRLLEDESLCGVIDDRGKFIAITRDELDEVARFIKVRGRVSIQELVENSNRLINLASDIMKQQEEAKAAALKHVVTMLQIPDQLDKVEQHRKRVQRKKASVEAMLKTAVQSQLEGVRTGILLLAAARDDVNDIQKDEADTIYDLSKLVQLQDVREESFRHSQTGTLMEHLKHIFNVPGSITRTQDLIQDGNLLLAHKLSDLECSRDELLYELHKQANNQASDRAMLKQYFADVERLSDELAKQLWLILKRTLNTVRKEPQVIVTALRLIMREEWAALKRQQST-GFLPPGRPKLWRKKAIETLEQSVAERLEGNQIEVRSENNMWLVRHLEVTRQLIIEDLKTVKHHCTPCFPPCFDIFNEVRMTHNCLSQRLQTIISGLVDSEYIHVLQWLNTYNSRELMGHPDLHVDVALLPDTIERLMERYLAGLRTKFEEWLRNALSDHKDWPEQDSNGYYRTETPMLIYQMITQHVEVARTVDLVSRVLRLAMNRMDYFLSQYNQLVTDYNFEDRSYTAYMIAIANNAVNMK-LRELKDNSLDYLCEEVLTDIKVMPDIMTETVTVTLADYGGDYIEKEVAASYVKAIC--EKRMSFRNYEERRAAAELEKLKKSKLDVLKLMAEVLKMKDSSLLSLEMNGLLFLLVFCLVW------DLRKGDTPTRVEEDIGKIKEGSRTDAEVIAREEEAIKVDSLSVAQLKEIREKAQKHAFQAEVARMMKLIINSLYRNKEVFLRELISNASDALDKIRLLSLTDPDALKKLQDLSIRIMADKENNVLHITDTGIGMTKEDLMKNLGTIAKSGTAEFLQKVSEGSSGKDLNDLIGQFGVGFYSAFLVADRVAVASKHNDDPIQHVWESNAAEFSVADDPRGDTLKRGTTVSLYMKDEAKDFLEHDTLKKLIEKYSQFINFNIYLWSSKT-VTEEVTESEKPAEE-----ADDDEAKVEDAKEP--LKTKKVDKTVWDWELINSAKPIWTRKEKEVSDEEYNEFYKAVTRDSQNPLAKTHFTAEGELTFKSLLFVPSKQPQDSFNRYGQKTDHIKLYVRRVFITDDFQDMLPNYLSFLRGVVDSDDLPLNVSRENLQQHKLLKVIKKKLVRKALEMFRKISEEEYVKFWKEYSTNIKLGVIEDSANRSRLAKLLRFPSSLDSTDKLVSLGDYVQRMKEKQTAIYYIAGNGMDEVKKSPFVERLLKRGYEVLYLTEPVDEYAISSLTEFEGKKFQNVAKEGLSLDD---NKDIREAMEKEFEPLTKWLSETALKDKISKAVISERLVETPMALVASQFGWTGNMERIVAAQTHMKENDPQKSFYMTQKKTLEINPRHPLIKELLRRVDDSPSDEMAKYLTEMMFETATLRSGFQLNDNAQFASNVERMLRKMMGVSEDAQVDAELEEAELPEVNPSDKDEDIESEPHDELGRRGEVKPLSAQEMKMIVISEIIQELVQAHNDKRDVNLNRVKYDVSARYGLKSQPKLVDIIAAIPPQFKQILLPKLKAKPVRTASGIAVVAVMCKPHRCPHINYTGNICVYCPGGPDSDFEYSTQSYTGYEPTSMRAIRARYDPFLQTRHRVEQLKQLGHDVDKIEFIVMGGTFMSLPESYRDYFIRNLHDALSGHTSANVDEAVKYSERSKTKCIGITIETRPDYCLQRHLTDMLRYGCTRLEIGVQSVYEDVARDTNRGHTVQAVCETFHIAKDCGFKVVTHMMPDLPNVDFERDVLQFVELFKNPSFRMDGLKIYPTLVIRGTGLYELWKTGRYKSYPPALLVDLIAKIFSLVPPWVRIYRVQRDIPMPLVSSGVENGNLRELALARMKDLGLVCRDIRTREVGIQEIHHKVVPYHIELVRRDYVANGGWETFLAYEDPQQDILIGLLRLRKCTEQTYRPELLGQCSIVREHVYGSVVPVHSRDPSKFQHQGFGTLLMEEAERIAREEHCSKKIAVISGVGTRNYYRKLGYELDGPYMSR

>Varroa_jacobsoni

PGQRVNVLSKELMTSVVLISGKKGCFIAGADITMLEQCRSEAKNLKPIVAAIMGSCLGGGLETALACRYR-IAVEEPKTTLGLPEVMLGVLPGGGGTQRLPKLIQLPTALDMMLTGKSLHAKKAKKVGLIDAIVKPLGPGLYLEEVAARDLATGELKIRARPLTERIVRDM-IFDKARGQVMKLTNGLYPAPLKILDAVRAGLEKQNFAELCTKESKGLMGLYHGQVHCKKNAFGKPTENIAVLGAGLMGAGICQVSLKDFNRVVMKDGLVRGQNQIKKKKIQKDRLMSTLLPTLDYSDMIIEAVFEDIHVKHKVVKEVEAEHCVFASNTSALPIAKISEVSKRPEKIIGMHYFSPVEKMQLLEVITTDKTSKDTAATAVDVGLRQGKVVIVVKDGPGFYTTRILAPMMSEAMVLLMEGCQVKELDKLKAFGFPVGAATLLDEVGIDVGAHIAEGVFGDRKEMVNNFLGRKSGKGCYIYRAVNPIQRKYTTEQVQFRLATRFMNEAVMCLQEGILANPVEGDIGAVFGLGFPPNRGGPFQFIDTYGADKIVNMRQFQEFEPCQLLLDHA-NDPLKKFH???????????????????????????????????????????????????????????????????????????????????????????????????????????????????????????????????????????????????????????????????????????????????????????????????????????????????????????????????????????????????????????????????????????????????????????????????????????????????????????????????????????????????????????????????????????????????????????????????????????????????????????????????????????????????????????????????????????????????????????????????????????????????????????????????????????????????AKVLNRLEAFREHEMAVTRDYISQPRMIYKTVCGVNGPLVILDQVKFPKFAEIVQLVLADGTPRTGQVLEVSGDRAVVQVFEGTSGIDAKNTVCEFTGDILRIPVSEDMLGRVFNGSGKPIDKGPPVLAEDFLDIQGQPINPWSRIYPEEMIQTGISAIDVMNSIARGQKIPIFSAAGLPHNDIAAQICRQGGLVKRPQKSVMDDNFAIVFAAMGVNMETARFFKQDFEENGSMDNVCLFLNLANDPTIERIITPRLALTTAEFLAYQCEKHVLVILTDMSSYAEALREVSAAREEVPGRRGFPGYMYTDLATIYERAGRVEGRNGSITQIPILTMPNDDITHPIPDLTGYITEGQIYVDRQLHNRQVYPPINVLPSLSRLMKSAIGEGFTRKDHADVSNQLYACYAIGKDVHAMKAVVGEEALSPEDMLYLEFLGKFEKNFISQGRYENRTIFESLDIGWNLLRIFPKEMLKRIQHSLLAEFYPRAEAKRVVTA------TGLFEDPNGFYLLKENAITQAEQLIAEAMYRKRKMVQIFDDLSDCLCRVADLAEFVKVGHPQGRYAQAAEHASLAISSLVEKLNTNRELYSALRSVIENG-DIVPTTAEQHVGRLFLFDFEQCGIHLDEERRQRVVALNDHILYVGGQFLQNSHRPRYVRQSGMPENLLVVSGLQADCSNELVREAAYRIYLYPDDHQLGLLDELLRSRHELARLCGFDTYAHRVLKGSIAETPENVTFLSYLSAELGPRAERDYQEMMGMKPWDVPYFTAYLSLASCMEGLDMIFNALYGINLEVVGELWHSSVVKLVVKNLASHMGVIYCDLFERPGKPHQDCHFTIQGGRRSDGSYQIPKVVLMLNLPPPLLTPSLMDNLFHEMGHAMHSMLARTEYQHVTGTRCATDLAEVPSILMEYFASDPRVVSKFARHYRTGELMPAEMAASLDASRVIFQASETQLQVFYAFVDHEYHSKYPLNTTEILRDVQNKHFGVKYVDNTAWQLRFGHLVGYGAKYYSYLMSRAVAATFWHRAFNADPFSRCVGTYREEVLAHGGALPPAQLIQNFLLAESLIRDIMPSDAKKKRDAKKKEALKNRNNPDATNGEDEMDEVTKKFEEDMKLNAAARAVTGVLSIHPRSRDIKIENLSITFHGWEVLQDTKLELNCCRRYGLIGLNGCGKSTLLSAIGRRELPIQECLDIYHLTRECPPSEKTALQMVLDVDKERARLEKLAEELAASDDDTSQEQLMDVYERLDAMSADTALAKASYILHGLGFTQSMMHKKCKDFSGGWRMRIALARALYVKPHILLLDEPTNHLDLDACVWLEEELKTYSRILILISHSQDFLNGVCTNIIHMNLRKLEYYGGNYDQFVITRNEMLENQMKRYNWEQAQMSHMKDYIARFGHGSAKLARQAQSKEKTLAKMVAGGLTDKVVYDKTVSFYFPSCGTIPPPVIMVQNVSFRYTDKTPFIYKNLEFGMDLDTRVALVGPNGAGKSTLLKLLCGALVPTDGIIRTHSHLKIARYHQHLHESLDVDLSALEYMMKSFPDVREKEEMRKIIGRYGLTGRQQVCPIRQLSDGQKCRVVFAWLAWQVPHMLFLDEPTNHLDMETIDALAEAINNFEGGMVLVSHDFRLISQVANEIWVCENQTVTKWRGDIKTYKQHLKNKVMKEMEKMVLADLGRRITSALRNLSTATVINQEVLDSMLKEICAALLESDINVRLVKQLRENVKAAIDIDEMAVGLNRRKVVQSAVFKELVKLVDPGVRAWQPSKGRSNVIMFVGLQGSGKTTTCTKLAYYYMKKGWKTALVCADTFRAGAFDQLKQNATKARIPFYGSYTEVDPVVIAADGVSKFKAEHFEIIIVDTSGRHKQEDSLFEEMLEVSNAVSPDNVIFVMDASIGQACELQARAFKEKVDVASVIITKLDGHAKGGGALSAVAATRSPVIFIGTGEHIDDFEPFRVKPFIQKLLGLGDIEGLIDKVNELKLDENHELIEKLKHGEFTLRDMYEQFQNIMKMGPFNQIMGMIPGFSADFMSKGNEQESMARLKRLMTMMDSMTDEELDDREGAKLFARQQTRITRVARGSGCSTFEVHELLNQYTKFAAMVKKMGGMKGLFKGNDLARNVNPAQMNKLSAEMAKMIDPRVLQQMGGFSGIQNMMRQMNASSLMQDGTFIKLKKLYSMKKGTLNINSLFENDPSRAEKYTIKLADGDETLLIDYSKNLIDDEILVNLVELAKNREVELMRAKLFSGEKINFTENRSVLHVALRNRSNRPITADGEDVMPKVNAVLEHMKAFCQQVISGEWKGYTGKKITDVVNIGIGGSDLGPLMVTEALKPFQVGPRVHFVSNVDGTHLFETLKKVDSETTLFIIASKTFTTQETITNAESTKQWFLDKAGDKAHVSKHFVALSTNKPKVEAFGIDAANMFEFWDWVGGRYSLWSAIGLPIALFIGMPNFEKLLAGAHFMDEHFRTTPLDKNVPVILAMVGVWYINFFGAESHCLLPYDQYLHRFAAYFQQVDMESNGKYVQRNGERVDYQTGPILWGEPGTNGQHAFYQLIHQGNRLIPCDFIAPVKTHNPIRGGVHHKILLANFLAQTEALMKGKSEQEAKSELKASGLSEEVLERILPHKVFLGNRPTNSIIVQQVTPFTLGALIAMYEHKIFVQGVIWNINSYDQWGVELGKQLAKRIELELNGKDPVSSHDPSTNQLINFINSYNTNVSYQDRDKPAQVRQSNITASKAVCDAVRTSLGPRGMDKMIQAVSGDVTITNDGATILQQMQVLHPAAKMLVELSKAQDIEAGDGTTSVVVIAGSLLDAASKLLLRGIHPTIISEAFQAAAKECVDILSCLAIPIELSDRESLLQSATTSLCSKVVSQHSDVLAPMAVDAVLKVIDPNNVDLRDIKIIKKLGGTVEDTELIDGLVFTEKLAGGNSPHRVEKAKIGLIQFCISPPKPNMDHQVIVSDYTVMDRVLREERAYLLNIVKVVKKAGCNVLLIQKSILRDAVSDLALHFLAKMKIMVIKDIERDDIEFISKSLGCRPIASLDHFVPEALGSAELVEEVTSAKYVKVTGVANP---KTVSLLLRGSNKLVLEEADRSIHDALCVVRCLVKKRALVPGGGAPEIELSLRLAERAREIEGLHSYCYRAFADALEIIPYTLAENAGLNPIQTVTELRNRHAQDKRTYGINVRRGCVTDILEENVLQPLLVSTSAITLAAECVRSILKIDDIVQTVRMSKRDVVYLWDPDVGNFHYGPGHPMKPQRIAVTHSLVLNYNLHTKMRIYRPYRANPHDMCKFHSEEYVNFIERVTPKNIQTFSKSLTHFNVGDDCPVFDGLYDFCSMYTGASIDGAWRLNNKSCDIAINWSGGLHHAKKFEASGFCYINDIVVAILELLKYHARVLYIDIDVHHGDGVQEAFYLTDRVMTVSLHKYGAYFFPGTGDMYEVGAESGKYYALNVPLKEGECFLNLKYVFKSVISSVIEHYNPGAIVLQCGADSLAGDRLGCFNLSIKGHGECVRFVRDLNIPLLVLGGGGYTLRNVARAWTNETAILVDEQVSSEIPFNEYLEFFAPDFSLYPD-------ENANSKQYLEQIIKYTTENLRCLDHAPSVQMQDVPPELADLGDDQEKRETTKEHPAEFYD---------------MSDAEEDYELEYSEDDDSQPDVDLENQYYNSKALKEDDPQAALQSFQKVLDLEGGQKGDYGFKALKQMVKINFQLGEFKEMMSRYKQLLTYIRTAVTRNYSEKSINSILDYISTSKKMDLLQEFYEVTLEALRDAKNDRLWFKTNTKLGKLYLDREEWNRLARILRQLHLSCQNVDGSDDLRKGTQLLEIYALEIQMYTSQKNNKELKKLYEASLQIKSAIAHPLIMGVIRECGGKMHLREGEYNSAHTDFFEAFKNYDESGSPRRTTCLKYLVLASMLMQKEINVLDSQEAKPYKDDPEIVALTDLVDAYQAHDISRFESIVSPHKESIMKDAFIKEHIEQLLLNIRRQVLIRLIRPYTRITISFISRELNIPSAEVESLLVSCILDNTINGRIDQVKQVLELNP-SPTDRRYTAMEKWAAQVQLIQSTVVGKLAQLGTLNPVQILNQQAEEEKAENARLSSFVGAIAIGDLLKSTLGPKGMDKILLCETSRDSKVEVTNDGATILKAIGIDNPAAKVLVDISKTQDDEVGDGTTSVAVLAAQLLQEAEKLVGMRLHPQTIIAGWRKAVVAARAALEEFSQNRSNDEAQFRIDVLNIARTTLGSKILSQHKDFFAQLAVDAVMRLKGKSNLDAIHIIKKLGGSMLDSHLEQGFLLDKKPGLNQPKRVEKAQILIANTPMDSDKIKAGKLRDECFSRVVVAELEDAEKLKMKRKVDAILAHKCNVFINRQLIYNYPEQLFADAGVMAIEHADFDGIERLALVTGGEIVSTFTSPESVRLGTCDVIEEVMIGEDKLLKFSGVPLGEACTIVLRGATQQILDEAERSLHDALCVLAFVVKEKKICYGGGSAEMLMAAAVDSVAQTTPGKEALAIEAFARALRQLPTIIADNAGLDSAQLVSELRAAHANGQSTFGINIADAKIDDMEKLGVTEAFVVKRQVLLSASEAAEMILRVDSIIKDAPRKRVPDRSHMAASIKPSGKPQLLCRLDGHTDTVNQVVLIGDADAVISVSDDRTIRVWARRDTGQYWPSVCHTMPSLASAMDYDPQLRRLFVAMDNGSITEFELADDLNKITYRRSYIAHQQRVTSMKFSPVTEWLISAGKDKYFQWHCTETGRRLGAFLGSAWCTTVALDQASRHAFVGDYSGEITMLKLTETSYQPVTTLKGHSGSVQSLLWDERRRLLISGGFDQIIIVWDIGGGKGTAYELSGHRARITGLALYSASSLLSVSEDSTLVVWDVAAQRQETPEWTTRDCCERCARPFFWNLRARTSNSDVGSRQHHCRRCGRALCDACSENRSTLPRLGFEFPVRICNECHLHISDGDREPLAKFHDLKAPVTAMSLSAESKTMVTIGTDRSIKIWDLSKVLMGDQVKEGTASKKALKKAQKEAEKAARKAAHK---AAGKENGSDDGSQGLYGQYPMIQSTEKLQREMIEISQCTLERADQMVWLRGRLHTSRAKSKQCFFVLRQQHYTLQCLLDVSEGTSKQMLKFISAIPKETIVDVEGKLVKSPLKIESCSQQEVELRIYQFWIVSLSDTRLPLQVEDASRPEPAEGDEEALKIRVNQDTRLDHRVLDLRTPANQAIFRLQAGVCHLFRESLNRRHFIEIHTPKIISAASEGGANVFEVTYFKGKAYLAQSPQLYKQMAIAADFDRVYTIGAVFRAEDSNTHRHLTEFVGLDLEMAFKYHYHEVLDTIAEMFVDIFKGLRDRYQPEIDTINKQYPSEPFKFLEPSLRLEYSEGVAMLRAAGVEMADDEDLSTPNEKLLGRLVKAKYDTDFYVLDKYPLAVRPFYTMPDPNNQMLSNSYDIFMRGEEIMSGAQRIHDPEYLTQRAKAHGIDISTIQAYIDSFRYGAPPHAGGGIGLERVVMLYFGLNNIRKTSMYPRDPKRLTPAMEEGLQLNEINPLEFSRVKMVYPKECRIRRISYRGKLDLTLWWMNGIKQEPIKRTCGEIPIMVKSLKCNLYGLDPEQLVERGEEMEEFGGYFVVNGNEKVIRLLIMQRRNYPIAMARNGWKNRGSMFSEFGVSLRSCRRDAQNMVLHYLTNGTVQVMITYRKEVYFVPAVLLLKALVNKSDYDIYRTLTQGCENDSFYKGCITNMLRLVQENILTQDAKEFIGDKFRWSSNADVCDQLLKRCVCVHLDSNEDKFNLMCFMVRKLFAVAKNKCALESADSTMNQELLLPGHIYLHILKEKIEGLLVGVKISIDKKMSNALDVTNAMNYFLSTGNLVSKTGLSLQQTSGFTILAEKLNFWRYLAHFRCVHRGSFFMEMRTTTVRKLLPEAWGFICPVHTPDGGPCGLLLHLSAMCEVVVQLDGRCIGWVLRYLKATGNIPPTLEICVIPRTEQNSLFPGLYIFSTPARMMRPVLNRTQTVEWIGTLEQVHMDICVIAEEATTHQELRETAMLSVLANLIPWPDFNQSPRNMYQCQMGKQTMGSPMHTFRYRADNKLYRLYPQSALVRPTSYDHFQMDEYPSGTNAVVAVISYTGYDMEDAIVLNKMSVERGFKAGVYKTETINLRVDVDGLPYIGSQGDPVCAYIQLKTVRYYSTEPAIVHEVKILGNLQQIQLTYLIRTPMIGDKFASRAGQKGICSALWPTESMPFTDSGMVPDIIFNPHGFPSRMTIGMVVESMAGKSAALHGYVHDASPFKFSEEYPSSAYFGELLQRAGYNYYGTERMYSGVDGREMEADIFFGVIYYQRLRHMVADKYQVRTTGPVDSLTRQPVKGRKRGGGIRFGEMERDSLLAHGTAFLLHDRLFNCSDKTLCATCGSIISIPIPYVFRYLAAE-MVSINMKIIRDTSRDLHRHQRNAPELHPMTVVREYTAAVNAAKLDRIFAKPFLCSLGHRDAVELLAKHPDRISGAVSASADGELRWWDLSNRKCVRALQAHDGPIRGLVGQDQTIKTILSKLAHHYNNMFATAGETVSLWEERNEPLRSFW-GVDTIYVIFSPIESLSSDRSIVLYDIREASPLRKVILEMRSNALAFNPMQAMHFTVANENYNLYTFDMRHLKKALQSHTDHVGAVLSVDYSPTGTEFVSGSYDKSVRIYRSREVYHTKRMQRVTSVMYSLDSKYILSASDEMNIRLWKAKASEQLGIQNYNETLLQRFQHHPQVKRIVRHRHIPKTLYQEKQTMVTARKRKMGMQFLQRGAYQDALSHYHAAIEGDDTNYQSFYWRATVYLALGKSKLAVEDLNRVIELKDDFLKAREQRGNILLKQGHLDEAHIDYEFVLRLEPDNPEAMIEELKNDVIHILQRVWNLKLRELRASCYESIGDIQSAITDLRPAIRSVPDNTGGYLHLAQLYYKHGDPDDSLTTIRECLKLDPDHKECYKNVKKLAKSMQEECVEKMVGLIKTKCQCASKGGSAVQICTEALALDPILCDRGEAYINQDDFKQDFAAARELDQRAGEGLKRAQKLEKSRGKRDYYKILASKREIAKAYRKLAAEWHPDQY-QGID--KKNAEKKFIDIAAAKEVLTDPDKRAKFDRGEDPLDPESGFHYTFPFMLVLFESPAGYAVFKVLDEKKVQKTDNLFKEFEDASGAAKILKLKHFQKFQDMTQALSAATAAIEGKLCKPLKKVLKKLAVSDAHETLAVADAKLGNIIKEKMDILCVANSSIQELMRCIRSQQEALITGLSQKEATAMALGLAHSLSRYKLKFSPDKVDTMIIQAVSLLDDLDKELNNYVMRCKEWYGWHFPEMSKVVTDNMLYVKTVRKMGMRSNAINLDLSDILPEDQEAKIKELAEVSMGTEIAPDDVANIMHLCDEVIQMTEYRGTLYEYLKNRMTAVAPNLTVLVGELVGARLIAHAGSLLNLSKQPASTVQILGAEKALFRALKTKHDTPKYGLIYHAQMVGQSSQKCKGKASRWLAAKSALAIRVDALGEDTDTEMSLRNRANLEARLKMLEDGKLTRISKTGSKSNKFDVYRHKSEILEYKPAKDSTL--GKKRKFADDDDDAHEPKQFKSK------HSD--VQQEQEEDQ-KVEQGSEEQ-----------SPVEKKKKKKKKSK------VAEEQEEVE------------------LFLWPKNSTKLQFSIFVCFGIMIVGRVCTPIAPIMQKKIVDGLVSSTGLLF---LFQGANSLMANVRSYLWLGVQQYTTKATQVSLYAHLHSLSISWHLSRKTGEVLKVLDRGTSGVQNLCSYLLFQIFPALTDIVIAFGYFTYAFNWFALVAFVCMGSIVLTEWRTKFRHEMNHLENAAYAKSVDALLNFETVKYYNAEEIEIAKYQNAEWKSQSSLALLNIIQGSTSSIIGALLCAYITAGDYVLFIAYNAQLYAPLTFLGTYYRMIQQSFTDMENMFELLDVVDHHLKL--EIEFRDVCFRTVLKHISFVVPHKHTVALVGHTGSGKSTILRLLLRFYDVQSGSILIDGQNISAVSLRSHIGVVPQDTVLFNMSIRENIRYGRPSEDVEAAAAAADLHHSIFQMYETVVGERGLKLSGGEKQRVAIARTILKAPSIIVLDEATSALDTQTERNVQKALNVLENRTSIVIAHRLSTIINANQIMVLEHGEIVEQGHEELLSKKYASMWRQQQAFLLRRQLTHLTRTFSQSLLNVPETRVTTLSNAVRVASEDTGAPTATVGIWIDAGSRYETEKTNGVAHFLEHMAFKGTGKRSQTDLELEVENAGMHLNAYTSREQTVYYAKCLKKDLARAVDIIADITQNPKLGEQEIERERGVILREMEEVEGNLQEVVFDHLHSIAYQGTPLGLTILGPTENIKSLQRQDLKDYIDTHYKGSRIVLAGAGGVDHDELVKIAEQTFGKVSNSMDSQAPCRYTGSDIRVRDDDMPFAHIAIAVEGAGWANADNIPLMVANTMIGSWDRSHGGGANASSRLAAWAQSVKSMHSFQSFNTCYKDTGLWGLYFVADGDELDDIMIAVQEEWMRICTEATDGDVTRAKNLLKTNLLLQLDGTTPLCEDIGRQMLCYGRRIPLHELEARIDAIDADTIRNVCQTYIYDRCPVVAAVGPVEGLTEYTRIRGQMYKHSVHTLVFRSLKRSHDMFICEEGALPPIDEKAHKLRVGTKGRDEYGPVMHLVSEGRRSVSSGPLVLAGQQLTAQLKKPSIPKPTWHPPWKLYRVISGHTGWVRCVAFDPTNEWFCTGSNDRIIKIWDLASGKLKLSLTGHISGVRGLAVSQHHPYLFSCGEDKQVKCWDLEQNKVIRHYHGHLSGVYTIGLHPTIDVIITGGRDSTARVWDMRTKANIHVLSGHTNTVASVLVQATEPQVVSGSHDSTIRLWDIVAGKTRVTLTHHKKSVRALVLHPKLNMFASGAPDNIKQWMCPDGKFIQNLSGHNTIVNCLAMNEDGVLVSGGDNGSLQFWDWKTGYNFQKLTTPVQPGSIDSEAGIFAMSFDLSGTRLVTCEADKTIKIFKEDETATEETHPINWRPEIVTGSTSEYLINCSAEHYLEILGINVKAKNFLVFQGAVESIAMKNPKERTVLFEEISRSMISAEEETYQKKKGIAAERKEAQIEEAEKYQKLKEDVQVNLHLFRLFHQEEELQKKRRKDKIEAELKEKKKVEQTHRDDVELNKRKPAYIKAKEKTAHMQKKLDAAKKSLEAATKTHKSHQGIEELEHELSQVEEAYEQEDVSLEESQVKEYNRLKEKAGKMASAALQEYDSVAREQKTDQDHLDNELRKRNECEAKLKELEENQRRINKLVDLQDLKEEEKKLEAKKRLTQKFEDVASLGDAKVDKHEDARRKRKSEIVEHFKKLYPGVHDRLVNLCHPIHKKYNVALTKVLGRNMEAIVVDTEKTGRACIQYLKEQMLEAETFLPLDYIDFKPLKERLREFKDVPNVKLLYDVLKYEPLSIKKAVLYATNNALVCETAEDAAKVAFQSPDGKRYDAVALDGTYYQKNGFISGGSSDLAKRAKRWDDKDFHKLKDQKEKLQEDLREAMKTARKESDLTTIESQIKGLETRIKYSKVKQLEQSMREREGRINEIKARQNTVEDDVFRDFCEQIGVANIREYEERELHASQEREQRAELENQKNRIASHLEYER--TKDTLAVEQDQQELGRLKEIEQKQKELIEQQMEAISTLKNERQSKKIRVDEIDEEVAEIRKRLTAQQKEVTGVQKTVTQAEAKLEQKRSERHTLLQSCKLEGIRIPLIRGSMSQMYAREAMEIDYNQLRMEKEINLQRIQAPNFKAMEKLDSVKERLKDTDTEFEHARRKAKSNFELVKRERTCFEHVSNCIDEIYKSLTNNPSAQAFLGPENPEEPYLEGINYNCVAPGKRFQPMSNLSGGEKTVAALALLFAIHSYQPAPFFVLDEIDAALDNTNIGKVARFIREKTQTSFQCIVISLKEEFYGHADCLVGICPDPGECTISRIYTIDLSMSEAMLLYLGQAIREGNLAEMQNIYERSLVNLMDQYLIIIYKE--LYYRQIYADDRFSSYYNYVNLFNYILSSGPVSLDLPNQWLWEIMDDFVYQF--QSFSTFMWNVHSVLNVLHSLVQKSNINQQLEVFSKGGNPDEVAGEYGSRPLYKMLGYFSLIALLRLHSQLGDYYQAIKVLQHLELNRKGLSRVPACQVSTYYYVGFAYMMMRRYEDAIRTFSDVLVYIGRTKRNYQLRQMNNQMEKMYALLSICMVLHPQRMDESVLQQLKLKHMMKMSQGDLDTFERCPRFLSPVQWRAFRGEVVAQMNMRSFLKLYTTMPVEKLTKFVLMCFKHLMSNVVLDGEFQTGSDMDFFIDKDMIHIADTKLARKYGEFFAKQYNRFEEWYEMAALRNGSLLIPRLVQALRERNQSFFAHVEMGPPDAILGVTEAYKKDPNPKKMNLGVGAYRDDDGKPFVLPSVRAAERQLMSKNLDKEYLPIGGLNDFCKNAAILALGDNSAVIKEDRNATVQGISGTGSLRIGAMFLDEFLKGNKTVYMPNPTWGNHIPLFKRCNFQVKQYRYYDPKTCGLDFQGALEDISSIPEGSVILLHACAHNPTGVDPRPEQWTEIEKVVRERNLFPFLDMAYQGFATGDIDRDASAVRLFAGSG-PMCLAQSFAKNMGLYGERVGAFSLICSSAEEQARCMSQIKILIRPLYSNPAVNGARIANLILSDPQLRAQWLKDVKGMADRIITMRSRLRSGLKREGSTHDWKHITEQIGMFCFTGMTAEQVTRLIKDYSVYLTKDGRISVAGISSHNVDYLARAMHEVTKHIRPSFYHALHCILMAVLMYVFVEFSKPPDQVDPFSEYGVIFTIILYLFRLLPLLALPQSLTNLFGLTLYNAFPPRVRLKVKPHEAPFLCIRVVTRGDYPGLVRENVKRNLATCLDTGIDNFVIEVVTDKEVYVTANSKIRQTVVPKSYNTTTGAMFKARALQYCLEDNVNLLADGDYILHLDEETLLTKDALRGVLNFISAGRCSFGQGLITYANERIVNWFTTSADMYRVADDLGKLRFQFNFFHKPLFSWKGSYVCTRVGAEREVSFDHGPDGSVAEDCYFSMVAFSKGYSFEFIEGALWEKSPFTISDLIQQRKRWMQGIYLVVHSAKIPWRYKVWLSCSLYAWATMPLSTSNLVLAPNFPLPCPQTFNIICAFIGALNIYMYIFGLIKSFSISRYGFFGFWLCFMLVVIAIPLNIVVENVAVVWGLLGNKHKFYIVDKNIVMETQTEGCGEPAKLQCPTCLKLGIKGSYFCNQVCFKGSWNTHKAIHKTAKNSPYNPWPHYHFSGKLRPGRISTKRTVPAHIKRPDYADHPEGIPVSEQAMKGA-EIKVLNEAEQEAVRKASLLARECLDVALAAAKPGVTTDELDRLVHEAAIARNCYPSPLNYYKFPKSCCTSVNEVICHGIPDDRPLKDGDILNVDVTVYHNGYHGDLNETIFIGKVDEAAKKLVRVTYESLQKAIECCRPGVLYREIGKIIQKHVQQNGFSVVKSYCGHGIHSLFHTAPSVPHYAKNKAVGVMKAGHCFTIEPMISEGVWQDEVWPDNWTAVTTDGKRSAQFEQTLLVTDTGVDILTRRRKKNGQPWFMDQSTLRECLKERSAKRRELLARQLGAGCAENLGLLLGNDKTTATEQGVTLDDEEVMAYRDSSTFLKGTQSANPHNDYCQHFVDTGQRPQNFIRDVGIQDRFEEYPKLKELIKLKDELIHETATPPMYLKCDLLQYNLRELNGKFDVILIEPPLEEYQRSCGVTNTRFWSWEEIMKLEIEEVAAPRSFVFLWCGSSDGLDLGRQCLRKWGFRRCEDICWIKTNINNSKVKNVEPRAVFQRTKEHCLMGIKGTVRRSTDGDFIHANVDIDLIISEEPPFGMMEKPEEIFHIIEHFCLGRRRLHLFGRDLTIRPGWLTLGPELTNSNLNTEAYNAHFNTANDYLTGCTERIEALRPKSPPPKSALGGPSRGQRGRTSLNKLNLLALDVAGKRVVMRVDFNVPLKDGKITNNQRILAALPSIKYCLDKGAKSVVLMSHLGRPDGQSNSKYSLAVVAEELNQLLGKKVIFLNDCCGAQIEAACADPTPGSVILLENLRFHIEEEGKGVDTAGNKIKADASKVKEFRASLTRLGDVYVNDAFGTAHRAHSSMVGVELPRRAAGFLMKKELDYFSKALDQPARPFLAILGGAKVKDKIQLIENLLDKVNEMIIGGGMAYTFLKVTRGMKIGDSLFDDDGAAIVEKLMAKAASNNVQIHLPSDFVIADKFHEDATTGTADISNGIPDGWMGLDCGPKSVELFAGAVSRAKTILWNGPAGVFEFEKFAVGTKGLMDVVVAATDRGAVTIICGGDTATCAAKWGTEDKVSHVSTGGGASLELLEGKILPGVAALSDAMRTFGDRPVSFQLEDGGDYYYIGTEVGNYLRLFRGTLYKKYPSLWRRAVTVDERKKISQMNMSQHSAANFISLLKKSEVDDLIDGNEEKYRAAPVQENEGGGHGAKNARPSFMPAAPNNAHHLDAVPCSTPINRNRLQHKKNKSFPMLYDDLDPAMLHESAALPECLVPIRLDMEIEGSKLRDTFTWNRHEAHISPEQFAELLCDDLDLPPLLFVPQIAASMRQQIEAFPSESLLDEQTDQRVLIKLNIHVGNISLVDQFEWDMSERANSPEEFATKLCSDLGLGGEFVTAIAYSIRGQLAWHQRTYAFSEAPLAQLEMPFRAQSEAEQWCPFLETLTDQEMEKKIRDQDRNTRRMRRLANTGWMGANGSTLDRDDALPSSERYLGLVNFGNTCYCNSVLQALYYCKPFREKVLEYKAKNKRTRETLLTCLADLFHNIHSHKKKTGTLAPKKFIARLRKDNEVFDNYLQQDAHEFLNYLLNTIGDLLQAESSWVHDIFQGTLVNETRCLTCETVSSKDEDFLDLSVDISPNTSISHCLRGFSSTETLRGEHKYHCEQCNSKQEAQKSLKVKKLPPILALHLKRFKYTEQQNRNTKLSWRVVFPLELRLFNTSDDALNGDRLYDLVAIVVHCGTGPNRGHYISIVKSHGLWLLFDDDMVDKIDPSTIDDFFGLTQDTPKSSESGYILFYQSKENADIDNWIELAKQCKYLPEADLKKLCNMVCQILIEENNVQPVSSPVTVCGDIHGQFYDLEELFRCGGHVPDTNYVFMGDFVDRGYYSLETFTRLLTLKAKYPKKMTLLRGNHESRQITQVYGFYDECQQKYGNANAWKYCCKVFDLLTLAAIIDGEIFCVHGGLSPEIKALDQIRTIQRNQEIPHKGAFCDLVWSDPDEVETWSCSPRGAGWLFGAKATHEFMTYNSLSLICRAHQLVHEGYKYMFEDKLVTVWSAPNYCYRCGNVAAVLEISNDQKKNPKIFNAVPDHERVIPERHAPYFLVDSASPEETQDAEAATIDELKEHTAHIQKAVAQKESRFILRILRLLPATRKKLNSKLLRKTINGFYTHDKMHREVLLSFVDAEDADTDAAQKSAHLALLPEVDVYLHLLLLVHMIDAANMERAIRCAELLKGKVEAHSRRSMDLLAAKTYFYYSRVYELDGNLSSIRGFLLKRLRTATLRSDFEGQAVLINCLMRNYLHYSLFKQAAKLVSKVTFPEMASNNEWARYLYYLGCIKAIQLYYTDAHKNLLQAIRKAPQHSALGFKQTVYKLAVTVELLLGDIPDRTTFRQPALRKSLAPYFQLTQAVRTGNLGLFNQVLESYGARFQADHTYTLIIRLRHNVIKTGVRMINLSYQRISLADVAAKLQLGSAEDAEFIVAKAIRDGVIEATIDHDKGYVQSAENIDVYCTGEPQSQFDQRISFCLDIHNQSIKAMRFPPKSYNKDLESAEERREREQQDMEYAKEEDDDTFQVAVRLTLALAAMAARTLWQSAVTDMIENFRDSQPLLFEFLARLPEEATAGYIYYTSVLSLCQSALASNLRITAMRTVPANLCITLL-DLTDDHACDAILSFLQHPDGHYPKLMGDLLEQVVKCGPIVETKRACGDQDSIYSLLTGMGEMHTNLILASLLPDSSNRTEMLLKMLLDCVGTPGQYPSEEIISRIPITFWHILLDELARVEPSTQMAKQLQPVYEQLVKMLRKSQLPDPGTMDLDEKEDLRCYRQDIAD--CYMYIATMLPAPVFYFFISALDTAKNAKVIEACLFALNAIGDMADSEDDSPVVGAVLALLPRIPAGDEVLSQVMTAVGIFAEENIGPLVHLLLRGLQQTSASASMALKDLARTHGDRLAPAANDILQAIAVLKHRDRVRLVAIVGHVVSALSSEQALTSLSALMAPFVQQLNEITNLVEQLMPLFKLIAAKY-SCDAEVVSNLAECIRKAVPVLELEVVLSELLSMCGALLLGAATSVDLHGCTDAVESFYKLAVLFKKFSLDDLIQLCPEQFTYRALTQFVNASERSPVKEALENIIAQLVQNIRVSRNFIEAEADVFLALNKTAAALQRFVSSPESRQNFARQILRERSNKRMLCKVI--IDFTLKVKVKWKEVY-DVEVHLPEVFRAQLFALTGVLPERQKVMFKGAILKDS-WGATILMMGTKEELPQPTEKTVFMEDMDDSEISTALKLPTGLNNLGNTCYMNAVVQCFKTVPELTDLAKFTGTITGALRDLYRSMESYSTAPIVLLQALHTMFPRFAEKGEHGQQDANECWTEMMRMLQNLIDQLFGGKLVALQCTESEEESTEDFLQLSCFIS-NEVKYLIAGLKLRM-QETITKMSPTLNRDASYKTSKISRLPAYLTINLVRFYYKERESVNAKILKNVVFPMMLDVYELCSTDLQQKLSPQREKFKKWDNEFSFSEDGSNNSGFYQLQAVLTHKGRSSSSGHYVGWVRREWFKCDDDVVVSEEEILKLSGGGDWHVAYVLLYGPRVLAGLINEKLPKELLLKIFSFLDIVSLCRCAQVSKEWNVLAMDGSNWQNIDLFSFQRDVTYDVVSYIAQRCGGFLRRISLRGCQNVPDQALSVFAQYCHNIEQVLTNCHKLSDDSVVSLSMSLHVDSCVELTDRSLRFF----NRLRVIDISWCRKITGQGIGTIAG--DQLLRFTAKGCLDNEAIIKLATKLQVLNLQCCSFLTDSAVIAVAQNCPDLRHLCVSGCSLLTDASPQALAGGCLHTLEMANCQRCGDAGLAPLLKACHDLRRLDLEECNLITDSTLNHVAAFCPLMEQLTLSHCDQITDQGVHKLAVQCIEIDNCPFISDTSLEYLADHLRRVELYDCQLITQDAIGKFQPEVRLHTYFAPATPRQRYCRCCVIVSSKAARVFCPDHPGANLIEDYRAGDMICPQCGLVVGDRIVDVGTEWRVFQNEKSSNDPTRVGAAENPLLGGSDLSTIIGRTGDASDESGNAKYANRKTMSASDRALIGAFREISAMGDRINLPKTIMDRSNLLFKQVHDGRSLKGRSNDAIASACLYIACRQEGVPRTFKEICAVSKVSKKEIGRCFKLILKALETSVELITTGDFMSRFCSNLALPPSVQKAATHIARKAVEMDIVAGRSPISVAAAAIYMASQASAEKKSQKDIGDIAGVAEVTIRQSYKQMYPKAAQLFPEDFKPANESPWVEKYRPEKFTEIVGNEETVARLEVFSRQGNMPNIILCGPPGVGKTTTILCLARLLLGNSFKDAVLELNASNDRGIDVVRNKIKMFAQTKI---------IILDEADSMTEGAQQALRRTMENYSKTTRFALACNTSDKIIEPIQSRCAVVRFGKLNDAQILAKVIDVCRKENISYTEDGLEAIVYTAQGDMRQAIGNLQSTHVGFGHVNAKNVFKVCDEPHPLIIKEIIEFCSKGDIDEAYTRMKTLYSLGYAAEDIISNMFRVTKSHGLAEFVKLEFVKQIGLTHMTILQGLGSLLQLSSLLANLCLIVQDKKYMKKEHSLVKPYQGSGMNMPNWDFTGTTMVSSNYIRLTRDSQSQQGSIWNKVPWEIQIQFKVYGSGKDLYGDGFAIWYTKDPLQPGPVFGSRDFQGLGIFLDTYANQNGHHNHGHPYISAMVNNGSLSYDHDRDGTHTELAGCEAKFRNSEYDTSVSIRYEHDTLVVSTDIMGKKEWKECFRVSGVRLPTKYHFGVSAATGDLSDNHDVIGIKVFELDEAREFILPQAAPHRDHIDDAMSGTKFFFVVLFSMLFLMFYFYQKHQENARKRFYRDRDAFDEIRDKNCRIQDINELFLPNDTVTHVPNIKQLNIDPVFPNRTNLLHIHNMAISKAFFFSFILQRAKDDEPGFMYYFMSVIADVAANRFINASAIYYAPNMSFTPSYKGFFNKTMPLFAPRAFRSDDFNDPYHLEGTSTLNTIEAIDLGAISLNYSSDQYRINEWYSAWLPDLTKRQDSKTTYTVQITGNNDTFVWHGPPAGNDNPGPVKWVRPYFDCTRSDKWVYGATSPIPDIYPRHTQWRHIEIPRYVAVSVMELDFERIDINQCPFGPGN-PRPNYFAGTSRCKNDTTDCEPVHGYGFRRGGYQCRCKPGFRRPRVVRNPYHGELIERASKHEYENGYQCDKIGYIAVLTQTLNNYMAIGTRIDPLLGGDVVYGKEVQLENEARMAVRLANFVSGFLQIVDPKDLFAEFRVPDKSLTADQMIGEVMSIVIGDQKVVGAGIYFDYKAFFGPY--AWRKGRNERKYFVDDTTKIRIRYNSTGIKYDHFPLQYKAADVGYWTSPYFDCGGYHNSWMVTYAAPFFGWDSLRARLQFKGVVAVIELEQLEINQCNAFENTHKCDRRSSRCVPILGRGFQGGYKCECNQGYEYPYNDPITYFDGQIVEAPSRFERMTHVSGTDKSKYGGRFMVTALPGDGIGPELIGYVKEVFRYGGVPVDFEEVHLDSSRDDVDLLEQAIIAVKRNGVALKGNIETRHNDPNCKSRNVELRLRLGLFANIVHVTSQPGIETRHSGIDIVLIRQNTEGEYSCEEHMSIKGVVESLKVITQSKSDEIARYAFEWAKNNGRKKITCVHKANIMKLSDGLFLRCCTEISKEYPELEFDNIIIDNCSMQLVSNPKQFDVLLLPNLYGNILTNLACGITGGPGIASGRNYGREYAVFETGTRNTGKSIAGKNIANPIAMMNAGVDLLYHLNLREHAEVIATAIDKTINVDKIHTPDLGGQATTTDVVQNIIKEVQKHAMTYSGSSNEVILRDPPTDGISAVKFGQTSNQFLVASSWDGFIRLYDIQGERCRAKFDLGGPVLDTCFQGSSHVWSAGVHRSVRLFDINQGTELKAGSHEDTVRCIEYASDVSQIVSGGWDGAVKLWDPRKPIGPSSHSQDNKVYAIAIAGERIIVGTANRKVLIWDLRNMAFVLQKRDSSLKFQTRAIKAFPDKTGYVLSSIEGRVAVEYLDPSPEAQKKKYAFKCHRMKDSTKIEHIYPVNAIAFHTVHGTFATGGSDGFVNVWDGRNKKRLCQFHKFPTSISSLAFSPDGSALAIAASFQHEYRLEQNPPADQIYIRHVTDQETKTMSCLDQLKELTIVVADTGDFEAMKEYKPTDATTNPSLILQAAKLTQYQALIDKAVSYGRGPCEQLEEAMDKLFVLFGNEILKIIPGRVSTEVDARLSFDKDASINKAVKLISLYKELGVPKERVLIKLASTWEGIEAARILEKEHGIHCNMTLLFNFTQAIACAEAGVTLISPFVGRILDWYTANTDKKAFEPLEDPGVKSVTRIYNYYKKFGYKTVVMGASFRNTGEVKALAGCDLLTISPGLLKELANSNELVPHYLKAENATSIELEKISVDEKRFRWDMNEDQMATDKLSDGIRKFAADARKLEALIQEKLKMGDCVCRPLATVYAEDNEQSGTNVSMPAVFRVPIRPDLISFVHHQLLKNKRTPYAVSKEAGHQTSAESWGTGRAVARIPRVRGGGTHRAGQGAFGNMCRGGRMFAPTKTWRRWHRRVNVTQRRHAAASAISASGVTALVMAKGHAIERVNEVPLVVSDKVQEYKKTKQAVALLKSVKAWDDVEKVYKSKRQRPGKGKKRNRRYKKKCGPLVIYEKDNGIVRAFRNIPGVDTCDVNALSLFKLAPGGHAGRFIVWTESAFRKLNDIFGTFTKTSKVKKGYKLPRPMMTITDLGRLFKSEEIRGALRPKKSIIISKKNKPNPLKKIHLLGRLNPYALVEKRKTILAQQEQKSQRIQKKAALEKVAALKKKRLQSPFPKNRKLNQGAVLKKMLDAIKDLINEGNYDDIVSIYLHQAMDNSHVSLVALNLRADGFDKFRCDRNLSMGMNLTSMAKILKCAENNDIITLKAQDDADTVTFVFESQNQDKVSDFEMKLMNLDSEHLGIPETDYSVVVKMPSAEFQRICRDLSQIGDSVQLTCTKDGIRFSASGDLGTGNIQLSQTADVEKEEEAVIIDMQEAVTLTFALKYLNSFTKATPLSTQVCLSMSADVPLVVELERTFVRYLIISGIYIYDMFARNGSK-LAGIAL---RNFSTSQRNNVKVAVLGASGGIGQPLSLLLKQHPGISYLSLYDIAHTPGVAADLSHINTGSKVKGFVGNDQLKAALEGIEIVVIPAGVPRKPGMTRDDLFNTNASIVRDLADACAQTCPKAMLAIISNPVNSTVPIASETFKKRGVYDPKRIFGVTTLDVVRANTFIAEAKGLDPVSMSVPVVGGHAGITIIPLVSQASPKVDFPQDQLEKLTKRIQEAGTEVVQAKAGAGSATLSMAFAGARFVFSLVSAIQGKNIVECAYVKSDIGDAGFFSTPLLLGKNGMEKNLGLGKLSKFEEKMVTDAMDELKKSVKKGIDFANKEYEWLLKFEVNDIVEQLVIAECSKRFPL-ARSDKFIM-QSKVVATLTGDSISHADISLRL-PKHSQRTIVQNDAQWKLQQIQDAGNHLMQAMNLLRFKFTSGQEVRNLMSDVMTCVGRGRACLVVPKKRTIEEIMQSRNMKSLQPPLPNDVAVSFYIQSYKLVFAVYHV-QKDPQKF-DAECSVPWLSEVLVLFTVALQLCQQLKDKVEVFQYNDFLPMFIPLFLEFNAPISEISDLLGVCDVCFKDAESVL--NGFVSILVCLFCERLESATVQPVAFN--VLYNFYEGLRVDVFCSLLRVAAGSVPEVFQDVSVTKKWLPVDKARQVYRNIHSALSDMPLRVMVELLSTYQDHDAAEANQDAIKCIAFAISDPNTYLMDHLIPLKPIKALENQPINELLKIFVYGKLSEYREFYRKHKEVV-EQLGLDHEKNVEKMRYLTFMYLAEKNQEISFDDIKREVEI--DDVEGFTINVLRTKLVTAKVNQPNQKVIVVSTMHRSFMKNEWEQLREILRLEKVEQSTQQFLEYLG-TR-RKKEVEELAEEAKAIDEGEERAVIEESPQFKELVNILVEWINDELAKHRII--VKNIEEDLYDGQILHKLLEQLTNSRIDVVEMTQNEEGQREKLKVVLERASQALGLKWSVDAVHSKNVVAIVHLLVALARHFRAPVRARLPENVVVSVVSVTKR-EQLTRTYDEYGMKVE-RDAFDQLFDHAPDKLTVVKKSLLTFVNKHLNKINIKDLD-KQFHDGFYLALLMGLLEGYFIPLYLTLTP----------------------NNKVNNVAFAFGLMKDAGLKPKARPEDIVNYDLKSTLRVLYNIFMQ-------------------MLNYGCSVMELSHRSTRIIKDAERDLRELLSVPPTYKVLFMQGGGTGQFAAIPLNLCPADYLVTGTWSRKAAEEAKQYVKYTRVPPASEWNLSPDAAYFYYCDNETIHGVEFPIVCDMSSNILTRPVDISKFGVIFAGAQKNLGPAGVTIVIVREDLVAVASVCPSILAYKVFAENDSLYHTPPTYAIYLLLVLKWIKSEGGVSGMARSAAKSQAIYDLIDQSGGFYCRSRTNIPFRI----RSNDALEKKFLKEAEMIQLKGHRSIGGIRASVFNAMDVVQAMKLYRERPEWQDVTPVLKISYTNTFRDCFGYLRAVISGELSERVFELTTTCADENPACYTVWVLRRRLLEHLKKDLNEEMEFMSRQIFDNQKNYQVWYHRQRLVQLRELDFIERVLDAKNYHAWQYRQWLLKTFNLWSKELNFCSSMLNEDIRNNSAWNQRYFVLKNTTGFVVEEEIKFTLDKISCNESAWNYLGGILNIYLYATLLSEALEIDIPRSPYWTFKQKLLRQLFCAGIALSLTCTLCLVFWLAIIIDRFEELLEFVPHLSRFLTAQGVAYRFIVINQGDRFRFNRGALINIGYLVSRAQCDYMVMHDVDLLPLNPKLSYRFPQGDNVHIAAPHLHPKYHYSTFVGGILLMRHAVFARLNGLSNKYWGWGLEDDEFYVRAKEANVRFERPTDIGTGINDTFRHIHDARRRPRDMVRIGSQREESRKRDRVTGLNNVVFQHQGLYQVRIGEVPVDVHNVHLHCDLTITPWCEKPRMSPGETITTDGYMRGHGGLLSAVAGVVEKVNKLITVRPLKTRYNPEVGDVVVGRIVQVC--QKLWKVDVGARLHAALHLHSVNLPGGELRRKSIEDELMMSQYFIDGDLVSAEVQNVGVDGAVSLHTRNLKYGKLGQGALVTVSPSLIKRCKHLHNLA-NGVHLIIGLNGFIWVTSSKFTREAICRTRNVILCLAIHNIMLYDTSIVHAYDIS-VAQLTRHRADKLVGSWLLTCSGMALGAVFLGGLTRLTKSGLSMVDWHPLNEGRPRTAEWEAEFAKYQQFPEYKVRNKDMTLEQFKSIYWMEYIHRMWGRTVGAAFYIPAAAFWARGYFNRGMKKQIIYLGTLLAAQGLMGWYMVRSGLEEKPRVSNLRLAVHLGAAFFFYGLLFRAALHRLIPSKPLVRFSWATGLVFTTALSGALVAGIEAGLVYNSFPKMADSWIPSDILAFPKWKNFVENPTTVQFDHRLLGETVLTALYIYSRKVPLPPRARLATHAMLAAWLQVGLGITTLLTYVPTPVAVSHQVGALTLLTTLLWLTHELKLIRR-L--------------------------TEKNACQWSKDKLHSLFSDLEIND-SIMSVIKELKKCEGEATANNRKAKLIFFYEWELELCAGS-VVKGRVEIPNLSDENDIHEVSVNVTLVDEKIKGMMRSKGTEVIRSKLDEYVSSLKMDFSQGLILPTKDSGANSTATKGKKISTKELIMEDFKCRAEELYRAFTMVQAFTKGAAGGHFQMLDTNVSGKFLKLNELEFEWRFKSWPAEHYSFVITIEEVSFSLLIL-----LAVRETIEVLH----------IRYDQSQMDPETFLEIANHVSKLKMYPYFELAHCIVTLLYLREDLGTGSQLFSRKHPLSCWVSSMFSIYAGGIFAALLLGEPVLAVLKSNQSLILATACWYLIFYSPFDIVYKFCKILPIKLAIALAKEVTRAKKVHDGVHHAAKIYPSAYIIMVIIGVVKGNGTSFLKVFERLLRGFWTPQAMEIMQPSFATKACVIASLVFVVDKKTDLISAPHSLVYFGVVVFFVYFKLSSVVLGLHDPFIPFENLACAIFFGGIWDAISRAI-----NKGGDAAGSGLKDNKDTAAKKKEMFRPDGIVQRRTAAVIARDREEADRKDGDGCDKDKETRLTLMEEVLLLGLKEKEGYTSFWNDCISSGLRGCILVELGIRGRIDLEAAGMRRKSLLMRKVVVKNDAPVGDVILDEALKHIKETATPETLQNWVDYLSGETWNPLKLRYQLRNVRERLAKGLVEKGILTTEQQNFLLFNMTTHPLVDQNVKDKLIKRVQDSVLAKWVNDVHRMERRHLALLLLSHASDVLENAFNPLSDEEYEMAMRRVRDLLDMDFEAECAKSQTCDIMWGVFAAFVKMADGVPTFKCVLVGDGGTGKTTFVKRHLTGEFEKKYVATLGVEVHPIVFHTNRGAIRFNVWDTAGQEKFGGLRDGYYIQAHCAIMMFDVTSRITYKNVPNWHRDLVRVCENIPIVLVGNKVDVKDRKVKAKSIVFHRKKNLQYYDISAKSNYNFEKPFLWLARKLIGDANLEFVAMPALAPPEVQMDPEWQSKLENDMKEAQNIVLPDEEDDDLLAAQITPILRESKFRESGMLTPEEFVLAGDHLVATCPTWAWAKG-DKSYLPEDKQFLVTKNVPCSKRCRDMEEKIIEGD-EGWVDTHDEDNEDDEGVDMDDFLDEDLETAEILATRTYDLNITYDNYYRTPRLWLTGYDEKMKPLTTEQIYEDISQDFVKKTVTVENHPHIEGVPQASVHPCRHAQAMKNLIQTVEEGGGLEVHMYLIVFLKFVQAVIPTIEYDYTANFNMLSNLERKLPYDKLSANIDIVKKRLNRPFTLSEKVLYSHLDQPQSEEIVRGTSYLKLRPDRVAMQDATAQMAMLQFISSGLPKVAVPSTIHCDHLIEAQLGGDKDLSRAKDLNKEVYNFLATAGSKYGVGFWKPGSGIIHQIILENYAFPGLLMIGTDSHTPNGGGLGGLCVGVGGADAVDVMAGLPWELKCPKVIGVHLTGKMSGWTSSKDVITKLAGILTVKGGTGAIVEYFGPGVQSISCTGMGTICNMGAEIGATTSVFPFNSRMADYLASTNRRAIADAAEQVKDLLSADAGCKYDQVIEINLDSLEPHVNGPFTPDAAHPISKLGQTAKEKGWPLDVKVGLIGSCTNSSYEDMSRSAMLAKQALDHGVKSKSLFTVTPGSEQIRATIERDGQAKVLKEFGGMVLANACGPCIGQWDRKDIKKGEKNTIVTSYNRNFTSRNDANPQTHAFVTSPEMVTALAIAGRLDFNPLTDELTGSDGKKFKLKAPVGDELPRAGFDPGQDTYQAPPADGSNVKVDVDPKSQRLQLLSPFSKWDGKDLIDMVVLLKAKGKCTTDHISAAGPWLKYRGHLDNISNNMFIGAIPEESGEANKIQNRVSGSWGTVPEIARDYKAKGQPWVVIGDENYGEGSSREHAALEPRHLGGRAVIVKSFARIHETNLKKQGLLPLTFADPSDYDKIKSDDKISIVGLNGFAPGMESRGGMFKNTFQSGFLSILYSLGSKPLQIWDKKVRNGHIKRITDNDIQSLVLEIVGSNVSTTFITCPADPRETLGIRLPYIILIVKNLKKYFTFEVQILDDKNIKRRFRASNFQSTTRVKPFICTMPMRLDEGWNQIQFNLADFTRRAYGTNYVQTLRVQIHANCRLRRVYFADRLYAEDELPAEFKLYLPVQHRTAAQALRER---------ERCINSSYQYMQKARADIKRVTQQYRGLAETFIFNNGSSKELVCLDGTIPVRYKYNIPIRIWVLDIHPYHAPFCYVCPTPTMQIKTSQYVDESGRVYLPYLHDWNRNS-SDLIGVIQVMIMIFGEQPPVFSKSPNPYPSSNTLSITDQHIRISLLSAVESRITDRALEKSKAEEEVLRKTNEELQAGKQKLDRYMSDMERDREMESEKSEQLKQLDVDNAVTPTAPLYRQLLQAYAEESAVEDAIYYLGEGLRKGVIDLDTFLKHVREQSRKQFMLRALMQKCRQKAGLPMGRKFYVGGNWKLNGTKQSIQVICDRLKTSQSETEVCVGVPAPYIQFVRDLLPPSIHVAGQNCYKASSGAFTGELSVDMIRDCGGDSVILGHSERRNVFGENDQLIAEKCAFALQGGLVVIACIGELLEEREAGKTEEVVFRQTKAYADLIKDWKNVVIAYEPVWAIGTGKTATPEQAQEVHAKLREWLSKNVSEEVGLNIRIIYGGSVTAANCKELAQKPDVDGFLVGGASLKPEFVDIVNAKKMATALSKKRRFVADGVFNAELNEFLRRELAENGYSGVEVRNGAMKTDIIIMATRTQDVLGEKGRKIRELTAVVQKRFNFKEGTVNLFAEKVSARGLCAITQCESLRYKLIGGLAVRRACYSVLRCIMEAEAMGCEVVVSGKLRGQRAKSMKFVEGLMIHSGDPTNHYVETAVRHVLLKQGVLGIKVKIMHPFDAQGKRGPALMLPDKVTVMVPKYEDDNVETRSDNKDNAA--------GVAEPTTL----SKIEQLKQWSLSTYKCTRQILAEKMGKGTRTVDGELEANIELLRETHQKYLNILRLAKLLTTHFNTVATQAALGECFSDLAQK-------ELQQEFLYNAETQKNLSKNGDTLLGALNFFVSSLSTLCNKTIEDTLITIRHYENARLEFDAYRCEARANFKEKYERLRGDVQIKMKFLHENKVKVMHKQLLLLHNAVSAY-FSGNQSSLEATLKQFNISWLEQ-MVDLVLDRDIRIWVFLPIVVITFLVGIVRHYVSILLTSSRKAELQQVYDSQALIRVRYLRENGKYLPARGFFMRKHFFNDEETGWLK-TQKRAPPMNNPMSDPSMMSEMLKGNLTNVLPMIVIGGWINWTFSGFLTTKVPFPLTLRFKPMLQRGIELVSLDASWVSSASWYFLNVFGLRSIYTLVLGEDNAADSTRAMQDSMVPQAAAMPQDPKAAFKAEWEALEVVDHKWALTGIEEDLCSKPLQF---------MICYKGAASEAGRAMQILKRRERQKEEVELKRQKIEQEMRVS-MGDKFSSHFDAVEAQIKSATVGLVTLNEMKAKQQDAVKEREKRLAQKELEEKQRAEDQKKAQKEKQKKAIQALSFNLDED-DEVEEENGEVYDKCKSDGASKCNSSSSAAGSNQSSGDDSHDNDAGLKDSRESK--IKVKKNPDVDTSFLPDREREEKERMIREELRQEWKDRQRALKEENIQITFSYWDGSGHRRVVDMKKGNSIYQFLQRCLDSLRKEFYELRVVSADQLMYIKEDLIIPHHYTFYDFIVTKARGKSGPLFAFDANEDIRMTSDASKEKEESHAGKVLLRSWYERNKHIFPASRWEPYDPTKIYLEANMSMSKERMSTATKKSVKPMVPETLLKQRKHNAELRQQRLLAAAAKKKAARARRVLAFRRAEQYVREYRRIETSEKNNRLVAKVNGNFFVPDEPKVAIVIRIRGITGVSPKPRKVMQLFRLRQINNAMFVRLNKATINMLRLAEPYLAWGYPNLKTVRDLIYKRGFGRVNGRRVPLIDNSIIEEKLGKYGIICMEDLVHEIYTVGPNFKQVVNFLWHFKLNNPKGGWRKKTTHFVEGGDYGNRETLINSLLRKMIVRLGAEDLWKSVTSVSNAGRKRGRASGHSKKASKDLNRGQKIGSGRINMLWPGLNAPIFRGREVVERQQLPPDPEREKRLIELRDKQQNFRRIRLTPLERGWSGTRAPGRSLGPPDTPEDIDIKDFDSVVLDLRLVSNMTSHLGRVRRHKAVVAVGNGKGLLGFATGRGPDGKTALRKAKNRALLRLCHYPMFEETVLHDFFSEYGCTRIVVKQKERGYGLKCHRAIMSLCKLIGIKNLYAKVDTRNTNLLSLTRAFLIALMYVRSKFDFADEKRLHVVEFRPEQLYFPRIVASPKTVRKEDEIDPYEELDINMIVSEGRMIYARKKAEPFYTRLPGWEVHLKKTDNLKNQRQVRLRLMKKYGALKSFLNIRKPLNQIGPRKSDEKYKWTNKQRLLIFASRGITYRDRHLMNSFRTMLAHSKEECKFE--KKDINEVAEMKNCNKVMYMENRRRSDTYMWLANMQTGPTLKFLVQNVHTMEELKFSGNCLRGSRPFLSFDPAFDSHPLVKEVLAQTFGTPAYHPRSQPFFDHVFVFRLLDKRIWFRNYQIVEE-DGSLVEIGPRFCLNLVKIFDGPFSGAVIYTNPHYVAPNKMRRLAQKE--NKYMYMDVFETKNTFYRMAVGKNKGLSKGGKKGLKKKIVDPFSRKDWYDVKAPSMFSIRNVGKTLVNRTQGTKIASDGLKGRVYEVSQADLQTGEDAFRKFKLVCEEVQGRHCLTNFHGMDLTTDKLRSMVKKWQTLIEAQVDVRTTDGFVLRLFCIGFTKKAQNQVKKTCYAQHAQVRAIRRKMVEIMHREVSSSDLKEVVNKLIPEAIGKDIEKNCIMIYPLHDVHIRKVKVLKKPKFDMGKLLEMHGEGKGSGGGDGMAVDRPDNYEPPVLEDVVKIGIIGGSGLE-DPELLKEDTPYGKPSDA-LISGKIDGIDVVILSRHGRRHTINPSNVNYRANLFALK-QEGCSHILVTTACGSLKEEVRPGNFTPNTFIDRTRIQTFYVCHLPMTKPFLSKLLSEHPKGTVVCIEGPRFSTVAESNVFRQWGADLVNMTVVPEVVLAHELGIPYAALAITTDYDCWR-E--ETVDVEKVSTLKLAADGACRILRATLPKVLKIAVM-------------------------VMRVLRHEEFEKGCLAACNGRYDGFWSKTMVGYGSEDDHFAMELIYNYGVDKYKKGNEFKGIVIQMSNVLKRAQENRWTVQQENDKSYVEAPGGFKFYVEEDSS-ERKDPVRQVIYSCTNVEKTRRFWVTMLGCEVVESGDDFLEVAYDKSKTSLRFEKIFEPIDRGEAYGRVAFACPRNQLPEIEAQVKNADGTVITPLVSLETPGKASVEVVILGDPDGHEICFVGDEGFHELSKEDPEASQVLQEKMK--EYDAYVEKVQRRDRHFRLPPLPTPADLLRFYRLRATKQLSQNFLLDPICSKFVRSSGHVIEVGPGPGCLTRPIFEQGAESVVVIEKDKRFMAALELLANATN-NKLKIVHGDVLNSKLEDLI-PAEKAKPWEGIHLIGNLPFSISTILIIKWLHMISRKSSAWQYGRVRMTLSFQKEVAERITSPERCRLSVIAQGCYVKNGGAFVPPPEVDVNIVRFVPVQPIFQPFDLVEKVLRCLFNKTLKIELAQELLQRSDVDPPVVISVEDYSKICDAYAGLCKEPGLYEYDFRSPRIVLLNRVATTLRLAVGVRLHTSCRLNMPMVPIVIEQTGRGERAYDIYSRLLKERIICLMGSIDDDIASLVVAQLLFLQSESSKKPIHMYINSPGGSVTAGLGIYDTMQYVLPPIATWCVGQAASAASLLLAAGEPGMRHSLPNSRIMVHQPSGGVSGQATDIQIHAEEILYLKRTVNLLYAKHTNQALETIESAMERDRFMNPEQAKEFGLIDTVLEQPP-VREN---MRIERPVGTWLTLLPLWALTTAAPAGSLPDAALFCTGALLMRGFGCTINDMWDRDIDRQVERTRQRPLAAGRLSRWDALWFAGGQGLACLVLLHFNWNTVMLGLASVGLVVIYPVMKRFTYWPQAILAVVFNWGALLGFSATALPMYAGAFSWTLIYDTIYAHQDKRDDLLVGMKSTALRFGARTPLWLGAFSTMTTGLMGWPYYAAITLKIDNIGLLVGCIGGSLLKVARVDQPKTRKGSKILKAREPQVIENPKTSFVKGNNINQRTTQILKDIYALKKSESVFYQQRNPFEDPSPLEKLGKKDTSLFAFGSHNKKRPQNIVLGRTFDRMIIDQFEFGVENFKSLQEFKVPKIGVMLKPILVFSGESWQEMKRLKNFFIDFFKGDYIAVTGLEHVISFTILDILLRSYKVQLKKSGQKSPR-VEIEEIGPRMDLKIRRNKIASDDLFKQALRRPRELKVKRKKNMEQNDLGTSLGRVHMERQDFKLQTRKMKGLKVQILEALDDNYMYLVITREAAVVDPVNPEKVLRAVKVKLTTVLVTHHHWDHAGGNEKLLVFGGDDRIKELTDKDNVNITIGSLAQSLFTPCHTTGHVCYFIVFTGDTLFTSGCGKFFEGTAQQMQAAMLGSLPPETRVYNGHEYTVNNLKYAQHVEPDNQVKEKLLWAQKKPTIPSTIAEEKSINPFMRTSVQKHTDPVSTMNALRQEKDRFRMNRLFGKGKAKEPPPNLSNCIEVLDQRANNMDEKIKKLDAELFKYKEQMSKMREGPPKDLVKQKALRILKQKKMYESQRDNLMQQSFNMEQANFATQQLKDTKTTMEAMRLGVKEMKHEYKKVNLNEIEDLQDDLEDMLEQANEVQEALGRSYGMPEMDDEELEAELTMLNDEIALEDTSYLEDVTAPKVPSKGPSQKQKAEGGIMVDEFGLPKKEAQVRMVSRTRGKATLPDLPYDYNALEPVICAEIMTLHHSKHHNAYVTNYNIAAEKLQEAIQKNDTNAQIALQNAIRFNGGGHINHSIFWQNLCNPKSGEPSAELLAAIKKDFGSFEAMKDKMSAAAVAVQGSGWAWLGLNKANKQLQVAVCPNQDPLEASTGLVPLFGIDVWEHAYYIQYKNVRPDYVKAIWKVANWKDISQRFIKA-DDHIQHDVIKEALESGRDLREYSRSVEQQLKGSEDEAIRDYMHNCRDIAALHGEIASCDAILQRMESILRGFQNDLGSISSEIQSLQRQSVAMNLQLKNRQAVRGELSQFVDDFIVPEATINVILDCPVIDEDFLTQLALLDQKISFVKVQSFKEASSCQDVKDILDKLKVHAVTKIREWLLQKVFSFRKANANFQLAQNTMLKHRLFFQFLATHEREVAKEIREEYVDTISKVYLSYFKAYQTRLMKLQFEDVPDKDDLMGVDDTPKWGLFNKPSLKNRSTIFTLGSRNAVLGADLEAPVIVPHASAKSEKHYPFEQLFRSTQYALCDNAAREYLFVSEFFLLTKQGAVDMFDNILEKSMSLFAKHTESFVAECFDSIALFLCIHIIRKLKVVMHSRNVPVMDRYWDLLVKIIFPRFETILRMNIASIRDCDPSKLGSIDNRPHYITRRYAEFSAAIVSINENHPDERVTQLLGQLQIEVENFVLRMAAEFNGRKDQLIFLINNYDMMLGVLQQRTHEDSKETTNFRTLLTARQSEYVEQILTVHFGGMMTFIKESEYYVEKGQINELEKESHKVATLVRNFNSGWKKAIDDMNGDIMKTFTNFKCGTSILQEALKQLLQYYHRFNKVISQPPLCSLPVRSELINIHHLMVDIKKYKATFMNLAVTAVCLLVNVVVD---GYFIVVDAHSEECFHDRVVKGTKMGLTFEVAEGGFLDIDVKITGPDEKVVYNGERESSNKYTFAAYMDGVYKYCFSNAMSTMTPKTVMFTMDIGEEPKADGK---DGGENKLEDMISELHTAMTGVKHEQEYMMIRDRIHRSISESTNSRVVIWAFFENLIIIAMTLGQVYYLKRIFEVRRVVMRLSHILKLPKDYANLPKSYVKRAMAQVEWRTPNGRQFRRAVVQKPYTMNRPWTNEFMTNPPGVAIPVQVMFRGDRVEVMVGDDKGKQGTVNYIVPERNWVTVEGLNTEELPLLMGTQVKLVDPVDEKATDVEWRYTESGERVRVSVRTGRIIPIPLAYETIDYKYVDKAKDTSATELEKITFVP-KLATFEMDIMEQHGIQDDRVPHRTFWY--MNAEIKIHVDLVEQKMELLLWLSRLACTMFTVSYLLGNPYSLYQKALICNGVTSALRLHQRAPTVRLNAEFLTTILVEDSCHYLIYSLIF-LPIS-MVLLPPMLFAVLHSLQLLERAGILLQ---SRVRVFRLIAMTEIFLMPTVIIGLFCGVSLMAPFMYYRFLKLRYASMRNPYNRNVFYELKLHANQLAAPNCPMIIQRVRFIESRAGLAIAVITVYLSFPEMRPEEQPYVKLPTSLEDAKNLGRVLSRYTDDAVVLAFFCTYIFLQSFAIPGSIFLSFLSGFLFPFLLAIFLVCLCSAIGASLSYLISYSVGSRLILAWIPNMVYYIIFLRITPFLPNWFINLASPIVDVHIVPFFIGTFIGVAPPSILAIRAGISLQQLASALTWENIGLLTGFAAFSLLPVLLKLKNKFEEVQDKLTRIAIVSIDKCKPKRCRQECKKSCPVVRMGKLCIEVTPNDRIATISENLCIGCGICTKKCPFEAIQIINLPSNLERDTTHRYSANSFKLHRLPTPRPGEVLGLVGTNGIGKSTALKILAGKLKPNLGRYNDPPDWTEILGYFRGSELQNYFTRILEDHLKAVIKPQYVDQIPKAVKGTVRELLDRRNDLGKEDYLCELLELVNVMDRQIADLSGGELQRFATAMVCIQKGDIFMFDEPSSYLDVKQRLKSAQAIRGQIEATKYVIVVEHDLSVLDYLSDFICCLYGTPGCYGVVTMPFSVREGINIFLDGFVPTENLRFRESSLVFKVSDNTDEDVKRLARYEYPSMVKKMGDFELKVQGGSFTDSEIIVMLGENGTGKTTLIRMLAGRLKPDGDEDVPTLNISYKPQKISPKSTGTVRFLLHEKIRDAYQHPQFVADVMKPLLIDNIIDQEVQNLSGGELQRVALALCLGKPADVYLIDEPSAYLDSEQRLAAAKVIKRFILHAKKTGFVVEHDFIMATYLSDRVIVFDGKPSVSTTANMPQSLLVGMNRFLELLNITFRRDPNNFRPRINKLNSVKDSEQKKNGTFFFLEDEEDGEDLFGPELDRYDAAEEEMRRRDRGRLPRGLESIENLEDMKGHTVKEWVTQGPKTEIFNRFKNFLRTYKEKIRAMEQNRMSLEVDYTLAQSEQVLAFFLPEAPAEVLPIFDEAAKDIVVGMFPHYGRIHHEIRVRITDLPILEEIRTLRKIHIDQLIRTSGVVTSTTGVLPQLRMVKYDCVKCKYILGPFVQSQEVKPSSCPECQSTGPFSVNVAQTIFQDYQRVTIQESPGKVNAGRLPRSKDAILLNDLCDSCKPGDEIEITGIYSNKFEGSLNKANGFPVFATVIIANHILKKDTDEDVKEVVKLSKEELAERIIASIGPSIYGHDDIKRAIALSLFGGVSKNPGQKHRIRGDINVLLCGDPGTAKSQFLKYVQQIAPRAVYTTGQGATAVGLTAYVSPVTRDWTLEAGALVLADKGVCLIDEFDKMNDADRTSIHEAMEQQTISIAKAGIVTSLRARCTIIAAANPIGGRYDTFHQNVNLSDPILSRFDVLCVVRDERLARFVVDSHSRHHPISQDLLQKYILYAREKIEPKLDQDKISQLYSDLRRESMATGSMPITIRHLESIIRLAESHARMHLREHVDVNTAIRVMLDSFVTQKFSVMRMGLLDDVKEIFGADTLYEVLGVKKSVRSDLLKKAYRQKSLLCHPDKANEENKEEFTKKFQILCKCYEILCDEEKRKIYDETGSV--DDSFDEKKDWKSFWRTLFPTVTDSQIQDFFDKYHGSEQEREDLKRAYEKTKGDMNKIAECFIGYDEDRLCSLLQKMIDDEEIPEYRAFTKETASSKAKRQKKFAREAKEAEK------NEMSSLELIL----KREGDDFLAALEAKYSKPK-KKLARSKK-TLLLAIAIGFLLLGKVGTKKLRKLEEKAEKRRLRELELQEREERAQKQAEDEYRKKVEQKKEDEQKQKEEEERRQKEEKERREHEEYLKMKAAFAVEEEGFDQEDDP-NSSQKLHEFITYINEHKVVQLENLAAQFRMKTQDCIDRVQRLLEDESLCGVIDDRGKFIAITRDELDEVARFIKVRGRVSIQELVENSNRLINLASDIMKQQEEAKAAALKHVVTMLQIPDQLDKVEQHRKRVQRKKASVEAMLKTAVQSQLEGVRTGILLLAAARDDVNDIQKDEADTIYDLSKLVQLQDVREESFRHSQTGTLMEHLKHIFNVPGSITRTQDLIQDGNLLLAHKLSDLECSRDELLYELHKQANNQASDRAMLKQYFADVERLSDELAKQLWLILKRTLNTVRKEPQVIVTALRLIMREEWAALKRQQST-GFLPPGRPKLWRKKAIETLEQSVAERLEGNQIEVRSENNMWLVRHLEVTRQLIIEDLKTVKHHCTPCFPPCFDIFNEVRMTHNCLSQRLQTIISGLVDSEYIHVLQWLNTYNSRDLMGHPDLHVDVALLPDTIERLMERYLAGLRTKFEEWLRNALSDHKDWPEQDSNGYYRTETPMLIYQMITQHVEVARTVDLVSRVLRLAMNRMDYFLSQYNQLVTDYNFEDRSYTAYMIAIANNAVNMK-LRELKDNSLDYLCEEVLTDIKVMPDIMTETVTVTLADYGGDYIEKEVAASYVKAIC--EKRMSFRNYEERRAAAELEKLKKSKLDVLKLMAEVLKMKDSSLLSLEMNGLLFLLVFCLVW------DLRKGDTPTRVEEDIGKIKEGSRTDAEVIAREEEAIKVDSLSVAQLKEIREKAQKHAFQAEVARMMKLIINSLYRNKEVFLRELISNASDALDKIRLLSLTDPDALKKLQDLSIRIMADKENNVLHITDTGIGMTKEDLMKNLGTIAKSGTAEFLQKVSEGSSGKDLNDLIGQFGVGFYSAFLVADRVAVASKHNDDPIQHVWESNAAEFSVADDPRGDTLKRGTTVSLYMKDEAKDFLEHDTLKKLIEKYSQFINFNIYLWSSKT-VTEEVTESEKPAEE-----ADDDEAKVEDAKEP--LKTKKVDKTVWDWELINSAKPIWTRKEKEVSDEEYNEFYKAVTRDSQNPLAKTHFTAEGELTFKSLLFVPSKQPQDSFNRYGQKTDHIKLYVRRVFITDDFQDMLPNYLSFLRGVVDSDDLPLNVSRENLQQHKLLKVIKKKLVRKALEMFRKISEEEYVKFWKEYSTNIKLGVIEDSANRSRLAKLLRFPSSLDSTDKLVSLGDYVQRMKEKQTAIYYIAGNGMDEVKKSPFVERLLKRGYEVLYLTEPVDEYAISSLTEFEGKKFQNVAKEGLSLDD---NKDIREAMEKEFEPLTKWLSETALKDKISKAVISERLVETPMALVASQFGWTGNMERIVAAQTHMKENDPQKSFYMTQKKTLEINPRHPLIKELLRRVDDSPSDEMAKYLTEMMFETATLRSGFQLNDNAQFASNVERMLRKMMGVSEDAQVDAELEEAELPEVNPSDKDEDIESEPHDELGRRGEVKPLSAQEMKMIVISEIIQELVQAHNDKRDVNLNRVKYDVSARYGLKSQPKLVDIIAAIPPQFKQILLPKLKAKPVRTASGIAVVAVMCKPHRCPHINYTGNICVYCPGGPDSDFEYSTQSYTGYEPTSMRAIRARYDPFLQTRHRVEQLKQLGHDVDKIEFIVMGGTFMSLPESYRDYFIRNLHDALSGHTSANVDEAVKYSERSKTKCIGITIETRPDYCLQRHLTDMLRYGCTRLEIGVQSVYEDVARDTNRGHTVQAVCETFHIAKDCGFKVVTHMMPDLPNVDFERDVLQFVELFKNPSFRMDGLKIYPTLVIRGTGLYELWKTGRYKSYPPALLVDLIAKIFSLVPPWVRIYRVQRDIPMPLVSSGVENGNLRELALARMKDLGLVCRDIRTREVGIQEIHHKVVPYHIELVRRDYVANGGWETFLAYEDPQQDILIGLLRLRKCTEQTYRPELLGQCSIVREHVYGSVVPVHSRDPSKFQHQGFGTLLMEEAERIAREEHCSKKIAVISGVGTRNYYRKLGYELDGPYMSR

>Dermanyssus_gallinae

PGSKVNTLSQEVMASAVLISGKKGCFIAGADITMLEKCKTEAKKLKPVVAAIMGSCLGGGLETALACRYR-IAVEDPKTTLALPEVMLGILPGGGGTQRLPLYVQLPTALTMMLTGQNMRADKAKKAGLVDVTVKPLGPGLYLEEVAARDIASGKLKIRVRPLSERLVRNT-IFNKAKAQVMKNTNGLYPAPLRILQVVRTGIEKQNFAELCTPECKSLMGLYHGQVQCKKNAFGQPTQNVAVLGAGLMGAGICQVTLKDFNNVIMKDGLVRGHNQIKKKKIEKDKLMSKLLPTLDYSDMVIEAVFEDLAVKHKVVKEVEAPHCIFASNTSALPITKIAEASKRPEKVIGMHYFSPVDKMMLLEIITTDKTSKDTAAAAVDVGLRQGKVVIVVKDGPGFYTTRILAPMMSEAMVLLMEGCQVKELDKLKDFGFPVGGATLLDEVGVDVGAHIAESVFGDRKDMVSNFLGRKSGKGCYIYRPVNPLQRKYTPEQLQFRLATRFINEAIMCLQEGVLANPVEGDVGAVFGLGFPPNRGGPFQFVDTYGAAKIVDMKQFQDFQPCQLLLDHA-KDSSKKFHMVTIVIIDPAKTLKILGYTCMLYNIEASFSICVFIDFVLLASESAKLPLRTPATALTRKENAKITNIQDGAYACVSLQLNIVISSISVVSSVETDLVASKKEFFAKMKCLLQTCLIRDTIAGTLASAIYSWLYQYNKLYYGVGSNDQRISFELQSLFTLLYIYRDNRKQYTSYETSQASTRITMHALKILSGIKKVSGGALEDSMLVAGVAFKKTFSYAGFEMQPKQYTSPKIALLNIELELKAERDNAEIRVENVEEYQKIVDAEWNILYDKLAKIHASGAKVVLSRLPIGDVATQYFADRDMFCAGRVAEEDLHRTMKACGGCVLTTVQDLKNENLGSCEHFDEVQIGGERYNIFKGCPNSKTVTMILRGGAEQFIEETERSLHDAIMIVRRAVKNDAVVAGGGAIEMELSKYLRDYSRSVAGKEQLLVAAFAKALEVIPRQLCDNAGFDATNILNRLRERHAKGEKWSGVDINAEDIADNLVACVWEPAVVKVNALTAATEAACLILSVDETIKAPQSNTDPSAGRPFMFHIHNRICLHEQALDRTRLRRSVNRLRLPAQTAVVSPKVCSDCCRPSKTARAVAAAAATTTPRTVEMLRTVSASAVVKLLEHMATSDQKNTVAEQNRTFIMSPIMEKMMPSTFIMASKHMDNHIASLLSAYKGCQIVQISDYSKVYYYLVVVVVVVAEEVVNEVKRNQCERAFREHGMAYAEIA-------GLVKRAGKSVMDDNFAIVFAAMGVNMETARFFKQDFEENGSMDNVCLFLNLANDPTIERIITPRLALTTAEFLAYQCEKHVLVILTDMSSYAEALREVSAAREEVPGRRGFPGYMYTDLATIYERAGRVEGRHGSITQIPILTMPNDDITHPIPDLTGYITEGQIYVDRQLHNRQVYPPINVLPSLSRLMKSAIGEGFTRKDHADVSNQLYACYAIGKDVQAMKAVVGEEALSPEDLLYLEFLTKFEKNFISQGRYENRNIFESLDIGWNLLRIFPKEMLKRIQHSLLAEFYPRTDAKRCLTTARFND-TGLFGEPTGFYVLKDNAIQQAEELVAEATSRKRKMVHIFDDLSDCLCRVADLAEFVRVGHPQGRYNDAAKHASLAISSLVEKLNTNLDLYSALRSVVENG-DISASSTEKHVERLFLFDFEQCGIHLDEQRRQEVVALNDLILHVGGYFLQNSQNPRYVPKSMVPENILLVNGLQADCSNELVRQAAYRMYLSPDDQQQKLLDQLLEARHRLAQLCGFETYAHRVLKGSIVETPENVTFLSYLSAELKPRAERDYQEMLTMKAWDIPYYTAYLSLASCMEGLDMIFHELYGVRLEVLLLITHSSRMLLIT--SSSPMGVIYCDLFERPGKPHQDCHFTIQGGRRSDGTYQTPKVVLMLSLPPPLLTPPVMDNLFHEMGHAMHSMLARTEYQHVTGTRCATDLAEVPSILMEYFASDPRVVLRFARHYETGRPMPSYMATSLEASRVVFQASETQLQVFYAFVDHEYHSKYPLSTTEILRNVQNKHFGVKYVDDTAWQLRFGHLVGYGAKYYSYLMSRAVAASFWHRVFKADPLSRSSGTYRNEVLAHGGALPPAQLVQDFLLAESLIADIMPSDAEV---GPNRDLAKARQDK-AANGISDEEKFALEMDELLVVLTMTKRCSTVRGIFSLSRIARYRALSYRYVAVTGIASSSLSKGCGIKNYLSTANILGISSLVKIRQREVVPHPDLTHLLHLERQIHQSLIYAFIRVRALHRGREALAELVASLHHHRVVPSQQQCRSTGSRFDEAMRLNAAARAVTGIVHLAWSGLYPGDAAQEVQGLFRMRIALARALYVKPHILLLDEPTNHLDLDACVWLEEELKTYSRILILISHAQDFLNGVCTNIIHMNLRKLEYYGGNYDQFITTRAETLENQMKRYNWEQAQMSHMKDYIARFGHGSAKLARQAQSKEKTLAKMVAGGLTEKVVNDKTVNFYFPSCGPIPPPVIMVQNVSFRYSDDTPLIYKNLEFGMDLDTRVTLVGPNGAGKSTLLKLLCGALVPTDGIIRTHSHLKIARYHQHLHETLNFDLSALEYMLQCFPDVREKDEMRKIIGRYGLTGRQQVCPIRQLSDKQKCRVVFAWIAWQVPHMLFLDEPTNHLDKETIDALADAINNFEGGMVLVSHDFRLISQMAKEIWVCENQTVTKWAGDIKSYKQHLKTKVLKDMVLMVLADLGRRITGALRSLSTATVINQEVLDSMLKEICAALLESDINVRLVKQLRENVKAAIDIEEMAVGLNRRKVVQSAVFKELVKLVDPGVKAWQPSKGRNNVIMFVGLQGSGKTTTCTKLAYHYMKKGWKTALVCADTFRAGAFDQLKQNATKARIPFYGSYTEVDPVVIAADGVSKFKAENFEIIIVDTSGRHKQEDSLFEEMLEVSNAVSPDNVIFVMDASIGQACELQARAFKEKVDVASVIITKLDGHAKGGGALSAVAATKSPVIFIGTGEHIDDFEPFRVKPFIQKLLGLGDIEGLIDKVNELKLDENHELIEKLKHGEFTLRDMYEQFQNIMKMGPFNQIMGMIPGFSADFMSKGNEQESMARLKRLMTMMDSMTDEELDDRDGAKLFAKQQTRITRVARGSGCSTYEVHELLNQYTKFAAMVKKMGGMKGLFKGNDLARNVNPAQMNKLSAEMAKMIDPRVLQQMGGFSGIQNMMRQMNASVLTEDPAYLKLQQLYESKGKTLRLNDLFKADATRAEKYTLKLSEGPETLLLDYSKNLVDGEILAALFELAKNRQVEGMRDRMFAGDKINFTENRSVLHVALRNRSNRPIQVDGADVMPGVNAVLEHMRTFSLKVISGDWKGYTGKKITDVINIGIGGSDLGPLMLCVILGYFFVGPRVHFVSNVDGTHLFETLKKVDPETTLFIVASKTFTTQETITNAESAKAWFLGKASDKAHVAKHFVALSTNKAKVEAFGIDAANMFEFWDWVGGRYSLWSAIGLSIALFVGMANFEKLLAGAHFMDEHFRTAPLDRNAPMILALLGIWYINMFGAESHCLLPYDQYLHRFAAYFQQGDMESNGKYVQRDGTRVAHQTGPIVWGEPGTNGQHAFYQLIHQGTKLIPCDFIAPAKTHNPVQGGLHHKILLANFLAQTEALMKGKSSDEARTELEASGLSGDTLNKILPHKVFQGNKPTNSIMVQQVTPFTLGALIAMYEHKIFVQGVIWDINSYDQWGVELGKQLAKVIQPELDGKDPVSSHDSSTNQLINFIKSFN??????????????????????????????????????????????????????????????????????????????????????????????????????????????????????????????????????????????????????????????????????????????????????????????????????????????????????????????????????????????????????????????????????????????????????????????????????????????????????????????????????????????????????????????????????????????????????????????????????????????????????????????????????????????????????????????????????????????????????????????????????????????????????????????????????MDS-TVSVLWKYSIHNI------PLKHKRCAIFRSIRGNFFLHKKYTVAPIYRANPHDMCKFHSEEYVNFIERVTPKNIQTFSKSLTHFNVGDDCPVFDGLYDFCSMYTGASIDGAWRLNNKTCDIAINWSGGLHHAKKFEASGFCYINDIVIAILELLKYHARVLYIDIDVHHGDGVQEAFYLTDRVMTVSLHKYGAYFFPGTGDMYEVGAESGKYYALNVPLKEGIDDASYYQVFKSVISSVIEHYKPGAIVLQCGADSLAGDRLGCFNLSIKGHGECVRFVRNLNIPLLVLGGGGYTLRNVARAWTNETAILVDEQVSAEIPYNEYLEFFAPDFSLYPD-------ENANSKQYLEAIIKYTTENLRCLDHAPSVQMQDIPPDLADLGDDQVKRETTKEHPGEFFD---------------MSDAEEDYELEYSEDDDSQPDVDLENQYYNSKALKEDDPQAALQSFQKVLDLEGGQKGDYGFKALKQMVKINFQLGHYEEMMARYKQLLTYIRTAVTRNYSEKSINSILDYISTSKQMQLLQEFYEVTLDALRDAKNDRLWFKTNTKLGKLYLDREDWQRLSRILRQLHLSCQNDDGSDDLRKGTQLLEIYALEIQMYTGQKNNKELKKLYEASLQIKSAIAHPLIMGLIRECGGKMHLREGEYNSAHTDFFEAFKNYDESGSPRRTTCLKYLVLASMLMQKEINVLDSQEAKPYKDDPDIVALTDLVDAYQAHDIGRFEAIVSPHKESIMKDAFIKEHIEQLLLNIRRQVLIRLIRPYTRITISFISRELKIPSADVESLLVSCILDNTIHGRIDQVRQVLELNP-SPIDRRYAAMEKWSAQVQLIINTVCHKL???????????????????????????????????????????????????????????????????????????????????????????????????????????????????????????????????????????????????????????????????????????????????????????????????????????????????????????????????????????????????????????????????????????????????????????????????????????????????????????????????????????????????????????????????????????????????????????????????????????????????????????????????????????????????????????????????????????????????????????????????????????????????????????????????????????????????????MAASINPSGKPQLLCRLEGHSDAVNQVALIGDADAVISVSEDRTVRVWARRDTGQYWPSVCHTMPSAASALDYEPTSRRLFVAMDNGSITEFELADDLNKITFKRSYIAHQQRVTSVKFSHITGWLLSAGKDKYFQWHCTETGRRLGAFQGSAWCTTVELDSASRHAFIGDYSGHVTMLKLTETNYQPVTTLRGHSGSIQALLWDERRQLLISGSFDQVIIVWDIGGGKGTAYELSGHRARVTGLALFGPSSLLSVSDDASLVVWDIGAQRQETPEWSERDCCERCARPFFWNVRAMLDQKQVGLRQHHCRRCGRALCDRCSANRSTLPRLGFEFPVRICDECHLHISDRDRQSLATFHDLKAPVTAMTLLQPAKIMATVGSDRSIKIWDLSKVL--------------------------------------------DVSAGLYGKYPLIQSKEKIDREILKVCQCTPKFADQIVWLRGRLHTSRAKGKQCFFVLREQHFTLQCLLSVGETTSKQMVKFVANVTKESLVDVQGKLVKSPSRIESCSQQEVELQVHQFWVVSLSEARLPLQVEDAARPECADDDEEALKIRVNQDTRLDNRVLDLRTPANQAIFRLQAAICHLFRESLNRRGFTEIHTPKIISAASEGGANVFEVTYFKGKAYLAQSPQLYKQMAIAADFDKVYTIGAVFRAEDSNTHRHLTEFVGLDLEMAFKYHYHEVLDTIADMFVDMFKGLRDRFQPEIEAIGRQYPAEPFKFLEPTLRLEYAEGVAMLRAAGVEMADDEDLSTPNEKLLGRLVKAKFDTDFYVLDKFPLAVRPFYTMPDPTDMKWSNSYDIFMRGEEIMSGAQRIHDPEFLTERAKAHAIDLSTIQSYIDSFKYGAPPHAGGGIGLERVAMLYLGLDNIRKTSMFPRDPKRLTPALDEGLPVEQIDPLEFSRVRIMYPKECRTRRVTYRGKLDVTFWWVNGLKQEPIKRTCGEIPIMVRSRKCNLYGLNPKEIVARGEEMEEFGGYFIVNGNEKVIRLLIMQRRNYPIAMARNGWKNRGSMFSEFGVSLRSCRRDGQNMVLHYLINGTVQVMLTNRKEIYFIPVILLLKALTDKSDYEIYKALTKSCEKDTFYKGCISNMLRLVQENILTQDAKEFIGSNFRWASNADVCDKLLDKCVCVHLDNNEDKFNLICFMVRKLFAVAKNKCAIESADSTMNQEVLLPGHIFLGVLKEKMEGILTGVKLSIDKKMSSALDLTNGMNYFLSTGNLVSKTGLGLQQTSGFTIIAEKLNFWRYLAHFRCVHRGSFFMEMRTTTVRKLLPEAWGFLCPVHTPDGGPCGLLLHLTAMCEMVVQLDGKCIGWVLRYFKASGNIPSTLEICVIPRTGTNSLYPGLYLFSTPARMMRPVLNRARCVEWIGTLEQVHMDICVVAEEATTHQELRETAMLSVLANQIPWSDFNQSPRNMYQCQMGKQTMGSPMHTFRNRADNKLYRLYPQSALVRPTAYDFYKMDEYPSGTNAIVAVISYTGYDMEDAIVRTQSICERYLLKMIQITYLVDRTLNLFGIYLIISFEHRPCCHKTTKINRYRNS---FEINSSVDGRF--IGLVYYQLRHMVADKFQASAHQRYRIKIVQPYLDMVCAECGTLLQVLGCGNGFEFRLELCPEVNTSTAASCSHEGLRHINDRFYININNFSGVYYEPINSNHGRNYLGTHVRSSCINGKLVEVDVAAEILLYLCRRIISRDTNDAAGSGPLNIFVEVMVQDYRRCNYPTYVEMLRSSRLASTSLFILVIHLLSLLLLSLVMRCRLVLLVELKRYHIYTVTL-LTSVAVKLLRETSRDLHRHQRNEPELHPMATAREYTAAVNAAKLDRIFAKPFLCSLGHRDAVELLAKHPERISGAVSASNDGELRWWDLTHRKCVRALQAHDGPVRGLAGQDQSIKTILSKLAHHYSTTFATAGETVSLWEERNEPLRNLW-GIDTVYVTFSPIESLSSDRSIVLYDIREASALRKVVLEMRSNALSFNPMQAMHFTVANENYNLYTFDMRNLRRALQSHTDHVGAVLSVDYSPTGTEFVSGSYDKTVRIYRSREVYHTKRMQRVTSVLYSLDAKYVLSASDEMNIRLWKAKASEQLGVQNYNDTLLQRFGQHPQVKRIIRHRHVPKSLHQEKQVMLASRKRK???????????????????????????????????????????????????????????????????????????????????????????????????????????????????????????????????????????????????????????????????????????????????????????????????????????????????????????????????????????????????????????????????????????????????????????????????????????????????????????????????????????????????????????????????????????MLVLFESPAGFAVFKVLDEKKVQKTENLFREFEDASGAAKILKLKHFQKFDDMTQALSAATAAIEGKLCKPLKKVLKKLVASDAHETLAIADAKLGSIIKEKMDISCVANSSIQELMRCIRSQQEALITGLSQKEVTAMALGLAHSLSRYKLKFSPDKVDTMIIQAVSLLDDLDKELNNYVMRCKEWYGWHFPEMSKVVTDNMLYVKTVRKMGMRSNAINMDLSDILPEDQEAKIKELAEVSMGTEIAPDDVENIKHLCDEVIQMTDYRATLYEYLKNRMAAVAPNLTVLVGELVGARLIAHAGSLLNLSKQPASTVQILGAEKALFRALKTKHDTPKYGLIYHAQMVGQSSQKCKGKASRWLAAKAALAIRVDALGDDADTEMSIRNRANLEARLKMLEEGKLTRISKGGSGSNKFDVYRHKSEVLEYKPAQDSTLA-GKKRKFNDDDEDDSEPKQFKPKVDELAGASD--QQAEEE-------------------------VVEKKKKKKKKSKVPEEMEVAEEAEEQEVSK--KDKKKKKKKSKLPPFLWPKNNVKLQLAVLVCFATLVLGRVLNPISPIMQKKIVDGLSASTKITI---MVQGSNSWLTNLRSFLWISIQQFASKTTQIGLYSHLHSLSLGWHLSRKTGEVLKILDRGVASVDALLSYFLFQILPAFADILIAFGYFTYAFNWFGLIALSCAVSICITEWRIKFRREMNQLDNAAQAKGVDALLNFETVKYYNAEEIEISKYQKAEWKSSASLSLLNVVQGSILTTIGSLLCAYMTAGDYVLFLMYSAQLYTPLNFLGTYYRVIQRSFIDMENMFELLDVVDHHLKV--EIEFRDVCFRPILKHVSFVVPHGHTVALVGHTGSGKSTVLRLLLRFYDVSSGSILIDGQNIAGVSLRSAIGVVPQDTVLFNTSIRENIRYGRPSDEVELAAQAADMHQSIVQMYDTVVGERGLKLSGGEKQRVAIARTILKAPAIVVLDEATSALDTRTERHVQKALAVVQNRTSVVIAHRLSTVVNADQIIVLEHGEIVEQGHRELLTKKYAAMWFAQLEFILRRQLNHLARTFSQTLFNMPETRVTTLANGVRVASEDNGAPTATVGIWIDAGSRYETEKNNGVAHFLEHMAFKGTGKRTQTQLELEVENAGMHLNAYTSREQTVYYAKCLRKDLANAVDIVADITQNPKLGEQEIERERGVILREMEEVEGNLQEVVFDHLHAVAYQGTPLGLTILGPTKNIKSLQRQDLKDYIDTHYTGSRIVLACAGGVDHDELVKLAEQHFGKVGTGFDQQSPCRYTGSEVRVRDDDMPFAHVAIAIEGAGWTNPDNIPLMVANTMIGSWDRSHGGGANASSRLAAVAATDRTMHSFQSFNTCYKDTGLWGVYFVANGDELDDCMSAVQNEWMRICTECVEADVTRAKNLLKTNLLLQLDGTTPLCEDIGRQMLCYGRRIPLHELEARIDAVTANTVRDVALKYIYDRCPVVAAVGPVSGLIDYVRIRSQMYKHSVHTLVFRSLKRSHDMFLCDEGALPPVDDRAHRLRVATKARDDYGPVMHLVSEGQRASATGALMLAGSQLTAQLKKPSIPKPQWHPPWKLYRVISGHTGWVRCVAFDPTNEWFCTGSNDRIIKIWDLASCKSSDSPATTVRCLHSLHARCRYCRRCCCCCGWQVKCWDLEQNKVIRHYHGHLSGVYTIGLHPTIDIIVTGGRDSTARVWDMRTKANIHVLGGHTNTVASVLVQATEPQVVSGSHDSTIRLWDIVAGKTRVTLTHHKKSVRALVLHPKLNMFASGAPDNIKQWMCPDGKFIQNLSGHNTIVNCLAMNDDGVLVSGGDNGSLQFWDWKTGYNFQKMTTPVQPGSIDSEAGIFAMGFDLSGTRLVTCEADKTIKVFKEDENASEETHPINWKPEIVTGSTSEYLINCSAEHYLEALGINVKAKNFLVFQGAVESIAMKNPKERTALFEEISRSLHRSSIDIAQRASYISELRCDHKAEEMMKYQKLK--VHHLLAIEGCGHQAEELQKKRRKEKIENELKEKKKVEQTHRDDVELNKRKPAYIKAKEKTTHMQKKLDAAKKSLEAATKTHKSHQGIEELEHELAQVEEAFEQEDVSLEESQVKEYNRLKEKAGKMASAALQEYDSVAREQKTDQDHLDNELRKRNECEAKLKELEENQRRVNKLVDLHDLKEEEKKLEAKRRLTEKFEDVASLGDAKVDKHEDTRRKRKSEIVEHFKKLYPGVHDRLVNLCHPIHKKYNVALTKVLGRNMEAIVVDTEKTGRACIQYLKEQMLEAETFLPLDYIDFKPLKERLREFKDVPNVKLLYDVLKYEPLSIKKAVLYATNNALVCETAEDAAKVAFQSPDGKRYDAVALDGTYYQKNGFISGGSSDLAKRAKRWDDKDFHKLKGEHTKLQEELREAMKTARKESDLTTIESQIKGLETRIKYSKVKQLEHSMREREVRINEIKSRQNTVEDDVFHDFCEQIGVANIREYEERELHASQEREQRAELENQKNRIASHLEYER--TKDTLAVEQDQQELARLKDIEQKQKELIEQQMEAISALKNERQSKKMKVDEIDEEVAEIRKRLTAQQKEVTSVQKTVTQAEARLEQKRSERHTLLQSCKLEGIRIPLIRGSMAQMYEREALQIDYSQLRLEKEINLQRIQAPNFKAMEKLDSVKERLKDTDTEFEHARRKAKSNFELVKRERTCFEHVSNCIDEIYKSLTNNPSAQAFLGPENPEEPYLEGINYNCVAPGKRFQPMSNLSGGEKTVAALALLFAIHSYQPAPFFVLDEIDAALDNTNIGKVARFIREKTQTSFQCIVISLKEEFYGHADCLVGICPDPGECTISRIYTIDLSVSEAMLVYLGEAIREGNLVEMQNLYANSLATLMHNYVHTIYKE--LYYRQIYADDRFNSYQNYVNLFNYILSSGPVSLELPNQWLWEIMEDFVYQF--QSFSTFMWNVHSVLNVLHSLVQKSNINQQLEVFSKGGNPDEVAGEYGSRTLYKMLGYFSLIALLRLHSQLGDYFQAIKVLQHLELNRQGLSRVPACQVSTYYYVGFAYMMMRRYEDAIRTFCNVLMYISRTKRSYQQRQMISQTDKMYSLLSICYVLHPQRMDESVMAQLKAKHMLKMTQGNMEVFERCPRFLSPVQWNAFRGEIEAQRTIRSYLKLYTTMPVEKLTKFFLMCFKHLMSNVVLEGEFQTGSDMDFFIDKDMIHIADTKIAPKYGEFFAKQFNKFEEMYEMSIVFSRCNLV-----------------------DPTLAKRRVFMFDDFLRRKAKTVCAYRDDNGKPFVLPSVRMAEQELMTKNLDKEYLPIGGLGEFCNNAAKLALGDDSPVVKDGRNATVQGISGTGSLRIGAMFLDEFLKGNKSVYMPNPTWGNHIPLFKKCNFQVKQYRYYDPKTCGLDFDGALEDISNIPEGSVILLHACAHNPTGVDPRPEQWVEIEKVIRSRNLFPFLDMAYQGFATGDIDRDAMAVRLFAKSG-PMCLSQSFAKNMGLYGERVGAFSLICESKEEMSRCMSQLKILIRPLYSNPPVNGARIANLILSDAKLRAQWLADVKLMADRIISMRTRLRDGLKREGSTRDWKHITDQIGMFCFTGMTPDQVAKLTKDFSVYLTKDGRISVAGISSNNVDYLAHAIHNATKRIIPKLRHVAHCVLMATLIYLFVKWSRPPDSVDPFAEYGVVFTIILYLFRLLPLLALPQSLTNLLGLTLYNAFPPKVRLKVAAQEAPFLCIRVVTRGDFPALVRHNVDRNLATCLDLGIENFAVEVVTDKAVYLASHPKIRQTIVPPDYRTRTNAMFKARALQYCLEDDVNVLADNDYILHLDEETLLTRDAVRGVLNFISSGQHPFGQGLITYANERIVNWVTTMADTYRVADDLGKLRFQFNCFHKPLFSWKGSYVCTRAGAERAVSFDHGPDGSVAEDCYFSMVAYARGYSFEFIEGALWEKSPFTISDLIQQRKRWLQGIYLVVHSSKIPWRFKVWLACSLYAWATMPLSTSNLFLAPNFPLPCPQTFNVTCAFIGALNLYMYIFGVLKSFSIKRLGFFGFFMCLALVVVAIPVNIFVENVAVVWGLLGKKHKFYIVDKNIVMETQTEGCSEPAKLQCPTCLKLG------------KQAWGXSKSVHKTASASPYNPWPNYRYTGKLRAGRVTPKRPVPEHIQRPDYADHPEGVPISEQKMKGA-EIKVLSKAEQDGVRRASLLARECLDVALAAAKPGVTTDELDRLVHEAAIERDCYPSPLNYYMFPKSCCTSVNEVICHGIPDDRPLEDGDILNVDVTVYHNGYHGDLNETVFIGKVDDNAKKLVRVTYEALQKAIDACKPGMLYRDIGNIIQRHVQQNGFSVVRSYCGHGIHSLFHTAPSVPHYAKNKAVGVMKPGHCFTIEPMISEGNWHDTVWPDNWTAVTTDGKRSAQFEQTLLVNECALSI---HKQANIEIRFFNFSTLRESLKERSARRRELLAQQLGAGCADNLGLLLGNDKTPTVGQGTQPDDDEVMAYRDSSTFLKGTQSANPHNDYCQHFVDTGQRPQNFIRDVGIQDRFEEYPKLKELIRLKDDLIRETATPPMYLKCDLLQYNLRELNGKFDVILIEPPLEEYQRSCGVTNTRFWSWEEIMKLEIEEVAAPRSFVFLWCGSSDGLDLGRQCLRKWGFRRCEDICWIKTNISNSKAKNVEPRAVFQRTKEHCLMGIKGTVRRSTDGDFIHANVDIDLIISEEPPFGMMEKPEEIFHIIEHFCLGRRRLHLFGRDLTIRPGWLTLGPELTNSNLNTDAYNAHFNTANDYLTGCTERIEALRPKSPPPKSALGGSSRGSRGRASLKKLGLSSLDVSGKRVVMRVDFNVPLKDGKITNNQRIVAALPSIKHCLDKGAKSVVLMSHLGRPDGTANPKFTLAPVAGELKKLLEKEVTFLKDCTGSEVEAACADPAPGTVILLENLRFHVEEEGKGVDAAGNKIKADPAKVKEFRASLTKLGDVYVNDAFGTAHRAHSSMVGVELPQRAAGFLMMKELEYFSKALDEPARPFLAILGGAKVKDKIQLIENLLDKVNEMIIGGGMAYTFLKVSSGMKIGGSLFDEDGAAIVEKLLKKAADKKVQIHLPVDFITADKFAEDAATGSADVDGGIPDGWMGLDCGPKSVALFVEAVGRAKTIVWNGPVGVFEFDKFAAGTKAVMDAVVSATGRGVVTIIGGGDTATCAAKWGTEDKVSHVSTGGGASLELLEGKVLPGVAALTDAMRTFGDRPHSFQLEDGGEYYYIGTEVGNYLRLFRGTLYKKYPSLWRRAVTVEERKKISQMNMSQHSAANFISLLKKSEVDDLIDGNEEKYRAAPVQENDGGAHGAKNARPSFMPAAPNNAHHLDAVPCSTPINRNRLQHKKNRSFPMLYDDLDPAMLHESAALPECLVPIRLDMEIEGSKLRDTFTWNRHEAHISPEQFAELLCDDLDLPPLLFVPQIAASMRQQIEAFPSESLLDEQTDQRVLIKLNIHVGNISLVDQFEWDMSERANSPEEFAAKLCSDLGLGGEFVTAIAYSIRGQLAWHQRTYAFSEAPLAQLEMPFRPQSEAEQWCPFLETLTDQEMEKKIRDQDRNTRRMRRLAHTAWMNR-----ERIDALPSSERYLGLVNFGNTCYCNSVLQALYYCKPFREKVLEYKAKNKRTRETLLTCLADLFHNIHSHKKKTGTLAPKKFIARLRKDNEVFDNYLQQDAHEFLNYLLNTIGDLLQAENSWVHDIFQGTLVNETRCLTCETVSSKDEDFLDLSVDISPNTSISHCLRCFSSTETLRGEHKYHCEQCNSKQEAQKSLKVKKLPPILALHLKRFKYTEQQNRNTKLSWRVVFPLELRLFNTSDDALNGDRLYDLVAIVVHCGTGPNRGHYISIVKSHGLWLLFDDDLVDKIDPATIDDFFGLTQDTPKASESGYILFYQSKE??????????????????????????????????????????????????????????????????????????????????????????????????????????????????????????????????????????????????????????????????????????????????????????????????????????????????????????????????????????????????????????????????????????????????????????????????????????????VDGSGPEETQDAEAATIDELKEHTAHIQKAVAQKESRFILRILRLLPATRKKLNSKLLRKTINGFYTHDKAHRELLLSFVDAEDTDADAVQKSAHLALLPEVDVYLHLLLLVHMVDAANMERAVRCAELLKSKIETHSRRSMDLLAAKTFFYYSRVYELDGKLCYCISFLLKRLRTATLRSDFEGQAVLINCLMRNYLHYSLFKQAAKLVSKVSFPEMASNNEWARYLYYLGCIKAIQLYYTDAHKNLLQAIRKAPQHSALGFKQTVYKLAVTVELLLGDIPDRTIFRQPALRKSLAPYFQLTQAVRTGNLGLFNQVLESYGARFQADHTYTLIIRLRHNVIKTGVRMINLSYQRISLADVAAKLQLGSAEDAEFIVAKAIRDGVIEATIDHDKGYVQSAENIDVYCTGEPQAQFDQRISFCLDIHNQSIKAMRFPPKSYNKDLESAEERREREQQDMEYAKEEDDDSYQVAVRLTVALAAFAALTMWKSAVTDMTENFRESHPLLFEFLANLPDQVVRSLTLSSVVLSLCHSALLSPLRQAALRAVPLNFINALL-DISDEPACEAVLSLAQHQDGNQPKLMMTWLVQITKCVPVVQSKQAARDYDSIYSMLTGVADTHASLILKG-LRDTETQMEMLVKMLLDCVGTPGVYPAEETLSRVALPFWHTLLDEHNRLLP-AETAKNLQPVYEQLVRLLNKSQLPDPDCLDSDEREELRCYRQDITD--CYMCIMTVLPVQLIRFFMTELETANNSRIIEACLFAINSVGDVIDGDEDVGIVSAILEILPRVPASDEIQSQVMTAVGTFADENIGPLVHILLGGLQRTSAAASMALKDVARAHGDRLEPAANDILQAIRVLKHRDRQRLVSIVGHVASALSSDQALTSLTALMAPFVLQMNEMVNLAEQLLPLFKQIATKYANSDCEVVSSLAECIRKMVPILEIDVILLELLTICGGLLINTTTSCS----TELVEAFYRMGTLLKKLSPDDLIQLCPEHHTFKSLTQFVIATERSEIKEALEGIVIQLIQNMCVSRQYITCQADIFLALSRSAAALHRFARTADSRQNFVRQILRERSNKRILINVV--SDFTVQVKVKWKDSY-DVHLHLPDVFRAQLFALTGVLPERQKIMCKGSILKDS-WGATILMMGTKEELPQPVEKTLFVEDMDDSEISAALKLPTGLTNLGNTCYMNAVVQCFKTVPELTELDKFVVSITAALGSLYRSMDKATRAPLVLLQALQTAFPRFAEKGEHGQQDANECWTEMMRMLQSLVDQIFGGKLVSLKCTESEEESTEDFLQLSCFID-KDVKYLMAGLKLRM-RETITKMSPVLKRDAAYKTSKISRLPAYLTVNLVRFFYKERESINAKILKDVKFPMMLDVYELCSNDLQEKLLPQREKFKQWDDEFSFEDDGSNNSGYYQLQAVLTHKGRSSSSGHYVGWIRREWFKCDDQVVVPEEEILKLSGGGDWHVAYVLLYGPRLLVGLINKRLPKELLLKIFSFLDVVSLCRCAQVSKEWNVLAMDGSNWQSIDLFSFQRDVTSEVVAYIARRCGGFLRRISLRGCQGVPDDSLYVFAQHCRNIEQVLSNCHKLTDDSVLALALSLHIDSCVELTDLSLGSF----KHLRVVNISWCRKITGQGIGMIAG--EHLLRFTAKGCLDNEAIVKLATKLQVLNLQCCSFLADSAVIAVAQNCPDLRHLCVSGCTQLTDAAPQALATGCLHTLEMANCTRCGDAGLIPLVKACHDLRRLDLEECALVTDSSLNHVAAFCPLMEQLTLSHCDQITDQGVHKLALQCIEIDNCPFISDTSLEYLADHLRRVELYDCQLITQDAIGKFQPDVRLHTYFAPATPRQRYCRCCVIVSSKGARVFCPDHPNANLIEDYRAGDMICPQCGLVVGDRIVDVGTEWRVFQNEKSSNDPTRVGAAENPLLSGSDLSTIIGRTGDASDDQGNAKYANRKTMSASDRALIGAFREISAMGDRINLPKTIMDRSNLLFKQVHDGRSLKGRANDAIASACLYIACRQEGVPRTFKEICAVSKVSKKEIGRCFKLILKALETSVELITTGDFMSRFCSNLALPSAVQKAATHIARKAVEMDIVAGRSPISVAAAAIYMASQASPDKKSQKEIGDIAGVAEVTIRQSYKQMYPKAAQLFPEDFKP????????????????????????????????????????????????????????????????????????????????????????????????????????????????????????????????????????????????????????????????????????????????????????????????????????????????????????????????????????????????????????????????????????????????????????????????????????????????????????????????YVKKEHSLVKPYQGSGMTMPNWDFTGSTMISSNYVRLTSDTQSQQGSIWNKVPWEIHLQFKVHGHGKDLFGDGFAIWYTKDPLQPGPVFGSRDFQGLAVFLDTYANQNGHHNHAHPYISAMVNNGTLSYDHDRDGTHTELAGCEAKFRNSDYDTLISVRYEHDTLVVSTDIMGQKEWKECFRVSGVRLPTKYHFGVSAATGDLSDNHDIISIKVFELDELREFIAPQAAPHRDHVDDSMSGTKFFFLMLFLMLVLVCFFYQRRQEDARKRFYRDRDAFDEIRDKNCRIQDINELFLPNDTVTHVPNIKRLNIDPVFPNRTNLLHIHNMAISKAFFFSFILQRAKDDQPGFMYYFMSVISDVAANRFINASAIYYAPNMSFTPSYKGFFNKTMPLFAPRAFRSDDFNDPYHLEGTSTLNTIEAIDLGAISQNYSSDQYRINEWYSSWLPDLTKRQDSKTTYTVQITGNNDTFVWHGPPAANDNPGPVKWVRPYFDCGRSDKWVYGATSPIPDIYPRHTQWRHIEIPRYVAVAAMELDFERIDVNQCPFGPGN-PRPNYFAGTSRCKNDTTDCEPVHGYGFRRGGYQCRCKPGFRRPRMVRNPYHGELIERASKQEYEKGYQCDKIGYIGVLTQNLNNYMAIGTRMDPALGGNVLYGKEVQLENEARMAVRLANFVSGFLQIVDPKDLFAEFRVPDKPLTADQMIGEVMSIVIGDQKVVGAGVYFDYKAFFGPY--AWRQGRNERKYFVDDTTKIQIRYNSSGIKYDHYPLQYKAADVGYWTSPYFDCGGYHNSWMVTYAVPFFGWDSLRARLQFRGVVAVIELEQLEINQCNAFENTHKCDRKSSRCVPILGRGFQGGYKCECNQGYEYPYNDPITYFDGQIVEAPSRFERMTHVSGTDKSKYGGRYMVTALPGDGIGPELIGYVKEVFRYGGVPVDFEEVHLDSSRDDIDLMEQAITAVKRNGVALKGNIETRHNDPNCKSRNVELRLRLGLFANIVHVTSQPGIETRHKDVDIVLIRQNTEGEYSSEEHTSIKGVVESLKVITKARSDEIARYAFEWAKNNGRKKISCVHKANIMKLSDGLFLQCCTETAKEYPEIEFDNIIIDNCSMQLVANPHQFDVLLLPNLYGNILTNLVCGITGGPGIASGRNYGREYAVFETGTRNTGKSIAGKNIANPIAMMNAGVDLLYHLGLTEHAEIISTAIDKTLNVDRVHTPDLGGQATTTEVVQNIVKEVQKHSMTYSASSNEVLLRDPPTDGISAVKFGQTSNQFLVASSWDGFVRLYDIHGDRVRAKFEHGSPVLDTCFQGSSHVWSAGSDGGVRLFDVNQGTELKAGSHDDTVRCIEYASDVSLVVSGGWDGAVKLWDARRPVGASSHTQDNRVYAVAIAGERIIVGTANRKVLIWDLRNMAFVLQKRDSSLKFQTRAIKAFPDKTGYVLSSIEGRVAVEYLDPSPEAQKKKYAFKCHRMKDVTKVEHIYPVNAIAFHMIHGTFATGGSDGFVNVWDGRNKKRLCQFHKFPTSIASLAFSPDGSALAIASSFMHEYRLEPNQPPDQIFIRHVAIAGQR-----------------------MKEYKPTDATTNPSLILQAAKLPQYADLIDKAVSYGRSPLEQLEEARDKLFVLFGSEILKIVPGRVSTEIDARLSFDKDASIAKALKLIALYKELGVPKERILVKLASTWEGIEAARVLEQEHGIHCNMTLLFNFTQAVACAEAGATLISPFVGRILDWYVANTDKKSFEPQDEPGVKSVTSIYNYYKKFGYKTVVMGASFRNVGEIRALAGCDLLTISPSLLKELANSDEPVVQHLQAEKAASLPLEKIEVDEKRFRWDMNEDQMATEKLSDGIRKFSADARKLEVLIQEKLKMGDSVCRPLATVYTDTNEASGTNAGMPAVFRSPIRPDLISFVHHQLLKNKRTPYAVSKEAGHQTSAESWGTGRAVARIPRVRGGGTHRSGQGAFGNMCRGGRMFAPTKTWRRWHRRVNVTQRRHAAASAISASGVTALVMAKGHAIEQVSEVPFVVSDKVQDYQKTKQAVALLKSVNAWPDIEKVYKSKRLRPGKGKRRNRRYKKKCGPLVIYEKDNGIVRAFRNIPGVDTCDVNALSLFKLAPGGHAGRFIIWTESAFRKLTEIFGTFTKNSKVKKGYKLPRPMMTITDLGRLLKSEEIRSSLRPRKSIILKKTNKPNPLKKVHLMDRLNPYAIVEKRHRILTLEKEAGKNLKAVRAKERMKAAKQKRLANPFPKNRKLVQGAVLKKTLDAIKDLINEGTWDCSASGISLQAMDNSHVSLVALNLRADGFDKFRCDRNISMGMNLASMAKILKCAENNDVITIKAQDDADTVTFVFEAQNQEKVSDFEMKLMNLDSEHLGIPDTDYSVVVKMPSSEFQRICRDLSQIGDSVQLTCTKDGIRFSASGDLGTGNIQLSQTADVEKEEEAVIIEMQEAVTLTFALKYLNSFTKATPLSPQVSLSMSADVPLVVDYLRLFLRILFVPKEF---MFARALRQLVAGQAAV-SRSFSTSQRSNVKVCVLGASGGIGQPLSLLLKQHPGISYLSLYDIAHTPGVAADLSHINTGAKVKGFVGAEQLKAALEGVEVVVIPAGVPRKPGMSRDDLFTTNASVVRDLATACAEVCPKAMFAIISNPVNSTVPIASEVFKKHGVYDPTRIFGVTTLDVVRANTFIAEAKGLDPVSMSVPVVGGHAGITIIPLVSQASPKVDFPQDQLEQLTKRIQDAGTEVVQAKAGAGSATLSMAFAGARFVFSLVSAINGKNVVECAFVKSDIGDAGFFSTPLVLGKNGMEKNLGLGKLSPFEEKMVADAMPELKKSVQKGVSH---EYEWLLKFEVNDIVEQLVIAECSKRFPL-ARSDKFVM-QSKVVATLMGDSISHADITLRL-PKHSQRTIVQNDAQWKLQQIQDAGNHLMQAMNLLRFKFSSGQEVRNLMAEVMGCLGRGRACLVVPKKRTIEEIMQSRNMRSLQPPLPNDVAVSFYIQSYKLVFAVYHV-QKDSQKF-DAECSVPWLSEALVLFTVALQLCQQLKDKVEVFQYNDFLPMFIPIFLEHDAPLPEISELIRTCDVCFKDAESIL--NGFVSILVCEFCRRLESASTPKVAFN--VLYNFFEGLRVDVYCSLIKVAGRSVTEVFADVGPIKTWLHVSKVRDVYRNLHRELSDMALRVMVELLSTYSEHDAAEAAEDAERCIASTLADPNAFLMDHLLPLKPIKALEGKPIHDLLKIFIFDKLSAYQDFYKKNKAFV-EGLGLDHESNVEKMRLLTFMLMAEKQREISFDDIARELEV--TDVEEFTIRALKTKLVSAKLNQMTQKVVVISTMHRTFGMSEWTQLRDILRLEKVEQSTQQ-------------NVEELAEEAKAIDEGEERAVIEESPQFKELVNILIEWINDELAKHRII--VKNIEEDLYDGQILHKLLEQLTNSRIDVVEMTQNEEGQREKLKVVLERASQALGLKWSVDAIHSKNVVAIVHLLVALARHFRAPVKARLPENVTVSVVSVTKR-EQLTRTYDEYGMKVE-RDAFDQLFDHAPDKLVVVKKSLLTFVNKHLNKIKFEDLD-KQFHDGIYLALLMGLLEGYFVPLYLMLTP----------------------KNKVNNVAFAFGLMKDAGLKPKARPEDIVNYDMKSTLRVLYNIFMK???????????????????????????????????????????????????????????????????????????????????????????????????????????????????????????????????????????????????????????????????????????????????????????????????????????????????????????????????????????????????????????????????????????????????????????????????????????????ILYRNRAEWADVTPILRISYSEKFQDCFGYLRAVISGELSERVFELTTTCAGENPACYTVWVLRRTLLQHLNKDYRDEMRFMSRMILDNQKNYQVWYHRQRLVKWLELAFLDEILDSKNYHAWQYRQWLLTTFNLWDDELDFTTALLIDDLRNNSAWNQRYFVISKTTGFVVEREIRFTQEKILVNESAWNYLEGLLSVFLFSTLLQKATEVDGLRTQYWAFRENLL?????????????????????????????????????????????????????????????????????????????????????????????????????????????????????????????????????????????????????????????????????????????????????????????????????????????????????????????????????????????????????????????VSPGTTITTDGYMRGHGGLLSAVAGVVEKVNKLITVRPLKTRYNPEVGDVVVGRIVQVC--QKLWKVDVGGRLFAALHLSSVNLPGGELRRKSIEDERMMSQYLIDGDLVSAEVQNVGVDGGVSLHTRNLKYGKLGQGTLVKVSPSLIKRCKHFHNLP-NGVHLIIGLNGFIWVTSSKFTREAICRTRNVILCLAKYNLMLFDTPIVYAYEIS-LVQLTRHRSDKLVGGWLLACGGMAFGAVILGGVTRLTKSGLSMVDWHPLNEGRPRTAEWEAEFAKYQQYPEYKARNKDMTLEQFKSIYWMEYIHRMWGRSIGAVFYVPATIYWFLGYFSPAVKKRVVVLGGLLAAQGLMGWYMVRSGLQEKPRVSNLRLAAHLGTAFVFYSLLFRSALQHLLPDAAVVRFVRAKALVFITALSGALVAGIEAGLVYNSFPKMADRWIPSDILALPTWKNFFENPTTVQFDHRLFGETVITALYIHSRKVPLPRRARLATHAMLAAWIQVGLGISTLLTFVPTPLAAAHQAGALTLLTTLLWLTHELKLVKK-IAKWGEGDPRWIVEERPDATNVNNWHWTEKNACQWSRDKLMALFTDLEITD-TVMCVIKQVKKCEGEATANNRKAKLIFFYEWELELCAGS-LVKGRVEIPNLSDENNIDEVAVNIVLVDEKLKAMMRSKGTEQIRAKLEEYVSSLKSDFSQGLILPTKDSGANSTSSKGKEFSTSELSMEEFKCTADELYRALTMVQAFTQGPVGGSFQILDTNVNGKFTKLNELEFEWRFKSWPAEHYSKVITIEQANDCSLLQQGVPTDEVDRTRGGWQRYYFDAIKRTFGFGAILMDPETFLEIANQVSKLKMYPYFEIAHCVVTLLYLREDLGTGSYA-SR--------VSMFSIFAGAMFAALLLGEPVLAAFKSNQSLVLATGVWYLIFYSPFDIVYKLSKVLPIKLVLTLAKEVTRAKKVHDGVHHAAKLYPNGYLIMVIIGVVKGNGSSFLKVFERLLRGLWTPLAMEIMQPSFATKACVVASLVFVIDKKTDLISAPHSLVYFGVVCFFLYFKLSSVVLGLHDPFTPFENLVCAVFFGGIWDAISRAL-----NKGGDASGGGPKENKDAASKKKEMFRPDGIVQRRTAAVIARDRDDLDRKDSDGGDNDKETRMTLMEEVLLLGLKEKEGYTSFWNDCISSGLRGCILVELGIRGRIDLEAAGMRRKSLLMRKVVVKNDAPVGDVILDEALKHIKETTTPETLQNWVDYLSGETWNPLKLRYQLRNVRERLAKGLVEKGILTTEKQNFLLFDMTTHPLEDQNVKDKLIKRVQDSVLSKWVNDVHRMERRQLALLLLSHASDVLENAFNPLSDEEYELAMRRVRDLLDMDFEAECAKSQSCDIMWGVFAAFVKMADGVPTFKCVLVGDGGTGKTTFVKRHVTGEFEKKYVATLGVEVHPILFHTNRGAIRFNVWDTAGQEKFGGLRDGYYIQAHCAIMMFDVTSRITYKNVPNWHRDLVRVCENIPIVLVGNKVDVKDRKVKAKAIVFHRKKNLQYYDISAKSNYNFEKPFLWLARKLIGDANLEFVAMPALAPPEVTMDPEWQSKLENDMKEAQNTSLPDDEDDDLLAAQMTPVLKESKFRESGMLTPEEFVAAGDHLVATCPTWAWARG-DKTYLPEDKQFLVTKNVPCHKRCRDMEERVIEED-EGWVDTHDNEDDDEAGVDMDAFLDEDAEAVDILATRTYDLNITYDNYYRTPRLWLTGYDENCRPLTTEELYEDISQDFAKKTVTVENHPHIEGLPQASVHPCRHAQAMKNLIQTVEDGGGLEVHMYLIVFLKFVQAVIPTIEYDYTANFNMMSNLEKKLPYDKLCANVDVVKKRLNRPLTLSEKILYSHLDQPQSEEIVRGQSYLRLRPDRVAMQDATAQMAMLQFISSGLPKVAVPSTIHCDHLIEAQLGGVKDLARAVDINKEVYSFLASAGAKYGVGFWKPGSGIIHQIILENYAFPGLLMIGTDSHTPNGGGLGGLCIGVGGADAVDVMAGIPWELKCPNVIGVHLTGKMSGWTSSKDVITKLAGILTVKGGTGAIVEYFGPGVQSISCTGMGTICNMGAEIGATTSVFPFNSRMADYLASTNRRDIADAAESVKDLLTSDSGCKYDQVIEINLDTLEPHVNGPFTPDAAHPISQLGKVAKEKGWPMDVKVGLIGSCTNSSYEDMSRSAMLAQQALDHGLKSKSLFTVTPGSEQIRATIERDGQAKVLKEFGGMVLANACGPCIGQWDRQDTKKGEKNTIVTSYNRNFTSRNDANPQTHAFVTSPEMVTALSIAGRLDFNPLTDELTGSNGQKFKLKPPVGDELPRAGFDPGENTYQGPPPDGSSLTVDVDPKSQRLQLLSPFDKWSGSDLKDMVVLLKAKGKCTTDHISAAGPWLKFRGHLDNISNNMFIGAIPEESGEANKVQNRLTGAWGAVPDTARHYKAQNVPWVVIGDENYGEGSSREHAALEPRHLGGRAIIVKSFARIHETNLKKQGLLPLTFADPADYDKIKSDDRISIVGLDSFAPGMESRAGMFKNTFQSGFLSILYSLGSKPLQIWDKKVRNGHIKRITDNDIQSLVLEIAGSNVSTAFITCPADPRETLGIKLPYIILIVKNLKKYFTFEVQILDDKNIKRRFRASNFQSTTRVKPFICTMPMRLDEGWNQIQFNLADFTRRAYGTNYVETLRVQIHANCRLRRVYFADRLYAEDELPAEFKLYLPVQHRTAAQVLRER---------??????????????????????????????????????????????????????????????????????????????????????????????????????????????????????????????????????????????????????????????????????????????????????????????????????????????????????????????????????????????????????????????????????????????????????????????????MGRKFYVGGNWKMNGVKASIQKICEQLKNAQPCTEVCVGVPAPYLQLVRDELPANFHVAAQNCYKAASGAFTGELSVDMIKDCGCDTVILGHSERRNVFGEKDQLIAEKCAFALNNGLTVIACIGELLEEREANKTEEVVYRQIKAYADLIKEWKNVVIAYEPVWAIGTGKTATPDQAQEVHAKLRQWLTNNVSEEVGLNTRILYGGSVTAANCKELAQKADVDGFLVGGASLKPEFVQIINAKQMSTAVSKKRKFVADGVFNAELNEFLRRELAENGYSGVEVRTGTMKTDIIIMATRTQDVLGERGRKIRELTAVVQKRFGFKEGSVNLFAEKVSSRGLCAITQCESLRYKLIGGLAVRRACYSVLRCIMEAEAMGCEVVVSGKLRGQRAKSMKFVEGLMIHSGDPTNHYVETAVRHVLLKQGVLGIKVKIMHPYDPLGKRGPALPLPDKVTVTASKHEDDNVEIRSE-KDNTG???????????????????????????????????????????????????????????????????????????????????????????????????????????????????????????????????????????????????????????????????????????????????????????????????????????????????????????????????????????????????-MVDLVLDRDIRIWVFLPIVVITFLVGIVRHYVSILLTSTRKAEIQQVYDSQALIRCRYLRENGKYLPAKGFMMRKHFFNDEDTGWLK-TQKRASPMSNPMHDPGMMTDMLKGNLTNVLPMIMIGGWINWTFSGFLTTKVPFPLTLRFKPMLQRGIELVSLDASWVSSASWYFLNVFGLRSIYALVLGEENAADSTRAMQDQMTGPAMQMPQDPKAAFKAEWEALEVVEHKWVLAGVEEDLCSKPLQF---------MVIYKGAASEAGRAMQILKRRERQKEDVELKRQRIEHEMRVS-MGDKFSSHFDAVEAQIKSATVGLVTLDEMKAKQENAVKEREKRLAQKEQEEKQRAEDKKWAQKEKQKKAIQALSFNLDEDEDEVEGEDDDGLDVSR-------SESSDA-HSNGGSGNNSSDNDDDDGNSRESG--IRVKKNPDVDTSFLPDREREEQERLIREELRQEWKDKQKTLKEESIQITFSYWDGSGHRRVVDMKKGNSIYQFLQRCLDSLRKDFYELRVVSADQLMYIKEDLIIPHHYTFYDFIVTKARGKSGPLFAFDANEDIRMTSDASKEKEESHAGKVLLRSWYERNKHIFPASRWEPYDPTKCYDKYTIKDKKKNMPPAAKKPAKPTVPETLLKERKFNAELRQQRLLAAAGKKKAARARRVLAFRRAEQYVSEYRRMAESEKSNRLVAKVNGNFFVPDEPKVAIVVRIRGITGVSPKPKKVMQLFRLRQINNAMFVRLNKATINMLRIAEPYLAWGYPNLKTVRDLIYKRGFGRVNGRRVPLTDNVIIEEKLGKYGIICMEDLVHEIYTVGPNFKQAVNFLWHFKLNNPKGGWRKKTTHFVEGGDYGNRETLINSLLRKMVVKISADDLWRGVTSVSNAGRKRGRASGSSRKFAKNLNKGQTIGFGKANMVWPGLNVPVIRGREVVQQQALPPDPERQQRLLAIRDKQHIFQRAKRGPLERGWSGAKAPGRSLGPPDPIADQKFDDFKSVIIQLRTISIMRGYRGRIRRHKAVVIVGNGAGLCGLGIAKAPEVRVALRKAKNRAIRRQCYFQRYEDTVLHDFISEVAFTRISVKKKQRGFGLVCHRAIREVCKCVGIKDLYAKVDSRCTRPVPTIRAFLLGLHRQRTHQEIADEKRLHVVEYRPENNYFPRIVASPQHVRTEEEVDPYEYLDMDLITSKGRLLVQKPKYEPFYRRLPCWQIHLKKTDNLKNDRIIRQRLILKYGSLKSFLTVREAKALAGG????????????????????????????????????????????????????????????????????????????????????????????????????????????????????????????????????????????????????????????????????????????????????????????????????????????????????????????????????????????????MAVGKNKGLSKGGKKGLKKKIVDPFSRKDWYDVKAPSMFNVRNVGKTLVNRTVGTKIASEGLKGRVYEVSQADLHSGEDAFRKFKLVCEEVQGRHCLTNFHGMDLTTDKLRSMVKKWQTLIEAQVDVRTTDGFVLRLFCIGFTKKAQNQVKKTCYAQHAQVRAIRRRMVETMQREVASCDLKEVVNKLIPESIGKDIEKSCNYIYPLHDVHIRKVKVLKKPKFDMGKLLEMHDEGKGPSGGDGMAVDRPDNFEPPVLDEVVKIGIIGGSGLE-RPNLLKEVTPYGKPSDA-LITGKIDGVDVVILSRHGRGHTINPSNVNYRANLFALK-QEGCTHIIATTACGSLKEEVHPGALTPRSFIDRTREQTFYVCHIPMVKPFLMELISKHPSGVIVCIEGPRFSSRAESVLFRQWGADLVNMTLVPEVVLAQELGVPYAALAIVTDYDCWR-D--DAVDVSKVATLKDAADGVCRLLRAVLPKINKIAIF-------------------------GMRVLRHEEFETGCKAACNGRYDGFWSKTMIGYGPEDTHFVMELTFNYGVDTYRKGNEFRGIVIEDCGIIKRCEELKYATEKDRNRTVLNGPGGHKFYIVDKTS-K-EDPVKQVIYSCTDVAKTSKFWVNTLGCRLLNTGDDFLEVAYDEAKTSLRFEKISEAMDRGEAYGRIAFACPKSHLPELESQVKNGGGSVITPLVSLSTPGKASVEVVILGDPDGHEICFVGDEAFRELSKEDPTSLKVLQEHMA--EYEAFLKKTSARPHQD??????????????????????????????????????????????????????????????????????????????????????????????????????????????????????????????????????????????????????????????????????????????????????????????????????????????????????????????????????????????????????????????????????????????????????---------MKLLSSSPFHTGRRLAMQMVPIVIEQTGRGERAYDIYSRLLKERIICLMGPINDDVASVVVAQLLFLQAEAPKQPIHMYINSPGGNVTAGLGIYDTMQYVTPPIATWCVGQACSAASLLLAAGEAGMRHSLPNSRIMVHQPSGGISGQATDIQIHAEEILFLKKRVNLIYAKHTKQPIETIDAMMERDRFMSPEQAQELGLIDRVMERPP-VREGSP-MRLDRPVGTWLTLLPLWALAAAAPAGSLPDLGIFCTGALLMRSFGCTINDMWDRDIDRQVQRTRQRPLAAGDISRWDALWFAGGQGLACLVLLQLNWHTVMLGLASVGFVVIYPLMKRFTYWPQAMLALVFNWGALLGFSATAIPMYAAAFSWTLIYDTIYAHQDKKDDILVGMKSTALRFGERTPLWLGAFSTMATALMAWPYFAAVTLKIDNIGLLVGCIGGTLFKIARVDKPKTRKGSRILKSKEPLLIENAKTSFVKGANINQPTVQILKDIYTLKKTESVFYQKKHPFEDATPLERLAKKDTSLFAFGSHNKKRPQNIVLGRCFDATILDQFEFGVQNYKALHEFKVAKIGIMVKPVLVFAGEAWQEMKRLKNFLTDFFRGDYIGLSGIEHVISFTASDVLLRSYRIQLKKSGLKTPR-VEVEEIGPRMDLKVRRCKIASDDLFKQALRRPKELKAKKKKNLEQDDLGTALGRVHMERQDFRLQTRKMKGLKVEILPALQDNYMYLVITREAAVVDPVNPQKVFEAVKVKLLTVLTTHHHSDHAGGNDQIVVYGGDDRIKQLTHKDDFTIRIGQMVRTILTPCHTSGHVCYYIVFTGDTLFTAGCGKFFEGTAKQMLAAMLGALPFETRVYNGHEYTVNNLKFALHVEPNNDAKRKLSWAKDEPTIPSTIAEEKSFNPFMRTAVQRHTDPIAAMDSLRREKDTFRMNRLFGKAKAKEPPPNLSDCIQTVDQRANNMDEKIKKLEIELVKYKEQMAKMREGPAKNLVKQKAMRILKQKKMYESQRDNLMQQSFNMEQTNFATQQLKDTKVTVEAMKIGVKEMKQEYKKVNLNEIEDIQDDLEDMLDQANEVQETLGRSYGMPEIDDEELESELAMLNDEIALEDTSYLDDVTAPTVPSKDPSVKEKAEGGIMVDEFGLPKIPS----MQAARAKATLPDLPYDYNALEPVISAEILKVHHDKHHLAYVNNFNMLKEKFEEAVQKGDLTAQFSLAGAYRFNYGGHLNHSIYWQVLCSAKSGEPSADLLAAINRDFGSLDVMKEKVSAAAIGVQGSGWSWLCYNKATKKLQCVTSANQDPLEATTGLVPIFCIDVWEHAYYLQYKNCSAGLCKG--HLAG-GQLEGDLRPMQ?????????????????????????????????????????????????????????????????????????????????????????????????????????????????????????????????????????????????????????????????????????????????????????????????????????????????????????????????????????????????????????????????????????????????????????????????????????????????????????????????????????????????????????????????????????????????????????????????????????????????????????????????????????????????????????????????????????????????????????????????????????????????????????????????????????????????????????????????????????????????????????????????????????????????????????????????????????????????????????????????????????????????????---RVLLVTVFLALLVDDVRAYFIVVDAHSEECFHDRVAKGTKMGLTFEVAEGGFLDIDVKITGPDDKVVYNGERESSNKYTFAAYMDGQYKYCFSNSMSTLTPKTVMFSMDIGEEPSDDAKAGADAHENKLEDMIRELHTAMTGVKHEQEYMMIRDRIHRSISESTNSRVVIWAFFENLVIIAMTLGQVYYLKRIFEVRRVVMRLTQILKLPKDYANLPESYVNRVSTKIEWKTPKGRQYRKAVLKKPYGMDRPWTQEWQHNKVGTYPQPKILFRGDLVEILVGDDKGKQGTINYIVPERNWVTVEGLNTVTYPSLQRTLVLLVDPADERPTRIEWRYTEEGKRVRVSLRTGRIIPIPAAEATIDYKYRDKPKDTSAAELAQITFIP-KLATFEMDIMEQHGIEDDRVPARTFWYMSDNSA--FFVDMVEQKLEFILLLSRLASVVFAVLCMLGNPYSFYQKAIICNGFTSALRLHHRMPTVRLNAEFLGAVLLEDACHYLIYSLIF-LPIS-MVLLPPTLFATLHSLQLLDRAGTLHQ---NRVKVFRLIAMTEIFLMPTVVIGLFWGVSLMAPFMYYRFLKLRYASVRNPYTRNVFYELRLMAQQLAAPSCPAVVSRIQFISSRADLAIVSIAVYLSFPEIRPEERPYVKLPTSLEDAKNLGNVLSNYTDDQVLLAFFCTYIFLQSFAIPGSIFLSFLSGFLFPFLLAIFLVCLCSALGASLCYLISYSVGTRLVMHCMPNILYYLIFLRITPLLPNWFINIASPIVGVHLLTFFIGTFIGVAPPSILAIRAGISLQQLATAFTWENMLLLLGFAVLSLVPVLFKFKKKFEEVQEKLTRIAIITEDKCKPKRCRQECKKSCPVVRMGKLCIEVTPNDKIAFISENLCIGCGICVKKCPFEAITIINLPSNLERETTHRYGPNSFKLHRLPTPRPGEVLGLVGTNGIGKSTALKILAGKLKPNLGRYNEPPDWTDILAHFRGSELQNYFTRLLEDELKAVIKPQYVDQIPKAIKGTVKELLAKKNDLGKQDHLCDLLELVNVKDRQIADLSGGELQRFATAMVCIQRGDIFMFDEPSSYLDVKQRLKAAVAIRGQLEATKYVIVVEHDLSVLDYLSDFICCLYGTPGCYGVVTMPFSVAEGINIFLDGYVPTENLRFRESALKFKVSDNTDEEVKRLARYEYPSMSKTMGDFELVVEGGSFTDSEIIVMLGENGTGKTTLIRLLAGRLKPDGDEEVPTLNISYKPQKISPKSTGSVRSLLHEKIRDAYQHPQFVADVMKPLLIDNIIDQEVQNLSGGELQRVALALCLGKPADVYLIDEPSAYLDSEQRLVAAKVIKRFILHAKKTGFVVEHDFIMATYLADRVIVFEGRPSVSSRATVPQSLLVGMNRFLELLNITFRRARNNFRPRINKLNSVKDSEQKRNGTYFFLEDEEDGEDLFGPELDRYDVAEEEMRRRDR--VPVPGESIENLEDMKGHTVKEWVTQGPKTEIFNRFKNFLRTYKEKIRAMEQNRMSLEVDYTLAQSEQVLAFFLPEAPAEVLPIFDEAAKDIVVGMFPHYSRIHPEIRVRITDLPILEEIRTLRKIHIDQLIRTSGVVTSTTGVLPQLRMVKYDCVKCKYILGPFVQSQEVKPSSCPECQSTGPFAINVSQTIFQDYQRITIQESPGKVNAGRLPRSKDAILLNDLCDSCRPGDEIELTGIYSNKFEGSLNKANGFPVFATVIIANHILRKDTDDDVKEVVKLSKEELAERIMASIGPSIYGHDDIKRAIALSLFGGVSKNPGQKHRIRGDINVLLCGDPGTAKSQFLKYVQQIAPRAVYTTGQGATAVGLTAYVSPVTRDWTLEAGALVLADKGVCLIDEFDKMNDSDRTSIHEAMEQQTISIAKAGIVTSLRARCTIIAAANPIGGRYDTFHQNVNLSDPILSRFDVLCVVRDERLARFVVDSHARHHPINQDLLQKYILYAREKIEPKLDQDKIAQLYSDLRRESMSTGSMPITIRHLESIIRLAESHARMHLREHVDVNMAIRVMLDSFVTQKFSVMRMGLIDDIQQSFGSENLYDVFGVPKTADADSIKKAYRRKSLLCHPDKAGQEDRDEFTRKFQLLSKCYEILRDPEKRKIYDETGEV--DDSLNSTSDWQGYWRRMFPKVTVTQITDFMNRYIGSDTEREDLKHIYEKCKGDMNKISEYHIGYEEDRLRSIIHELIEAGDVPNYTAFSKETAASKRKRRQRYTREAQELEE------KSYGDLALLIHQNEQRESESFLASLEAKYASKPKKKSVGTKRK????????????????????????????????????????????????????????????????????????????????????????????????????????????????????????????????????????????????????????????????????????????????????????????????????????????MEMEVYVEAKHTALKHVITMLQMPDQLDKVEQHRKRVQRKKASVEAMLKTAVQSQLEGVRTGLSLLATCLDDASDIRKEEADAIYELAKLLQLQDVREESIRHSQTGTLMEHLKHIFNVPGSVTRTQDLIQDGKLLLAHKLSDLECSRDELLYELHKQANNQASDRAMLKQYFGDVERLSDDLAKQLWLILKRTLNTVRKEPQVIVTALRLIMREEWAALKRQEST-GFLPPGRPKLWRKKAMETLEQSVAERLEANQIEGRSENKMWLVRHLEVTRQLIIDDLKTVKHHCTPCFPPSFDIFNEVRMTHNCLSQRLQTIIAGLVDSEYIHVLSWLNTYNSRELMSHPDIHVDVALLPDTIEKLMERYLAGLHTKFDEWLRNALNDHKDWPEQDTDGYYRTEAPMLIYQMITQHIEVARTVKLVSRVLKLAMDHMGKFLKDYTDYVTEYNFEDRSYTAYMIAIANNSVYMK-LSDLKRNALSYLCEEVLTDIKLMEDIMTETVTVTLADYGEDYIEKQVASSYVKAIC--EKRMSFKNYEERKSAAEFEKLRNSELEVLKLMAEVLKMKDSSLLSLEMNG---------------------------------------------------------------------------------MMKLIINSLYRNKEVFLRELISNASDALDKIRLLSLTNPDALKALQDLKIRIMADKENNVLHITDTGIGMTKEDLMKNLGTIAKSGTAEFLQKVSDGSGGTDLNDLIGQFGVGFYSAFLVADRVAVASKHNDDPVQHVWESNASEFSVTEDPRGDTLKRGTTVSLYMKDEAKDFLEHDTLKKLIEKYSQFINFNIYLWSSKT-VTEEIAEAE--TTE-----ADDDDAKVEDEKEP--PKTKKVEKTVWDWELVNSAKPIWTRKEKDVSDEEYNEFYKTVTRDSQNPLARTHFTAEGELTFKSLLFVPQRQPQDSFNRYGQKTDNIKLYVRRVFITDDFQDMLPNYLSFLRGVVDSDDLPLNVSRENLQQHKLLKVIKKKLVRKALEMFRKISDEDYEKFWKEYSTNIKLGVIEDSANRSRLAKLLRFPSSNDSTGKLVSLSEYVERMKEKQTAIYYVAGGSLDEVQKSPFVERLLKRGYEVLYLTEPVDEYAISSLTEFEGKKFQNVAKEGLSLDD---NKDVREALEKEFEPLTKWLTETGLKDKISKAVVSERLVETPMALVASQFGWTGNMERIVSAQTHMKENDPQRQFYLGQKKTLEINPRHPLIKDLLRRVDDSPSDETAKYLTEMMFDTATLRSGFQLGDHAQFASNVEKMLRKMMGVSEDAQIDAEPEEADLPEETAEDRAEDIESEPHDELGRRGEVKELSAEEQKMAVISEIIQELVSAHHERRDVNLNRVKGDASSRHGLKSQPKLVDIIAAIPPQFKQLLLPKLKAKPVRTASGIAVVAVMCKPHRCPHIAYTGNICVYCPGGPDSDFEYSTQSYTGYEPTSMRAIRARYDPFLQTRHRVEQLKQLGHDVDKIEFIVMGGTFMSLPEDYRDFFIRNLHDALSGYTSANVDEAVKYSERSKTKCIGITIETRPDYCLQRHLSDMLRYGCTRLEIGVQSVYEDVARDTNRGHTVKAVCETFHMAKDAGFKVVTHMMPDLPNVDFERDVLQFVELFKNPDFRMDGLKIYPTLVIRGTGLYELWKTGRYKSYPPALLVDLIAKILSLVPPWVRIYRVQRDIPMPLVSSGVENGNLRELALARMKDLGLVCRDVRTREVGIQEIHNKVLPYQIELIRRDYVANGGWETFLAYEDPQQDILVGLLRLRKCTEQTFRPELVGQCSIVREHVYGSVVPVHSRDPSKFQHQGFGTLLMEEAERISRDEHGSTKMVVISGVGTRNYYRKLGYELDGPYMSK

>Tropilaelaps_mercedesae

PGARVNVLSKELMASVVLISGKKGCFIAGADITMLEECQSEAKRLKPVVAAIMGSCLGGGLETALACRYR-IAVEDSKTVMALPEVMLGILPGGGGTQRLPRLIQLPTALDMMLTGKNIRAAKAKKMGLIDATVKPLGPGIYLEEIAARNLASEKLKIRVRPLTERLVRDM-IFNKAREQVMKLTNGLYPAPLRILDVVRAGIEKQGFAELCTKEARSLMGLYHGQVQCKKNAFGKPTDNVAILGAGLMGAGICQVSLKDFNKVVMKDGLVRGQNQIKKKKIGKDKLMSKLLPSLDYSDMIIEAVFEDINVKHKVVKEVEAAHCIFASNTSALPITKIAEASKRPDKVVGMHYFSPVEKMMLLEIITTDKTSKDTAAAAVDVGLRQGKVVIVVKDGPGFYTTRILAPMMCEATVLLMEGCKVKELDKLKEFGFPVGGATLLDEVGVDVGAHIAEGVFGERKEMVSNFLGRKSGKGCYIYRPVNPIQRRYTVEQMQYRLATRFINEA------------------------------------------------------------------------MQRPIILLQEGTESQQGKTHVMSNINACQAISDAVRTTLGPRGMDKLMVDSRGKTVISNDGATIMKQLDIVHPAARTLVDIAKSQDSEVGDGTTSVVLLAGEFLKQAKPYIEEGLHPQIIAKAYRKASKMAIEKIHEIAVKVDKGEMKALLEKCAMTTLSSKLVAAKKEFFAKMVVDAVLQLDELLPLNMIGIKKVSGGALEDSLLVSGVAFKKTFSYAGFEMQPKQYNQPKIALLNIELELKAERDNAEIRVSNVEEYQKIVDAEWNILYDKLAKIHASGAKVVLSKLPIGDVATQYFADRDMFCAGRVAEEDLRRTMKACGGCVLTTVQDLRDSNLGSCERFEEVQIGGERYNIFKGCPNSKTVTLILRGGAEQFIDETERSLHDAIMIVRRAVKNDAVVAGGGAIEMELSKYLRDHSRTVAGKEQLLIAAFAKALEVIPRQLCDNAGFDATNILNRLRERHAKADRWAGVDVNSEDVADNLAACVWEPAVVKVNALTAATEAACLILSVDETIKAPQSNTDPSVGRPFSKTLNRENAFREHGMAVTRDYISQPRMIYKTVCGVNGPLVILDQVKFPKYAEIVQLVLADGTPRTGQVLEVSGDRAVVQVFEGTAGIDAKNTVCEFTGDILRIPVSEDMLGRVFNGSGKPIDKGPPVLAEDFLDIQGQPINPWSRIYPEEMIQTGISAIDVMNSIARGQKIPIFSASGLPHNNIAAQICRQGGLVKRPEKSVKDDNFAIVFAAMGVNMETARFFKQDFEENGSMDNVCLFLNLANDPTIERIITPRLALTTAEFLAYQCEKHVLVILTDMSSYAEALREVSAAREEVPGRRGFPGYMYTDLATIYERAGRVEGRNGSITQIPILTMPNDDITHPIPDLTGYITEGQIYVDRQLHNRQVYPPINVLPSLSRLMKSAIGEGFTRKDHADVSNQLYACYAIGKDVQAMKAVVGEEALSPEDMLYLEFLGKFEKNFISQGRYENRTIFESLDIGWNLLRIFPKEMLKRIQHSLLAEFYPRAEAKRCLNTAQFNN-TGLFGDPTGFILLKENAIAKAEELTAEATSRRRKMVQIFDDLSDCLCKVADLAEFVRVGHPQVRYAHAAEDASLAISSLVEKLNTNRELYSALRSVIEKG-DIVPTTAEQHVGRLFLFDFEQCGIHLDEQRRQQVVALNDHILFVGGRFLQDSHQPRYILKSSMPENILVVSGLQADCSNEVVREAAYRIYFRPDEHQLHLLDELLESRHRLARLCGFDTYAHRVLKGSIAGTPENVKFLSYLSTELKPRSQRDYQEMLGMKAWDVPYYTAYLSLANCMEGLDMLFNALYGIKLEVVGELWHSSVVKVAVRDPSPTMGVIYCDLFERPGKPHQDCHFTIQGGRRSDGSYQTPKVVLMLNLPPPLLTPSLMDNLFHEMGHAMHSMLARTEYQHVTGTRCATDLAEVPSILMEYFASDPRVVSQFARHYRTGEPMPAEMAANLEASRVIFQASETQLQVFYAFVDHEYHSKYPLSTTDVLREVQNRHFGVGYVENTAWQLRFGHLVGYGAKYYSYLMSKAVAATFWHRVFHTNPFSRSAGTYREEVLAHGGALPPAQLIENFLLAESLIRDIMPSDAKKKRDAKKKEALKNRNNPDTPNGEDEMDEVTRKFDEDMKLNAAARAVTGVLSIHPRSRDVKIENLSITFHGWEVLQDSKLELNCGRRYGLIGLNGCGKSTLLSAIGRRELPVQDCLDIYHLTRECPPSEKTALQMVLDVDKERARLERLAEELASAEDDTSQEQLMDVYERLDNMSADTAQGKASYILHGLGFTQAMMSKKCKDFSGGWRMRIALARALYVKPHILLLDEPTNHLDLDACVWLEEELKSYSRILILISHSQDFLNGVCTNIIHMNLRKLEYYGGNYDQFVITRSEVLENQMKRYNWEQLQIAHMKDYIARFGHGSAKLARQAQSKEKTLAKMVAGGLTDKVVYDKTVSFYFPSCGTIPPPVIMVQNVSFRYSIDTPLIYKNLEFGMDLDTRVALVGPNGAGKSTLLKLLCGALVPTDGIIRTHSHLKIARYHQHLHESLDVDLSALEYMMKSFPDVREKEEMRKIIGRYGLTGRQQ---------------------------------------------------------------------------------------------------------MVLADLGRRITSALRNLSTATVINQEVLDSMLKEICAALLESDINVRLVKQLRENVKAAIDIEEMAVGLNRRKVVQSAVFKELVKLVDPGVKSWHPSKGRSNVIMFVGLQGSGKTTTCTKLAYYYMKKGWKTALVCADTFRAGAFDQLKQNATKARIPFYGSYTEVDPVVIAADGVAKFKTEHFEIIIVDTSGRHKQEDSLFEEMLEVSNAVTPDNVIFVMDASIGQACELQARAFKEKVDVASVIITKLDGHAKGGGALSAVAATRSPVIFIGTGEHIDDFEPFRVKPFIQKLLGLGDIEGLIDKVNELKLDENHELIEKLKHGEFTLRDMYEQFQNIMKMGPFNQIMGMIPGFSADFMSKGNEQESMARLKRLMTMMDSMTDEELDDREGAKLFLKQQTRITRVARGSGCTTFEVHELLNQYTKFAAMVKKMGGMKGLFKGNDLARNVNPAQMNKLSVEMAKMIDPRVLQQMGGFSGIQNMMRQMNASILTEDAAFIELKKLHSTKGKSLNLNTLFKDDLSRAGKYTIKLSEGPETLLIDYSKNLIDDEIFTTLLELAKNRQVETMRDRMFSGEKINFTENRSVLHVALRNRSNRPIEVDGADVMPGVNAVLEHMKIFCQQIISGEWKGYTGKKITDIVNIGIGGSDLGPLMVTEALKSFQVGPRAHFVSNVDGTHLFETLKKVDPETTLFIIASKTFTTQETLTNADSAKAWFLGKAGDKAHVAKHFVALSTNKAKVESFGIDPTNMFEFWDWVGGRYSLWSAIGLSIALFIGVPNFEKLLAGAHFMDEHFRTAPLEKNVPVILAVLGVWYINMFGAESHCVLPYDQYLHRFPAYFQQGDMESNGKYVQRNGVQVSYQTGPIVWGEPGTNGQHAFYQLIHQGTKLIPCDFIAPVKTHNPVQGGLHHKILLANFLAQTEALMKGKSSQEAKAELEASGINGEELAKILPHKVFQGNKPTNTIIVQQVTPFTLGALIAMYEHKIFVQGVIWDINSYDQWGVELGKQLAKVIQPELDGKDPVSSHDASTNQLINFIKSFNKNVSYQDRDKPAQVRQSNITAAKAVCDAIRTSLGPRGMDKMIQAVNGDVTITNDGATILQQMQVLHPAAKMLVELSRAQDVEAGDGTTSVVVIAGSLLDAASKLLIRGMHPTIVSDAFQVAAKECVDILSNLAISVELSDRESLLKSATTSLSSKVVSQHSDILAPMAVDAVLKVIDPNNVDLRDIRIIKKLGGTVEDTELIDGLVFTEKLAGGNAPHRMEKAKIGLIQFCISPPKPNMDHQVIVSDYSAMDRVLREERAYLLNIVKVVKKAGCNVLLIQKSILRDAVSDLALHFLAKMKIMVIKDIERENIEFITKSLGCRPIASLDHFVPESLGSAELVEEVTSAKYVKVTGVANP---KTVSLLLRGSNKLVLEEADRSIHDALCVIRCLVKKRALVAGGGAPEIELSLRLAERARQIEGLHSYCYRAFADSLEIIPYTLAENAGLNPIQTVTELRNRHAKGERTCGINVRRGCVSDITKENVLQPLLVTTSAITLAAECVRSILKIDDIVQTVRMSKRDVVYLWDPDVGNFHYGPGHPMKPQRIAVTHSLVLNYNLHTKMRIYRPYRANPHDMCKFHSEEYVNFIERVTPKNIQTFSKSLTHFNVGDDCPVFDGLYDFCSMYTGASIDGAWRLNNKSCDIAINWSGGLHHAKKFEASGFCYINDIVVAILELLKYHARVLYIDIDVHHGDGVQEAFYLTDRVMTVSLHKYGAYFFPGTGDMYEVGAESGKYYALNVPLKEGIDDASYYQVFKSVISSVIEHYKPGAIVLQCGADSLAGDRLGCFNLSIKGHGECVRFVRDLNIPLLVLGGGGYTLRNVARAWTNETAILVDEQVSPEIPYNEYLEFFAPDFSLYPD-------ENANSKQYLEAIIKYTTENLRCLDHAPSVQMQDVPPDLADIGDDQEKRDSTKEHPAELFD---------------MSDAEEDYELEYSEDDDSQPDVDLENQYYNSKALKEDDPQAALQSFQKVLDLEGGQKGDYGFKALKQMVKINFQLGHYEEMMSRYKQLLTYIRTAVTRNYSEKSINSILDYISTSKRMQLLQEFYEVTLDALRDAKNDRLWFKTNTKLGKLYLDREEWPRLARILRQLHLSCQNVDGSDDLRKGTQLLEIYALEIQMYTSQKNNKELKKLYEASLQIKSAIAHPLIMGVIRECGGKMHLREGEYNSAHTDFFEAFKNYDESGSPRRTTCLKYLVLASMLMQKEINVLDSQEAKPYKDDPEIVALTDLVDAYQAHDISRFESIVSPHKESIMKDSFIKEHIEQLLLNIRRQVLIRLIRPYTRITISFISRELNIPSAEVESLLVSCILDNTIHGRIDQVKQVLELHP-SPTDRRYTAMEKWAAQVQLIQNTVPMLLAHLGTLNPVQILNEQAEEEKAENARLSSFVGAIAIGDLLKSTLGPKGMDKILLCETSRDSKVEVTNDGATILKAIGIDNPAAKVLVDISKTQDDEVGDGTTSVAVLAAQLLQEAEKLIAMRLHPQTIIAGWRQAVVAARTALEDFSQDRSQNETQFRIDVLNIARTTLGSKILAQHKDFFAQLAVDAVTRLKGKCNLDAIHIIKKLGGSMLDSYLEQGFLLDKKPGMNQPRRVENAQILIANTPMDSDKIKVFGSRMKCDSIAKVAELEEAEKLKMKRKVDSILAHKCNVFINRQLIYNYPEQLFADAGVMAIEHADFDGIERLALVTGGEIVSTFTSPEAVQLGTCELIEEVMIGEDKLLRFSGVPVGEACTVVLRGATQQILDEAERSLHDALCVLASVVKEKKICYGGGSGEMLMAAAVEKLAQTTPGKEALAIEAFARALRQLPTIIADNAGLDSAKLVAELRAAHANGQNTFGINIVDAKVDDMQKLGVTEAFVVKRQVLLSAAEAAEMILRVDSIIKDAPRKRVPDKSH----MKSTDPFPFLCTLQQHRPYVGQVVLIGNADAVISVSDDRTIRVWARRDTGQYWPSVCYTMPAAASALNYDQPSRRLFVAMDNGSITEFELADDLNKIIFRRSYIAHQQRVTSIKFSPTTEWLLSAGKDKYFQWHCTETGRRLGAFQGSAWCTTVELDEASRHAFIGDYSGHVTMLKLTETSYQPVTTLRGHSGSVQSLLWDERRNLLISGSFDQVIIVWDIGGGKGTAYELSGHRARITGLALYSASSLLSVSEDSTLVVWDVAAQRQETPDWNERDFCERCARPFFWNVRAMLDQKQVGLRQHHCRRCGRALCDRCSANRSALPRLGFEFPVRICDECHLHISDGDRQPLASFHDLKAPVTAMSLSAASKTLVTVGTDRSIKIWDLSKVLMADSANESAASKKALKKAQKEAEKAAKKAAKKAERAAGKEDQSGDVSEGFYGQYHMIQSTDTPQREIIKVSQCTLQLADETIWLRGRLQTSRAKGKQCFFILREQQFTLQCLLAVGEKTSRQMVKYIANISRESLVDVQGKLVKSPSKIESCSQQEVELQVLQFWVVSLSDARLPLQVEDAARPEPAEGDEEGLKIRVNQDTRLDNRILDLRTPANQAIFRLQAGVCHLFRESLNRRGFIEIHTPKIISAASEGGANVFEVTYFKSKAYLAQSPQLYKQMAIAADFDKVYTIGEVFRAEDSNTHRHLTEFVGLDLEMAFKYHYHEVVDIIAAMFVDMFKGLRDRYQPEIDAINKQYPAEPFKFLDPSLRLEYAEGVAMLRAAGVEMADDEDLSTPNEKLLGRLVKAKYDTDFYVLDKFPLAVRPFYTMPDPTNEKWSNSYDFFMRGEEIMSGAQRIHDSEFLTRRAKAHGIDISTIQSYIDSFKYGAPPHAGGGIGLERVAMLYLGLDNVRKTSMFPRDPKRLTPAMEKGLQLNEIDPLEFSRVKIMYPKECRIRRVTYRGKLDLTFWWVNGLKQEPVKRSCGEIPIMVKSRKCNLAGLNPQQTVGHGEEMEEFGGYFVVNGNEKVIRLLIMQRRNYPIAMARNGWKNRGAMFSEFGVSLRSCRRDGQNMVLHYLTNGTVQVMITFRKEVYFVPALLLLKALVDKSDYEIYKALTKGCENDSFYKGCITNMQRLVQENIMTCDAKEFIGDKFRWASNAYVCDKLLDRCVCVHLDSNEDKFNLMCFMVRKLFAVAKNRCALESADSTMNQEVLLPGHIFLSVLKEKIEGLLIGVKLSIDKKMSSAMDVTNAMNYFLSTGNLVSKTGLSLQQTSGFTIIAEKLNFWRYLAHFRCIHRGSFFMEMRTTTVRKLLPEAWGFICPVHTPDGGPCGLLLHLSSMCEIVVQLDGKCVGWVIRYLKVSGDIPPTLEICVIPKTEQNSLFPGFYMFSTPARMMRPVFNRTQTIEWIGTLEQVHMDICVISEEATTHQELRETAMLSVLANQIPWPDFNQSPRNMYQCQMGKQTMGSPMHAFRNRADNKLYRLYPQSALVRPTAYDHFKMDEYPSGTNAIVAVISYTGYDMEDAIVVNKMSVERGFKTGVYKTETINLRVDVDGLPYIGCHGDPVCAYIQLKTVKYHSTEPATIHEVKILGNLQQIQLTYLVRTPMIGDKFASRAGQKGICSTLWPTESMPFTDSGMVPDIIFNPHGFPSRMTIGMVVESMAGKSAALHGYVHDASPFKFSEENPSSAYFGDLLRRAGYNYHGTERMYSGVDGREMDADIFFGVVYYQRLRHMVADKYQVRTTGPIDSLTRQPVKGRKRGGGIRFGEMERDSLLAHGTAFLLHDRLFNCSDKTLCIACGSVLSVPIPYVFRYLVAE-MVSVNMKIIRETSRDLHRHQRNAPELHPLAITREYTAAVNAAKLDRIFAKPFLCSLGHRDAVELLAKHPDRISGAVSASADGELRWWDLTNRMCVRSLQAHDGPIRGLTGQDQSIKTILSKLTHHYSTTFATAGETVSLWEERNEPLRSFW-GIDTVYVYFSPIESLSSDRSIVLYDIREASPLRKVVLEMRCNALAFNPMQAMHFTVANENYNLYTFDMRSLNKALQSHTDHVGAVLSVDYSPTGTEFVSGSYDKTVRIYRSREVYHTKRMQRVTSVMYSLDAKYILSASDEMNIRLWKAKASEQLGIQNYNATLLERFGYHPEVKRIVRHRHVPKQLYQEKQTMVAAKKRKMGMQLLQKGAYQDALSHYHAAIEGDDRNYQSYYWRATVYLALGKSKLAVEDLDRVVELKDDFVKAREQRGNILLKQGHLDEAHIDYEFILRLDPHNVEAIIEELKNDVIHILQRVWNLKFREMRASCYEAIGDIQAAITDLRPAIRSVPDNTGGYFRLAELYRKFGEPDDALNTIRECLKLDPDHKECYKNIKRLVKSMQEECVEKIVTLIRNKCQCASKGGNAVKICSEALQLDPILCDRGEAYINQDDFAQDFAAARELDSRAAEGLKRAQKLEKAQGRRDYYKILANKGEISKAYRKLAAKWHPDQY-QGDD--KKNAEKKFIDIAAAKEVLTDPEKRAKFDRGEDPLDPESGFHYTFHFMLVLFESPAGYAIFKVLDEKKVQKTDNLFKEFEDASGAAKVLKLKHFQKFEDMTQALSAATGAIEGKLCKPLKKVLKKLAANQAHETLAVADAKLGSIIKEKMDISCVANSSIQELMRCIRSQQEALITGLSQKEVTAMALGLAHSLSRYKLKFSPDKVDTMIIQAVSLLDDLDKELNNYVMRCKEWYGWHFPEMSKVVTDNMLYVKTVRKMGMRSNAINMDLSDLLPEDQEAKVKELAEVSMGTEIASDDVENIKHLCDEVIQMTEYRATLYEYLKNRMTAVAPNLTVLVGELVGARLIAHAGSLLNLSKQPASTVQILGAEKALFRAFKTKHDTPKYGLIYHAQMVGQSSQKCKGKASRWLAAKAALAIRVDALGDDTGTEMSLKNRANLEARLKMLEEGKLTKISKVGSKPNRFDVYRHTSEVLDYKPASDSTLP-SKKRKFADDDENADTPKQFKSK------------RFEDEDMKLKVDEASEEQT---------SVEIEKKKKKKKKSK---SMKAEEDQDE--------------------KYVWPKERPDIRRTVTLALGILVTAKLVNVSVPFIFKYLIDFLNQTTGLVLAYGLARAGSAGLNELRNAVFASVAQHSIRSMGKRLFMHLHDLDLSFHLQRQTGALSKAMDRGTRGINFVLTALVFNVVPTIFEVALVSTILWYKCGQFAVVTLGCIGTLAVTQWRTQFRVDMNKADNRAGSRAIDSLLNYETVKYFSNEKYELSDYERASLKTTVSLAGLNFGQNAIFSAAVMYLACQLTVGDLVMVNGLLFQLSLPLNFLGSVYREVRQSLIDMQTMFALSEIKTKEIIISREIEFDNVTFQKILDGVSFSIPTGKKVALVGGSGSGKSTTVRLLFRFFDPSNGRVLINGQDIRDVSLRHGIAVVPQDAVLFHDTIHFNLSYGDLTAEVENAAKMAEIHDSIKAWYDTQVGERGLKLSGGEKQRVAIARAILKDAPILIFDEATSSLDSITEHKIMMALRAASGKTTLCIAHRLSTIVDADQIYVLRNGSIIEAGHQTLLASFYAHLWNQQHSFLLRRHMNHLARTFSQCLQNIPETRVTTLANGIRVATEDNGAPTATVGIWIDAGSRYESEKNNGVAHFLEHMAFKGTGKRSQTELELEVENAGMHLNAYTSREQTVYYAKCLTKDLARAVDIIADITQNPKLGEQEIERERGVILREMEEVEGNLQEVVFDHLHSVAYQGTPLGLTILGPTANIKSITRQDLKDYIDCHYKGPRIVLAGAGGVDHDELVKIAEQTFGKVSASPENFVPCRYTGSDVRVRDDDMPFAHVAIAIEGAGWTNPDNIPLMVANTMIGSWDRSHGGGANASSKLASLAATARSLHSFQSFNTCYKDTGLWGLYFVADGDELDDIMFAVQDEWMRICLSATESDATRAKNLLKTNLLLQLDGTTPICEDIGRQMLCYGRRIPLPELEARIDAVDAKAIRDVCLKYIYDRCPVVAAVGPVEGLTDYVRIRGQMYKHSVHTLVFRSLKRSHDMFLCDEGALPPVDETAQKFRIGTKARDEYGSVMHLVSEGRRATASGALVLAGQQLTAQLKKPSIPRPQWHPPWKLYRVISGHTGWVRCVAFDPTNEWFCTGSNDRIIKIWDLASGKLKLSLTGHISGVRGLAVSQHHPYLFSCGEDKQVKCWDLEQNKVIRHYHGHLSGVYTIGLHPTIDVIVTGGRDSTARVWDMRTKANIHVLSGHTNTVASVLVQATEPQVVSGSHDSTIRLWDIVAGKTRVTLTHHKKSVRALVLHPKLNMFASGAPDNIKQWMCPDGKFIQNLSGHNTIVNCLAMNEDGVLVSGGDNGSLQFWDWKTGYNFQKLTTPVQPGSIDSEAGIFAMGFDLSGTRLVTCEADKTIKIFKEDETASEETHPINWRPEIVTGSTSEYLINCSAEHYLEFLGINVKAKNFLVFQGAVESIAMKNPKERTVLFEEISRSMMVAEEETYQKKKGIAAERKEAQIEEAEKYQKLKEDVQVNLHLFRLFHQEEELQKKKRKDKIEAELKEKKKVEQNHRDDVELNKRKPAYIKAKEKTAHMQKKLDAAKKSLEAATKTHKSHQGIEELEHELSQVEEAFEQEDVSLEESQVKEYNRLKEKAGKMASAALQEYDSVAREQKTDQDHLDNELRKRNECEAKLKELEENQRRVNKLVDLHDLKEEEKKLEAKKRLTQKFEDVASLGDAKVDKHEDARRKRKSEIVEHFKKLYPGVHDRLVNLCHPIHKKYNVALTKVLGRNMEAIVVDTEKTGRACIQYLKEQMLEAETFLPLDYIDFKPLKERLREFRDVPNVKLLYDVLKYEPLSIKKAVLYATNNALVCETAEDAAKVAFQSPDGKRYDAVALDGTYYQKNGFISGGSSDLAKRAKRWDDKDFHKLKDQKEKLQEDLREAMKTARKESDLTTIESQIKGLETRIKYSKVKQLEQSMREREGRINEIKSRQNTVEDDVFRVFCEQIGVANIREYEERELHASQEREQRAELENQKNRIASHLEYER--TKDTLAVEQDQQELARLKDIEQKQKELIEQQMETISALKNERQSKKMKVDEIDEEVAEIRKRLTAQQKEVTGVQKTVTQAEARLEQKRSERHTLLQSCKLEGIRIPLIRGSMSQMYAREALQINYSHLRMEKEINLQRIQAPNFKAMEKLDSVKERLKDTDTEFEHARRKAKSNFELVKRERTCFEHVSNCIDEIYKSLTNNPSAQAFLGPENPEEPYLEGINYNCVAPGKRFQPMSNLSGGEKTVAALALLFAIHSYQPAPFFVLDEIDAALDNTNIGKVARFIREKTQTSFQCIVISLKEEFYGHADCLVGICPDPGECTISRIYTIDLSMSEAMLLYLGQAIRDGNIAEMQNIYEMSLVNLMDQYLIIIYKE--LYYRQIYAEERFSSYYNYVNLFNYILSSGPVSLDLPNQWLWEIMDDFVYQF--QSFSTFMWNVHSVLNVLHSLVQKSNINQQLEVFSRGGNPDEVAGEYGSRSLYKMLGYFSLIALLRLHSQLGDYYQAIKVLQHLELNRKGLSRVPACQVSTYYYVGFAYMMMRRYEDAIRTFSNVLVYIGRAKRSYQQRQMNNQTEKMYALLSICMVLHPQRMDESVLQQLKLKHMLKMSQGDIDTFERCPRFLSPVQWRAFRGEVLAQMTIRSFLKLYTTMPVEKLTKFVLMCFKHLMSNVVLDGEFQTGSDMDFFIDKDMIHIADTKIARKYGEFFAKQYNRFEEWYEMAALRSGALLMPRAVQVLGARSQSLFSHVEMGPPDAILGVTEAYKKDSNPKKMNLGVGAYRDDNGKPYVLPSVRAAEQQLMLKSLDKEYLGIAGLAEFCKNSAALALGANSAVTKEGRNATVQGISGTGSLRIGAIFLDEFLKGNKTVYMPNPTWGNHIPLFKKCNFQVKQYRYYDPKTCGLDFPGALEDISRIPEGSVILLHACAHNPTGVDPRPEQWTEIEKVVRKRNLFPFLDMAYQGFATGDIDRDAMAVRLFAASG-PMCLSQSFAKNMGLYGERIGAFTLICDSAEEAARCMSQIKILIRPLYSNPPVNGARIANLILSDAQLRSQWLIDVKEMANRIISMRTRLRDGLKREGSSRDWKHITDQIGMFCFTGMTPEQVSKLTRDYSVYLTKDGRISVAGISSNNVDYLAHAMHNVTKRT-PGIFHLLHCMLMATVVFAFMNFSKPPDEVDPFSEYGVIFTIILYLFRLLPLLALPQSLTNLFGLTLYNAFPPKVRLKVKPHDAPFLCIRVVTRGDYPALVRENVERNLATCLETGINNFVIEVVTDKEVYVAVNSKIRQTVVPKSYNTSTGAMFKARALQYCLEDSVNSLAEGDYILHLDEETLLTKDALRGVINFISAGRHPFGQGLITYANERVVNWFTTSADMYRVADDLGKLRFQFNFFHKPLFSWKGSYVCTRFGAERDVSFDHGPDGSVAEDCYFSMVAYAKGYSFEFIEGALWEKSPFTVGDLIQQRKRWLQGIYLVVHSGKIPRRFKIWLSCSLYAWATMPLSTSNLLLAPTFPLPCPQAFNVVCAFIGALNIYMYVFGLIKSFSIRRHGFFGFWLCLVLVVLAIPLNIVVENVAVIWGLLGDKHKFYIVEKNIVMETQTNGCGEPAKLQCPTCLKLGIKGSFFCNQSCFRNSWNTHKAIHKTVKNNPYNPWPHYHFSGKLRPGRITPKRPVPAHIARPDYADHPEGVPISEEAMKGA-EIKVLSKTEQEGVRKASVLARECLDVALAAAKPGVTTDELDRLVHEAAIARNCYPSPLNYYKFPKSCCTSVNEVICHGIPDERPLEDGDILNVDVTVYHNGFHGDLNETVFIGKVDEAAKKLVRVTYDALQKAIEACRPGVLYRDIGNIIQKHVQQNGFSVVKSYCGHGIHSLFHTAPSVPHYAKNRAVGVMKPGHCFTIEPMISEGAWHDEVWPDNWTAVTTDGKRSAQFEQTLLVTDRGVEILTSRREKNGQPWFMDQSTLRDSLKERSAKRRKLLAQQLGAGCAENLGLLLGNDKTSTTKQGTAPQEEEVMAYRDSSTFLKGTQSANPHNDYCQHFVDTGQRPQNFIRDVGIQDRFEEYPKLKELIKLKDELIRETATPPMYLKCDLLQYNLCELNGKFDVILIEPPLEEYQRSCGVTNIRFWSWEEIMKLEIEEVAAPRSFVFLWCGSSDGLDLGRQCLRKWGFRRCEDICWIKTNISNSKVKNVEPRAVFQRTKEHCLMGIKGTVRRSTDGDFIHANVDIDLIISEEPPFGMMEKPEEIFHIIEHFCLGRRRLHLFGRDLTIRPGWLTLGPELTNSNLNTEAYNAHFNTANDYLTGCTERIEALRPKSPPPKTTLGGSSRGPRGRTSLNKLSLAAIDVAGKRVIMRVDFNVPLKDGKITNNQRIVAALPSVMHCLDKGAKSVVLMSHLGRPDGQPNENYTLGPVAEELKKLLVKPVIFLKDCCGSEVEAVCADPSPGSVILLENLRFHVEEEGKGVDASGTKVKADPSKVKAFRASLTKLGDVYVNDAFGTAHRAHSSMVGVELPQRAAGFLMMKELQYFSKALDKPARPFLAILGGAKVKDKIQLIENLLDKVDEMVIGGGMAYTFLKVNRGMQIGNSLFDEDGAAIVEKLLGKAAANNVKIHLPLDFITADKFHEDAATGTADISSGIPDGWMGLDCGPKSVELFAGAVARAKTIVWNGPAGVFEFDKFATGTKAMMDSIVGATARGAITIIGGGDTATCAAKWGTEDKVSHVSTGGGASLELLEGKVLPGVAALSDAMRTFGDRPVSFQLEDGGDYYYIGTEVGNYLRLFRGTLYKKYPSLWRRAVTVEERKKISQMNMSQHSAANFISLLKKSEVDDLIDGNEEKYRAAPVQENEGGGHGAKNARPSFMPAAPNNAHHLDAVPCSTPINRNRLQHKKNRSFPMLYDDLDPAMLHESAALPECLVPIRLDMEIEGSKLRDTFTWNRHEAHISPEQFAELLCDDLDLPPLLFVPQIAASMRQQIEAFPSDSLLEEQTDQRVLIKLNIHVGNISLVDQFEWDMSERANSPEEFAAKLCSDLGLGGEFVTAIAYSIRGQLAWHQRTYAFSEAPLGQLEMPFRAQSEAEQWCPFLETLTDQEMEKKIRDQDRNTRRMRRLANTG-MGANGSTIERDDVLPSSERYLGLVNFGNTCYCNSVLQALYYCKPFREKVLEYKAKNKRTRETLLTCLADLFHNIHSHKKKTGTLAPKKFIARLRKDNEVFDNYLQQDAHEFLNYLLNTIGDLLQAESSWVHDIFQGTLVNETRCLTCETVSSKDEDFLDLSVDISPNTSISHCLRGFSSTETLRGEHKYHCEQCNSKQEAQKSLKVKKLPPILALHLKRFKYTEQQNRNTKLSWRVVFPLELRLFNTSDDALNGDRLYDLVAIVVHCGTGPNRGHYISIVKSHGLWLLFDDDIVDKIDPSTIDDFFGLTQDTPKSSESGYILFYQSKEKSDVDHWIELGKQCRYLPEADLKKLCNMVCQILIEENNVQPVSSPVTVCGDIHGQFYDLEELFRCGGQVPDTNYVFMGDFVDRGYYSLETFTRLLTLKAKWPKKMTLLRGNHESRQITQVYGFYDECQQKYGNANAWKYCCKVFDLLTLAAIIDGEIFCVHGGLSPEIKALDQIRTIQRNQEIPHKGAFCDLVWSDPDEVDTWSCSPRGAGWLFGARATHEFMGYNALSLICRAHQLVHEGYKYMFDDKLVTVWSAPNYCYRCGNVAAVLEISDDQRKNPKIFNAVPDHERVIPERHAPYFLVDPVQPDESQDAEAATIDELKEHTAHIQKAVAQKESRFILRILRLLPATRKKLNSKLLRKTINGFYTHDKVHRELLLSFVDAEDTDMDPTQKTAHLALLPEVDVYLHLLLLVHMLDAANMERAVRCAELLKGKVEAHSRRSMDLLAAKSYFYYSRVYELDGNICMIRGFLLKRLRTATLRSDFEGQAVLINCLMRNYIHYSLFKQAAKLVSKVTFPEMASNNEWARYLYYLGCIKAIQLYYTDAHKNLLQAIRKAPQHSALGFKQTVYKLAVTVELLLGDIPDRTTFRQPALRKSLAPYFQLTQAVRNGNLGLFNQVLESYGSRFQADHTYTLIIRLRHNVIKTGVRMINLSYQRISLADVAAKLQLGSAEDAEFIVAKAIRDGVIEATIDHDKGYVQSAENIDVYCTGEPQAQFDQRISFCLDIHNQSIKAMRFPPKSYNKDLESAEERREREQQDMEYAKEEDDDTFQIAIRLTLALAALAARTLWQSSVTDMIENFRDSQPLLFEFLARLPEETAQGAILGTSVLLLCQSALASSLRMTAMRAIPVNLCIALL-DIVDEHACDAILSFLQHPDGHYPKMMADLLEQVTRCGPVVDAKRACGDHESLYSLLAGIGEIHTNLILENLLPDSTRRIELLLKILLDCVGTSGQYPSEETLSRIPITFWHILLDELGRVEPNTQMAKQLQPVYEQLVKLLRKCQLSDPGTMDSDEKEDLRCYRQDIAD--CYMSIATMLSSPVFYFFISALETAKNSKLIEACLFALNAIGDMADSEEDAPVVDAVLALLPRVPAGDEVLSQVMTAVGIFAEENIGPLVHLLLRGLQQTSAAASMALKDLARAHGDRLAPAANDILQAIAVLKHRDRVRLVAIVGHVVSALSSEQALMSLSALMAPFVQQLNEMTNLVEQLLPLFKLIAVKYSSCDPEVVSNLAECIRKAVPVLEVEVILSELLSMCGALLINATTFIDLHGCTDTVESFYKLGTLLKKFSLDDLVQLCPPLINIRCATQFVNASERSPIKEALENIIAQLIQ-VRISRNLIESEADRF------------------------------------------------KVKVKWKEVY-DVEVDLPEVFRAQLFALTGVIPDRQKVMFKGAILKDS-WGVTVLMMGTKDDVPQPTEKTVFMEDMDDSEISTAFKLPTGLTNLGNTCYMNAVVQCFKTVPELTGLEKFTGTLTSALRDLYRNMGSCTTAPLVLLHALHTLFPRFAEKGEHGQQDANECWTEMMRMLQNLIDQLFGGKLVSLKCMESEEESTEDFLQLSCFIS-SDVRYLIAGLKLRM-QESITKMSSTLNRDAVYKTSKICRLPAYLTVNLVRFFYKEKQSVSAKILKNVAFPMMLDVYELCETDLQQRLLPQREKFKKWDCEFSFEDDGSNNSGFYQLKAVLTHKGRSSSSGHYVAWIRSEWFKCDDDVVVPKEEILKLGGGGDWHVAYVLLYGPRILDGLINKKLPKELLLKIFSFLDIVSLCRCAQVSKEWNVLAMDGSNWQNIDLFSFQRDVTYDVVSYIAQRCGGFLRRISLRGCQNVPDQALSVFAQHCHNIEQVLTNCHKLSDDSVVSLSMSLHVDSCVELTDRSLRFF----NRLRVIDISWCRKITGQGIGMVAG--EQLLRFTAKGCLDNEAIIKLASKLQVLNLQCCPFLTDSAVVAVSQNCPDLRHLCVSGCTLLTDASPQALAVGCLHTLEMANCQRCGDAGLAPLLKACHDLRRLDLEECNLITDSTLNHVAAFCPLMEQLTLSHCDQITDQGVHKLAIQCIEIDNCPFISDTSLEYLADHLRRVELYDCQLITQDAIGKFQPEVRLHTYFAPATPRQRYCRCCVIASSKGARVYCPDHPGANLIEDYRAGDMICPQCGLVVGDRIVDVGTEWRVFQNEKSSNDPTRVGAAENPLLGGSDLSTIIGRTGDASDESGNAKYANRKTMSASDRALIGAFREISAMGDRINLPKTIMDRSNLLFKQVHDGRSLKGRSNDAIASACLYIACRQEGVPRTFKEICAVSKVSKKEIGRCFKLILKALETSVELITTGDFMSRFCSNLALPPSVQKAATHIARKAVEMDIVAGRSPISVAAAAIYMASQASAEKKSQKEIGDIAGVAEVTIRQSYKQMYPKAAQLFPEDFKPANEAPWVEKYRPENFSEIVGNEETVSRLEVFSRQGNVPNVILCGPPGVGKTTTILCLARLLLGSSFKDAVLELNASNDRGIEVVRNKIKMFAQTK-------------------------ALRRTMEVYSKTTRFALACNTSDKIIEPIQSRCAVIRFGKLSDAQVLAKVIYICRKENISYTEDGLEAIVYTAQGDMRQAIGNLQSTHVGLGHVNGENVFKVCDEPHPLIIKEIIEFCAKGDINEAYARMKTLHSMGYAAEDIISNMFRVTKSHELAEYIKLEFIKQIGLTHMTILQGLGSLLQLSALLANLCLVVLDKKYVKKEHSLVKPYQGSGMNMPNWDFTGTTMVSSNYIRLTRDVQSQQGSIWNKVPWEVQIQFKVHGTGKDLFGDGFAIWYTKDALQPGPVFGSRDFQGLGVFLDTYANQNGHHNHAHPYISAMINNGSLTYDHDRDGTHTELAGCEAKFRNSDYDTSISIRYEHDTLVVSTDIMGKKEWNECFRVTGVRLPTKYHFGVSAATGDLSDHHDIIGIKVFELDELREFIVPQAAPHRDHIDDSMSGTKFFFVVLFSMLFLMLYFYQKHQEKARKRFYRDQDSFDAIRDRNCRIQDINELFLPNDTVTHVPNIKRLNIDPVFPNRTNLLHIHNMAISKAFFFSFILQRAKDDEPGFMYYFMSVISDVAANRFINASAIYYAPNMSFTPSYKGFFNKTMPLFAPRAFRSDDFNDPYHLEGTSTLNTIEAIDLGAISQNYSSDQYRINEWYSAWLPDLTKRQDSKTTYTVQITGNNDTFVWHGPPAANDNPGPVKWVRPYFDCERSDKWVYGATSPIPDIYPRHTQWRHIEIPRYVAVSVMELDFERIDINQCPFGPGN-PRPNYFAGTARCKNDTTDCEPVHGYGFRRGGYQCRCKPGFRRPRIVRNPYHGELIERASKQEYENGYQCDKIGYIGVLTQNLNNYMAIGTRIDPFLGGDVVYGKDVQLENEARMAVRLANFVSGFLQIVDPKDLFAEFRVPDKPLSADQMIAEVMSIVIGDQKVVGAGVYFDYKAFFGPY--AWRQGRNERKYFVDDTTKIQIRYNSSGIKYDHFPLQYKAADVGYWTSPYFDCGGYHNSWMVTYAVPFFGWDSLRARLQFKGVVAVIELEQLEINQCNAFENTHKCDRKSSRCVPILGRGFQGGYKCECLQGYEYPYNDPITYFDGQIVEAPSRFERMTHVSGTDKSKYGGRFMVTALPGDGIGPELVGYVKEVFRYGGVPVDFEEVHLDSSRDDVDLLEQAITAVKRNGVALKGNIETRHNDPNCKSRNVELRLRLGLFANIVHVTSQPGIETRHSGIDIVLIRQNTEGEYSCEEHMSIKGVVESLKVITQLKSDEIARYAFEWAKNNGRKKITCVHKANIMKLSDGLFLRCCTEIAKDYPEIEFDNIIIDNCSMQLVSNPKQFDVLLLPNLYGNILTNLACGITGGPGIASGRNYGRDYAVFETGTRNTGKSIAGKNIANPIAMMNAGVDLLYHLNLREHAEVIASAIDKTINVDKIHTPDLGGQATTTEVVQNIIKEVQKSAMTYSGSSNEVILRDPPTDGISAVKFGQTSNQFLVASSWDGFVRLYDIQGERCRAKFDHGEPVLDTCFQGSGYVWSAGSDQSVRLFDINQGTELKAGSHDDAVRCIEYASDVSQIVTGGWDGAVKLWDPRRPVAPSSHSQDNKVYAIAIAGERIIVGTANRKVLIWDLRNMAFVLQKRDSSLKFQTRAIKAFPDKTGYVLSSIEGRVAVEYLDPSPEAQKKKYAFKCHRMKDSTKMEHIYPVNAIAFHTIHGTFATGGSDGFVNVWDGRNKKRLCQFHKFPTSISSLAFSPDGSALAIASSFQHEYRLESNPPADQIYIRHVKSYCYV-----------------------MREYKPTDATTNPSLILQAAKLPQYAALIDKAVSYGKSPCQQLEEAMDKLFVLFGNEILKIVPGRVSTEVDARLSFDKDASIAKALKLISLYEELGISKERVLIKLASTWEGIQAARVLEEQHGIHVNMTLLFNFTQAIACAEAGATLISPFVGRILDWYVANTDKKSFEPLEDPGVKSVTKIYNYYKKFRYNTVVMGASFRNTGEVKALAGCDLLTISPGLLKELANSNEAVPQHLKAENAANLQLEKISVDEKRFRWDMNEDQMATEKLSDGIRKFAADARKLEALIQEKLKMGDSVCRPLATVYAESNEASGTNAGMPAVFRAPIRPDLISFVHHQLLKNKRTPYAVSKEAGHQTSAESWGTGRAVARIPRVRGGGTHRSGQGAFGNMCRGGRMFAPTKTWRRWHRRVNVTQRRHAAASAISASGVTALVMAKGHAIEKINEVPLVVSDKIQDYRKTKQAVGLLKNIKAWDDVEKVYKSKRLRPGKGKRRNRRYKKKCGPLVIYEKDNGIVRAFRNIPGVDTCDVNALSLFKLAPGGHAGRFIIWTESAFRKLNDIFGTFNKASKVKKGYKLPRPMMTITDLGRLFKSEEIRKELRQKKSIIISKKNKPNPLKRIHLLGRLNPYAIVEKRNYALTLAAESVKSAQKKRRQEKLKTLKMKRLQNPFPKNRKLNQGAVLKKTLDAIKDLINEGTWDCSASGVSLQAMDNSHVSLVALNLRADGFDKFRCDRNLSMGMNLASMAKILKCAENNDIITLKAQDDADTVTFVFESQNQDKVSDFEMKLMNLDSEHLGIPDTDYSVVVKMPSAEFQHICRDLSQIGDSVQITCTKDGIRFSASGDLGTGNIQLSQTADVEKEEEAVIIEMQEAVTLTFALKYLNSFTKATPLSAQVCLSMSADVPLVVEYNMGHLRFYLAPKIDDSEMFARNGTK-LAATAV---RSFATSQHHNVKVAVLGASGGIGQPLSLLLKQHPGISSLSLYDIAHTPGVAADLSHINTTAQVQGFVGADQLKDALKGMEIVVIPAGVPRKPGMSRDDLFNTNAGIVRDLADACAQVCPKAMLAIIANPVNSTVPIASETFKKRGVYDPARIFGVTTLDVVRANTFIAEAKGLDPMSLSVPVVGGHAGITIIPLVSQAEPKVEFPQDQLEKLTKRIQEAGTEVVQAKAGAGSATLSMAFAGARFVFSLVSAIKGKNVVECAYVKSDIGDAGYFSTPLILGKNGMEKNLGLGKLSKFEEEMVAKAMDELKKSVKKGVDFVSKEYEWLLKYEVNDIVEQLVIAECSKRFPL-TRSDKFVM-QSKVVATLTGDSISHADITLRL-PKHSQRTIVQNDAQWKLQQIQDAGNHLMQAMNLLRFKFTSGQEVRNLMSDVMTCVGRGRACLVVPKKRTIEEIMQSRNMKSLQPPLPSDVAVSFYIQSYKLVFAVYHV-QKDSQKF-DAECHVPWLSEVLVLFTVALQLCQQLKDKVEVFQYNDFLPMFIPLFLEHNAPISEISELIGACNVCFKDAESVL--NGFVSILVCLFCERLESTTTQPVSFN--VLYNFYEGLRVEVYCSLLRVAAGAVADVFQDVSVTKRWLPVEKARNVYRNIHAALSDMPLRVMVELLSTYEEHDAKEATQDAIKCISFAMGDPNTYLMDHLIPLKPIKALEGQPIHELLKIFVYGKLSEYREFYRKHKDVV-DQLQLDHEKNVEKMRYLTFMYLAEKNQEIPFDDIRREVEI--DDVEAFTINVLGTKMVTAKVNHLTQKVIVVSTMHRSFMRNEWEQLRDILRLEKVEQSTQQFLDYLG-TR-RKKEVEDLAEEANAIDEGEERAVITESPQFEELINILIEWINDELAKHRII--VKNVEDDLYDGQILHKLLEQLTNSRIDVVEMTQNEEGQREKLKVVLERASQALGLKWSVNAIHSKNVVAIVHLLVALARHFRAPVRARLPENVVVNVVSVTKR-EQLTRTYDEYGMKVE-RDAFDQLFDHAPDKLTIVKKSLLTFVNKHLSKIYVDDLD-REFHDGVKLAFLMGLLEGYFIPLYLILKP----------------------DNKVNNVTFAFGLMKDAGLKPKARPEDIVNYDLKSTLRVLYNIFMQFSAGPSAIPVPVLRQAQQQMLNYGCSVMELSHRGAKIIKDAEKDLRELLSIPLSYKVLFMQGGGTGQFAAVPLNLCPADYLVTGAWSQKAAKEAGQYVKYTKIPPASEWKLSPDSAYFYYCDNETIHGVEF-LVCDMSSNILTRPVDVSKFGVIFAGAQKNLGPAGVTIAIVREDLLSSASVCPSVLSYKTTAENDSLYNTPPTYAIYLLLVLKWIKSEGGVAEMARSEKKSQGLYDLIEQSRGFYFRSRITIPFRI----RCDDALEKKFVKEAEMIQLKGHRSVGGIRASMFNAMDVSQAARLYRDRPEWQDVKPVLKISYTEAFKDCFGYLCAVISEELSERVFELTTTCADENPACYTVWVLRRKLLEHLKKDLHEEMDFMSRQIFDNQKNYQVWYHRQRLVQLLELEFIDKILDAKNYHAWQYRQWLLKTFNLWDDELNFCTAMLKEDIRNNSVWNQRYFVLKNTTNFVVEDELKFTLDRISCNESAWNYLGGMLNIYLYATLLSEAQEIDIPRSSYWTFRQRMLRQLLAAGLLLSLACTICLVFWLAVVIDRFEELLEFVPHMSRFLTAQGVAHKFFLINQGDRLRFNRGALINIGYLASRAQCDYMVMHDIDLLPLNSKLSYRYPDKDNVHLAAPHLHPKYHYSTFVGGILLMRHEVFAQLNGLSNKYWGWGLEDDEFYIRAKEAKIQLERPHNIGSGVNDTFRHIHDARRRPRDMVRIGAQREESRKRDRITGLHNVVFQLQGVYRMQIAGVPVEVHNVQLHCDLNATPWCEKPRVSPGTTITTDGYMRGHGGLLSAVAGVVEKVNKLITVRPLKTRYNPEVGDVVVGRIVQVC--QKQWKVDVGGRLNAALHLHSVNLPGGELRRKSIEDELMMSQYLIDGDLVSAEVQNVGVDGGVSLHTRNLKYGKLGQGALVRVSPSLVKRCKHFHNLP-NGVHLIIGLNGFIWVTSSKFAREAICRTRNVILCLAKHNIMLYDTPIVYAYDIS-VAQLTRHRADKLVGGWLITCSGMVFGAVLLGGITRLTKSGLSMVDWHPLNEGRPRTAEWEAEFAKYQQFPEFKIRNKDMTMEQFKSIYWMEYIHRMWGRSIGAVFYIPAAVFWMIGYLRKGMKQRIVYCGALLAAQGLMGWYMVRSGLEEKPRVSNLRLAAHLGTAFVLYSLLLRTGFDHILPNESLVRFSRAMGLVFCTALSGALVAGIEAGLVYNSFPKMADRWIPSDILALPKWKNFFENATTVQFDHRLLGEMVLTALYIHSRKVPLPPRARLATHTMLAAWLQVGLGITTLLTYVPTPIAVSHQAGALTLLTTLLWVTHELKLIQR-LSR------------------------TEKNACQWSKDKLNSLFADLEIND-SIMCVVKELKKCEGEATANNRKAKLIFFYEWDLELCAGS-VVKGRVEIPNLSDENDIDEVTVNVILVDEKIKGMMRSKGIEVIRSKLEEYVSSLKTDFSQGLILPTKDSGVNTTATKGKTISTKQLTMEEFKCTADELYRAFTMVQAFTRGPAGGRFQMLDTNVEGKFVRLNELEFEWRFKSWPAEHYSLVLSIEQVSVIVLRF---------QSIKGFH----------FRFRAMLMDPETFLEIANHVSKLKMYPYFEIAHCVVTLLYLREDLGSGSHLFSRKHPLSCWVSSMFSIYAGGIFAAFLLGEPVIAVFKSNQSLLLATACWYLIFYSPFDVVYKFCKILPIKLVIALAKEVTRAKKVHDGVHHAAKLYPNGYIIMIIIGVVKGNGSSFLKMFERLLRGFWTPQAMELMQPSFATKACAIAALVFVVDKKTDLISAPHSLVYFGVVCFFLYFKLSSVVLGLHDPSIPFENIACAMFFGGIWDAITKAI-----TKGGDAAGTGPKENKDAATKKKDMFRPDGIVQRRTAAVIARDRDDADRKEGDGCDNDKETLLTLME----------EGYTSFWNDCISSGLRGCILVELGIRGRIDLEAAGMRRKSLLMRKVIVKNDAPVGDVILDEALKHIKETATPETLQNWVDYLSGETWNPLKLRYQLRNVRERLAKGLVEKGILTTEKQNFLLFDMTTHPLVDQNVKDKLIKRVQDSVLAKWVNDVHRMERRHLALLLLSHASDVLENAFNPLSDEEYEMAMRRVRDLLDMDFEAECAKSQTCDIMWGVFAAFVKMADGVPTFKCVLVGDGGSGKTTFVKRHLTGEFEKKYVATLGVEVHPIVFHTSRGTIRFNVWDTAGQEKFGGLRDGYYIQAHCAIMMFDVTSRITYKNVPNWHRDLVRVCEGIPIVLVGNKVDVKDRKVKAKSIVFHRKKNLQYYDISAKSNYNFEKPFLWLARKLIGDTNLEFVAMPALAPPEVQMDPEWQAKLEDDMKQAQNTVLPDEEDDDLLAAQITPILKESKFRESGMLTPEEFVAAGDHLVATCPTWAWAKG-DKSYLPEDKQFLVTKNVPCYKRCRDMEEKIIEDD-EGWVDTHEKEDDDEEGVDMDAFLDEDSEAAGILSTRTYDLNISYDNYYRTPRLWLTGYDEAFRPLTTEELYEDISQDFAKKTVTVENHPHIEGLPQASVHPCRHAQAMKNLIQTVEEGGGLQVHMYLIVFLKFVQAVIPTIEYDYTANFNMMSVLEKKLPYDKLSDNINIVKKRLNRPLTLSEKILYSHLDQPESEEIVRGQSYLKLRPDRVAMQDATAQMAMLQFISSGLPKVAVPSTIHCDHLIEAQVGGDKDLSRAKDINKEVYNFLATAGAKYGVGFWKPGSGIIHQIVLENYAFPGLLMIGTDSHTPNGGGLGGLCIGVGGADAVDVMANIPWELKCPKVIGVHLTGKMSGWTSSKDVITKLAGILTVKGGTGAIVEYFGPGVQNISCTGMGTICNMGAEIGATTSVFPFNSRMADYLASTKRRDIADAAEKVKDLLSADSGCKYDQIIEINLDTLEPHVNGPFTPDAAHPVSKLGQTAKEKGWPLDVKVGLIGSCTNSSYEDMSRSAMLAKQALDHGLKAKSLFTVTPGSEQVRATIERDGQAKVLKEFGGMVLANACGPCIGQWDRKDIKKGDKNTIVTSYNRNFTSRNDANPQTHAFVTSPEMVTALAIAGRLDFNPLTDDLVAADGKKFKLKPPVGDELPRSGFDPGEDTFQAPPSDGSNVKVDVDPKSQRLQLLAPFDKWNGKDLTDMVVLLKAKGKCTTDHISAAGPWLKFRGHLDNISNNMFIGAIPEESGEANKVQNRLSGKWGGVPETARDYKAKGQPWVVIGDENYGEGSSREHAALEPRHLGGRAIIVRSFARIHETNLKKQGLLPLTFADPADYDKIKSDDQISIVGLKDFAPGMESRGGMFKNTFQSGFLSILYSLGSKPLQIWDKKVRNGHIKRITDNDIQSLVLEIAGSNVSTAFITCPADPKETLGIRLPYIILIVKNLKKYFTFEVQILDDKNVKRRFRASNFQSTTRVKPFICTMPMRLDDGWNQIQFNLADFTRRAYGTNYVQTLRVQIHANCRLRRVYFADRLYAEDELPAEFKLYLPVQHRTAAQTLRER---------GLSASHRYQYMLKARNDIKRVIGKYRALAETFIFNDGSSKELVCLDGTIPVRYKYNIPIRVWVLDTHPYHAPLCYVCPTPTMQIKVSQYVDESGRVYLPYLHEWNSNL-SDLTGVIGVMVVVFGETPPVFSKGPSPYPTSNTLSITDEDIRISLLSAVESRITERALEKSKAEEDVLRKTNEDLQAGKQKLTRYMADMERDREMESEKMDQLKQLDVDSAVTTTAPLFRQLLQAYAEESAVEDAIYYLGEGLRKDVIDLDTFLKHVREQSRKQFMLRALMQKCRQKAGLPMGRKFYVGGNWKMNGTMETIQEICCRLKNSEPKTEVCVGVPAPYLQFVRNIVSSKIHIVAQNCYKVPSGAFTGELSVEMIKDCCSDGVLLGHSERRNVFGEKDELIAEKCALVLDNKLEVIACIGELLEEREAGKTEEVVFRQLKAYADKIKDWARVVIAYEPVWAIGTGKTATPDQAQEIHAKLRDWLSKNVSEEVSLNTRIIYGGSVTAANCKELAQKPDIDGFLVGGASLKPEFIDIINAKKMSSTVSKKRRFVADGVFNAELNEFLRRELAENGYSGVEVRNGATKTDIIIMATRTQDVLGEKGRKIRELTAVVQKRFNFKEGTVNLFAEKVSARGLCAITQCESLRFKLVGGLAVRRACYSVLRCIMEAEAMGCEVVVSGKLRGQRAKSMKFVEGLMIHSGDPTNHYVETAVRHVLLKQGVLGIKVKIMHPYDLQGKRGPALMLPDKVIVTVPKDEDDNVGINSDNKENAI--------GVAEPTTL----SKIEQIKQWSLSTYKCTRQILAEKMGKGTRTVDGELEANIELLRETHQKYLNILRLAKLLTTHFNTVATQAALGECFSDLAQK-------ELQQEFLYNAETQKNLSKNGETLLGALNFFVSSLSTLCNKTIEDTLITIRHYENARLEFDAYRCEARANFKEKYERLRGDVQIKMKFLHENKVKVMHKQLLLLHNAVSAY-FSGNQSSLEATLKQFNISWLEQ-MVDLVLDRDIRIWVFLPIVVIMFFVGIVRHYVSILLTSSRKAELQQVYDSQALIRVRYLRENGKYLPARGFFMRKHFFNDEESGWLK-TQKRPPPINNPMSDPGMMSEMLKGNLTNVLPMIVIGGWINWTFSGFLTTKVPFPLTLRFKPMLQRGIELASLDASWVSSASWYFLNVFGLRSIYALVLGEDNAADSTRAMQDSMVPQAAAMPQDSKAAFKAEWEALEVVDHKWALAGIEDDLCSKPLQF---------MLEQPGAASEAGRAMQILKRRERQKEEVELKRQKIEQEMRVS-MGDKFSSHFDAVEAQIKSATVGLVTLDEMKAKQEDAVKEREKRLAQKELEEKRRAEDKKRAQKEKQKKAIQALSFNFDDEVDE-DEENQEGNDSDETG-----NDSAQEKEDNRKDGFDGVDDDDASSDSKEDKPKIRVKKNPDVDTSFLPDREREEAERMIREELRQEWKDKQKALKEENIQITFSYWDGSGHRRVVDMKKGNSIYQFLQRCLDALRKEFYELRVVSADQLMYIKEDLIIPHHYTFYDFIVTKARGKSGPLFAFDASEDIRMTSDASKEKEESHAGKVLLRSWYERNKHIFPASRWEPYDPTKCYDKYTIKDKKKNPPAATKKPAKSAVPETLLKQRKHNAELRQQRLLAAASKKKADRARRVLAFRRAEQYVREYRRIEASEKNNRLVAKVNGNFFVPDEPKVAIVIRIRGITGVSPKPKKVMQLFRLRQINNAMFVRLNKATINMLRIAEPYLAWGYPNLKTVRDLIYKRGFGRVNGRRAPLTDNAIIEEKLGRYGIICMEDLVHEIYTLGPNFKQVVNFLWHFKLNNPKGGWRKKTTHFVEGGDYGNRETLINSLLRKMIVKVDAKDLWKSVTSVSNAGRKRGRASGNSKKTSKDLNKGQKIGEGTVNMVWPGLNAPIVRGREIVKQGSLPPDLERTKRILALRDKQPNFRRLKLNPMERGWSGTKAQGRSLGPPDPIGGSEFPDFNSVILSLRLVSHMTGHLGRVRRHKAIVAVGNGKGLLGFADAKASVAKSALRRAKNKAFQRLCFYERFKDTVLHDFITEYGCTRIVVTKKERGFGLICHRALRDLCKLIGIKDMYAKVDTRNTNMLHLIRAFLLGLQKQKSHQDIAEEKRLHVVEFRPERSFFPEIIASPKNVRTSDEIGSYEELDINRYISGGKMIIQKPKKMPFYTRLPGWEIHLRKTDNLKNERMVRLRIFRKYGELKSFLNVRQPLMQKGARSSEGKYKWTNKQRLLIFASRGITYRDRHLMNSLRSLLAHSKEEVKFE--KKDINEIAEMKNCNKVMYMENRRKSDTYMWLANMRTGPTLKFLIQNVSTMEELKFSGNCLRGSRPLLSFDPTFNNNPLVKEVLAQTFGTPAYHPRSQPFFDHVFVFRLLDKRTWFRNYQIVEE-DGSLVEIGPRFCLNLIKIFDGPFSDVVIYTNPHYVAPNKIRRLAKKD--SRYVHLDVFETKHAYYRMAVGKNKGLSKGGKKGLKKKIVDPFTRKDWYDVKAPSMFAVRNVGKTLVNRTQGTRIASDGLKGRVYEVSQADLQTGEDAFRKFKLVCEEVQGRHCLTNFHGMDLTTDKLRSMVKKWQTLIEAQVDVRTTDGFVLRLFCIGFTKKAQNQVKKTCYAQHAQVRAIRRKMVETMQREVASCDLKEVVNKLIPEAIGKDIEKCCNTIYPLHDVHIRKVKVLKKPKFDMGKLLEMHGEGKGAGGGDGMAVDRPDNYEPPVLEDVAKIGIIGGSGLE-RPDLLKQKTPYGEPSDA-LITGKINEIDVVILSRHGRRHTINPSNVNYRANLFALK-LEGCTHILVTTACGSLKEEVRPGNFTPDSFIDRTRTQTFYVCHLPMTKPFLSDLLSKHPKGVVVCIEGPRFSTRAESAVFRQWGADLVNMTIVPEVVLAQELGIPYAALAIATDYDCWS-N--DTVDVQKVATLKKAADEACHILKTVLPKILNIAVL--------------------------MRVLRHEEFETGCQAACNGRYDGFWSKTMVGYGSEDDHFVMELTYNYGVNSYKMGNDFRGILIQSDEILKRAALHKCPVELEKGRTYLKSPGGYKFYVDNLKS-E-GDPVKQVIYNCTNLAKTRRFWEDILWCEVLDSGDDFLEVTYDKSKTSLRFEKIIEPIDRAEAYGRVAFSCPRGQLEDIESQVKNSNGVVITPLVTLPTPNKPPVEVVILGDPDGHEICFVGDEAFRELSKEDPKSERMLEEKMA--EYDAFIKKTAATGRRFRLPPLPTPADLLRFYRLRALKQLSQNFLLDPICAKVVRSSGHVIEVGPGPGCLTRPILEQGAESVVVIEKDRRFLPTLKLLADATN-NRVKVVHGDVLLQELHNLI-PPEKAEPWEGIHLIGNLPFSISTILIVRWMKMVSERTGAWKFGRTRMTLTFQKEVADRITCPERCRLSVLVQGCYTKSGGSFVPPPMVNVGVVRVVPIEPLFQPFDIVEKVLRCLFNKEIRNDLVPEILKMANVDPTLFLDIEEFSRICDVYAKLCDEPGFSGFDYRGNCNITTKRVVTYVRSSIVLRLHTSRSLNLPLIPIVIEQTGRGERAYDIYSRLLKERIICLMGPINDEIASVVVAQLLFLQSESPKKPIHMYINSPGGSVTAGLGIYDTMQYVLPPVATWCVGQACSAASLLLTAGEPGMRHSLPNSRIMVHQPSGGVSGQATDIQIHAEEILYLKRKVNLLYAKHTNQSIETIDAAMERDRFLSPEQSKEFGLIDTVLEQPP-VRSS---VRVERPVGTWLTLLPLWALTSAAPAATLPDAVLFCTGAFLMRGFGCTVNDMWDRDIDRQVERTRYRPLAASKLSRWDAFWFAGGQGLAALVLLQLNWHTVCLGLASLGIVVVYPLMKRFTYWPQAVLAIVFNWGVLLGFSAAALPMYLAAFSWTVIYDTIYAHQDKKDDLVVGIKSTALRFGTRTPIWLSAFTAMTTGLMGWPYFIAVTLKIDNIGLLVGCIGGTLLKIARVDRPKTRKGSKIVKAREPQLIENPKTSFIRAGNINQRTTQILKDLYTLKKTESVFYQKKNPFDDAVPLEKLAKNDTSLFALGSHNKKRPQNIVLGRTFDRMVMDQFEFGVENYKSLEEFKVPKISIMVKPILVFAGESWQELKRLKNFLIDFFKGDYIGVSGLEHVISFTALDILLRSYKVQLKKSGQTTPR-VEIYEMGPRMDLKLRRNKIASDDLFKQALRRPKELKAKKKKNLETDDLGTSFGRIHMERQDFKLQTRKMKGLKVRILEAFSDNYMYLVITKEAAVVDPAEPQKVISAVEVKLTTVLTTHHHSDHAGGNEQLLVYGGDARIKKLTHRDDMKITIGSFVQTLLTPCHTSGHVCYFIVFTGDTLFTSGCGKFFEGTAQQMQAAMLAALPPETRVYNGHEYTVSNLKFALHVEPGNKVSDKLSWAKFQPTIPSTIGEELTYNPFMRTDVQAHTDPVSTMAALRLEKDNFKMNRLFGKAKAKEPSPSLTDCISTVDQRANNVEEKIKKLDIELVKYKEQMSKMREGPAKNMVKQKAMRILKQKKMYETQRDNLMQQSFNMEQANFATQQLKDTKITVEAMKLGVREMRHEYKKINLNEIEDLQDDLEDMLEQANDVQETLGRPYGMPEIDDDELEAELAVLNDEIALEDTSYLDDVTTPKVPSKDPSEKEKTEGGIMVDDSGRHECTHPVRSLSRSRGKATLPDLPYDYNALEPVISAEIMQLHHSKHHNAYVTNYNVAAAKLQEAVQKGDITAQVALQSALRFNGGGHVNHSIFWQNLCNPKTGEPSADLLAAIKRDFSSLEAMKEKVSASAVGVQGSGWAWLGYNKTTKRLQVTTCPNQDPLEATQGLIPLFGIDVWEHAYYLQYKNIRPDYVKAIWKVANWKDISQRFATAQDDHIQHDVIKEALESGRDLREYSRSVEQQLKGSEDEAIRDYMHNCRDIAALHGEILSCDAILQHMESILRGFQNDLGSISSEIQSLQRQSVAMNLQLKNRQAVRGELSQFVDDFIVPEATINVILDCPVTDEDFLIQLALLDQKISFVKVQSFKEASSCQDVKDILDKLKVRAVTKIREWLLQKVFSFRRANANFQLAQNTMLKHKRFFQFLATHEREVAKEIREEYVDTISKVYFSYFKAYQGRLMKLQFEDVPDRDDLMGVDDTPRWGLFNKPSLKNRSTIFTLGNRNAVLSSELEAPVIVPHASAKSEKHYPFEQLFRSMQYAFCDNAAREYLFVSEFFLLTKQGAADMFDNILEKSMTLFAKHTESFVADCFDSIALFLCIHIVHKLKVIMHVRNVPAMDRYWDLLAKIIFPRFETILRINITSIRDCDPSKLGSIDNRPHYITRRYAEFSAAIVSINENHPEERVSQLLGQLQIEVENFILRMAAEFNGRKDQLIFLINNYDMMLGVLQQRTHEDSKETTNFRGLLTARQSEYVEQILTVHFGGMITFIKESEYYLEKGQTEKLEKESQQVATLVRTFNSGWKKAIDNMNADIMKTFTNFKCGTSILQEALKQLLQYYHRFNKVLSQPPLCSLSVRSELINIHHLMVDIKKYKATFMAPAVPILWLFASKVAD---GYFIVVDAHSEECFHDRVVKGTKLGLTFEVAEGGFLDIDVKITGPDDKVVYNGERESSNKYTFAAYMDGQYKYCFSNAMSTMTPKTVMFTMDIGEEPKDDAK--GDGSENKLEDMISELHTAMTGVKHEQEYMMIRDRIHRSISESTNSRVVIWAFFENLVIIAMTLGQVYYLKRIFEVRRVVMRLSLILKLPKDYANLPESYVKRTMEQVEWRTPKGRQYRKAVIQKPYTMNRPWTDEFRNNRPGVKISVKAMFRGDRVEIMVGDDKGKLGTVNYIVQERNWVTVEGLNTRELPLIMGSQVKLVDPLDEKATDVQWRYTGSGQKVRISLRTGRIIPIPLAHETIDYKYIDRSNDTTSAELTKITFIP-KLATFEMDIMEQQGIEDNRVPQRTFWYMADNSA--MIVDLVEQKMELLLWLSRLACAIFTFLYLLGNPFSLYQKALICNGVTSALRLHQRSPTVRLTAEFLGSVLIEDSCHYLIYSMIF-LPIS-MVLLPPMLFALLHSLQLLSRAGILHQ---SRVKAFRLIAMTEIFLFPTVVLGLFWGVSLMTPFMYYRFLKLRASSHRNPYTRNVFYELKLHATQFAVPNCPSIVSRVRFIESRADIAVVIIAVYLSFPEMRPEERPYVKLPTSLEDAKNLGRVLSNYTDKTVLLAFFCTYIFLQSFAIPGSLFLSFLSGFLFPFLLAITLVCLCSAIGASLCYLISYNVGRRLVLHYIPNMIYYIIFLRITPFLPNWFINITSPIVDVAIFPFFVGTFIGVAPPSILAIRAGTSLQQLASAFTWENILLLTGFAALSLVPVLFKLKSKFEEVQDKLTRIAIVSTDKCKPKRCRQECKKSCPVVRMGKLCIEVTPNDKIATISENLCIGCGICIKKCPFEAIQIINLPSNLERDTTHRYSVNSFKLHRLPTPRPGEVLGLVGTNGIGKSTALKILAGKLKPNLGRYNDAPDWTEILAYFRGSELQNYFTRILEDHLKAIIKPQYVDQIPKAVKGTVRELLDKKNDLGKEADLCELLELVNVMDRQIVDLSGGELQRFATAMVCIQKGDIFMFDEPSSYLDVKQRLKSAEAIRGQIEATKYVIVVEHDLSVLDYLSDFICCLYGTPGCYGVVTMPFSVREGINIFLDGFVPTENLRFRESSLVFKVSDNTDEDVKRIARYEYPSIMKKMGDFELQVRGGSFTDSEIIVMLGENGTGKTTLIRMLAGRLKPDGDEEVPTLNISYKPQKISPKSTGTVRYLLHEKIRDAYQHPQFVADVMKPLLIDNIIDQEVQNLSGGELQRVALALCLGKPADVYLIDEPSAYLDSEQRLAAAKVIKRFILHAKKTGFVVEHDFIMATYLADRVIVFDGKPSVSTVANEPQSLLVGMNRFLELLDITFRRDPNNYRPRINKLNSVKDSEQKRSGTFFFLEDEEDGEDLFGPELDRYDAAEEEMRRRDRGRLPRGLESIENLEDMKGHTVKEWVTQGPKTEIFNRFKNFLRTYKEKIRAMEQNRMSLEVDYTLAQSEQVLAFFLPEAPAEVLPIFDEAAKDIVVGMFPHYGRIHHEIRVRITDLPILEEIRTLRKIHIDQLIRTSGVVTSTTGVLPQLRMVKYDCAKCKYILGPFVQSQELKPSSCPECQSTGPFTINVAQTIFQDYQRITIQESPGKVNAGRLPRSKDAILLHGLCDSCKPGDEIEITGIYSNKFEGSLNKANGFPVFGTVIIANHILKKDTDEDVKEVVKLSKEELAERIMASIGPSIYGHDDIKRAIALSLFGGVSKNPGQKHRIRGDINVLLCGDPGTAKSQFLKYVQQIAPRAVYTTGQGATAVGLTAYVSPVTRDWTLEAGALVLADKGVCLIDEFDKMNDADRTSIHEAMEQQTISIAKAGIVTSLRARCTIIAAANPIGGRYDTFHQNVNLSDPILSRFDVLCVVRDERLARFVVDSHCRHHPINQDLLQKYILYAREKIEPKLDQDKIAQLYSNLRRESMATGSMPITIRHLESIIRLSESHARMHLREHVDVNMAIRVMLDSFVTQKFSVMRMGLLDDAKETFGANTLYDVFGVEKTVAADRLKKAYRKKSLLCHPDKAKQGHKEEFTKKFQILCKCFEILQDNNKRKLYDETGSV--DDSFNADKDWEAYWRCLFPKITVNQIQEFIDKYHGSEEEREDLKRSYEKSKGDMNKIAEYLIGYNEDRLCKLLYEMIDAGEITEYRAFTKESAASKRKRQKKYAREAKEAEQ------KSMNELALMIHQNDRKEGEDFLAALEAKYATSKQKKTSGSKKKTVLLLAATCALLLGKIGTKKLRKLEEKAERRRLRELELQEREERQQKQAEDERRRKEEQKKEDEEKRKEEEQRKQEEERERHEHEEYLKMKAAFAVEEEGYDEKDDL-NSSQKLSEFIAYVNDQKVVQLEDLAARFRLKTQDCIDRIQRLLEDGSLCGVIDDRGKFISITREELEEIARFIKIRGRVSIQELVENSNRLINLASIIMKQQEEAKALGLKHVLSQYQLPRESQFVEQHRKRVQRKKASVEAMLKTAVQSQLEGVRTGLLLLSAARDDVNDIQKDEADTIYGLSKLVQLQDVREESFRHSQTGTLMEHLKHIFNVPGSVARTQDLIQDGRLLLAHKLSDLECSRDELLYELHKQANNQASDRAMLKQYFADVERLSDELAKQLWLILKRTLNTVRREPQVIVTALRLIMREEWAALRRQEST-GFLPPGRPKLWRKKAMETLEQSVAERLEANQIEIRSENKMWLVRHLEVTRQLIIEDLKTVKHHCTPCFPSILDIFNEVRMTHNCLSQRLQTIIAGLVDSEYIHVLQWLNAYQSRELMGHPDLHVDMALLPDTIERLMERYLAGLNTKFEEWLRNALSDHKDWPEQDSHGYYRTEAPMLIYQMITQHVEVARTVTLVSRVLTLAMEQMNKFLSDYNQLVTDYNFEDRSYTAYMIAIANNAVNMK-LRELKENSLDYLCEEVLTDIKVMPDIMTETVTVTLADYGNDYIEKEVASNYVKAIC--EKRMSFRNYEERRSAAELEKLKNSKLDILKLMAEVLKMKDSTLLSLEMNRLLFLLVFCLAW------DVRKADAPTKVEDDIGKIQEASRTDAEVVAREEEAIKLDSLSVAQLKEIREKAQKHAFQAEVARMMKLIINSLYRNKEVFLRELISNASDALDKIRLLSLTNPDALKALQDLSIRIMADKENNVLHITDTGIGMTQEDLMKNLGTIAKSGTAEFLQKVSEGGGGTDLNDLIGQFGVGFYSAFLVADRVAVASKHNDDPNQHVWESNASEFSIADDPRGDTLKRGTTVSLYMKDEAKDFLEHDTLKKLIEKYSQFINFNIYLWSSKT-VTEEVIEGEKPSEETAT-LDDDEEAKVEDAKEA--PKTKKVEKTVWDWELINSAKPIWTRKEKEVTDEEYNEFYKTVTRDNQDPLTRTHFTAEGELTFKSLLFVPQKQPQDSFNKYGQKTDHIKLYVRRVFITDDFQDMLPNYLSFLRGVVDSDDLPLNVSRENLQQHKLLKVIKKKLVRKALEMFRKIPETEYTKFWKEYSTNIKLGIIEDSANRSRLAKLLRFPSSLDSLDKLVSLSEYVQRMKDKQSAIYYIAGMGLDDVKKSPFVERLIKREYEVLYLTEPVDEYAISSLTEFEGKKFQNVAKEGLSLDD---NKEIREALEKEFEPLTKWMTETALKDKISKAVISERLVETPMALVASQFGWTGNMERIVAAQTHMKENDPQRMFYMGQKKTLEINPRHPLIKELLRRVDDSPSDETAKYLTEMMFETATLRSGFQLNDNVRFASNVERMLRKMMGVSEDAQVDAEPEEAEMHKETLADQEEDIESEPHDELGRRGEVKELSPQELKMVVISEIIHELVRAHNDKRDVNLNRVKCDTSSRYGLKSQPKLVDIIAAIPPQFKQILLPKLKAKPVRTASGIAVVAVMCKPHRCPHINYTGNICIYCPGGPDSDFEYSTQSYTGYEPTSMRAIRARYDPFLQTRHRVEQLQQLGHDVDKVEFIVMGGTFMSLPDDYRDFFIRNLHDALSGHTSASVDEAVKYSERSKTKCIGITIETRPDYCLKRHLSDMLRYGCTRLEIGVQSVYEDVARDTNRGHTVRAVCETFHMAKDCGFKVVTHMMPDLPNVGFERDVLQFVELFKNPDFRMDGLKIYPTLVIRGTGLYELWKTGRYKSYPPALLVDLIAKILSLVPPWVRIYRVQRDIPMPLVSSGVENGNLRELALARMKDLGLVCRDVRTREVGIQEIHNKVVPYQIELVRRDYVANGGWETFLAYEDPQQDILVGLLRLRKCTDQTYRPELVGQCSIVREHVYGSVVPVHSRDPSKFQHQGFGTLLMEEAERISRDEHGSAKIAVISGVGTRNYYRKLGYELDGPYMSK

>Osperalycus_tenerphagus

FSEKMNSMRFYRAHMLIVLSAIGDCF---ALPDLLHRVRDSVRRRRPAVNTLTGASFGGILN-TFSVHRRNLASNEHRSGFKLPTAKQGQCPNGDRQRRKIKIINLIARSDTRLQEMLVRPD-------LDLTVKNILMHVILQEVNGRGCYHGKLSGRTRSGSRLSLRNF-IFNKAREQVMKMTKGLYPAPLKILDVVKTGLEKRGFAELSTLHSKALIGLYHGQVLCKKNRFGKPTKTIAVLGAGLMGAGICQVSIDKGLDTIMKDGLARGETQIKKRRLDRDLYMSRLYSQTDYSDMVIEAVFEDLNIKHKVIQEVEQDDCIFASNTSALPISKIAEASKRPDKVIGMHYFSPVDKMMLLEVITTDKTSPETAAAAIQVGLKQGKVVITVKDGPGFYTTRILAPVLSEAIRLLQEGVKPKELDNLRSFGFPVGAATLIDEVGIDVASHVAEKVFVERKEMVAGFLGRKSGKGFYVYRNMNDILKRYTQEELQMRLAVRFVNEAVLCLQEGILANPLEGDIGAVFGLGFPPFLGGPFRYLDNHGADKIVAMHKFAEFKPCQLLLDHA-KDTSKKFHMQAPIILLKEGTENTQGRNQVVSNINACQAIAEAVRTTLGPRGMDKLIVDSKGSVTISNDGATILKQLEVVHPAAKTLVDIARSQDAEVGDGTTTVAILAAEFLKQCKPFVEEGLHPQIIIKSFRKGCQLAISKINEIAVTI-QDKRRAILEKCAATTLSSKMIAQQKEYFAKMVVDAVMLLDELLPLDMIGIKKVQGGALEESKLIAGVAFKKTFSYAGFEMQPKKYQNLKIALLNVELELKAERDNAEVRVDSVKEYQNIVDAEWNILYDKLKKIHESGAKVVLSKLPIGDVATQYFADRDMFCAGRVPEDDLKRTMRACGGSILTTVYDLKDENLGTCELFEEMQMGGERYNLFTGCPNTKTVTIILRGGADQFIEETERSLHDAIMIVRRALKNDAVVAGGGAIEMELSKYLRDYSRTIAGKEQLLIGVMAKAFEVIPRQLCDNAGFDATNVLNKLRQKHASGGVWYGVDIFNEDIADNFEHCVWEPAVVKSNAIISATEAACLILSVDETIKNPKSSNEMTAARAMVNEMNEGAGRNQHSLAVTRDYISQPRLTYKTVSGVNGPLVILDDVKFPKFAEIVNLVLSDGTKRTGQVLEVSGSKAVVQVFEGTSGIDAKHTVCEFTGDILRTPVSEDMLGRVFNGSGKPIDRGPPVLAEDYLDIMGQPINPESRTYPEEMIQTGISAIDVMNSIARGQKIPIFSAAGLPHNEIAAQICRQGGLVKAPGKSVIDENFAIVFAAMGVNMETARFFKQDFEENGSMENVCLFLNLANDPTIERIITPRLALTTAEFLAYQCNRHVLVILTDMSSYAEALREVSAAREEVPGRRGFPGYMYTDLATIYERAGRVEGRDGSITQIPILTMPNDDITHPIPDLTGYITEGQIYVDRQLHNRQIYPPINVLPSLSRLMKSAIGEGMTRKDHADVSNQLYACYAIGKDVQAMKAVVGEEALTSEDLLYLEFLGKFEKNFISQGSYENRTVFESLDIGWQLLRIFPKEMLKRIPQNLLAEFYPRGGQARTIASSAFNKLTGLFNDPNGFYLLRENAVIEAEHLVKEALSRQRKMVQIFDELSNALCKVADLAEFIRIGHPSERFQMCAEQASMAISAEVERLNTHRPLYDELRRVTLES-DNLPTTLDSHVAKLFLFDFEQSGIHLDSADRKVVVQLNEHILHVGSYFMNNTNQPRTVAKSKLPEEIAIVTGLFADFDNELLREAAYRIYLHPDDHQDQLLSQLLRSRLKLAKICGFESYAHRAVKHSIAENPEMIQFLDILNERIRPLADRDYKEMLMLRPWDVPYFTPYFSLGACMDGLNTIFGHLYGVRLQVAGETWHKDVVKLSVVDSSELLGYIYCDLFERATKPHQDCHFTIQGGCLGDGTYQVPIVVLMLSLPPSLLTPHMVDNLFHEMGHAMHSMLARTPYQHITGTRCSTDLAEVPSILMEFFASDPRVVSKFAKHYITGEPIPDEWLATWIQSKRVFTASDTQLQVFYAALDQAYHSEKHVSTTDVLAAIQNRYYGLPHIPNTAWQLRFAHLVGYGAKYYSYLVSRAVAAAIWKKLFAADPLSACAGRFKDEVLAHGGGKPARDIAEGVLIASSIVDDV????????????????????????????????????????????????????????????????????????????????????????????????????????????????????????????????????????????????????????????????????????????????????????????????????????????????????????????????????????????????????????????????????????????????????????????????????????????????????????????????????????????????????????????????????????????????????????????????????????????????????????????????????????????????????????????????????????????????????????????????????????????????????????????????????????????????????????????????????????????????????????????????????????????????????MVLADLGRKITSALRSLGNATIINKDVLDSLLKEICTALLEADVNIRLVKQLRENVRQVIDFDEMAAGLNKRRMIQMVVFKELVKLVDPGVKAWQPTKGRCNVVMFVGLQGSGKTTTCTKMAYYYVRKGWKCALVCADTFRAGAFDQLKQNATKARIPFYGSYTESDPVVIASEGVDKFMNEGFEIIIVDTSGRHKQESSLFEEMLAISTAVQPNLIIYVMDASIGQACEAQARAFKSKVDVGAVIVTKLDGHAKGGGALSAVAATQSPIIFIGTGEHIDDFEQFKVKPFVSKLLGMGDIEGLIDKVNELKLEENEELIEKLKHGEFTLRDMYEQFTNIMKMGPFNQLLSMIPGFGADLMKGASEAESMSRLKRLMTIMDSMSDSELDSREGAKLFTKQPPRIARVARGSGVTQKEVQELLTQYTKFSAVVKKMGGIKGLFKGGDMAKNVNPTQMAKLNQQMAKMMDPRLLQQMGGMSEVLGIYKKMHGFSMSLKILLFRLSRFKVK---------------QRRPSRSISMTMPRDGLLFDYSKNLVNDDVLKLLFDLARSRKVEEMRDKMFAGEKINFTEGRAVMHVALRNKSNAVTLVDGKDVMPDVNRVLDQMRRFSVALISGSWTGYTGKKITDVVNIGIGGSDLGPLMVAEALKAYQIGPNVHFVSNIDGTHLTTVLRRLNPETTLFIIASKTFTTQETITNAVTARDWFLKTAGGKSAVAKHFVALSTNTEKVKEFGIAESSIFEFWDWVGGRYSLWSAIGLSIACHIGFDNFRQLLDGAHWIDNHFKTTTLEKNIPVIMALLGIWYIDFFGAETLAILPYDQYMHRFAAYFQQGDMESNGKYVTRNGEPVNYSTGPIVWGEPGTNGQHAFYQLIHQGTKLIPCDFIAPVKTLNPVRDGVHHKILLANFLAQTEALMKGKTRGEAEAELKKKGMKDEEIAKILPHKVFEGNRPTNSIVIDKLRPFNLGALIALYEHKIFVQGVIWDINSYDQWGVELGKELAKAIEPELDSAETITSHDSSTNLLINHIRRVG-------------------------------------MDKMIQSANGDVTITNDGATILKQMQVLHPAAKMLVELSKAQDVDAGDGTTSVVVIAGSLLEAASKLLAKGIHPTTISDAFQNAAKTSTKILSSLAIPVHLTDIESLVRVAATSLNSKVVSQYSSLLAPIAVNAVLRVKDNDNVD-------XXXXXXXXXXSLIEGLLLPLRFANDFGAKKVEKAKIGLIQFCISPPKTDMDNQVIISDYTQMDRVLKEERNYILNIIKQIKKTGCNVLLIQKSILRDALNDLALHFLGKTKIAVIKDIEREDIEFICKSLGCRPIASLDHFTSENLVSADLVEEIPGAKFVKITGISAGSHNKTVNILVRGSNRMIIDEAERSLHDALCVIRCLVKNAALVPGGGAPEIELSLRLSELSRSMTGLEAVCYRAFAEALEVIPFTLAENAGLNPIGTVTELRQRHAAGEKNTGINVRKGSVTDILEENVVQPLLVTSSAITLAAETTRSILKIDDVINTVRMSPSNVVYFWDPEVGNFHYGPGHPMKPHRLAVTHSLVLNYGLYKKMNVYKPYRASFHDMCRFHSEDYIDFLKRVTPSNVANFTKHLSQFNVGDDCPVFEGLYDFCSRYCGASLQGAVQLNNHCCDIAINWSGGLHHAKKFEASGFCYVNDIVIAILELLKYHPRVLYIDIDIHHGDGVQEAFYLTDRVMTVSFHKFGNYFFPGTGDMYETGSEAGRYYSVNVPLKEGIDDHTYSTVFKPVVSDVIQFYQPTAIVLQCGADSLAADRLGCFNLSIKGHGECVKFVKNLNLPLLVLGGGGYTLRNVSRCWTYETSLLLEEQISNEIPYSEYFEYFAPDFSLLLDKPCGETTHNANSRVYLDSIVKYVHENLKCVAHSPSVQMHQTPSDWLDLSENPIHLLSERESENEFYDREEGHRNS-------MCADEEDYDLEYSEDSNSEPDVDLENQYYNSKSLKADTPLVALASFQKVLDLE-GDKGDWGFKALKQMIKINFKLGNYPEMMARYKQLLTYIKNAVTRNYSEKSINSILDYISTSKQMELLQEFYETTLEALKDAKNDRLWFKTNTKLGKLYFDRGEFNKLAKILKQLHQSCQNDDGSDDLKKGTQLLEIYALEIQMYTAQKNNKKLKKLYEQSLHIKSAIPHPLIMGVIRECGGKMHLREGEYEKAHTDFFEAFKNYDESGSPRRTTCLKYLVLANMLMKSGINPFDSQEAKPYKNDPEILAMTNLVSAYQNNDINEFEKILKNNRRTIMDDPFIKEHIEDLLRNIRTQVLIKLIKPYTRIHIPFISKELNIDVSEVENLLVSCILDNTIQGRIDQVNSVLELNKGSQSAARYNALDKWTNQLSSLHQSIVNKMIRQMSLNPVQILKGDADEEKGELARLSSFVGAIAVGDLVKSTLGPKGMDKILLCQGRGEGKVEVTNDGATILRSIGVDNAAAKVLVDISKTQDDEVGDGTTSVAVLAAELLKEAEQLIAMKLHPQTIIAGWRKATQVARDALEAVAIDVSDDEEVFRKHLINISKTTLGSKILSRHNDFFSKLAVDAVLRLKGSGNLDAIQVIKKLGGTLEDSFLDAGFLLDKAPGHGQPRRVEKARILIANTPMDTDKIKVFGSRVRVDSVAKVAEMELAEKEKMRDKVNLILKHNINVFLNRQLIYNYPEQLFADAGVMAVEHADFDGIERLALVTGAEIVSTFGNPDKVKLGTCDLIEEVMIGEDRLLKFSGVPLGEACTVVLRGATQQILDEAERSLHDALCVLSQTVKEPRIIYGGGAAEMLMANAVGDLAAKTPGKESFAMESFAKALRQLPTIIADNGGHDSAQLISELRAAHAKGQHSYGLNMDVGLVDDMAKLNVIEAYVVKRQVLISAAEAAEMILRVDNIIKAAPRKRNPDHLFMAAEIRPPGRLQLVSKLEGHQDVVNMAIILRDEEGLISISDDKTVRIWLKRDVGSYWPSVCHIMPSAATAMDFNHETRSLFVGMENGSISQFSVSDDFNRITHDRNYLAHQARVTAVIFSLVTEWVLSVGRDKYFQWHCSETGRRLGGFGCSSWCTALQFDVQSKHVFIGDYSGQVTMLKIEETGYKPVTTLKGHSGCIRRLEWDADRSMLFSASSDQVIICWDIGGKRGTAYELQGHHNKVTSLCYAAASKLLSGGEDCAIIAWDMKAKRKETPEWAESDFCQRCSRPFFWNIKAMVDQKIIGLRQHHCRKCGKAVCDACSTNRTTIPVIGYEFKVRVCDECHSVITDADRVSLATFHESKHSIAHMDLDESRCQLLTAGADRVIKIWDVTRLLM---GGEGSVSKKAQKKAAKQVEKAAKKAEKDAKQASSLVNSNQIMMDNRYGKMAMVQSSQKTDRVLVSVMSLTRDIVDKKVWLRGRLHTSRSKGKQTFFVVRQQQFSVQCLVSVSDIVSKAMVKFAATITKESIIDVEGVVKSVPMKIESCSQQDVEIHVEQLFVVSNSEPKLPLQIEDAARPETEGDEEGGLAIR--------------------AIFRLEAGVCKLFRETLEKRGFVEIHTPKIISAASEGGANVFEVSYFKGSAYLAQSPQLYKQMAIAADFGKVYTIGAVFRAEDSNTHRHLTEFVGLDLEMAFNYHYHEVLDVIGEMFVDIFKGLQSMYATEIATVNRQYPAEPFEWIEPSLRLEYKEGVAMLGAAGVEMGDEEDLSTANEKLLGKLVKQKYGTDFYILDKYPMAVRPFYTMPSPDDPKYSNSYDMFMRGEEIISGAQRIHDSELLTERAKAHGIILEKIESYIDAFRYGCPPHAGGGIGLERVTMLFLGLDNIRKTSLFPRDPRRLTPMLEEGIHIQNLRPLELDRVRVVYPSECRMRGTTYKGKLVLNFWSLNGNMQDVFEEVVGEIPIMVKSDKCNLAKMTPKQLIEKREDAEELGGYFIVNGNEKLIRMLTAQRRNYPLALSRKSWKEAGQFFSEYGVSMRCVRNDGANMVLHYLTNGTAKLRFFYQAQPIFLPLVMVLKALCDVSDQYIYNELIRGKEDDTFFKSCSANMLRLVQEGLFTQQVKKYIGERFRWYTDEEVTHFLSKESIAVHLNSNVDKFNLLVFMTRKLFAVAKGENALENADNPMFHEVYMSGHIYYTLFLERLSMFLSSVKSVIDRTIKKSLEIVRPLEYLLSTGNLLSKSGLGLLQRSGLSVMAEKINFWRFLSHFKSVHRGAFFAQMKTTACRKLYPEAWGFLCPVHTPDGAPCGLLNHFSEMCVLTVSLDGKLLGYLLRVMKVKCKIPQTLEIGFVQKTGKATQYPGVFLFSTPARMMRPVFNLTQSVELIGTFEQVYLDICVVGEEATTHQELRENGILSVLASQIPYPDFNQSPRNMYSCQMGKQTMGTPSHTLRFRCDTKMYDITPQSPLVRPTMHDHYHLDDYPLGTNAVVAVISYTGYDMEDAIILNKAAVERGFKHGVYKTQVVDLKVDVDGLPMIGSYDDPVCSYITAKVEKFKSTEVAYIYDVKLLGSLQKIAIMYWIRRPIIGDKFANRHGQKGICSIKWPQENMPFTESGLTPDIIFNPHGYPSRMTIGMMIESMAGKSAAIHGTIHDATPFQFSEDNVASDYYGKLLEAAGFNYYGTERMYSGVDGRELTADIFMGIVYYIRLRHMVGDKYQVRSTGPIDTLTHQPVKGRKRCGGIRLGEMERDSLLAHGTSFLLQDRLFHCSDKSSCVRCGSIISVTLPYVLRYLVSE-LASVNIKVMRETKYDIHKIPRNDPKLHPFEVAREYQRAMNAVKLDRVFAKPFLGSLGHSDVVNCLMKHQHSLSILVSGSCDGQVKIWNIAERKCLRTIQAHNSIVRSLCDDDVDIDTIIAKMDHHFKPLLMTVGEKVDLWEERQEPLRSYW-GVDSVQVKFNPVESAASDRSIILYDIRKANPLRKVVLDMRSNAICWNPMEAFHFTVANENYDLYTFDMRKLRIPIQIHKDHVSAVIDLDYSPTGKEIVSGSYDKTIRIFRSREVYHTKRMQRLTSVVWSLDNKYIVSASDEMDIRLWKSDASEKLGLKRYQSKLKEKFGNHPEIKRIARHRHVPKHIYREQRIMLESRKKKAKQIVLSNREYIDLTDEIRAKIEGCSKLYLSIHWWLLYQDALSKHRAALDDLNEVVLLKPDFAAARQQRGSALLKQGRLDEAHIDYEYLLREDPANEEAMLESLKRDAVDLLTKLWDVKLREMRSMAYEKLGDLMNAISDLRATTKMRADNTEGYLKLSKLHYDLGEAEESLTTIRECLKLDPDHKLCYKKVKKLAKGMFDECLEKMVHLIKSRCHCLNKAGDGLTVCSEALRLSPVLCDRADILIQSEDLDNDYQLAHNIDKRAKEGIQRVQKLLKQSKKRDYYKILANKKEIMRAYRKLAKKWHPDSC-EGED--KEMAEKRFMDIAAAKEVLTDKVKRHKFDNGEDPLDHDEQFTFRFHYMLVLFETASGYAVFKLLDEKKLQKTDNLFKAFQSAEKAGKIISLKHFEKFHDTTEALSSSTALIEGKMSKKLKKMLKKVVVKGDGDSLAVADAKLGNTIKEKLSINCVTSTAVQELMSCIRSQVENLIPEWSPDDESAMQLGLSHGIGRYKLKFSPDKVDTMIIQAVSLLDDLDKELNNYIMRCKEWYGWHFPELGKVVTDNTAYVKTILQLGMRSNATNADLTDILPEEVETKVKQLAEVSMGTDIADEDIFNISHLCENILELQEYRSQLFEYLKNRMMAIAPNLTILVGELVGARLISHAGSLISLAKHPSSTVQILGAEKALFRALKTKHDTPKYGLIYHAQLIGQSNPKIKGKMSRMLAAKASLACRVDALGEESSVELGTEHRAKLEARLKFLEEGGMRRISGTGKGKAKHDKYEHKSHLVQYKSASDSTIP--TKRKFED------------MQTKQEEEGAEAEGEGEDSPPMFSAKKMKKE-EHTGG------DHVPKKKKKKSKVKE-EPAQVAEQVEEDQAGEEPAVEEPAKKKKXXXGYVWPKEKSDIRKRVVISVALLIFAKVLNSYVPFIFKYAVDYLNLHTGLVVGYGIARAGSSLFNELRNAVFAKVAHDSIRRVARNVFLHLHNLDLNFHLNRQTGALSKAIDRGSRGINFVLSALVFNVVPTIFEVGLVSSILYYKCGQYAAVTLGCIGTLVVTQWRTKFRVEMNKAENDAGAKVVDSLINYETVKYFNNEQYELKKFEHASLKTATSLALLNFGQSVIFSASIMVLAANMTVGDLVMVNALLFQLSLPLNFLGSVYREVRQSLIDMQTMFSLMNIKNKPIWVSPEVRFEDVSFQPILDGLSFEVSAGQKIAIIGGSGSGKSTIVGLLYRFFDPLQGRICVAGKDIKNVSLRKAIAIVPQDPVLFHNTILYNLHYGNFTEDVYQASRMAELHDKILRWYETQVGERGLKLSGGEKQRVAIARAILKNSPILVFDEATSSLDSITEYKIMTALRAAENRTSICIAHRLSTVVDADQILVLDNGRIVEKGHLSLVSSLYASLWYKQHEMFISRPCHHLATAYEQTLLNVPETRVTELDNGLRVATEDSGIQTCTVGVWIDAGSRWETPKTNGVAHFLEHMAFKGTAKRSQTQLELEVENMGAHLNAYTSREQTVYYAKCLSKDLPKAVEILSDITQNSKFGEAEIERERGVILREMQEVETNLQEVVFDHLHSVAYQKTPLGMTILGPTENIKSIKRDDLVTYIKTHYKAPRIVLAGAGGIDHDELVKLANEHFGKVPHTYEDVNPCRYTGSEIRVRDDSMPLAHVAIAVEGAGWENPDNIPLMVANTIIGSWDRSHGGGSNXXXXXXXXXXX-XXXXSIQSCSTYNKHTKFRKRPFGSHAKAVTSSNCQKCRRIRSACIVIVIVEVTSNSACQRHNFRYQIQAITALLENKCTQAIHWPHRSSIIQTEQRPNSIGSD-VDDVSLTLLPARAPCLSLPPKLRATIRIRHIRXXXXKHSVHTLVFRSLKRTHDMFVANHGDLPPIDEHVEQHTFNIKARDQFGPVMHLVQQNSKLTTKNAVVLSSGTAVIPRRAPTMPKPEWHPPWKLMRVISGHIGWVRCLAVEPGNMWFASGSNDRIIKIWDLASGKLKLSLTGHISGVRGLAVSARQPYLFSCGEDKTVKCWDLEYNKVIRHYHGHLSGVYTLAVHPTIDILITGGRDAVARVWDMRTKAQIHCLSGHTNTVATLQCQATEPQVLTGSHDTTVRLWDLVAGKTRVTLTHHKKSVRALVLHPKLNMFASGAPDNIKQWRCPDGNFIQNLSGHNAIVNCLAMNGDNVLVSGGDNGTMFFWDWRTGYNFQRHSAPPQPGSIESESGIFTMTFDQSSARLITGEADKTIKIYKEDENATEETHPINWKPDIVVGSSSDYRIDVNKQDYLEKLGINVKARNFLVYQGAVESIAMKNPREITSLFEEISHSMDKSEEELFIKKKGIAAEKKEAQGEEAKKYQDMKDELQVQLQLFKLNYLQKDKDKKKKKLAIDEEIKGKKNIEKQLREELEVNKKRPAYIKAKENKSHIEKKLDTAKKSLAAARRAQTNHREIEDLEKELAETIEKFIAEDVELREEQKQRYNELKKTAAEQSAKYLKDLDSLEREHKADSDRLEAEQRKKNEIESRIREQDENKKRIEKLEDLAELKDKETEIKAKERINTELESICELGDAKVDRHGEERRKKKTEIVDHLKKLYPGVYDRLLNLCKPIHKRYNMAVTKVMGKSMEAIVVDSEKTGRLCIQYLKDQMLEPETFLPIDYIEVKQVRERLRNIQHPKGVKLVYDVIKYDPPPIKRAILFATNNALVCETADDANIVAFDLGDGKRYDAVSLDGTFYQKCGFISGGSAELERRARRWDEKEIHKLKYQKEKLTEELKDQLKKTRKESDLMVIQSQIKGLETRLKYCDIEEIRKRMHSREAKINDIRDKMNTVEDRVFSDFCEELGVENIRQYEERQTKASQERERKLQLENDRNNIENRLAYERGYAANLGGVKDEEEHLEKAKQKEREEMRAIEVEMNKIEQLKNDRLSQRQDCDRVEDEIGEKKRNLSNVQKEVSAVQKAQMTTECKMESRKADRHAVFLHCKMECIDLPLLAGNVQKSYENDNIKPDFSKLEMQEDIKLMKFQAPNMRALERLDGVKERLKETDTELSNLRKNAKAAFEKVKRDRACFEAVAQRVDSIYKSLANNASAQAFLVPENPEEPYLEGINYNCVAPGKRFQPMSNLSGGEKTVAALALLFAIHSFKPAPFFVLDEIDAALDNTNIGKVARFIRDRTESAFQCIIYSFESPFF----------LQPGDCTISRVFTVDLSVPDLVLLLLKKAITECNVFEIQNLYENSFVKLTDTYFLIFYKE--LYYRHIYADHRFESYFNYCRLFNHILSANPVSLELPNQWLWDIIDEFIYQF--QSFSLFVWNVHSVLNVLHSLVDKSAINEQLKEFNSGRDPDLVSGDFGRQPLYKMLGYFSLIGLLRLHSLLGDYYQAVKVLENIDLNRQPLARVLACQMTTFYYVGFAYMMMRRYSDAIRTFANILVYLQRTRRTLQIEMIEKQTDQMYLLLAICLVLHPQRIDESVLSQMQEKYMLKLQRGDLKEFETCPKFLSPVQMKVFMDEVNQQLTIRSYLKLYTTMPISKLAGFLLLCFKHKMQNLVLDGEFRSGSDVDFYIDKDMIHIADTKVARRYGDYFLRQIHKFDELYRMSAMCT-------------LRMNSWWSHVEMGPPDAILGVTDAYKKDTNSKKINLGVGAYRDDNGKPFVLPSVVAAEEAIMAKKLDKEYLPISGNAEFCQAAARLAFGDTSRVIQEGLNATVQGISGTGSLTIGAFFLRDFFTGNKEVYMPTPTWGNHIPLFKKAGFVVKQYRYYDPKTCGFDFTGALQDLAKIPEKSIILLHACAHNPTGVDPKPEQWKEISKVVKNRNLFPFFDMAYQGFASGDIDRDAAALRMFIEDGHEVALAQSFAKNMGLYGERVGAFTMVGATKEETAKILSQLKIIIRPTYSNPPLHGARIAQLILTDASLRKQWLKDVKGMADRIISMRTKLRDSLQKEGSTRNWQHITDQIGMFCFTGINADQVDRLTKEFSVYLTKDGRISVAGVTSNNVEYLAHAMHQVTKHLSSSTKHLLHCLLFLAVIFFFEYMSEDLDNPDPFVTYGSLLACFLYSIRFLTLLSLPQCLCNFLGLTLYNAFPEKVTLKGTPLLAPFIAIRTVTRGDFPELVGKNVHRNIQTCLNVGLENFIVEVVTDKPINLQKHPRIREVVVPNSYRTKTGALFKARALQYCLEDENNILGDGDWIVHLDEETILTENAVCGILNFVYNGKHEFGQGLITYANEEIVNWVTTLADSFRVADDLGKLRFQFYMFHRPLFSWKGSYVVTQYKAERSVSYDHGLDGSVAEDCYFSMIAYKKGYTFDFIEGEMWEKSPFTIRDFLQQRKRWMQGIFLVVHSPKIPLENKFFLAMSLYAWMTVPLSTSNLIFASLYPLPSTAWCNFLSAFVGAVSIYMYIFGVMKSFSLIRLGLKKLMLCMFGAFLTIPLNIVIENVAVIWGFFGKKHKFYVVQKEVVMSPESGGVHGG-----PIDMSAGAART--CESECFKGSWSKHKTAHK---AAEYAPWPGFEFTGILRPFPVTPRRDVPDTIQRPDYADHPEGRSACEEAIKGTTQIKVLNDDEIEAMRVACKLAREVLDVAYSAIDVGVTTDEIDRMVHEACIDRDCYPSPLNYYEFPKSCCTSVNEVICHGVPDLRPLQDGDIVNVDITVYHEGFHGDLNETFFVGNVSDESKKLVRVTWECLQKAIEIVKPGERYREIGNVIQKHAQQHGLSVVRTYCGHGIHRLFHTAPNVPHYAKNKAVGVMKPGHCFTIEPMIAEGTWRDVTWPDDWTAVTTDGKRSAQYEQTLLVTDTGCEILTRRRDRDGQPYFMDLYFVRRILKKRSQKRRVLLAQQLGAGSVDNLSTILGNVDERCADLHDDDDDDELCTYTDSSTFLKGTQSANPHNDYCQHFVDTGQRPQNFIRDVGLHDRFEEYPKLKELIRLKDELIGKTATPPVYLKCDLKQFDLRELKSKFDVILIEPPLEEYQRTQGVTNNEFWTWDDVMKLEIEEVAAQRSFVFLWCGSSDGLDLGRQCLRKWGFRRCEDICWIKTNIKNGHSKNLEPKAIFQRTKEHCLMGIKGTVRRSTDGDFIHANIDIDLIITEASEYGSIEKPEEIIHIIEHFCLGRRRLHVFGRDSTIRPGWLTAGPGLTNSNFNSDTYTGYFNIPNDYLTGCTDRIESLRPKSPPPKAKGQSGARAA--RMALNKLSIDQLNLQDKRVLIRVDFNVPMTDGKISNNQRIVAALDTIKYARDNGAKSVVLMSHLGRPDGQRVDKYTLRPVAEEVGKLLNCNITFLNDCVGPEVENACANPTKGTIILLENLRFHIEEEGKGVDASGKKVKADTKAVEAFRASLTKLGDVYVNDAFGTAHRAHSSMIGVDLPQRAAGCLMKKELMYFAKALDNPDRPFLAILGGAKVKDKIPLISNLLDIVDEMIIVGGMAFTFLKVAKNMEIGTSLFDEEGAKIVNELIEKAAKKNVKMHLPIDFVIGDKFDENATVGEATIESGINQGWMGLDCGPKSVVLFAEPIERAKVIVWNGPVGVFEWDNFAKGTKSVMDMIVKATQRGTTTIIGGGDTATCCAKWGTEDKVSHVSTGGGASLELLEGKELPGVAALTNAMRTFGDRPTAFQLEENGEYYYIGSEVGNYLRMFRGSLYKRYPSLWRRLVTVEERKKISALGLGPHTLATNITLLKASEVDEIFEGKDDKYKAVSISTEPSQPREPKPKRTNWMPTLPNSSHHLDAVPCSTAIARHRLSHKKVRTFPMLYDDLDPAGIHENASQSEVLVPIRLDMEIEGHKLRDTFTWNKNETTITPEQFAEVLCDDLDLPSASFVPAIAQSIRQQIEAFSTDNLLDDQTDQRVILKLNIHVGNISLVDQFEWDMSEKENSPELFALKLCSELGLGGEFVTAIAYSIRGQLSWHQRTYAFSEAPLPTVDVPFRNQSEADQWAPFLETLTDAEMEKKIRDQDRNTRRMRRLANFALMGQNASQLEKEIGFPQNEHYFGLVNFGNTCYCNSVIQALYFCRPFRDKVLEFKTKNKRSKETLLTCLADLFHNIANQKKKTGTFAPKKFIARLRKENEVFDNYMQQDAHEFLNYLLNTIADLLSAEKGWVHDIFQGTLTNETRCLNCESLSSKDEDFLDLSVDVDQNTSITHCLRDFSNTETLSSEHKYYCENCCSKQEAQKRMRIKKLPMILALHLKRFKYMESQNRHTKLSYRVVFPLELRLFNKTSNSEGDDKLYDLVAVVIHCGSGPNRGHYISIVKSCGFWLLFDDDLVDKIEASAIEEFYGLTSDTQKNSESGYILFYQSREMPDLDKWIEIARECKYLPENDLKQLCDIVCDILLEESNIQPVSTPVTVCGDIHGQFYDLEELFRNGGQVPDTNYIFLGDFVDRGYYSLETLIRLLTLKAKWPEKITLLRGNHESRQITQVYGFYDECQSKYGNANAWKNCCRVFDLLNIAAIIDDQVFCVHGGLSPEINTLDQVRTIERNQEIPYKGAFCDLVWSDPEEVDTWSMSPRGAGWLFGAKVTHEFMHINNLKLICRAHQLVQEGYKYMFDDRLVTVWSAPNYCYRCGNVAAVLAFNDVDNREAKIFSAVPDCDRVIPPRNTPYFLMKNVEGQSTADIDHLVIEDIKEHARQIEKSVNMKETRFVLRVLRSLVTTRKKLNARVLRKIISGFYTHSAEQRDALLAFVEPMDMDAHNVAKTSQLPLLPELDVYFNLLLLLYLIDLARYDSAVKCSDQLMAKVKSHNRRTLDVLAAKCYFYHMRCYELTDQLNQIKSFLHSRLRTATLRNDFEGQAVLLNCLLRLYLHYNLYDQAAKLVSKSVFPESASNNEWARFLYYLGRIRATQLEYSEAHKNLLQAIRKAPQHTAVGFKQIVQKLAIAVELLLGDIPDRSLFRQPTLRRTLAPYFQLTQAVRSGDLARFNEVIENFGQKFQADHTFTLIIRLRHNVIKTGVRMINLSYSRIHLADIAKKLKLDSSEDAEYIAAKAIRDGVIEATINHEKGFMQSKETTDVYCTTEPQAAFHQRIAFCLDIYNQSVRAMRFPPKSYNKDLESAEERREREQQDLEYAKEDDDDGFIVQVRLCVALAALILHTIWPNAIQELIFCLQPHRILLLDLLVIIPEEFQSRTMTREQIFRLVQEFLQLELKQAVIKCYHEQLLNLVL-QVADEYAVDAITSIYSHPEMKYPNSVLKLIDKISSVDVVLQKAIQESNPDNIYSLFIQVGEAHSRLLLDAVIDRPEHKILKLMTVVLQCSATPGYFPVDETCSEQAFNFWYTLQDDIRASDQ-RKIEAIFNPLFQSLIDLLVKVQYPTESIFSDEEKESFRCYRQDIGD--SFMYCYNVLRVTFLTSLLTHFTLSRPWQHLEAVLYCLGSIAENVEIDEDVYL-KEIVRALPTIPSSPRLLATAMEMMGAFCEEMLPNVISLLILGLKSVTIYATMALKDLTRECQAVIKPLAPDILCACQELKSKERARLMASIGHVLSTL-SEEIINYLNALLTPIINELQEISSVLEQMMPVLSRIGFKW-SHDEAITESLCECLKRAFTTL-LDPLVRDMIELLSHLY----------------------------------------------------------------------------------------------------------------------------------------KVKVKWKQLFPDVELNTPVLFKAQLFALTGVHPGRQKVMIKGTVINDDEWGTMLLMMGTVEELPSPKEKIVFAEDMSENELAAALDLPAGLTNLGNTCYMNATVQCLRTVPELKDLRNFQGSVTAALRDLYDAMDSATIHPLIMLQVLHVAFPRFAEKSEHGQQDANECWTEVMRMLQNFIDQYFGGTFVTMKCTEAENESIENFLQLSCFIS-HEVKYLQAGLKSRM-QENITKFSPTLNRDAVYKTSKISRLPAYLAIQFVRFFFKERESVNAKILKDIKFTLCLDVFEMCTEDLQLKLIPMRNKFKELEDKFSFVDDGSSNSAYYELQAVLTHKGRSSSSGHYVAWIKREWFKCDDDKVVTSEEILKLSGGGDWHCAYVLLYGPRVLEALINRKLPRELLLRIFSYLDVVSLCRCGQVSKAWNVLALDGSNWQIVDLFDFQTDIEGAVVENISRRCGGFLKKLSLRGCKSVTDISLRTFALNCNNIEELLNDCKKVTDSTCASLSRHLNLASCSEITDLSLKVLGEGCQHLEHINISWCDQITKYGVEQLARGCRKLRAFISKMCINDEAVVAMARYLEVINLHGCSTITDDAVQCIAQSCANLTYLCVSNCPHLTDQSLIAMAQRCLKTLEVASCSQFTDAGFQALARTCHKLENMDLEECVLITDNALFYLAAGCPCMQRLSLSHCELITDEGIRHLGNSVLELDNCPLITDASLDNLVMCLERIELYDCQLITRAGIRRLRPELKVHAYFAPVTPRQRYCRCCVIL??????????????????????????????????????????????????????????????????????????????????????????????????????????????????????????????????????????????????????????????????????????????????????????????????????????????????????????????????????????????????????????????????????????????????????????????????????????PTHIPWVEKYRPVDFADIVGNEEAVARLAVFAAQGNVPNVILSGPPGVGKTTTILCLARTMLGASYKDAVLELNASSDRGIDVIRNKIKMFAQTKVTLSAGKHKIIILDEADSMTEGAQQALRRTMEIHSSTTRFALACNMSDKIVEPIQSRCAVVRFNKLTDKQILNKITEICHKEKVNFTTDGIEAIVFTAQGDLRQAINNLQSTVDGFEKVDSENVFKVCDEPHPLMLKEMIKHCIDGNFEEAYKIIVHLHKMGFSSEDIVGNIYRVMKTYEMAEYLKLEYIKEIGLTHMRVAQGVTSLLQLSALVARLCKKTFKPPYLKKDHSLTKPYQGTGMTIPNWDFFGSTMVTTSFIRLTPDQQSRQGALWNNVV-SLPVRFVIG-----------SFPYVEDCRRSGPVFGNMDFDGLGIFLDTYANQNGVHNHGHPYISSMVNNGTLHYDHDRDGTHTELSGCEAKFRGVDHNTHILVRYENDVLTVKTNIEGKNEWKECFTASGVQLPTGYYFGVTAATGELSDNHDVISIKTFELEEDRSKILPSAAPPRDHIDDAMSGLKLFLIVVCAIIGIAVVVFQKQQETSRKRFYQTRDSFDEIVQKNCRVVDINDMFLPHSTVTHVPDIKQLGIDPIFPNRTNLLHIHNMAMSRAFFYSYILQKVDDTEPGFMYYFLSSIADVAANRFINSSAIYYGPNMAFTPSYKGFYNKTMPLFAPRAFRADDFNDPYHLRGTSTLNTIAAADLGAIDNNYTSTFYKINEWYNAWLPDLTKRHDSKTTYTVQITGTNETFVWHGPPAASDTPGPVKWVRPYFDCSRSNKWVLGASVPIPDIFPRHTGWRHIEIPLYVAVAVMELDFDRLDINQCSIGEGN-PAPNYFAGTARCKNETTQCEPIHGYGFRRGGYQCRCRPGYRLPRVVRTPYLGEIVERSTESEYRNGFQCERIGYIAVRTQNVETLTGLSRRIDPNFRGDVAFGKEEQLENQARMALRLANFISSFSQVVDPKELFAEFRVPDRPLTEDQIIGEALSAVIGDRRLQGLGVYFDRNQFFAPY--AYRLERNTRKFFVVDTMKINIRYNSSGIRYDHYPKQYQAAELGYWTSPYFDCGGFHNQWLVTYAAPFFGWDKIKSRLEFKGVVAVVKLDELDLNQCNAFKDTHKCDRKSSRCVPIPGRKYSGGYKCECLQGYEYPYNDPITYFDGQILEAPSRFDTLREFRLTTAAKYGGRYMVTMLPGDGIGPEMMNYVKTIFKVGGIPVDFEEVHLDSMHEDIDNVDEAITSIRRNGAAIKGNIETREHSKHFRSRNVELRLRLNLFANVIHCKSQPGIQTRYKNIDIVLIRQNTEGEYSCLEHESVSGVVESMKIVTKKRSEQIARYTFEYAKKYNRKKVTAVHKANIMKLSDGLFLNTARDMSQEYPDIEFNDMIIDNCSMQLVSNPHQFDVLLLPNLYGNILTNIACGLVGGPGITSGRNFGEEYAVFETGTRNTGKSVAGKNVANPLAMINASADLLEHLGLEKYAEIIRDSIYKVINEAQIHTPDMGGHNTTTDVVNYILDYKQIKYMTLSESPNEFKLDNPPTDCVQSVKFGLQSSQYLLSASWDCTVRLYDITNNQLRAKYTHSAPVLDCAFQDTYNVWSGGCDHQVKIYDFNSSSETVLGSHSAPIRCVEHAPEVNLMATGGWDACVKLWDPRTPCATGSYAQPDKVYSMAVCGDKLIVGTAGRRVLVWDFRNMGYVQQRRESSLKYQTRCIRCFPNKQGYVLSSIEGRVAVEYLDPSPELQKRKYAFKCHRNKDTTGIEVIYPVNAISFHSGYNTFATGGSDGYVNIWDGFNKKRICQFHKYPTSISSLAFSPDGTYLAIASSFLYETDEQRDIPPDSIYLRRVSDQETKMSSSLEQLRQYTVVVADTGHFETINKYKPTDATTNPSLILQAAQKSQYQHLIEDAVKFGKQHGKNLEATMDYLFVSFGCEILKIIPGRVSTEVDARLSFDVEGSVKKATNLIKLYEERGIKKERILIKLASTWEGIEAAKILEKQHGIHCNMTLLFNFAQASACADADVTLISPFVGRIYDWYVTATGKKEYEPADDPGVKSVTRIYNYYKRHGYKTVVMGASFRNINQVKGLAGCDLLTISPDLLEQLNEKNVELEQVMGVEKAQKLEL------ERQF---------------EGIRRFAADAVKLEQLIKGKHSMSVAAYRPRVSVYTEKNENSGVSVKLPAVFRAPIRTDVVNFVHTNMRKNRRHPYAVSKKAGHQTSAESWGTGRAVARIPRVRGGGTHRSGQGAFGNMCRGGRRFGPTRVWRRWHRKINRNQRRYALVSAIAASGVPALVMAKGHRIEEVPEFPLVVSDKIQEYTKTKQAVRLLRKVRAWRDVERVYNSKRFRAGRGKMRNRRRIQALGPVVVYSKDSGLTRAFRNIPGIETINVEKLNLLRLAPGGHVGRFIIWTESAFRKLDDLYGTWKSESKMKRHYNLPQPKMANADVSRLLKSDEIQKVIRPRITKVIRRKVKRNPLKNPLVMRRLNPYAPVLKKYARLTDERRKWARLKKKKLGDKVKDTKKKEYKAELLKKKKLAQGNVLKKILDAIKDLVNEASWDCSPSAMSLQAMDTSHVSLVSVNLKADAFRKYRCDRNVTLGMNLGSLAKIVKCAGNDDEITIKAPDDGDKITLVFEANSDVEYSEYEIKLMNLDTEYLGIPDTPYAVTVKMPSAKFQRICRDLNQIGDAVTISCAKDGIRFSASGDLGSGSVHLSQTANADKPEESVTVKMSEPICLSFALKYLNNFSKATPLSSQVSLQLSPDVPLVVEYEIGYIRYYLAPKIDDADMISRLA----ASAFVANTRAFSTSKQRCSKVAVLGASGGIGQPLALLLKHSPLISHLSLYDIANTPGVAADLSHINTRAQLSGHLGPEQLAAALQGCDVVVIPAGVPRKPGMTRDDLFNTNASIVRDLVEACAKNCPKAMVAIISNPVNSTVPIASEVYKKRGVYDPKRIFGITTLDVVRANTFVAELKSLDPTKVNVPVIGGHAGITIIPVLSQATPPVSYPKDQLDALTKRIQDAGTEVVKAKAGAGSATLSMAYAGARFAISLLEAINGKNVVECAYVKSDVTEATYFSTPILLGKTGIEKNLGLGKLNDYEQELVKNALPELKASIKKGEDFVNKEFEWLLNEEVNVILNQLIILECCRRFPV-VRPEKYQL-MMKMNVTLSGDNITYADITMKL-HKHTIRTNIQSDAAWKLHQIQDATNHLTAALCFLKYDFKTADEVIQMINGVMSCLQKGRSSLIVPKKRTIEELESSRNMKSIKPSLPNDLAVNFHIQAHKLVCAVYHM-QKDPVKVHD--QSVPWLSEALVLFTVALQFCQQLKDKVSVFQYKDMIPTFIDILKNLGADISDLKQIIDVCDVTFKDVESVL--NSVVS-LLIAFCDKLTKPPSNKICLR--VLQNLFEGLKYQVYVSLVKVAADQILLVFNDIVKVKSSFGNDKVQRLFRLLHDALSELASKVMIELLSTYTEENASQARDDAYRCIVSFLADPNTFLMDHLLTLKPVKFLEGEPIHDLLTIFVSEKLNAYINFYNSRKDFV-DSLGLLHERNLQKMRLLTFMQMAESKKEIPFETIENELQIKASDVEAFVIDVLRTKLARARVDQVNKKVLVSSTMHRTFGRQQWQQLRDTLNLGQVEHTIKT---FLG-TR-KKK----IEEGKQAIDEYEERSMIEELSDFKELIKILVNWINDELSDQRII--VKDLEEDLYDGQILGKLVEKLSGMRLDVVEVTQNEDGQKHKLRTVLENVNRLLGVKWSVEGIHGKNLVQIIHLLVTLIRHYRPPI--RLPVNVTVNLVVVQKR-EQLTGTYDELGMRVEPRDAFDTLFDHAPDKLQIVKRSLINFVNRHLNKINIEDLDPNQFSDGLLLVFLMGSLEGYFVPLGIFTTP-TDPIADVTTKETALQPDNYVNTSKLHNINVAFQLMEDAGIRQKVRAEDIVNADLKSSLRVLYMIFTKFSPGPAKIPEEVMKQAHEELLDYGISVMEMSHRSSQICETTEKDLRDLLNIPDNYRVLFMHGGAQGQFSAIPMNLCAADYFVTGTWSAKAAKDASKYVQYTGISDQSVWKLDPSARYLYYCDNETIHGVEFPLVCDMTSNFLTRPVDVSKYGCIFAGTQKNCGMAALNIAIIRDDLIGNMDICPPVFDWKVINDNKSVYNTPPCYAIYVTLCLKWIKKNGGVEEMKKSEQKSRLIYDVINDSNGFYVRSRMTVPFRV-GGPNGNEELERKFVVEAEMIQLKGHRTVGGMRASLFNAITSQELKPPYRTRPEWRDVEPIVAIQYSEQFADAFDYFRAILRDELSERALELTRDCITLNPSNYTIWYFRRRCLRALDKDLREELPYIEDIIMGNPKNYQVWHHRKAIVECLEKKLIQRALDPKNYHAWQHRQWVVAEFALWDDELRFTEELIDDDVRNNSAWNHRFFVVSHTNDIKLITALEFEMRNAMNIEEILNFFHKVIPSYPLHPQLDGSFSLGVNFEMRWKYPNRGVKTKLVTFLLVSLCLSLLLTVFIAIIVDRFDELLKFVPHMHNFLSRQRVRYKIYVMNQADNLRFNRASLINVGFLISRQECDYIAMHDVDLLPLNPDLSYAYPTVGPFHVAAPELHPKYHYKSFVGGILLLTRVHFELVNGLSNKYWGWGLEDDEFYARLKEAKLNISRPVGIKTTAKDTFLHVHDRSNRKRDTARLLNQKEVTRKRDRQTGLNTVQYEILRQNELTIDGAPCTVLNVKLICDHRITPWCVFSNILPGDVITESGFMRGHGELSASVAGAVEPINKLISVRPVKTRFNGDIGDVVVGRVIEVQ--QKRWKVETNSRLDSVLLLSSVNLPGGELRRKTAEDELMMRKYFKEGDLVVAEVQSVFQDGSLSLHTRSLKYGKLGQGVLVRVSPSLIERRKHFHNLP-FGAHIILANNGYIWISSCGFVREAIARLRNCVLVLASAKMMISDTSCTYCYEAS-VARLTKENSTRNIN----XXXXVTFGTVVIGGVTRLTKSGLSMVDWHLFKEFPPMTAEWEREFNKYQQYPEYKLRNSNMNLEEFKWIWWMEYAHRSLGRSIGAIFFLPAVFFWYKKWFNRAAKIRVVALGSLLAFQGLLGWFMVKSGLQEKPRVSHYRLAAHLGTALLFYSLAFWSGLSHLLPSLKMLSFKKTKGLIFVTALSGALVAGLEAGLIYNSFPKMADRWIPSDLFAKPLWRNFFENSSTVQFDHRLLGELSVTACWLRSRRLPLTPRMRLAANMMMIALAQVSLGIATLLLYVPKPLAASHQAGALTLLSTALWLGHELKLVRR-IAKWGEGDPRWIVEDRPDAVNVNNWHWTEKNASQWSKDKLTELLSGVRVDDDQLGWCIDEVTSIEGEAVANNRKAKLIFFYEWVVKMVNGS-TVVGKIEVPNLSEENEPKDVEVEVSVTDRTLEALMKSKGADLIRKQFAEYIKSLREEFSQGMILPTAKGTINSAVNASSKLETAMLKLETMKCTADEFYRALTMVAAFTNGNCNGRFELYDGTVSGTFTRLKVIGQRWRLKSWPADHYSDVIEIKQKDDCTLVQIGVPKSKLDETEHGWKNFYFQRMKHTFGFGAMLMDPESFLEMANQVTKLKMFPYFELAHAIISCLYVREDLAQGSHPFSRKHPFACWASCMISIFSGLILANFLLGEPILGALKSSNQVLLATGVWYLMFYSPFDVAYKICKFLPVKIVLAAMKEVIRCKKVHDGVVHAAKIYPNGYLNMVIIGTVKGNGAAFLKVIERIYRGVWTPNAIEFLSPTFPTKASIAASIIFIIDKKTDWISAPHSLVYFGIVIFFVYFKLSAMLLGIHDPFLPFENLFCAIFLGGLWDALSRVISGKGSGDMNNPKSDVARNGKSEATKKKD?????????????????????????????????????????????????????????????????????????????????????????????????????????????????????????????????????????????????????????????????????????????????????????????????????????????????????????????????????????????????????????????????????????????????????MQDGVPTFKCVLVGDGGTGKTTFVKRHLTGEFEKKYVATLGVEVHPLLFHTNRGPIRFNVWDTAGQEKFGGLRDGYYIQGQCAIIMFDVTSRVTYKNVPNWHRDLVRVCENIPIVLCGNKVDIKDRKVKAKSIVFHRKKNLQYYDISAKSNYNFEKPFLWLARKLIGDPNLEFVAMPALAPPEVQMDPEWQAKLEQEMKQAQEVSLPDDDDDDLVAEYFTPLLKESKFKETGVLTPEETVSR-----HQ----KWSA---LCTVPGFKSPVSLVPVPCYKRCKDIEEKVIDAD-GGWVDTHDDDDDDGVAEDMDEFMDEEDKATTIVQTRTYDLNITYDKYYQTPRLWLVGYDETQKPLAIEQMYEDISQDHAKKTVTMETHPHLPG-VMASVHPCRHAEVMKKIIQTVEDGGKLAVESYLIIFLKFVQAVIPAIEYDFTQNFTLMSRFDNCIPYEKLQANLKIVKSRLNRPLTLSEKVLYSHLDDPQNQDIERGKSYLKLRPDRVGMQDATAQMAMLQFISSGLPRVAVPSTIHCDHLIEAQIGGVKDLARAIDINKEVYNFLSTAASKYGVGFWKPGSGIIHQIILENYAFPGVLLIGTDSHTPNGGGLGGLCIGVGGADAVDVMANIPWELKCPNVIGVHLTGNMSGWTSSKDVILKVAGILTVKGGTGAIIEYFGPGVESISCTGMGTICNMGAEIGATTSIFPYNNRMRDYLIATNRKEIADLADENTELLTSDKNPKYDQVVEINLSELEPHVNGPFTPDLAHPVSKLGESAKKNGWPLDIRVGLIGSCTNSSYEDMNRAASVASQAIKHGLKTKSMFTVTPGSEQIRATIERDGQAQILRQFGGVVLANACGPCIGQWDRKDVKQGEKNTIVSSYNRNFTGRNDANPATHAFVTSPELVTALSIAGTLEFDPLNDELTGANGEKFKLKAPSGDELPNRGFDPGEDTYT-PPADGSRVSVDVDPKSQRLQLLKPFDKWDGKDLTDMVVLIKVKGKCTTDHISAAGPWLKYRGHLDNISNNMFIGAINAENGVANKVKNLLTNEWGPVPDTARYYKS-------------XXXXSREHAALEPRHLGGRAIIVKSFARIHETNLKKQGLLPLTFDNASDYDKIQPNDKISLLDLQKLAPG------MFKNTFQSGFLSILYSIGSNPLQIWDKKVKNGSIKRITDNDIQSLVIELSGANVATTFITCPAQARLSLGIKLPYLVMIVKNMKKYFSFEVEVMDDKQCKRRFRASDFQSQTRVKHFICTMPLRLDEGWNQIGFNLADFTKRAYGTNYVETVRVTVNANCRLRRIYFADRLYSEDELPPEFKLYLPITASKSS---------------RLLTQAKYRYQDQAKRDVQNALQHYRNLVDKYVFPDGTVRDLLCLDGTVPVSYRYNIPVCIWLTDTHPYTAPICYVRPTHDMTIKQSKHVDGSGRIYLPYLSDWTAKG-SDLLGVIQVMIIVFGETPPVYAK-PKPYPTTNSGTITEEHIRASLLSAVEDKLKSRTREQVQAEVEVLKKTENDLNKGKTKLEEIVSRMEGEVEVEDAKDAQLAELDVDNAFGPTQPLYKQLLNAFAEENAVVDAMYYLGEGLRKGTIELEVFLKHVRELSRRQFMLRALMQKCREKAGLPAGSKRYVSQQQKNEANGSFDCIVCLFLA------EVVIGVPA-----------AKIGVAAQNSYKVASGAFTGEISPMMIKDVGAEWVILGHSERRNVFGETDELVAEKVDHALDEGLKVIACIGELLKEREAGKTMDVVSRQMKAIADKVKDWNLVVIAYEPVWAIGTGKTATPEQAQEVHAYLREWLGTNVSKDVGESTRIIYGGSVTAANCKELAREKDIDGFLVGGASLKPDFVEIVNAKQMASGPSQKRKIVANGVFKAELDEFLRRELAEDGYSGVEVRKTTNRTEIIILATRTQSVLGEKGRRIRELTSVVQKRFGCEDGVVEMYAEKVSDRGLCAIAQCESLRYKLIGGLAVRRACYGVLRFIMESGAKGCEVVVSGKLRGQRAKSMKFVDGWMIHSGEPTNDYVDTAVRHVLLRQGVLGIKVKIMLPWDMAGKKGPKRPLPDNVKVLEAKEEQYPAQPYSEPKEEVISNAIMQEIAINDPATLPVQNSPMDALKEWSMTTLKCTRQLVNEKLGKCPRTVDVELEAEIEHLRDTQRKYAAILKMAQQMTTQYQLIASQITLYEMMNEMSMRESKSPALNLGVDFRQNADTLRTVAKNGEKLILALRFFVSNLSTLANKSIEDCVFTIRSFESARLEYDAERN--GDKLRSRYEQLRDDVQVKMKFLEENKAKVMHKQLILFHNAFAAY-ASGNASALDSTLKQFSISWLEK-MIGKLFIFAIRCWSSRPMDVYDFLDGAMIKTESIVCVTSKKVELQQVQDSQALIRARLLRENGKYLPKQSFLMRRHFFNNTDNGYFYMGRNRPSVQANPMTDPTMMTDMLKGNVTNVLPMILIGGWINWTFSGFVTTRVPFPLTLRFKPMLQRGIELMSLNASWVSSASWYFLNVFGLRSIYTLVLGENNAADQTRVMQDQMSGAALSMPTDPKQAFKAEWEALEISDHQWALRNVDNELTGGPSYQEALKQ----MAHYKGAASEAGRALQLQKKREKAKEEMEIRKRKIEDELKINNIGNKFASHYDAIEAQLKSSTIGLVTLDEMKARQEDVVKEREKKLAQKDEERKLREIEQKKEQKERQKRQIQTLSFRFDDDGDEEDEEDGEDEQDAIRVAMNQXENDKESDMPDKRVSESDSCGDESNSSFNAMAKKRKMTKNPDVDTSFLPDRAREEEDKRLREELRQQWEHKQEKLKDEDIEITFSYWDGSGHRRSVKMKKGHSIYQFLHRALEILRKEFHELRAVSADQLMYVKEDLIIPHHYTFYDFIVTKARGKSGPLFSFDVHDDIRLLGDVTVEKDESHAGKVLLRSWFERNKHIFPASRWEPYDPTKNYDKYTISDRKNKMFPSKQKTVVPTVPESILKKRKLRAKQKAKNAVNAIKRRQVKKQKRSLIFRRAEQYIKEYRRQEHDVIRLKRQAKLHGNFYVPAQPKLAFVLRIRGVNGVSPKPRKVLQLFRLRQINNGTFVKLNKATINMLRIAEPFIAWGYPNLKTVRELIYKRGYGRENGQRKALTSNDIIEKKLGRYGIICMEDLVHEIYTVGAHFKQANNFLWHFKLNNPRGGWRKKTTHYVEGGDFGNREDKINNLLRNMISVLTAEQLWKGVTSVSNAGRKRGRGKGAGRKTIKDLNKGQVIGVGKVNMIWPGLNAPVVRGRELVERRELPPDKDYEEKLVKIRNEMDVHRKVRIHPLERGWSGTRMAGRWIGPPDPVAGDEFTGFDTAVLQMRPLFRMTGNFGKTKRIAVLSITGNKNGLAGFAIGKAKDARTAMRQSKNRAGQRLRYLELFESTVLHDFYSRCGATAVFVEKKPRGYGIVAHRCIKAICETIGITDLYAKTEGQTKNYINLTKAFIVGLMQQKSYQQMADEKRLHIVELREERDQYPVVLASPSSCRTDAEIPSDEHLDFHLYIYNGKVKQRDVKRPPNYMGSIGWEQYLKKRDVTKNRAKVRVALICKHYSLTSFLYQRKLHFRIPPRFSDEKVKWINKQRVLIFASRGITYRDRHLLLNLRGLLPHSKADSKME--KKDINEIAEMKNCNKCIYFENRKQADLYMWMANIPNGPSVKFLVENVHTMEELKMTGNSLKGSRPLLSFDPRFDQSPLLKELFIQIFSTPNHHPKSQPFIDHVVTFSCLDNRIWFRNYQIVDESDASLAEIGPRFILNPIKIFNGSFGGPVLWSNPSYISPNRHRAIIKKQAAEKYKHDQXX--RLVLVRMAVGKNKGLAKSGKKGVKKKVVDPFTRKEWYDVKAPSMFNVRNVGKTLVNRTQGTKIASEGLKGRVFECSLADLQNDEIAFRKFRLIAEEVQGKIVLTNFHGMDLTTDKLRSMVKKWQTLIEANVDVRTTDGYLLRVFCIGFTRKWPNQIRKTSYAQHTQVRAIRKRMVEIIQREVTSADLKEVCNKLIPDSIAKDIEKSCQSIYPLHDVMIRKVKVLKKPKFDLGKLLEMHGEGKTTTSTEPMKVDRPEGSPPPTRRSRIKIGIIGGTGLGRDTEFLLDETPFGVTSDGTVVEGSLQGVPVVVMARHGREHTVSPSNVNYRANLWALKHQLACTHVLVTTACGSLREQVKPGDVIPDQYIDRTRARSFYVSHIPQARPFVQDILVEHPRVTAVTIEGPRFSTLAESRLYRTWGADIVNMTTVPEAQLAAELGLPYAALALVTDYDCWHESEDESVCVELVQRLKQLSVIAKKVLAVAVKRIAQSAIMTRRALHFVLKMTQRQENIFFFKDVLGMKVLRHEEFEEGCKAACNGPYDGKWSKTMIGYGSEDQHFVVELTYNYGIGSYAMGNDFLGYTIASQTAIDRVKQHNWPSAEVDGKLVVLSPDGYKFSLLGDEARASPDPVVKVTLASSHLERSINYWHDLLGLTVHERDEKSALLSYGEHQCKLELVDIRKPVIHAKAFGKIAFSCPTTELPAIEEIMKINKQKILTPLVSLDTPGKATVHVVILADPDGHEICFVGDEAFRELSQVDQQADELLRAAIDGDKSDDWYSEKNKKKPPARLPPLPTPKDLLKLYRVRASRHLSQNFLLDKISQRIVRSAGYVLEVGPGPGNMTRHILEQGPRELYVVEKDRRFLPMLEMLADIAHPGQMKIVIGDVMDFNFEQLF-PEDAKRDWLDIRIVGNLPFNVSTPLIIKWLRQISQRTGLWRYGRTQLILTFQLEVAQRICAAQRCRLSAMCQNCSVETSTSFVPPPLVDVGVVKFTPLSPITLPFNIYEKFNRHLFHKFIRNDLSMEMFSRTDIDPCYMLSTQEIGELCKIYQQFCEESGLFDYDYRARKK-MLSRIGVGFGIRSRIRLHTTTVS--RLIPIVIEQTGRGERAYDIYSRLLKERIICVMGPITDDLSSLVVAQLLFLQSESNKKPIHMYINSPGGVVTAGLGIYDTMQYILPPIATWCVGQACSMASLLLAAGTPNMRHSLPNSRIMVHQPSGQAIGQATDIQIHAEEILYLKKRINSLYQKHTKQDLARIEDVMERDKFMSPEEAKQFGLVDVVLEQPPAKEKRLDS????????????????????????????????????????????????????????????????????????????????????????????????????????????????????????????????????????????????????????????????????????????????????????????????????????????????????????????????????MQRVVKAKTHKGKKVLQKRQAQIIEPNKAVFIRGSTANDKMVKLMKDICLIKKPNSVFLSKKNPFENSTKIEFLTKNDSSLFMFASHSKKRPNNLVFGRTFDNHILDMVEVGVENYKPLADFKTPKISLGTKPIIIFGGEPFEEMKRFKNLLLDLFSGDAIRLTGIENVVMILAAEILVRNYRIALKKSGTKLPR-VELEEMGPRLDMVMRRTHLASDDLFKRACKHPKQTKPKKVKNIKKDPFGSTLGRIHMERQDYKLQVRKLKGLK?????????????????????????????????????????????????????????????????????????????????????????????????????????????????????????????????????????????????????????????????????????????????????????????????????????????????????MNRLFGRGAPKEPPPNLTDCIANVDSRVESIDKKILRLDAELIKYKEQMKKMREGPGKNAIKQKALRVLKQKKMYEQQRDNMHQQSFNMEQTNFATQMLKDTKTSVDAMKLGVKQMKKEYKNINIDSIENLQDELEDMMESANEVQEVLGRSYGVPDVDEEELEAELEALSDELNADDTTYLDEVSAPNAPTKEPGAESVNADGQLVDEFGLPKIAAMRAAVGAVRGKHTLPDLPYDYNALEPTISAEIMQLHHSKHHAAYVNNLNVAEEKLAEATHKNDIATIIQLESALRFNGGGHINHSIFWKNLSPKGSGDPDGELLKAIKDNFGSIENMKSQLSTAAVAVQGSGWAWLGFCPKSKRLVVKPCPNQDPLQPTTGLIPLFGIDVWEHAYYLQYKNARPDYVKAIWNVVNWKDAYSRLSQFIDVDVNDGFVEDVLQSGVDLRSYSKQVEKQLRDVENGCVADYINESGNIANLHAQITSCDQILERLEHMLCTFQADLGNICQEILTLQEQSVSLNVRLKNKQAVRSQLSQFVDDMIVPQPVICHIFDTPASEKQFLEQLHVLDHKIAFVKEQSFRDAHSCHDVAEILNSLKLKAIAKIREYVLKKIQSCKKSLSNYQIPQNALLKNKFFYQFLLTHDRGSAREIQSEYIDTMSKIYYSYFKEYLHRLVKLEYEEKPDKDDLMGGEDVSKTSLFGKPSLKNKSTIFTMGCRADVLSTDLEAPLIVPHAAQKAETKYPMESLFRSHQYALVDNACREYLFVSEFFMANAPTAHELFNAVLGKTLSLMTKHIEDQFQSSYDSIALFLCLHVVYRYRLLAHKRTVPALDSYWESVVKCLWPRFEYVVRLHVQSIKSCDATKLGMCDTRPHYITRRYAEFSAAVMTVNDTFPDERVSILLGSLQNEVENFILKMASQFQHAKDQLIFIINNHDMILGVLLEKTKEDSKESESIKLQLNKRIQDFVEELLYPHFGGMICFVKDCEAYLEKNDVEALKREEKKVTSLVKSFNVGWRKALEDISREVMNCFSNFKNGNNIQQAALTQIIQYYHRFQKIVSQAPFKNNPVRNEFINIHQLMVEVKKYKTNFMK-EALLIVCLIAVLLDTCRAYFLTIDAHADECFFDKVTTGTKMGLTFEVVEGGFLDIDVKITGPDAKVIHNEERGSSGKYTFAAHMDGMYTYCFGNKMSTMTPKVVMFSMDVGEAPK-TGQMDTEGDHNKLEEMIKELSSSLTAVKHEQEYMAVRDRIHRSINESTNSRVVSWAFFEALVLVAMTLGQVYYLKRFFEVRRVVMRLTLILKLPKNYSNLPERYIKKQTEFVVWETPKLPNYQRRVIRWRYDIHRPWEEGFQRNAPNVFVYVEPIFRGDRVEILRGKDKGKQGLVNYIVKERNWVLVEGLNCEEKPLLINRDVALVDSTDNKPTHVEWRFDEEGNKLRVSARTGRIIPIPSAFETIDYKYREQSKDTAADEVKKVTFVP-KVCTFEMEIMQEMNISEDRVPYPMYWYMSSNEQN-LVAHLIANKTDAVLWLTRLATIVFTVLYVTGSPYSYYQKALIGNGATSALRLHQRLPPFRLTREYFSMLLLEDSAHYLLYSIIF-VPIT-LVLLPIALFALLHFVVMLDRMGSMVE---RQRSILQTVSLCEIFLMPLVLVSILTGAYLFTPFIYYRFLYLRYASRRNPYTRNMFHELRLATEQM----CPLVIRPIAFLERLAPVILLAILVYYNFPKLDPSEKEHLKLPKNIDDAKKLGRVLSRYSDKTVLSGFLVTYVFLQSFAIPGSIFLSILSGFLFPFPLALFLVCLCSAVGASLCYLLSYLVGRRLVLKYLPHLLNYIIFLRTTPFLPNWFINLASPVIDVPLMPFFVGTFLGVAPPSFVAIHAGTTLHKLSSSLSWTSIIMLLLFAGLSLVPVLFKLKGKF????????????????????????????????????????????????????????????????????????????????????????????????????????????????????????????????????????????????????????????????????????????????????????????????????????????????????????????????????????????????????????????????????????????????????????????????????????????????????????????????????????????????????????????????????????????????????????????????????????????????????????????????????????????????????????????????????????????????????????????????????????????????????????????????????????????????????????????????????????????????????????????????????????????????????????DDEGEDLFGPELDVYDAVEAEMRERDKGKMRRGLESIDNLDDTRGMSVREWVSQAPKREIYNRFKNFLRTFREKIRQMEENKQSFDVDYILANDEQVLAFFLPEAPIEMLDTFNLAAKEVVLSMFPAYDRIAKEICVRITDLPLLEDLRSLRQLHLNQLIRTHGVVTSTTSVLPQLSLVKYDCQKCGYILGPFVQNQEVKPGSCPECQSLGPFSINMEETIYQNYQRITIQESPGKVSAGRIPRSKDAILIGDLCDSCRPGDEIELTGIYSNSYDGSLNIANGFPVFSTVIMANHILKKDTDEDIKKIVALSKDFIVDRIMASIAPSIYGHKSVKRAIALALFGGQPKDPAKKHRIRGDINVMICGDPGTAKSQFLKYVTKIAPRAVFSTGQGASAVGLTAFVSPVTKEWTLEAGALVLADRGVCLIDEFDKMSDRDRTSIHEAMEQQSISISKAGIVASLQARCSVIAASNPIGGRYDTFSENVDLTEPIISRFDILCIVRDEQLAKFVVRSHIKNHPIPQDLLRKYIMYAKERIHPKLDHERIARLYSELRRESELTGSIPITVRHVESIIRCAEANAKMHLRDFVDVNMAIRVILESFITQKFSVMRMGLLDDIEQYFGTRNLYDVLGVKQTASEREIKSAYRKLSLKIHPDRVQDDKKVEATKKFQVLAKVHFILSDEDKKAAYDESGIIIDEDEMDNQANWEQYWRLLFPKVTVKDIDSFLAKYVGSAEESGDLKELYLRYKGDMDKIFESAICFDEERTRCLLMEMVEAGEVPKYDQFVNEPESKKVNRLKRAEKERKTFEKEEKKKPDGDGDLVKAIQARSKGSFDSMIASLEKKYAS--NGKAKTNKKKDVVLILAISLMVLGKIGVKKRRKLEMKAEKRAMRDRELEERDERKQRMTIEEERRKDEQRAKAEQEKREEEERKIKEEKERQELEEYLKLKQSFVVEEEGFDQDIDENEAQDALKEFIDYIKHTKVVLMEDLAARFGMRTQDVINRIQDMLAQELLVGVIDDRGKFIYITREELESVAKFIRQRGRVSIAELVESSNSLIRIQPDVEALEVEARVTAAKHVANMLQRPDQLEKVDQFKRRVMHKKASVEGMLKSAMQTQLDGVRTGLIHLQTSLQDIQDVKAKEIEETFVIPNLDKLRQVREESLRHSQYAAAMENLKHIFNVPESVQKTRDWINEGNLLLAHQLTDLENSRDDLLYELHRLPSTSAADKNMLKHYFSDVEKLSEELGKQIWLIIRLCLNSVRTEPQVIVTALRIIEREEKLALKRFDST-GFMPQGRPKQWRKRVFEILEEAVSERIAGNQFESRHENKMWLVRHLEVTRQLILEDLKVIKTACVPCFPPHYDIVKELRLYHRCLSFHLQDL-AQLEGNEYVTLLNWVQAYSAAFAATHADKAFENKLRGDLQNNLAKGYITALLQNLREDMAKTNVRSRERPDESSSSTYRTIITGQVYQAVINMISVTVTFTPRTQIL----GHICPFVTCPVD--DEQTFDARS-------LTSLSAAFVCLDRVQLVSYRFLKIQLLFVLDDLENFVCSRLMIVMHHHHHHHLREELAEA--KAIM-KETKLTEELRHLRK-----ICLRNGRIQQIQLLGKDLKDADPFMLATPCSATRTLLQALFLFGLCKCEDVFESE-DAQMDNNLKASR--SPTDDNVVAREEEAIQLDGFNFEQMEKMREKAEKFVFQAEVSRLMKLIINSLYTNKEIFLRELISNASDALDKIRFLSLTDKSALDAIDELVVRIKADKDNAVLHITDTGIGMTKDALITNLGTIAKSGTSDFLQKVEETGNLNELSDLIGQFGVGFYSSFLVADRVVVASKSNDHPVQHIWESESGEFSVVEDPRGDTLKRGTQVSLHLKEEAKDFLQEDTLKALIRKYSQFINFPIYLWISKTQIVEEPIEDE--TTEEEKKSEEGDEAKVEEEKEE-KPKTRKVEKTIWDWELINTSKPIWTRKPDSITDDEYHEFYKSITKDTQEPLARTHFIAEGEVTFRSLLFIPKVQPGESFNRYGTKTSDIKLFVRRVFITDDFQDMMPTYLSFIRGVVDSDDLPLNVSREMLQQHKLLKVIRKKLVRKVLDMINKIADDLYEAFWKEFSTNLKLGVIEDAANRQRIAKLLRFHSSSTSSTGWTSLGDYVTRMKEKQDHIYYVAGANWEEVSKSPFVERLLNLGYEVLYLTDPVDEYCLSNLPEFNGKKFQNVAKDGLKLDDSEKAKDKQEEYEKRFEPLLKWLTSEALSERVSSVKISQRLHDSPAALVASQFGWSGNMERLARSNAHAKSQDITRDFYLNQKKILEINPRHPIIKELLRRAEKDDLDANTRQMALLMFEVATLRSGYMLEDPIDFSRRMDKHIIKSLNVDDSVPVDDEED-----SDKDENEPIEDVTDSEAEIARKRKKNGLTVGEMTVMTVGEIIKELIVAHQENRDVNLSKLKSDISRKYGLESQPRLVDIISAVPYEYKKILVPKLRAKPIRTASGIAVVAVMCKPHRCPHINMTGNICVYCPGGPDSDFEYSTQSYTGYEPTSMRAIRAHYNPYLQTRNRLDQLRQLGHDVDKVEFIVMGGTFMALPAEYRDYFIRNLHDALSGHSSSSVDEAVKFSERSRTKCIGITIETRPDYCLKRHLSDMLDYGCTRLEIGVQSVYEDVARDTNRGHTVKAVTESFQLAKDSGFKVVAHLMPNLPNVDLERDIAQFVEFFENPAFRADGLKIYPTLVIRGTGLYELWKTGRYRSYPPSVLVDLIAQILALVPPWTRVYRVQRDIPMPLVSSGVEHGNIRELALARMADFGTKCRDVRTREVGIQEIHHKLKPYQIELIRREYVANGAWETFLSYEDVEQDILIGLLRLRKCSGETFRPELKNGCSIVREHVYGSVVPVSAKDPTKFQHQGFGMLLMEEAERIASQEHGSTKIAVISGVGTRNYYRKLGYQLDGPYMSK

>Speleorchestes

??????????????????????????????????????????????????????????????????????????????????????????????????????????????????????????????????????????????????????????????????????????????????????????????????????????????????????????????????????????????????????????????????????????????????????????????????????????????????????????????????????????????????????????????????????????????????????????????????????????????????????????????????????????????????????????????????????????????????????????????????????????????????????????????????????????????????????????????????????????????????????????????????MQAPIILLKEGTENAQGKSMVLTNINACQAVAEAVRTTLGPRGMDKLIVDSKGSVTISNDGATILKKLDIVHPASKTLVDISKSQDAEVGDGTTSVVLFGAEFLKQAKPFVEEGLHPQVIIKSYRKACNLAIEKLKELAVTIKETKKRQVLEKCAATTLSSKLVAHQKDFFSKMVVDAVCQLDELLPLEMIGIKKVQGGALEESVLVSGVAFKKTFSYAGFEMQPKSYKNPKIALLNIELELKAERDNAEVRVDSVQEYQNIVDAEWNILYEKLRKIHESGAKVVLSKLPIGDVATQYFADRDLFCAGRVQEEDLKRTMKACGGAILTTVADLTDANMGTCADFEEIQIGGERYNIFKGCPNAKTVTFILRGGAEQFIEETERSLHDAIMIVRRALKNDAVVAGGGAIEMELSKHLRDYSRSVAGKEQLLIAAFAKAFEVIPRQLCENAGFDATNILNKLRQKHAQGGVWFGVDVYTEDISDNFAACVWEPALIKTNAIVAATEAACLILSVDETIKSPKAQSETSAARRM??????????????????????????????????????????????????????????????????????????????????????????????????????????????????????????????????????????????????????????????????????????????????????????????????????????????????????????????????????????????????????????????????????????????????????????????????????????????????????????????????????????????????????????????????????????????????????????????????????????????????????????????????????????????????????????????????????????????????????????????????????????NFISS------TGLFSDANGFYLLKENAILSAERLVREALSRKRKMVEIFDDLSNSLCLVADLAEFVRVGHPELRFQQAAEQTSIFISAEVERLNTNLELYVALREVVETGKDVAPTTLDNFVAKLFLFDFEQSGIHLDDDRRKEVVRLNESILHVGSYFVNNTNQPRTVPRSKLPEEVCIVNSLYPESENELLREAAYRIYLFPDDHQENLLQQLLLARKQLASLCGFKSYAHRAVKGSIADTPEVVEFIDCVTEKLMPLALRDYEAMFKLKPWDVAFYTPYFSIGACMDGLNLVFRELYQVQLNVCGEVWHKDVIKLSVSPSDEPLGFIYCDFLERAGKPNQDCHFTIQGGCLKGGVRQLPIVVLMLSLPPSLLNPHMVDNLFHEMGHAMHSMLGQTRYQHVTGTRCSTDLAEVPSILMEYFASDPRVVSRFAKHYVTGEEIPKNLLSSWIKSKKVFSASDTQLQVFYAALDQAYHSEDPLDTTAVLADLQNKYYGLPHVPYTAWQLRFGHLVGYGAKYYSYLVSRAVASSIWEKHFKADPLNFDSGKFYDQVLRHGGGKPAKEIVEGVLLANAIIDDV????????????????????????????????????????????????????????????????????????????????????????????????????????????????????????????????????????????????????????????????????????????????????????????????????????????????????????????????????????????????????????????????????????????????????????????????????????????????????????????????????????????????????????????????????????????????????????????????????????????????????????????????????????????????????????????????????????????????????????????????????????????????????????????????????????????????????????????????????????????????????????????????????????????????????MVLADLGRKITNALRSLGNATIINEDVLNSLLKEVCTALLEADVNIRLVKQLRENVRSVIDFEEMAAGLNKRRMIQMAVFKELVKLVDPQVKAWQPVKGRPNVVMFVGLQGSGKTTTCTKLAYYYQKKGWKTALVCADTFRAGAFDQLKQNATKARIPFYGSYTEVDPVVIAAEGVEKFRNENFEIIIVDTSGRHKQEASLFEEMLAISQAVDPHLVIYVMDASIGQACEAQAKAFKSKVDVGAVIVTKLDGHAKGGGALSAVAATQSPIIFIGTGEHIDDFEPFKVKPFVSKLLGMGDIEGLLDKVNDLKLEENTELIEKLKHGEFTLRDMYEQFTNIMKMGPFNQILSMIPGFGPDLMKGASEQESMARLKRLMTIMDSMSDKELDSSEGEKLFSRQPTRIARVARGAGVTQREVQELLTQYKKFAQVVKKMGGIKGLFKGGDLSKNVNQAQMAKLNQQMAKMMDPRVLQHMGGMQGLQSMMKQLQGA?????????????????????????????????????????????????????????????????????????????????????????????????????????????????????????????????????????????????????????????????????????????????????????????????????????????????????????????????????????????????????????????????????????????????????????????????????????????????????????????????????????????????????????????????????????????????????????????????????????????????????????????????????????????????????????????????????????????????????????????????????????????????????????????????????????????????????????????????????????????????????????????????????????????????????????????????????????????????????????????????????????????????????????????????????????????????????????????????????????????????????????????????????????????????????????????????????????????????????????????????????????????????????????????????????????????????????????????????????????????????????????????????????????????????????????????????????????????????????????????????????????????????????????????????????????????????????????????????????????????????????????????????????????????????????????????????????????????????????????????????????????????????????????????????????????????????????????????????????????????????????????????????????????????????????????????????????????????????????????????????????????????????????????????????????????????????????????????????????????????????????????????????????????????????????????????????????????????????????????????????????????????????????????????????????????????-------MDNDDEDYDLVYSEDSNSEPDVDLENQYYNSKALKEDDPKAALQSFQRVLDLEAGEKGEWGFKALKQMIKINFRLGNYAEMMARYKQLLTYIKNAVTRNYSEKSINSILDYISTSKQMELLQEFYETTLDALKDAKNDRLWFKTNTKLGKLYYDRGEFNKLAKILKQLHQSCQTDDGSDDLKKGTQLLEIYALEIQMYTAQKNNKKLKKLYEQSLHIKSAIPHPLIMGVIRECGGKMHLREGEYEAAHTDFFEAFKSYDESGSPRRTTCLKYLVLANMLMKSGINPFDSQEAKPYKNDPEILAMTNLVSAYQNNDIAEFEKILKNNRRTIMDDPFIKEHIEDLLRNIRTQVLIKLIKPYTRIHIPFISKELNIDEVEVENLLVSCILDNTIQGRIDQVNQVLELNRAGQSGCRYAALDKWTLQLSSLHQTILNKM???????????????????????????????????????????????????????????????????????????????????????????????????????????????????????????????????????????????????????????????????????????????????????????????????????????????????????????????????????????????????????????????????????????????????????????????????????????????????????????????????????????????????????????????????????????????????????????????????????????????????????????????????????????????????????????????????????????????????????????????????????????????????????????????????????????????????????????????????????????????????????????????????????????????????????????????????????????????????????????????????????????????????????????????????????????????????????????????????????????????????????????????????????????????????????????????????????????????????????????????????????????????????????????????????????????????????????????????????????????????????????????????????????????????????????????????????????????????????????????????????????????????????????????????????????????????????????????????????????????????????????????????????????????????????????????????????????????????????????????????????????????????????????????????????????????????????????????????????????????????????????????????????????????????????????????????????????????????????????????????????????????????????????????????????????????????????????????????????????????????????????????????????????????????????????????????????????????????????????????????????????????????????????????????????????????????????????????????????????????????????????????????????????????????????????????????????????????????????????????????????????????????????????????????????????????????????????????????????????????????????????????????????????????????????????????????????????????????????????????????????????????????????????????????????????????????????????????????????????????????????????????????????????????????????????????????????????????????????????????????????????????????????????????????????????????????????????????????????????????????????????????????????????????????????????????????????????????????????????????????????????????????????????????????????????????????????????????????????????????????????????????????????????????????????????????????????????????????????????????????????????????????????????????????????????????????????????????????????????????????????????????????????????????????????RRETKLDIHKVQRNDPALHPLELPREYKRALNAVKLDRMFAKPFIGALGHSDVVQSLMKHPTRLSVMVSGSCDGQIKLWNIPTGKCIRTIQASNSD-EELQDDSSNIKTVIANIDAHYKPLLMTCGERVDVWEERSEPLVSYW-NIDTIYVKFNPVEASGSDRSLILYDIRQKEPLRKVILALRTNALCWNPMEGMHFTAANEDYDCYTFDMRKLSEPLIIHKDHVQAVIDLDYAPTGKEFVTGSYDKTIRIFHSREVYCTKRMQRLTSVIWSLDNKYVVSASDEMDIRVWKSNASEKLGPKKYQDKLKEKFAHHPEIRRIANNRHVPKSVYKEHKIIERSQLRK--MTLLAKGQYGDALSHFHAAVEGDPKNYLTYFKRATVYLAIGKHRSALDDLNEAVNLKPDFHAARLQRGSALLKSGRLDEAHIDLEAVLRDDPTNQEASIEPIKYEAVDLLTKLWDVKLRELRSQSFEKLGDYVNAISDLRASTKMRADDTDGFLKLSKLHYELGEADESLSTIRECLRLDPDHKECYKKVKKLANAMTEDCVDKIVSQIKAKCHCLNKGKDGIKICTEAIQASPIYCDRADLYIHNQLYESDYRKAAQLDERAKEGIQRAGKLLKQSQRRDYYKILATKKEIMKAYRKLAQKWHPDNFASEDETQRKLAEKKFIDIASAKEVLTDEEKRQKFDMGEDPLDPEAQ-------?????????????????????????????????????????????????????????????????????????????????????????????????????????????????????????????????????????????????????????????????????????????????????????????????????????????????????????????????????????????????????????????????????????????????????????????????????????????????????????????????????????????????????????????????????????????????????????????????????????????????????????????????????????????????????????????????????????????????????????????????????????????????????????????????????????????????????????????????????KNVWPKERPHIRRRVLIALGLLVLAKVANVQVPFLFKYAVDYLNEKTGILIGYGIARAGASLMNELRNAIFAKVAHDSIRRVATSVFLHLHNLDLSFHLGRQTGALSKAIDRGTRGIQFVLSALVFNVVPTIFEVGLVSTILYIKSGEYALLTLGCIGTVITTQWRTKFRIAMNKAENEAGTQAIDSLINYETVKYFGNEKYELRKFEDASLKTTTSLAFLNFGQNAIFSAAIMIMATQMTVGDLVLVNGLLFQLSLPLNFLGSVYREVRQSLIDMQTMFSLLRVSSKPLLATPKVKFNDVHFSPILRGLSFEVPAGKKVALVGGSGSGKSTVVRLLYRFFDPKNGSVSIAGKDIRDVSVRQSIAVVPQDAVLFHNTILYNIRYGNLKAEAIEASRMAELHDSIERWYETQVGERGLKLSGGEKQRVAIARAILKNSPILVFDEATSSLDSITEHKIMNALKASQNRTSICIAHRLSTVVDADLIYVLKDGRVLEEGHLSLVQSFYAYLWTRQHQSILRRPAHAAALNQREVVVNVPETLVTEIENGIRVATEDSGLQTATVGVWIDAGSRWETTENNGVAHFLEHMAFKGTQKRTQTDLELEVENMGAHLNAYTSREQTVYYAKCLSEDIPKAIEILADIIQNSKLGEAEIERERGVILREMQEVETNLQEVVFDHLHSVAYQGTPLGLTILGPTENIKKINRQDLLDYINTHYKAPRM-------------------HFGKVPALYQGTKHCRFTGSDIRIRDDDMPFVHCAIAVEGCGWENPDNIPLMVANTLIGSWDRSMGGGTNVSNYLALVAGNQQLAYSFQSFNTCYKDTGLWGVYFVSDKMKCHEFVWHLQKMWMTLCTDVKEAEVERAKNLLKTNMLLQLDGSTPICEDIGRQMLCYGRRIPLPELIARIDAVSVDTLRDVXVKISVDKCSVTGAGSPFLSL-----------????????????????????????????????????????????????????????????????????????????????????????????????????????????????????????????????????????????????????????????????????????????????????????????????????????????????????????????????????????????????????????????????????????????????????????????????????????????????????????????????????????????????????????????????????????????????????????????????????????????????????VIGSSSEFRIDVNKQEYLEELGINVKAKNFLVYQGAVESIAMKNPREITALFEEISHSMDRSDEELYVKKKGIAAEKKEAQGEEAKKYQQLKEDLQVELQLFKLYHIQEEMEKKKKKEKIESDIKEKKQIDRNIRDELNLNKKRPSYIKAKENASHIEKKLETAKKSLNQAKKAHQNHEKIEELEEELDRVKKEFIAEDLELQEEQRREYDRLKDEAARTSAKYLKDLDSLEREQKADGDKLENETRRQNEIEAKIRERQENIKRIEKLKDLKELEQKEHEIDAKERITKELESLSQLGDAKVDRHEQERRKKKEEVVDHLKKLYPGIYGRMINLCKPIHSRYNMAVTKVMGKSMEAIVVDTEKTARQCIQYLKDQMLEPETFLPLDYITCKEVIERLRSIQHPRNVKLVYDCLKYDPPAIKRAVLHATNNALVCETPEDASIVAFDLGDGRRYDAVALDGTFYQKCGFISGGSAELERRARRWDEKDIYKLKISKEKLTEELKEAIKKTRKESDLMVMQSQLNGLKTRLKYCTIDEIKSRMAKREERINEIRDSMNNVEDQIFADFCRQLGVQNIRQYEERQSKDRQEKARRLQYENEIHSLQSRKEYEK--SKNTLEVKEEEKNLEQARETERREMKSIEEEMNKVEEYKNQKIEKKTQCDRIEDEISEIKRGLSGIQKEISTIQKALMQIECRLESKKADRHAILLHCKMECIDLPLEDGSLQKSYQADNIRPDFGILEMQSEINMRKIQAPNMRALERLEGVKDKLKETDNELNNLRKTAKTNFERVKRQRECFESVAQKVDSIYKSLTNNASAQAFLVPENPEEPYLEGINYNCVAPGKRFQPMSNLSGGEKTVAALALLFAIHSFKPAPFFVLDEIDAALDNTNIGKVARFIREKTESQFQCIVISLKEEFYGHADALIGVAPDPGDCTISRVFTVDLSVPDLVLKLLKKAITERNVFEIQNLYENTFVTLTEKHFLILYKE--LYYRHIYAENRFESYYNYCHLFNYILSAAPVPLELPNQWLWDIIDEFIYQF--QSFSLFVWNVHSVLNVLHSLVDKSNINEQLKEVNANRDPYAVTGVFGRQPLYQTLGYYALIGLLRLHSLLGDYYQAIKVLENIDLNRT--ARILACQMTTFYYVGFAYMMMRRYSDAIRTFSNMLVYLQRTRRTLQAEMIEKQTDQMYRLLTICLVLQPQRIDESVASVLQEKNMIKLQRGDLDEFQNCPKFVSPVQLEVFMKEVTQHLTIRSYLKLYTTMPISKLAAFMLLCFKHKMQNLVLEGEFQTGSDVDFYIDKDMI---------------------------------------------------------MGPPDPILGVTEAYKRDTNPKKINLGVGAYRDDNGKPYVLPSVLMAEEQMMAAKLDKEYAPISGSPEFCAEAAKLAFGSDSDVIKEGLNATVQGISGTGSLMIGGIFLQAFWPHNKEIYLPTPTWGNHIPLFKRSGFAVKQYRYYDPKTCGFDFDGALQDLAKMPEKSVVLLHACAHNPTGVDPKPEQWQEMSKVIKSRNLLPFFDMAYQGFASGDIDRDAQALRLFVREGHRVLLAQSFAKNMGLYGERCGAFSLIASSKEEQAKVLSQLKILIRPTYSNPPIHGARIAQRILSTPELRKQWLVDVKGMADRIISMRTMLRKGLEKEGSSRNWQHVTDQIGMFAFTGMNAEQVERIIKEFSVYLTKDGRISMAGVTSKNVEYLAHAMHQVTKQLTSRQKHILHCCLYCCVILGFEYASDDPEALDPFEAYGTFMALFLYFLRFLALLSLPQVICNALGLTLYQAFPSKVEAKGSPLVAPFICIRTVTRGMFPDLVKQNVSRNMNTCLAVGLENFVIEIATDRPLNIQKHPRIREVVVPPEYKTKNGSLFKARALQYCLEEDVNILGDDDWIVHLDEETILTENAVRGILNFVYSKKHEFGQGLITYANEEVVNWLTTLADTCRVADDMGKLRFQFYMFHRPLFSWKGSYVVSKFKAERDVSYDHGPDGSVAEDCYFSMIAYKKGYTFDFIEGELWEKSPFTIKDFLQQRKRWLQGIFLVVHSDKIPIQNKIFLAMSLYAWMTMPLATLSLFLSTFYPLPPTAWCNFLSAFVGATWIYMYIFGVFKSFSLMRLGVKRFIICIIGALCAIPFNIVIENVAVIWGLFGSKHKFYVVQKQLV--------------------------------------------------PLEFI---NYNFTGNLRPWPQTPMRKVPDTIERPDYADHPDGRSKCEEQVKGSGVIKVLDEEEIEGVRLASKLAREVLDEAYKAAGVGVTTDEIDRIVHEACIDRECYPSPLNYYNFPKSCCTSVNEVVCHGIPDTRPLQDGDLLNVDITVFHRGFHGDLNETFFVGNVDAESKRLVKATWECLQKAIAIVKPGEKYREIGNVIEKHARANGFSVVRTYCGHGIHRLFHTTPNVPHYAKNKAVGVMKPGHVFTIEPMINMGNWRDTTWPDDWTAVTLDGRRSAQFEQTLLVTETGVDILTRRRDKNGQPYFMDQSDVMTALRRLSKKRRELLVKQFGTGTFENLAKLLGNGMEP---DTDESEIDTLLTYTDSSTFLKGTQSANPHNDYCQYFVDTGQRPQNFIRDVGLQDRFEEYPKLKELIRLKDELISRTATPPMYLKCDLRNFNLSELGSKFDVILVEPPLEEYQRTAGVINMDLYAWDDIINLPIETVAAQRSFIFLWCGSSDGLDLGRQCLKKWGFRRCEDICWIKTNLKHGHSKNLEPKAIFQRTKEHCLMGIKGTVRRSTDGDFIHANVDIDLIITEEPEYGCIDKPEEIFHIIEHFCLGRRRLHLFGRDSTIRPGWLTVGPALTNSNFHAETYANYFTTPNGHLTGCTDRIEALRPKSPPPKARQGLARHGILPKNLSKKLSLDKVDVKDKRVLIRVDFNVPMKDGKITNNQRITAALPTIQHCLKNGAKAVILMSHLGRPDGQKMEKFSLRPVAVEVGKLLNTNVSFLDDCVGQAVEQACANPSSGSVILLENLRFHVEEEGKGVGPNGEKLKASDSDVAKFRESLTKLGDVYVNDAFGTAHRAHSSMVGVNLPQKAAGFLMKKELEYFAKALENPEKPFLAILGGAKVKDKIQLIENLLDKVNEMIIGGGMAFTFLKVLNNMKIGKSLFDEEGAGIVQKLMDKAKSKNVKVHLPVDFVTGDKFAEDATPGYANVEQGIPDDQMGLDIGPKSVEAFAAVVQRAKTVVWNGPPGVFEFDKFANGTKGMMNAVVAATKSGATTIIGGGDTATCCAKYNTEDKVSHVSTGGGASLELLEGKVLPGVAALTDV------------------------------------------------VTNEERKKITALGLGPHGLATNITLLKASEVDEIFSGFDEKYKALSVSTEPTVPREPKQRKQNWNISMPNSSHHLDAVPCSTPVYRSRLAHKKVRTFPLLYDDLNPEQLQENANQPEVLIPIRLDMEIEGHKLRDTFTWNKNEQIITPEQFAEVLCDDLDLPAASFVPAIAQSIRTQIEAFPTDNILEEQTDQRVVLKLNIHVGNISLVDQFEWDMSEKDNSPEGFALKLCQELGLGGEFVTAIAYSIRGQLSWHQRTYAFSEAPLPTVEVPFRNPSDADQWCPFLETLTDAEMEKKIRDQDRNTRRMRRLANTAW????????????????????????????????????????????????????????????????????????????????????????????????????????????????????????????????????????????????????????????????????????????????????????????????????????????????????????????????????????????????????????????????????????????????????????????????????????????????????????????????????????ISDLDRQIEQLRRCETIKESEVKALCAKAREILVEESNVQRVDAPVTVCGDIHGQFYDLKELFKVGGDVPETNYLFLGDFVDRGFYSVETFLLLLALKVRYPDRITLIRGNHESRQITQVYGFYDECLRKYGSITVWRYCTEIFDYLSLSAIIDGKIFCVHGGLSPSIQTLDQIRVIDRKQEVPHDGPMCDLLWSDPEDTQGWGVSPRGAGYLFGSDVVQQFNATNNIDMICRAHQLVMEGFKWHFNETVLTVWSAPNYCYRCGNVAAILELDENLQRDFTIFEAAPQEARGIPCKKQPYFLMKPAE--SMADPDLQTIEDVKEQARQIEKSVNTKEPRFMLRVLRSLVTTRKKLNARVLRKVVSGFYTHSPQQRDELLAFIEPMDTDAASPSKTEKLPLTPEVDIYLHLLVLLYLVDLSKKQEAVKCADLLMKKVEGQNRRTLDLLAAKCYFFYMRAYELTNQLNKIKSILHQRLRTATLRNDHEGEAVLLNCLLRIYLHYNLYDQAAKLVSRSTFPDTASNNESARHLYYLGRIKAIQLEYSEAHKNLLQAIRKAPQNTAIGFKQTVHKLAVTVELLLGDIPDRSLFRQAHLRRPLFPYFQLTQAVRAGNLAQFAQVLLRYGPQFQQDHTYTLIIRLRHNVIKTGIRMLNASYSRISLNDVARKLALDSAEDAEFIASKAIKDGVIEAMIDHDQGYLQSKDSIDIYSTGEPLSAFHERITFCLDLYNSSVKAMRFPPKSYSKELESAEERREREQQDLEYAKEDDDDEF??????????????????????????????????????????????????????????????????????????????????????????????????????????????????????????????????????????????????????????????????????????????????????????????????????????????????????????????????????????????????????????????????????????????????????????????????????????????????????????????????????????????????????????????????????????????????????????????????????????????????????????????????????????????????????????????????????????????????????????????????????????????????????????????????????????????????????????????????????????????????????????????????????????????????????????????????????????????????KVKVKWKETYNDVELNTVTLFKEILFSLTGVQPDRQKLLLKGKEIKND-WGMMLLMMGTPDPLPQPAEKPRFLEDMDESEVASALDLPAGLNNLGNTCYINATVQCLKTVPELRELNAFTGALIAKLRDLYRNMDNTEIAAFFMLSAIHAAFPRFAEKGENGQQDANECWTELLRVLQSVIDQYFGGTFVEMRCDEAPDESTENFLQLSCFIS-QEVKYLQSGLKLRL-QETLTKRSPTLNRDAKYKTSKICRLPAYLTVQFVRFFYKERESVSAKILKDIKFPLVLDVFELCSESLQQKLLPMRAKFKELEDLYQFPDDGSNNSGYYSLQAVLTHKGRSSSSGHYVAWIRREWFKCDDDVIVSEEEITKLSGGGDWHTAYVLLYGPRQLEALINKKLPKELLLRIFSYLDVVSLCRSAQVSKAWNVLALDGSNWQRVDLFNFQTDIEGPVVENISRRCGGFLKKLSLRGCRSVTDASLKTFAQNCNNIEDLLNDCKKLTDSTCQYLSLHLNVGSCSEITDLSLKALGEGCRNLEEINISWCEQITKEGVEHLAKGCPKLRAFIAKMCINDDAVKVLTWNLEVINLYGCSTITDEATISISQNCPRLCYLSVSNCPHLTDMTLNSLSQGCLKTLEVAGCSQFTDAGFQALARNCRLLENLDLEECVLITDNTLAYLAAGCPNLRKLSLSHCELITDDGIRQIGLSVLELDNCPLITDSSLEHLISCLQRIELYDCQLITRAGIRRLRPDLKVHAYFAPVTPRQRYCRCCVIL???????????????????????????????????????????????????????????????????????????????????????????????????????????????????????????????????????????????????????????????????????????????????????????????????????????????????????????????????????????????????????????????????????????????????????????????????????????????????????????????????????????????????????????????????????????????????????????????????????????????????????????????????????????????????????????????????????????????????????????????????????????????????????????????????????????????????????????????????????????????????????????????????????????????????????????????????????????????????????????????????????????????????????????????????????????????????????????????????????????????????????????????????????????????????????????????????????????????????????????????????????????????????????????????????????????????????????????????????????????????????????????????????????????????????????????????????????????????????????????????????????????????????????????????????????????????????????????????????????????????????????????????????????????????????????????????????????????????????????????????????????????????????????????????????????????????????????????????????????????????????????????????????????????????????????????????????????????????????????????????????????????????????????????????????????????????????????????????????????????????????????????????????????????????????????????????????????????????????????????????????????????????????????????????????????????????????????????????????????????????????????????????????????????????????????????????????????????????????????????????????????????????????????????????????????????????????????????????????????????????????????????????????????????????????????????????????????????????????????????????????????????????????????????????????????????????????????????????????????????????????????????????????????????????????????????????????????????????????????????????????????????????????????????????????????????????????????????????????????????????????????????????????????????????????????????????????????????????????????????????????????????????????????????????????????????????????????????????????????????????????????????????????????MANTLEQLRKMTIVVADTGDFESMKRFKPTDATTNPSLILQAAKLPQYQKLIDEAVEFGKNNGNDLEATMDMLFVLFGCEILKIIPGRVSTEVDARLSFDVQGSVNKALNIIKLYEKRGISKDRILIKLATTWEGIKAAEILERDHGIHCNMTLLFNFAQAVAAADAGVTLISPFVGRIYDWFVKSTGVKVYEGYDDPGVKSVTRIYNYYKKYGYKTVVMGASFRNTSEIKCLAGCDLLTISPSLLEQLNETGGNLTQALSVEKAKAAELERRTFDEKTFRWELNEDEMATDKLSEGIRKFAIDARKLEDLIRSKLN??????????????????????????????????????????????????????????????????????????????????????????????????????????????????????????????????????????????????????????????????????????????????????????????????????????????????????????????????????????????????????????????????????????????????????????????????????????????????????????????????????????????????????????????????????????????????????????-----------------------------------MDTSHVALVSVKLKAEGFDMYRCDRNQVLGTNLNSLSKVLKCANNDDTITLRAQEDGDTLTMIFEAKDESELHEYEVKLMNLDTEHLNIPVTSYQVTAKLPSAKFQRICRDLSQIGDSVTISCAKDGIRFSTQGDIGSCTIRLKQTTDPDKPEEAVSIKMTEPIIQSFALKYLDKFAKATPLSSHVLLCMSPDIPLTVDYIIGSIRYFLAPKIDDDD------------------RLFSTGKADLNKVAVLGASGGIGQPLSLLLKQNKLVSHLSLYDVANTYGVAADLSHMNTRARVTGHLGPDQLDEALQGCDVVIIPAGVPRKPGMTRDDLFNTNASIVRDLTDACARNCPNALVGIISNPVNSTVPIASKVYEKRGV-DASRIFGVTTLDVVRANAFVAELKGLDPEQVNVPVIGGHAGITIIPLISRATPSVSFPQDQLDALTKRIQDAGTEVVKAKAGTGSATLSMAFAGARFAISLLEAINGKNVVECAYVRSNVTEATFFSTPITLGKNGIAQNHGLGKLSPYEEELVKAAIPELKASIKKGEDFVK-????????????????????????????????????????????????????????????????????????????????????????????????????????????????????????????????????????????????????????????????????????????????????????????????????????????????????????VP-FIDLFASIGAEISDLRDIINVCDSCFKDVESVL--NSIVS-LLIAFCQKLNA-ADPKVCIR--VLQNLYEGIKYEVYLALVEVAGDQIHLVFNDINKLKSTFGVDRIQRLLRQLHRVLSELASKVMIELLSTYTEEHASHAKDDAQRCIVSFIADPGTFLMDHLLTLKPVKYLEGEKIHDLLTIFVSDKLSSYINFYNANKNFV-DSLGLNHEQNLQKMRLLTFMQMAETKKEIPFQAIQEELQIESDQVEEFVIEVLRTKLVRAKVDQVNKRVLVTSTMHRTFGRPQWEKLRDTLNLAHVQRTVHT--------------------------EYEERSMIEDQPSFQELVKILVNWINDELNDQRII--VKDLEEDLFDGQILGKLVEKLSGIKLDVVEVTQNEEGQKHKLRTVLDAVNKLLGVKWSVEGIHGKNLVQIIHLLVALIRHYRAPI--RLPQNVSVNLVVVQKR-EQLTESYDELGMRVEPRDAFDTLFDHAPDKLQVVKRSLVTFVNRNLNRINIEELDPNQFSDGLLLCFLMGMLEGYFVPLGLFTTP-TDPIEDVTGKETALKPENYINTSKLHNVNIAFELMEEAGI---------------------------??????????????????????????????????????????????????????????????????????????????????????????????????????????????????????????????????????????????????????????????????????????????????????????????????????????????????????????????????????????????????????????????????????????????????????????????????????????????????????????????????????????????????????????????????????????????????????????????????????????????????????????????????????????????????????????????????????????????????????????????????????????????????????????????????????????????????MNEIFRKPFISLCASFRI----AVIVDRFHELLRFVPHISQFLRRQGVNFKIYVINQVDSLRFNRASLINVGFLLSRHDSDYIAMHDVDLLPLNDKLSYKFPTDGPFHVASPDLHPKYHYKTFVGGILLLTREHFELVDGLSNKYWGWGLEDDEFYVRLKEAGLTIKRPQGINTGPDNTFLHIHDRNHRKRDTAKILNQKQETRKRDRETGLSTVQFEIDSEHELKVDGYVCKVINVKLICDYKLTPWCDFKN???????????????????????????????????????????????????????????????????????????????????????????????????????????????????????????????????????????????????????????????????????????????????????????????????????????????????????????????????????????????????????????????????????????????????????????????????????????????????????????????????????????????????????????????????????????????????????????????????????????????????????????????????????????????????????????????????????????????????????????????????????????????????????????????????????????????????????????????????????????????????????????????????????????????????????????????????????????????????????????????????????????????????????????????????????????????????????????????????????????????????????????????????????????????????????????????????????????????????????????????????????????????????????????????????MDPESFLEMANAVTKLKMYPWFEVAHCIISCLYIREDLGQGAHAFSRKHPFALWVSCMTSIFAGGILANLLLGEPILGVLKNNNSILLATGVWYLIFYSPLDLCYKLCKFFPVKLVVAAMKEVTRCKKVHDGVVHAAKIYPNGYLIMVIVGTVKGNGAAFLKILERILRGSWTPNAIEFMVPTFPTKASILASVIFIVEKKTELITAPHAIVYLGIVICFIYFKLSSMLLAISDPFLPFENVFCALFMGGLWDALANMGKRQNGGDGNNPKIDLSRNG-----KKKDMDQENKLIRRRNV-----DKSNSGARNDSDDFDSKETRLTLMEEVLLLGLKDKEGYTSFWNDCISTGLRGCILVELALRGRIELEKAGMRRRSLLLRKVLLKNGHPTGDVLLDEALKHIKDTQPPETLQSWIDYLSGETWNPLKLKYQLKNVRERLAKNLVEKGVLTTEKQNFLLFDMTTHPLVDSASKNKLIKKVQDAVLGKWVNDPHRMNKRVLALILLAHASDVLENAFAPLSDDDYEVAMKRVRELLDLDMEAESLKPGTNETMWGVFAAFVK??????????????????????????????????????????????????????????????????????????????????????????????????????????????????????????????????????????????????????????????????????????????????????????????????????????????????????LTEWFTPVLKESKFRETGMLTPEEFVIAGDHLVHHCPTWVWCAG-DKDYLPSDKQYLSTRRVPCHKRCREMEEKIIDPE-GGWVDTHTQDDDDEEPEDMDDFIDEVDPTINIVATRTYDLNITYDNYYRTPRLWLSGYNENQQPLTIEEMYQDISQDHAKKTVTMESHPHIPGPHMASVHPCRHAEVMKKIIETMEENGRLQVHQYLIVFLKFVQAVIPTIEYDYTQNFSM??????????????????????????????????????????????????????????????????????????????????????????????????????????????????????????????????????????????????????????????????????????????????????????????????????????????????????????????????????????????????????????????????????????????????????????????????????????????????????????????????????????????????????????????????????????????????????????????????????????????????????????????????????????????????????????????????????????????????????????????????????????????????????????????????????????????????????????????????????????????????????????????????????????????????????????????????????????????????????????????????????????????????????????????????????????????????????????????????????????????????????????????????????????????????????????????????????????????????????????????????????????????????????????????????????????????????????????????????????????????????????????????????????????????????SILVVMHYKYQDRSKRDINNAINHYKNLIEKFTFANGTTRDLVCLDGTIPVRYRYNIPVCIYLLDNYPYSAPMCYVRPTHDMTIKQSKHVDASGRIYLPYLSDWKDTT-SDLLGVIQVMIIVFGELPPVYSKPPP-YP--GTGTITEEHIRASLLTAVEDKLKSRLREQKQAEIDVLKRTSDELSKGKQRIEDLISKMEKDVSVQEDKERQLTDLNIDESFGPTMPLYKQLLDAFAEENAIVDAIYYLGEGLRKGSIDLEVFLKHTRELSRRQFFLRALMQRCREKAGLT???????????????????????????????????????????????????????????????????????????????????????????????????????????????????????????????????????????????????????????????????????????????????????????????????????????????????????????????????????????????????????????????????????????????????????????????????????????????????????????????????????????????????????????????????????????????????????????????????????????????????????????????????????????????????????????????????????????????????????????????????????????????????????????????????????????????????????????????????????????????????????????????????????????????????????????????????????????????????????????????????????????????????????????????????????????????????????????????????????????????KTDDLLLDPAIRVWVFLPIVVITFFVGILRHYVSILISSQKKIELQQVQDSQALIRARLLRENGKYIPKQSFLMRRHFFNNDENGFFKIQQKRPSTQPNPMTDPSMMTDMLKGNVTNVLPMILIGGWINWTFSGFVTTRVPFPLTIRFKPMLQRGIELMSLNASWVSSASWYFLNVFGLRSIYALVLGENNAADQTRAMQEQMSGAAQAVPADPKQAFKAEWEALEIWDHQWALRGVDMHLCGLPPNNSSGDE--------------AGRALQIQRRREKEREDLEIRKKKIEEETRIGKIDNKFATHFDAIEQQLKSSTIGLVTLEEMRAKQEDVVKEREKQLAQKQSEEQRRAEEKRKLEKERQKRQIATLSFKFDDEEEEDEDEEDEKEKSSDDNSQSSFLENDESNEASCNGTVKSNDEDSRSSIDSQTGKKLKITKNPEVDTSFLPDREREEEEKRLREQLRQEWQEKQERIKNEEVDITFSYWDGSGHRRSVRMKKRNTIYDFLLKCLELLKKEFHELRAVTADQLMYVKEDLIIPHHYSFYDFIVTKARGKSGPLFSFDVHDDIRLLHDATVEKDESHAGKVLLRSWYERNKHIFPASRWEPYDPTKNYSKYTISDKGKKMARKRSERSLRPLPEYVLRAMKRRQRKRKKALLTRVKTRK-RKLLKAEYINRARKYEAEYERQKKAIIQNHRMARKHDNFYVPPEPKLALVIRIRGINGVPPKPRKVLQLFRLRQINNATFVKLNKATLNMLRIAEPYIAWGYPSLKTVRRLIYKRGFLKVNGRRVPLVTNETIQRHLGRQKIICLEDIVHEIFTVGPSFKRVNRFLWHFKLSNPRGGWRNKTRHYVEG----------------VITRTTAEQLWKGVTSVSNAGKKRGRGRGAGRKAIKDLNRGQTIGVGRINMLFPGLNAPIIRGRETLSQRSLPPDPEFDKKLIEIREKQSAFRVREMHDLDRGFSGAFMPGRWIGPPDPVNGEPFEGFDTKALKVKQHNIMCP-LGRRRVCDVVVAVGNKNGVAGFSEVTGKDQRQSLRKARSQAGQRLVHIPLFEGTVIHDFYSKYHCTELFVYKRPPNFGVEGHKVIRALCEVIGIKDLTVVTEGEPRNVLAMTKAFFLGLIKQKTFQEMADEKRLHVVEFDYDKDNYPVIVASPSDVRQKHEIGSEEVLDFQMYLHNGHVLDNPPPRPPPYIGTHGWEFFIKMQSTYKARARSRVQLIAQYGELKSHLNVWEAELRQKHRLSDEVGEWKNKQRLLIVAARGITARDRHLLLNLAESMPHSKREPKVE--KNDLNEICEMRNCNKCIYFETMKKRDTYMWISSVPNGPSIRFLVENIHTMEELRMTGNCLKASRPILSFDQHFENSPLMKEVFTQTFGTPQFHPKSQPFFDHVFSFSWLDNRVWFRNYQIVDEEKGTLAEIGPRFVLNPIKILEGSFRGEVLWSNPHYVTPSQQRAMAKLAAQQKYK-------------MAVGKNKG-SQKGKKGGKKKVADPFARKEWYDVKAPATFSVRQVGKTLVNRTQGTRIASDSLKGRVFESSLADLNNDEIAYRKFRLIAEEVQGRIILTNFYGMDLTTDKLRSMVKKWQTLIEAVVDVRTTDNYLLRLFCIGFTRKSPNQVKKTCYAQTTQVRAIRKKMTDIILREVGRSDLREVVTKLIPDAIGKDIEKACQSIYPMHDVMIRKVKVLKRPKFDLGKLLEMHSDGKGKAAAGTKKVDRPDGYEPPVQQAV????????????????????????????????????????????????????????????????????????????????????????????????????????????????????????????????????????????????????????????????????????????????????????????????????????????????????????????????????--------------------------MKVLRHEEFKEGCQAACNGPYDGQWSKTMIGYGAEDKHFVMELTYNYGIGSYKKGDDFSSITIADSEILKRV----------NGQTVLFSPDQYQFNIAGSEQKNSSDPVQKVTLASSNLERTIEYWNEMLQMPIVERHEKSVQFAYAEDQARLEFINGGKEIDHGKAYGRIAFSCPTSHLKPIEAKMKSSNQTILTPYTELDTPGKATVAVVILADPDGYEICFVGDEAFSALSQVDPKADDLLNKAIEEDKSDDWYAKKNKQKPAA??????????????????????????????????????????????????????????????????????????????????????????????????????????????????????????????????????????????????????????????????????????????????????????????????????????????????????????????????????????????????????????????????????????????????????TITRSMTGVLKHKAGLSIRNSSTIHQTLIPMVVEQTGRGERAYDIYSRLLKERIICLMGPINDDVSSLVVAQLLFLQSESSKKPIHMYINSPGGVVTAGLGIYDTMQYILPPVATWCVGQAASMASLLLAAGAPGMRHSLPHSRIMLHQPLGSASGQATDIRIHAEEILFIKSLINGLYAKHTKQPVEVIENALERDHFMRPDQAKDFGIIDQVLEHPPVQPTSNNNMRLDKPTGSWLLLLPFWSIGLATPAGHLPDLALFTAGAVLMRGAGCTINDMWDKDFDRHVERTKNRPLAAGHLTMQDAWFFLGGQGLALMVLLQFDWNSILLGSGSLLLVSTYPLFKRFTYWPQLILGMTFNWGALLGYSVTVLPLYTAGIFWTLIYDTIYAHQDKSDDLMIGLKSTAIKFGDSTKTWLSAFSVMVSNLQSWPYFLALSLDINTLGSILGIFGSAFFS?????????????????????????????????????????????????????????????????????????????????????????????????????????????????????????????????????????????????????????????????????????????????????????????????????????????????????????????????????????????????????????????????????????????VQILEALSDNYMYLLIHREACVVDPVEPGKVVDAINVALKAVLTTHHHWDHAGGNQELAVYGGDDRVQALTQEGQ-QINVGSMITCMKTPCHTSGHICYFVVFTGDTLFQAGCGRFFEGTGEQMYQALLGSLPSHTLVYCGHEYTVNNLKYAKHVEPNNAVGERMAWAKGQPTVPSTIAEEKTFNPFMRVSVKKHTDPIEVMTFLRKEKDHFRMNRLFGRGKPKEPPPNLTDVIANVDSRAESVDKKIARLDQDLVKYKDQMKKMRDGPAKNAIKQKALRVLKQRKMYETQRENLLQQSFNMEQTNFATQMLKDTHTTVAAMRTGVKEMKQAYKKVNIESIEDLQDELEDMLDQANEVQDVLGRSYGCPDVDEEELEAELEALGDELAADDTSYLDEVAAPTAPTKEPGAESV-AEGQ-VDEFGLPKIPA????????????????????????????????????????????????????????????????????????????????????????????????????????????????????????????????????????????????????????????????????????????????????????????????????????????????????????????????????????????????????????????????????????????????????????????????????????????????????????????????????????????????????????????????????????????????????????????????????????????????????????????????????????????????????????????????????????????????????????????????????????????????????????????????????????????????????????????????????????????????????????????????????????????????????????????????????????????????????????????????????????????????????????????????????????????????????????????????????????????????????????????????????????????????????????????????????????????????????????????????????????????????????????????????????????????????????????????????????MELHIILWATYLLFLVGDSWAYYITIDAHDENCFFDKVTTGTKMGLTFEVIEGGFLDIDVKITGPDQKVIHEEVRASSGKYTFAAHMDGVYTYCFGNKMSTMTPKVVMFSMDVGDAPAPDAAHDGDANHNKLEEMIKELSHALTAVKHEQEYMAVRDRIHRSINESTNSRVVLWAFFEALVLVAMTLGQVYYLKRFFEVRRVV?????????????????????????????????????????????????????????????????????????????????????????????????????????????????????????????????????????????????????????????????????????????????????????????????????????????????????MADSSSD-VIDHFAMNKTDAVLMVTRFCTVLFTFMYLIPSPYACYQKALIANAATSALRLHQRLPRFQMTREFIALVLLEDSCHYLLYSIIF-LPIT-LSLLPIALFAFLHLVTLFDKAGSIVE---NQRSILQTISLCEIFLMPCIVLGIFSGVSLFAPFLYYRFVYLRYASRRNPYTRQMFHELRIATESLIYPSCPQIIRGISLMEGLAP????????????????????????????????????????????????????????????????????????????????????????????????????????????????????????????????????????????????????????????????????????????????????????????????????????????????????????????????????????????????????????????????????????????????????????????????????????????????????????????????????????????????????????????????????????????????????????????????????????????????????????????????????????????????????????????????????????????????????????????????????????????????????????????????????????????????????????????????????????????????????????????????????????????????????????????????????????????????????????????????????????????????????????????????????????????????????????????????????????????????????????????????????????????????????????????????????????????????????????????????????SEDGEDLFGPELDRYDQAEAEMRRRDRG-MRRGLEEIADLENTRGMSIKEWIAQGPRKEIHHRFRSFLLSFKERIRQMEENKSSFEVNYHLAAQEYVLAYFLAEAPQQMLEIFNEAAKEVVLSMYKNYDRIAKEIFVRITDLPLIEDIRALRITHLNGLIRTHGVVTSTTPVLPQLSLIKYDCQKCGFLLGPFVQHQEVKPGSCPECQSLGPFALNMEETIYQNYQRIYVQESPGQVNAGRIPRSKEAILLGDLCDSCRPGDEIELIGVYTNTYDGSLNIAHGFPVFSTVIMANHIRKKDTDEDIKQITKLSKDIIIDRIVASIAPSIYGHKNIKRAIACALFGGESKDPGQKHRVRGDINVLLCGDPGTAKSQFMKYISKIAPRAIFTTGQGASAVGLTAFVSPVTKEWTLEAGALVLADKGICLIDEFDKMSDQDRTSIHEAMEQQSISISKAGIVATLQARCAVIAAANPIGGRYDTFNDNVDLTEPIISRFDIIQVVRDEKLARFVVRSHMKHHPIPQDLLRKYITFAKQRVHPKLDREKIAKLYAELRRESLRTGSIPITVRHVESIIRCSEALAKMQLHDYVDVNIAIRIILESFITQKFSVMK??????????????????????????????????????????????????????????????????????????????????????????????????????????????????????????????????????????????????????????????????????????????????????????????????????????????????????????????????????????????????????????????????????????????????????????????????????????????????????????????????????????????????????????????????????????????????????????????????????????????????????????????????????????????????????????????????????????????????????????????????????????????????????????????????????????????????????????????????????????????????????????????????????????????????????????????????????????????????????????????????????????????????????????????????????????????????????????????????????????????????????????????????????????????????????????????????????????????????????????????????????????????????????????????????????????????????????????????????????????????????????????????????????????????????????????????????????????????????????????????????????????????????????????????????????????????????????????????????????SRWKVTAFAVLVTNAQEDVLESEAGVNLDADIGLSREGSRTDDETLAREEEAIKLDGLSVKQMKEIRDRAEKYAFQAEVSRMMKLIINSLYRNKEIFLRELISNASDALDKIRVYSLTDRSALDTKSELEIKIKADKDSHMLHITDSGIGMTKEELVKNLGTIAKSGTAEFLQKVSEASELQNLNDLIGQFGVGFYSAFLVADRVIVTSKSNDDPVQHIWESDSAQFSIVEDPRGDTLKRGTQVSLVLKEEARDFLEVDTLRNLIQKYSQFINFPIYLWSSKTEIVDEPIEDE----EEEKKDEVDEEAKVEEAKEEKKPKTKRVEKTVWDWELINTAKPIWQRKPDDITDDEYNEFYRALTKDHQDPLTRIHFTAEGEVTFKSLLYVPTLQPSESFNRYGTRQDHIKLYVRRVFITDDFQEMMPSYLSFIRGVVDSDDLPLNVSRETLQQHKLLKVIRKKLVRKALDMIKKIPADKYDQFWKEYSTNMKLGIIEDATNRQRLAKLLRFHTSSLSPDKWTSLNDYVKRMKPKQEYIYYVAGASYDEVSRSPFVERLLKKGYEVLFLTDAVDEYSISNLPEFEGKKFANVAKEGLKLDADEKAKEKQEALEREFEPLTKWLETKALKSRVTKVKVSHRLYESPAVLVASQFGWTGNMERLAKSNAHSKTQDATRDYYLSQKKILEINPRHPIVRELLRRINDDEDDMTARQMSLLMFETATLRSGYMLEDTEDFGRRVEKLLRKSLGVDE---------------------------------GKKRKDAKLSKNELMMLTVGEVIQELIKSHHEGKDVNLTKLKGQIASKYGLESQPRLVDIIAAVPSDYRKILLPKLRAKPIRTASGIAVVAVMCKPHRCPHINMTGNICVYCPGGPDSDFEYSTQSYTGYEPTSMRAIRARYDPFLQTKSRLDQLRQLGHDVDKVEFIVMGGTFLALPEDYRDFFIRNLHDALSGHHSQSVAEAVRHSEKSRVKCIGITIETRPDYCLRRHLNDMLLYGCTRIEIGVQSVYEDVARDTNRGHTVKAVQESFQLAKDCGFKVVSHMMPNLPNVDLERDLAQFVEYFENPAFRSDGLKIYPTLVIRGTGLYELWKTGRYRSYPPSVLVDLLAQILALVPPWTRVYRVQRDIPMPLVTSGVEHGNIRELALARMADYGTKCRDVRTREVGIQEIHHKIRPYEVELVRRDYVANGGWETFLSYEDVEQDILIGLLRLRQCSVDTFRPELKGGVSIVREHVYGSVVPVSSKDPTKFQHQGFGTLLMAEAERIAIEEHGSWKISVISGVGTRNYYRKLGYELDGPYMSK

>Aceria_tosichella

PDSKVNVLNQPLMEAAVIISGKPSSFVAGADINMIAQCKTETEEMKPFVAAVMGDCLGGGLELALACHYR-IAVDSPKTGFALPEVMLGLLPGAGGTQRLPQLIDIPTALTMMLTAKKLRASQAKKAGLVDMVVKPVGP--KLEEVAANQLADKTFKVRKRPLVENLARDF-VFNKAKDQVMKMTNGLYPSPLRIIEVVRGGIENKAFAELATTHSKALVGLYHGQTLCKKNRFGAPAQTVAVLGAGLMGAGICQVSIQRGHNVIMKDGLARGFDQIQKRAMEKEVTMARLQPQTDYTDLVIEAVFEDLGIKHKVVQEVEKDDCIFASNTSALPINQIAQASKRPENFIGMHYFSPVEKMQLLEVITTDKTSKRAAAIAVQAGLQQGKVVITVKDGPGFYTTRILSPVLSEAILLLQEGVTPKELDALKAYGFPVGAATLVDEVGVDVAMHVSKKVFEKRVDMVSGFKGRKSGKGFFVYRPLNEIIKKYTMEDHQMRLGTRFVNEAVLCLQEGILANPLEGDIGAVFGLGFPPFLGGPFRYVDTFGADKIVGMEKYRKFEPCSLLLEHA-RDRSKKFHMNGPIILLKEGTESDQGRAQILNNINACNLVANTIRTTLGPRGMDKLIIDGKGAVTITNDGATILKQLEVVHPVARTIVEIAKSQDAEIGDGTTSVVVLAAEILKEAKPFIEEGVHPQVIIRSFRNSLNIVLKKLDEVSVKLDNN-SDEIFIKCASTTLSSKMIAQKKAFFARLVVDAVKSLDDILPINMIGIKKVNGGALEDSQLIHGVAFKKTFSYAGFEMQPKVYENPKIALLNVELELKAERENAEIRVNNVAEYQNIVDAEWKILYDKLEKIAQSGAKVVLSKLPIGDVATQYFADRDMFCAGRVAEDDLKRTMRACGGNILTTCSDLNEQNLGKCGLFEEKQVGSERYNFFKDCPSAKTVTIIIRGGADQLMEEVDRSLHDAIMVVRRLYRKDSVVAGGGAIEMELSHHLRQQSRDVKGKEQTIMTALAKAFEIVPKQLCHNAGLNATAILSELREKHAHGELWHGVDVFSGTVANNLDLCVWEPAFSKKNSITAAIEAATMVLSIDETIKNAKSQAP-------??????????????????????????????????????????????????????????????????????????????????????????????????????????????????????????????????????????????????????????????????????????????????????????????????????????????????????????????????????????????????????????????????????????????????????????????????????????????????????????????????????????????????????????????????????????????????????????????????????????????????????????????????????????????????????????????????????????????????????????????????????????----------------GPDGFHALRDDALERANLIVNEIVQGSDNVVSLFDELSNCLCKVADLAEFIRVGHPQPRFRATAEQASIAISTLVEQLNTDRRLYDSLKKAKFND-------LDEHVAKLFLFDFEQCAINLDDDRRKKVLQLNESILKLGSIFAANANQGRLVKTEKLPQNITLINGLHADSDNEQVREFAYKTYLQPDKTQDEILTSLLRCRQDLAQICGFESYAHRAVKGSIAGSPEVVDFLDLLNDRIRHLANRDYDQMLQLKAWDVPYYSPYFSIGVCMDGLNLIFNELYGVRMQVDGELWHNDVIKLSIIESNELLGHIYCDLFERPMKQHQDCHHTIRGGCLRDGTYQLPIVVLVLSLPPALLTPSMVDNLFHEMGHAMHSMMARTKYQHITGTRCSTDLAEVPSILMEFFASDPRVLSRFARHHATGQPMPEELMTRWIKSKKVFTASETQLQVFYAALDQAYHSSNIFNTTDILARIQSQYYSIPYVENTAWQLRFSHLVGYGAKYYSYLVSRAVASAIWTRLFAENPLSRSAGQYREQVLAPGGGKPAQKIAEDVLIARSVVDSFMPSDSKKKRDAKKKEAAKVRASGNTSKG---VDELTAMLEKDLELAAQARSCTGVLGVPEQSRDIKIDNLSVTFHGVEILQDTRLELNYGRRYGLIGLNGCGKSTLFSVIGRREVPIQEQIDIYHLAREIPPLDKSALDAVLDCEKERIRLERLAEELAGQDDDDAQEQLIDIYERLDDMGADRAKAKAGFILKGLGFDKNMQLKKCKDFSGGWRMRIGLARALYLKPHLLLLDEPTNHLDLEACVWLEQELKTYKSILVIISHSQDFLNGVCTNIIHLDKTRLNYYTGNYDAFVKTREELQEHQMKRFNWEQAQISHMKDYIARFGHGSAKLARQAQSKEKTLAKMVNAGLTEKVTADKLVEFYFPSCGKIPPPVLMVQKISFRYSKSTPWIYKDLELGIDLDSRIALVGPNGCGKSTFLKLLCGEVMPEDGLIRRHSHLRIARYHQHLHEALDLDLSALDYMMKCFPDVTEKEVMRRIIGRYGLTGRQQICPMRHLSDGQRCRVVFAWLSYQVPHLLLLDEPTNHLDMETIDALADAINDFEGGMVLVSHDFRLIGQVAKEIWICENGGIRKWDKDIMQYKNHLRNKMLKERENMVLADLGSKITSALRSLGNATIINQDVLDSLLNQIVRALISADVNIQLVKKLSDNVKQVIDFDEMAQGLNKRRMIQMVVFQELVKLIDPGVKPWTPVKKKSNVIMFVGLQGAGKTTTCTKMAHYYRRKGWKCALVCADTFRAGAFDQLKQNATKACIPFYGSYEEADPVVIANEGVEMFKKEGFEIIIVDTSGRHKQETSLFEEMLSIQAAVKPHQIIFVMDASIGQACESQAAAFKSVVDVGAVIVTKLDGHARGGGALSAVAATKSPIIFIGTGEHIDEFEEFRVKPFVSKLLGMGDLEGLIDRVNELRLEENEDLMEKLKHGEFTLRDMYEQFTNIMKMGPFNQILSMIPGFEAGLLKGASEAESMSRLKRLMTMMDSMSDSELDSKEGVKLFSKQPTRLVRVAQGSGVRVQEVKELLKQYGNFAAVVKKMGGVKGLFKTGDITRNVNPNQMAKLNQQMAKMIEPKMLAQMGGVNGLQHMLKQIQTQ-LTKDEQFVKLKNYYNQHAKQMNMRKMFHDDPNRAQKFHVKLEKEEKHLLFDYSKNIINEDVMKMLIDLAKCRGVEEKRHAMVTGSKINFTENRAVLHVALRNRANRPIKVDDKDVMPDVNRVLDKMKGFCDKVISGDWKGWTGKKITDVVNIGIGGSDLGPVMVTEALKPYQRGPDVHFVSNIDGTHLATAIKKLNPETTLFIVASKTFTTQETITNATSARDWFLAAAKDKVHVAKHFVALSTNLAKVQEFGISPDSIFEFWDWVGGRYSLWSAIGLSIAVHIGFDNFCSLLEGAHYMDGHFYHAPLERNVPVIMAMLGVWYINCFGAETHAILPYDQYMHRFAAYFQQGDMESNGKYVTASGEQVDYATGPIVWGEPGTNGQHAFYQLIHQGTRLVPADFIAPVKTQNPISGGLHHTILLANFLAQTEALMRGKTESEARKELEAKGTDKEAVDKLLPHKVFPGNRPTNSIVVRKLDPFTLGALIAAYEHKIFVQGAVWAINSYDQWGVELGKELAKAIEPELKSAGDVTSHDSSTNMLINHIKANQIERKFEDKDKPSQIRQSNMKAAKAVADAVRTSLGPRGMDKMIQAANGDVIISNDGATILKQIQVLHPAAKMLVEVSQAQDVDAGDGTTSVVVLAGSLLDAAHKLLQKGIHPTVISEAFQSAAKTCREHLTNLAIPVDLNDLESLQRVASTSLNSKVVSQHSSLLAPLAVQAVLKIRQTDNVDLRNIKIIRKLGGTLDDTQLIDGLVLDSKFCSDFGSKRVEKAKIGLIQFCISPPKTDMDNQVVITDYTQMDRVLREERNYILNIVKQIKKTGCNVLLVQKSILRDALTDLALHYLGKTKIAVVKDIEREDVEFVTKTLGCRPIASLDHFTAEMLASADLVEEVTSAKYIKITGITPNPINKTVNILIRGSNKLVMEEAERSFHDALCVIRCLVKSSYLIPGGGAPEIHLSHHLTKLSHTMTGLQAVCYRSFAEALEIIPYTLADNAGLNPIGTITELRQKHANGDKNFGINMRKGTVTDMLSENVLHPLLVAVSAVTLAAETARSILKIDDLINTVRMTSKRVTYFWDPEVGNYHYGPNHPMKPHRLAVTHSLVMNYGLHKKMNIYRPYMASYHDMCRFHSDRYINFLYNVTPSNIHDYAHELQRFNVGEDCPVFEGLYSFCSRYCGASLQGAQQLNNKQCDIAINWSGGLHHAKKFEASGFCYVNDIVIAILELLKYHTRVLYIDIDIHHGDGVQEAFYLTDRVMTVSFHKYGNLFFPGTGDMYETGNESGRYYSVNVPLKEGIDDSNYAQLFKPVILDVIQYYQPTAIVLQCGADSLAGDRLGCFNLSIRGHGECVKFVKGFNLPLLVLGGGGYTLRNVSRCWTYETSLLVDEEINNEIPYSEYVQYFSPDFTLLLDKPCGDFTHNANSRLYLDTIYRHVSENLKRVAHSPSVQFQPTPGGWFSEES-----LEECEMKTEREESNSEPITEMSDDNDFMCHDEEDYDLEYSEDSNSEPDVDLENQYYNSKALKDTDPNLALESFQKVLDLEKDEKGDWGFKALKQMIKINFKLGNHEEMMNRYKQLLMYIKSAVTRNYSEKSINSILDYISTSHRMDLLQEFYETTLEALKDAKNDRLWFKTNTKLGKLYFDRGDFKRLSNILKQLHSSCQNDDGTDDLKKGTQLLEIYALEIQMYTVRKNNKELKKLYNQSLHIKSAIPHPIIMGVIRECGGKMHLREGEYEKAHTDFFEAFKNYDESGSPRRSTCLKYLVLASMLMKSDINPFDSQEAKPYKNDPEILAMTNLVNAYQSNDIVGFEKILNDNRNSIMDDMFIREHIEDLLKNIRTQVLTRFVKPYTRVRLDLISKELNLTVDEVEALLVTCILDNTIEGRIDQVARVLELMKHHQNNARYSAMDKLASQLASLQTTVMSRIIKPVSLNPVQILKGEADEEQGEQARLSSFVGAYAVGDLIKSTLGPKGMDKILCGTGRDEGRVEVTNDGATILRAIGVDNPAAKVLVNIAKVQDDEVGDGTTTVAVLASELLKEAETLVSKKFHPQTIIGGWREATQVAKQALEKSARDDPQDVENFRSKLITIAKTTLSSKIISQSKDLFAKLCVDAVLRLKGSGNLNSIQIIKKLGGVLEESFLDEGFLLDKMPGNGQPKRIENAKILIANTPMDTDKIKVFASRVSVDSVAKVAELELAEKEKMKDKVNLILKHNINVFVNRQLIYNYPEQLFADANVMAIEHADFDGVERLALVTGGEIVSTFGDPEKVRLGTCDLIEEVMIGEDKLLKFSGVPLGEACTIVIRGATQQILDEAQRSIHDVLCVLATVVKEPKLVYGGGASEMLMANAIEELAKKTMGKQQFAMEAYAQALRTLPTIIADNGGYDSSQLIPQLRALHAAGDSTKGIDMNKGQVADMTELGINESLAVKRQALVSAAEAAEMILRVDNILKAAPRRRQPDHRGMASNL----IPPRLAALEGHDDVINQATILKDEDGILSISNDKTIRIWLKRERGSYWPSVCHLLPFAPTCFHFDQNLKRLFVGLENGTISEFLVTDDFNRIDHQRYFSSHQSKVTSILYSSTCRLLLSVGKDKQFHWDDAESGTRLGSHPFQNSCTAVQFDPQSKHVFVAEQNGQISMLKLEQNSCRHITTLHGHSASIKSLLWDPFNKWLFSAGADKVITCWDIGGCKGTAHELQGHRNRIGALCYSRMNKLISGGEDCAIIAWNMEAKRMEAPQWSESDDCQRCKRPFFWNFKSMYETKTIGLRQHHCRNCGKAVCGDCSQRRSTIPLLGFEYQVRVCDECFPLFSDVTRKPLAKFFSATHHVNCMDLNESKGMLLTSGYDRTITLWNLSEMS?????????????????????????????????????????????????????????????????????????????????????????????????????????????????????????????????????????????????????????????????????????????????????????????????????????????????????????????????????????????????????????????????????????????????????????????????????????????????????????????????????????????????????????????????????????????????????????????????????????????????????????????????????????????????????????????????????????????????????????????????????????????????????????????????????????????FLDTGLMIKAIRPFHYQKLEVLYPSECRERAISYMGKLNLEFWYLNGNFMGSIDECVGQIPVMVKSKLCNLANATPKQFIRRKEDAEELGGYFIQNGNERIIRMLIAVRRNNPIGLSRNTWKDSFPFYSEFGVSMRSVGPGNQNMVLHYLTNGTAKLKVFYNARSVTMPVLMILRALVDYSDKKIFERLTAGKERQ-FFENSVITMMKLVKHGINRVDALEHLGSKLKWWSDLEAGKDLLRSSVAIHLDNDEDKFNCIALMTRKVYALAKAECAIENEDNPMFHEVYSSGQIYFMLLIERIELLLKGLKLCFDKHLEKMLTVVNPMKALVGTGNLQSQSGLGLKQAVGLSVTCEKINYLRFLSNFRAVHRGAFFAQMRTTACRKLYPEAWGFMCPVHTPDGTPCGLLNHLTIGCMVTVLVDGRLVCYVLRKYKVRGGVPNDIEICLIPKTGHPTQFPGVYIFTNIFRLVRPVINALNDYEFIGSFEQVYMDISINDKEVTTHTEISPTIFLSVLGCLIPYPDFNQSPRNMYCCQMTKQTMGSSSHTLRYRNDTKMYNITPQSPLVRPAIYDHYRLDEFPLGTNAIVAVISYTGYDMEDAMIINKASAERGFTMGVTKTITVDLKLDESGLPYVGSSNVPFCAYYKTKYEVYKYPEHAYVTDVKLIGSAQKATITFWQRRPFVGDKYANRHGQKGVCSLLWPQESMPFTESGMTPDIIFNPHGYPSRMTIGMMLESMSGKVSALQGEAIDATPFRFSEKDTASDHYGKLMEKYGFNYYGTESMYSGVDGSLMNAEIFTGIVYYIRLRHMVSDKYQVRSTGKVDQVTHQPVKGRRRGGGVRFGEMERDSLLAHGTSFLLQDRLFNCSDKTICTKCGSLLTVFIPHVLLYLTAE-LASVNISLLRETKHDIHRVQRNDPKLHPFALQREYQRALNASKLERVFAKPFIGNMGHGDIVSNLMKHQTKLSIMASGAYDGVIKIWNLTNRKCLRTIQAHNSQVRAMCDSGSNIKTILAKMDHHYEPYLITSGDKVELWEERKEPIRQWW-GADSTQCKFNSVETLSSDRAITLFDMRKPAPLRKVVLTMRSNQLCWNPMEAFKFTVANEDHDLHTFDMRNLAKPLALHKDHTAAVISLDYSPTGAEIVSGSYDKTIRIFHSRDVYYTKRMQRITDVIWSLDAKYIVSASDEMDIRMWRANASEKIGPKETNEALKKKFQHHPEIKRILRHRHLPKHVYKLKRTMLDARKKKLAKKLMMAGKYSDALPHFHEAINNDPNNYLTYFKRATVFLALNRPKSALDDLNKAIELNPNFTSALSQRASLHVKLGNLDEAHIDYERYLQSDPDNNEAKIEKLKEDALRLLEPIYSTQLMKTRANAFERVGEIRRAISDYRAVAKLSVDSAT-YLKIASLCYKLGEVEEALSNVRECLKLDPDHKSCYKPTKKLNKSMLSDCNIQQLPLIYSICRCLSKAGDGLKTCDQAL-DSSVICDKADLLVEKEDLAKLYQESQKLRQRAREGIEKIKKMQKQAKKRDYYKILAGESEINRAYRKLAAQWHPDRH-QDAEA-KKLAQAKFMDIADAKAVLTDPEKRQQYDRGEDPLDPESKFQFKFNFMLVLFETAQGYAFFKLHDDKKLAKVDKLVKTFQKGDDLSNLISLHHFEKFKSTSEAVEGAAALLDSKIGKKLKKAIKKSIVKSLEDELAVADPKLGTKIKDKFDITCVSTNAVQELMSLIRNKVEDLIPEWSNDDEMVMQLGTSHGIGRYKIKFSPDKVDTMIIQAVSLLDDLDKELNNYIMRCREWYGWHFPELSKILPDHMTYVRTILTIGMRSAATDVDLSEVIEEETANKVKEYSEISMGTDIADEDLENIKFLCQNIIELTEYRAQLYDYLRNRMMTIAPNLTILVGELVGARLISHAGSLMNLAKHPASTVQILGAEKALFRALKTKHDTPKYGLIYHAQLVGMSNQQTKGKMSRMLAAKASLATRVDALGDEPTNALGTEHRAKLETRLKMLEDTKLRVVNGFGKKRAKDRPVDHKSKRFKFE---------------------------------------------------------------------------PKDE----------------------------------------QYVWPKDRPDIKRRVVISLALLVTGKLISIQAPILFKNAIDCMNVGTDLILGYGAARAGSSLFNELRNSIFAKVASESIRQVAVNVFSHLHRLDLKYHLNRQTGALSRSIDRGSRGINFVLSSIVFNVVPTIFEVSLVSSLLYYKCGQFALVTLGCIGTFAFTRWRTKFRVQMNSAESQAGSKSIDSLINYETVKYFNNEKHELKQYQQASLKTTTSLAALNFGQQAIFSGWIMMLSAETTVGDLVMVNGLLFQLQMPLNFLGTVYREVRQSLIDMQAMFGLMRIKNKPLILNPSIVFDNVNFRNVLNGISFEVKSGKKVAIVGGSGCGKSTIVRLLYRFYDPLSGRVLINGHDIRDVSLRKQIAVVPQDAVLFHDTIKYNIHYGKFEEKVLAVSQLAELHDTIMKWYEAQVGERGLKLSGGEKQRVAIARAILKNSPILIFDEATSSLDSITEHKIMTALQAVENRTSVMIAHRLGTVVGADQILVLENGKIVERGHKELIRSLYSHLWHQQQS???????????????????????????????????????????????????????????????????????????????????????????????????????????????????????????????????????????????????????????????????????????????????????????????????????????????????????????????????????????????????????????????????????????????????????????????????????????????????????????????????????????????????????????????????????????????????????????????????????????????????????????????????????????????????????????????????????????????????????????????????????????????????????????????????????????????????????????????????????????????????????????????????????????????????????????????????????????????????????????????????????????????????????????????????????????????????????????????????????????????????????????????????????????????????????????????????????????????????????????????????????????????????????????????????????????????????VQGSSSDYRIDVSKHDYLETLGVNVKAKNFLVYQGAVESIAMKNPREITSLFEEISHSMDKAEEELFVKKKGITAEKKEAQGEEAKRYQSIRNEYQIDLQLFKLYHIDKEMSRRKKKNAIEEDIKNSRNIDKSIRDELNLNKKRPSYIKAKENAAHIEKKLEMAKKSLTGARKSHANHEDIKELEQDLERVIQEFLIEDVELQEEQRAEYTKLKKKASSMSAKYLKDLDSLEREQKADSDRLEAEVAKQREIEHKIREKEENVRRIEKLTDLSEFKKREREVHAKRELTQELADISSLGDAKVDRSENERQRKKGEIVENLKKIYPGVYDRMLNLCKPIHKRYNIAITKIMGKSMEAIVVDSEKTGRLCIQYLKDQMLEPETFLPLDYIASKPIKERLRNIQSPSNVKLIFDVIKFEPQDIKPAVLFATNNALVCETAADANKVAFELGDGQRYDAVSLDGTFYQKCGFISGGSLELERRARRWDEKEIHSMKHKKERLAEDLKENVKKTRKEGDLMVITSQIQGFESRLRYSKIDEIKVRMDKRAVEIKKIRDLMNGVEDDIFKDFCAQLGVDNIRQYEERQSKASQEKERLLQYESEKNSIQSRLLYER--SKDTSEVEQEDKNLLSAKEAERREMHAIEEEMRKVEQLKNDKISQKTECDKVEELITEKKRALQTIQKEISTIQKNIMTLECKMDSKRADRHAVFMHCKMESIALPLKEGSLQRAYQSDNIKPDFDVLEMQQEIAFRKIQAPNMKADERLDSAKERLRNTDSELNNLRKQAKDVFEAVKRERECFDTVSQRVDSIYKQLTNNPSAQAFLVPENPEEPYLEGINYNCVAPGKRFQPMSNLSGGEKTVAALALLFAIHSFKPAPFFVLDEIDAALDNTNISKVARFIRQRTVSSFQCIVISLKEEFYGHGDALIGVAPDPGDCTISRIFAVDLGVPGPVLIRLKNGIEQNNLVALQYLYDEAFPELTAQFFGILYGE--LHYRHIYANVRCDSYFNYCHLFNFIVSAQPINLQLPHKWMWDIIDEFIYQF--QNCSLLIWNVHSVLNVLHSLIDKSNIIEQLKVVNSGGDPTEAGGEFGNHPLYRMLGYYSLIGLLRLHSLLGDYHRAIRGLENVDLNKQMMVKAVACQTTTYYYVGFAYMMMRRYSDAIRTCTNLLVYLQRSKKTFQQELVEKQTDQMYVMLALCLALHPQRIDESVTTQMQEKFYAKLQRGDVAEFEKCPKFVSAVQLSIFMEEVTQQLVIRSYLKLYKTMSIEKLAGFLLLCFKHKKQSMVLDGELMSGSDVDFYIDRDMIHIADTKVARRYGDYFMRQIHKFEDLYK------------------------------MGPPDPILGVTEAFKKDTNPKKINLGVGAYRDDNGKPYILPCVKDAEKKVAAANFDHEYLPIGGNAKFCHAAAELAFGADSSVIKDKVNVTVQGLSGTGSLTLGAAFLRDHFPGSKDLYLPSPTWGNHIPLFKKNSFNIKHYRYYDAKTCGFDLKGCLDDISKIPEKSVIVLHACAHNPSGVDPKPQEWELISELVKKRNLFPFFDMAYQGFASGDIDRDAHALRLFIKHGHQVALAQSFAKNMGLYGQRVGAFTLTARDPEEAARLMSQVKIVIRPMYSNPPLHGARIAEAVLTDKALYGQWLKDVKGMADRIISMRKALRSGLTREGSKRDWAHVTDTIGMFCYTGMTADQVTRLWNEFSVYLTKDGRVSIAGITSKNVDYLAHAIHQVTK??????????????????????????????????????????????????????????????????????????????????????????????????????????????????????????????????????????????????????????????????????????????????????????????????????????????????????????????????????????????????????????????????????????????????????????????????????????????????????????????????????????????????????????????????????????????????????????????????????????????????????????????????????????????????????????????MARDGHGCHNEAKLKCPKCISLS-IDSSFCSQDCFKTNYNEHKLLHQASG--KYQPYPGFEYTGKLRPYPRTGPRKVPDHIKKPDYADHPEGRALSEEDIKESTHIKILNEQEIESMRLVSRFAREVLEEAAKITDVGVTTDEIDRAVHEAAIERDCYPSPLNYYKFPKSCCTSVNEVICHGIPDKRPLENGDIVNIDVTVYHKGFHGDLSETLIVGEPSEQHKKLVQVTWECLQKGIAAVKPGVKYREIGDVIQKHASLHGFSVVKSYCGHGIHRLFHTAPKVPHYAKNKAIGVIRPGHTFTIEPMIAEGSWRDTSWPDDWTAVTVDGLRSAQFEETLLATESGCEILTRRRTQKGKPYFMAS--MLQWMRDQSKRRRLLLSEQLGAQESDSLCQVLGTEDERTTSLADEQLIDTRYEYKGSSTFLKGTQSANPHNDYCQHYVDTGQRPQNFIRDYELHDRFEEYPKLKELIRLKDELIAQTATPPMYLKCDLRQGSLGQLDSKFDVILIDPPLEEYQRTQGVTKTKFWSWDDIMHLEIEEVAAPRSFIFLWCGSSDGLDLGRQCLRKWGFRRCEDICWIKTNKKNGHNKNLEPKAIFQRTKEHCLMGIKGTVRRSTDGNFIHSNVDIDLIISEEPPYGTYEKPDEIFHIIEHFCLGRRRLHLFGRDTTIRPGWLTVGPELTNSNFNSDLYKDYFSGSTDYLTGCTDRIESLRPKSPPPKGNVNGVSSV-LYR???????????????????????????????????????????????????????????????????????????????????????????????????????????????????????????????????????????????????????????????????????????????????????????????????????????????????????????????????????????????????????????????????????????????????????????????????????????????????????????????????????????????????????????????????????????????????????????????????????????????????????????????MRTFGDKPSPFQLEEGGELFYIGSEVGNYLRMFRGALYKKYPSIWRRTVSTEERRHISALGYGGHSIATNLTLLKASEVEDIFQGRDEKYKAVSLTTHDLTPRRLDHSRRNAISGVPSPTHHLDAVPCSTPIPKSRLQNKRVKTFPLIYDDLDPASLHRISNVGEVLVPIRLDMEIEGYKLRDTFVWNKNEISISPEQFADVLCDDLELPPQTFVPAIAQSIRQQVEAFTVPSNGDSGTDQRVLIKLNVHVGNISLVDQFEWDLSEKNNTPEQFAMKLCSELGLGGEFVTTVAYSIRGQISWNQKTYAFSEAQLSAIEFPYRSQDEGDQFSPFLETLTDAEMEKKIRDQDRNTRRMRRLAAQ--MGSNVSQLEREIGFPPNEHYFGLVNFGNTCYCNSVLQALYFCMPFRNKILESKAKSKRSKETLLTCLADLFYTIANQKKRTGSYAPKKFINRLRKENEIFDNYMQQDAHEFLNYLLNTIADLLRSDTSWVQEIFQGTLTNETRCLNCESISSKDEDFIDLSVDVEQNTSLTHCLRVFSKTETLDSEHKYYCEKCCSKQEAQKRMRIKKPPKILALHLKRFKYMEAQNRHTKLTSRVVFPLELRLFNDNSTEECEDRLYDLFAVVIHCGFGPNRGHYISIVKSYGFWLLFDDDFVEKLDTANLEDFFGLTNDSQKTSESAYILFYQSRD??????????????????????????????????????????????????????????????????????????????????????????????????????????????????????????????????????????????????????????????????????????????????????????????????????????????????????????????????????????????????????????????????????????????????????????????????????????????IK-------------------EQAKQIERSVAQKDNRNILRVLRT-------ISSKEFRK-----------LDERVLAGV----------KKTAVLPLIPELNVYFNLLILINLIESGRYEEAVKCSDQLMQIVKSQNRRTLDALAAKCYYFHTRCYELTGQLHQIKGFLHSSLRTATLRNDFEGQAVLLNCLLRLYLNYDQYDQASKLVSKSVFPESASNNEWARFLYYLGRIKAIQLEYSDAHKNLLQAIRKAPQNGAIGFKQTVHKLAIIVELLMGEIPDRSLFREPTLRRSLAPYFQLTQAVRSGDLNRFGIVIENYGPKFQADHTFTLIIRLRHNVIKTGMRMISLSYSKIYLADIAKKLNSDDSQDAEYIAAKAIRDGVIEAKINHEGSFLQSKETSDVYCTPEPQAAFHQRIAFCLDIYNQSIKAMRFPLRSYHADAETAAENADWQGYWMDED--EEFDG-IVIDRLALALANFALHSIWPEAIEDILKTFTINSVILLRILIFIAEEYSVRAKLNPLVFKFLHALLVD-----------RSLLDRIY-DIPIEEACATLAASFTSQKSNYTNSVIAFLPKIANLKDVIQLYLANEEIEKVYSLVINFSENHSRTLLRVLLNDGFQEVFTIIKIILDCTAAEGTFGQDEQYSDISFPFWFSFFENFYYYSE-CYTDMLFDPLVDTLMSLVTKAQYPTATIYDDDKRESFRCYRQDLGDNISLVSQFPRARDRILKQLYEQLSYEKPWQKLEAVIFAIKSIGEAVPYDEPNYV-PKIFNLLSKLPSRVELYCSVAEMISAYSDDHLATAFTILFMGVTSVRLMSTLSLKDLTTECQSELKTYSAQIVESCSKLNTNEKSRLMHTIGTTLAIT-SDIATASMSNLTMPLICEL-----LLRQLVPILLVIAEKY-RSDEDIMDKISGTIKRSAKSL-SIPLLNDLVSLVVNAYLEALIPLSLRQLSTTVEKYFNFNSVFKKINIEYIFRLAPEKRTLTELALYRQKSLGVELHRIFDLLLNNVFNIFGTPRNAIETVTDMLFYVTDA-GSLQRIVASREQKARFVSRLAQER-NRRKYKECC--NEFVMNVKVRWKKEFENVDLNLPTLFKAQLYTLTGVPIERQRLMCSGSMIKDDVWATTFMLMGSADKLPEPVEKPKFVEDLSETELAAAMDLPAGLTNLGNTCYMSATIQCLKVVPEFCVLKKFDASVTAALRDLYKSMDSAVVEPVILLRMMHLAIPRFAQRGEGNQQDANECWTELTRILQNFIDEYFSGVLGELKCDESPEESNENFLQLSCFIS-QDVRYLQAGLISRM-KETITKFSPSLNRDASYKVSRISRLPAYLTIQMVRFHYKGNVQTNAKLLRDVKFSMILDVYDLCTNELQEKLAPMRLKYKNADDLYTFEDDGSNNSGFYELRAVLTHKGRSSASGHYVAWIKKQWYKCDDDYVIDSEEILKLSGGGDWHCAYVLLYGPRYLRDLINTKLPKELILKIFSFLDIVSLCRCAQVSKYWNKLALDGDNWQSVNLFDFRVAVQGQVVENLSARCGDFLKRLTLRGCRSVSDSAMQTFSKNCRNLEEILDDCKQLTDETCKSLAEHLNIAS---------------CNQLKHINISNCNKIRPAGIESLAKNCTNLVSFIGTACINDESLKALSQKLKMINLNGCSAITDTGVKYLAENCHQLFYCCLSKCFALTDQALISLGQGCLKTLGLIGCHQLTDHGFQALTKNCKQLQDLDLEDCVLITDLTLYHLTNNCNNLKRLALSHCDLITDEGIKYIGTNYLELDNCIQLTDTAIDHLISCLKRLDIYDNNRISRQATKKLYPQLAIHTYFQASTPRQRYCRCCSIL??????????????????????????????????????????????????????????????????????????????????????????????????????????????????????????????????????????????????????????????????????????????????????????????????????????????????????????????????????????????????????????????????????????????????????????????????????????YLQMAWVERFRPKEFEDIVGNEEAVARLAQFAKEGNMPNIILHGPPGCGKTTVILCMARKILGPNIKEAVLELNASNERGIDVVRNKIKMFAQTKITLEPGKQKLVILDEADAMTEAAQQALRRIIEIYSKTTRFAFACNIFDKIIEPIQSRCAIVRFNRLNDEQLKRKLVDICNLMNVKYDDKGIQAVIYTAQGDMRQAINNLQSTHDGFEEITEDKVFKVCDEPPPIVIAKIIESCLKRRLDEAENTLAHLYSLGYCTEDIVSGMFRVVKSYNMPEFTKLEYLKQIGIAQIRVVQGVNSMLQMKGLISHFCAQAIKSPVYQREHSLVKPYQGSGMTIPNWEFFGSTIVTSNYIRLTQDTQSRQGGIWNVVPWQVEVAFRVHGHGSELFGDGLALWYVKEPPKGGPIFGNRDFTGLTVVLDTYANQNGVHSHGHPYISAMVNNGTQHYDHENDGVNSELAGCECKFRGLEHEARILVQYYNEELIIKTDIENSGIWRDCLSVKGVYLPTHYYFGITSATGDLSDNHDILSIKMRQLSQPKPDDLPRALPNRDKLNDGMSALRKLFLVVCVIAGVVAFLYQSKRNTARKRFYHQSDNMDEIIGRNCAVLDRNHLFLPMSTVSHIPDIKHFGIDPIYQNRTNLLQIHNIALNRAFFYSYILQKAQDAEPGFMYYMLAASADVSANPSVNSSAIYYSPNRAFTPSYNGFFNKTMPLFAPRAYRIDDYNDPYQLKGVSTMNTIAVTDLGAIDSNYTAEVYKINEWYSAWLPDLTKRHDSKPTYGVQISGTNETFVFHGPPGASDEPGPVKWQRPYYDCGRSNKWLVSASVPIADLFPRHTGWRHIELPIHVAASVIEMDFHRLDINQCPASEANGAESNYFADTAKCKRDTTTCEPIHGYGFRRGGYQCRCRPGHRLPKHVRAPYLGELIERASDFEYKQGFGCQKIENLAVKTQNVQTVTGVASRLDINMPGNIAHGKEHQFENQARAALRLSHFISSFLQVVDTNEMFAEFRVPDKPLTRDQVIGEALSTLIGDRQIVGLGVWFDRNQFFAPY--AYRLERNARNFFVLDTVKANIRFNSSGIKYDRYPIQYKVAQLGYWSEPFLDC-GLHNQWLISYASPFFGPDKLRLRVEFKGVVVVLKLSELDVNQCNAFKGTHKCDRKSTRCFPTSGRKFSGGYRCECKQGYEYPFNQPTTYIDGQMMEAPSRFDSLRFLSTSTKAKYGGRFMVTLLPGDGIGPEMMRHVKTVFAIGGIPVDFEEINLDSMNENIEKVEEAITSIKRNGVALKGNIETREHLKYFKSRNVELRTRLNLYVNIVHIKSQPSIETRHKDIDLFLIRQNTEGEYSSIEHETVPGVVSCLKVVTREKSEQIARYAFKFAVENNRKKISCVHKANIMKVSDGLFLNVAREVSKEFPQIEFEDIIIDNCSMQLVSNPWQFDVLLLPNLYGAVLTNIACGLVGGPGLISGANFGDEYAVFETGCRSTGKNIIGKNIANPLAIMNASADLLQYLKLDYHAQLIRNAVNKSLNEARVHTPDLKGQYTTTDVVNFIIDDIRHQL???????????????????????????????????????????????????????????????????????????????????????????????????????????????????????????????????????????????????????????????????????????????????????????????????????????????????????????????????????????????????????????????????????????????????????????????????????????????????????????????????????SKSSLEQLREHSVVVADTGDFELISKYKPTDATTNPSLILQAASKPQYAKIIEEAVKLGKGN---LEDTMDLVFVLFGCEILKIIPGRVSTEVDARLSFDVQKSVAKALKLIKLYEERGIKKERILIKLASTWEGIQAAKILENDHQIHCNMTLLFSLVQAVPCAEVGATLISPFVGRIYDYY----QVKKYEPFDDPGVKSVTKIYNYYKKYGYKTVVMGASFRNLDEIKCLAGCDLLTISPSLLEGLNETGLQVARSLDESKAK--DLEKVHYDEAAFRFYMNEDEMAHFKLGEGIRKFVQDQVKLEQMMKAKLGMSISTARPLISVYSEKNEATGSTIALPAVFKAPIRPDVVNFVHQNMAKNHRQPYCVNDQAGHQTSAESWGTGRAVARIPRVRGGGTHRSGQGAFGNMCRGGRMFAPTKTWRRWHRKINVNQKRYAMVSAIAASGVPALVQSKGHVIDSVPELPLVVSDKVQEISKTKQAVSFLKSINAWADVEKVYKSRRMRAGKGKMRNRRRIQRRGPLVIYGNDQGVTRAFRNIPGVDCVPVEKMNLLKLAPGGHVGRFIIWTESAFQALDKLYGTWKQAAKDKKGYNLPMPKMGNTDLTRLIKSEEIRKVLRPAQKKVVRRVRRLNPLRNQKAMLRLNPYAAVLKRNAILTIQKRMKQRLLAKKRGVKVPKYEAKPKKAGVKKPKRLVQGQLFKKILDAVKDLVNEASWDCSPSGMALQAMDTSHVSLVAAQLKSEAFDQYRCDKPIMLGMNLPNFFKFLKCAGNDDVITIRATDECDKVTLLFEDKNATETSEYELKLINLDNEYLGIPDQEYTVDIEMPSAKFSRICRDLNQIGDNVTISCVKDSVRFSISGDLGTGSINLSQNADADKPEDGVTIKMIEPICLSFSLKYLIQFSKAAPLSPRMKLSLKTDAPLVVSYEIGYMRYYLAPKMDEGE???????????????????????????????????????????????????????????????????????????????????????????????????????????????????????????????????????????????????????????????????????????????????????????????????????????????????????????????????????????????????????????????????????????????????????????????????????????????????????????????????????????????????ELEWLLKVDVKTSIKHLILADCAT-----STPTQYNL-TIKVNTTIEGYRITNADINIKLSSKHIIKTCIKNPFCWRLYQIQDANNHLNNAIDLLEAGFESAEEVLQLINDIMNSLLKSRSSLLTPKKSSVEELQHCQNMQSISPALPLDYTISFYVQANNLVCSVYQLSQSNNVK--NAEVSIPFLSDVLILLGLALQVCQQMLDKIQTL--NNLVPTFSPLLKELNPSLSNIQEVITGLDVVMEDVESVM--NAVVT-LLFTFASTLADKSSDKVSLR--VVKNLHDGLQYIAYTAMVKLATKRLPEVFTDVESVKSKYGFEKAQNLYRLLKTSASELASQIMIELLSTYTEENASQAEQDAVTCITSFLKDPNTFLLDHLLALKPVLYLEGKPIFDLLTIFVSEKLQNYIEFYNKNKSFV-DGLGLNHEQNLQKMRLLTFMQMAEGQKEISYETIMAELKIEHNEVEPFVFDVLRTQLVRAKIDQLNKKVLVQSTMHRTFGRPQWEQLRTVLNFAHIEKTIRMFLASLG-TR-RKKTVEQVNEGKHAIDENESRAIVDRSPQFQDLVQTLVNWINDELNDQRII--VKHLEMDLYDGQVLARLVEKLSGTKLDVVDVTQNEDSQRRKLREVLETVNRIFSIKWSVEGIHSRNTVQIIHLLVTMIRHFRPPI--KLPPNVTARVNVHMKQSEKLTEQYDETGMKVEPRDAFDTLFDHAPDKLAMVKRSLCGFVNKHLNKINLEELDPNQFSDGLLLVFLMASLENYFVPLGIFTQP-TDPVADMTGVQTALQHENYVNTSKLHNVNVAFQLMEDADVKNRVRAEDIVNADLKSTLRVLYEIFSKFTAGPAKIPHEVMLQVQRELLDYNISVMEMSHRSKAIIEETEANLRKLINIPDDYSVLFMHGGAKSQFDTVPMNLCSVEYIINGSWSKMAVKEAEKYATYTRQPAYEELQEFDDVTYRYYCDNETIQGVEFPLVCDMTSNFLSRPIDVKKFGVIFAGAQKNCGIAGLVIVIIRNDLIGKMRIVPNVQNYQIMQKDKSLHNTPLTFAIYVALCVKWALERGGLEGMRFSKEKSQILYDLIDQSNGFYSRSRMNVVFLL-----SNKDLEAKFLEESRLFELKGHRSVGGFRASLYNGIELDDVKISYRTRPEWADVEPVMDIKYTPRFEEAFSYFRAMLRDELSERSLALTADCIELQRSNFSVWYFRRRILRKLTHHLENELEFVGKMIEDEPKNYQVWHHRKTIVEWLDKQLTASVLDSKNYHAWQHRQWVIKEFSLWEGELEFTDAQIAMDVQNNSAWNHRYFVVTETKRFWLAEEIRYVHKRIENNESTWSYLRGILSPHLLAFVLDKACDLDQIRSRYWKYIKLKV?????????????????????????????????????????????????????????????????????????????????????????????????????????????????????????????????????????????????????????????????????????????????????????????????????????????????????????????????????????????????????????????ILPGDLVTSHNLMRGHGELISSVAGRVVQINKLISVHAPRARFVGETGDVVVGRIIEVQVGQRRWKVETGARLDSVLILNHINLPGGELRRKTVEDEIMMRSYFKEGDLIVAEVQSTFQDGSLSLHTRSLRYGLVGQGALVRVPPNLVERCKVFINLPQIGVHLILANNGYVWISEYGFDRQTIARMRNCIHIMKTGKIMLNSNSCTKCFEES-IIGKLNK??????????????????????????????????????????????????????????????????????????????????????????????????????????????????????????????????????????????????????????????????????????????????????????????????????????????????????????????????????????????????????????????????????????????????????????????????????????????????????????????????????????????????????????????????????????????????????????????????????????????????????????????????????????????????????????????????????????????????????????????????????????????????????????????????????????????????????????????????????????????????????????????????????????????????????????????????????---MELIEFANKVLKLEMFPYFEIAHVIMVCLSVKSDFGKGAVIFSRRHPLACWLSCMFATFSGTILANFLLNEPIVGAFKNTQQVLLASAVWYLMFYSPFDLVYKLCNFLPFKLLVACMKEVNRCHKIHHGVLYASKIYPSSYFIIVLIGTVKGNGAGLLKVMERMFRGIWQPSTIEFIQPTFATKASIAASILFIVDKKTDFISAPHSLVYFGVVIFLVYFKLSSVLLGINDPFGPFENLFCAIFFGGIWDALEQTITTTQNKDSHNKMSDM-RNGKDKGGKTKSMSSDEGLVRRRVTCSSNTNNCGTEADLFDNNDDARNIKLSLLEEVFLLGLKDRQGYTSFWNDCISIGLRGCILAELVLRKRITLDKSDRRRTSLSLRKVHVINAEHTGDALLDEALKHIHDTHPPESIHSWIYYLSGETWNPFNLKFQLKNVRERIAKNLVEKGVLSTEKKSFVIFDMTTHPLVNCPIKSKLVRRIQDAVLSDWVSDPHLMDKRLLALIILAYHSDVLENAFGPLSDDDYETAMSRSRTLLDLDMDYESSKPNANEALWAVFAAISKMQQPVKSFKVVLVGDGGTGKTTFVKRHKTGEFEKKYIATLGVEVHPLVFYTKYGPIEFSVWDTAGQEKFGGLRDGYYIQSKAAIIMFDVTSRVTYKNVPNWHRDLVRVCDNIPIVLCGNKVDVKDRKVKAKAIVFHRKKNLQYYDISAKSNYNFEKPFLYLARKLTGDSTLEFVAMPALAPPEVHMDPETIRKLEAEMKDAEIAPLPEDDDDDLVAEYFTPVLKQSKFKQTGVLTPEEFVIAGDHLVHQCPTWSWSSASDKPYLPADKQYLITKSVPCYKRCKDIEEKVILTD-EGWVDTHDDNDDDDEAEDMDKFMEENDTSVNIVATRTYDLNITYDKYYQTPRLWLVGYDENLKPLSIDDMYQDISQDHAKKTVTMENHPHIPA-VMASVHPCRHAEVMKKIISTVEEGGRLYVHSYLIIFLKLVQSVIPTLEYDFTQNFTI---------YQQLQAKLKLVNKKLARPLTLSEKILYSHIDNVETQDIVRGESYLKLRPDRVAMQDATAQMALLQFISSGLPKVSLPTTIHCDHLIEAKLGATKDLSKAKEVNKEVYDFLSSAASKYGIGFWHPGSGIIHQIVFENYAYPGCLIIGTDSHTPNGGGLGGLCIGVGGADAVDAMASLPWELKCPKVIGVHLTGNLNGWASFKDVILKIADILTVKGGTGSIIEYFGPGVDNIACTGMGTICNMGAEIGATTSVFPYNGQMRDYLIATNRSEIAAAADDNTNLLSADKNAKYDKVIELNLDTLEPYVNGPFTPDRGHTISQLGESAKKNNWPLDIKVGLIGSCTNSSYGDMTRAASVARQAIEHGLKAKSSFTVTPGSEQIRATIERDGQAETFRKFGASVLANACGPCIGQWNRQDIKKGDVNTIVTSYNRNFTGRNDANPQTHAFVASPEIVTALAITGRLDFNPLTDELVDSAGKKFKLQPPHGDELPSRGFDPGQDTYQPPAEDGSKVKVNVDPKSERLQLLSPFQKWDGKDLQDLLVLIKVKGKCTTDHISAAGPWLKFRGHLDNISNNMFIGAVNAENGEVNKVKNTLTNEYGSVPDTARYYKSKGLGWVAVGEENYGEGSSREHAALEPRHLGARAIIVKSFARIHETNLKKQGLLPLTFNDPKDYDKVQQTDKIDLVNLKELAPG------MFRNTFQSGLLSILYSVGSQPLQLFETRVKNGMVKRVTDEDIKSLVLEITSSNVSTTYITCPK-ISKSLGITLSHIVLIVKNLDRFFTFEIEIIDDTKAKRRFRASNYQTRTRVKDFICTMPLKLESGWNHINLNLADFTRRAYGTNYVETSRITVNANCRLRRIYFADRQYTDEELPAEFKLYLPVQDNPEEDSPT-----------QLLSAAKYKHPEHTKQDILNACKYIKTLMERYTFPTGVSKELICLDGTIPVTFRYNIPVGIFISDNHPFEAPLCYVRPTRDMTIKTSRHVDGSGRVYLPYLSEWNKNT-SDILSTIQVMQIVFGQMCPVYQK-AKALPNSTTGTITEEHIRLSLLSAVEDRLKYRMKEQIQDEIEVLKKTSNDLNRGKIQLDDMKSRMAQEASLSEELDGQLQEVDPDEVYGPTQPLFKQLLDAFAEENAVVDAIYHTGEGLRKGRISLDVFLKNVRELSRRQFMLRALMRACRAKASLP????????????????????????????????????????????????????????????????????????????????????????????????????????????????????????????????????????????????????????????????????????????????????????????????????????????????????????????????????????????????????????????????????????????????????????????????????????????????????????????????????????????????????????????????????????????????????????????????????????????????????????????????????????????????????????????????????????????????????????????SSSSLSSSTVINPTIGPLPSSPMDSLKEWSMSTFKCTKQLINEKRGLCPVTNDSQLQNDIEQLRGNREKLVQMLRFGQQMTDHYQLVRTQKQLHSLMNEMSIKCFTNNS-NLVDDFKKNAITLNVAINNGEKLITALNFYCSNLSTLIYKTIEDTLTTVKQFESARLEYDAEKN--SDKVRLRYEQLKQDVQIKMRFLEENTIKVMHKQLLLFNGAFASY-TSGNTAALDTTLKQFCIRFL--LKSDLLIDPDIRVWVFLPIVVITFLVGVLRNYVTILLMSTKKVDLQQIQDSQALIRARLLRENGKYLPKSSFLMRRHFFNNPETGYLTLAKNRPSTQPNPMNDPTMMSDMLKNNLTNMLPTILIGGWINWTFSGFVTTRVPFPLTLRFKPMLQRGIELASLNSSWVSSASWYFLNVFGLRGIYLLVLGENNQADHTRGMQDQMSGAAMSMPTDLKPAFKAEWEMLEIYEHRDTNSV---------------------MAHYKGDANEATRAMHISRRREKAKEELEEKRKKIEGDVKLSTIDNQFSTHTDSVEAQLKSSTVGLVTLDQMRQKQENAVKEREKQLAQKEDGSLKIGLDHGLKKSSIPTKNSKSLSF-YDDDDEDEDDDEDDKNE----------DTKPKCDDCNGSEGINEPKSDDSDNTSS-----TNVRKKPDIDTSLLPDRDREEEERRLREELAAEWRERQQRLKEEEIEITFSYWDGSGHRRVVRMKKGDSIYQFLQACLETLRKDFNELRSVSADQLVYVKEDLIIPHHYTFYDFIVTKARGKSGPLFSFDVHEDVRLVADASVEKDESHAGKVLLRSWYERNKHIFPASRWEPYDPTKTYDKYTISDKRSK???????????????????????????????????????????????????????????????????????????????????????????????????????????????????????????????????????????????????????????????????????????????????????????????????????????????????????????????????????????????????????CKSTAANVWKSVISVSAAGKKRGRGKGTGRVIAKDFNRGQQIGVGRHKLILPGLNTSVFAAKKPVEIQDLGKNDEFQEKLQAVRNEMNTFRKFRELPIERGFSGRRAHGRHAGQPDDHNETSFDGFDSIVLMLRPIQNMTGVMGRTKSMQALVVAGNKNGLAGFGMASGKDGRAVVRHARNRAAQALVYIPRFEGTVMHDFFSRYYQTTVFVERKPRGHGINAHRVIKAICEMFGITDLYANVEGVTRNQINMTKAFFLGLMNQKSYQDIANEKQLHLVDVREENYFYPQILASPEGVKTDSDIASGENLDFTYYINDGRVKLVTPKRKPFYEGDATWYKHLDRLDYGKNREKTKLILAAKYGSLDVFPHFKAFNRDDT-????????????????????????????????????????????????????????????????????????????????????????????????????????????????????????????????????????????????????????????????????????????????????????????????????????????????????????????????????????????????????????????????????????????????????????????????????????????????????????????????????????????????????????????????????????????????????????????????????????????????????????????????????????????????????????????????????????????????????????????????????????????????????IIVGIIGGTGLDQDSSLLTDETPYGRASDTQAIAGQIEGVDVFIISRHGKNHDVSPSHVNYRANLWTLVKQLNCTHVLVTSACGSLKEHIEPGHIILDQYIDRTRDRSFYVCHVEQRNPIMQQFILEHEGLTCVTIEGPRFSTLAESRLHRSWGCDVVNMTSVPEVQLATELATMYACILLVTDYDCWR-DDGECVSSSGVERMKDLGAKARKIIPGVIRRMLKDSIMSSRALHYVLKVGDLKKNIEFFRDKLAMRVLRHEVFDEGCEAACNGPYDNKWSKTMIGYGPEDDHFVLELTYNYSVGSYKLGNDLQYLKISLKDLFDKVLSTEQVSSGESRNFSLKSPDGYRFLIETGRG-QSKNDVTEVCLSCTNLNRSKQYWTELLKMTPAEESSNETVLSYSEQQARLRLCQINTALDHASAYGRIAFACPSADLRGIQAAVEAANEKVLTPFLSLDTPGKATVQVVILADPDGHEICFVGDEGFRELSQVDPEANTLIEKSMADDKSNEWFEKKGKSKSDAKLPPLPRTRDLLHVYGIRAKKNLSQNFLLNQLIRGLVRAAGRVIEVGPGPGNLTRAILEQSPFEVLAVEKDRRFLPLLEQLADSVLPGQLKILLGDALDHDFENIFGPKQLEREWEDVRLIGNLPFSISTPLLIKWLHHISLRNSFWQYGRVPMLLTFQDEVARRICAYERTRLSVMSQNCHVQYGKSFTPAAGVDTGVVRLEPKQPLCVPFKLFEKFNRHLFHHNLNYDRIEHAFDKAQVNPPYMISNKETARLCEEYYKLCLEPALVEYDYRASKR-MLPRFGQLL-------LISSHRRQLGYVPIVIDKTGKGERAYDVYSRLLKERIICVMGPIEDNMASSVIAQLLYLQSEHNRQPIHMYINSPGGVVTAGLGIYDVMQYVQPSIATWCVGQACSMASLLLAAGAPGWRHALPNSRIMIHQPSGHASGQATDIQIHAEEILYLKKRLYGIYEKHTKQDYDVIHAHMERDRFMNAEQAKEFGLIDTIIESMPTTTAEKK-?????????????????????????????????????????????????????????????????????????????????????????????????????????????????????????????????????????????????????????????????????????????????????????????????????????????????????????????????????????????????????????????????????????????????????????????????????????????????????????????????????????????????????????????????????????????????????????????????????????????????????????????????????????????????????????????????????????????????????????????????????????????????IHPIKALQDNYMYLLVTRHAAAVDPVNASAMASAVAVDLKAILTTHHHYDHAHGNSDMLVYGGDNRVQALNKHGD-VIKIGTLIECLATPCHTKGHICYYVVFTGDTLFIAGCGRFFEGSSEQMNQNLLASLPSDTKVYCGHEYTVTNLKFALSVEPQNIIKNKLDWAKREPTVPSTIGEEKKINPFMRLQVKQFTDELEVMTVLRHKKNEFVMNRLFGKTKPAAPGPSLSDIGSNLDKRAEQFDKKIQMLDAELFKYREQMKKMRDGPAKNSVQQKALRVLRQKKQYEQQRENLSQQSFNLDQANFTTQMLIETKGTVDAMKAGVKQMKQEYKNLNIGEIEDLQDDLQDMMADANEVQEALSRSYGVPDVDESELEAELEALGDELQKEDSSFLDEIATPSTSVAAEPQRRAN---QMI-----PSKSLMTSYLSSVRSKHTLPDLPYDYNALEPAISAEIMKLHHTKHHATYVNNLNVAEEKLAEALHKGDTTAVVQLQNVIKFNGGGHINHSIFWHNLSPSGGGDPPSELLTLINGSFGSVDNLKQAMSTAAISVQGSGWAWLGYNKDAQKLHVTTTANQDPLQPTHGLVPLLGIDVWEHAYYLQYKNVRPDYVKAIWRVVNWKDVAERLHKARSAEIAHESPKDVPRTGVDIRVQRLEIEKQLKDLEIDCVADYMKEKENIIDLYHRVNSCDQILERLETILCKFQADLGNICQEIISLHEQTVSLNMQLKTKQSVRTKLGQFIEDMTIPQPVIQHIMYTPACDKNFSDHLVILDQKIHFFKEQDFRDALSCNDVHQTLLGLKTKAIYKVREYVLRKIHDCRKYLSNYQVPQTALLKNKFFYQFLLSHERERAREVQTEYLDTMSKVYHSYFKEYIQRLCKLEYDDKPDENDLMASDDQGANIIFNKSSLKNRSTVFTVGTRASVIKEDLEAPLIMPST-AKQDVKYTPEAIFRTVHYALLDNTCREFVFLRDFFMTSDQQTTDLFNSVFAKTLSMIHMHFNDQFKSSYDTIAIFLCLHLVYRYREMARRKKVTVLDPYWDSTVRCLYPRFEKLIQLQINSVRNFNNDKFNNVDTMPHSITRRYAEFASALSSINDTYPDERISQLLSDLQNEVKNFILRVAAVFGQPKEQQIFMINNYDHILSVFKQSNREDSKDIEEIKLQLNKRTQEIVEELLYPHFGSIICFVKDCEVFLERDDQESLRASEKKVGALVEAFNLNWQKALDEISRDILSSFSNFENGNTIQQATMAQLLQYHLRLQKLLENPIMKDSQRKSKLLGLHELMNYVKKYKTNF???????????????????????????????????????????????????????????????????????????????????????????????????????????????????????????????????????????????????????????????????????????????????????????????????????????MRLTRFLRIPENYHNMPQRYVDRHTTFISKRAPRLPQYTQKIFRYKYDEWRPWQDDFKRNSEMVKVFVEPKFRGDRVEVLKGQDKGKQGVIIHMVKERNWVYVQGLNIKEEPYLIDEEVKLVDPSDLAPTDFEWRYDDQGLLLRVSTRTERVIPIPEAFETVDYVYKEQPKDTPADLVRQVTFEP-EVKTFEMDICETMGIKDERIPYPMYWY?????????????????????????????????????????????????????????????????????????????????????????????????????????????????????????????????????????????????????????????????????????????????????????????????????????????????????AYSVLAIVTCYSSDSPVSIRDYLKIPSTVGDAKRIGSLILRYKDDTVFGAYFSTYILLQSFCIPGSIVLSILAGYLFPALLALVIICICSTIGASTFYILIYN-RKKTLLKFLSNLFICVFLLRATPIFPNWTINLCSPLINIPLKPFVWGTFTGVAPLSVIHVWTGRILNDLSNDVNWQSVLMTSLVA-ISIVFLVFVFQTSTNSSQAKLDRIAIVNQDKCKPKRCNQECKRLCPVVKSGKLCVEVTPSDKIAFISEQLCIGCGICVKRCPFGAIEIINLPSNLDKDTTHRYGPNSFKLHRLPTPRPGQVLGLVGTNGIGKSTALKILAGKIKPNLGRWTDPPDWTDILAYFRGSELQNYFTKILENQLKAVIKPQYVDLIPKAFKGTVQEALDKKNETGRLEEFCKLLDLTPVRSRNIDELSGGELQRFAIALLCIQKADVYMFDEPSSYLDVKQRLKAAEAIRSLVTPKNYVIVVEHDLSVLDYLSDFTCCLYGKPSVYGVVTMPFSVREGINIFLDGFVPTENLRFRDTELVFKVAESAQEEIKRMCHYEYPRMTKALGGFKLNIEPGTFTDSEIIVMLGENGTGKTTFIKILAGGLKPDGDSDLPSLNISYKPQKISPKSQKSVKDLLMEKIFDMYRHPQFQTDVLKPLEIERIQDQLVTELSGGELQRVALVLCLGKPADVYLIDEPSAYLDSEQRLVAAKVIKRFLLHSKKTGFIVEHDFIMATYLADRVIVFEGQPGLDAKAKSPQSLLTGMNTFLDMLNITFRRDPNNYRPRINKLNSLKDVDQKRSGNFFFLEE----EDLFGPELDHYEAAEATMRQRDRMKSRRGLETIDNIEDTRGMTNKEWLSQATKNEIRNRFKNLLKTFKEKLRTMELNQQSFELEYILAQEQPALALFLVEAPHQMLDIFNQAAKEVVFSMYPAYGKIAEEIFVRIKDLALAEDIRSLRQLHLNQLIRTQGVINSATTILPQLSLVKYDCLKCKYMLGPFVQQQEIKPGTCPGCQSTGPFAINMEETLYKNYQRLTIQESPGKISAGRVPRAKDVIVLGDLVDSCRPGDEVDLTGIYTNSYDGSLNIATGFPVFTTVIQANSIVRKQSDADVHQIVNISKDPVVERIMASICPSVHGHSNIKRAIALSLFGGCAKNPSGKHRLRGDINILLCGDPGTAKSQFLKYTQTIAPRAVYTTGQGASAVGLTAFVSPVTREWTLEAGALVLADNGVCLIDEFDKMSDRDRTSIHEAMEQQSISVSKAGIVASLQARCAVIAAANPVGGRYNTFNMNVDLTEPIISRFDIICVVRDSVLAKFIVRSHRKSHFIDKDLLRRYIVYARDRFKPKIQMETIAQVYADMRRESAQTNSIPITTRHIESIIRCSEAFAKMHLRNHVDVRMSIKVILESFITQKHQEQKMTLLGEIEEYFDAKDLYQVLGIDKNATSDQIKKAYRKASLKVHPDRVGEKLKEKATKRFQVLSKVHYVLSDEERRRMYDDHGVIDSEGNLETGTDWLDYWRLLFPKVTVKDVDSFFDRYIGSEEEEKDLISIYNKYEGDLDKISDSHIGYDEERTVKDLERLIEAGKIEKFDKFVNEPAAKKAKRLRLYKREAKEAAKI--KSASSFDELSALVQQKNTRNFDDLISGLEAKYSKKPSERGTKRKRA???????????????????????????????????????????????????????????????????????????????????????????????????????????????????????????????????????????????????????????????????????????????????????????????????????????????????????????????????????????????????????????????????????????????????????????????????????????????????????????????????????????????????????????????????????????????????????????????????????????????????????????????????????????????????????????????????????????????????????????????????????????????????????????????????????????????????????????????????????????????????????????????????????????????????????????????????????????????????????????????????????????????????????????????????????????????????????????????????????????????????????????????????????????????????????????????????????????????RRAPRLAILLL--------------------------------TAHSQDEAGV-----------------EKHAFQAEVSRMMKLIINSLYTNKEIFLRELISNASDALDKIRVLALTDKDTLDSLGELEIRIKADDARGALHVMDTGVGMSKSELITNLGTIAKSGTSEFIKKSLDNPEKVQLNDLIGQFGVGFYSSFLVADKVSVRSKSHQEPFEHVWESNSTEFSVVEDDGSDQLKRGSVVTLHLKDEAKDFLKPDTLRELIKKYSQFINFPIYLWTSKTVTEEVPVDDD-----ESTD-KKDEDATSTTEEEKTKKKTKKVEKTVYDWERINVAKPIWQRKPKDISDEEYDEFYKSITRDHQPPIVRTHFTAEGELTFKSLLFIPKVQPTESFNKYGNKNDNIKLYVKRVFISDDFNDLMPAYMRFIRGVVDSEDLPLNVGRETLQQHKLLKVIKKKLVRKTLDMIKKIDDNKYIDFWKEFGTNLKLGIIEDQNNRNRIAKLLRFHSSKTGPDGWTSLEEYVKNMLPEQEQIFYIAGSSYDEVSQSPFVESVLKKGYEVLYLTDAVDEYTLSNLPDFDGKRFQNVAKEGLTLDKSKKRELYKKALEGKYAKLVSYLQETALKGKVHKVVLSERLSESPAALVATAFGWTGNMERLAKSNAHSKTNDATRDYYLQQKKILEINPHHPVIKELLKRVEADESDERVQEAASMVFAIATVRSGYMLQDLEDFGKKIERYMRTDLHVPLDAPVEEEQIEESATKTEESTKEDESKADTEEKP---MAQLKLSHQEKQVLVVREIIETLIEAHEKNEDVDLSKLRNKIASKYATSKVPSLVDIISGVPHEYKPILLPKLRAKPIRSASGIAVIAVMC--------------------GPDSDFEYSTQSYTGFEPASMRAIRARYDPYLQTKGRIEQLQRLGHIVDKVEFIVMGGTFMSLPPDYRDFFIRSLHDCLSGHQSKSVAEAVKMSERSKIKCIGITIETRPDYCLKRHLNDMLDYGCTRLEVGLQSIYEDVAVDTNRGHTVKSVCQSFQLGKDCGFKMVSHIMPNLPNVDLERDLNQFVELFQNPAFRPDGLKVYPTLVIRGTGLYELWKTNRYKSYPPSVLIDLLAQALSLVPPWTRIYRIQRDIPMPLVSAGVEHGNIRELVLARMADYGMKCRDIRTREVGIQEIHHKIRPYNVELIRRDYVANDGWETFLSYEDVEQDILIGLLRLRKCSPGTFRPEFIENTSIIREHVYGSTVPVNTKNPVKFQHQGFGMLLMEEAERIALEEHGSSKISVISGVGTRNYYKKLGYQLDGPYMSK

>Fragariocoptes_setiger

--------------AGVIISAKPGCFVAGADINMLSNCKTETENLKPIVAAIMGDCLGGGAELALACHYR-IAVNNPKTVISFPEVMLGLLPGSGGTQRLPKLIDIPTALTLCLTAKRVRADKAKKMGLVDLVMHPLGPGLYLEEVAAKQLADGQLKVRKRPLMENLARDF-VFNKAKDQVMKMTNGLYPAPLKIIDVIRNGIENKTFAELATTHSKALVGLYFGQVLCKKNRFGTPAKSVAVLGAGLMGAGICQVSIEKGVQTIMKDGLARGQNQIKRRNIQRDTTMAKLFPQTDYSDLVIEAVFEDLALKHKVVKEVEEDDCVFASNTSALPIHMIAQASKQPENFIGMHYFSPVDKMQLLEVITTDKTSDRAASIAVQAGLRQGKVVITVKDGPGFYTTRILSPVLSEAILLLQEGISPKELDVLKSYGFPVGAATLVDEVGVDVASHVSKQVFGQRTEMVGGYKGRKSGRGFFVYRALNDILKKYTREDHQMRLGTRMVNEAVLCLQEG---------------------------------------------------------QDQSTSF-MQAPIILLKEGTESQQGLSQVINNINACQLVAQTIRTTLGPRGMDKLIVDGKGAVTITNDGATILKQLDVVHPVARTLVDIAKSQDSEVGDGTTSVVVLAAEFLKQARPFVEEGVHPQIIINSFRNSLQFVLKKLDEIAVKVNDN-SDEIFMKCAATTLSSKMIAQKKEFFARMVVDAVKSLDGHLPLNMIGIKKVSGGALEDSQLIAGVAFKKTFSYAGFEMQRKKYQNPKIALLNVELELKAERDNAEIRIDNVSEYQSIVDAEWKILYEKLEKIHKSGAKVVLSKLPIGDVATQYFADRDMFCAGRVPEDDLKRTMRACGGSILTTCNDLEDSNLATCELFEESQIGGERYNLFKGCPNAKAVTIIIRGGADQLMEEVDRSLHDAIMVVRRLYKKDSVVAGGGAIEMELAHHLRQHSRSIAGKEQSLAAAVAKAFEIIPRQLCQNAGIDATTILSQLREKHAGGATXATFDEFESEMANETAVCTTEPSVSNDTTATTGAEAQ----AIPSTTKDTKSAAPTETANPSVNQLLPEALTKTGETG-PTQKLSQPRLSYKTISSVYGPLLVVDLVRYPQYGEIVQIKLPDGSRRTGQVLEFKGSKAVVQVFEGTSGLDVKNTQCEFSGELLRVPVEEDMLGRVFNGCGKPIDGGDDITPEKYLDINGEPINPASRDYPKEMIQTGISAIDVMNSIARGQKIPIFSAASLPHNEIAAQICRQAGLVRAHDNSDKD-NFAIVFAAIGVTSETARFFRQEFIANGSMENVCLFINLANDPTFERIITPRIALTTAEYLAYECGRHVLVILTDMTSYCDALREISSAREEVPGRRGYPPYMYSDLATIYERAGRVEGRSGSITQIPILTMPNDDITHPIPDTTGFITEGQVYVDKNLHGRKIYPPINVLPSLSRLMKSAIGPGMTRPDHSDVSNQLYACYATAQDVAPMKKVAGEEALTADDLLYLEFMEKFEKGFLAQDMHENRTVFESLDLAWKLLRMFPKQMLRRIPPKVLEEYYPHTRHV-----------ANLLM--SAFRLLIEEVVSASERAKSRSGSDNFEVVEIFDELSNCLCKVADLSEFVRVGHPHPRFQAAAEHASMAISSLVEKLNTDKRLYQSLKDSLRYS-----TTLDRYVAKLFIFDFEQSGIHLDDDKRKLVLQLNEDILKLGSIFTANSNQPRLVPVSCLPEEIALINGLCADSDNENLREVAYKHYLRVDERQENLLSKLLYARSRLARLCGFKSYAHRAVKESIISKPESIEFLDILNERIRPLAERDYAQMLDLKAWDVPYYSPYFSLTACLNGLNVIFNHLYDIRMQETGELWHNHVNKYAILDTNERLGIIYCDLFERHGKPHQDCHFTIRGGCSRDGTYQEPVVVLMLSLPPSLLTPTMVDNLFHEMGHAMHSMLARTKYQHVTGTRCSTDLAEVPSILMEFFAADPRVLSKFARHYATGDAMPNELMHSWIKSKKIFAASDTQLQVFYAALDQAYHMPFGLCLVRQVNNIRNKCY---HVDEHPFEIHSCQLLHVLARWLSVFMILEIGNVILMVMVKDDNYTSQRWTYINTLLKASSLSPFACVCETVLISLAIVVNIMPSESKKKRDAKKKEAAKARQGGGTINGETNVDKVTAMLERDLELAAQARSCTGVIGVHPRSRDIKVDNLSITFHGHEILTDTKLELNYGRRYGLIGLNGCGKSTLLSVIGRCEIPIQSTIDIYHLTREIPPLDKNALEAVLDVEQERIRLEKLAESLANCEDEESQDQLMDIYERLDDMGADRAKAKAAYILYGLGFDKAMQQKKCKDFSGGWRMRIALARALYLKPHLLLLDEPTNHLDLEACVWLEQELKNYKRILVIISHSQDFLNGVCTNIIHMNKLRLEYYGGNYDAFVRTRLELLEHQMKRYNWEQDQIAHMKDYIARFGHGSAKLARQAQSKEKTLAKMVASGLTDKVVNDKLVEFYFPACGKIPPPVVMIQNISFRYNDKSPWIYKNLELGIDLDSRIALVGPNGCGKSTFLKLLVGEVIPQDGLIRKHSHLRIARYHQHLHEALDLSLSALEYMQKCFPEVKEREEMRRIIGRYGLSGRQQICPMRHLSDGQRCRVVFAWLAFQVPHMLLLDEPTNHLDMETSDALADAINDFEGGLVLVSHDFRLIGQVAKEIWICENSTIRKWEKDIIEYKNHLRSKILKETEFMVLADLGRQITSALRSLGNATIINQQVLDSLLEEIVRALLSADVNIKLVARLRQNVKQVIDFEEMAQGLNKRRMIQMVVFQELVKLVDPGVKPWQPVKACSNVIMFVGLQGSGKTTTCTKMAYYYQRKGWKCALVCADTFRAGAFDQLKQNATKARIPFYGSYDESDPVVIASEGVEKFQNEGFEIIIVDTSGRHMQEASLFEEMLSIQTAVQPDQIIYVMDASIGQACEAQAAAFKSKVDVGAVIVTKLDGHAKGGGALSAVAATQSPIIFIGTGEHIDEFEPFKVKPFVSKLLGMGDLEGLIDKVNELKLEENEELIEKLKHGEFTLRDMYEQFTNIMKMGPFNQLLNMIPGFGADLLRGASEAESMSRLKRLMTIMDSMSDSELDSREGAKLFSKAPTRVARVAQGSGVRQREVQELLAQHTKFAAVVKKMGGIKGLFKGGDMSKNVNPAQMARLNQQVARIMDPRMLAQMGGMNGIQNMMRQLQAQDLDCGSNF----------TMAMFMSK------NKSQLTKISVSLMLHNLLFDYSKNLINEDVITSLLELAKSRGVEEKRHAMVTGANINFTENRAVLHVALRNRANRPINVDGKNVMPDVNRVLDQMKAFTESLINGTWVGFTGKKITDVVNIGIGGSDLGPVMVTEALKAYQCGPNVHFVSNIDGTHLHSVIKKLDPETTLFIVASKTFTTQETITNATSAREWFLQAANDSKHVSKHFVALSTNVPKVKKFGISETAIFEFWDWVGGRYSLWSAIGLSIACHIGFDNFVKLLEGAHYMDGHFYHSPLERNLPVLMAMLGVWYINFFNAETHAILPYDQYLHRFAAYFQQGDMESNGKSVTDTGERVNYQTGPIVWGEPGTNGQHAFYQLIHQGTRLIPCDFIAPVKSHNPIRNGLHHTILLANFLAQTEALMMGKTAEEARRELEAKGTPSDTLDKLVPHKVFIGNRPSNSIVVDKVDPFTLGALIAAYEHKIFVQGIVWGINSYDQWGVELGKELAKAIEPELKSSTAVTNHDTSTNMLINYIRDRQQDRSFKDKDKPGQIRQSNMAAAKAVADAVRTSLGPRGMDKMIQGANGDVTITNDGATILKQIQVLHPAAKMLVELSQAQDIDAGDGTTSVVVLAGSLLDASQKLLQKGIHPTIISDAFQEAAKKCRESLEQLAISVDLKDMESLQRVASTSLNSKVVSQHSSLLAPIAVNAVLKIRQTDNVDLRNIKVIKKLGGTLDDTHLIEGLVLDSKFCSDFGSKKVEKAKIGLIQFCISPPKTDMDNQVVITDYTQMDRALRDERNYILNIVKQIKKAGCNVLLVQKSILRDALTDLALHFLGKSKIAVVKDVEREDVDFICKTLGCRPIASLDHFTSDHLVSADLVEEVTSAKYIKITGIQPNPVNKTVNILIRGSNKLVMEEADRSLHDALCVVRCLVKSPFLIPGGGAPEIHLSHHLTQLSHSMTGLHAVCTRAFAEALEIIPYTLAENAGLNPISTITELRQKHASGDKNYGINIRKGTVTDMLGENVLHPLLVATSAVTLASETTRSILKIDDLINTVRMTQRRVSYFWDPDVGNYHYGKHHPMKPHRLSVTHSLIMSYNLHTKMNIYRPYNASIMDMCRFHSDQYIDFLSRVTPSNINEFSKYLTAFNVGEDCPVFEGLYNFCSKYCGASLQGAQQLNNKQCDIAINWSGGLHHAKKFEASGFCYVNDIVIAILELLKHHVRVLYIDIDIHHGDGVQEAFYLTDRVMTVSFHKYGSLFFPGTGDMYETGAESGRFYSVNVPLKEGIDDTNYATVFKPIITDVIQFYQPTAIVLQCGADSLAGDRLGCFNLSIRGHGECVKFVKSFGLPLLVLGGGGYTLRNVSRCWTYETSLLVDEEISNEIPYSEYVQYFSPDFTLLLDKPCGDVTSNHNSRAYLEAIVGMVRENLRCVAHSPSVQMQAIPTDFFKPEE---------MEATKWPDLSEEKIDAMSDDADFMCDDQEEYDLDFSDNSNSEPDVDLENQYYTSKSIKESDPQAALVNFARVLELENGVKGDWGFKALKQMIKINFKLGQYEEMMRRYKQLLTYIRSAVTRNYSEKSINSILDYISTSKQMELLQEFYETTLDALKDAKNDRLWFKTNTKLGKLYFDRGEFQRLSKILKQLHASCRNDDGTDDLKKGTQLLEIYALEIQMYTVQKNNKKLKRLYEQSLHIKSAIPHPMIMGVIRECGGKMHLREGEYEKAHTDFFEAFKNYDESGSPRRSTCLKYLVLANMLMKSGINPFDSQEAKPYKNNPEILAMTNLVSAYQNNDINEFEKILKENRNTIMDDMFIKEHIEDLLRNIRTQVLTKLIRPYTRIHIPFISKELNLDPHEVETLLVSCILDNTIEGRIDQVNQVLELTRQSHNNAKYLALDKLTNQLASLQTTMVEGQIRPISLNPVQILRGDADEEQGEQARLSSFVGAYAVGDLIKSTLGPKGMDKILYGSGRDEGRIEVTNDGATILRAIGVDNPAAKVLVDIAKIQDEEVGDGTTTVAVLASELLKEAEALVSKKFHPQTIITGWRQATQAAKKALEDSAVSDVDDPEKLRAQLMSIAMTTLSSKILSQSKELFAKLCVNAVLRLKGSGNLDSIQIIKKLGGALEDSFLDDGFLLDKTPGNGQPKRIENARILIANTPMDTDKIKVFASRVKVDSVAKVAELEIAEKEKMRDKVNLILSHNINVFVNRQLIYNYPEQLFADANVMAIEHADFEGVERLALVTGGEIVSTFGNPDKVRLGTCELIEEIMIGEDKLLRFSGVPVGEACTIVIRGATQQILEEAQRSIHDALCVLSRVVKDPRIVWGGGCSEMLMANAVAELAKKTSGKHQFAMESFATALRTLPTIIADNGGYDSSQLISELRAAHALGKKKLGFDMYQGCIADMSELNISEALVVKRQALISASEAAEMILRVDNIIKAAPRRRQADHRGMSVELREFETLELLSRLDGHQDLITQACILRNEDGVISISDDKTIRLWLKRDTGSYWPSVCHILPSPATCMHFDHDSRRLFVGLDNGTISEFMISEDYNRINHQRFFPSHQSRVTALLLAPSCRWLLSAGRDKHFHWYCSDSAYRLGSYQCTAPCTSVQFDELSKHVFIGDQHGQITMLELIQSSYKFVKTLGGYQTTIRFLEWDPASQMLFSAGSDHVIICWDIGGRKGTTYELQGHSSQVTALRYAKKSHLVSVGDDCRIISWSMKAQRTETPPWAESDNCQKCQKPFFWNFKVMFDQKTLGIRQHHCRNCGKAVCNDCSLNRTKIPPMGFEHSVRVCDECYAQFSNITCTSLANFYSAKHMITCMDLDESKKILLTTGPDRSIKLWDSSPILM-------------------------------------------LIQRDNYSXSMLFYCSV-TDRKLVLIKELSQELVGQTIWLRARLHSSRGRGKQAFLVLRQQQYTAQAILRVSEGVTKEMVSFASSIPRESIIDVFGLVKQSPVKIESCSQHDVELELSELYVVSQAKPQLPLLLEDASRSEAESADPGMLTIIVNQDTRLDNR---------------------LFRASLNKRGFIEIHTPKIIMAASEGGANVFEVTYFKRKAYLAQSPQFYKQMAIAADLDRVYTIGAVFRAEDSNTHRHLTEFVGLDLEMAFNYHYREVVDVIAQMFVDIFKGLQERYEHEIKVIARQYPSQKFVFLEPSLILNYTEGVAMLRESGVEIGDEDDLSTPNEKLLGKLVKEKYNTDFYVLDKFPLAVRPFYTMPDAQNPRYSNSYDMFMRGEEIMSGAQRIHDPELLIERAQLHQIDVTKIESYIDAFKYGCAPHAGGGIGLERVTMLFLGLDNIRKTSMFPRDPKRLAP----------------------------MRGRTYKGRLYLEFWCLDGKMMGTFNEYVGEVPIMVKSELCNLNRLTPKQMIERYEDAEEFGGYFIANGNERLIRMLIAQRRNYPLGILRNGWKDAGPMFSEFGVSMRCVGDSGQNMVLHYLTNGTAKLKLFYKGQQLFLPLMLVARALVDHTYFRIFQLFMRGKESNAFYRGCVVSMIRLVKQSINSEQALAYIGEKFRWYSDLEVGEHFIKTSMAIHLPNNEDKLNCLVMMTQKLFALAKVECAIENADNPMFHEIHTSGQIFFTLLIERIDQFLSGLKMCFDHHMLRMLTIVRPLEFLIATGNLQSTTGLGMMQRVGISVMAEKINYLRFVSHFRSVHRGAFFAQMRTTACRKLYPEAWGFLCPVHTPDGTPCGLLNHLTESCIITVTVDGKLIGYVVRDHKVKGGIPNQIEICLIPRTAHPTQYPGVFIFTTMSRMMRPVFHRLEAVEYIGTFEQVYMDICLVEEEATTHRELSETAFLSVLGALIPYPDFNQSPRNMYCCQMTKQTMGQASHTLLYRCDTKMYAVTPQSPLIRPAIYDHYHFDEYPLGTNAIVAVISYTGYDMEDAMIINKSAAERGFTHGIMKTVTVDLKIDRDGLPFIGSMDDPICVYHELKMEFYKSSEPAYVLDVKLLGDLQKVAITLWLRRPFVGDKYANRHGQKGVCSLLWPQENMPFTESGMTPDILFNPHGYPSRMTIGMMLESISGKASALRGEPLDSTPFRFSENSHASEHYGQMLADSGFNFYGTERMYSGTDGNELIADIFIGVVYYIRLRHMVSDKYQVRSTGPIDQITHQPIKGRRRGGGVRFGEMERDSLLAHGASFLLQDRLVNCSDGCLCKHCGSIITVYLPYVMRFLVAE-L-------MRETKNDIHKVFRNDRKLHPHRIEREYQRALNATKLERVFAKPYIGNLGHGDIVNCLMKHQTKLSVIVSGACDGQIKIWNLASRRCVRTIEAHNSVVRSLCDSQSTIKTVIAKMDHHYRPFFVTVGDKVDLWEERNEPLRSWW-GADSSQVKFNPIEATSNDRGITLYDTRKANPMRKIVLEMRSNQVCWNPMEAFVFTAANEDHDLYTFDMRNLNKPLIIHKDHTEAVITLDYSPTGLELVSGSYDKTIRIFRSRDVYHTKRMQKLTDVAWTHDGKYIISASDEMDIRLWRARASEKIGPKNVQETLKKKFANHPEIKRISRHRHVPRHVYKEKREMLDSRKRK???????????????????????????????????????????????????????????????????????????????????????????????????????????????????????????????????????????????????????????????????????????????????????????????????????????????????????????????????????????????????????????????????????????????????????????????????????????????????????????????????????????????????????????????????????????VFVVFKTAQGYAVFKLND-----KVEKLVKYFKKSDKYSSLIELHHFEKFKSTAEAVESATCLIEGKVAKKLKKTIRKCIINDIQDELAVAESKLGTKIKEKLQINCVTSQAVQELMSMIRLKIDDLIPDWSNENNEIMQLGVAHGLGRYKLKFSPDKVDTMIIQAVSLLDDLDKELNNYVMRLREWYGWHFPELCKIITDPTTYVSTIMAIGMRSNAADADLSEVLPEAMQQQVKEYAEISMGTDIADDDLINIKYLCGNILELTKYRSELYEYLKNRMMTIAPNLTVLVGELVGARLISHAGSLMNLAKHPASTVQILGAEKALFRALKTKHDTPKYGLIYHAQLVGQSGTALKGKMSRMLAAKASLATRVDALQEDVDATLGTEHRATLEARLKSLEDGTLRKVSGTGKRRVNDDSFSNKSKKIKSTVWNTSTSGT-----FRQ-----AVWKLL---------------------------------------MFSMNLNWPPDVLIF------DPGISF-------------------------MYIWPKDRPDIKRRVIIALGLMIAGKVISIQAPFMLKHVVDYLN----TNQSYGAARAGSSLFNELRNSVFAKVATDSIRRVAVNVFSHLHCLDLNYHLNRQTGALSKSIDRGSRGINFMLTSLVFNVVPTIFEVALVSSILYYRCGQFAVVTIGCISTFAVTQWRTKFRIQMNKSDQQAGSRSIDSLINYETVKYFNNEKHELKEYQVASLKTSTSLAALNFGQQAIFSACIMVLAAEMSVGDLVMVNGLLFQLSQPLNFLGTVYREVRQSLIDMQSMFGLLNIKSKPLALTPAVVFDNVTFRLVLDSMSFEVPSGKKIAIVGGSGCGKSTIVRLMYRFYDPTGGRVLVNGNDIRDVSLRRQIAVVPQDSVLFNETIEYNIHYGNFDEQVNEASSMAELHDTILRWYDTQVGERGLKLSGGEKQRVAIARAILKNSPILVFDEATSSLDSITEAKIMTALRAVENRTSVRIAHRLGTVADADTILVLEHGKIVERGHNELIGTLYAHLWHQQQN-MLRGARNLASAMAPQRIFNVPETQITELDNGMRVATEDSKLETCTVGVWIDAGSRYETPKTNGVAHFLEHMAFKGTSKRSQTELELEIENMGAHVNAYTSREQTVYYAKCLSKDVDKAVEILADILLNSKLGEAEIERERGVILREMQEVEQNLQEVVFDHLHTTAFQGTPLGMTILGPTENINSITRNDLLEYIQTHYKAPRMVLAGAGGIDHKKLVELGNKYFSGVTNTYDVITPCRWLISGCKFAHQRIPLGELEARIDAQRWDLLKNISVFVVLLVILLTIKSHLSGAKIHKLLVFVTGK-QFAEHINKLNKGNSDCALSGQHTLALIKAACFIISKQIELFLLMFTQKDQLAVYNAQNIVRVVACMLLLFAQIACVILLRACTCYNRHSMNAHAEADISQVRECLLSIVC--------PVLNTSTLYTNISRQHNNQS---FRDRHSALTACPIINNDICIKHLINVTPY---WEYINMDHEEHSESGRTLAFVRSNAKSSPPTSDVLNK-MTVAVRKPTQILKPTWHAPWKLYRVISGNSGWVHSLAVEPGNEWFASGSRDGLIKIWDLASGRLRLTLTGHISSVRGLAVSDRQPYLFSGADDKMVKCWDLEQNKVVRHYHGHLSGVYTLALHPTLNILVTGGRDSVARVWDMRTKAQIHCFTGHSNTVATVKCQSSEPQIISGSHDTTIRLWDLREARTHVTLTNHKKSVRSIAIHPEQNMFASGATDNIKEWSFPDGTFVQNLSGHESLINSLAINQDNVLVSAADNGSMFFWDWRTGYNFQRLLSRPQPGSIESEAGIFAVTFDQSGSRIITGETDKSIKIYQVEQRLTKHVKKLNARLNVVTGSTSDYRIDVNKQDYLEKLGVNVKARNFLVYQGAVESIAMKNPREITSLFEEISHSMDRSEEELFVKKKGITAEKKEAQGEEAKRYQNTRNEYQIDLQLFKLYHIDKEMARRRKKTAIDDDIKAKRQIDKNIRDELNLNKKRPSYIKAKENASHIEKKLESARKSLAAAQKAHANHEDIKELEQDLERVNKKFLVEDVELQEEQKTEYNRLKKQAASMSAKYLKDLDSLEREQKADSDRHENEVGKQRELDHKLREREENLRRIEKLKDLIDFKQREREIIAKKELSQELNGISNLGDAKVDRHEEDRRRKKSEIVEHLKKIYPGVYDRLLNLCKPIHKRYNIAITKIMGKSMEAIVVDTEKTGRSCIQYLKDQMLEPETFLPLDYIHSKPIKERLRNIQSPPNVKLVFDVIKFEPQDVREAVLFATNNALVCETASDANKVAFELGDNQRYDAVSLDGTFYQKCGFISGGSLELERRARRWDEKEIHNMKHKKEKLAEDLKENVKKTRKEGDLMVITSQITGFESRLRYSKIAEIKDRMDAREVEINKIRSAMNRVEDDIFKDFCIQLGVDNIRQYEERQSKASQERERLLQYESEKNSIQSRLLYER--SKDTLEVEVEQHNLEEAKEAERREMKAIEEEMSKVEQLKNDKISQKTDYDKIEEQVTEKKRSLTTIQKEILAIQKNITNLECRMESKRTDRHAVFVHCKMESINLPLKQGSLQRAHQSDNIRPNFDLLELLAEIAFRKIQAPNMRADERLDSAKERLRETDSELNSLRKQAKEAFESVKQERECFDTVSQRVDSIYKQLTNNPSAQAFLVPENPEEPYLEGINYNCVAPGKRFQPMSNLSGGEKTVAALALLFAIHSYKPAPFFVLDEIDAALDNTNISKVARFIRQRTESAFQCIVISLKEEFYGHGDALIGVAPDPGDCTISRIYAVNLGLPDGYVLGGRQFAQEEDGGLTQNLLSVGLTN-----IITLVIE--LISIHYES--KITGHIEVVTLLNLKLAHDPHSSECRPRLCWNRRIIFYYPFSNRADSLHIWNVHSVLNVLHSLIDKSNVIDQLREYNAGRDPQAVAGVFGSHNIYKMLGYYSMIGLLRLHSLLGDYHQAIKVLENIDLNRQIMVRVLACQTATYYYVGFAYMIMKRYSDAIRTFTNMLAYLHRTKRTFQVELVDKQTDQMYVLLAMCLVLHPQRVDEGVTVVLREKYMARLQRGDINEFETCPKFLSPVQLKVFLEEVTQQLIIRSYLKLYKTMSIEKLANFLLWCFKHKKQSIVLDGELRSGSDVDFYIDKNMIHIADTKVARRYGDYFLRQVHKFDELYR------------------------------MGPPDPILGVTEAFKRDTNPKKINLGVGAYRDDNGKPFVLPSVKVAEERIFKAGLDHEYLPITGNANFCQAAATLAFGNDSHIITNKLNATVQGLSGTGSLTVGAAFLRDFHNYSKEVYMPAPTWGNHIPLFKRNGFNVKQYRYYDPKTCGFDFVGAMEDLNNMPEKSIVLLHACAHNPSGVDPKAEQWQEISHIIKKKKLFPFFDMAYQGFASGDIDRDAHALRMFIRDGHMVALAQSFAKNMGLYGQRIGAFTLTAKDQQEAERILSQLKIIIRPMYSNPPLHGSRIVETVLTDTNLRQQWLKDVKLMADRIISMRHALRDGLVSEGSTRDWSHVTETIGMFCYTGMNAEQVGRLWNEFSVYLTKDGRVSIAGITSKNVSYLAHSIHSVTKAMTTSITSAAHVMECFSIHLALLSPVFDIARISHFRNGDTVLYYFLYVIRLFMLLATPQCLFNFLGLISFNPFPGKVTLKHDDKTRPFICIRVVTRGLFADLIRNNVRRNLQTCIDSGLDNFVIEVVSDRDITLNDSQNVRLLVVPKDYRTSTGALYKARALQYALEDKVSQLNDGDYIVHLDEETLLTENVIYGILNFAKEGKYDSGQGLITYANEEVVNLITTLADCFRVADDLGKIRFQFRAFHRPLFGWKGSFIVNKYTVEKDVSFDHGPDGSIAEDCYFSMVAYKKGYKFQFIEGEMWEKSPFTVADLIKQRTRWLQGIFLVVHSRKIPIVNKFFLAVSLYAWMSVPLITCTLILGPIYPMPEIIWLNSITAFNGVVTMGMFLFGVMISFRIKRVGVSRLFVYLVGTIVILPLYVCIENIVVIWGLFSPKHKFYIVQKETVIASTAWLFHPD-----PKYIS----DSIFSTESCFKKNWSDHKHIHRKCK--IYNPWPNYVYTGDLRPYPRSALRTVPPNIQRPDYADNPEGRAISEEAIKDSTQIKCLNDEEIESMRVVCKLAREVLDEAAKVAVVGVTTDEIDRVVHEASIERDCYPSPLNYYKFPKSCCTSVNEVICHGIPDMRPLQDGDIVNVDITVYHKGFHGDLSETLLVGNVAEQYRKLVQVTYECLQKGIEIVKPGVKYREIGDVIQKHASQAGYSVVKSYCGHGINRLFHTAPKVPHYAKNKAIGIIKPGHTFTIEPMISEQSWRDTQWPDQWTAVTVDGKRSAQFEETLLATETGCEILTRRRSNGGQPYFMDYMTLLKMMKGQSKKRRHLLAQQLGISSGDNLSAILGTRVERTTT-------EQTYRYTGSSTFLKGTQSANPHNDYCQHYVDTGQRPQNFIRDIGLHDRFEEYPKLKELIRLKDELIARTATPPMYLKCDLRQFDFRELECKFDVILIEPPLEEYQRTQGVTNTDFWSWDEIMKLRIEEVAAPRSFIFLWCGSSDGLDLGRQCLRHWGFRRCEDICWIKTNATNGHTKNLEPRAIFQRTKEHCLMGIKGTVRRSTDVDFIHANVDIDLIISEEPKYGTCEKPEEIFHIIEHFCLGRRRLHVFSRDTILRPGWLSIGPDLTNSNFKAKLYNSYFNSPQDCLTGCTDRIEALRPKSPPPKATILTSSSAALIK--MQKLTIEHIKIPSKRILIRVDFNVPMKDGRITNNQRIVGALQTIKFCLENKAKSVVLMSHLGRPDGQVKPEYSLRPVADEVSRLLHRNVTFLCDCVGLDIEEVCKDPQDGSVILLENLRFHVEEEGKGVDEHGNKIKADPKSVEKFRKSLTSLGDIYINDAFGTAHRAHSSMVGINLPHKAAGFLMKAELDYFAKALNNPPRPFVAILGGAKVKDKIQLINNLLDRVNEMIIVGGMAFTFLKVLKGMDIGSSLFDSDGAAIVKDLMDKAAKMNVKMHLPVDFVTGDAFKEDAKVGEATVESGIPDGHMGLDCGKKSMQLFEEPLKRANIILWNGPCGVFEWDAFSHGTKAVMDMVVAATQRGAITIIGGGDTATCAAKFQTESKVSHVSTGGGASLELLEGKELPESKISVRMMRTFGDRPTAFQLEEGGEYFYIGSEVGNYMRMFRGSLYKKYPSLWRRMVSVEERKKISSLGLGAHTIATNVTLLKATEVDEIFAGKDERYKAISISSEPSASRVDRDKRINWAASLPTSSHHLDAVPCSTAIARNRLTHKRVRTFPMIYDDLDPKTLTQIANVTEVLVPIRLDMEIEGHKLRDTFVWNKNEISITPEQFAEILCDDLDYPPQAFVPAIAQSIRQQIEAFPTESILDQQTDQRVLIKLNIHVGNISLVDQFEWDLSEKNCTPEQFALKLCAELGLGGEFVTAIAYSIRGQLSWHQKTYAFSEAPLPALDFPFRPNNDADQYAPFLETLTDAEMEKKIRDQDRNTRPTARLYLCHVMGSNASQLEREIGFPQNEHYFGLINFGNTCYCNSVLQALYFCQPFREKVLEYKLKSKRTKETLLTCLADLFHTIANQKKKTGSYAPKKFINRLRKENEVFDNYMQQDAHEFLNYLLNTIADLLRGEKGWVHEIFQGTLTNETRCLNCESMSSKDEDFIDLSVDVEQNTSLTHCLRVFSKTETLGAEHKYYCEKCCSKQEAQKCMRIKKPPMILALHLKRFKYMESQNRHTKLTSRVVFPLELRLFNKTSNSDSDDRLYDLVSVVIHCGFGPNRGHYISIVKSFGFWLLFDDDYVDKIDASSLEDFYGLTNDTQKTSESAYILFYQSRD??????????????????????????????????????????????????????????????????????????????????????????????????????????????????????????????????????????????????????????????????????????????????????????????????????????????????????????????????????????????????????????????????????????????????????????????????????????????LRNVA---------QVVESAAEQPYQARLSVTHSNTQDLNQLCKL-------IQIHSSRKVVQ-YEATVDAMDDGMLKSAEP--------AKTANLPLLPELDVYFNLLVVVNLIDLGRYEIAVRCSNQLMGMIVSQNRRTLDAMAARCYYYHTRCYELIGQLNQIKSFLHSRLRTATLRNDYEGQAVLLNCLLRLYLNCNLYDQAAKLVSKSVFPEAASNNEWARFLYYLGSIKALQLEYSEAHKNLLQAIRKAPQTGAIGFKQTVHKLAIIVELLMGEIPDRSLFREPTLRRSLAPYFQLTQAVRSGDLTRFGVVIDRYGSKFQTDSTFPLIIRLRHNVIKTGVRMINLSYSRIHLADIAKKLKSDFTEDAVFIAGKAIRDGVIEAKINHENGYLQSKETTDVYCTPEPQAAFHQRIAFCLDIYNQSVKAMRFPLKSYNVDPESAEERPQIETISAEHP--QENS--??????????????????????????????????????????????????????????????????????????????????????????????????????????????????????????????????????????????????????????????????????????????????????????????????????????????????????????????????????????????????????????????????????????????????????????????????????????????????????????????????????????????????????????????????????????????????????????????????????????????????????????????????????????????????????????????????????????????????????????????????????????????????????????????????????????????????????????????????????????????????????????????????????????????????????????????????????????????????RQHCRW-ATLENVELCIPLVFKAQLFALTGVPTDRQKLMFKGSVIKDDAWGCMFMLMGSNEKLPEPVVKPKFVEDMSEHELATALDMPSGLTNLGNTCYMSATVQCLKTVPALRELRRYQGNVTASLRDLYTSMDSS-VEPVILLSVLQSAIPRFAQKAEGGQQDANECWTELTRMLQNFIDEYFGGTFVEMKCDEAPEESNENFLQLSCFIS-QDIKYMQSGLISRM-KETLTKSSPTLGRDASYKTSRISRLPAYLTVQFVRFFYKGNVQTNAKMLRDVKFSMNLDVYELCTPALQEKLAPMRQKYKEADDHYSFVDDGSNNSGFYELKAVLTHKGRSSSSGHYVAWIRRQWFQCDDDRVVDEEEILKLSGGGDWHCAYVLLYGPKLI---INQVLPRELILKIFSYMDIVTLCRCAQVSKYWNQLALDGDNWQDVSLFDFRAGVKGRVVEYLSSRCGNFLKRLTLRGCRSVTDSSIDIFANNCRNLEEILDDCKQLTDRSCLSLANSLNIASC-EVTDESLIALGTNCKNLQHIDISGCNKITGAGIRALADGCPKLRSFISIACVNNESLQYLASQLRTINLNACSSITDEAVIALSENCDDIVNCCLSKCTNIADQSLIALSQHCLKTLGLIGCNLLTDAGFQALTRGCKYLENLDLEGCVQITDQTLYYLTLNCLKLKRLVLSYCEFITDEGIKHLGASFLELDNCPQLSDVAIGHLANCLKRLDIYDNQMITRQAIQLLL--VSIHNVHST--------------GSRGATVCCPRHPTANLIEDHRAGDMICPECGLVVGDRVVDVGSEWRTFSNERGNTDPSRVGAAENPLMGGNDLSTMIGRTGSAGDEVGNPRYSNRRTMSSSDRTLQTAYRDISNMADRLGMPRMLVDRASETFKKVHESKALRGRSNQAIAAASLYIACRQEGVPRTLAEIRSASDVLKRDVGRCFKLIMGLLGTSVDMISTSDYMSRFCSNLGLPTYVQRAACIISERATQRDINAGRSPVSLAAAAIYMASQASDVKKSQKEISDVAGVAEQTIRQAYKAMFPRAGELFPDDFRPHSKMAWVEKFRPKEFKDIIGNKEAISRLEVFSREGNLPNIILSGPPGCGKTTTMLCLARKILGDQMKDAVLELNASNDRGLEVVRNKIKMFAQTKVTLPAGKQKLIILDEADSMTEGAQQALRRIIELYSKTTRFAFACNTFDKMIEPIQSRCAVVRFTRIPDNEIRSKIIEICKMMGVKYDGSGIDALLHTAQGDMRQAINNLQSTYDGFGEVTSDNVFKVCDEPPPIMIKDIINHCLQGKLREAEESLVLLYKRGYCSEDLISSIFRVVKAYKTEEAFIIELFIHYGNTLLTILTVVQSVAVFAIFSTRLPKKILNLMTPKREHTLTKPYQGAGMTIPNWEFFGSTIVTNNYIRLTQDAQSRQGGIWNSMPWQVEVGFRIHGQGTELYGDGFAIWYVRDPPQVGPVFGNQDFTGMGIFFDTYANQQGVHSHGHPYISGMINNGTMHYDHDMDGTHSELAGCECKFRGSDHETRALITYYNDEITIKIDVEGDNEWHDCFKVTGVYLPPYYYFGITAATGDLSDNHDILYVKAAMLSAPKDDDLPKAAPPSERLESGMPALRRFFMIVCIGAAIIAFLYTSQLRNTRKRLYGNQDNFDDIMEKNCNVLDRNSLFLPESSVSHIPDIKQLGIDPTYENRTALLHIHNTALGRAIFFSYLLQKAFDAEAGLMYYYLSTNADVAASRYANASAIYFSPNRAFTPSYNGFFNKTMPLFAPRAFRADDFNDPYQLRGTSTLNSVIEHDHGAIDSNYTTEFYKINEWYGTWLPDMTRRHDSKPTYSVQITGHNETFTFHGPAGASDTPGPVRWTRPYYDCGRSNKWLVAAHTPIADLYPRHTGWRHIELPLYVAVAVVEIDYERLDINQCPLSDGN-PSPNHFHDTAKCVKDTTMCEPISGYGLRRGGYQCRCRPGYRLPRHVKSPYLGEIVERATDDDRRNSFKCEKIDNLAVKTQNVQTLTGLSSRMDASLPGSVAHGKEHQFEIQSRAALRLSHFISSFIQLVDPKEVFAEFRVPDRALTKDQVIGEALSTMMADRSIQGLGVWFDRNQFASPFFDTHKCDRKSSR--VMNSNQIR-KFHSILILFSCFTLQRSSPSLSCLSVVAHDFNLIHKTWHVYFSHIYMNWLNRTTITHLKLTCDTAALSQLEIDVIDDFVNCRVCHLLHGSQVIIIGSKLIIDFQAKCHVKMNYIRLLYVCLFGGII---PQLTAVI------LKAKYGGRFMVTMLPGDGIGPEMMRHVKTIFKVGGMPVDFEEINLDSMNENIEKVEEAITSIKRNGVAIKGNIETREHSKYFKSRNVELRTRLNLYANVVHIRSQPGVATRHHDLDLVLIRQNTEGEYSSIEHETVTGVVECLKVVTRAKSEQIAHYAFNFAIKHGRKKVTCVHKANIMKLSDGLFLDVARSVAKSYPEIEFEDIIIDNCSMQLVSNPHQFDVLLLPNLYGAVLTNIACGLVGGPGLISGANYGDEYAVFETGSRNTGKTIAGKNIANPIAVFNASADLLQHLKLENYASLMRGAVHKALNESKVHTPDLHGIHTTTDVVNLIIDEIRQQT------MDEYQLNNPPTDTIQSVKFSPHNGRHLLVASWDSAVRLYDVESNIVKARYNHSEPVLDIAYEGSEAFWSGSVDKTVKRYDISTQTATTVGSHTDAVRCVEHIPDLNLMVSGGWDSQLKFWDPRQGKGAALSSQVDKVHTLDVAGHRVLVGTASRRVIIWDARTHKFVQ--RESPLKYQTRCIRAFPDGQGYVLSSIEGRVAVEYLDPDPEVQKQKYAFKCHRNKDEGEMVVIYPVNAVSFHRKHGTFATGGSDGYVNIWDAQRKKRLVQFRKYPTTISSLSFSCDGTMIAIASSYLYENEEINEIPPDAIYIRRVSDNETKMVSSLEQLRKLSVVVADTGDFELMKKYKPTDATTNPSLILQAAQKEEYSGIIDKALEYGRQNGRSLEATMDYLCVLFGCEILKIIPGRVSTEVDARLSFNTQGSVNKALKLIQLYKDHGVDKERVLIKLASTWEGIQAAKILEQQHGIHCNMTLLFSMAQAIACAEVGATLISPFVGRIYDYY----KVKSYEPFDDPGVKSVTKIYNYYKRHDYKTVVMGASFRNISEIKCLAGCDLLTISPALLGELAEKGVTLNKELDVAKAKSLDLEKRTYDAARFAWEMNEDEMAHFKLAEGIRKFAQDQTRLETIIKAKL-MPTACVRPLVTVYTDKNQKSGKTVKMPAVFSAPIRTDIVQYVHTNIRKNSRQPYSVSKLAGHQTSAESWGTGRAVARIPRVRGGGTHRSGQGAYGNMCRGGRRFAPTRVWRRWHRKVNRNQRRYAVVSAVAATGVPSIVQSRGHRVENIAEFPFVVSDKVQELNKTKQAVKFLRQVRAWKDIERVYKSKRMRAGKGKMRGRRRVKGLGPVIIYSRDSGLTRAFRNIPGVETINVEKLSLLRLAPGGQIGRFCIWTEGAFRKLDRIFGTWTKKSMLKRDYMLPQPTMANTDIARILKSDEIKKAIRPRRSRLPKRCIKRNPLKNIRVMARLNPYAPVLKKVRKIETAHRKAKKIEKAKKLHKAEARKKDKKKQRIVKKDMLKNGQIIKKILDAVKDLVTEATWECSPNGLSLQAMDTSHVSLVTAQLKSEGFDHYRCDKPLNLGMNLPHLTKIIKCAGNDDTITIKAIDECDKITLLFEDKNETETSEYEMKLINLDSEYLGIPEQDSEVEVEMPSSKFARICKDLSSMGEAVTISCVKESVRFQVQGDLGSGSVNLVQKSSADKPDEAVCIKMVNPICLSFSLKYLNHFSKAAPLSPRVKLLLKAEAPLVVSFEIGFIRYYLAPKIEDVQ------------------------MSLVSKVAVLGACGGIGQPLSLLLKTSPMISHLALYDVANTAGVAADLSHINTKAKVSAHQGPAQLADCVKDASVVVIPAGVPRKPGMTRDDLFNTNASIVKDLCKVCAQIAPKAMLAIISNPVNSTLPIASEVYKKMGVYDPLRLFGVTTLDIVRANAFVAELKQLDPNQVNVPVVGGHAGTTIIPLLSQTTPSMQISKEQREPLIKRIQDAGTEVVKAKAGTGSATLSMAFAGARFTFSLLKAMAGENVVECSYVKSDVVGLSYFSTPIVLGKNGQEKNLGLGQINDFEKKMVEDAKAELKASIAKGEEFASK????????????????????????????????????????????????????????????????????????????????????????????????????????????????????????????????????????????????????????????????????????????????????????????????????????????????????????IPTFIGVLNELKPSLNDIKKAVDVCDVVFTEVEPSL--NSVVT-LLFTFCENVLKAPSESSCLR--VMKNLYDGLQYVAYITLVKLANNQLSEVFDSLASVKSKFGVESTQNLYRLLHSSLSKLASQVMIELLGTYTEENASHAKQDAMTCITSFLKDPNTFLMDHLLTLKPVKFLEGEPIHDLLTIFVSEKLQDYLEFYSKNIQVI-ENLGLVHEQNMKKMRLLTFMQMAESKKEISYETICEELQLEHDNVEPFVIDVLRTKLVRAKVDQLGRKVLVQSTMHRTFGRPQWQQLRDILNFTHVEQTIKTFLEYIGSTR-KKKTVEEVQEGKKAIDDNESRIIIDQSPQFKELVTTLANWINDELNDQRIIVSVKHLEHDLYDGQVLGKLVEKLTGTQLDVVEVTQNEDSQRRKLREVLETVNRIFNIKWSVEGIHAKNTVQIIHLLVTMIRHFRAPI--RLPPNVTVRCNVLSKQGEKLTETYNELGMRIEPRDAFDTLFDHAPEKLDVVKRSLCNFVNRHLKKINVEDLDPNQFSDGLLLIFLMASLENYFVPLGIFTTP-TDPIADMNGAQTALQHENYVNTSKLHNVNVALQLMEDADVKQRVRAEDIVNADLKSTLRVLYMIFSKFTAGPAKIPEDVMIQAQKELLSWGRSVLEMSHRSKEILTTTEKNLRALMKIPDNYTVLFLQGGAQAQFDSVPMNLCSAEYLVTGIWSSKASKEAQKYVNYERLPTEEEMPLCGCACYRYYCDNETIQGVEFPLVCDMTSNFLSRPVDVSKFGIIFASAQKNCGTSGLAIVIVRDDLIGKMSVTPSTQNYQIMRDNKSNYNTPPVYAIYLALCVKWILDKGGLGAMKISCEKSSLLYHTIDESKGFYSRSRMNVVFLI-GGSKGNAELEKKFLAEAEFHDLKGHRLVGGLRASLYHGVTLDDSK???????????????????????????????????????????????????????????????????????????????????????????????????????????????????????????????????????????????????????????????????????????????????????????????????????????????????????????????????LRRAKQIALLAIMCLILITITKALVVDRFDELLKFVPYISEFLHKQGITHKIYVMNQVDNLRFNRGALINAGFLVHAGQSDYIVMHDVDILPVNNNLSYAYPENGPFHIASPEYHPQYNYARYIGGILSISNEHFRLVNGFSNRYYGWGLEDDEFFTRLTSKDLRIARPTRLQTNKTNTFIHMHR---RPRDKSRLFNQKEATSKRDRQNEWHVLPHKDDFLYPQSVQSIAFAVSSAFFTGQLFETFWCFQSVALPGDLIIENGYMRGHGELVSSVAGRVERINKLVSVHAPRARFVGETGDVVVGRIIEVQVSQKRWKVETKSRLDSILLLASINLPGGELRRKTAEDELMMRKYFKEGDLIVAEVQSIFQDGALSLHTRSLKYGRVGQGILIKVPPSLIERRKLFHNLNTLGVHLILANNGFVWISEHGFDRRAVARMRNCIEILCFAKIMINDKSCTVCFEES-IVTLSHECNPKHVGVWLLGVSGITAFTVSLGGITRLTKSGLSMVDWHPVLESPPRTTDWNAEFDKYKQYPEYQMRNFDISLDEFKRIWWMEYIHRTFGRVIGISFFVPAAYFAYKGYFKGRTRYVIVALGGLILFQGGLGWYMVKSGLEQPPRVSHLRLASHLGTAFTFFSISFLTALHYLLPNVQLLRYKKLTGSVFVTALSGALVAGLGAGLTYNSYPKMADRWIPTDLFAKPVWRNFLENSTTVQFDHRWLGQITASGCWLWSLRLPLTPRLRVVSNVVMMLCCQLSLGIATLLLYVPKHLAATHQAGALGLLTLSLWLAHELKYVKK-V????????????????????????????????????????????????????????????????????????????????????????????????????????????????????????????????????????????????????????????????????????????????????????????????????????????????????????????????????????????????????????????????????????????????????????????????MQAKLKIQWAGFITRAPIYPYLELVHIVSRFMQVRAHLG--SLVVSRRQPFACWLVCMASIFGAPMLALTLINEPPVGALQASQHVMLASISWYLIFYTPLDLFHRCVAWAPCRLALALANELHRCKRIHESIVHTSKIYPTSYFVIVLVAYVRANTPSLSPLLAKTL-GLPVPTAMSIHEPEVAPKISLLAALVFLIERRTTWLSAPHSLVFTCTALVMIYVRLTLALGVTGSPLASLEQMLRSLVFGGFRTVTHATVSSVG----------------------KEMLSNALSVKTNLPLRDQKDYGSNST-------DLTGEAITFMEDVLILG--SSASYTTFGQKCRTSGLPGRLTSSVALR-VIELENTNMRRRCLSLRKVIVKNNAKTGEVLLDETLRHISTTEPPLSVQAWIEYLSGETWNPLKLKYQLKNVRERLAKNLVEKKVLTTDKTNWVIFDMTTHPLVNSTIKNKLIRKVQDALLRNWINIPQRMDKRILSLLLLAHYSDVLENAFGPLSDEEYETVVNRLNTLTSLDMEAESQKPDTNESIWAVFSYFNKMQPPIAKYKCVLVGDGGTGKTTFVKRHKTGEFEKKYIATLGVEVHPLVFFTRYGPIEFSVWDTAGQEKFGGLRDGYYIQSQAAIIMFDVTSRVTYKNVPNWHRDLVRVCDNIPIVLCGNKVDVKDRKVKAKAIVFHRKKNLQYYDISAKSNYNFEKPFLYLARKLMGDASLEFVAMPALQPPEITMDPQWTARLEQEMQQAQDVPLPDDDDEDLVAEYFTPVLRSSKFKQTGVLTPEEFVAAGDHLIHHCPTWTWSSSSEKSYLPSDKQYLVTKSVPCYKRCKDIDEKVIMTDEEGWVDTFDYDDDDDEAMDMDKFLEDHDDAVNIIATRVYDLNITYDKYYQTPRLWLVGYDEHHKPLTIEKMYEDISQDHRKKTVTMENHPHIGR-VMASVHPCRHAEVMKRLISTVEDGGGLQVHQYLIVFLKFVQSVIPTIEYDYTQNFTIISRYDPVMPYGKLQNNLAQVRKKLDRPLTLAEKILCPHLDDIKNQDIKRGESYLRLRPDRVAMQDATAQMAILQFISSGLPKVCVPSSIHCDHLIEAKSGASSDLSKAKEVNKEVYEFLRTAAAKYGIGFWHPGSGIIHQIIFENYAFPGCLIIGTDSHTPNGGGLGGLCIGVGGADAVDVMANLPWEVKCPNVIGVHLTGQLNGWASFKDVILKVAGILTVKGGTGAIIEYFGPGVDHISCTGMGTICNMGAEIGATTSVFPYNGMMRDYLVATGRQAIADMADENLSLLTPDKNAKYDQVIEINLSTLEPHINGPFTPDLATPISKLAEAAKKNNWPMDIKVGLIGSCTNSSYGDMNRSVAVAKQAIEHGLKTKSAFTVTPGSEQIRATIERDGQADTLRKFGAMVLANACGPCIGQWNRQDTKKGEVNTIVTSYNRNFTGRNDANPKTHAFITSPEMVTALSIVGRLDFNPLTDHLTGAKGEKFKLEPPTGEELPSRGFDPGQDTYQPPAEDGSNVTVNVDPASNRLQLLQPFDKWDGRDLTNMAILIKVKGKCTTDHISMAGPWLKFRGHLDNISNNMFIGAVNAENGEVNKVRNQLTKEFGSVPDTARYYKSQGIKWVAIGEENYGEGQ------------------------------------------------------------------------MFRNTFQSGFLSILYSIGSQPLQLYDTKVRNGSIKRITDDEIKSLVIEITSSNVSTTFITCPKGLERNLGIKLPIFVMIMKGLNRYFTFEIEILDDTKTKRRFRASNFQSETKVRDNICTMPLRLEHGWNQITLNLPDYCRRAYCTNYVETVRLTVNANCKLRRIYFCDKVYPEEELPAEFKLCLPVT--------------------RLLQHAKYRQIEHTRKDILNAIKYYRTLLEKYTFPDGVSKELICLDGTIPVTFRYNIPVGIWLSDSHPFVAPICYVRPTRDMTIKQSRHVDGSGRIYLPYLSDWNCAT-SDILSTIQVMQIVFGQTPPVYQT-QRPLP--NTGTITEEHIRASLLSAVGDKVKARLREQIQAEIEVLKKTSNDLNRGKMQLDEMETRMAKEVDLNDSLDAQLQEIDVDNIYGPTQPLYRQLLNAFAEENAVVDAIYYTGEGLRKGVISLDVFLKNVRELSRRQFMLRALMQACRAKANLPMKRKKHFYATDKGYTYRESIEEVVVRVEDSLSNADVVVSCPAPYLEHTHKSLPNYILVAAQNCYKANSGAFTGEISPVMISDCGAKWVIIGHSERRQLFKESDLLIAEKIAFAIENQLSVIACIGETLQERESGHTFDVVHRQLKAIVDKVPEERNCNMILAPDYAIIRGNYDEP-EAKEMISTPQKFDFKNSSPHVRRKQESIFSKSMMTASCQQVLKSLAEVVVVESSSSLSCHFVIPIQAHSMASRLSQKKKVVASGMFKAEIDEFLRRELAEDGYSGVEIRKNASRTEIIISATRTQQVLGDKSRRIRELTSVVSKRFNCEDGTVEMYAEKVNDRGLCAVAQCESLRYKLVGGLAVRRACYGVLRYIIESGAKGCEVVVSGKLRGQRAKSMKFVDGYMIHSGEPTNHYVDTAVRHVLLRQGVLGIKVKIMLPNDPTGKQGPKKPLPDTIHVIEPKEEVMPIVPSASNVDGMVTYDEMHDVDIEDPTALSANRSPMDSLKEWSVSTFKCTKQRLEEKLGKCSRTFDGPLEAEIERLRENQRKYTMMLNTARSMSIQYSLVHTQRQLCELMNEMALKYTAGAV-NLSEDFRKNALVLSIAAKNGEKLCQALSFFCANLSTLVHKTIEDTLFTIRAFEAARLEYDAERN--SDKLKSRYEQLREDVLIKIRFLDENKFKVMHKQLVLFNSAFASY-TSGNTAALEAALKQFKI-----MSDEATATADIRLWVFLPIVIITFLIGVLRNYVTILLMSTKKLELQQVQDSQALIRARLLRENGKYLPKNSYLMRRHFFNS-EHGYLTVAKNRPSVQPNPMTDPSMMTDMLKGNLTNMLPTILIGGWINWTFSGFVTTRVPFPLTLRFKPMLQRGIELASLNSSWVSSASWYFLNVFGLRGIYTLVLGENNSADTTRMMQDQMSGAALSMPADPKPAFKSEWEMLEIHEHHFAL-----------------------MNSSTSGPSSSRRCKSRDAKRRRA-SQTETLKKKIEEETKVADIGGRFATHHDAIEAHLRASTVGLVTLDEMRIRQEEAAKERAQQLVQLQEEKNARPSVDPKELQEKHRRQRQALSFQFDDDEDDEDDEEDGSETLQNKQDDSTDASICATDGTIKKDTIARIDSDSNSRTSLQPKKKKKMLKNPDVDTSFLPDREREEEERRLREELRLQWQERQEKLKEEEIEITFSYWDGSGHRRVVKMKKGHSIYQFLQKCLEQLRREFNELRSISADQLMYVKEDLIIPHRYTFYDFIVTKARGKSGPLFSFDVHDDVRLKTDATVEKDESHAGKVLLRSWYERNKHIFPASRWEAYDPTKTYDKYTISDKKGRMRNPAAVKKVPTVPESVLKKRQLRKTIKEAQRVKAQKRQKERRVKHTKIFKRAEKYVKEYRLRERDQIRLKRQAKKHGNFYVPPEPKLAFVIRIKGINGVSPKPRKVLQLLRLRQINNGVFVKLNKATVNMLRIAEPFITWGYPSLKMVRELVYKRGFGRLNGQRVPLTSNEYIEKRLKRYDIICMEDLIHEIYTVGPHFKQANNFLWHFKLNNPRHGWNKKTTHYVEGGDAGNREDKINDLLKKMVCRGSAEQLWKAVTGVSNAGKKRGRGRGAGRRQVKDFNRGQMIGVGRKTLIMPGLNAPVVKAKQMVEPQLVGENTEFRDNLSKMRNEMNIFKKYRQHPMERGWSGNKAAGKHMGPPDPVAGTPFENFESTVLMLRPLVMMRGPLGRTKEMNALVVTGNGNGLAGFSTSTAKDGRAAVRNARNRAGQALVYIERGDGTVLHDFFSRYYYTTIFVERKPKGYGIKAHRVIKAICEAFGITDLYAKCEGASDNTINITKAFFLGLMNQRPYQDMADEKKLHLVELREENNYYPRILASPRDARTERDIAPGEMLDFTYFIYNGKIRQVTRKNPPLYVGSAGWQVHLNRMDFVKNREKTKLALAAKYDDFEVFPGFKAIKRPEQA-----QSRMAPKTRVLVLCSRGVTHQDRHLMLDMRKLIPHSKKECKFD--KKDLNELSIIDNCDKCIYFENRKGKLLFMWASNVTGGPTVKFLVQGVHTMSDLKFVGNCLKGSRPILTFDSNFDKLPLIKELFIQIFNVPFKHPKSQPFVDRVVTFSYLDNHIWFRNYQIIDEAEISLAEIGPRMILLPLLALSSSFHGRILWANVKAVLPIKNKKV----------DNDVDEDSEVDDD????????????????????????????????????????????????????????????????????????????????????????????????????????????????????????????????????????????????????????????????????????????????????????????????????????????????????????????????????????????????????????????????????VIVGILGGTGLDQDSSILSDETPYGRASDTQAISGVIEGVKVYILARHGKSHDRSPSHVNYRANLWTLVQQLNCTHILVTSACGSLQEHIEPGHAILDQYIDRTRDRSFFVCHIAQGRPIMRDILIEHKDLCAVTIEGPRFSTLAESLLHKSWGCHVVNMTSVPEVQLAAELGVFYGCLLLITDYDCWK-EGEECVSAEMVDRMKSLRSTAVKIIPRAIKKIVKEAMM--------------------------MTVLRHEVFDSGCEAACNGPYDNKWSKTMIGYGPEDDYFVLELTYNYSVGKYNLGNDINYLKIKAADLFNKIKESEE-SQESDETLELVSPDGYKFIVEQLEE-GKKTDVTQVCLSCIDLNKSKNYWINLLKCELYSESERELVFGYSDKQTSLKLIKIDSELNHATAYGRIAFSCPASELKSIEALVADNKHTILTPYISLDTPGKASVEVVILADPDGHEICFVGDEGFRELSKVDPDANKLIDQSIADDKSAEWFAKKKKFKKDA??????????????????????????????????????????????????????????????????????????????????????????????????????????????????????????????????????????????????????????????????????????????????????????????????????????????????????????????????????????????????????????????????????????????????????--MSQLSKFLGKLCRSS-HNATRSI-GTIPIVIEQTGRGERAYDIYSRLLKERIICVMGPIDDHLSSLVVAQLLFLQSESSKKPIHMYINSPGGLVTAGLGIYDTMQYILPPVATWCVGQACSMASLLLAAGTPNMRHSLPNSRIMIHQPSGAASGQATDIQIHAEEILYLKKRLNGIYEKHTKQPLEKIEAFMERDKFMNTEQAKEFGLIDVIVERPPAVVKENAT????????????????????????????????????????????????????????????????????????????????????????????????????????????????????????????????????????????????????????????????????????????????????????????????????????????????????????????????????FFRIVKDLKHGTSITLLPSEPALVEGDKTLYIRGYTGNKLTTNVMKDIYSLQKPLSSFLNRKNPFDDASTIEYLQKNESPLFMFSAHSKKRPNNLVMGRTFGGQILDMIELGIDSYKSISEFKNLKISLGSKPVLVFAGEAFEDMIRIKNLLNDFFCGTSIRVAGIEHTIQFIAHGIHMRVYLVEQKKTGSSSKSIVELTEMGPRLELSVRRTRIASFDHFKLACKQQPKVKSKKTRNVSKDAFGATRGKLHMQRQALSLHHLEVVSLSIHPIKALQDNYMYLLVSREAAAVDPVNASAMTDAIQADLKAILTTHHHYDHANGNAELLVYGGDARVQALTKHGD-KIKVGKIITCLSTPCHTKGHICYYVVFTGDTLFIAGCGRFFEGTADQMNRNLLGNLPDETLVYCGHEYTVNNLKFAIHVEPRNNIKRKLDWAEREPTVPSTIGEEKKINPFMRLTVKRFTDENEVMSVLRVKKNQFHMNRLFGRSKPTAPGPSILDVIQSVDQRVEQFDKKIAMLDKELLKYREQMNKMRDGPGKQSVQQKAIRVLKQKKMYESQRENLIQQSFNMEQTNFATQMLKETKTTVDAMRAGVKQMKQEYKNVNIDDIESVQDDLEEMLADANEVQEVLGRTYGVPDVDESELEAELEALGEELQQDDTSFLDEVKAPLPTTKIPYKSRDDICKQLLDLLKGPSRPSL-GTISGVRAKHSLPDLPYDYNALEPTISAEIMKLHHGKHHAAYVNNLNIAEEKLSEAMQKNNVSQIIELQKAIVFNGGGHVNHSIFWKNLSPSGGGDPSGALLNAINSSFASVDDMRQRVSSTAVALQGAGWAWLAYCPQTKKLTVRGMPNQDPLHPLTGLVPLFGIDVWEHAYYLQYKNARPDYVKAIWNIVNWKDVEERFIQAQEAPIKQDLPSDIPSLKQELETRRQNIEKKLKHVEHACIADYMKEGNNISDLHQRVICCDQILERLETMLCKFQADLGNICQDILSLQDQSVSLSTQLQNKRAVREKLGQFIDDMTIPQPVINHIMNAPTNEALFMEHLQILDQKINFFKEQDFRDAKACNDIHETLMSLKSRAVSKVREYILKKIQGCKKCLSNYQIPQNSLLKNKFFYRFLLTHEREKAREIQAEYVDTMSKVYFSYFKEYLARISKLDYDDKPDEHDLMGGDDQSNLTLFSKTSIKNRSTVFSMGQRASVINTDLEAPLILPTMQQKSDTKYPSECLFRSIQYAIVDNACREYQFLCEFFMVSDGQAADLFHSVFGKTLAIVHAYVVEQFKMSYDTIAMFLCLHVIYRYRRLALKQNVPVLESYWDSLVKCLWPRFDKVFRMHIDSVKNCDPQRIGPVDTLPHPVTRRYAEYACSMAAVNNTFPDERVMFLLSSLQNEVKNFILRTAAIFVHPKEQLIFMINNYDHILSVFKRAVKEDSKDIEDIKLLISKRTQEIVEELLYPHFGSIICFVKDSEVYLERNDQESLKRNEPKVTTLVKSFNSDWRKALDEISHEIMGSFSNFENGNNIQQAVLTQIIQYYHRLQKIVALSPFKNNPVRNELIDTHQLMVDLKKYKTNFMSEPPLLLSLVVVYTIQPGDALMQTIDAHAEECFFERAEAGTKLGFTFEVIDGGFLDIDIHIRDPEGRILHQEERASSGKYTIEATTTGAYDYCFSNKMSTMTPKVVMFSIEKSDGTH---SKPGDAEHAKLQSMVQTLVYSGVSVKRELEYMAVRDRIHRRINEETNSRVLTWSLFEFVLFLTASVAQVVYLKRFFEVKRVIMRLTQLLALPPSYHNMPQRYVDRNTKFIEYRAPKLPHYVRKVIKYRYDIWRPWQREFQENRAGSKIYVEPIFRGDRVELMTGPDKGKQGYINYIVKQRNWVCVEGLNLEEKPLLINHEVKLVDPTDLLPTDIEWRFDDQGKRVRVSSRTQRVIPIPRAFETIDYVYRDQPKDTDADEAAKITFKP-RAMTFEMEICEQVGIKDDRVPYPMYWYMSSNGGT-VIDHVSRNKIDLALFFTRQCTLLFTIYYLVGVTNNHYKRALLANAATSALRMHQRMPTVQLSRQFLVNLMHEDSAHYLFFSVIF-LPVS-LALLPIALFSMLNTMTIMDKHGAFIE---KQQSILQTIALCEIVLQLVCVVGVLSRLFLVTPVLYYRFLLLRYESRRNGHTRLFNREHNTHTHTFLWKLFETMIPKIISVSVVSQ????????????????????????????????????????????????????????????????????????????????????????????????????????????????????????????????????????????????????????????????????????????????????????????????????????????LAGKDKLDRIAIVNNDRCKPKRCNQECKRSCPIVKSGKLCIEVTTNDKIARISEELCIGCGICIKRCPFEAISIINLPSNLEKDTTHRYGPNSFKLHRLPIPRPGQVLGLVGTNGIGKSTALKILAGKLKPNLGRFSDPPDWTDILAHFRGSELQNYFTKILEDELKAVIKPQYVDVIPKALKGTVQANLDRKNDTGRLKEICDILDLNEIRSREVDQLSGGELQRFAIALVCIQNADVYMFDEPSSYLDVKQRLQAAQAIRSLIAPEKYVIVVEHDLSVLDYLSDFTCCLYGVPSAYGVVTMPFSVREGINIFLDGFVPTENLRFRDVALVFKVAESAQEEIKRLCHYEYPRMDKGLGGFHLDIEPGTFTDSEIIVMLGENGTGKTTFIRMLAGALKPDGDSELPTLNISYKPQKISPKSQKTVKELLMEKISDLFRHPQFQTDVVKPLQIDQILDQNVADLSGGELQRVALVLCLGKPADVYLIDEPSAYLDSEQRLVAAKVIKRFLLHSKKTGFVVEHDFIMATYLADRVIVFEGEPGVEAKACSPQSLLTGMNKFLSMLHITFRRDPNNFRPRINKMNSLKDVDQKRSGNFFFLEEEKSVHSLMKPPLKTVDAVEAELRERDRGRLRRGLETIDNLEDTRGMSNKEWVSQATRNEIKNRFKHLLKTFREKLRAMEQNQQSFELEYILAREQQALALFLPEVPHQMLELFNSASKEVVLSIYPNYTKIAPEIFVRIKDLAITDEIRSLRQLHLSQLIRTEGVVTSSTSIIPQLSLIKFDCLKCKYVLGPFIQTQEVKPGTCPGCQSLGPFAINMEETIFKNYQRLTTQESPGRISAGRVPRAKDVIVLGDLCDSCRPGDEIDLTGIYSNTYDGSLNIANGFPVFSTVIMANNIIRKEVDEDISRIVNLSKDPIFERIMASICPSVYGHKPVKRAIALALFGGVAKTQE-KHRVRGDINVLLCGDPGTAKSQFLKYTSKIAPRAVYTTGQGASAVGLTAYVSPVTREWTLEAGALVLADNGVCLIDEFDKMSDRDRTSIHEAMEQQTISVSKAGIVASLKARCSVIAAANPIGGRYNTFSQNVELTEPIISRFDIICVVRDQLLTKFVVRSHIKSHPIRQDLLRKYIVYARDKIKPVLQQDQIAKVYADMRRESAMTNSIPITARHIESIIRCSEAYARMHLRNYVDVRMAIKVTLESFVTQRAMVRKMTVLEDIENAFGKSNLYEVLGIDKHASNEQIKKAYRRMSLKVHPDRVSEEKKVEFTKKFQILAQVHYVLTDDERRKMYDEHGIIMNEDSLSGEADWSQYWRVLFPKVTKKDIESFIQNYQGSQDEKDDLSQLYNRYEGDMDKISQCHIAFEEDRTRKLIEEMIELGQVPALEKFINEPESKREKRRKKAAREAKQAEKVRKKAEGGIDDLVAMIRGKRQDSFDAMMASLESKYAKKET-KGKKRKRH????????????????????????????????????????????????????????????????????????????????????????????????????????????????????????????????????????????????????????????????????????????????????????????????????????????DASRVNKAISQGNANHLRDQL--------------------VSSSSKLHKSYSEYLSNVKTGVARLEQAINNIKEVNLEDVDKTFEVPKLDKLNGIREESFRYSQLCTARDNFKHIFDVPANVATTRQYIREGKLLMAHHLSELENSRSALLYEIHRHPKSSSSDRTTLKHYFSEVDKLSEELEKQILNIMSRALNTVRQDPHTLVSALRIIEREEKFSIKRQAAT-GFMPQGRPKLLRKKMFENIEENVGERLSGNQFEDRTNNKLWLARHLEIIRLLILEDLRVVKKGFVQCFPPSYNIAHLLKLYHRCLRTHLQEL-AHLEGNEYVTLLNWVKAYPGPELLGHPDLDIDLPLLSDTLNELTNAYFVTIEKNYTEWMGNTINDTRDWPETDDSGHYQTTTPVFIFQMIDQHLQVAKSFTLVNRVLALSMNQLAIFAKKYREAIEKYHFTDRQFTPYMVAITNNCSAFS-FEALRDDSIDYLLQELFLDVDKIATIGTENICLTLEDYFQDYLQNQLAKGYIRAIL--QRKITLRDYSQRKDFCKREVLKASPFDALKSLAGCLELKDSSLLSLEVSGVIAGLFIVLL---------------------------------QALSQEEDG------------------EKHEFQAEVTRMMKLIINSLYTNKEIFLRELISNASDALDKIRVLALTSKEALDSGSELEIRIKADDTTNTLHITDTGIGMTRQELINNLGTIAKSGTSEFLKQNLESSDQVQLNDLIGQFGVGFYSSFLVADKVVVSSKANDDPERHLWISNSSAYTVAVDDSEDKLKRGTTVTLYLKEEAKDFLKQDTLQELIKKYSQFINFPIYLWTSKTVQEEVPVEGD-----DEEDVEKNDDATVEEEEQNKKAKTKKVDKTVWEWKRINVAKPIWQRKPIEVTENEYNEFYKSITRDYQNPLARTHFTAEGELTFKSLLFVPKQQPSETFNKYGTKSDNIKLYVRRVFISDEFNDLMPNYLSFVRGVVDSDDLPLNVGRETLQQHKLLKVIKKKLVRKTLDMIKKIDESKYTEFWKEYSTNLKLGVIEDQSNRNRIAKLLRFHSSKTGADKWTSLSDYVKDMQSDQEQIYYIAGSSFDELSQSPFVESALKRGYEVLYLTDAVDEYCLSNLPEFDGKKFQNVAKEGLVLGKSKS-ESQKKAIESKFKPLVGWLKGTALKDKVQNVVLSERLSESPAALVAPTFGWTGNMERLAKSNAHSKSKDMTRDYYLSQKKILEINPHHPIIKELLKRVALDEGDSKARQTAELLFETATIRSGYMLQDTEEFGKRVEKLMRVNLDVPEDAPVEEETVEDDAIENNES--DDSSKDNDHEEL--GAEGYTMSAQQNTVLTIGEIIKELIAAHERNEDVNLTKLRFHVARKYKLAKFPRLIDIISAVPPQYRNILLPKLRAKPIRSASGIAVIAVMCKPHRCPHINYTGNICVYCPGGPDSDFEYSTQSYTGYEPTSMRAIRARYDPYLQSQGRIEQLQQLGHTVDKVEFIVMGGTFMSLPADYRDYFIRNLHDSLSGHHSQSVDEAVRMSEKSRTKCIGITIETRPDYCLNKHISDMLAYGCTRLEIGLQSIYEDVAVDTNRGHTVRSVCQSFQLGKDSGFKIVSHIMPNLPNVDLERDLEQFVELFANPAYRPDGLKIYPTLVIRGTGLYELWKTGRYKSYPPSTLIDLIAQILSLVPPWTRIYRIQRDIPMPLVSSGVEHGNIREMVLARMGDLGLKCRDVRTREVGIQEIHHKIRPYNIELIRRDYTANDGWETFLTYEDVEQDILIGLLRLRLCSIHTFRPELMQQTSIVREHVYGSTVPISTKDPTKFQHQGFGMLLMEEAERIAREEHGSQKLAVISGVGTRNYYRKMGYELEGPYMTK

>Brevipalpus_yothersi
[truncated: 553,319 more chars]
