# Supplementary material for: Symbiotic bacteria of the gall-inducing mite Fragariocoptes setiger (Eriophyoidea) and phylogenomic resolution of the eriophyoid position among Acari
Source: Sci Rep. 2022 Mar 9;12:3811. doi: 10.1038/s41598-022-07535-3 (PMC8907322; doi:10.1038/s41598-022-07535-3)
Supplement: Supplementary file 6 — Supplementary Information 4. [file 41598_2022_7535_MOESM6_ESM.docx]

#NEXUS

[Supplementary Data S3. Curated concatenated phylogenomic matrix for 27 chelicerate genomes. Divergent amino acid ends were manually trimmed and bacterial contaminants from GenBank sequences were removed. This is a nexus file generated by Mesquite v.3.61(build 927). This file was used to infer mites' phylogenetic relationships (Fig. 2)]

[written Sat May 09 20:03:32 GMT-05:00 2020 by Mesquite version 3.61 (build 927) at ummzx-pklimov-macpro-1e36/192.168.43.250]

BEGIN TAXA;

TITLE Taxa;

DIMENSIONS NTAX=27;

TAXLABELS

'Limulus_polyphemus' 'Ixodes_scapularis' 'Galendromus_occidentalis' 'Varroa_destructor' 'Varroa_jacobsoni' 'Dermanyssus_gallinae' 'Tropilaelaps_mercedesae' Speleorchestes 'Osperalycus_tenerphagus' 'Aceria_tosichella' 'Fragariocoptes_setiger' 'Brevipalpus_yothersi' 'Tetranychus_urticae' 'Leptotrombidium_deliense' 'Dinothrombium_tinctorium' 'Hypochthonius_rufulus' 'Platynothrus_peltifer' 'Alaskozetes_antarcticus' 'Hermannia_gibba' 'Achipteria_coleoptrata' 'Steganacarus_magnus' 'Tyrophagus_putrescentiae' 'Psoroptes_ovis' 'Sarcoptes_scabiei' 'Dermatophagoides_farinae' 'Dermatophagoides_pteronyssinus' 'Euroglyphus_maynei'

;

END;

BEGIN CHARACTERS;

TITLE Character_Matrix;

DIMENSIONS NCHAR=33393;

FORMAT DATATYPE = Protein GAP = - MISSING = ?;

MATRIX

'Limulus_polyphemus' PGSKVNALSSEVMQGVVLMSSKPGCFIAGADIGMLEACQTEVQKLKPIVAAIMGSCLGGGLEVALACQYR-IAVKDKKTTLGLPEVMLGLLPGAGGTQRLPKLISIPSALDMMLTGRSIRPDKAKKMGLVDQLVMPLGPGLYLEEVAASKLARGEMKPRKRPLIERVIREQ-LFKKVQAQVIKQTQGLYPAPLKILEVVKTGLESMGFGELATPHSRALIGLYHGQTLCKKNRFGAPAKKVGVVGAGLMGAGIAQVSVDKGYEVILKDGLARGEHQIKKKKIEKERFLSNLEPVLNYSDIVIEAVFEDIEIKHIVLKEVEQDHCIFASNTSALPISKIAEASKRPEKVIGMHYFSPVDKMQLLEVITTDKTSSDTAAMAVDVGLKQGKVVITVKDGPGFYTTRILAPMLAEAMRILQEGTNVKELDSLKKFGFPVGAATLVDEVGIDVAAHIAEGVFGERKDLVAGCLGRKSGKGCFVYRDINPILKKYTTEELQMRLICRFVNESVLCLQEGILANPLEGDIGAVFGLGFPPFLGGPFRYIDSYGADKIVGMEKFTEFKPCELLIDHA-RDPTKNFHMQQPIILLKEGTENIQGKSQLISNINACQAVAEAVRTTLGPRGMDKLIVDNNGKATISNDGATIMKELDIVHPAAKTLVDIAKSQDAEVGDGTTTVVLLAAEFLKQCKSYIEEGVHPQVIIKSYRKAVQLAVDKINEISVKVKKEKHRAILEKCAMTTLSSKLVANQKTFFSKMVVDAVMQLDELLPLNMIGVKKVQGGALEESKLISGVAFKKTFSYAGFEMQPKKYVNPKIALLNVELELKAEKENAEVRVESVQEYQNIVDAEWNILYDKLKKIHDSGAKVVLSKLPIGDVATQYFADRDMFCAGRVQEEDLKRTMKACEFCYLTMVSPVRASSGCICRDIEYKVLFPYRYNLFTGCSNTKTVTIILRGGAEQFIEETERSLHDAIMIVRRAMKNDAVVAGGGAIEMELSKYLRDYSRTIPGKEQLLIGAMAKAFEIIPRQLCDNAGFDATNILNKLRQKHATGELWCGVDINNEDIANNFDACVWEPAIVKINALVAATEAACLILSVDETVKNPKSETDPSKGRPFMNEVNPAAANKQHQLAVTRDFISQPRLTYKTVSGVNGPLVILDDVKFPKFAEIVHLTLADGNVRTGQVLEVSGSKAVVQVFEGTSGIDAKHTVCEFTGDILRTPVSEDMLGRVFNGSGKPIDKGPHVLAEDFLDIQGQPINPWSRIYPEEMIQTGISAIDVMNSIARGQKIPIFSAAGLPHNEIAAQICRQGGLVKVPGKSLDDDNFAIVFAAMGVNMETARFFKQDFEENGSMENVCLFLNLANDPTIERIITPRLALTTAEFLAYQCEKHVLVILTDMSSYAEALREVSAAREEVPGRRGFPGYMYTDLATIYERAGRVEGRNGSITQIPILTMPNDDITHPIPDLTGYITEGQIYVDRQLHNRQVYPPINVLPSLSRLMKSAIGEGMTRRDHADVSNQLYACYAIGKDVQAMKAVVGEEALSSEDLLYLEFLGKFEKNFISQGNYENRSVFDSLDIGWQLLRIFPKEMLKRIPQKTLTEFYPRDSRHKGLFGEPDGFYLLKEEAMLETDKLVREATSRTRKMVQIFDELSDTLCKVADMAEFVRIGHPQNRFAKAAENASMAISGLVEKLNTNRELYNALKKVTQEG-DIVLTNVDEHVAELFLFDFEQSGIHVDDLMRERAVALNEYILHIGSHFVNGTNQPKSVLKSQLPENIILVTGLFADSENDLVREAAYKIYLFPDSHQAKLLDELLISRNELANLCGFPTYAHRAVRGSLAGDPQTVEFLDILSDGLRERAQNDYNDMLQLKPWDIPYYTPYFSLGICMEGLNNLFQSLFGTTFDVEGELWHKDVIKLSVHEEGSILGYIYCDFFERPGKPHQDCHFTIQGGRLENGTYQLPVVVLMLNLPPSLLTPGMVDNLFHEMGHAMHSMLARTPYQHVTGTRCATDLAEVPSILMEHFSSDPRVVLQFAKHYQTGAPIPHSLIEKWNASKQLYSASETQLQVFYAALDQVFHGEHPLSTTEILADVQNQYYGIPHVPNTAWQLRFGHLVGYGAKYYAYLMSRAVASCIWQVYFKDDPFSSSAGEYRRGVLAHGGSRPPRELIQDFLLAESLLNDIMPSDSKKKREAKKKEAAKQRAQKKTANGLASENELVKKLEHDMDLNAKARAVTGVQGLNPHSRDIKIENFSITFHGAEILADTKLELNCGQRYGLIGLNGSGKSSLLASIGRREIPIQDHIDIYHLTREIEPSNNTALQAVLDVDQERIRLEKLAEELAHYPDDDSQEQLMDIYERLDDIGADKAVSKAAYILHGLGFNKRMQEQKCKDFSGGWRMRIALAKALYIRPHLLLLDEPTNHLDLDACVWLEEELKSYKRILVIISHSQDFLNGVCTNIIHLHKKRLNYYGGNYDAFVRTRLELLENQMKRYNWEQSQISHMKDYIARFGHGSAKLARQAQSKEKTLSKMVSAGLTEKVMLDKNVQFYFPSCGTIPPPVIMVQHVSFRYTENGPWIYKDLEFGIDLDTRVALVGPNGAGKSTLLKLLCGELIPSDGLIRKHSHLRIARYHQHLHEQLDLDVSALEYMMRCFPDVKEKEDMRKIIGRYGLTGRQQTCPIRQLSDGQRCRVVFAWLAWQTPHLLLLDEPTNHLDMETIDALADAINDFDGGMVLVSHDFRLISQVAEEIWVCANQTISKWSGDILSYKEHLRKRIISEIQKMVLADLGRKITSALRSLSNATIINKEVLDSMLKEICAALLEADVNIKLVKQLRENVRSVIDFEDMAGGLNKRRMIQSAVFKELVKLVDPGVKAYQPTKAKSNIIMFVGLQGSGKTTTCTKLAYYYQKKGWKTSLVCADTFRAGAFDQLKQNATKARIPFYGSYTEVDPVVIAQDGVEKFKNEGFEIIIVDTSGRHKQEDSLFEEMLQVSIAVSPDNIIFVMDASIGQACEAQARAFKEKVDVGAVIVTKLDGHAKGGGALSAVAATHSPIIFIGTGEHIDDFEPFKVKPFVSKLLGMGDIEGLIDKVNELKLDDNEELIEKLKQGEFTLRDMYEQFQNIMKMGPFSQIMGMIPGFSSDFMTKGNEQESMSRLKKLMTIMDSMNDQELDHREGAKLFSRQSGRVTRVARGAGVTSREVQELLSQYTKFAAMVKKMGGIKGLFKGGDMGKNVNPAQMAKLNQHMAKMIDPKVLQQMGGMSGLQNMMRQLQGALLRDDPAFKKLQDYYDRNGSKINMLQLFNEDPDRFKKFSLNVKTAVGEILFDFSKNLINDEVLQLLLELAKSRKVEAARDAMFSGEIINFTENRAVLHIALRNMAGTSIIIDGQDVMPGVMRVLNHMRDFTDFVRNGEWKGYTGKPITDVVNIGIGGSDLGPLMVTEALKAYCCGPNVHFVSNIDGTHLAETLKHLNPETSLFIIASKTFTTQETITNAQSAKTWFLQHAKDDTAVAKHFVALSTNEPKVKEFGIDEKNMFEFWDWVGGRYSLWSAIGLSIALHIGMDNFEQLLTGAHFMDQHFKNMPLERNVPVLLALLGVWYGNFYGAESHALLPYDQYLHRFAAYFQQGDMESNGKYVTRSGAIVNYTTGPIVWGEPGTNGQHAFYQLIHQGTRLIPCDFIAPVKTLNPISDGLHHEILLANFLAQTEALMRGKTKEEAETELTKAGLDGEQLQKILPHKVFEGNKPTNSIMIPKLTPFNLGVLIAMYEHKIFTQGIIWDINSFDQWGIHAIFLFIKFGQLGLAGPDPISGNFSVTGYCLNFIIYSV-------------------------------------MDKMIQAGNGDVTITNDGATILKQMQVLHPAAKMLVELSKAQDVEAGDGTTTVVIIAGSLLDAASKLLAKGIHPTTISESFQKAAIKSVEVLSDMAVPVDLSDRESLLKSASTSLNSKVVSQHSSQLAPLAVNAVMKVIDPTNVDLRDIKIIKKLGGTVDDTELIEGLVFAQKTSGAGGPNKIEKAKIGLIQFCISPPKTDMDNQVIVSDYTQMDRVLREERAYILNIVKQIKKAGCNVLLIQKSILRDALSDLALHFLAKMKILVVKDIEREDIEFVCKSLVCKPIASLDHFVPEALGSADLVEEIHGSKFVKITGVANP---KTVSIFMRGSNKLVLEEAERSLHDALCVIRCLVKKRALIAGGGAPEIEISLRLSEHARSLTGMEAYCYRAFAEALEVVPYTLAENAGLSPISTVTELRNRHAQGEKTAGINVRKGAVTNILEENVIQPLLVSTSAVTLAAETVRSILKIDDIWL-----KKSVIYFWDPDVGNFHYGPGHPMKPHRLSVTHSLVLHYGLYKKVQVYRPYRASAHDMCRFHSDEYIDFLQRVTPQNIQSFTKSLSHFNVGDDCPVFDGLYDFCSMYTGASLEGAVKLNNMCCDIAINWSGGLHHAKKFEASGFCYVNDIVIAILELLKYHPRVLYVDIDIHHGDGVQEAFYLTDRVMTVSFHKYGNYFFPGTGDMYEIGAESGRYYSVNVPLKEGIDDQSYFQVFKPVIQHVVEFYQPTCIVLQCGSDSLAGDRLGCFNLSIRGHGECVKFVKELGLPLFVLGGGGYTVRNVARCWTYETSLLVEETISNEIPYNEYFEYFAPDFLLHPE-------ENANSRQYLEAIIKATTENLKCLIHAPSVQMQDVPPDTLNFDNNESEADAKAEPANEFFDKDQDKDNE??????????????????????????????????????????????????????????????????????????????????????????????????????????????????????????????????????????????????????????????????????????????????????????????????????????????????????????????????????????????????????????????????????????????????????????????????????????????????????????????????????????????????????????????????????????????????????????????????????????????????????????????????????????????????????????-MALSLNPVRILKGDAEEEKAEHARMSSFIGAIAIGDLVKSTLGPKGMDKILLCEGRQEGKVECTNDGATILRSVGVDNPAAKILIDISKVQDDEVGDGTTSVAVFASELLKEAEQLINMKLHPQTIIAGWRKAAIEARDALTSFARDNSGNQEKFYEDLINISRTTLSSKILSQSKDFFAKLAVDAVVRLKGSGNLQAIQVLKKLGGSLTDSFLDEGFLLDKKVGVNQPKRIEKARILIANTPMDTDKIKVFGSRVRVESVAKVAELELAEKEKMKDKVEMILKHNINVFINRQLIYNYPEQLFADVGVMAIEHADFDGIERLALVTGGEIVSTFGCPEKVKLGFCNLIEEVMIGEDKLLKFSGVPLGEACTIVLRGSTQQILDEAERSLHDALCVLSQTVKETRIVFGGGSAEMLMATAVGRLAERTPGKEAMAMESFAKALRQMATIIADNAGYDSAQLISELRAAHAEGKNTYGIDI------------------------------------------------------MAAEIKPGGKPQLLNKLEGHQDTVNMAVIIPGEDGVISISDDKTVRIWLKRDTGQYWPSICHYMPSAATCMDYNSETKRLFVGMENGSISEFQVAEDYNRMAHQRNYLAHQAHVTGVVFSLITEWVLSVGRDKYFQWHCSESGRRFGGFHCNAWCTALQFDMQSKHAFIGDYSGQITMLKIESSGYKPVTTLKGHSGSICCLSWDAERQLLFSGSFDQSIVVWDIGGKQGTAYELQGHHNKVTALCYAGMSKLISGSEDSILVFWHMEAFPLDTPEWAESDCCQRCTRPFFWNVKAMIDQKTIGIRQHHCRRCGKAVCDACSTNQSSIPTMGYEFDVRVCDECHSVITDDDRIPMATFHDARHCIVDMDLDETRFHLLTVGSDRIMKLWDVSSLLIVTCLIQVTKVAPKKKDVINKWTRNLKEGVNTEEDYSSGQYGKLPMNQSQKKIVRHLVEVKVLDLNFADQKVWVRGRLHTSRAKGKQCFFVLRQQHFTVQCLVSVSETISKAMVKFVALITKESILDVEGHVRTVFQKIESCTQKDVELHVEQLFVVSASEPRLPLQIEDASRPENE-----------------------------EAIYCLQAGVCELFREALKKMGFIEIHTPKIISAASEGGANVFEVSYFKGSAYLAQSPQLYKQMAIAADFDKVFTIGAVFRAEDSNTHRHLCEFVGLDLEMAFHYHYHEVITVIGMMFVEIFKGLRDRFSAEIAAVHRQYPAEPFKFLEPSLRLDYPEAVAMLRETGVDMGDEEDLSTPNEKLLGRLVKAKYDTDFFILDKFPLAVRPFYTMPDPHNSKYSNSYDMYMRGEEITSGAQRIHDPEFLAERAKNHGINIEHIKAYIDSFRYGAPPHAGCGIGLERVTMLYLGLDNIRKASMFPRDPRRLTP------------------------------------------------------------------------------------------------------------------------------------------------------------------------------------------------------------NMLRLVQEGLYSIQIKKYIGERFRWYPDERVTDFLLKQCICVHLDSNVHKFNLLVYMTRKLFALAKGECAIENPDNPMNQEVLLGGHLYLMVLKEKLESWLNTIKMILNRKMLQYIDISRPMEYLLATGNLVSRSGIGLMQFSGLTIVADKLNFFRYLSHFHSIHRGSFFAEMRTTAVRKLLPEAWGFLCPVHTPDGAPCGLLNHISAMCEIVVLLDGKFVGWVLRYMKVMQKVPSTLEIAFVEATTKASQYPGIYLFSTPARMLRPVVNTTNTIELIGTFEQVYLNICVVPEEATTHQELRETSMLSVLANMIPFSDFNQSPRNMYQCQMGKQTMGTPCYTYQYRSDNKMYCITPQSPLVRPVMYDFYHIDDYPLGTNAIVAVISYTGYDMEDAMILNKASLERGFKNGVYKSEFINLRIDMDGLPFIGTYGTPMCSYIETRVEKYKSTESAYVLYVKLCANLQKICITLWIRNPIIGDKFASRHGQKGVCSQQWPAENMPFTESGMTPDIVFNPHGFPSRMTIGMMIESMAGKAAAVHGLAFDATPFKFNEKKPAAEYFGELLQSAGYNYYGTERMYSGVDGRELEADIFFGVVYYQRLRHMVADKFQVRTTGPIDILTHQPVKGRKRAGGIRFGEMERDSLLAHGTSFLLQDRLFNCSDKSLCTRCGSLLSITVPYVFRYLVVE-LAAMNIKI-----------------------------------------------------------------------------------------------------------------ISHHLEPLFVTCGEKVDLWEERSEPLRSFW-GVDSLHVKFNPVESTASDRSIILYDIREAHPLRKVVLELRSNTICWNPMEAFIFTAANEDHNLYTFDMRKLKVPLQIHLDHVSAVIDVDYSPTGKEVVAGSYDKTVRIFRSRIVKKEGRSHVTTTDVTVTDDRTIPDGADEP-------------------------------------------------------------MDVIALHQIEDGISYYRLYVEKFSKDHLSHQMIGNVYQLLFKLKLTLNDLIGIVTDDYSFCYARLQRGNILLKQGRLEEAHIDYEWVLRLDPLNGDADLEPVKRDAIDVLTRLWDGKLREMRAGCYEALNDLVNAIGDLRSATKMRSDNTEGYLKLSQLHYKLGEVDESLTVIRECLKLDPDHKQCYKKVKKLARAMQDSCVEKIVQHIKGRCHCLNKGGEAISICSEALKLNAALCDRADAYLLNEDYDEDFQHAVNIDERAKEGLEKTQKLVKQAKKRDYYKILAGKREIMKAYRKLAMKWHPDNF-QGDE--KKTAEKKFIDIAAAKEVLTDPEKRQKFDNGEDPLDPDSQFTFKFHFMLVLFESPAGYAIFKLLDEKKLQETENLYKDFESSETASKVVKLKHFQKFQDMTEALSAATAAVEGKMSKGLKKLLKKLVAKEAQESLAVADAKLGNVIKEKLNVSCVYNSAIQELIRCIRMQIDNLIVGLPQKEITAMALGLAHSLSRYKLKFSPDKIDTMIIQAVSLLDDLDKELNNYIMRSKEWYGWHFPELSKIITDNNQYVHTVLAIGLRTNAAECDMSDFLPEELEHKVKEIAEVSMGTEVSEEDIMNIKHLCNEVLEMQNYRAQLYEYLKNRMMAIAPNLTVLVGELVGARLIAHAGSLLNLAKHPASTVQILGAEKAFFRAIKTKHDTPKYGLIYHAQLIGQCNPKLKGKMSRMLAAKSSLATRVDALGEEASQELGIEHRAKLETRLKQLEEGSLRKISCYLWPKDKPGIKGRVVVALGLLVGAKLLSVEVPFIFKYAVDFLNSHSGLMLGYGAARAGAALFNELRNAVFAKVAHNSIRRVARRVFLHLHDLDLSFHLSRHTGALSKVIDRGTRGINFVLSALVFNVVPTIFEVTLVSSILYYKFGKFALVTLGCIGTFAVTSWRTRFRLEMNKAETQAGAKAVDSLINYETVKYFNNEEYELTKYERASLKTTTSLALLNFGQNAIFSGAIMILASQMTIGDLVMVNGLLFQLSMPLNFLGSVYREVRQSLIDMQTMFELMAIETKLLQVTPTVTFEDVCFQTILNNLSFTVPTGKKVALVGGSGSGKSTIIRLLYRFFNPDKGRIMIAGKDIKDVSLRKAIAIVPQDTVLFHNTILHNLHYGDLTDEVYQAARLAELHDTIMRWYETQVGERGLMLSGGEKQRVAIARAALKDSPILVFDEATSNLDAITEYKIMNALRASKGRTSIFIAHRLSTVVDADDIIILEGGQVVERGHHSLLSSLYTHLWFKQHELNVPETRVTTLTNGLRVATEDSGIPTCTVGLWIDTGSRYETDKNNGVAHFLEHMSFKGTSKRSQTDLELEIENMGAHLNAYTSREQTVYYAKSLSKDLPRAVEILADIIQNSKFGEQEIERERGVILREMQEVETNLQEVVFDHLHAVAYQGTPLGRTILGPTENIKSISRQDLVEYITNHYKGPRIVFAGAGGVGHEELVKLVDQHFGSLKTTYEGK--TPCRFSGSEIRVRDDSMPFAHVAVAVEGCGWTNPDNIPLMVANTLIGSWDRSHGGGSNVASKLGQAATLGNLCHSFQSFNTCYKDTGLWGIYFVSEGLSLEDMLFNVQSEWMRLCTSVTEGEVSRAKNLLKTNMLLQLDGSTPICEDIGRQMLCYGRRIPLPELEARIEGIDIEIKNSI-------KCYNI-----VESKFNFITTDKQLLKHSVHTLVFRSLKRTHDMFLSDQANPPPPDETSEKIKLMVKAADEYKPVMHLIRNNERRSVSQALVLAGTQAIS-RKAPTMPKPQWHPPWKLYRVISGHLGWVRCIAVEPGNEWFCTGSNDRIIKIWDLASGKLKLSLTGHISGVRGLAVSPRQPYLFSCGEDKQVKCWDLEYNKVIRHYHGHLSGVYALALHPTIDVLITGGRDSVARVWDMRTKANIHTLAGHTNTVASVQSQATEPQVMTGSHDCTIRLWDLVAGKSKVTLTHHKKSVRALLIHPKLNMFASGAPDNIKQWKCPDGKFIQNLQGHNAIVNCLAMNVDNVLVSGGDNGTLFFWDWRTGYNFQRLQTPVQPGSIDSEAGIFAAIFDQSGTRLITAEADKTIKIFKEDDMATEDSHPINWKPEIAVLNKNSYVCGVSSSVRLDIIQDGLHLMYSIVYQGAFPVCICQFSKLRTVLQ?STSYVNDKRVEETHTKKEKLWKKSKILKFDEAVLLQHILQDSGARKLVVDFFNQD--LVYKFIKNSMYHTTLYSNKTSQNHRIEIELSKKRPAYIKAKERTAHMQKKLESAKKSLKAAKKVHEAHEGIKELENELKEVEEEFEEQDLTLEESQVQEYNSLKEEAGKLSSRYLQELDSVNREQKSDQDRHDNELRKKAEMEAKVKELEENVRRVEKLNELSDLMKQEEGFDAKKRINDELESIIELGDAKVDKHEDSRRKKKAEIVDHFKRLFPGVYDRLVNMCQPIHKRYNVAITKVLGRNMEAIVVDTERTARSCIKYLKEQMLEAETFLPLDYIDAKPLKERLRNIQSPRNVKLLYDVLQYDPPAIKRAVLYATNNALVSETAEDASKVAYELGDGKRYDAVALDGTYYQKNGFISGGSTDLAKRARRWDEKALHNLKYRKEKLTEELKEMVKKTRKESDLNTIQSQIRGLETRLKYSIIQSVEWRMKEREVQIAEVKEAMNTVEDRVFADFCHTIGVENIRQYEERELRATQERDRRLEFENQKNRIINRLEYER--SKDTNEVTDDEKELEKLKEAESKQMQLIDEEMQNLDRLKTSKITKKTEVDGMEDAMTEVRKRLTAMQKEITSVQKVVTGLETRLEQKKADRHSHLQACKLEDIIIPMKRGSMQRIYEKEKIQIDYIDLHLQKEISLQRIQAPNMRAMEKLDGVRERLRLTDSEFESARKKAKQVFEKVKRERSCFDHVSNKIDDIYKALTNNQSAQAFLGPENPEEPYLEGINYNCVAPGKRFQPMSNLSGGEKTVAALALLFAIHSYQPAPFFVLDEIDAALDNTNIGKVARFIREQTETSFQCVVISLKEEFYGHADALVGIVPDPGECTISRVLTLDLTVPEGVLVYLRKAIGEGNVYEIQDLYENSYVKLTERYFLILYKE--LYYHHLYADQRFESYYNYCDLFNYILSAGPVPLELPNQWLWEIIDEFIYQF--QSFSHYIWNVHSVLNVLHSLVDKSNINRQLEVYTSGGDPDSVAGEFGRHPLYKMLGYFSLVGLLRLHSLLGDYYQAIKVLENIELNKKSLSRVPACQITTYYYVGFAYMMMRRYADAIRTFADILLYVQRTRKTAQAEEISKQTDRMYHLLAICLVLHPQLIDESVLSQLKEKHMLRLQKGDLQEFENSPKFLSPVQLKVFMDEVQQQLTIRSYLKLYTTMPISKLAAFLLLCFKRKLLNV-FFTEVQSRSFLNVFFKPDMIHIADTKVARRYGDFFIRQIHRFDELYRMGPPDPILGVTEAFKRDTNPKKINLGVGAYRDDQGKPFVLPSVRKAEELMMARHMDKEYAAIHGLQEFCQASASLAFSENSSVIKEGLNATIQGISGTGSLTMGAFFLRDFFKGNKEVYMPAPTWGNHIPLFKRAGFAVKQYKYYDPKTCGLDFKGLLEDISKIPENSVILLHACAHNPTGVDPKPEQWKEIQKPCKGKKLFPFFDMAYQGFATGDIDRDAMAVRMFVDNGHRIGLAQSFAKNMGLYGERVGAFTMVCNSKDEAQRVLSQLKIIIRPTYSNPPIHGARIAQTVLGEPELREQWLKDVKGMADRIISMRTRLRDGLKKEGSTRNWQHITDQIGMFCFTGMNPKEVEKLTAEFSVYLTKDGRISVAGISSNNVDYLAHAMHQATKILNSRTKHLLHCFLCFAVIITFEIFTFEFDNIDPIQKYGYIGSSILYLLRILTLLALPQCVFNFLGLVVYNAFPDKVQLKGSPLLAPFICVRTVTRGDYPELVKTNVTRNMNICFDIGMENFMIEVVSDKPVNLPKHPRIRELVVPSTYRSKSGALYKARALQYCLEDDVNILSDTDWIVHLDEETLLTENSLRGIMNFVFDGTHKFGQGLITYANENVVNWVTTLADCFRVADDMGKLRFQLQTYHRPLFGWKGSYVVTQAGAERKVSFDNGLDGSVAEDCFFAMMAYKEGYTFDFIQGEMWEKSPFSFWDFLQQRKRWMQGIFLVVHSPAIPFRNKIFLSLSLYSWITVPLSTSNLVLAGLYPIPCWQVLNVICAFVGAMNIYMYIFGVIKSFSLYRLGFFKFVFFVVGALCTIPFNIIIENIAVIWGFFGNKYRFYPWPGFQFTGKLRPFPKTPKREVPDLIQRPDYADHKKGWPLSEQAVKGSSHIKVLDDDEIEGMKVVCKLAREVLDVAADAAGVGVTTDEIDRLVHEASIERECYPSPLNYYGFPKSCCTSVNEVICHGIPDARPLEDGDLLNVDVTVYHRGFHGDLNETFLIGNVDEEGRRLVQVTHESLMKAIEIVKPGVKYREIGNVIQKHVQAHGFSVVRSFCGHGIHRLFHTAPSVPHYSKNKAVGIMKAGHCFTIEPMISEGTWRDLLWPDDWTAVTQDGKRSAQFEQTLLVTDSGCEILTQRREKGGQPHFMDKKNMLKVLKERSQKRRLLLAQQLGAGSVDNLSIVLGNQEDKYSRSSSKSNL---KSYSSSSKTGRLSTSYLLYNIYQEHFVHLIFYSSTFANFIYFEKAFEQSAYIQYLHRVKDHLIKGLREIW?YLKCDMETFELRELNCKFDVILVEPPLEEYQRSQGVSNSKFWTWDEIIKLEIEEVAAPRSFIFLWCGSSDGLDLGRQCLRRWGFRRCEDICWIKTNIKNSHSKNLEPRAIFQRTKEHCLMGIKGTVRRSTDGDFIHANVDIDLIISEETEYGSLDKPEEIFHIIEHFCLGRRRLHIFGRDTTIRPGWLTIGPELTNSNFNSETYNAYFNGPNEFLTGCTDRIEALRPKSPPPK?????????????????????????????????????????????????????????????????????????????????????????????????????????????????????????????????????????????????????????????????????????????????????????????????????????????????????????????????????????????????????????????????????????????????????????????????????????????????????????????????????????????????????????????????????????????????????????????????????????????????MRTFGEKPTAFQLEEGGDYYYIGSEVGNYLRMFRGSLYKKYPSLWRRLVTVDERKKIASLGLGPHSLATNITLLRAVEVDEIFEGNDDRYKAVSISTEPPLPRETKSKRTTWMPALPNSSHHLDAVPCSTPINRNRSGHKKVRTFPHVYDDLDPSVIHENSSQPEVLVPIRLDMEIDGHKLRDTFTWNKNDSLITPEQFAEVLCDDLDLPPLSFVPAIAQSIRQQIDAFPTDNLLDEQTDQRVILKLNIHVGNISLVDQFEWDMSEKENSPEQFALKLCSELGLGGEFVTAIAYSIRGQLSWHQRTYAFSEAPLPTVDVPFRSQTEADQWCPFLETLTDAEMEKKIRDQDRNTRRMRRLANTAWYGANASQLEKEIGFPSNEHYFGLVNFGNTCYCNSVLQALYFCKPFREKVLDYKAKNKRTKETLLTCLADLFYNIATQKKKTGTIAPKKFIARLRKENDLFDNYMQQDAHEFLNYLLNTIADILQA---WVHDIFQGTLTNETRCLNCETVSSKDEDFLDLSVDVDQNTSITHCLRGFSNTETLCSEHKYYCENCCSKQEAQKRMRVKKLPMILALHLKRFKYMEQQSRHTKLSYRVVFPLELRLFNTSDDAYNPNRMYDLVAVVIHCGSGPNRGHYISIVKSHGLWLLFDDDIVDKIDASAIEDFYGLTSDTQKTSESGYILFYQSREMPDIDKWIDIAKECKYLPENDLKKLCDIVCDLLLEESNIQPVSTPVTVCGDIHGQFYDLEELFRTGGQVPDTNYIFLGDFVDRGYYSLETFTRLLTLKAKWPDRITLLRGNHESRQITQVFYIQNECQTKYGNANAWRYCCKVFDLLTVAAIIDEQVLCVHGGLSPEIRTLDQVRTIERNQEIPHKGAFCDILFVESIDVDTWAVSPRGAGWLFGSKVTHEFMHLNNLKLICRAHQLVHEGYKYMFEEKLVTVWSAPNYCYRCGNIAAVLAFTDVENRTAKLFHAVPDSERVIPPRNTPYFLMKNAEGETVKDTDLLTIEDLKEHARHIEKSVSSKEPRFIFRVLRALVTTRKKLNSKVLRKIICGFYTHSVEQRDSLLTFVEPMDTEQAVAGKAAQLPLLPELDVYFHLLVLLYLIDLQRIDQAVKCSNLLMQKVHGNNRRSLDLLASKCYFYHSRCYELADHLKDIRSFLHSRLRTATLRNDYEGQAVLLNCLLRNYLHYNLYEQAAKLVSKSVFPDMASNNEWARFLYYLGCIKAIQLEYSEAHKNLLQAIRKAPQHTAVGFKQTVNKLAITVELLLGDIPDRSIFRQPTLRRTLAPYFQLTQAVRTGNLGRFNEVLENFGAKFQADHTYTLIIRLRHNVIKTGVRMINLSYSRISLADIAQKLELDSSEDAEFIVAKAIRDGVIEATIDHEKGCMQSKENIDIYCTREPQAAFHQRISFCLDIHNHSVKAMRFPPKSYNKDLESAEEFSLKNELLLLHPKKEKLEMYIVLTQLCVALSAYIIHTVWPTAIADVAATVQPSHLILLELLTVLPEEFQTKGIIRHQVSELLLEVLSQDLHQMGLKCYYESLFRLSL-SVYNEEALEALSNVVTHPEARYPNLILKLLNSVIQLEDLLNKSLEEKDMDSIYGLFISFGEHHTKLLLDTLIEKPEFRIMKLIELIKQASATPGHYPVDELCSEQTFGFWYTLQDDIVASEP-PQFEATFNPVFHSLVDYLLKVQYPPDDLYLDDEKESFRCYRQDIGD--SFMYCYNILREAMLANLLGHLELA-QWQHLEASLFAFQSVAESVAFEEQHYL-PKFFNFLPKVP---RIFSAVMDAVGAYAEQVLGDVVPLLLLGLQNAP-ASTMALKDITRDCQACLKPFSEQILKYSQELKSREQVRLMNTIGQVLSMM-PNFILSYLDPVLTPMLQQLEHALTVTQKIMPVFNTVATVV-AQDEQVAEALCDALKRAVLTL-LEPIVEDILQLILRLYLDVTKQ-DLREHTSLLEIFYQTSQLIKKLDLSALFQCSPEKPTVKSLAEFINCSREVPLLNVVESLVVQVLRIIGSPRHVVEYMSDILMALNKKYRWMSTYLHSTEQKENFVRLILKERSNKRKLKETV--NEFTLRVKVKWKEVFPDVEVNTPMVFKAQLFALTGVQPDRQKVMLKGVTLKDESWGATVLLMGSKEELPGPTEKPVFMEDMTESELATALDLPAGLTNLGNTCYMNATVQCLRTVPELRDLRNFQGSITAALRDLYESMDTATIPPIILLQVLHMAFPRFAEKSEHGQQDANECWTEVMRMLQNFIDQYFGGTFVSLKCVESDDESKENFLQLSCFIS-QDVKYLHSGLKSRL-QETITKYSPTLNRDAQYKKSVISRLPAYLTIQFVRFYYKEKGSINAKILKDVKFPLRLDAFDLCSKELQQKLMPMRARFKEQEDKFSFPDDGSNNSGYYDLQAVLTHRGRSSSSGHYVSWIKREWFKCDDDKVVTSEEILKLSGGGTVHMFYCMVH---VF??????????????????????????????????????????????????????????????????????????????????????????????????????????????????????????????????????????????????????????????????????????????????????????????????????????????????????????????????????????????????????????????????????????????????????????????????????????????????????????????????????????????????????????????????????????????????????????????SSRHAKIFCPAHPDAPLIEDYRAGDMICPECGLVIGDRVVDVGTEWRTFNNEKSTNDPTRVGAAENPLLNGSDLSTVVGRTGDASDESGVAKYQNRRTMSSSDRALTNAFREINNMADRINLTKTIVRTTTLLFKQVHDGKTLKGRSNDAIASSCLYIACRQEGVPRTFKEICAVSKVSKKEIGRCFKLILKALETSVDLITTGDFMSRFCSNLGLPNTVQRAATHIARKAVELDIVPGRSPISVAAAAIYMASQASQDKKSQKEIGDIAGVADVTIRQSYKLMYPKAAELFPEDFRPSDGRPWIEKYRPVNFSDIVGNSETVSRLEIFAKEGNVPNIILAGPPGVGKTTTILCLARTMLGPSYKDGILELNASNDRGIDVVRNKIKMFAQQKVTLPKGKQKIIILDEADSMTEGAQQALRRTMEIYSKTTRFALACNTSDKIIEPIQSRCAMVRFGKLSDAQILSKLLAVCEKEDVSFTEDGMEAVVFTAQGDMRQALNNLQSTYSGFGHVNSDNVFKVCDEPHPLLIKDMLQHCVSGNFEDAYKIMAHLWKLGYASEDIISNIFRVCKNESIAEYLKLEYIKEIGYTHMRIVQGVSSLLQMSGLLAKLCQKVAVPDYLKKEHSLMKPYQGSGMTVPNWDFVGSTMVTSNYIRLTADEQSRIGAIWNKVPWELQVHFKVYGQGKDLFGDGLALWYAKEPLQIG-------HTFIKVFYISLIHQSN-HSHGHPYISAMVNNGTLHYDHDRDGTHTELAGCEAKFRGVDHETHISIRYEKDTVTVSTDIEGKNAWKECFKVSGVKLPTGYYFGASAATGELSDNHDIISMKLYELEEDRSNIEPSAASPRDHIDDPMSGTKLLLIMLCAIFGVFIVVFQKQQETSRKRFY--RGCVKDLR--NCLLSCAKRNGKTGDTVYYEIQLFSVGVYPVLANSSDSAHLLEVSIAVILFNSYILQKAPDEEPGFMYYFLSTIADVAANRFINSSAIYFAPNMSFTPSYKGFFNKTMPLFAPRAFRADDFNDPYHLEGTSTLNTIDAIDLGAISSNYTHEQYKINDWYFAWLPDQTRRHDSKTTYTVQITGTNETFVWHGPPAASDNPGPVKWTKPYFDCGRSNKWIVGASVPIPDIYPRHTGWRHIEIPTYVAVAVLELDFDRVDINQCPIGEGN-PRPNYFAGTARCKNRTTECEPIHGYGFRRGGYQCRCKPGFRLPRVVRNPYLGEIIERATQEEYNEGFHCKNINYLMVMTQNVESLMGFSTRLDPTLKGDIAFGKENQLENEARTAIRLANFISAMQQIIDPEELFAEFRVPDRPLNEDQVIGEVLANVIANEKIVGCGVFFNRNQFFAPY--AYRTKRNTQNFFVEDTTKIMIRYNSSGIRYDHYPLQYKAADIGYWTSPYFDCKGFHNEWLVTYAAPFFGWDKIKSRLEFKGAVTVMKLEELDINQCNAFKDTHKCDRKSSRCVPILGRGFMGGYKCECKQGYEYPFSDPVTYFDGQIVEAPNCLTSFRKFKLTNRAKYGGRFMVTMLPGDGIGPELMGHVKEVFRYAGVPVDFEEVHLESSQEDMKHVHKAITSIQRNGVALKGNIETRTNCPNFKSRNVELRLQLGLFANVLHCQSQPGVRTRHRDIDIVLIRQNTEGEYSCLEHENVKGVVESLKIITREKSEQIARYAFDYAQQHNRKKVTAVHKANIMKLTDGLFLQCCKEVAAEYSDIQFDNMIIDNCSMQLVANPHQFDVLLLPNLYGNILNNIACGLVGGPGLTSGRNYGNEYALFETGTRNTGKSIAGKNIANPIAMLNAGVDLLKHLGLTTHSKVISAAVDKTLNVDQIHTPDLGGQATTTDVMQNIIKEVVANTMKLQETPNEYKLENPPTDCVSSVKFGPNSNQFLLASSWDCSVRLYDVISNTMRLKYNHSGPVLDCCFQDAVHAWSGGLDCQVKVFDFNSSTESLAGNHDAPVRCVEFCPEVNMIITGSWDQTVKLWDPRTPCGAGTFSQPDKVYTMAVCGQKLIVGTAGRRVLVWDLRNMGYVQQRRESSLKYQTRCIRCFPNKQGYVLSSIEGRVAVEYLDPSPDVQKKKYAFKCHRIKDSSGMEYIYPVNAISFHNVYNTFATGGSDGYVNIWDGFNKKRLCQFHRYPTSISSLSFSTDGTVLAISSSFLYEQEDVKEIPPDAIYIRNV--------------------MKQYKPTDATTNPSLILQASSLPQYQTLIEKALKYGRGFDEQLSIAMDKLFVLFGCEILKIIPGRVSTEVDARLSFDKEGSIVKAKKIIQLYEEEGIGKERILIKLASTWEGIQAAKVLEEEDNIHCNMTLLFNFAQAVCCADAGVTLISPFVGRILDWYVQNTDKKSYEPEEDPGVLSVRKIYNYYKKYDYKTVVMGASFRNIGEVKALSGCDLLTISPKLLGELAESTEEVHAHLTEQSAKKEDLEKVEIDEKTFRWELNEDQMANDKLSEGIRKFAADARKLENILRERLKMGSLALRPLVTVYTEKNERSGASVALPAVFKAPIRPDVVNFVHMNMAKNRRHPYAVSEQAGHQTSAESWGTGRAVARIPRVRGGGTHRSGQGAFGNMCRGGRMFAPTKTWRRWHRKININQRRYAICSAIAATGIPALVMSKGHKIEETPEVPLVVSDKIQEYNKTKQAVQFLRKLKVWNDIQKVYKSKRFRAGKGKMRNRRRIQRLGPVIIYCSDNGLTRAFRNIPGIETLNVEKLNLLKLAPGGHVGRFVIWTESAFRKLDSLYGTWRKPSEKKKNFNLPMPKMSNTDLSRLLKSEEIRHAIRPVNRAIERRKLKKNPLKNIRVMLRLNPYAAVTRRNTILNAEKRKKMKILAKKRGIEIKPTRHQQAKKPKAKKVRLVQGSLLKKVLEAIKDLINEATWDCSATGISLQAMDNSHVSLVSVNLRSDGFDKYRCDRNISMGMNLGSMSKILKCAANDDIITVKAQDEADSVTFVFEAPNQEKVSDYEMKLMNLDTEHLGIPDTEYSVVIKMPSVEFQRICRDLSQIGDSVIISCTKEGVQFSASGDLGTGNIKLSQTANVDKEEEAVVIEMQEAVSLTFALRYLNSFTKATPLAAQVQLSMSADVPLAVEYKIADMGYMKYYLAPKIETNNVKVAILGASGGIGQPMSLLLKNNPMITHLSLFDVAHTPGVAADLSHINTRARVTGHLGIEQLKESLDGAEVVVIPAGVPRKPGMTRDDLFNTNASIVRDLTDACAQTCPKAMICIIANPVNSTVPIASEVFKKRGVYDSNRIFGVTSLDVVRANTFIAEAKGLDPTQVNIPVIGGHSGVTIIPVISQATPAVSFKADELDAMTKRIQEAGTEVVKAKDGAGSATLSMAYAGARFTNSLLEAMKGKGVVECTFVKSSETEAAYFASPILLGPNGIGKNLGIGKLSPYETELVKTAMPELLSNIKKGEEFV--EFEWLLQEEVSAVLEQLILLECCHRLPV-PKTEKYFM-QVKVVVTLSGDNISHADINLRI-HKHSHRTIVQNDCQWKLQQIQDAGNHLMLALHLLKYNFRSAEEVTEMVNKLMSCLQRGRACLIIPKRKTIDELQKSRNMKSLQPPLPNDLAVSFYVQAHKLVFAVYHL-QKDQMKF-DAESSVPWLSEVLVLFTVSLQLCQQLKDKVDNIHYRGMVPAFIDIFKSLGADISDLQQIIGVCDVCFKDIESVL--NSIVS-LLVAFCEKLTKTPSVRICLR--VLQNLYEGLLFDVYYSLVRIAGDSISSVFTDVRKLKCWFSTEKAKKLLRLLHEVLSELASKVMIELLGTYTEDNASHARDDAHRCIVACLADPTMFLMDHLLALKPVRFLEGELIHDLLTIFVSEKLSSYLKFYNNNKDFL-NSLSLSHEQNMQKMRLLTFMQMGETKKEISFETIQGELQLKFEEIEGFVIDVLRTKLVRAKIDHVNKKVLVSSTMHRTFGKSQWQQLREVLNLSMVEPSMES????????????????????????????????????????????????????????????????????????????????????????????????????????????????????????????????????????????????????????????????????????????????????????????????????????????????????????????????????????????????????????????????????????????????????????????????????????????????????????????????LSHRSTTIMQGAKKDLRDLLNIPENYDILFLQGGGTGQFSAVPMNLCTADYLITGSWSAKATKEGEKYCAYNGIPDQSTWNLSPDAKYVYFCANETIHGVEFPIVTDMSSNILTKPVDVSKYGIIFAGAQKNIGIAGATVVIIRDDLVGHVPFCPSILDYKLNLQNNSVYNTPSTFSIYIMLVFKWIKKQGGAEAMEKSQVKSKLIYDVIDSSSGFYDRSRVNIPFRI-GGSEGSEYLEKKFLDEAHMVSLKGHRSVGGIRASLFNAITVDEVEVLYKDRPSWRDIEPIVQIAYSEKFRDVFDYFRAVLKDEKSERSFKLTEDAISLNPSNYTVWYFRRVLLQALKKDLHKELIYVQSIIEDNPKNYQVWHHRQVIVDWLEKQLTEAVLDPKNYHAWQHRQWVLEEFGIWEGEIEFVEQLLEDDIRNNSAWNHRWFCISQSKGFVL-YNIVYVLDCIKSNESSWNYLHGILSPYLLAFMVDRAIEIDKIRAEYWKFMARNLALIVDRFEELLVFAPYMQNFLNEQKIKHKIFIINQVDQHRFNRASLINVGHLQSREECDYLAMHDIDLLPLNPALNYSYPEGGPFHIASPDLHPRYHYRTFVGGILLLTREDFELVNGLSNKYWGWGLEDDELYARMKKAKLNISRPKDINTGQKNTFKHIHDTKKRKRDTARLYNQREATRWRDHQTGLNTVQYRVVSKHKLMIDEASMTVINVELICNYTVTPWCDHKSVTPGDIITSDGFMRGHG-LVASVAGIVERVNKLVSVRPLKTRYNGEIGDVVVGRITEVQ--QRRWKVETNSRLDSVLLLSSVNLPGGELRRKTAEDELAMRKYLAEGDPISAEVQSVFSDGSLSLHTRSLKYGKLSQGTLVLVSPSLIKRRKHFHNLP-CGARIILGNNGYIWISVNEIWRETVARLRNCILALARHRLLLYDTTILYSYEAS-ISQLVRQKAQKVVGSWLMVCSGMVFGTVVLGGVTRLTKSGLSMIDWHPFKEFPPKTKQWEEEFHKYQQYPEYKLMNHEMTLQEFKKIWYMEYVHRMSGRSIGAIFFLPAAYFWYKGYFSKAMKPRVVAFAGLLAFQGLLGWYMVKSGLEEKPRVSHYRLAAHLGTAFVFYSLLLWSGLSHLLPTAQVRKFRMTKGVIFLAALSGALVAGLEAGLVYNSFPKFADRWIPSDILAYPKLRNVTENPTTVQFNHRILGETVVLGLWIYSRKVPLPPRARMAMNYVLVVALQVSLGIATLLFYVPKVLAASHQAGALTLLSTAIWLTHEMKLLRR-LAKWGEGDPRWIVEERPDATNVNNWHWTEKNASFWSQNLLKELLNDLTI-EGEVGRCITEITKVSGEAVANNRKGKLIFFYEWEITLHKDS-EIEGTAEIPNLSEENDPCEVDVNISV--QQLKDMMRTVGIQNIREKLAEYIKRLKDEFSQGMILPTKESNVLKVTPTKQRLDLTELNMESFKCKAEEFFRALTMVQAFTQGPCGGKFSLFDGNVHGIFVELKKIVQKWRFKTWPSGHYSDVLEITEKEDSTLTQKGVPRNELEQTREGWKNYYWQSMKRVFGFGAML??????????????????????????????????????????????????????????????????????????????????????????????????????????????????????????????????????????????????????????????????????????????????????????????????????????????????????????????????????????????????????????????????????????????????????????????????????????????????????????????????????????????????????????????????????????????????????????????????????????????????????????????????????????????????????????????????????????????????????????????????????????????????????????????????????????????????--------MCVLVGDGGTGKTTFVKRHLTGEFEKKYVATLGVEVHPLVFHTNRGPIRFNVWDTAGQEKFGGLRDGYYIQGQCAIIMFDVTSRVTYKNVPNWHRDLVRVCENIPIVLCGNKVDIKDRKVKAKSIVFHRKKNLQYYDISAKSNYNFERPFLWLARKLIGDPNLEFVAMPALAPPEVSMDPEWQAKLEKDMKEAQNTSLPDDDDDDLVAEFLTPILKESKFKETGVVTPEEFVICGDHLVHHCPTWQWASG-DKQYLPRDKQFLVTRNVPCYKRCKQMEEKLIETE-GGWVDTHSDEDDDEEAEDMDAFVDNEDKATEIVSTRTYDLNITYDKYYQTPRLWLYGYDENHKPLSIEEMYEDISQDHAKKTVTMETHPHLPGPPMASVHPCRHAEVMKKIIETVTEGGGLGVHMYLIVFLKFVQAVIPTIEYDYTQNFTM????????????????????????????????????????????????????????????????????????????????????????????????????????????????????????????????????????????????????????????????????????????????????????????????????????????????????????????????????????????????????????????????????????????????????????????????????????????????????????????????????????????????????????????????????????????????????????????????????????????????????????????????????????????????????????????????????????????????????????????????????????????????????????????????????????????????????????????????????????????????????????????????????????????????????????????????????????????????????????????????????????????????????????????????????????????????????????????------MFKNTFQSGFLSILYSIGSKPLQIWDKKVRNGHIKRITDNDIQSLVLEIVGTNVSTTYITCPADPKKTLGIKLPFLVMIIKNLKKYFTFEVQVLDDKNVRRRFRASNYQSTTRVKPFICTMPMRLDEGWNQIQFNLADFTRRAYGTNYIETLRVQIHANCRIRRVYFSDRLYSEDELPAEFKLYLPVQQIAI--CYQHQDLTKRDVLNAIHHYKNLIDRFVFNDGTTKDLMCLTGTIPVPFKYNIPVKIWLLDTHPVNSPMCYVTPTSDMKIKVSRNVDQNGRIYLPYLHEWNPNS-SDLIGLIQVMIIVFGETPPVYSK-PKPYP--NTGTISEEHIRASLLSAVEDKLKKRLREQTQAEIQVLKKTQDDLNNGKIKLDDIISKLEQEVELESEKNNEMKEVEIDDAVVTTAPLYKQLLNAFAEENATEDAIYFLGEALRKNVIDLDVFLKHVRELSRKQFMLRALMQRCRQKAGLPSNRKFFVGGNWKMNGSRSKIDDIVNFMVTGPVNTEVVVGCPSIYLDYSRKALPPTIGVAAQNCYKVASGAFTGEISPAMIKDVGVEWVILGHSERRNVFGENDQLVAEKVGHALAEGLKVIACVGELLEERESGKTEEVVFRQTKAIADKVSDWSKVVIAYEPVWAIGTGKTASPQQAQEVHAQLRKWLTDNVSADVASKTRLIYGGSVTAGNCKELAKESDVDGFLVGGASLKPDFVEIINAKAMAPEMERLRLFSHDKIRNFRTNNFLDRHPVCNGFALGKLGDTNTTLYITKMVTITNFVLGCCLILILNLLTEVETLLNNRVMTYELYAEKVAARGLCAIAQCESLRYKLIGGLAVRRACYGVLRFIMESEARGCEVVVSGKLRGQRAKSMKFVDGLMIHSGEPTNDYVETAVRHVLLRQGVLGIKVKIMLPWDRNGKIGPKRPLPDNVSIVNPKEETVPQQPYSEQK--AVSKIDTLKQWSISTYKCTRQIISEKLGKGSRTVDSELECQIEQLRETQRKYANILRLARALASHFHVVQTQHALAESFSDLAHK---ELQEEFLYNSETQRNLGKNGETLLGALNFFVSCLNTLCNKTIEDTLLTVKLYENARLEFDAYRSEARKNYKEKYEKLRSDVAVKMKFLEENKVKVMHKQLLLFHNAVSAY-FSGNQSMLESTLKQFNISWLEQ-MAELLLDSDIRLWVFLPIVILTFLIGIVRHYVSILISSTKKIELQQVQDSQALIRSRMLRENGKYIPKQAFLMRKHFFNNEESGYFK-TVKRAPAMQNPMTDPSMMTDMLKGNLTNVLPMILVGGWINWTFSGFVTTKVPFPLTLRFKPMLQRGIELSSLDASWVSSASWYFLNVFGLRSIYTLVLGENNAADQTRAMQDQMSGAAMAMPPDPKQAFKAEWEALEICEHQWTLDNVENMALYKGAASEAGRAMQLKKKREKALEELEHQKKKIEEELKLSNIGNKFASHYDAVEQQLKSSTIGLVTLDQMRARQEDVVKERERQLAQKQEFLRQQEIEAKRRKKEQQKRQIASLSFKIDEKKIGKNPNVDTSFLPDKEREEEEQRLREELRQQWEEKQQKLKSEEILITFSYWDGSGHRRSVKMKKGNSIYQFLQKCLELLRKEFNELRAVMADQLMYVKEDLIIPHHYTFYDFIVTKARGKSGPLFSFDVHDDIRLVSDATVEKDESHAGKVLLRSWYERNKHIFPASRWEPYDPTKNYEKYSISDKKK---LVSETLLKKRKKRAEQKAKAIQNAVRERKKQRQRKKGGFKSAEKLVKEYRRIERDNKRLKWAMRCPAKFDFPTEHRLGFIIRIRGSDGVCSRTSKILDLLKLRAVNIGVFVEMNEAMLQLLKVIEPFVTWGYPNLKTVRELIYKRGYGRAMGRRFPLSDNAFIEERLGKYGIVCLEDLLHQLYTIGPHFKKVANFLWKFKLNPPKNGWTKKSLRFTEGGDYGNRDIKINDLLRKMI--------------------------------------------------------------------------------------------------NRGWIAMLIVGIISVTQVPYFIDTFDGFDTKVLEMKTVFNMKANFGRTRRMSALCVTGNKNGLAGFALGKSPEGRAAMRKTKKVAVLEIIF------PVFHDFYTCFGKTQIFVKKKPKGFGLVCHRAIKTICEIIGIKDLYAKVEGS-TNINNLTKAFFLGLQNQKTHQQLAQEKGLHVVEYREERDNFPQVIASPSKCRTMKEINPQEVIDYNEHINEGKVELIKTKRRPFYEYLDSWEYRVKKIERFRNHENVHVNLLVEHGKLTSFLNIEQVNTESNE--MDGQVRWTNKQRVLIFASRGITYRDRHLMQNLQSLLPHSRKESKME--KKDINEICEMKNCNKSIYFENKKKEDLYMWVSNVPRGPSAKFLVQNVHTMEELKLTGNCLKGSRPLLSFDKNFDKVPLLKELLVQVFGTPYHHPKSQPFTDHVFTFSILDNRIWFRNYQIMEE-DGSLVEIGPRFVLNPIKIFEGSFGGPVLYSNPKYISPNVHRAMLKKMSSGRYKLDEIFTTKKTFFRMAVGKNKGLVKGGKKGAKKKVVDPFTRKDWYDVKAPAMFSVRNIGKTLVNRTQGTRIASEGLKGRVFEVSLADLQNDEIAFRKFRLIAEEVQGRNVLTNFHGMNLTTDKLRSMVKKWQTLIEANVDIKTTDGYLLRVFCIGFTRKWPNQVKKTCYAQHTQVREIRKKMVEIMTREVSSSDLKEVVNKLIPDSVGKDIEKACQGIYPLHDVMIRKVKVLKKPKFELGKLLEIHGEGAGPTTTEGTVIERPEGYEPPIQEAVIKVGIVSGSGLD-DPDILQDETPYGKPSDA-LVTGKICGVETVLVARHGRKHSINPTEINSRANIWALK-QEGCTHILATTACGSLREEIHPGDFIPDQFLDRTRASTFYVCHITMHTPFTRQVLKDHESGTVITVEGPRFSSRAESNLFRCWGADIINMTTVPEVVLAKEAGLCYAAVAMATDYDCWH-DEQEAVHLEAVKVLKENGQKAIELLKKAVEIIVASCVLMKILRHEEFEEGCKAACNGPYDGKWSKTMVGFGPEDNHFVAELTYNYGVNQYQLGNDFLGITIKSQTAIENAKNLNWPVKEDNNCYVIEAPGGYKFYLINDSQPQEKDPVQKVTLASSDLKKSVDFWHGLLDMKIYSQEEKTAVLGYSDNQCKLELCDIGTAVDHAKAFGRIAFSCPAEELQQIESKMKEEGQTILTPLVSLDTPGKATVQVVILADPDGHEICFVGNEAFCELSKVDPKADQLLEEAMAADKSDEWFAKKGISKAQARLPPLPTIKDIIRLYKLRALRQLSQNFLLDLLSDKIVKMAGHVCEVGPGPGNITRAILERGADRVTVIEKDRRFLPSLEFLASATN-GRLHIIIGDVLEYDIGHLF-NRELAKDWNDFHIIGNLPFNVSTPLIIKLLRQVSNREGPFAYGRTKMTLTFQEEVAERMIADQRCRLSVMCQNCKVEHGQAFLPKPKVNVGVVLLTPKQPLILPFEIVEKVARCLFHKYCKRDLVEETIRLADIDPTFHLSVEEVGQIAKAYDYICQQPKLKNYEFRMGPLIPVVIEQTGRGERAYDIYSRLLKERIICVMGPITDELSSLVVAQLLFLQSESNKKPVHMYINSPGGSVTAGLGIYDTMQYILPPIATWCVGQACSMASLLLCAGAPAMRHSLPNSRIMMHQPSGHAAGQATDIQIHAEEILYLKKKLNSIYVKHTNQPIEKIETYIERDRFLSPEQAKEFGLIDFVLEHPPMRIDRPIGTWLLLWPAWSIGLATPAGHFPDLALFAAGALLMRGAGCTVNDMWDKDIDKQVARTKDRPLASGQLNMIDAWVLLGGQSLALFVLLQLNWYSIVLGASSLGLVVTYPLMKRFTYWPQVMLGLTFNWGAFLGWSSAVLPLYTACIFWTLIYDTIYAHQFKMFGLTRSMTQQKIRCENNNKNIIITVSTQHINYVTFFFYYAYSLKKHNILTILGICLKHSMFIYISRKPQTRKGKLYLENKEPKVQENAKAIFVRGSTASEVVLKAMKDFGNVHKPHGVFFNRKNPFEDPLSLEFFKKNDASLFMFGSHNKKRPHNLIIGRMYDYHVLDMLELGVESFQPLAEFEGPKIPVGTKPVLLFAGETFEDYQRLKNLFLDFFRGSRVRLQGLEHVIIFIAVEIYWRNYRILMKKSGTKIPR-IELVEMGPRMNLSVRRTKLASEDMFKLAKKQPSVLKPKKKKNVKQTALGSTVGRIHMKRQDFKLQTRKIKSLKVHLLPALSDNYMYLLVTKEAAIVDPVEPEKVTAAVNVKLTTVLTTHHHWDHAGGNEKLVVFGGDSRIGALTHHGD-EFKVGSLIRCLFTPCHTSGHICYFVVFTGDTMFVGGCGRFFEGSPEQMYQALLGALPDNTRVYCGHEYTVKNLKFAQTVEPENTIKEKLSWAEQEPTIPSTIGEEKTFNPFMRVAVQKHSDPIKTMAIIQLKNSNFVMNRLFGRSKPKEPPPNLTDCISNVDSRGESIDKKISKLDQELVKYKDQMKKMRDGPSKNVVKQKALRVLKQKRMYENQRENLMQQSFNMEQANYATQMLKDTKTTVEAMRTGVKEMKREYKKVNIDDIENLQDELEDMMGQAEEVQEALGRNYGMPEVDDDELEAELDALGDEIALDDTSYLD???????????????????????????????????????????????????????????????????????????????????????????????????????????????????????????????????????????????????????????????????????????????????????????????????????????????----------------------------------------------------------------MEQMLRTFQEDLGSISHEILTLQEQSVSMNIRLKNRQAIRGELSQFVDDMVVPDVLINHILETPVVEKDFLEQIYVLDHKISFVKEQAFKEVHSCQDVRDILDKLKFKALSKIREYLLQRIYSFRKPMTNYQVTQDTMLKFKFFYQFLMTHEREVAREIRDEYVDTMSKIYFSYFKSYTTRLMKLQFEETADKDDLMGVEDTARKNILLKLKLKKNATIFTLGDRGNVLTTDLESPILVPHTAQKNETKYTFESLFRSQQYALVDNACREYLFLTEFFMVSGTGAQDLFHAVIGKTLTMFMKHVDVYIQDCYDSIAVFLCIHIIHRFQLLMHKRAVPALDKYWEALLQLFWPRFEYILQLNIQSVRDCDPQKFGSFDLRPHYITRRYAEFSAALVGINENFPSEQVTRLLAALQGEVENFILRMAAEFPGRKEQLVFLINNYDMMLSVLLERTREDSKESESFKELLNARTHEYVEEILSPHFGGLISFVKMCENYLERGQSEHLKKEEKKVPQLVRSFNSGWKGSLDDINKDIMKSFTNFKNGTNILQVALTQLIQYYHRFHKVLSQNPFKNLPIRSELINIHHLMVEVKKYKANFQQYQVWDACNEPCFSLRLTKKIIMSLYREDTRKDKYFVVLQITGPDGKVIYKGERETNGKYTFAAHMDGVYTYCFSNAMSTMTPKVVMFSMDVGNTPK---EQDNDAHQNKLEDMIKELSTSLTAVKHEQEYMAVRDRIHRAINENTNSRVVLWAFFEAIVLVCMTGGQIFYLKRFFEVRRVVMRLFGILRLPKDYSNFPKQYVERAMEQVGWRTPKGPQYLRKEIKKKYNMSRPWTEEFRKNEPGTHIFLEPVFRGDRVEVMVGKDKGKQGIVNYIVKERNWVVVEGLNCHEGPLLVTGQVSLVDPSDNKPSEVEWRYTDEGEKVRVSVRTGRIIPIPKAEETHDYKYLEQPKDTKSEEISKITFEP-KLMTFEQDIMNHLGIKEERQPHRTYWYTSNKVSALLWMTRMTTMFLTFCYLIPNPYSCYYKALMSNAATSALRLHQRLPNFQFNREFLALLLIEDSCHYLFYSLIF-LPIT-LVLLPIFLFALLHSISILDKLGSLVG---QQINILRLVAFTEIFLMPLTLFMIFTGGSLLTPFVYYRFLTLRYASRRNPYTRNVFHELRLSIEMQANPRCPETLRRIAFVTRLAPTILVAIIVYYNFPELQEDEKLHIKLPKNIDDAKNLGKVLSRYTGDTVIGGYFLTYIFLQSFAIPGSIFLSILSGFLFPFPLALTLVCLCSALGASFCYILFYLVGRKLVLKYLPHLLNYIIFLRITPFLPNWFINITSPVIDVPLAPFFLGTFLGVAPPSFVAIQAGTTLHQLTSSFSWTSLTVLIVFALLSMIPVLFKLKEKF------MERVCVAHHENRTTDRISLYYKKTQLIVKITKIIFFWEKKDKRPTILTINFMKCSFCCYKCPFEAISIINLPSNLEKDTTHRYNQNSFKLHRLPTPRPGEVLGLVGTNGIGKSTALKILAGKLKPNLGRFNAPPDWTEILTYFRGSELQNYFTKILEDDLKAIIKPQYVDQIPKAVKGSVQQLLDKKDELRNQDTVCEMLDLKNVRDRNVENLSGGELQRFACAMVCIQKADIMIIIMCTMYLYLRQLFTCVRSQFFTVSVNIVIMMLMIDMRFVSYIQNFIKLLFIDRACYNYKCFYFFFTIGINIFLDGFVPTENLRFRETSLIFKVAETAEEEIKRMCRYEYPTMKKKMGNFSLSVEPGTFTDSEIVVMLGENGTGKTTFIQLLAGRLKPEGGCEIPSMLISYKPQKISPKSQGTVRQLLHEKIRDAYVHPQFVADVMKPLQIDNIIDQEVQNLSGGELQRVALVLCLGKPADVYLIDEPSAYLDSEQRLVAAKVIKRFILHSKKTGFVVEHDFIMATYLSDRVIVFEGQPSVKAVASSPQTLLAGMNRFLELLNITFRRDPNNFRPRINKLNSVKDTEQKKSGNFFFLDD?????????????????????????????????????????????????????????????????????????????????????????????????????????????????????????????????????????????????????????????????????????????????????????????????????????????????????????????????????????????????????????????????????????????????????????????????????????????????????????????????????????????????????????????????????????????????????????????????????????????????????????????????????????????????????????????????????????????????????????????????????????????????????????????????????????????????????????????????????????????????????????????????????????????????????????????????????????????????????????????????????????????????????????????????????????????????????????????????????????????????????????????????????????????????????????????GLIIILIVLLIATGKVGAKKRKKLEMKAEKRAQREREEEEREERRQRNALEEERKKEEEKQKEEEQKREEEEKRVREEKEKQEYEEYLKMKEAFSVEEEGYDEAADEQESHNKLQEFVDYIKTQKVVLLEDLAAKFKLKTQEAVDRVQELLSQERLVGVIDDRGKFIHITREELESVAQFIHLRGRVSITELMESSNNLINLQPDVEHLEAEAKATAAKHVANMLQRPDQLEKVEQYKRRVIRKKASVEAMLKTAMQSQLDGVKTGLSHLETALSDIKEIKQRDIEDSFEIPRLEKLQDVREESLKHSQYAAAMENLKHIFNVPESVQKTQEWISDGKLLLAHQLADLENSRDDLLFEMHKLPNQSPTDRNMLKQYFSDVEKLSEDLGKQLWLILRRTLNSVRKEPQVIVTALRIIEREVRAAIARQQTT-GFMPTGRPKRWKKRCFEILEMSVQDRIEGNQFEERSQNKMWLVRHLEVTRQLILEDLKVVKTACVPCFPPSYDIVEKVKMYHDSLSKHLQDIISQLEGNEYITLLNWLNVYESSELMGHPDLMIDVSLLDNVVENLICRYVSMLETNYQDWMKNTVMDVKDWPEADSEGYFHTSLPVIMFQMIDQHIQVAKTVELVERVLMVSIEQVVSVTRLYKEAIVEYHFEDRSFTHYMIAIANNCLQFV-FESLKMDTLGYLEDELFLDLDKINDLLTDTICATVEDYCHDYIQNRVAKGYITAIL--QKKLSLKNYEERKEVADKKKLRESPFDALPLLAEVLKLKDGSLLSLEISGSRTDDEVVQREEEAIKLDGLNVAQVKELREKAEKFVFQAEVNRMMKLIINSLYRNKEIFLRELISNASDALDKIRLLSLTENEALSATEELSIKIKVFFDIHSEHSTDSKVFNTQ-----NLLKVCESFQSQFVKVIKK----KNMGHIWGQFGVGFYSAFLVADRVVVTSKHNDD-KQYIWESDSGSFSIVEDPRGDTLKRGTTVSLYLKEEARDFLEEDTLKQLIKKYSQFINFNIYLWTSKTETVEEPVEED--EEEKEKP-ETEEDGKVEEE---EKPKTKKVEKTTWDWELMNSSKPIWQRKPADINEDEYKDFYKSITKDTSDPLTKTHFIAEGEVTFKSVLYIPSSQPTETFNRYGSKVDHIKLYVRRVFITDDFQDMMPNYLNFIRGVVDSDDLPLNVSRETLQQHKLLKVIKKKLVRKTLDMIKKIPKEDYEKFWKEYSTNVKLGIIEDPTNRTRLAKLLRFISSN-DPEKLTSLAEYVERMKEKQEHIYYIAGSSLEEVKKSPFVERLLKKGYEVLYLTEPVDEYSVSALPEFEGKKFQNAAKDGLKLDESGKAKERLEDLEKEYEPLTKWLQDESLKDKILKAKISQRLHNSPCALVASQFGWTGNMERLARSNAHSKTYDTTRDYYLSQKKTLEINPRHPLIKELKKRIEDDKDDPTAKNMANIMFETATLRSGYILDDTFSFAERVETLLRKTLGVPEDAQIEDEP???????????????????????????????????????????????????????????????????????????????????????????????????????????????????????????????????????????????????????????????????????????????????????????????????????????????????????????????????????????????????????????????????????????????????????????????????????????????????????????????????????????????????????????????????????????????????????????????????????????????????????????????????????????????????????????????????????????????????????????????????????????????????????????????????????????????????????????????

'Ixodes_scapularis' PDSRVNSLNAEVMESAVLISSKPGCFIAGADITMLDKVTSHASELKPIVAAIMGSCLGGGLEVAMACHYR-IAVKERKTVLGLPEVMLGILPGAGGTQRLPKLIQLPTALDMMLTGRNIRADQAKKMGLVDQTVELLGPGLYLEEVAAKGLASNTLTPRTRPLMERLARNY-VFEKAKGQVMKMTQGLYPAPLKILEVVRTGIEKEGFGELVTPQSKALIGLYFGQVTCKKNRFGEPVKTVGVLGAGLMGAGIAHVSVDKGYNVLLKDGLGRGEQQIKKKRLEKDRFMSALEPTLKYDDIVIEAVFEDIGIKHAVLKEVEQPHCVFASNTSALPIAKIAEASKRPEKVVGMHYFSPVDKMQLLEVITTDKTSQDTAAMAVDVGLKQGKVVITVKDAPGFYTTRILSAMMAEALRVLQEGTTVKELDSLKKFGFPVGAATLMDEVGIDVASHIAAKEFGARHDLVAGFHGRKSGKGCYLYRPTNTLLKKYTVEELQMRLAGRFVNEAILCLQEGVLANPVEGDIGAVFGLGFPPFLGGPFHYVDTYGADRLVQLDKFAQFEPCQLLRDHA-NDTAKKFHMQAPIILLKEGTENSQGKSQVISNINACQVIADAVRTTLGPRGMDKLIVDNSGKATISNDGATIMKLLDIVHPAAKTLTDIAKSQDSEVGDGTTSVVLLASEFLKQCKPYIEEGIHPQIIIRSLRKAAHLAVSRIREIAVAVEKNGQRELLEKCAMTTLSSKMIAGQKEFFARMVVDAVMQLDELLPLNMIGIKKVQGGALEESLLVSGVAFKKTFSYAGFEMQPKKYQNPKIAMLNVELELKAERDNAEVRVQNVQEYQNVVDAEWEVLYEKLRKIHESGARVVLSKLPIGDVATQFFADRDMFCAGRVVDEDLRRTAKACGGAILSTVFDLQESNLGRCELLEEIQIGGERYNLFTGCPETRTVTIILRGGAEQFIEETERSLHDAIMIVRRALKNDAVVAGGGAIEMALSKHLRDYSRSVAGKEQLLVAAVAKALEVIPRQLCDNAGLDATTLLNKLRQRHAAGDLWAGVDIQAGDVADNLAACVWEPAVVKTNALVAATEAACLVLSVDETIKAPKSQGDDERGRPFMPERSAAVASKEHALAVSRDYISQPRLVYKTVCGVNGPLVILDEVKFPKYAEIVHLVLADGATRTGQVLEVSGSKAVVQVFEGTSGIDAKNTVCEFTGDILRIPVSEDMLGRVFNGSGKPIDKGPPVLAEDFLDIQGQPINPWARIYPEEMIQTGISAIDVMNSIARGQKIPIFSAAGLPHNEIAAQICRQGGLVKLPGKSVLDDNFAIVFAAMGVNMETARFFKQDFEENGSMENVCLFLNLANDPTIERIITPRLALTTAEFLAYQCEKHVLVILTDMSSYAEALREVSAAREEVPGRRGFPGYMYTDLATIYERAGRVEGRNGSITQIPILTMPNDDITHPIPDLTGYITEGQVYVDRQLHNRQVYPPINVLPSLSRLMKSAIGEGMTRKDHADVSNQL-------------------------------------------GSYENRTIFESLDIGWQLLRIFPKEMLKRIPQALLQEFYPRDGRQTGLFGEPNGFYLLKEQAIQDAETYVDEATDRSRKMVQVFDDLSDALCRIADMAEFVRIGHPENRFSSAALDASVSISNLVEKLNTNKNLYEALKRVTEHG-DVVPTTEDDYVSKLFLFDFEQSGIHLDAETRKKVVSLNDYTLHVGSYFANNALQARAVKKSELPENIVIVPGLYADANNELLREAAYRAYLYPDKHQSELLDELLAARHQLAVLCGFPTYAHRALRGSIAGSPDGVEFLGILSAQLKPRAEQDYKEMLNMKPWDVPYYTPYLSLGCCMDGLNEIFHSLYGISLEAEGEVWHPDVVKLAVKEENRLLGYIYCDFFERQEKANQDCHFTIQGGRLPDGSYQTPIVVLMLNLPPPLLTPSMMDNLFHEMGHAMHSMLARTRYQHVTGTRCATDLAEVPSILMEYFSSDPRVVSMFARHYQSGEPMPYQMALSLRHLRYHFAASETQLQVLYALLDQRYHSHHPLSTTQVLAELQDQHYGLPYVEDTAWQLRFGHLVGYGAKYYAYLVSRAVAAWTWQEVFKRDPFHKEAGNYRQKLLSHGGSVPAKQLVSDFLLATFLISDI????????????????????????????????????????????????????????????????????????????????????????????????????????????????????????????????????????????????????????????????????????????????????????????????????????????????????????????????????????????????????????????????????????????????????????????????????????????????????????????????????????????????????????????????????????????????????????????????????????????????????????????????????????????????????????????????????????????????????????????????????????????????????????????????????????????????????????????????????????????????????????????????????????????????????MVLADLGRKITNALRSLSNATIINKEVLDSMLKEICTALLEADINIKLVKQLRENVRSAIDIDEMGAGLNKRRMIQSAVFKELIKLVDPGVKAWQPTKGKNNIIMFVGLQGSGKTTTCTKLAYHYLKKGWKTCLVCADTFRAGAFDQLKQNATKARIPFYGSYTEVDPVVIAQEGVDKFKSEGFEIIVVDTSGRHKQEDSLFEEMLQVSNATNPDNIIFVMDASIGQACESQARAFKEKVDVGAVIVTKLDGHAKGGGALSAVAATHSPIIFIGTGEHIDDFEPFKVKPFISKLLGMGDIEGLIDKVNELKLDDNEELIEKLKHGEFTLRDMYEQFQNIMKMGPFSQIMGMIPGFSSDFMTKGNEQESMGRLKKLMTIMDSMNDQELDHREGAKLFSRQTGRVTRVARGAGVTTREVQELLAQYTKFAAMVKKMGGIKGLFKGGDMAKNVNPAQMNKLNQQMAKMMDPRVLHQMGGFSGLQNMMRQLQGAFLLDDPSYKKLQEYYNAKGKTLNMPALFKEDPERFRRYSVRLKTPVDGLLVDYSKNLINHDVMTRLFDLARSRKVEPARDAMFQGEKINFTEGRAVLHIALRNRSNNPIMVEGKDVMPSVNAVLKHMREFSDSVRNGVWKGYTGKSITDVVNIGIGGSDLGPLMVTEALQPFQKGPNVHFVSNIDGTHLAKTLKKLSAETTLFIIASK------------------------------------------------------------------------------------------------------------------------------------YLHRFAAYFQQGDMESNGKYVTRSGQRVAYTTGPIVWGEPGTNGQHAFYQLIHQGTRLIPCDFIAPVKTHNPISHGIHHEILLANFLAQTEALMKGKTKEEAEAELKKAGMTGEVLQKILPHKVFEGNKPTNSLMVDKMTPYSLGMLIAMYEHKIFTQGIIWDINSYDQWGVELGKQLAKVIQPELRGKSEVSTHDGSTNGLINFIKSYNEKATYKDKDKPSQVRQSNITAAKAVADAIRTSLGPRGMDKMIQAANGDVTITNDGATILKQMQVLHPAAKMLVELSKAQDVEAGDGTTSVVVICGSLLDAAAKLLLKGIHPTIISESFQRAASMAVEVLESMAHPLDLSDRDSLLKSATTALSSKVVSQHAGQLAPMAVDAVLRVVDPTNVDLRDIKVIRKLGGTVEDTELVDGLVFTQRLAGSGGPHRVEKARIGLIQFCISPPKTDMDHQVIVSDYTAMDRVLREERAYILNIVKQIKKAGCNVLLIQKSILRDALNDLALHFLAKMKILVVRDIEREEVEFVCRSLGCRPVASLDHFGPEALGSAELVEEVASAKYIRVTGVAHP---RTVTLLVRGSNKLVLEEAERSLHDALCVVRCLVKRRALIAGGGAPEMEISLRLAERARLLSGLDAYCVGAFAEALEVVPYTLAENAGLHPIATVTELRNRHAKGERDAGINVRKGSVTNILEENVVQPLLVSTSAVTLAAETVRSILKIDDIVQTVRMDKKNVMYFWDPDVGNFHYGPGHPMKPQRLSVTHSLVLHYGLYKKMQASQLSSSNSHDMCRFHSEEYIDFLERVTPQNIQTFTKSLSHFNVGDDCPVFDGLYDFCSMYTGASLEGAVKLNNECCDIAINWSGGLHHAKKFEASGFCYVNDIVIAILELLKYHPRVLYIDIDIHHGDGVQEAFYLTDRVMTVSFHKYGNYFFPGTGDMYELGAESGRYYSVNVPLKEGIDDASYFQVFKPVIQGVMEFFQPSCIVLQCGADSLAGDRLGCFNLSIRGHGECVRFIRELGLPLLVLGGGGYTVRNVARAWTYETALLVDEPVSSEIPYNEYFEYFAPDFTLHPE-------ENANSKQYLETIVRAVSENLKCLVHAPSVQMHHVPPDMLKPGST-SAVPTPVEPALSTTPTAAATVAEMSDEDDFMCDDEEDYDLEYSEDSNSEPDVDLENQYYNSKALKEDDPTAALASFQKVLDLEAGDKGEWGFKALKQMIKINFKLSKYEEMMARYKQLLTYIKSAVTRNYSEKSINSILDYISTLAEMELLQEFYETTLDALKDAKNDRLWFKTNTKLGKLYFDRSEFNKLAKILKQLHQSCQTDDGADDLKKGTQLLEIYALEIQMYTAQKNNKKLKKLYEQSLHIKSAIPHPLIMGVIRECGGKMHLREGEYEKAHTDFFEAFKNYDESGSPRRTTCLKYLVLANMLMKSGINPFDSQEAKPYKNDPEILAMTNLVSAYQNNDISEFELILKTNRRNIMDDPFIREHIEDLLRNIRTQVLIKLITPYTRIHIPFISRELNIDSNEVENLLVSCILDSTIQGRIDQVNQVLELDSKGQGAARYNALDKWTAQLGTLHQTISSFIANVGSLNAVRILKGEAEEEKAETARLSSFVGAIAIGDLMKSTLGPKGMDKILMCNNRGESKVEVTNDGATILKAIGIDNPAAKILVDISKVQDDEVGDGTTSVTVLASELLKEAEQLISKKLHPQTIVAGWRKATAVARQALEAFAADHSANAEQFREDVLNIARTTLGSKILSQHQEFFAKLSVDAVMRLKGSGNLDAIQIIKKLGGSLTDSYLEEGFLLDKKPGVHQPKRVEKARILIANTPMDADKVKVFGSRVRVESVAAIADMELAEKVKMKEKVDQILKHNISVFINRQLIYNYPEQLFADAGVMAIEHADFDGIERLALVTGGEIVSTFASPELVRLGTCDLIEEVMIGEDKLLKFSGVPLGEACTIVLRGATQQILDEAERSLHDALCVLSQVVKDNRVVFGGGSSEMLMASAVSKLAESTPGKESLAMEGFARALRQIPTIIADNAGFDSAQLVSELRAAHSEGKKTAGINMTEGCVDDMCKLGVTEAFVVKRQVLLSASEAAEMILRVDNIIKAAPRQRHPDRSHMAAEIKPGSKPQLINKLVGHQEAVNVAVIIPGEDGVISISDDRTVRVWLRRDTGQYWPSICHNMPSAASSMDYNSETRRLFIGMDNGSISEFLVADDFNKMTHQRNYLAHQGKVVGIVFSLIAEWVLSVARDKYFQWHCSETGRRLGGFQCNAWCTALQFDAQSKHAFIADYSGHITMVKVEETGYKPVTTLKGHSGSIQCLSWDAERKLLFSGGFDQTIIVWDIGGKQGTAYELQGHHNKVTALCYHSPGKLISASEDSTLVFWNMTTKRIETPEWGESDTCQRCSRPFFWNIKAMMDQKTIGIRQARRSLCGKAVCDKCSSNRSRIPIMGYEFDVRVCDECHVIITDDDRVSMATFHEARHPVVHMHLDVTRGHLLTTGSDRVIKIWDISHMLSKKALKKQAKEQEKAAKKSSRQSAESKQAVDTEVDYSEGHYGKMAMLQSSEKVSRELAKVKDLSSSIAGSYIWIRGRLHTSRSKGKQCFFVLRQQQYTVQCLVAVSEKVSKAMVKFCALVTKESLLDVYGIVQSSPQKVEGCTQQDIELHIEQAGAPSRFERMLYFQLDQNRTPAQD----EGLNIRVNQDTRLDNRILDLRTPANQAIYRLQAGVCSLFRESLAARGFVEIHTPKIISAASEGGANVFEVSYFKGSAYLAQSPQLYKQMAIASDFDKVFTVGAVFRAEDSNTHRHLCEFVGLDLEMAFNYHYHEVLDIIGQMFVDIFKGLRDRQATLICVMDFTYYCRFLHSRKQSLRLEFPEAVAMLRAAGVEMDDEEDLSTPNEKLLGRLVKAKYDTDFYILDKYPLKVRPFYTMPDPHNAKVSNSYDMFMRGEEILSGAQRIHDPTFLTERAKEHGSSVTLLQAYIDSFRYGVAPHAGGGIGLERVTMLYLGLDNIRKTSMFPRDPKRLTPMLKEGLSVADIPPSEFDRIKIIYPAECRNRGTTYRGKLDVTLWSLNGVQQDVIKKTAGEVPIMVKSQMCNLSKLSPKELVQRGEESEEFGGYFVVNGNEKIIRMLIMTRRNYPIAMMRNSWKNRGKMYSEYGVSLRSVKPDGTNMVLHYLTNGTVQVMFSYMKEIFFMPVMMLLKALCDVTDYHIYSELVAGKENDSFYKGCIINMLRQVQENLLTDEVRSYIGGKFRWYSDEQVALFLLRHCICIHLDSNFDKFNLILLMIKKLFALAKGECAIESSDNPMNHEVLLAGHLYLMVLKEKLGSFLFSIRQNIEKKFSKVLEVTGAMNYFLATGNVVTKSGLGLMQFSGTTVLAEKLNYWRYLSHFRCVHRGAFFAEMRTTTVRKLLPEAWGFLCPVHTPDGSPCGLLNHMTAMVEMVVVLDGRVLGYVLRTMKAHGKVPPTLEIGFVPKTSKASQYPGLFLFSTVARMMRPVLNATGTVEWIGTFEQVHLNISVIPDEATTHQELRQTSMLSVLGNMIPYSDFNQSPRNMYQCQMGKQTMGTPCQALRYRSDNKLYRITPQTPFVRPTAYDHYHMDDFPTGTNAIVAVISYTGYDMEDAMVLNKSSVERGFKHGVYKTELVNLRVDLDGLPYIGVQNDPVCSFVETKVHKYKSTESAYIHDVKLLGNLQNICITYWIRNPMIGDKFASRHGQKGVCSQLWPIENMPFTESGMTPDIIFNPHGFPSRMTIGMMIESMAGKSATLHGFVHDATPIEIEDISMFVQCKREFLLAAGYNYHGTERMYSGVDGREMEADIFFGVVYYQRLRHMVADKYQVRTTGPVDTLTHQPVKGRKRAGGIRFGEMERDSLLAHGSSFLLQDRLFNCSDRSLCRKCGSVLSISMPYVFRYLAAE-LAGINMGVLRETKHDIHKVQRNDPSLHPFEVCREYTRAMNAVKLDKIFAKPFLGSLGHRDGVQVLQKHPKSLSHLISGACDGEVKIWNLAERKCVRTIQAHRGMIRGITGDDKNIKTIITKISHHQENLFATCGEDVHLWEERSEPLRTLW-GVDTVYVRFNPIESASSDRSIVLYDTRESQPLRRVILEMRSNALCWNPMEAFIFTCANEDYNLYTFDMRRLKSPLSVHMDHVSAVMDVDYSPTGKEFVSGSYDKSVRIFHSREVYHTKRMQRLTCVLWSLDNKYIMTGSDEMNIRLWKACASEKLGMLQYQAKLKEKFAQHPQVKRIARHRHVPKHIYQEKRTMLEARKRKLGRQLLSKGQYADALSHYHAAVEGDPENYLNYYKRATVFLALGKSKPALEDLHEVIALKPDFLAARHQRGTVLLKQGNLDEAHIDFEWVLRLDPNNPDASIEPLKRDAIEVLTRVWDVTLREMRASCYENLGDVMNAIMDLRPTTKMVPDNTVGYLKLSKLYYKLAEAEESLNVIRECLKLDPDHKDCYKKVKKLARSIQDECVDKLVQHVRGRCHCHSKAGEALETCSEALKLDPALCDRAEAYLNDGQYDHDFQQAANADE-GPEGLKRAQRLERQSKKRDYYKILAAKREILKAYRKLAQKWHPDNY-QGDS--KKDAEKKFIDIAAAKEVLTDPEKRKRFDSGEDPLDPESQFTYKFVFMLVLFESPAGYAIFKVLDEKKLQQTDNLFKDFETPDKASRVVKLKHFEKFEDMTQALAAATSAIEGKMSKTLKKVLKKVVAKEAHESLAVADAKLGNVIKDKFDISCVANSSIQELMRCIRSQVDGLITGLPRKEMTAMALGLAHSLSRYKLKFSPDKVDTMIIQAVSLLDDLDKELNNYIMRCKEWYGWHFPELSKIVTDNLQYVRTVQKVGLRTNAIETDLSDILAEDLEAKVKEIAEVSMGTEVSDEDIRNILHLCEEVLQMSSYRSQLYEYLKNRMTAVAPNLTILVGELVGARLISHAGSLLNLAKHPASTVQILGAEKALFRALKTKHDTPKYGLIYHAQLVGQSTQKLKGKMSRMLAAKASLATRVDALGEDGGTELGVEHRAKLETRMRVLEEGGNRRIS???????????????????????????????????????????????????????????????????????????????????????????????????????????????????????????????????????????????????????????????????????????????????????????????????????????????????????????????????????????????????????????????????????????????????????????????????????????????????????????????????????????????????????????????????????????????????????????????????????????????????????????????????????????????????????????????????????????????????????????????????????????????????????????????????LNVPETRVTTLDNGVRVATEDSGNPTCTVGVWIDAGSRYENEKNNGVAHFLEHMAFKGTSKRSQTDLELEVENMGAHLNAYTSREQTVYYAKCLSKDMPRAVEILSDILQNSKFGEAEIERERGVILREMQEVETNLQEVVFDHLHSVAFQGTPLGLTILGPTENIKSIQRQDLVDYISLHYKGPRIVLAGAGGVNHDELVKLASQHFGSMTTDYDAK--LPCRFTGSEVRVRDDDMPYAHVAIAVESCGWADPDNIPLMVANTLIGNWDRSHGGGANVSSRLAEECVKDNACHSFQSFNTCYKDTGLWGIYFVSEGREMDFLVHAIQREWMRICMSATEGEVTRAKNLLKTNMLLQLDGTTPVCEDIGRQMLCYGRRIPLPELEARIDAVTAQTVRDVCTKYIYDRCPAVAGVGPVEALTDYANVRSNMYKHSVHTLVFRSLKRTHDMFLSDQANPPPQDETSEKLKVSIKTKDEYGLVMHLVKSNNRPNVEQMMVLAGQLAVAPRKPVTIPKPQWHPPWKLYRVISGHTGWVRCLAVEPGNQWFCTGSNDRIIKIWDLASGKLKLSLTGHISGVRGLAVSPRQPYLFSCGEDKQVKCWDLEYNKVIRHYHGHLSGVYALSLHPTIDVLVTGGRDATGRVWDMRTKASIHSLVGHSNTVASVQSQAAEPQVLTGSHDCTIRLWDLVSGKSRVTLTHHKKSVRALLVHPKLYAFASGGPDNIKQWKCPDGKFIQNLSGHNAIVNCLGMNDDGVLVSGGDNGTLFFWDWRTGYNFQRLQAPVQPGSIDSEAGIFACLFDMSGSRLITAEADKTVKIFREDDTATEETHPVNWKPDIVVHSSSEFRINVSLDEYLEDLSINVKAKNFLVFQGDVETIAMKNPKERTLLFEEISHSMIKAEEDTYQKKKGIAAEKKEARLEEADKYQRLKEDLQVVCQTFKLYHIQDEMSHKRRKEKVEEEVRDKRKIEQQIREDVELNKKKPAFIKAKERTAHMQKKLEAARKSYKAAKKVDETHQGISELEEELEEINREFEHQDFSLEDSQLREYHQLKEEAGRQASLHLQNLDSVRRDQKSDQDRHDNEVRKRQETQNKARELDENLRRVDKLGELEELRRQEQEVEAKARINRELEALNEMGDAKVDKHEDSRRRKKAEIVDHFKQLYSGVYDRLVNMCQPVHKKYNVAITKVLGKNMEAIVVDSEKTGRACIKYLKEQMLEAETFLPLDYIDAKPLKERLRTISQPKNVKLLYDVLQYDPPAIKRAVLYATNNALVCETADDASRVAYDLGDNKRYDAVALDGTYYQKNGFISGGSTDLAKRARRWDDKAFHTLKQKKEKLTEELKEMMKRTRKESDLTTIQSQIRGLETRIRYSVVKKIEEEMRKRELKIDELKQAQNGIEDRVFSDFCASIGVENIRQYEERELRATQERDKKLEFENQKNRIVNRLEYER--SKDTSEVEDDERELENLKEAEANQMQMIDDQMQSLEKLKSSKLGKKQDLDKAEEQMAEIRKRLAAVQKDIAVVQKSLTSLEARQEQRRLDRHSVLQTCKLEQIPLRLLDGTLTRLYEREQLRIDYSILRLSKEIALQRTQAPNMRAMEKLDGVKERLKETDTEFENARKKAKMAFEKIKRERTCFDRVSNRIDEIYKALTNNQSAQAFLGPENPEEPYLEGINYNCVAPGKRFQPMSNLSGGEKTVAALALLFAVHSFQPAPFFVLDEIDAALDNTNIGKVAHFIREQTETSFQCIVISLKEEFYSHADALVGIVPDPGECTVSRVLTMDLSLSEAMLLYLKKAIQEGNVFEIQNIYENNLAKLMEKYFMILYKE--LYYRQIYADERFESYYNYCALFNYILSTGPVALELPNQWLWELIDDLLQNF--QSFSQFIWNVHSVLNVLHSLVDKSNINLQLEVYTKGGDPDNVAGEFGRHPLYKMLGYFSLIGLLRLHSLLGDYYQAIKVLENLELNKKSLSRVPACQITTYYYVGFAYLMMRRYADAIRTFSNILVYIQRTKRSYQVDQINKQTEKMYALLAICLTLHPQRIDESILSQLKDKYMNKMQKGDKGEFGDCPKFLNPVQLNVFLSEVQGQITIRSFLKLYTTMPVSKLANFLLLSFKHKMKNVVLDGEFQSGSDVDFFIDGDMIHIADTKVARRYGDFYIRQYHKFEELYR?????????????????????????????????????????????????????????????????????????????????????????????????????????????????????????????????????????????????????????????????????????????????????????????????????????????????????????????????????????????????????????????????????????????????????????????????????????????????????????????????????????????????????????????????????????????????????????????????????????QLSSRAKHLLHCCLCVGLILIVEVFTFSYDEVDPVRQYGWPWTIFLYLMRLLTVLALPQCICNCLGLLLYNAFPEKVRLKGSPLLAPFVCIRVVTRGDYADLVRGNVARNIETCADVGLENFIVEVVTDKALGLAKHPRIREVVVPTTYRPKSGALFKARALQYCLEDDVNILSDDDWIVHLDEETLLTEDSLRGILNFAFDGRHAFGQGLITYANERVVNWVTTLADSFRVADDMGKLRFQFWAFHRPLFGWKGSYVVARAGAERRVSFDHGLDGSVAEDCFFSMVAFREGYTFDFIPGEMWEKSPFSFWDFLQQRKRWMQGIFLVVHSGAIPIRHKFLLSLALYSWATIPLSTSNLVLAALWPIPCPTPLNFLCAFVGAMNIYMYVFGVIKSFSLYRLGPVRFLLCLLGAVSTIPFNVVIENIAVLWGCFGHKYRFYPWPSYTFTGKLRPFPLSPKREVPEHIMRPDYAEHPDGIPLSEQAAKHSSVIKVLTEEEIEGVTLASKFSLEVLDVALGAAEVGVTTDELDRLVHEASIERDCYPSPLNYYKFPKSCCTSVNEVICHGIPDMRPLQDGDLLNVDITVYHNGFHGDLNETVFIGNVDKTARKLVEVTHECLSKAIEAVMPGVRYREIGNIIQKHAQSHGFSVVRSYCGHGIHRLFHTAPSVPHYAKNKAIGIMKAGHCFTIEPMISEGTWHDAVWPDSWTAVTADGKRSAQFEQTLLVTDTGCEILTRRRNKNGQPWFMDNNSVRQSLKERSQKRRALLAQQLGAGTASNLSQILGNEREAGPDDAARSPLEEVYTYRDSSTFLKGTQSANPHNDYCQHFVDTGQRPQNFIRDVGLADRFEEYPKLKELIRLKDELIRETAAPPMYLKCDLETFDFRTVKSKFDVILVEPPLEEYQRTCGLAHTKFWSWEEIMRLEIEEVAASRSFLFLWCGSSDGLDLGRQCLRKWGFRRCEDICWIKTNAKNVHLKNLEPRAVFQRTKEHCLMGIKGTVRRSTDGDFIHANVDIDLIISEEPQFGGTEKPEEIMHIVEHFCLGRRRLHVFGRDVSIRPGWLTIGPELTNSNFCSDAYNAHFNGTNDYLTGCTERIEALRPKSPPPK?????????????????????????????????????????????????????????????????????????????????????????????????????????????????????????????????????????????????????????????????????????????????????????????????????????????????????????????????????????????????????????????????????????????????????????????????????????????????????????????????????????????????????????????????????????????????????????????????????????????????MKTFGDKPTAFQLEEDGEFYYIGSEVGNYLRMFRGSLYKKYPSLWRRLVTVDERKKIASLGLGPHSLATSISLLRAVEVDEIFDGKDDKYRAVSVSAEPPVQRETKSKRSTWMPTLPNSSHHLDAVPCSTPVNRNRIAHKKIRTFPLLYDDLDPATLHENSAQPEVLVPIRLDMEIEGHKLRDTFTWNKNESQITPEQFAEILCDDLDLPPLSFVPAISQSIRTQIEAFPTDNLLDDQTDQRVIIKLNIHVGNISLVDQFEWDMSEKENSPEQFALKLCSELGLGGEFVTAIAYSIRGQLSWHQRTYAFSEAPLPTVELPFRSQAEADQWCPFLETLTDAEMEKKIRDQDRNTRRMRRLANTTWFGANGSLLEKEIGFPSNEHYFGLVNFGNTCYCNSVLQALYFCKPFREKVLEYKARNKRTKETLLTCLADLFHSIANQKKKTGTIAPKKFIARLRKENDLFDNYMQQDAHEFLNYLLNTIADILQGAQGWVHDIFQGTLTNETRCLNCETVSSKDEDFLDLSVDVSQNTSITHCLRGFSNTETLCSEHKYYCENCCSKQEAQKRMRVKKLPMILALHLKRFKYTEQQNRHTKLSYRVVFPLELRLFNTSDDAFNPDRMYDLVAVVIHCGSGPNRGHYISIVKSHGLWLLFDDDIVDKIDAAAIEDFYGLTSDTPKTSESGYILFYQSREMLDVDKWIEIAKECKYLPENDLKKLCSMVCRLLLEECNIQPVSTPVTVCGDIHGQFYDLEELFRTGGQVPETNYIFLGDFVDRGYYSLETFTRLLTLKAKWPDRITLLRGNHESRQITQVYGFYDECQTKYGNANAWKYCCKVFDLLTVAAIIDEQVLCVHGGLSPQIKTLDQVRTIERNQEIPHKGAFCDIL-CPGTALHTYRIPPRGAGWLFGAKVTHEFMHLNNLKLICRAHQLVHEGYKYMFDDKLVTVWSAPNYCYRCGNIAAVLAFTDVNTRTAKLFHAVPDTERVIPPRNTPYFLMKSADGELESDPDTLTIEDIKEHARHIEKAVNSKEPRFILRILRALMPTRKRLNAKVMRKIVSGFYTHSNQSRDALLAFLEPMETEKEGRAKTSHLPLLPELDVYIHLLVLVHLIDSERLDQAVKCSDQLMAKIQAQNRRTLDLLASKCYFYHSRCYELTGQMSAIRSFLHGRLRTATLRSDYEGQAVLVNCLLRNYLHYNLYEQASKLVSKSAYPEAASNNEWARYLYYLGRIRAIQLDYSEARRHLLQAVRKAPQHAALGFKQTVHKLAITVDLLLGDIPDRSIFRQPPLRRTLAPYFQLTQAVRAGNLGRFNEVLENFGPKFQADHTFTLIIRLRHNVIKTGVRMINLSYLRISLADVAQKLQLDSPEDAEFIVAKAIRDGVIEASIDHDQGYMQSKENMDIYCTREPQAAFHQRICFCLDIHNQSVKAMRFPPKSYNKDLESAEERREREQQDLEYAKEEEDDGFLILTKILVAMASFVIHVIWSTAIPDLMSSIHPSVMLLLELLTVIPEELQARGPSRESVLQFLSRLVTL--SPSVLHCYHLQLLPRLV-QVENPEAAETLTNVATHPDARFPGFVMEVVSRIVGLEKTLAESLARGDRESIYSLLIEVGECHSHLLVESLLSKPEHKVLKLFRLVLQCSASPGHFPVDESWSRQALGFWYAVQDDVGAWEG-PQGESALHSLWQALLEFLRKARLPLDDTTTDEEKDSLRCYRQDISD--ALMYCYNMLRESLLATLAAHMELAKEWPFAEACVFALQAVAECVGVEQERYVAPLLAEALPALQ-HPRVVPTALSCLGAFGSSCLQATLPMLLRGLEDTAPAATLALKDISRDCSGALGPHGPHILAASQHLGNREKVRVMGLVGHILSSV-GDNARPWLSALVGPQLEALRLARPVFQTVVPLLN---------EQLVMTALCECVRRGAANL-EG---VKLLAVLVTLQLCSARAPNGKVHTVILEAFFQMYSIARKMDLFPVFACAPERSTVKALAEFILHSRPIPLMTVIELLVEQVLRATGSPRSVLDPMADILLALTKKYHWATVLIRTFEKKEHYLRLLLKERTNKRRIKEIV--SELSLKVKVKWKETFSDVEVDTPMVFKAQLFALTGVQPDRQKVMVKGAVLKDNDWGAVVLLMGSKEALPSPAEKPVFMEDMSESELATALDLPSGLTNLGNTCYMNATVQCLRTVPELKELAKFQGSVTAALRDLYGVMDSAVVPPIVMLQVLHMAFPRFSEKSEHGQQDANECWTEMVRMLQNFVDQYFGGIFVTLKCIESEDETTEHFLQLSCFIS-QDVKYMYAGLKSRL-LETIRKMSPTLGRDAEYKMSTISRLPAYLTIQFVRFFYKEKGAVNAKILKDIKFTMQLDMFELCSEALQQKLIPMRTKFKEEEDRFSGRDEGSNNSGYYELQAVLTHKGRSTSTGHYVAWIRREWFKCDDDKVVSADEILKLSGGGDWHTAYVLLYGPRLLEALINKKLPKELLLRIFSYLDVVSLCSCAQVSRLWHELALDGSNWQKIDLFDFQTDIEGPVVENISRRCGGFLKKLSLRGCQSVEDASLKTFAQNCNNIEDLLNGCKKLTDSTCQSLGRVLDLGSCCQVTDLSLRAIGQGCPNLEHLNISWCDQVSKYGVEALAQGCGRLRAFISKGCVNDEAVSQLANGLQTLNLHECTHITDAAVQCVSQHCPKLHFLCVSNCAQLTDASLVSLSQGCLCTLEVAGCTQLTDSGFQALSRSCHALEKMDLEECVLITDSTLLHLANGCPRLQQLSLSHCELVTDEGIRHLGAGVLELDNCPLITDASLEHLVPCLQRIELYDCQLITRAGIRKLRLDLKVHAYFAPVTPRPRYCRCCVVLTSGNARVFCPDHPDAALVEDYRAGDMICPQCGLVVGDRIVDVGTEWRVFPNEKSNNDPARVFLFKNPLLNGSDLATMIGKTGDASDESGTAKYQNRKTMSSSDRALVNAFREISNMADRINLTKTIVDRSNLLFKQVHDGKTLKGRSNDAIASACLYIACRQEGVPRTFKEICAVSKVSKKEIGRCFKLILKALETSVDLITTGDFMSRFCSNLGLPSSVQKAATHIARKAVELDVVSGRSPISVAAAAIYMASQASEDKKSQKEIGDIAGVADVTIRQSYKLMYPRAAELFPDDFKPKDCVPWVEKYRPAKFNEIVGNEETIARLEVFSREGNVPNVILSGPPGVGKTTTILCLARILLGPSFRDAVLELNASNDRGIDVVRNKIKMFAQKKVTLPPGKHKVIILDEADSMTEGAQQALRRTMEIYSKTTRFALACNTSDKIIEPIQSRCAVVRFSRLSDAQVLAKLLDICKQEDVSYAEDGLEALVFTAQGDMRQAINNLQSTFVGFSHVNSENVFKVCDEPHPLLIKDMLQHCVEGELDKAYKIMLHLWKLGYAAEDIISNVFRVCKSHTMPEYLKLEFIKEIGYTQMRTLQGVQSLLQMAGLLSRLCMKTKVIKYMKKEHSLVKPYQGAGMTIPNWDFLGNTMVTGQYIRLTPDRQSSKGAIWNNVPWEMHVHFKVHGSGKELFGDGFAIWYAKEALQLGPVFGSKDFSGLAIFFDTYANQNGPHNHGHPYISAMVNNGTLSYDHDRDGTHTELAGCEAKFRNLDHDTHIAVRYENDVLTVSTDIEGKNAWKECFTVKGVQLPTNYYFGASAVTGDLSDNHDIISMKLFEIDEDRSKIVPSAAPPRDHIEDAMSGTKLFLIVLCAILGLVLIVFQKQQETSRKRFYHVRDSFDEIRTRNCKLVDVNQLFLPNDTVTHVPNIKRLNIDPVFPNRTNLLHLHNMAISRAFFFSYILQKAADDEPGFMYYFMSVIADVAANRFLNSSAIYYAPNMSFTPSYKSFFNKTMPLFAPRAYRADDFNDPYHLEGTSTLNTIEAIDLGAISRNYSSDQYRINEWYHHWLPDLTKRQDSKTTYTVQITGTNETFTWHGPPAASDNPGPVKWTRPYFDCDRSNKWTYGATVPIPDIYPRHTQWRHIEIPKYVAVAVMEMDFERLDINQCPIGSGN-PRPNYFAGTSRCKNDTTECEPVNGYGFRRGGYQCRCRPGWRRPRIVRNPFHGELIERATDQEYKKGYNCEKIGYMMVQTQNLLNHMAIGAHFDPHLSGDVAYGRDQKLENEARSAVRLANFLSGFIQTVDPKELFAEFRVPDRSLTPDQVIAEALSLVIGNEKIQGVGVYFDHKQFFAPY--AYRKTRNALRFFVDDKAAESFRLPSKVVSNEVSPWVRVTPTSGHLFAETGTCGGMGKKAVKTIISWMFYFSSMSGRLYEHAIVERSKRKQKELKQVFYIKLTLKTDITSEACVPILGRGFSGGYKCECLQGYEYPYNDPITYFDGQIVEAPSKYDTL???????????????????????????????????????????????????????????????????????????????????????????????????????????????????????????????????????????????????????????????????????????????????????????????????????????????????????????????????????????????????????????????????????????????????????????????????????????????????????????????????????????????????????????MT--EAPNEFRLQQSPTDAISSLQFGPNSNQFLLVSSWDSNVRLYDVVSNTVRLKYSHKRPVLDCTFQDAVHAWSGGVDCQVKSFDFNCSAETVVGQHTAPVRCIEYCPDVNVVISGSWDSTVKLWDPRSPTNAGTFNQAERVYTMSVCGDKLVVGTALRKVLVWDLRNMGYVKQRRDSNLKYQTRCIRCFPNQQGFVLSSIEGRVAVEYLDPSAEVQKKKYAFKCHRTKDSAGIELIYPVNAIAFHNLYNTFATGGSDGHVNIWDGFNKKRLCQFHKYPSSIAALAFSHDGSLLAIGSSFLYEEDDVEVIPPDAIFVRRLSLDQLKEITTVVADTGDFEVMKQYKPTDATTNPSLILAASKLPQYSHLVDEAVQYGKKPKEKLTNAMDKLVVLFGSEILKIIPGRVSTEVDARLSFNTEASIAKALRLIALYKEAGVDKKRILIKLASTWEGIQAAKVLESEHGIHCNMTLLFNFTQAVACAEAGATLISPFVGRILDWHVANTAIKSFQPLEDPGVLSVTRIYNYYKRFGHSTVVMGASFRNVGQVRALAGCDLLTISPALLSELAISIEPIVQYLSEDKAKKLDLEKVEVNEAIFRWELNEDQMATDKLSDGIRKFAADAVKLENILKEKLQMAYTIARPLVNVYNDKNELSGTHVALPAVFKAPIRPDIVNFVHVNMSKNNRQPYAVSKEAGHQTSAESWGTGRAVARIPRVRGGGTHRSGQGAFGNMCRGGRMFAPTKTYRRWHRRINVAQKRYAICSAVAATGVPGLVLSKGHKIEEIPEVPLVVSDKIQDLKKTKEAVLFLKKVKAWTDVEKVYKSRRLRAGKGKMRNRRRIQRLGPLVVFEADNGITRAFRNIPGVDTLRVDKLNLLKMAPGGHVGRFVIWTESAFRRLDKLYGTYRKPSSEKKNFSLPKPQMTNSDISRILKSDEIRQVIRAPCKKVVRRTQKKNPLKNINAMLRLNPYAAVTRRASVLLNQKQKLKKLLAEKRGVKLEKLQPARRAAAIKKKGRLVQGSLLKKVLEAVKDLINEGTWDCSATGISLQAMDNSHVSLVALNLRSDGFDNFRCDRNLSMGMNLGSMAKILKCAANDDIITIKARDDADTVTFVFETTNQEKVSDYEMKLMNIDTEHLGIPETDYSVIVKMPSAEFQRICRDLSQIGDSIQITCTKEGVRFSVSGDLGSGNVKLSQTANVDKEEEAVIIEMQEAVSLNFALRYLNSFTKATPLCGQVQLSMSADVPLVVEYKIEDMGYVRYYLAPKIDKNHVKVAVLGASGGIGQPLSLLLKQHPAITYLSLYDIAHTPGVAADLSHINTRPQVKGFMGTDQLPESLKGMEIVVIPAGVPRKPGMTRDDLFNTNASIVRDLADACAQACPKAFLCIISNPVNSTVPIASEVFKKRGVYDPNRIFGVTSLDIVRANAFVAEAKGLDPATVNVPVVGGHSGVTIVPLLSQATPSVSFPQPELEALTKRIQEAGTEVVQAKAGAGSATLSMAFAGARFVFSLISALQGKGVVECAFVKSTETEATYFSTPLLLGKNGLAKNLGLGKLSPYESELVKTALPELKNNIKKGEDFSKKEFEWLLQEEVNIVLEQLVIMECSKRFPV-VKSEKFFM-QIKVVVTLTGDNISHADITLRI-PKHNLRTIVQNDCQWKLQQVQDAGNNLLQALSLLRFEFKSAEEVTQLMTTIMGCLQRGRASLIIPKKRTIEEILGSRNMKSLQPPLPSDIAASFYVQSHKLVFAVYHI-HKDSPKF-DAEASVPWLSEVLVLFTIALQLCQQLKDKVGVFQFRDFIPTFIDILKGFGADISNVQQIIGVCDVCFKDIEAVL--NSIVS-ILAAFCDKLSKAPSNRMCVR--VLQNLYEALRFDVYYNLVKVASDMMAAVFSDLNKVKAWLPVARVQKLLRLLHEALTELASKLMVELLSTYTEDNASQARDDAHRCIVSCLADPNTFLLDHLLPLKPVRFLEGELIHDLLTIFVSEKLSAYLQFYQANKDFV-SSLGLSHEQNLHKMRLLTLMQMAETQRELDFDVLQRELQL--QDVEGFVVEALRTRMLTAKINQTGRKVVVATTVHRTFGRHQWQQLKDILRLNLVENNMDRFLETRG-TK-RKKPIVELEEGKNAIEEMEERAMIEEDPRLKELISVLIEWINDELASHRII--VKDIEEDLYDGQVLQKLLESLTGKRLDVVEVTQSEEGQKQKLRTVVDCANQILGYKWSVESIHNKNIVSIIHLLVALARHFRAPV--RLPENVVVNVVVVQKR-EELTSTYDDLGMRVE-RDAFDTLFDHAPDKLQVVKKSLMTFVNKHLNKINLEDLD-TQFQDGIYLVLLMGLVEGYFVPLYFFPTP----------------------SSKVHNVAFAFSLMTDAGLKPKARPEDLVNADLKSTLRVLYNLFTKLSHRSPGIVETAEADLRELMGIPSNYKVIFLQGGGTGQFSAVPLNLCPADYVVTGTWSAKAAKEAEAFIKHTCIPPQSEWKRSSDASYLYYCDNETIHGVEFPLVCDMSSNILTKPVDVSKYGLIFAGAQKNLGMAGVTVVIVREDLVGQAGCCPSVLSYKINAENKSLYNTPPTYAIYILLVMKWVKRNGGVEGMRRSAEKSRLIYSLFDASNNFYNRSRVNIPFRI-GGAEGDDELEKLFLKEATMIQLKGHRSVGGIRVSVFNAMSVEAIAVFYRDRPEWKDVTPVVRIAYSEQFQDIFDYFRAVLLNERSERALELVTDAVDINTSNYTVWHYRRALLKDLGKDLHEELTYIQKVIEDNPKNYQVWHHRRVLVEWLEKAFTESVLDAKNYHAWQHRQWAISEFDLWDGELDYVSTLLQDDVRNNSAWNQRFYIISNTTGFVLDREVAYTFECIRHNESPWNYLRGILVAYLLAFMVDRALEFDVIRKEYWNFVARGIAVIVDRFDELLLFAPHMHKFLKAQRIQHRFLIVNQVDRLRFNRGSLINVGFLVAQSDCDYLVMHDVDLLPLNRELSYAYPANGGPHLAAPDLHPRYHYPTFVGGILLMSNARFRQLNGLSNKYWGWGLEDDEFYARMRDARLNVSRP-GLKTGIRNTFRHVHDKQHRPRDTARLHNQRAETRKRDRVTGLADVKYDLAALHRLVIDGAPVEVLDVRLRCNLTVTPWCQK--VAPGDVITTDGFMRGHG-LLSSVAGVVEKTNKLVTVRPLKTRYNGEVGDVVVGRIVEVQ--QRRWKIEMASRLNASLLLSSVNLPGGELRRKSAEDELLMRQYLSEGDLISAEVQSVFADGSLSLHTRSLKYGKLGQGTLVLVSPSLVKRCKHFHNLP-CGVHLILGNNGYIWIAGTKFVREAVARVRNCVLALARHHVMLFDTSVVYAYDVS-VADLTKQKSQKVVGGWLLVCSGMAGSNLNDAFAFRLTKSGLSMVHWHPFAEFPPSGKQWEQEFLKYQEYPEYKEYNQAMTLSEFKRIWYIEYLHRMWGRTIGAVFYTGASWLWWRGWLSRRAKGHVAVLGVGLAFQGALGWFMVRSGLHDQPLVGHMQKGYAIGQAFLLFTRILQTSEFIILKQTFSPRMSRVLGLVFVTAMSGALVAGLQAGLVYNSFPKMADRWVPSDILALPKLRNFTENPTTVQFDHRILGESVVTGLWLWGRKQPLPPRARKALHSHSGANMRATLGVSTLLTYVPVSLASSHQAGAVTLLSVALWLAHELKLLRR-ICCR-----------------------TEKNASQWSKDKLTELLTNLEVKD-GRGSCVVEMSKCDGEAVANNRKAKLIFFYEWAIELTDDS-TVEGKVEIPNLSEEHDPTDVDITVTV--ERLKELMRTKGEKLIRDQLETYISSLKKEFSQGMILPTKTDSINQTKSTGKALDTTTLTHETFKCTAQELYRALTMVQAFSQGPCGGRFELYGGNVTGTFTDLELISMRWRFSSWPQGHFSAVLELVQKEDCTLVQEHVPKAEADRTRDGWQRHYWDSLKRTFGFGAILMDPEMFLDVANQVTKLKMYPYFDIAHCVITCLYLREDLGPGSQLFSRKHPLSCWITSMFSIFAGGMLSSFLLGEPVLGIFKNNQQLLLATAVWYAIFYSPFDLVYKICKFFPCKLIIALMKEVTRCKKVHDGVTHAAKIYPNGYLIMVVIGVVKGNGSAFLKICERLLRGYWTPNAMEIMQPSFPTKACIVAAVIFVLDKKTEFISAPHALVYLGIVVFLLYFKLSAMLLGIHDPFLPFENLFCAIFLGGIWDALGRALKSDATKKKDMLRKEGLVQRKNAESEDDSKETRLTLME----------EGYTSFWNDCISSGLRGCILVELALRGRIELERCGVRRRSLLLRKVLLRSDTPTGDVLLDEALRHLKETRPPETLQAWIDYLSGETWNPLKLRYQLRNVRERLAKNLVEKGVLTTEKQNFLLFDMTTHPLVDQASKGKLVRRVQEAVLGRWVNDPQRMDRRLLALLVLAHASDVLENAFAPLSDEDYELSMRRVRDLLDLDMEAEAAKPNACEVLWGVFAAFVKMQDCTPTFKCVLVGDGGTGKTTFVKRHLTGEFEKKYVATLGVEVHPLLFHTNRGPIRFNVWDTAGQEKFGGLRDGYYIQGQCAIVMFDVTSRVTYKNVPNWHRDLVRVCENIPIVLCGNKVDIKDRKVKAKSIVFHRKKNLQYYDISAKSNYNFEKPFLWLARKLIGDPNLEFVAMPALAPPEVTMDPEWQAKLENDMKEAQNTSLPDEDDDDLFADRLTPILKESKFKETGVITPEEFVLAGDHLVHHCPTWQWATG-EKSYLPPDKQFLVTRNVPCYKRCKHMEEKVLEEEDGGWVDTHDDDDDDEEAADMDDFVDADDPATGIVSTRTYDLNITYDNYYRTPRLWLYGYDENRQPLTIEEMYEDISQDHAKKTVTMEAHPHLPGPPMASVHPCRHAEVMKRIIQTVTEGGGLGVHMYLIVFLKFVQAVIPTIEYDYTQNFAMISKFEPNLPYDKLQQNLQVVRKRLNRPLTLSEKILYSHLDQPETQEIERGTSYLRLRPDRVAMQDATAQMAMLQFISSGLKRVAVPSTIHCDHLIEAQVGGAKDLARAKDLNKEVYNFLSTAGSKYGVGFWKPGSGIIHQIILENYAFPVLFFVSFTCSVQNKSGLGGLCIGVGGADAVDVMADIPWELKCPNVIGVNLTGKMSGWTSAKDVILKVAGILTVKGGTGAIVEYFGPGVESISCTGMGTICNMGAEIGATTSVFPYNSRMKDYLAATNRKDIAEAADQNKELLTPDSGCKYDQVIDINLSELEPHVNGPFTPDLAHPISQLGKNAKEAGWPMDIRVGLIGSCTNSSYEDMSRAAFLAKQALDHGVKAKCTFTVTPGSEQIRATIERDGQSKIFDEFGGMVLANACGPCIGQWDRKDIKKGDKNTIVTSYNRNFTSRNDGNPATHAFVTSPELVTALAIAGRLDFNPMTDELTGSKGEKFKLESPRGDELPQRGFDPGEDTYQGPPKDGTGVSVDVDVKSQRLQLLAPFDKWDGKDLEDMVILIKVKGKCTTDHISAAGPWLKYRGHLDNISNNMFIGAISEESGEANKVQHRQTGEWGGVPDTARKYKASSLRIVASGNEAFDEGPQTLHSRCERERRGPCA----------ETNLKKQGLLPLTFQNPADYDKIKSDDKLSLLGLKDLAPG------MFKNTFQSGFLSILYSIGSKPLQIWDKKVRNGHIKRITDNDIQSFVLEILGTNVSTTFITCPADPRKTLGIRLPYLIMIVKNMKKYFTFEVQILDDKNVRRRFRASNFQSTTRVKPFICTMPMRLDEGWNQIQFNLSDFTRRAYGTNYVETLRVQIHANCRIRRVYFADRLYSEDELPAEFKLYLPVQSLLT--KYHYPDQAKKDVSNALQHYRNLSSQFVFNDGTKKELFCLDGTIPVSYKYNIPVCVWLLDTHPYNSPMCYVKPTAYMQIKVSRHVDQTGRVFLPYLHEWNPNS-SDLLGLIQVMIIVFGETPPVFSK-PQPYPT-NTGTITQEHIRASLLTAVEDKVKGRLKEVAQAEMDVLKKTHDELNAGKTRLEDMINRMDREAELESERNEEMKEVDVDEAVVTTAPLYKQLVNAFAEENATEDAIYYLGEALRKGVIDLDVFLKHVRELSRKQFMLRALMQKCREKAALPSGRKFCVGGNWKMNGNKSSIKEICDMLKTAKPNTEVVLGCPAPYLDYVRRILPAAIAVSAQNCYKVEKGAFTGEISPAMIKDCGATWVILGHSERRNVFKESDELIGDKVHHALESGLNVIACIGELLEEREAGKTEEVVYRQTAAIAAKVTDWNRVVLAYEPVWAIGTGKTASPEQA-EVHAQLRQWLSKNVSPDVAKKVRIQYGGSVTAANCQELAKKPDVDGFLVGGASLKPEFVEIINARQ???????????????????????????????????????????????????????????????????????????????????????????????????????????????????????????????????????????????????????????????????????????????????????????????????????????????????????????????????????SKIDYLRQWSVSTYKCTRQMLSEKLGKGTRTVDTELEAQIELLRDTQAKYLHVLRLARALASHFQVVQTQGLLAEAFQELAHR---PLQEEFRYNAEAQRSLGRNGETLLGALNFFVSSLATLCHKTIDDTLLTVRLYESARLEYDAYRTEAQRNFKDKYEKLKADVTIKMKFLEENKVKVMHKQLLLFHNAVSAY-FSGNQASLEATLKQFNISWLEQ-MAELLLDPDIRIWVFLPIVVITFLIGIVRHYVSILISSTKKVELQQVQDSQALIRSRFLRENGKYIPKQSFLMRKNFFNHEETGYFK-TPKRAPVMQNPMTDPSMMTDMLKGNLTNVLPMIVIGGWINWTFSGFVTTKVPFPLTLRFKPMLQRGIELMSLDASWVSSASWYFLNVFGLRSIYALVLGENNAADQTRVMQDQMSGAALAMPPDPKQAFKNEWEALEICEHQ--------???????????????????????????????????????????????????????????????????????????????????????????????????????????????????????????????????????????????????????????????????????????????????????????????????????????????????????????????????????????????????????????????????????????????????????????????????????????????????????????????????????????????????????????????????????????????????????????????????????????????????????????????????????????????????????????????????????????????????????????????????????????????????????????????????????????????????????????????????????VKLTAEQLWKGVTSVSNAGRKRGRASGHSRKLSKDLNRGQVIGVGKVNMVWPGLNAPVVRGKEVVERVQLPPDKQREEKLLQLRNEMHGFRPLKLTPLERGWCGTRAPGRSIGPPDPVGDETFEKFDTIVVESRMVSRMTGNLGRTRRHKAVVVVGNKNGLIGFASGKAPEAKAALRMAKNRAIQRLRYIDRFEDTVYHNFFTQYGPMKMMVRKKPRGYGLVCHRIFKEVCKLAGITDIHVKMDCRAGSATVLLRAFILGLHNQKTHQQLADEKRLHLVEFKRETDNFPRVVASPKNCRTAEQIGTDEELDFDLVVSGGRLVKPPPKYVPWYIRTKGWQVHLRKVDPYRNAYNTRLQLTARYGKLCSFVNVSSPKDKDASRKSDEKTKWINKQRVMIFASRGITFRDRHLMLNLRTMLPHSKPESKME--KKDINEICEMKNCNKCIYFENKKRKDLYLWMSNVPNGPSVKFLVENVHTMEELKMTGNCLKGSRPLLSFDKAFNENPLLKELLCQVFGTPRHHPKSQPFVDHVFTFSLLDHRIWFRNYQIVEE-EGSLKEDSPRFVLNPIKVFEGSFGGPVIYSNPHYVTPSAYRRTLKQAAASKYKIDEIFETKLLFQRMAVGKNKGLSKGGKKGVKKKIVDPFTRKDWYDVKAPTMYTVRNIGKTFVNRTQGTKIASEGLKGRVFEVSQADLTNGEDAYRKFRLIAEEVQGRNVLTNFHGMDLTTDKLRSMVKKWQTLIEATVDVRTTDGYLLRMFCIGFTKKCANQLKKTCYAQHNQVRLIRKKMTEMMVVEVSSSNLKDVVNKLIPGSIGKDIEKSCQHIYPLHDVLIRKVKVLKKPKFELGKLLELHGEGKGAASTEGVKVDRPEGYEPPVLETVIKVGIIGGSGLD-DPDILEHTTPFGEPSDV-LVSGQIGHVDCVLLARHGRNHGIMPTNVNYRANIWALR-KMGCTHVLATTACGSLHEDFAPGHVFPDQFIDRQRATT--VCHLSMASPFLRKLLIQHEKGTVVTIEGPRFSSAAESIMFRSWGCHVINMTT---VVLAKEAGLLYAAIALPTDYDSWR-TDLAHVDVKQVETMKENSERALRILRAAIPRISRISLVMKVLRHEEFEEGCRAACNGPYDLNWSKTMVGYGQEDNHFVVELTYNYGIGSYERGNDFLGIVIRSGDIVERARRHSWPVQEKGDVVVVMAPGGYPFLVCPKRNPLRQDPVERVILSSSDLARTTAYWRDILAMTLVHSSEKSAVLAYANDQCHLEFRYTAEPIRRGTAYGRIAFACPAAELQVIENRVKRAGHRILTPLTRLDTPGKATVTVVILADPDGHEICFVDAESFMLLSAVDPNANQLLSRAIEKDKSDEWFRDMAGGKKPS????????????????????????????????????????????????????????????????????????????????????????????????????????????????????????????????????????????????????????????????????????????????????????????????????????????????????????????????????????????????????????????????????????????????????PLIPIVIEQTGRGERAYDIYSRLLKERIVCVMGPITDELSSLVVAQLLFLQSESNKKPVHMYINSPGGSVTAGLGIYDTMQYIMPPISTWCIGQACSAGSLLLCAGTAGMRHSLPHSRIMIHQPSGQASGQATDIQIHAEEILHLKRVINGMYAKHTKQPLETIESAMERDRFMSAEQAKEFGIIDVVLEHPPMRADKPIGTWLLMWPAWSISLASQAGCLPDLALFGAGAFLMRGAGCTINDMWDRNIDSKVERTKYRPLAADQLSMFDALVFTGGQGLSTLILLQLNWYSIVLGASSVILVTLYPAMKRITYWPQLTLGLTINWGALLGWAAVVLPLYAASLCWTLIYDTIYAHQAKMDIGYNKLMSTALKFGPRTKRFLSLFSTMVSNLQCWPYYAAVTLKLNQIGFIMGILSSSLVRIPGVVKPRTQRGKRFLQNRESKLVENTKTIFIRGSNANSNVIKAMKNMCSVKRPHSVFFNKKNPFEDQTPIEFMKKSDASHFVFGSHSKKRPNNLVFGRTFNGHLLDMFELGMDSFKSLEDFKGPKVPVGTKPMLVFAGEQFEELERLKNYFIDFFKGEAVSLQGLEHVIMFTAVEVLLRSYRVLMKKSGTKLPR-VELEEMGPHLDFSLRRTKIASDDLFKTARRQPKQNKVTKKKNVEKTALGSTLGRIHMERQDFQLQTRKLKGLKIKVLNALQDNYMYLVVTKEAAVVDP-----VLEEVKVKLTTVLTTHHHWDHSGGNDKLVVYGGDDRVPKITRDGE-QLQVGQLVKCLHTPCHTSGHICYFFVFTGDTMFIAGCGKFFEGTADQMYKALLAKLPDATRVFCGHEYTINNLKFAAKVEPGNQIVDKMAWAKNEPTVPSTIAEEKTFNPFMRVAVHKHADPISTMAYLRKEKDHFRMRRSRRVPRAKEPPPNLTDCIAGVDGRAESVDKKISRLDAELKKYKDQMVKMRDGPAKNMVKQKAMRVLKQRKMYEQQRDNLMQQSFNMEQANFATQTLKDTKTTMEAMRLGVKEMKREYKKVNIDDIENLQDDLEDMLDQAGEVQEALGRSYGMPEVDDDELEAELNALGDEIALDDTSYLDITSTRASSRAKHTLPDLPYDYGALEPVISGDLMRVHHQKHHAAYVNNLNAAEEKLADALAKNCVRSIVSCSMAIKFNGGGHLNHSIYWTNLSPNGGGEPTGDLLEAIKKDFGSFEALKAQMSARAVGVQGSGWAWLGYNPKTQRLQVATTGNQDSLWDTEGLVPLFTIDVWEHAYYLQYRNVRPDYVKAIWDIANWNNVAERFQKAHDVHIQDEFIKEALQSGMDLRQYSLQVEKDLKEVENASIQDSIEESVNIASLHNQIAACDEILERMENMLKTFQEDLGSISHEILTLQQQSVSMNVRLKNRQAVRGELSQFVDDIIVPEAMINNILEMPVTEKEFLEQLYVLDHKISFVKEQSFKDSRSCQDVRDILEKLKFKAIAKIREYLLQKVYSFRKPTTNYQVPQNAMLKHKFCYQFLMTHEREIAREIRDEYVDTMGKILFSYFKSYTNRLMKLQFEEVAEKDDLMGVEDTTKRNILSKPSLKNRSTVFTVGTRNNILTTELESPIIVPHAAQKNDARYPFESLFRSQQYALLDNACREYLFVTEFFMCSGSAAQDLFNLIMSKTLTMFLVRLTLQFAGCYNSLYLFVYIAIQYYCIMFLCSPTQSALTLYWESLLQFLYPRFEYILRLNIQSIRDCDPQKLGSIDMRPHYITRRYAEFSAAVVSINENFPSERVASLLAALQGEVENFILKMAAEFQGRKEQLIFLINNYDMMLSVLLERTKEDSKETESFKELLNARTQEYVEEILAPHFGGMISFVKECELLQERGQADVMQREEKKVTMLVRSFNTGWKKAIDDINHDIMQTFTNFKNGTNILQAALMQLIQYYHRFQKVLSQAPFKQLTVRTELLNIHHLMVEVKKYKPNF???????????????????????????????????????????????????????????????????????????????????????????????????????????????????????????????????????????????????????????????????????????????????????MRLTNVLRLPKDYANLPESYVKRSMAMVEWRTPNAPQYQRKVIQRTYNMSRPWTDGFKSNMPGVRIYVEPIFRGDRVEVLVGRDKGKQGIVNYIVKERNWVVVEGLNCREAPLLVTTQVALLDPTDNKPTKVEWRYTEEGKKVRVSVRTGRILPIPLAEETMDYKYFEQPKDTKAKDLEKITYVP-KLMTFAQDIMEEMGIKEDRIPAKTYWYSIHKIESLLWFTRMCTVVFTLLYIIPNPYTCYQRALISNAATSALRLHQRMPTIQLSRMFLSQLLIEDSCHYLFFSLIF-LPMT-LVLLPVFLFALLHSLTLLDKFGLLLE---HSNNILRLVAFTEIFLMPLTVFSLLSGSSLVTPFVYYRFLSLRYASRRSPYSRTVFHELRLVTEHYAAPGCPAFLSRIGLICQLSPFILAMILVYLNFPKLEPSEKQHIKLPRDIEDAKGLGRVLNRYTDRAVTSGFFVTYILLQSFAIPGSIFLSILSGFLFPFPMALFLVCLCSALGASFCYFFSYLVGRRLVLKYFPHLLNYMIFLRITPFLPNWFINIAAPVIDVPIAPFFLGTFVGVAPPSFVAIQAGTTLHQLSSSVSWWSVSVLAAFALLSLLPVVFKLKQKVMSSQEKLTRIAIVNTDKCKPKRCKQECKKSCPVVRLGKLCIEVTPNDKIAAISENLCIGCGICVKKCPFEAISIINLPSNLEKDTTHRYSQNSFKLHRLPTPRPGEVLGLVGTNGIGKSTALKILAGKLKPNLGRYGDPPDWTEILQYFRGSELQNYFTKILEDDLKAIIKPQYVDQIPKAVKGSVQQLMDKKDEMQNQEEVCRVLDLNNVRDRSVGDLSGGELQRFACAMVCIQKADIFMFDEPSSYLDVKQRLKCAEAIRALIHPQKYIIVVEHDLSVLDYLSDFICCLYGVPGCYGVVTMPFSVREGINIFLDGFVPTENLRFRDTSLVFKVAETAEEEVKRMCRYEYPNMRKCLGDFEMSIDAGTFTDSEIIVMLGENGTGKTTFIRMMAGLIMPALSGDVPSLNISYKPQKISPKSQGTVRMLLHEKIRDAYVHPQFIADVMKPLQIDAIIDQEVQNLSGGELQRVALALCLGKPADVYLIDEPSAYLDSEQRLVAAKVIKRFILHAKKTGFVVEHDFIMATYLSDRVIVFEGQPSVKTRANTPQTLLAGMNKFLELLNITFRRDPNNFRPRINKLNSVKDVDQKRSGNFFFLEDEEDGEELFGPALDTYDAVEDQLRQRDRGRMRRGLESIENLEDLKGHTVREWVTQGPKTEIYNRFKNFLRTYKEKIRQMEENKHSLEVNYTLAAAEQVLAYFLPEAPTEMLVILDEAAKDIVLGMFPQYERITGEIHVRITDLPLIEEIRSLRQLHLNQLVRTAGVVTSTTGVLPQLSLVKYDCGKCNYVLGPFVQSQELRPGSCPECQSLGPFTVNMEQTVYQNYQRISIQESPGKVTAGRLPRSKDAILLGDLCDSCKPGDEIELTGVYTNNYDGSLNTVNGFPVFATVVMANHVVKKDTDDDTRHILALAKDDIADRIIASIGPSIFGHENIKRAIALSLFGGEQKNPGQKHRVRGDINLLICGDPGTAKSQFLKYVQQVAPRAVFATGQGASAVGLTAYVSPVTKEWTLEAGALVLADKGVCLIDEFDKMNDADRTSIHEAMEQQSISISKAGIVTSLQARCAVIAAANPIGGRYDTFSENVRQSFFALSEAAAVFFLKDERLARFVVESHMRHHPIPQELLRKYILYAREKVHPKLDQDKIARMYSELRRESMATGSVPITVRHIESMIRLAEAHARLHLRQHVDVNMAIRVMLESFITQKYSVMRMGLLADCERIFGSTDIYKLLDVPKDASPTAIKKAYRRLSLLMHPDRVNAVQKQAATQKFQVLSKVYVLLSDSEKRAVYDETGCVDDDEDLSNNKDWDAYWRLVFPNITVADIDRFMLTYKGSPEEIEDLKKRYEEHEGDFDAISECLMGYEEDRYDLVMLLTIVLIALGKIGAKKRKKLEMKAEKRLQRERDLEDREEMKQRQAEEARKQLDEQKRKEEEAERAAEEERLKKEQEQRELEEYLRMKEAFSVEGEGFDAATQEQASPSFLHEFIEYIKTEKVVILEDLAAKFQLKTQDCIDRVQALLVDETLVGVIDDRGKFIYITRKELEDVAHFIRQRGRVSISELFENSNSLINLTPDVTALEAEAKATAIKHIITMLQLPEQLDKVQQYKRRVSRKKASVEAMLKTALQSQLDGFRTALTLQKSIPNDVLEVQRNDMQEIFDLPGLERLQEVKEESMRHSQYGAAIENLKHIFNVPGSVQKTQELIASGKLLHAHQLSDLENSRDDLLFELHKLPSQSVTDRNMLKQYFADVEKLSEDLGKQIWLVLKRTLNSVRKEPQVIVTALRIIEREERAARERERSSAGFLPPGRPKEWRRRCFEVLESAVEDRIEGNQFEERHENKMWLVRHLEVTRQIVLDDLRTVKTVCVPCFPPEYDIVERVKMYHNCLSRHLQNIVAQLEGNEYITVLGWLTVYSGEELMGHPDLALDVPLLGRDVQGLIQKYLGTLVANYQDWLKNALQDVKDWPDTDSRGCYHTSAPMIVFQMVDQHLQVAKTVDLVRKVLTISLEQTTKFVGAYMEAVTEFHFEDRSFTHYVIAVANNCLQFS-LDRLQERTLDALRDELFLDLGKLGDVLSDTVSATIEDYFRDYIQWQLARCYVQAIL--QRKITFKGYEERKVAAEKEQLRASPLDALPLLAEVLKMKDTSLLSLEVSGSRTDDEVVQREEEAIKLDGLNVAQMKELREKAEKHVFQAEVSRMMKLIINSLYRNKEIFLRELISNASDALDKIRLLSLTDPDVLNTNPELTIRIKSDKDNGLLHITDSGIGMTKADLVNNLGTIAKSGTAEFLQKVTESDAPKELNDLIGQFGVGFYSAFLVADRVVVTSKHNDD-KQHVWESDSGEFTVAEDPRGNTLGRGTTVTLQLKEEARDFLEQDTLKKLIEKYSQFINFNIFLWTSKTETVEEPIEEP--TEEKEADTDKEEEDKVEEEEDDKKPKTKKVDKTTWDWELINSAKPIWTRKPAEIEEKEYEEFYKAITKDTQPPLMKTHFIAEGELTFKALLYVPAVQPTESFNRYGGKVDHIKLYVRRVFITDDFQDMMPSYLSFVRGVVDSDDLPLNVSREMLQQHKLLKVIKKKLVRKALDMMKRIPKEDYQRFWKEYSTNLKLGIIEDTTNRSRLAKLVRFHSSH-G-EELTSLSDYVTRMKDGQQFIYYIAGASLDEVKRSPFVERLIRKGYEVLLLTEPVDEYSISSLTEFEGKKFQNVAKEGLKVDE-GKARERHDALVKEFEPLTKWLEDDVFKGRILKAMVSERLATSPCALVANQFGWTGNMERLARSNAHAKSQDTMRDYYLSQKKNMELNPRHPLIKELLRRVKDDAKDSEARNMAELVYETATLRSGFMLEDTLAFATRVESLLRKNVGLPDDAAVEEEPMVKGE--RLSKEEVQMRVISEIIKELLVAQKEKRDVNLNSIKGSISSKYGLHSQPKIVDIIAAIPAQYKEVLLPKLKAKPVRTASGIAVVAVMCKPHRCPHINMTGNICVYCPGGPDSDFEYSTQSYTGYEPTSMRAIRARYDPFLQTRHRIQQLRQLGHSVDKVEFIVMGGTFMSLPEDYRDWFVRSLHDALSGHTSGSVGEAVLYGERGRTKCVGITIETRPDYCLKRHLGDMLLYGCTRLEIGVQSVHEDVARDTNRGHTVRAVCESFRLAKDAGFKVVAHMMPNLPNVDFERDIDQFVELFENPAFRMDGLKVYPTLVIRGTGLYELWKTGRYRSYPPGFLVDLIAKILALVPPWTRIYRVQRDIPMPLVTSGVEHGNLRELALARMRDFGTRCRDVRTREVGIQEIHHRVRPNQVELIRRDYVANGGWETFLSYEDPEQDILVGLLRLRRCSEETFRPELKGGASLVREHVYGSVVPVSVRDPTKFQHQGFGTLLMEEAERIALTEHGSSKIAVISGVGTRNYYRKLGYELDGPYMSK

'Galendromus_occidentalis' PGARVNVLSKELMLSVVLISGKKGCFIAGADITMLESCKSEAKKLKPVVAAIMGSCLGGGLETALACHYR-IAMNDSKTALGLPEVMLGVLPGGGGTQRLPKLVQLPTALDMMLTGKSLKANKAKKAGFVDVVIEPLGPGVYLEEVAARDLASQKLKVRTRPLSEKVVRDY-IFDKAKGQVMKLTNGLYPAPLRIIQVIRAGLEKQNFAELCTTESRGLMGLYHGQVQCKKTVIPCLSRNVAVLGAGLMGAGICEVSLKNFDKVIMKDGLVRGQNQIKRKRLEKDKLMSGLLPTLDYSDMVIEAVFEDLAVKHKVVKEVEAEHCVFASNTSALPITKIAEASKRPEKVVGMHYFSPVDKMQLLEVITTDKTSKDTAAAAVDVGLRQGKVVIVVKDGPGFYTTRILAPMMSEAMILLMEGCPVRDLDKLKDFGFPVGGATLLDEVGIDVGAHIAKTVFGEREEMVSNFLGRKSGKGCYIYRAVNPMQAKYTTEQMQYRLATRFINEAIMCLQEGILANPVEGDIGAVFGLGFPPNRGGPFHFVDTYGADKIVNMQQFQEFEPCQLLLDHA-KDPSKKFHMQRPIILLKDGTESHQGKPQVISNINACQVISDAVRTTLGPRGMDKLMVDSKGKTIISNDGATIMKQLDIVHPAARTLVDIAKSQDSEVGDGTTSVVLLAGEFLKQAKPYVEEHVHPQVIARSYRKASCMAIDKIREIAVTVDKGETRALLEKCAMTTLSSKLVASKKEFFAKMVVDAVMQLDELLPLNMIGIKKVSGGALEDSVLVSGVAFKKTFSYAGFEMQPKKYHNPKIALLNIELELKAERDNAEVRVDSVEEYQNIVDAEWNILYDKLAKIHASGAKVVLSKLPIGDVATQYFADRDMFCAGRVAEEDLRRTMKACGGCVLTTVQDLKDSNLGSCEKFEENQIGGERYNIFTGCPNSKTVTMILRGGAEQFIEETERSLHDAIMIVRRAVKNDAVVAGGGAIEMELSKYLRNYSRTVAGKEQLLIAAFAKALEVIPRQLCDNAGFDATNILNRLRERHAKGDKWSGVDINNEDIADNLEACVWEPAVVKINALTAATEAACLILSVDETIKAPQSNTDPTAGRPFAPTLNRVEAFREHGMAVTRDYISQPRMIYKTVCGVNGPLVILDQVKFPKYAEIVQLVLADGTPRTGQVLEVSGDRAVVQVFEGTSGIDAKHTVCEFTGDILRIPVSEDMLGRVFNGSGKPIDKGPAVLAEDFLDIQGQPINPWSRIYPEEMIQTGISAIDVMNSIARGQKIPIFSASGLPHNDIAAQICRQGGLVKRPGKSVMDDNFAIVFAAMGVNMETARFFKQDFEENGSMDNVCLFLNLANDPTIERIITPRLALTTAEFLAYQCEKHVLVILTDMSSYAEALREVSAAREEVPGRRGFPGYMYTDLATIYERAGRVEGRNGSITQIPILTMPNDDITHPIPDLTGYITEGQICVDRQLNNRLVYPPINVLPSLSRLMKSAIGEGFTRKDHADVSNQLYACYAIGKDVQAMKAVVGEEALNAEDMLYLEFLAKFEKNFISQGRYENRTIFESLDIGWNLLRIFPKEMLKRIQHKILAEYYPRGESKTGLFESPHGFIILRDKAITEAENLIAEAVSRKRKMVQIFDDLSDCLCRVADLAEFVRTGHPDARFSSAAREASVAISSLVEQLNTNVALYSALRSVVEGE-DIVPTNIEKHVGRLFLFDFEQCGIHLDERTRKDVVALNDHILYTGSYFLQNSQKPRVVSNKVLPQNLFAVTGLNADSPQEAIRETTYKIFLLPDDEQMSLLDNLLESRQELAQLCGFKTHAHRVLKGSIADTPENVSFLSYLSKELRPRAEKDYQEMLRMKAWDIPFFSAYLSLSTCMEGLDMIFQSLYGVTIRVAGELWHPSVIKMIVKDSDDPIGIIYCDLFERAQKPHQDCHFTIQGGRKADGSYQIPKVVLMLNLPPPLLTPASMDNLFHEMGHAMHSMLARTEYQHVTGTRCATDLAEVPSILMEYFASDPRVVTRFARHYKTGEPMPAILASNLEASRVIFQASETQLQVFYAFVDHAYHSQYPRSTTEILREVQNEHYGVPFVENTAWQLRFGHLVGYGAKYYSYLMSRAVAASFWQKAFKADPLSREAGLYREKVLAHGGSLPPAELVRGKLLAEALIRDIMPSDAKKKRDAKKKEALKNRNMPDPTNGEDEMDEVTKKFEEDMRMNAAARAVTGVLSIHPRSRDVKIENLSITFHGWEVLQDTKLELNCGRRYGLIGMNGCGKSALLSAIGRRELPVQDCLDIYHLTRECPPSDKTALQMVLDVDKERLRLEKLAEELASAEDDTSQEQLMEIYERLDDMNAETALAKASYILHGLGFTLAMQHKKCRDFSGGWRMRIALARALYVKPHILLLDEPTNHLDLDACVWLEEELKTYNRILILISHSQDFLNGVCTNIIHMNQRKLEYYGGNYDQFVITRAEQLENQMKRYNWEQAQIADMKDYIARFGHGSAKLARQAQSKEKTLAKMVAGGLTEKVVQDKSVSFYFPSCGPIPPPVIMVQGVSFRYSDNTPLIYKNLEFGMDLDTRVALVGPNGAGKSTLLKLLCGALVPTDGIIRTHSHLKIARYHQHLHESLDVDLSALEYMMKSFPDVREKEEMRKIIGRYGLTGRQQICPIRQLSDGQKCRVVFAWLAWQVPHMLFLDEPTNHLDMETIDALADAINHFEGGMVLVSHDFRLISQVAKEIWICEHQTVTKWPGDIKTYKQHLKNKVMKELEKMVLADLGRRITGALRNLSTATVINQEVLDSMLKEICAALLESDINVRLVKQLRDNVRAAIDIEEMAVGLNRRKVVQSAVFKELVKLVDPGVKAWTPQKGRSNTIMFVGLQGSGKTTTCTKLAYHYMKKGWKTALVCADTFRAGAFDQLKQNATKARIPFYGSYTEVDPVVIAAEGVAKFKAEHFEIIIVDTSGRHKQEDSLFEEMLEVSNAVSPDNVIFVMDASIGQACELQARAFKEKVDVASVIITKLDGHAKGGGALSAVAATKSPVIFIGTGEHIDDFEPFRVKPFIQKLLGLGDIEGLIDKVNELKLDENHELIEKLKHGEFTLRDMYEQFQNIMKMGPFNQLMGMIPGFSADFMTKGNEQESMSRLKRLMTIMDSMTDDELDNREGARMFAKQQTRITRVARGAGCTTFEVHELLNQYTKFAGMVKKMGGMKGLFKGNDLAKNVNPAHMNKLSAEMAKMIDPRVLQQMGGFSGIQNMMRQMNGGILTEDPAFLKLRSL-SPITKPLRLKDLFDKDPSRAEKYTLKLQSGPETLLVDYSKNLINDEIMAGLLELAKNRGVEAMRDRMFKGEKINFTEDRAVLHVALRNRSNRPIEVDGKDVMPGVNAVLEHMRSFCHQVISGEWKGYTGKKITDVVNIGIGGSDLGPLMVTEALRPYQIGPNVHFISNVDGTHLFETLKRVDPETTLFIIASKTFTTQETITNAESAKIWFLGKAGDKSHVAKHFVALSTNAAKVTAFGIDKENMFEFWDWVGGRYSLWSAIGLSIALFIGMANFEKLLTGAHFMDEHFRTTPLEKNIPVILALIGSWYINFLGADTHCLLPYDQYLHRFAAYFQQGDMESNGKYVQRDGRIVEHETGPIVWGEPGTNGQHAFYQLIHQGTKLIPCDFIAPAKTHNPIQGGIHHKILLANFLAQTEALMKGKSSEEAKAELEASGLKGEALEKILPHKVFRGNRPTNSIMVQEVTPFTLGALIAMYEHKIFVQGIIWDINSYDQWGVELGKQLAKVIQPELEGKEPVSSHDQSTNQLINFIKSFNNNASYQDKDKPAQVRQSNIAAAKAVCDAVRTSLGPRGMDKMIQAVSGDVTITNDGATILQQMQVLHPAAKMLVELSKAQDVEAGDGTTSVVVIAGALLDAASKLLHRGIHPTIISEGFQAAAKECVDILSSLAIPVELSDRESLLKSATTSLCSKVVSQHSDLLAPIAVDAVLRVIDPTNVDLRDIKIIKKLGGTVEDSELIDGLVFTERLAGGNAPHRMEKAKIGLIQFCISPPKPNMDHQVIVSDYAAMDRVLREERAYLLNIVKQVKKTGCNVLLIQKSILRDAVSDLALHFLAKMKIMVVRDIEREDIEFVCKSIGCRPIASLDHFVPEALGTAELVEEVTSSKFVKVTGIANP---RTVSLLLRGSNKLVLEEAGRSIHDALCVVRCLVKKRALVAGGGAPEIEMSLKLAERARQLEGLHSYCYRAFADALEIIPYTLAENAGLNPIQTVTELRNKHAQGEKTFGINVRKGCVTNILDENVLQPLLVSTSAISLAAECVRSILKIDDIVQTVRMSKRDVVYLWDPDVGNFHYGPGHPMKPQRIAVTHSLVLNYGLHRKMRIYRPYRANPHDMCKFHSEEYVNFLQRITPQNMESFDKKETLFNVGDDCPVFSGLYDFCSLYTGASIDGAWRLNNRTCDIAINWSGGLHHAKKFEASGFCYINDIVVAILELLKTYARVLYIDIDVHHGDGVQEAFYLTDRVMTVSLHKYGAYFFPGTGDMYEVGAESGKYYALNVPLKEGIDDASYFQVFKTVISSVIDHYRPGAVVLQCGADSLAGDRLGCFNLSIKGHGDCVKFVRDLNIPLLVLGGGGYTLRNVARAWTNETAILVNEQVSPEIPYNEYLEFFAPDFSLFPD-------ENANSKQYLEAILKYTTENLRCLEHAPSVQMQDVPPDIVDLADDEPLIENSKEHPAEFYD--------MSD--DNMSDNEEDYDLEYSEDDDSQPDVDLENQYYNSKALKQDDPKAALQSFQKVLDLEGGQKGDYGFKALKQMVKINFRLGQYEEMMSRYKQLLSYIRTAVTRNYSEKSINSILDYISTSKRMQLLQEFYEVTLDALKDAKNDRLWFKTNTKLGKLYLDREEYPKLQRILKQLHLSCQNVDGSDDLRKGTQLLEIYALEIQMYTARKNNKELKKLYEASLQIKSAIAHPLIMGVIRECGGKMHLREGEYNFAHTDFFEAFKNYDESGSPRRTTCLKYLVLASMLMQKEINVLDSQEAKPYKDDPEIVALTDLVDAYQAHNISRFEAIVSPHKESIMKDAFIKEHIEQLLLNIRRQVLIRLIRPYTRIKIDFISCELNIPSQDVESLLVSCILDNTIQGRIDQVRQVLELSS-SSVQKRYSGMEQWALQIQNLQNTMTNPAAHLGNLNPIQILNHQAEEEKAENARLSSFVGAIAIGDLLKSTLGPKGMDKILLCETSRDSKVEVTNDGATILKAIGIDNPAAKVLVDISKTQDDEVGDGTTSVAVLASQLLQEAEKLVGMKIHPQTIISGWRRSVNASRAALEDFSQDRSNNEAQFKKDVMNIARTTLGSKILAQHKEFFAQLAVDAVMRLKGKCNLDAIHIIKKLGGSMLDSYLEAGFLLDKKPGLNQPKRVEKAQILIANTPMDSDKIKVFGSRMKCESIARVAELEEAEKLKMKRKVDSILAHKCNVFINRQLIYNYPEQLFADAGIMAIEHADFEGIERLALVTGGEIVSTFTSPETVKLGTCDLIEEVMIGEDKLLRFSGVPLGEACTIVLRGATQQILDEAERSLHDALCVLTSVVKEKKICYGGGSAEMLMAEAVDKLALTTPGKESLAIEAFARALRQLPTIIADNAGLDSAQLVSELRAAHATGKKSFGINIVDAKIDDMEKLGITEAFVVKRQVLLSASEAAEMILRVDNIIKAAPRKRVPDRSH---------KPQFLCKLDGHQETVNQAILIGAEDAVISISDDKTIRIWSRRDSGQYWPSICHTMPAAASAMDYEHGTRRLFVALDNGTITEFDLSDDLNKITLRRSYIAHQQRVASIKFSLATEWLLSAGKDKYFQWHSTETGRRLGAFQSSSWCTTVEFDAASRHAFVGDYSGNITMLKLTETNYQPVTTLRGHSGSIQTLLWDEKKSLLISGSFDQVIIVWDIGGGKGTAYELCGHKQRVTGLAIFG-QKLLSVSEDSTLVVWDMSAKRQGTPEWAERDCCERCARPFFWNVKAMLDQKQVGLRQHHCRRCGRALCDKCSSNRSPLPVLGYEFPVRICDECHLHVSDRDRQSLAEFHDLKTPVAGMSLAPNGKLMATVGSDRNMKIWDIATVISKKALKKAQKDAEKAARKAQHKAEREAQNPNKDDDVSKGKYGSYGMIQSSERLERTLTPISSCTSKLVGQEVWVRGRIHVSRSKGKQCFLILRQACCSIQCVLAVGENVSREMLKFVSNIPKESIIDVCGKVMATPSKIDSCSQQDVELSTLQTWLVSASEARLPLQVDDAARPDVVKNEEDALNIRVNQDTRLDNRILDLRTPANQAIFRLEAGVCHLFRESLSKKGFVEIHTPKIISAASEGGANVFEVSYFKGSAYLAQSPQLYKQMAIAADFGKVYTIGAVFRAEDSNTHRHLTEFVGLDLEMAFEYHYHEVVDTIGHMFIDIFKGLERNYGAEIEAIRRQYPSEPFQFLEPSLVLKYSEGVEMLRSAGIEMEDDEDLSTPNEKLLGRLVKAKYNTDFYILDKFPLAVRPFYTMPDPENTKWSNSYDMFMRGEEIISGAQRIHDPEFLMQRAKQHGIDVSQIKSYIDSFRYGAPPHAGGGIGLERVVMLYLGLDNIRKTSMFPRDPKRLTPAMEEGLDLAEIEPLEFSRVKIIFPKECRIRRVTYRGRLELRFWWLNGLKQEPITRSCGEIPIMVKSRKCNLHGMSPDQLIERGEELEEFGGYFVVNGNEKVLRLLIMQRRNYPIAMARNGWKGRGSMFSEFGVSLRSCRKDSQNMVLHYLTNGTVQVMITNRKELYFVPIVLLLKALVDKSDYEIYKSLIKGFEDDSFYKGCITNMLRLVQENILTQDAKEFIGDKFRWATNSDVCDKLLDRCVCVHLDDNESKFNLMCFMVRKLFAAAKNKCALESADSTMNQEILLPGHLYLNVLKEKIEGLLLGVKLSTEKKMSNALDLTHAMNYFLSTGNLISKSGLSLQQVSGFTIIAEKLNFWRYIGHFRCVHRGSFFMQMRTTSVRKLLPEAWGFLCPVHTPDGGPCGLLLHLSAMCEIVVQLDGRCLGWILRYLKASG-IAPTLEICVIPRTEQNSLYPGIFLFSTPARMMRPVLNRTQAVEWIGTLEQVHMDICVVPEEATTHQELSETAMLSILANQIPWPDFNQSPRNMYQCQMGKQTMGSPMHSYRNRADNKLYRLNPQSALVRPTAYDHYKMDEYPSGTNAIVAVISYTGYDMEDAMVLNKMSVERGFKAGVYKTETVNLRVDYDGLPYIGSHGDPVCCYIQLKVVKYHSSEPATIVEVKILGNLQQVQFTYLIRTPMIGDKFASRAGQKGVCSTLWPTENMPFTESGMVPDIIFNPHGFPSRMTIGMMVESMAGKSAALHGYVHDASPFKFSEDDPSSEYFGELLQKAGFNHHGTERIYSGVDGREMDADIFFGVVYYQRLRHMVADKYQVRTTGPIDSLTRQPVKGRKRGGGIRFGEMERDSLLAHGTAFLLQDRLFNCSDKTLCAACGSVLSIPIPYVFRYLVAE-LASVNMKITRETSRDLHRHQRNEPELHPLATAREYTAAVNAAKIDRIFAKPFLCSLGHRDAVEIVAKHPEKISGVVSASVDGEVRWWDLTNRRCVGNVQAHDGPVRGLCGQDQSIKTILSKLAHHYSQMFATAGETVSLWEERNEPLRSLW-GVDTIYVTFSPIESLSSDRSIVLYDIREATPLRKVTLEMRSNALCFNPMQAMHFTVANENFNLYTFDIRHLKTALQAHTDHVGAVLSLDYSPTGTEFVSGSYDKSVRIFRSREVYHTKRMQKVTSVVYSMDAKYVISASDEMNLRLWKAKASEQLGITNYNETLLSRYGSHPTVKRIVKHRHIPKQLYKEKQIMLSSRKTKMGMQLLHRGAYQDALSHYHAAIEGDDSNYQSYYWRATVYLALGKSKLAVEDLNKVIDLKEDFIKAREQRGNILLKQGYLDEAHIDFEYVLRLDPHNLEAVIEQLKNDVISILQGVWNLRLRELRAECYEAIGDLNSAISDLRPAIRSVPDNTKGYLKLARLFHKHGEPEEGLTTIRECLKLDPDHKECYKSIKKLVKSMQDDCLEKIVTLVKTKCHCASKGGDAVNLCSEALRYDPILCDRAEAYLNADDFAQDFASARELDQRAAEGLKRAQKLEKAKGKRDYYKILASKGEITKAYRKLAAKWHPDQY-QGDD--KKGAEKMFIDIAAAKEVLTDPEKRAKFDRGEDPLDPDSGFHYTFHFMLVLFESPAGFAVFKVLDEKKIQKSENLFKEFEDASDASKILKLKHFQKFEDMTQALSAATGAIEGKLTKPLKKILKKL---EATETLAVADAKLGNIIKEKMDISCVANSSIQELMRCIRSQQEALITGLSQKEVTAMALGLAHSLSRYKLKFSPDKVDTMIIQAVSLLDDLDKELNNYVMRCKEWYGWHFPEMGKVVTDNMLYVKTVCKMGMRSNAITLDLSDILPEDQEAKVKELAEVSMGTEIAPDDVDNIKHLCEEVIQMTEYRATLHEYLKNRMAAVAPNLTILVGELVGARLIAHAGSLLNLSKQPASTVQILGAEKALFRALKTKHDTPKYGLIYHAQMVGQSSQKCKGKASRWLAAKAALAIRVDALGEDNDTDMSLKNRANLEARLKMLEEGKLTRISRYIWPKDKPNIKRTVALALGLLVAAKLVNISVPFMFKHLIDFLNENTGLVVGYGLARAGAAGLNELRSAVFASAAQHSIRSMGRKLFSHLHDLDLGFHVGRQTGALSKAMDRGTRGINFVLSALVFNVAPTLLEVTLVSTILWYKCGQFAAVTLGCIGTLLVTQWRTQFRIDMNKADNKAGSRAVDSLINYETVKYFNNEKYELKDYEKASLKTTTSLAALNFGQNAIFSTVIMYLASEMTVGDLVMVNGLLFQLSLPLNFLGSVYREVRQAIIDMQTMFALTEIESSKLSISKEIEFENVTFQPILRNVSFKIPTGKKVALVGGSGSGKSTTVRLLYRFFDPIDGRILINGQDIKDVSLRRGIAVVPQDAVLFHNTIRFNLQYGDLQAEVEESARMAEIHDAITGWYETQVGERGLKLSGGEKQRVAIARAILKNSPILVFDEATSSLDSITEQKIMKALRAATGKTTLCIAHRLSTIADADEIFVLKDGTLIESGHQSLLRSFYAYLWNQQHL--------------------------------------------------LPHMAFKGTEKRSQTDLELEVENAGMHLNAYTSREQTVYYAKCLTKDVAKAVDIIADITQNPKLGEQEIERERSVILREMEEVEGNLQEVVFDHLHSVAYQGTPLGMTILGPTENIKSLKKQDLQTYIKEHYTGSRLVIAGAGGIDHDELVKLAEQNFGKVSNSMDQK--MPCRYTGSDMRVRDDDMPFMHAAIAVEGAGWKNPDNIPLMIGNTMIGSWDRSHGGGNNATSRLAAAYAADQVVHSFQSFNTCYNDTGLWGIYFVATNGVVQRAVLQIQEQWMRLVTGATEADVTRAKNLLKTNLLLQLDGTTSICEDIGRQMLCYGRRIPLHELEARIDAVDAATLRKVCEEYLYDKCPVVAAVGPVEGLPDYTILRGHMWKHSVHTMVFRSLKRSHDMFLCDEGALPPIDEKAHKLRMDVKARDEYGRVMHLVNQNRRVPAPQALVLAPSQARAPLKKPSQPKPQWHPQWKLCRVISGHNGWVRCVAFDPSNEWFCTGSNDRIIKIWDLASGKLKLSLTGHIAGVRGLAVSQHHPYLFSCGEDKQVKCWDLEQNKVIRHYHGHLSGVYTIGLHPTIDVIVTGGRDSTARVWDMRSKSCIHTLTGHTNTVASVLVQATEPQIISGSHDSTIRLWDIVAGKTRVTLTYHKKSVRALALHPKLNMFASGAPDNIKQWMCPDGKFIQNLTGHNTIVNCLAVNHSNVLVSGGDNGSMQFWDWKSGYNFQKLTTPVQPGSIDSEAGIFQMAFDVSGSRLVTCEADKTIKIFKEDETATEEG------P--VTGSTSEYFINCSSEHYLEILGINVKAKNFLVFQGAVESIAMKNPKERTVLFEEISRSLLDSQEETYQKKKGIAAERKEAQMEEAEKYQKLKEDVQVNLHLFRLFHQEEDLEKRKKKERIENELREKKKVEQTFRDDVELNKRKPAYIRAKEKTAHTQKKLDAAKKSLDAATKTHRSHQGIEELEQELSQVEEAFDQEDVSLEESQVKEYNRLKEKAGKMASAALQEYDSVARDQKTDQDHLDNELRKRNECEAKLKELEENQRRVNKLVDLHDLKEEERKLEAKKRLSQKFEDVSSLGDAKVDKHEDARRKRKSEIVEHFKKLYPGVHDRLVNLCHPIHKKYNVALTKVLGRNMEAIVVDTEKTGRACIQYLKEQMLEAETFLPLDYIDFKPLKERLREFKDVPNVKLLYDVLKYEPLSIKKAVLYATNNALVCETAEDAAKVAFQAPDGKRYDAVALDGTYYQKNGFISGGSSDLAKRAKRWDDKDFHKLKDQKEKLQEDLREAMKTARKESDLTTIESQIKGLETRIKYSKIKQLEQSMREREVRINEIKAKQNTVEDDVFRDFCAQIGVANIREYEERELHASQEREQRADLENQKNRVASHLEYER--TKDTLAVELDQQELENLKEIEQKQKELIEQQVNLISQLKNERAVKKSKVDEIDEEVAEIRKRLTTQQKEVTAVQKGVTQAETKLEQKRSERHTFLQSCKLEGIRIPLILGSMQQMYEREALKIDYSQLRMEKEINLQRIQAPNFKAMEKLDSVKERLKDTDTEFEHARRKAKSNFELVKRERTCFEHVSNCIDDIYKSLTNNPSAQAFLGPENPEEPYLEGINYNCVAPGKRFQPMSNLSGGEKTVAALALLFAIHSYQPAPFFVLDEIDAALDNTNIGKVARFIREKTQTSFQCIVISLKEEFYGHADCLVGICPDPGECTISRIYTIDLSLTEAMLSFLRDAINKGDLNSMQDLYENSLTKLMDQYFLIIYKE--LYYRQIYADDRFNSYYNYVDFFNYILSSEPVTLELPNQWLWEIMDDFVYQF--QSYCVYMWNVHSVLNVLHSLVQKSNINQQLEVYSKGGNPEEVAGEFGRRPLYKMLGYFALIALLRLHSQLGDYYQAIKVLQHLELNRKGLSRVPSCQVSTYYYVGFAYMMMRRYEDAIRTFVNVLVYIMRTKRSYQLKQINKQMDKMYALLSICMALHPQRIDESVLQLLKSKQLQAMQSGDLDTFESCPRFLSPVQWNAFRGEIEAQLTIRSFLKLYTTMPVEKLAKFLLMSFKHLMSNMVLDGEFQTGSDMDFFIDKDMIHIADTKIARRYGEYFIRQYHKVEEAFEMGPPDAILGVTEAFKKDTNPKKMNLGVGAYRDDEGKPFVLPSVRQAEQKLAEQKHDKEYLPIGGLPAFCENAAKLALGKDSFVIKTGRNATVQGISGTGALRIGAAFLEKHLKGNKTVYMPNPTWGNHIPLFKHCNFEVKQYRYYQPKTCGLDLQGALEDISKIPEGSVILLHACAHNPTGVDPSASEWLEIEKVVRKRNLFPFLDMAYQGFATGDIDRDASAVRIFSQSG-PMCLAQSFAKNMGLYGERVGAFSLLCDSKEEAERCMSQIKILIRPMYSNPPVHGARIANLILSDPDLYAQWLADVKLMAGRIISMRERLQQGLKNEGSTRNWQHIVNQIGMFCFTGMKPHQVEKLTKDYSVYLTKDGRISVAGISSNNVDYLAHAIHNCTKRILRPVRHLAHCVLMAIVIYMFVESSTPASDVDPFAEYGVVLTILLYLFRLLPLLALPQSLTNFFGLTLYNAFPPKVKLKVDPLEAPFLCIRVVTRGGYPDLVKANVQRNLQTCLDLGMVNFVIEVATDKEVY---HPKIKQTVIPNDYQTSSGAMFKARALQYCLEDDVNVLDDDDFVLHLDEETIVTRDAMKGVLNFISQRRHPFGQGMITYANERIVNWVTTLADTYRVADDLGKLRFQFKFFHKPLFSWKGSYVCCRLGAERAVSFDNGPDGSVAEDCFFSMVAFSKGYSFEFIEGALWEKSPFTISDFLQQRKRWLQGIFLVVHSPRIPAKFKVWLACSLYAWATMPLSTSNLFLAPRYPLPCPQTFNILCAMIGALNLYMYFFGVLKSFSIKRQGLLKFLLCLGLVVVAIPINIFVENIAVVWGLLGDKHKFYPWPHYNFSGRLRPGRVGPMRDVPAHILRPDYADHPEGIAVSEQAMKGA-EIKVLSAAEIEGVRVASKLARECLDTALRAAKPGVTTDELDRLVHEAAIERNCYPSPLNYYLFPKSCCTSVNEVICHGIPDDRVLEDGDILNVDVTVYHKGFHGDLNETVFIGNVDDAAKNLVRVTYESLQKAIESCRPGVLYRDIGNVIQKHVQPHGYSVVKSYCGHGIHSLFHTAPSVPHYARNKAVGVMKPGHIFTIEPMISEGSYKDEVWPDNWTAVTIDGKRSAQFEQTLLITETGVEILTRRREKNGQPWFMDQSSIRRTLKAKSAKRRELLAQQLGAGCAENLGLLLGNEQDSASENKDQQNEDEVMAYRDSSTFLKGTQSANPHNDYCQHFVDTGQRPQNFIRDVGIQDRFEEYPKLKELIRLKDELIRETAKPPMYLKCDLMQYNLRDLNSKFDVVLIEPPLEEYQRSCGVTSSRFWAWEEIMKLEIEEIAAPRSFVFLWCGSSDGLDLGRQCLRKWGFRRCEDICWIKTNIDDSKVKNVEPRAVFQRTKEHCLMGIKGTVRRSTDGDFIHANVDIDLIISEEPQFGKMEKPEEIFHIIEHFCLGRRRLHLFGKDKAIRPGWLTVGPELMNSNFSSEAYNSYFNSPNEYLTGCTERIEALRPKSPPPKGKRVIIRVDFNVPIKEGKITNNQRIVAALPTIKHCLEKGAKAVILMSHLGRPDGNVNAKYSLAPVAEELKKLLSKEVTFLSDCVGPAVEKACENPAVGSVILLENLRFHIEEEGKGVDAAGNKQKADAEAVKNFRASLTRLGDIFVNDAFGTAHRAHSSMVGITLPERAAGFLMKKELDYFAKALDNPARPFLAILGGAKVKDKIQLIENLLDKVNEMIIGGGMAYTFLKINKGMKIGTSLFDEDGAAIVEKLLAKAKQNNVTIHLPVDFVTADKFDENATVGSATVETGIPDGWMGLDCGPKSIEQFKAAVGRAKTIVWNGPAGVFEFEKFAAGSKGIMDAVVEATKTGAVTIIGGGDTATCAAKWNTEDKVSHVSTGGGASLELLEGKDLPGVVALSEAMRTFGDRPIAFQLEDGGDYYYIGTEVGNYMRLFRGTLYKKYPSLWRRPVTVEERKKISQMNMSHHSAANFISLLKKVEVDDLIDGNEEKYRAAPVQESDGGAHGAKAARPSFMPAAPNNAHHLDAVPCSTPINRNRLQHKKNKSFPMLYDDLDPATLHENAAMPECLVPIRLDMEIEGNKLRDTFTWNKNEAQISPEQFAEILCDDLDLPPLLFVPQIAASMRQQIEAFPTESLLDEQTDQRVIIKLNIHVGNISLVDQFEWDMSEKLNSPEEFATKLCSDLGLGGEFVTAIAYSIRGQLAWHQRTYAFSEAPLSQLEMPFRPQSEAEQWCPFLETLTDQEMEKKIRDQDRNTRRMRRLANTGWMGANGSLLDREDAIPSTERYLGLVNFGNTCYCNSVLQALYYCKPFREKVLEYKAKNKRTRETLLTCLADLFHNIHSHKKKTGTLAPKKFIARLRKDNEVFDNYLQQDAHEFLNYLLNTIGDLLQAESIWVHEIFQGTLVNETRCLTCETISSKDEDFLDLSVDISPNTSISHCLRGFSSTETLRGEHKYHCEQCNSKQEAQKSLKVKKLPPILALHLKRFKYTEQQNRNTKLSWRVVFPLELRLFNTSDDAVNGDRLYDLVAIVVHCGTGPNRGHYISIVKSHGVWLLFDDDIVDKIDPTTIDDFFGLTQDTPKASESGYILFYQSKEGSDIDSWIKVAQECKYLPESDLKKLCNLVCNILIEENNVQPVSTPVTVCGDIHGQFYDLEELFRCGGQVPETNYVFMGDFVDRGYYSLETFTRLLTLKAKWPNKITLLRGNHESRQITQVYGFYDECQQKYGNANAWKYCCKVFDLLTLAAIIDGEIFCVHGGLSPEIKALDQIRTIQRNQEIPHKGAFCDLVWSDPDEVDSWCWSPRGAGWLFGAKATHEFMQYNGLSLICRAHQLVHEGYKYMFDDKLVTVWSAPNYCYRCGNVAAVLEISDPQNKNPKIFDAVPDNKRVIPERVTPYFLVD-VSAEESQDADATTIEELKEHTNHIQKAVANKESRFILRILRLLPATRKKLNSKLLRKIINGYYTHDKVHKDLLLSFV--EDTDTEAAQKSAHLALLPEVDVYLHLLLLVYIVDSKNMERAVKCSELLMAKVEGHSRRTIDLLAAKSYFYYSRVYELDGKLSNIRGFLLKRLRTATLRSDFEGQAVLINCLMRNYLHYSLFKQAAKLVSKVTFPEMASNNEWARYLYYLGRIKAIQLFYTDAHKNLLQAIRKAPQHTALGFKQTVYKLAVTVELLLGDIPDRATFRQPALRKSLAPYFQLTQAVRTGNLALFNKVLENYGTRFQADHTYTLIIRLRHNVIKTGVRMINLSYQRISLADVAAKLQLDSAESAEFIVAKAIRDNVIEATIDHDKSYMQSAENIDVYCTGEPHCQFDQRIQFCLDIHNQSIKAMRFPPKSYNKDLESAEERREREQQDMEYAKEEDDDAFQVSVKLAVTLSAFAARTLWKSAVHDMIANLKSSHPLLIEFLVALPDQNVK--CLTRDVLALCSSALADALREVSMRAIPPDLCITLL-NVPHNHACDALVNCLTHPDWRLPNTIAEILVQVSRCFIFRSTRSLARARNS--HRYSGVGETHSSVILQSL--QGEKRMEAFLQMLLECIGTPGYYPVDEILSRVPLTFWHLLLDDLSRLEV-SAKGRELHPVYEELVRLLTKSRLPDHGSMDADEMEDHRCYRQDIAD--CYVYVHTLLSKSMFRYLIFELKSAHNWKPIEACLFSLNAVGEMADGIEHGNVVHEVLDLLPQIPVNDEVMSQVMTAIGIFAEQQIGPLVHLLLRGLQEISFAASMALKDLARAHAEHLAPVANDILQAIGILKHRDRVRLVAIVGHVVSALSSDQALQSLTALMAPFVMQLSEMTNLVEQLAPLFSQIAAKY-PTNTKVVNGLAECLRRAVPVLETTPLLKQLLSLCCELQIECASSIDLHERTDLVEAFYTMASLLKKFAIETLLECTPETFTFRSLVQFVTASERIELRKTLEGIVATLIENMHVSRKLIEQEADVFLALNRSASWLNRFLQSQDSKTNFVRQILRERSNKRILTKVV--SDFTVKVKVKWKESY-DVDLDLPEVFKAQLFALTGVTPDRQKVMCKGAILKET-WGATILMMGSKGEIPQPAEKPTFLEDMDASQLATALKLPCGLYNLGNTCYLNAVVQCLRTVPELVKLATYQTQITCSLRDLFWNMEHAVIQPLFLVDFWRRTFPQFAEKGENGQQDANEAWTELVRVLDGFMKMLFGGELVTMKCSESESETTEEFFQLSCFIS-TEVRYLLAGLKLRM-QENITKMSPTLGRDAVYKTSKLSRLPGYLTVSIVRFFYKEKEAVNAKILKDVKFPMMLDVFELCSQDLQQSLIPQRDKFKVWEDEFAFPNDGSNNSGFYQLQAILTHKGRSSSSGHYVGWIRREWFKCDDETVIHEDDIMKLSGGGDWHVAYVLLYGPRLLENLINKMLPKELLLKVFSFLDIVTLCRCAQVSREWNLLAMDGSNWQNIDLFSYQKDINCDVVSYIAGRCGRFLTVISLRGCEDISGEALIQFSEHCPNIEKVLSCCRKITDDAIVALAKSLYIDSCVELTDRSIMSF----KNLRDVNISWCRKITQEGIGMLGS--EHLVRFTAKGCVTNEAMSRLASKLEALDLQCCPYVFDAAIIAVAQNCHELRNLCASGCSNLTDASTQALAQGCLHTLEMASCNRCGDAGFVPLVKACHELRRLDLEECVLITDSTLNSIALSCPFMDSLSLSHCDQITDQGVLKLSQNVIELDNCPFISDITLDCLVDCLQRVELYDCQLITQESIKKFKPGLRLHTYFAPTTPRQRYCRCCVIVSSKGARVFCPDHPEANLIEDYRAGDMICPQCGLVVGDRIVDVGTEWRVFQNEKSSNDPTRVGAAENPLLNGSDLSTIIGRTGDASDESGNAKYANRKTMSSSDRALIGAFREISAMGDRINLTRNIIDRSNALFKQVHDGRSLKGRSNDAIASACLYIACRQESVPRTFKEICAVSKVSKKEIGRCFKLILKALETSVELITTGDFMSRFCSNLALSTAVQKAATHIARKAVEMDIVAGRSPISVAAAAIYMASQASAEKKSQKEIGDIAGVAEVTIRQSYKQMYPRAAQLFPEDFKPANEVPWVEKYRPEKFTEIVGNEETVARLEVFSRQGNVPNIILCGPPGVGKTTTILCLARLLLGSSFREAVLELNASNDRGIDVVRNKIKMFAQTKVTLPPGRHKIIILDEADSMTEGAQQALRRTMENFSKTTRFALACNTSDKIIEPIQSRCAVIRFGKLSDAQVLAKIIDICRKENVSYAEDGLEALVYTAQGDMRQAIGNLQSTHVGFGHVNGKNVFKVCDEPHPLIIKEMIEYCAKGDIDEAYARMQTLYSLGYAAEDIVSNMFRVTKTNTLPEYLKLEFIKHIGLTHMTVLQGLGSLLQLTALLADLCQTIEIKSFVKKEHSLVKPYQGSGMSIPNWDFTGSTMVSSNYIRLTADVQSQQGSIWNKVPWEVQINFKVHGHGKDLFGDGMAIWYTKDPLQPGPVFGSKDFQGLAVFLDTYANQNGHHNHAHPYISAMVNNGSLSYDHDRDGTHTELAGCEAKFRNSEPETSISIRYEQDTLVVSTDILGKKEWKECFRVSGVRLPTKYHFGISAATGELSDNHDITSIKVFELDERREFITPQAAPHRDHVDDGMSGTKFFFVMLFSMLFLVCYYYKKHQENARKRFYRDRDAFDDIRDKNCKIQDINELFLPNDTVTHVPNIKRLNIDPVFPNRTNLLHIHNMAISKAFFLSFILQRAKDDEPGFMYYFMSVISDVAANRFINASAIYYAPNMSFTPSYKGFFNKTMPLFAPRAFRSDDFNDPYHLEGTSTLNTIEATDLGAISLNYSSDQYRINEWYSSWLPDLTKRQDSKTTYTVQITGNNDTFVWHGPPAANDNPGPVKWVRPYFDCGRSDKWVYGATSPIPDIYPRHTQWRHIEIPRYVAVSVMELDFERIDINQCPFGPGN-PRPNYFAGTSRCKNETTDCEPVHGYGFRRGGYQCRCKPGYRRPKIVRNPYHGELIEKASEYEYEHGYSCDKIGYIGVLTQNVNNYMAIGTRIDPLMGGDVVYGKEVQLENEARMAVRLANFISGFMQIVDPKDLFAEFRVPDKPLTADQMIGEVMSIVIGDQKVVGAGVYFDYKAFFGPY--AWRLGRNERKYFVDDTTKIQIRYNSSGIKYDHYPLQYKAADVGYWTSPYFDCGGYHNSWIVTYAVPFFGWDSLRARLQFKGVVAVIELDQLEINQCNAFENTHKCERKSSRCVPILGRGFQGGYKCECLQGYEYPYNDPITYFDGQIVEAPSRFERMTHVSGTDKSKYGGRYMVTALPGDGIGPELIGYVKEVFRYGGVPVDFEEVHLDSSRDDVDLLEQAITAVKRNGVAIKGNIETRHNDPNCKSRNVELRLRLGLFANIVHVTSQPGIETRHQDIDIVLIRQNTEGEYSSEEHTSIKGVVESLKVITKTRSDEIARYAFEWAKNNGRKKVTCVHKANIMKLSDGLFLSRCTEMAKEYPELEFDNIIIDNCSMQLVANPNQFDVLLLPNLYGNILTNLACGITGGPGIASGRNYGKDYAVFETGTRNTGKSIAGKNIANPIAMMNAGVDLLYHLGLTDHAQVIARAIDKTINVDKLHTPDLGGQATTTEVVQNIVKEVQKHARSEM-TQNEIALQDCPTDGISAVKFSNSSNQYLLASSWDEYVRVYDVQQDRCKHKFDHTSPVLDACFYNTTHIWSGGADKTVRLFDLHSGADLRAGTHDDAVRCVEYIPDVNQIVTGSWDGNIKLWDPRRPVGASTHAQDNKVYTLAVCGERLIVGTANRKILIWDLRNMAYVQQKRDSSLKFQTRAIRAFPDLTGYVLSSIEGRVAVEYLDPSPDVQKKKYAFKCHRTKE-NGIENIYPVNAIAFHSKYGTFATGGSDGFVNIWDGNNKKRLCQFHKFPSSIASLSFSPDGSMLAIASSFQHEYTLDPNPPPDQIFIRHHSLENLKKLTVVVADTGDFEAMREYKPTDATTNPSLILQAAKLPQYATLIDEAVAYGKSPSQQLEEAMDKLFVLFGNEILKIVPGRVSTEVDARLSFDKEASITKALKLIGLYKELGVDKERILIKLASTWEGIEAARVLEKNHGIHCNMTLLFNFTQAVACAEAGATLISPFVGRILDWHVANTDKKSFEPLEDPGVKSVTKIYNYYKKFGHKTVVMGASFRNIGEVKALAGCDLLTISPKLLKELADSSDDVPQHLSADKAKNLDMEEVPADEKHFRWDMNEDKMANDKLSEGIRNFAADSRKLEALIQEKLKMGENVCRPLATVYAESNEASGTSAGMPAVFRAPIRPDLISFVHHQLLKNKRTPYAVSKEAGHQTSAESWGTGRAVARIPRVRGGGTHRSGQGAFGNMCRGGRMFAPTKTWRRWHRRVNVTQRRHAAASAVSASGVTALVMAKGHAVEQINELPLVVADKVQDYQKTKQAVNLLKSLKAWSDVEKVYQSKRLRPGKGKRRNRRYKKKCGPLVVYEKDNGIVRAFRNIPGVDTCNVNSLSIFKLAPGGHAGRFIIWTEAAFRKLNDIFGTFSKASKVKKAYKLPRAMMTITDIGRLQKSEEIRAAFRSAKSIIISKKEKPNPLKNPYLLDRLNPFAIVEKRSRILKLKEEAGKNLKKPRALEKMKATKRKRLSNVFPKNRKLLQGSVLKKTLDAIKDLINEGTWDCSAAGISLQAMDNSHVSLVALNLRADGFEKFRCDRNLSMGMNLSSMAKILKCAENNDVITMKAQDDADTVTFVFEANNQEKVSEFEMKLMNLDSEHLGIPDTDYSVVVKMPSSEFQRICRDLSQIGDSVQITCTKDGIRFAAAGDLGTGNISLSQTAEVDKEEEAVIIDMQEAVTLTFALKYLNSFTKATPLSGQVSLSMSADVPLVVEYDMGHLRFYLAPKIDDSERNNVKVCVLGASGGIGQPLSLLLKQHPGISYLSLYDIAHTPGVAADLSHINTGAQVKGFVGQDQLKAALEGIQIVVIPAGVPRKPGMTRDDLFNTNAGIVRDLATACAQVCPKAMLAIISNPVNSTVPIASEAFKKAGVYDPNRIFGVTTLDVVRANTFIAEAKGLDPVSLSVPVVGGHAGVTIIPLISRASPKVDFPQDQLEKLTKRIQDAGTEVVQAKAGSGSATLSMAFAGARFVFSLVSAIKGKDVVECAYVKSDVGEAGFFSTPLLLGKNGLEKNLGLGKLSDFEAKLVAEAQDELKKSVQKGVEFANKEYEWLLKFEVNDIVEQLVIAECSKRFPL-ARSDKFVM-QIKVVATLTGDSISHADITLRL-PKHSQRTIVQNDAQWKLQQIQDAGNHLMQAMNLLKFEFISGQEVRNLMAAVMGCLGRGRACLVVPKKRTIEDIMQGRNM---VPPLPNDVAVSFYVQSYKLVFAVYHV-QKDSQKF-DAECSVPWLSEALVLFTVALQLCQQLKDKVEVFQFNDFLSMFIPVFLEYGAPLGEISDLIRVCDVCFKDAESVL--NGFVSILICGFCKCLEAAPTPKVAFN--VLYNFFEGLRVNVYMSLIKLAGKSVSEVFQDVAPLKKWLPVAEMREVYRALHKELSDMGLKVMVELLSTYSEQDATEARADAERCIAATLADPNTFLMDHLLPLKPIKALQGQPIHELLKIFIYEKVATYKEFYQHNKQLV-DGLGLDHERNVDKMRLLTFMMMAEKQREILFEDIARELDV--VDVEAFTISALKTKLVSAKINQMGRKIVVISTMHRTFERNEWEKLRETLRLDEVEQCTSQ-------------KLEDLIEESKAIDEGEERAVIEESASFQELVKILIEWINDELAPNRIL--VKSIEEDLYDGQVLHKLLETLSGARIDCVEMTQSNEGQREKLKVVLEKASQALGLKWSVDAIHSKNVVAIVHLLVSLARHFRAPVRARLPENVIVSVVSVTKR-EQLTTTYDEYGMKVE-RDAFDQLFDQAPEKLSVVKKSLLTFVNKHLSKINFEDLD-KQFHDGINLALLMGLLEGYFIPLHLSLTP----------------------EAKVNNVAFAFGLMKDAGLKPKARPEDIVNYDLKSTLRVLYNIFSQLSHRSAKIIKSAEQDLRDILSIPPNYKVLFMQGGGTGQFAAVPLNLCPADYVVTGTWSQKAAKEAASFVKFDNIPPESGWKTSPDAAYLYYCDNETIHGVEFPIVCDMSSNILTRPVDVSKFGVIIAGAQKNLGPAGVTLVIVREDLIENASVCPSVLNYKITADNTSLYNTPPTYSIYLLLTLKWIKEHGGVEGMERSAKKSKALYDLIEASEGFYCRSRTTIPFRI----RCDEALEKKFLAEAEMIQLKGHRSVGGIRASMFNAMEVEQALVLYKDRSDWADVKPVINIAYSETFRDCYGYLRAVLSGELSERVFELTTTCADENPSCYTVWLLRRKLIAHLKKDLREELDFMVTQIQENQKNYQVWYHRQKMVEWLELEFIRNMLDAKNYHAWQYRQWILRKFNLWDGELAVCDEMLAKDCRNNSAWNQRYFVVLNSTGFVMDSEIEFTLDSVRYNESSWNYLRGIFSVFSLSILLDLALKADLLRANYWDFRARLLAVIVDRFEELIEFVPHIGRFLKAQGVAYRIIVVNQGDTYRFNRGALINIGYHVSKAQCDYLVMHDVDLLPMNSKLSYRYPQQEEVHLAAPHLHPKYHYATFVGGILMMRHETFARLDGLSNKYFGWGLEDDEFYVRIKEAEFTLERPVDIGTGINNTFKHMHDARRRPRDMARLGNQREESRRRDRVTGLHNVVYRLQSHHKMQIDGISADVFNVFLHCDVTRTPWCEKPKVSPGTTITTDGFMRGHGGLLSAVAGVVEKVNKLITVRPLKTRYNPEVGDVVVGRIVQVC--QKLWKVDVGGRLYAALHLHSVNLPGGELRRKSIEDELLMSQYLIDGDLVSAEVQNVGVDGAVSLHTRNLKYGKLGQGSLVQVSPSLVKRCKHFHNLP-NGVHLVIGLNGFLWITSSHFARESICRTRNVIISLSRYNIMLYDTTILYAYEIS-VAHLTRQGKDKLVGGWLLTCSGMAFGAVVLGGITRLTKSGLSMVDWHPFDEGRPRTQDWQREFEKYQQFPEFKITNRDMTLEQFKSIYWMEYIHRMWGRTIGAVYFIPAAAFWALGYLRGGMKQRVVLMGGLLAGQGLMGWYMVKSGLEEKPRVSNLRLAAHLGTAFVLYAFLFRSGLQLLLPTQALRKLIVSKGLVFFTVLSGALVAGIEAGLVYNSFPKMADRWIPTDVLAFPKWRNFFENPTTVQFDHRILGESVITALYLYSRKVPMPPRARFAANVVLAAWMQVALGITTLLNYVPTHLAATHQAGALTLFTTLLWLTHELKIDKK-LAKWGEGDPRWIVEERPDATNVNNWHWTEKDATQWSKDKLNALFTNLEIED-TILSVITEVSKFEGDAVVNNRKAKLIFIYDLNLELCAGS-VVKGKVEIPNLSEENDLDEIVIDVMLSDEKIKAMMRSKGVDVIRDKLSDYLNALRADYSQGLILPTKG--LNVTSTKGKKINTTNLTMEEFKCTAYELYRVFTMVQAFSQGPAGGKILMLDTNVSGKFLTLDRLEFSWRFKSWPAEHYSKVMTLDQTNDSTMIQEGVPEAELERTRAGWQRYYFDAIKRTFGFGAILMDPETFLEVANHVSKLKMYPYFEIAHCAVTLLYLREDLASGSHLFSRKHPLSCYISSMFSIFAGYMFSALLLGEPVLSAFKNNQSLILASAVWYLMFYSPFDVVYKFCKLLPVKLLLSLAKEITRAKKVHDGVHHAAKLYPNGYLIMVIIGVIKGNGTSFLKVYERLLRGYWTPNAIEIMQPSFATKICVIASIVFVVDKKTDLISAPHSLVYFGVVCFFLYFKLSSMLLGLHDPFLPFENLTCAIFFGGIWDAISRALKDVAAKKKEMSRPDG-VRRRTPDKKDDDKETRMTLMEEVLLLGLKEKEGYTSFWNDCISSGLRGCILVELGLRGRIDLESAGMRRKSLLMRKVIVKNDAPVGDVILDEALKHIKETSPPDTLQNWVDYLSGETWNPLKLRYQLRNVRERLAKGLVEKGILTTEKQNFLLFDMTTHPLVDQNVKDKLIKRVQDSVLSKWANDIHRMERRQLSLLMLSHASDVLENAFNPLSDEDYEMAMRRVRDLLDMDFEAECAKSQSCDIMWGVFAAFVKMADNVPTFKCVLVGDGGTGKTTFVKRHLTGEFEKKYVATLGVEVHPIVFHTNRGAIRFNVWDTAGQEKFGGLRDGYYIQAHCAIMMFDVTARITYKNVPNWHRDLVRVCENIPIVLVGNKVDVKDRKVKAKAIVFHRKKNLQYYDISAKSNYNFEKPFLWLARKLIGDPNLEFVAMPALAPPEVQMDPEWQSKLENDMRDAQNTSLPDDDEDDLLAAQMTPVLKESRFRESGMLTPEEFVAAGDHLVATCPTWNWAKG-DKTYLPEDKQFLVTRNVPCSKRCRDMEEKVIEDG-EGWVDPYDNEDDDDGAVDMDAFLDEDPGAAGVLSTRTYDLNITYDNYYRTPRLWLTGYDEYHQPLRTKELYEDISQDFAKKTVTVEPHPHLDGPPQASVHPCKHAQAMKNLIQTVEDGGGLEVHMYLIVFLKFVQAVIPTIDYDYTTNFNMLSNLEKKLPYDKLSANIDVVKKRLNRPLTLSEKVLYSHLDQPASEEVVRGESYLKLRPDRVAMQDATAQMAMLQFISSGLPKVAVPSTIHCDHLIEAQHGGSQDLARAKDINKEVYNFLATAGAKYGVGFWKPGSGIIHQIILENYAFPGLLMIGTDSHTPNGGGLGGLCIGVGGADAVDVMAGIPWELKCPKVIGVHLTGKMSGWTSAKDVITKLAGILTVKGGTGAIVEYFGPGVQSISCTGMGTICNMGAEIGATTSVFPFNSRMADYLAATNRTAIADAASEVKDLLTADSGCKYDQVIEINLDTLEPHVNGPFTPDYAHPISQLGKVAKEKGWPLDVKVGLIGSCTNSSYEDMSRSAMLAQQALDHGLKSKSLFTVTPGSEQIRATIERDGQAKTLKQFGGMVLANACGPCIGQWDRQDAKKGDQNTIVTSYNRNFTGRNDANPQTHAFVTSPELVTALAIAGRLDFNPLQDELTAADGTKFKLQPPEGDELPRAGFDPGEDTYQGPPNDGTGVKVDVDPKSQRLQLLTPFAKWDGGDLQDLVILLKAKGKCTTDHISAAGPWLKYRGHLDNISNNMFIGAIPEESGEANKVQNRLTGEKGGVPDVARQYKAKGQGWVVIGDENYGEGSSREHAALEPRHLGGRAIIVKSFARIHETNLKKQGLLPLTFQNPSDYDKIKSDDKISIVGLKDFQPGMEARGGMFKNTFQSGFLSILYSLGSKPLQIWDKKVRNGHIKRITDNDIQSLVLEVMGSNVSTAFITCPADPRETLGIRLPFIILIIKNLKKYFTFEVQILDDKNIRRRFRASNFQSTTRVKPFICTMPMRLDEGWNQIQFNLADFTRRAYGTNYVQTLRVQIHANCRIRRVYFADRLYSEDELPAEFKLYLPVQEKCLSSKYQYVHKARKDIQAASDHYKGLAETFIFNDGSSKELVCLDGTIPVRYKYYFPVRVWVLDTHPYHAPLCFVCPTPTMQIKVSRNVDESGRVYLPYLHDWNGNTGSDIVGVLKVMIMVFSETPPVFSK-PLPYPSSNTLSITDEHIRISLLSAVESRITDRALEKSKAEEEVLKKTNEELIQGKAKLQKFMSDMENDRELDSEKNEQLKLVDIDNAVTTTAPLYRQLVNAYAEESAVEDAIYYIGEGLRKEVIDLDTFLKHVRELSRKQFMLRALMQKCRQKAGLPMGRKFFVGGNWKMNGNKASIKEMCDRLKNAKFCVGVCVAVPAPYLMLCRESLPDSIRVAAQNCYKVASGAFTAELSVDMIKDCGCDTVILGHSERRNVFGEKDQLIAEKCAFALQNGLTVIACIGELLEEREGGKTEEVIFRQTKAYADLIKDWENVIIAYEPVWAIGTGKTASPKQAQEIHAKLREWLAKNVSEEVSRNTRIIYGGSVTAANCKELAQEPDVDGFLVGGASLKPEFVQIINANQMPGQISKKRRFVADGVFNAELNEFLRRELAENGYSGVEVRNGATKTDIIIMATRTQDVLGEKGRKIRELTAVVQKRFGFKEGTVNLFAEKVSARGLCAITQCESLRYKLIGGLAVRRACYSVLRCIMEAEAMGCEVVVSGKLRGQRAKSMKFVEGLMIHSGDPTNHYVETAVRHVLLKQGVLGIKVKIMHPHDPLGKRGPAQLLPDKVHVVDSPHEDDNVEISSDNKDTAISKIEQIKQWSLSTYKCTRQILAEKMGKGIRTVDGELEANIELLRETHQKYLNILRLAKLLTSHFNTVATQAALGECFSDLAQK---ELQQEFLYNAETQKNLSKNGETLLGALNFFVSSLSTLCNKTIEDTLITIRHYENARLEFDAYRCEAKASFRDKYERLRGDVQIKMKFLHENKVKVMHKQLLLLHNAVSAY-FSGNQSSLEATLKQFNISWLEQ-MVDLVLDRDIRIWVFLPIVLLTFLLGVVKHYVSILITTTRKPELQQVYDSQALIRVRYLRENGKFLPLKSFLMRKHYFNDEDSGWLK-TQNRPAPASNPMQDPGMMTEMLKGNLINVIPMIVIGGWINWTFSGFLTTKVPFPLTLRFKPMLQRGIELVSLDASWVSSASWYFLNLFGLRSIYALVLGEDNAADSTDAMQEQMTGAAMKI-TDPKAAFKAEWEALEVVEHKWMLANVEEMNMYKGAASEAGRAMQILKRRERQREEVELKKAKIEQEMKVT-MDDKFSSHFDAVEAQIKSATVGLVTLDEMKAKQENAVKEREKRLAQKEQEEKQKHEKRKRAQKEKQKKAIQALSFSMDDRKMKKNPDVDTSFLPDREREEEERRIREELRQEWTDKQRKLKEEAIQITFSYWDGSGHRRVVEMKKGNSIYQFLQRCLETLRKDFHELRVVSADQLMYVKEDLIIQHHYTFYDFIVTKARGKSGPLFSFDAHEDVRMTSDASKEKEESHAGKVLLRSWYERNKHIFPASRWEPYDPTKCYDRYTVKDKKP-KPTVPETLLKQRKNNAELRQQRILAVAAKKKADRARRVLAFQRAEKYVREYRQKEKAEKNNRLVAKLEGNFFVPDEPKVALVMRIRGINGVSPKPKKVMQLFRLRQINNAMFVRLNKATINMLRIAEPYLAWGYPNLRTVRDLIYKRGFARINGRRVPLVDNSIIEEKLGKYGLVCMEDLVHEIYTVGPNFKQAVNFLWHFKLNSPKGGWRKKTTHFVEGGDYGNREAFINRLVRKMISKVTAEHLWKGVTSVSNAGRKRGRASGSSRKMARDLNRGQVLGVGKINMVWPGLSAPAIRGQEIIRQQKLEPDPEREKRLTALRNK-HGVRRTKIAPLDRGWSGGKAGGRYIGPPDPVGDENFEGFKSCVVKMGMVFVMRGNTGRTRQHKSIVIVGNGNGLIGFAQGKAGDARSALRKAKNGAAKNLVYIERFENTVLHDFYTEYGSCKLIVKKKQRGYGLVCQRVVRELCKVIGIKDIFVKADARRMNTLSVIRAFLLGLHNQRSLQSVADEKRLHVVEFREEYGYFPKVVASPKDVRTAEEINPNEELDFNMIINGGRLVMMPKKKTPFYWNLPGYQIYLKKTDPLKNHRTTHLRILKRYGALKSFLTIREKQASLKSRNSDDRNRWTNRQRLLIFASRGITYRDRHLMNSFKGMLAHSKQECKFE--KKDINEIAEMKNCNRVMYFENRKKSDTYMWLANMETGPTLKFLVQNVHTMEELKFTGNCLRGSRPFLSFDPGFDEHPVVKEVLAQTFGTPAYHPKSQPFFDHVFTFRILDKRIWFRNYQVVEE-DGSLVEIGPRFCLNLVKIFDGPFSGAIIYTNPNYVAPNKARRLAKQD-------------------MAVGKNKGLSKGGKKGLKKKIVDPFTRKDWYDVKAPSMFQIRNVGKTLVNRTQGTKIASDGLKGRVYEVSQADLNNNEDAFRKFKLVCEEVQGRHCLTNFHGMDLTTDKLRSMVKKWQTLIEAQVDVRTTDGFVLRLFCIGFTKKTQSQVKKTCYAQHAQVRAIRRKMVEIIQREVSSCDLKDVVSKLIPESIGKDIEKVCNAIFPLHDVLIRKVKVLKKPKFDMSKLLELHGDSKGSSGTDGMAVDRPDDYEPPVLETV????????????????????????????????????????????????????????????????????????????????????????????????????????????????????????????????????????????????????????????????????????????????????????????????????????????????????????????????????????????????????????????????????????????????????????????????????????????????????????????????????????????????????????????????????????????????????????????????????????????????????????????????????????????????????????????????????????????????????????????????????????????????RLPPLPTPAELLRLYRLKALKQMSQNFLLDPICRKLIRSAGHVIEVGPGPGNLTRPILELGA-TCSVIEKDLRFMPCLDLLAEAAE-GRLKVIHGDVLTYPIHSEI-PPELARPWESIHLIGNLPFAISTVLLVKWLKEISERSGPWQFGRVRMTLTFQKEVSTRITLMEKCRLSIISQGCEVEEGGSFVPPPLVDVGVVKLVPVKPVFQDFSLVEKVLRCMFNKTVEQDIKHDVLEKAELDPTLMLTTQEFSRLCDVYAEYCEKAGLYEYNYRGPPMVPIVIEQTGRGERAYDIYSRLLKERIICLMGPINDDIASLVVAQLLFLQSESSKKPIHLYINSPGGSVTAGLGIYDTMQYVLPPISTWCVGQACSAASLLLAAGEQGMRHSLPNSRIMVHQPSGGVSGQATDIQIHAEEILYLKKKVNRIYAKHTKQPIEAIDSIMERDRFMSPEQAKDFGLIDTVLEQPPIRFERPVGTWLTLLPLWSLTMATPAGQLPDIALFGGGAFLMRGFGCTINDMWDKDIDKKVERTRARPLASGELSRWDALWFSAGQGMACLILLQLNWESVQLGAASVGLVVLYPLMKRFTYWPQAFLAVVFNWGVLLGFSAAALPLYAAAFSWTMVYDTIYAHQDKHDDLMIGAKSTALKFGSRTPLWLGAFTTMTTNLQTWPYYSAVTLDTSNVGLLIGCIAGTLLKVARVDRPKTRKGSRIVKAREPLTIENPKTSFVKAANINQRTAQILKDLYTLKKTESVFYQKKNPFEDVSHLEKLAKKDTSLFAFGSHNKKRPQNIVLGRTFDRMVSDQFEFGVENYKALDEFKVAKIGIMVKPVLIFAGEAWQEMKRLKNFLIDFFRGEYLGVSGLEHAISFTAMGILLRSYKIHLKRSGQKTPR-AEVEEIGPRMDLKLRRHKIASDDLFKQALRQPKGLKVKKKKNLEQDDLGTSLGRVHMERQDFRLQTRKMKGLKVTIIGALDDNYMYLITTKEAAIVDPVNPEKVLETVEVNLTTVLTTHHHWDHAGGNEKLLVYGGDHRIKQLTDRDEVEIKIGEYNDTRFTPCHTTGHVCFYIVFTGDTLFLAGCGKFFEGTAAHMQEAMLGQLPDDTLVYCGHEYTVNNLKFAQQVESDNQTQRKLEWAAHQPTIPSTIAEEKTYNPFMRTAVQKHADAVSTMDSLRCEKDNFRMNRLFGKAKAKEPPANLNDCISTVDQRANNMDEKIQKLDKELIKYKEQLSKMREGPAKNSVKQKALRLLKQKKTYESQRDNLLQQSFNMEQANFTTQQLKDTKVTVEAMKLGVKEMKNEYKKVNLNEIEDLQDDLEDMLEQANEVQETLGRSYNMPDMTDEELEAELAELNDELALDDNSYLDSVRHLSKSRGKATLPDLPYDYNALEPVICAEIMQLHHSKHHNAYVTNYNISSEKLQEAVSKGDVSAQIALQGAIKFNGGGHINHSIFWQNLCNPKSGEPSAELQAAITKDFGSLESLKEKVSAAAVAVQGSGWSWLAYNKATKSLQVAACANQDPLEATTGLVPLFGIDVWEHAYYIQYKNVRPDYVKAIWKVANWKDVSQRFANAKDGHIQHDVIKEALESGRDLREYSRSVDQQLKGTEDEAIKDYMENCKDIAALHNEIASCDGILQVMENILRGFQNDLGSISSEIQSLQRQSVAMNLQLKNRQAVKGELSQFVDDFIVPESTINVILDCPVTDEEFLAQLSLLDQKISFVKVQSFKEASSCQDVKDILDKLKVCAVTKIREWLLQKVFSFRKPNANFQLPQNAMLKHKLFFQFLATHEREVAKEVREEYVNTMSKVYYSYFKAYHSRLMKLQFDDVPDKDDLMGVDDTPKWGLFNKPSLKNRSTIFTLGNRNAVMSVELEAPVIVPHASAKNEKHYPFEQLFRSMQYALCDNAAREYLFISEFFLLTKSGAAEAFDSIMGKSMSMFAKYTETFVAECFDSIALFLCIHVVHKLRILMHQRNVPVLDSYWDVLVANIFPRFETILRLNINSIRDCDPSKLGSIDNRPHYITRRYAEFSAAIVSINENHPDERVSILLGQLQMEVENFILKMAAEFNGRKDQLIFLINNYDMMLGVLQQRTHEDSKETTNFRTLLTARQNEYVEQILTIHFGGMMTFIKECEFYIEKGQSEKLEKESHKVATLVRGFNSGWKKAIDDMNADFMKTFTNFKCGTNILQEALKQLLQYYHRFNKVVSQPPLNALPVRSELINIHHLMVDIKKYKATFSYFIVVDAHAEECFHDRVAKGTKMGLTFEVVEGGFLDIDVKITGPDGKVVYNGERESSNKYTFAAYAEGMYAYCFSNAMSTMTPKTVMFSMDIGEEPKEEGKPAADAGDTKLEDMINELHTAMTGVKHEQEYMMIRDRIHRSISESTNSRVVIWAIFENLVIFAMTFGQVYYLKRIFEVRRLVMRLTRVLKLPKSWSNFPERYIKRSMEEIEYKTPVGRQYRRAVIKRPYGLDRPWTDGFKKNEPGKLQIIEPVFKGDRVQILVGKDKGKQGIVNYIVKERNWVCVSGLNCKEQPLVMFEQVALVDPTDERACKAQWRYTENNERVRISLRSGREIPIPLAEETYDYKYKNQPKDTSAADLVKITFLP-KLATFEMDIMEEHGIKDDRIPAEFYWYVEKHFDSLLWISRMSTIMFTVLYFLG-PSSYYQKALMTNGVTSALRLHQRIPEVRLNVQFLARLVTEDSFHYLFYSFFFLLPVSVMVLVPPLLFAVLHSIKLLTKAGTIYL---VRVSLFQIIAMTEIMLMVILIIGVFTGMMLMAPFIYYRFLSQRYASNRNPYSRYVCRDLRVQLESAARRSCPPFISRTMFVSSRAPIAISAITVYLFFPEMEPEEKPYVTLPTSLESAKDLGRVLSNYTDDMVLLAFFCTYIFLQSFAIPGSIFLSFLSGFLFPFPLALLTVCLCSAIGASLCYLISYCVGRRLIMHYFPNMLYYIIFLRITPFLPNWLINVASPIVSVNLAPFFLGTFLGVAPPSILAIRAGISLQQLASAFTLENGLLLTGFAVLSMIPVVLRFKNKFEDLQDKLTRIAIVSTDKCKPKRCRQECKKSCPVVRMGKLCIEVTPNDKIAEISENLCIGCGICIKKCPFEAIMIINLPSNLEKDTTHRYSANSFKLHRLPTPRPGEVLGLVGTNGIGKSTALKILAGKLKPNLGRYNDPPDWTEILAYFRGSELQNYFTRILEDDLRAVIKPQYVDQIPKAVKGTVRQLLDKKDEMGKKTHLADILELNSVMDRQIGDLSGGELQRFATAMVCIQRGDIFMFDEPSSYLDVKQRLKAALAIRGQIEATKYVIVVEHDLSVLDYLSDFICCLYGTPGCYGVVTMPFSVREGINIFLDGFVPTENLRFRESSLVFKVSDNTDEDVKRICRYEYPTMVKIMGDFKLQVKGGSFTDSEIIVMLGENGTGKTTLIRMLAGKLAPDGGETVPALNISYKPQKISPKTQGTVRFLLHEKIRDAYQHPQFVADVMKPLLIDSIIDQEVQNLSGGELQRVALALALGKPADVYLIDEPSAYLDSEQRLAAAKVIKRFILHAKKTGFVVEHDFIMATYLADRVIVFEGVPSVDTVANAPQSLLVGMNRFLELLNITFRRDPNNFRPRINKLNSVKDSEQKRNGTYFFLEDEDEGEDLFGPELDRYDAAEEEMRRRDRGGMRRGLESIENLEDMKGHSIKDWVTQGPKTEIFNRFKNFLRTYKEKIRAMEQNKMSLEVTYNLAQSEQILAYFLPEAPAEVLPIFDEAAKDIVIGMFPHYERIHHEIRVRITELPILEEIRTLRKIHIDQLIRTSGVVTSTTGVLPQLRMVKYDCAKCKYVLGPFVQSQEVRPTSCPECQSTGPFILNVSQTIFQDYQRITIQEAPGKVSAGRLPRSKDAILLNDLCDSCKPGDEIEITGIYSNKFEGSLNKANGFPVFATVIIANHILRKDTDEDVKEVVKLSKEDLAERIMASIGPSIYGHDDIKRAIALSLFGGVSKNPGDKHRIRGDINVLLCGDPGTAKSQFLKYVQQIAPRAIYATGQGATAVGLTAYVSLVTRDWTLEAGALVLADKGVCLIDEFDKMNDADRTSIHEAMEQQTISIAKAGIVTSLRARCTIIAAANPIGGRYDTFHQNVNLSDPILSRFDVLCVVRDERLARFVVDSHARHHPIEQELLQKYILYAKDKIEPKLDQDKISQLYSDLRRESMVTGSMPITIRHLESIIRLAESHARMHLREHVDVNMAIRVMLDSFVTQKFSVMRMGLLSDIKEIFEVDCLYEVFDVEKTATTNDIKKAYRKKSLMCHPDKAPAEKKDEFTRKFQTLCKTYDLLQDEERRKVYDETGDV--DDAIDSNRNWDTYWRNLFPKVTLKCVDDFLKKYIGSELERKDLKKYYERFKGDMNKISQCHIGYNEDRLNVVLLTALVFLFLGKIGTKKLRKLEEKAEKRRLRELELQEREERKQKQAEDERRKKEDQKRDEEEKRQEELERKAKEDAERREHEEYLRMKAAFDIEEEGFDQEED---SPGRLTEFIKYIDEQKVVQLEDLAARFKLKTQDCIDRLHRMIEEESICGVIDDRGKFISITKAELEEVAKFIKLRGRVSIQELVENSNRLINLSVDILEQQEVAKSTGLKHVITMLQIPDQLDKVDQHRKRVQRKKASVEAMLKTAVQSQLDGVETGLSLLVSARDDIADCQKDEVEQIYDLAKLLQLQDVREESIKHSQTGTLMEHLKHIFNVPGSVARTQDLIQEGKFLLAHKLSDLEGSRDDLLYELHKQANNLPSDKAMLKQYFADVERLSDELGKQLWVILKRTLNTVRKEPQVIVTALRLITREEWAALRRQETT-GFLPPSRPKLWKKKAIETLEQSVAERLEANQIEGRQENKMWLVRHLEVTRQLIIDDLKTVKHHCTPCFPPSFDIFNEVRMIHNCLSQRLQTIISGLVDSEYIHILGWLNTYNSRELMQHPELNVDIALLPDTIKKLMQKYLAGLQVKFEEWLRNALTDHKDWPETDSDGYHRTEAPMLIYQMITQHIDVARTVTLISLVLNLAMEHMNNFLTSYIQLVTEYNFEDRSYTAYMIAVANNAVNMK-LKTLKENALSYLCEEVLMDIKATPNIMTDTVIVTLADYGNDYIEKQVASSYVRAIC--EKRISFKNYEERKTAAELNKLEKSELTVLKLMAEVLKMKDNSLLSLELSGGRTDAEVVGREEEAIKIDSLSVSQLKEIRDKAEKQVFQAEVARMMKLIINSLYRNKEVFLRELISNASDALDKIRLLSLTNPDALKALQELSIRIMADKENNVLHITDTGIGMTKEDLVKNLGTIAKSGTAEFLQKVNDGEGSKDLNDLIGQFGVGFYSAFLVADRVAVASKNNDDDVQHVWESNASEFTVADDPRGNTLKRGTTVSLYMKDEAKDFLEHDTLKKLIEKYSQFINFNIYLWSSKT-VTEEVPEEEKDTTET----DEDDEAKVEEEKEA--PKMKKVEKTIWDWDLINSAKPIWTRKEKDVADEEYNEFYKAVTRDSQNPLARTHFTAEGELTFKSLLFVPVKQPQDSFNKYGQRTDHIKLYVRRVFITDDFQDMLPNYLSFLRGVVDSDDLPLNVSRENLQQHKLLKVIKKKLVRKALEMFRKISEEDFAKFWKEYSTNIKLGVIEDSANRSRLAKLLRFPSSIDSADKLVSLSDYVQRMKEKQSAIYYIAGGSMDEVKKSPFVERLLKRGYEVLFLTEAVDEYAISSLTEFEGKKFQNVAKEGLSIDE---NKEIREALEKEFEPLTKWLTETALKDKISKAIISERLVETPMALVASQFGWTGNMERIVSAQTHMKENDPQRQFYMSQKKTLEVNPRHPLIKELLRRVDDSPSDEMAKYFTEMMFDSATLRSGFQLSDNARFATNIEKMLRNMLGVSEEAQVDAEPGPRGEVKALSSEESKMIVISEIIQELVLAHNEKRDVNLNRVKCDASARHGMKSQPKLVDIIAAIPPQYKKILLPKLKAKPVRTASGIAVVAVMSKPHRCPHINYTGNICVYCPGGPDSDFEYSTQSYTGYEPTSMRAIRARYDPFLQTRHRVEQLKQLGHDVDKIEFIVMGGTFMSLPEDYRDYFIRSLHDALSGHTSSNVDEAVKYSERSKTKCIGITIETRPDYCLQRHLSDMLRYGCTRLEIGVQSVYEDVARDTNRGHTVASVCETFHMAKDSGFKVVTHMMPDLPNVDFERDILQFVELFKNPDFRMDGLKIYPTLVIRGTGLYELWKTGRYASYPPALLVDLIAQILSLVPPWVRIYRVQRDIPMPLVSSGVENGNLRELALARMKDLGLVCRDVRTREVGIQEIHNKVVPYHIELIRRDYVANGGWETFLAYEDPHQDILVGLLRLRKCSEQTFRPELMGQCSIVREHVYGSVVPVHSRDPTKFQHQGFGTLLMEEAERIAREEHNSTKIAVISGVGTRNYYRKLGYQLDGPYMSK

'Varroa_destructor' PGQRVNVLSKELMTSVVLISGKKGCFIAGADITMLEQCRSEAKNLKPIVAAIMGSCLGGGLETALACRYR-IAVEEPKTTLGLPEVMLGVLPGGGGTQRLPKLIQLPTALDMMLTGKSLHAKKAKKVGLIDAIVKPLGPGLYLEEVAARDLATGELKIRARPLTERIVRDM-IFDKARGQVMKLTNGLYPAPLKILDAVRAGLEKQNFAELCTKESKGLMGLYHGQVHCKKNAFGKPTENIAVLGAGLMGAGICQVSLKDFNRVVMKDGLVRGQNQIKKKKIQKDRLMSTLLPTLDYSDMIIEAVFEDIHVKHKVVKEVEAEHCVFASNTSALPIAKIAEVSKRPEKIIGMHYFSPVEKMQLLEVITTDKTSKDTAATAVDVGLRQGKVVIVVKDGPGFYTTRILAPMMSEAMVLLMEGCQVKELDKLKAFGFPVGAATLLDEVGIDVGAHIAEGVFGDRKEMVNNFLGRKSGKGCYIYRAVNPIQRKYTTEQVQFRLATRFMNEAVMCLQEGILANPVEGDIGAVFGLGFPPNRGGPFQFIDTYGADKIVNMRQFQEFEPCQLLLDHA-NDPLKKFHMQRPIILLQEGTESQQGKTHMMSNINACQTIGDAIRTTLGPRGMDKLMVDGKGKTVISNDGATIMKQLDVVHPAARTLVDIAKSQDSEVGDGTTSVVLLASEFLKQAKPYIEEGLHPQVIAKAYRKASKMAINKINEIAVKVDKGEMRALLEKCAMTSLSSKLVASKKQFFAKMVVDAVLQLDELLPLNMIGIKKVSGGALEDSVLVSGVAFKKTFSYAGFEMQPKQYSSPKIALLNIELELKAERDNAEIRVDNVEEYQKIVDAEWSILYDKLAKIHASGAKVVLSKLPIGDVATQYFADRDMFCAGRVVEEDLRRTMKACGGCVLTTVQDLKYSNLGSCERFEEVQIGGERYNIFKGCPNSKTVTMILRGGAEQFIDETERSLHDAIMIVRRAVKNDAVVAGGGAIEMELSKYLRDYSRTVAGKEQLLIAAFAKALEVIPRQLCDNAGFDATNILNRLRERHAKGEKWTGVDMNLEDIADNLSACVWEPAVVKMNAITAATEAACLVLSVDETIKAPQSNTDPSAGRPFAKVLNRLEAFREHEMAVTRDYISQPRMIYKTVCGVNGPLVILDQVKFPKFAEIVQLVLADGTPRTGQVLEVSGDRAVVQVFEGTSGIDAKNTVCEFTGDILRIPVSEDMLGRVFNGSGKPIDKGPPVLAEDFLDIQGQPINPWSRIYPEEMIQTGISAIDVMNSIARGQKIPIFSAAGLPHNDIAAQICRQGGLVKRPQKSVMDDNFAIVFAAMGVNMETARFFKQDFEENGSMDNVCLFLNLANDPTIERIITPRLALTTAEFLAYQCEKHVLVILTDMSSYAEALREVSAAREEVPGRRGFPGYMYTDLATIYERAGRVEGRNGSITQIPILTMPNDDITHPIPDLTGYITEGQIYVDRQLHNRQVYPPINVLPSLSRLMKSAIGEGFTRKDHADVSNQLYACYAIGKDVHAMKAVVGEEALSPEDMLYLEFLGKFEKNFISQGRYENRTIFESLDIGWNLLRIFPKEMLKRIQHSLLAEFYPRAEAKTGLFEDPNGFYLLKENAITQAEQLIAEAMYRKRKMVQIFDDLSDCLCRVADLAEFVKVGHPQGRYAQAAEHASLAISSLVEKLNTNRELYSALRSVIENG-DIVPTTAEQHVGRLFLFDFEQCGIHLDEERRQRVVALNDHILYVGGQFLQNSHRPRYVRQSGMPENLLVVSGLQADCSNELVREAAYRIYLYPDDHQLGLLDELLRSRHELARLCGFDTYAHRVLKGSIAETPENVTFLSYLSAELGPRAERDYQEMMGMKPWDVPYFTAYLSLASCMEGLDMIFNALYGINLEVVGELWHSSVVKLVVKNLASHMGVIYCDLFERPGKPHQDCHFTIQGGRRSDGSYQIPKVVLMLNLPPPLLTPSLMDNLFHEMGHAMHSMLARTEYQHVTGTRCATDLAEVPSILMEYFASDPRVVSKFARHYRTGELMPAEMAASLDASRVIFQASETQLQVFYAFVDHEYHSKYPLNTTEILRDVQNKHFGVKYVDNTAWQLRFGHLVGYGAKYYSYLMSRAVAATFWHRAFNADPFSRCVGTYREEVLAHGGALPPAQLIQNFLLAESLIRDIMPSDAKKKRDAKKKEALKNRNNPDATNGEDEMDEVTKKFEEDMKLNAAARAVTGVLSIHPRSRDIKIENLSITFHGWEVLQDTKLELNCCRRYGLIGLNGCGKSTLLSAIGRRELPIQECLDIYHLTRECPPSEKTALQMVLDVDKERARLEKLAEELAASDDDTSQEQLMDVYERLDAMSADTALAKASYILHGLGFTQSMMHKKCKDFSGGWRMRIALARALYVKPHILLLDEPTNHLDLDACVWLEEELKTYSRILILISHSQDFLNGVCTNIIHMNLRKLEYYGGNYDQFVITRNEMLENQMKRYNWEQAQMSHMKDYIARFGHGSAKLARQAQSKEKTLAKMVAGGLTDKVVYDKTVSFYFPSCGTIPPPVIMVQNVSFRYTDKTPFIYKNLEFGMDLDTRVALVGPNGAGKSTLLKLLCGALVPTDGIIRTHSHLKIARYHQHLHESLDVDLSALEYMMKSFPDVREKEEMRKIIGRYGLTGRQQVCPIRQLSDGQKCRVVFAWLAWQVPHMLFLDEPTNHLDMETIDALAEAINNFEGGMVLVSHDFRLISQVANEIWVCENQTVTKWRGDIKTYKQHLKNKVMKEMEKMVLADLGRRITSALRNLSTATVINQEVLDSMLKEICAALLESDINVRLVKQLRENVKAAIDIDEMAVGLNRRKVVQSAVFKELVKLVDPGVRAWQPSKGRSNVIMFVGLQGSGKTTTCTKLAYYYMKKGWKTALVCADTFRAGAFDQLKQNATKARIPFYGSYTEVDPVVIAADGVSKFKAEHFEIIIVDTSGRHKQEDSLFEEMLEVSNAVSPDNVIFVMDASIGQACELQARAFKEKVDVASVIITKLDGHAKGGGALSAVAATRSPVIFIGTGEHIDDFEPFRVKPFIQKLLGLGDIEGLIDKVNELKLDENHELIEKLKHGEFTLRDMYEQFQNIMKMGPFNQIMGMIPGFSADFMSKGNEQESMARLKRLMTMMDSMTDEELDDREGAKLFARQQTRITRVARGSGCSTFEVHELLNQYTKFAAMVKKMGGMKGLFKGNDLARNVNPAQMNKLSAEMAKMIDPRVLQQMGGFSGIQNMMRQMNASSLMQDGTFIKLKKLYSMKKGTLNINSLFENDPSRAEKYTIKLADGDETLLIDYSKNLIDDEILVNLVELAKNREVELMRAKLFSGEKINFTENRSVLHVALRNRSNRPITADGEDVMPKVNAVLEHMKAFCQQVISGEWKGYTGKKITDVVNIGIGGSDLGPLMVTEALKPFQVGPRVHFVSNVDGTHLFETLKKVDSETTLFIIASKTFTTQETITNAESTKQWFLDKAGDKAHVSKHFVALSTNKPKVEAFGIDAANMFEFWDWVGGRYSLWSAIGLPIALFIGMPNFEKLLAGAHFMDEHFRTTPLDKNVPVILAMVGVWYINFFGAESHCLLPYDQYLHRFAAYFQQVDMESNGKYVQRNGERVDYQTGPILWGEPGTNGQHAFYQLIHQGNRLIPCDFIAPVKTHNPIRGGVHHKILLANFLAQTEALMKGKSEQEAKSELKASGLSEEVLERILPHKVFLGNRPTNSIIVQQVTPFTLGALIAMYEHKIFVQGVIWNINSYDQWGVELGKQLAKRIELELNGKDPVSSHDPSTNQLINFINSYNTNVSYQDRDKPAQVRQSNITASKAVCDAVRTSLGPRGMDKMIQAVSGDVTITNDGATILQQMQVLHPAAKMLVELSKAQDIEAGDGTTSVVVIAGSLLDAASKLLLRGIHPTIISEAFQAAAKECVDILSCLAIPIELSDRESLLQSATTSLCSKVVSQHSDVLAPMAVDAVLKVIDPNNVDLRDIKIIKKLGGTVEDTELIDGLVFTEKLAGGNSPHRVEKAKIGLIQFCISPPKPNMDHQVIVSDYTVMDRVLREERAYLLNIVKVVKKAGCNVLLIQKSILRDAVSDLALHFLAKMKIMVIKDIERDDIEFISKSLGCRPIASLDHFVPEALGSAELVEEVTSAKYVKVTGVANP---KTVSLLLRGSNKLVLEEADRSIHDALCVVRCLVKKRALVPGGGAPEIELSLRLAERAREIEGLHSYCYRAFADALEIIPYTLAENAGLNPIQTVTELRNRHAQDKRTYGINVRRGCVTDILEENVLQPLLVSTSAITLAAECVRSILKIDDIVQTVR?????????????????????????????????????????????????????????????????????????????????????????????????????????????????????????????????????????????????????????????????????????????????????????????????????????????????????????????????????????????????????????????????????????????????????????????????????????????????????????????????????????????????????????????????????????????????????????????????????????????????????????????????-------MSDAEEDYELEYSEDDDSQPDVDLENQYYNSKALKEDDPQAALQSFQKVLDLEGGQKGDYGFKALKQMVKINFQLGEFKEMMSRYKQLLTYIRTAVTRNYSEKSINSILDYISTSKKMDLLQEFYEVTLEALRDAKNDRLWFKTNTKLGKLYLDREEWNRLARILRQLHLSCQNVDGSDDLRKGTQLLEIYALEIQMYTSQKNNKELKKLYEASLQIKSAIAHPLIMGVIRECGGKMHLREGEYNSAHTDFFEAFKNYDESGSPRRTTCLKYLVLASMLMQKEINVLDSQEAKPYKDDPEIVALTDLVDAYQAHDISRFESIVSPHKESIMKDAFIKEHIEQLLLNIRRQVLIRLIRPYTRITISFISRELNIPSAEVESLLVSCILDNTINGRIDQVKQVLELNP-SPTDRRYTAMEKWAAQVQLIQSTVVRKVAQLGTLNPVQILNQQAEEEKAENARLSSFVGAIAIGDLLKSTLGPKGMDKILLCETSRDSKVEVTNDGATILKAIGIDNPAAKVLVDISKTQDDEVGDGTTSVAVLAAQLLQEAEKLVGMRLHPQTIIAGWRKAVVAARAALEEFSQNRSNDEAQFRIDVLNIARTTLGSKILSQHKDFFAQLAVDAVMRLKGKSNLDAIHIIKKLGGSMLDSHLEQGFLLDKKPGLNQPKRVEKAQILIANTPMDSDKIKAGKLRDECFSRVVVAELEDAEKLKMKRKVDAILAHKCNVFINRQLIYNYPEQLFADAGVMAIEHADFDGIERLALVTGGEIVSTFTSPESVRLGTCDVIEEVMIGEDKLLKFSGVPLGEACTIVLRGATQQILDEAERSLHDALCVLAFVVKEKKICYGGGSAEMLMAAAVDSVAQTTPGKEALAIEAFARALRQLPTIIADNAGLDSAQLVSELRAAHANGQSTFGINIADAKIDDMEKLGVTEAFVVKRQVLLSASEAAEMILRVDSIIKDAPRKRVPDRSHMAASIKPSGKPQLLCRLDGHTDTVNQVVLIGDADAVISVSDDRTIRVWARRDTGQYWPSVCHTMPSLASAMDYDPQLRRLFVAMDNGSITEFELADDLNKITYRRSYIAHQQRVTSMKFSPVTEWLISAGKDKYFQWHCTETGRRLGAFLGSAWCTTVALDQASRHAFVGDYSGEITMLKLTETSYQPVTTLKGHSGSVQSLLWDERRRLLISGGFDQIIIVWDIGGGKGTAYELSGHRARITGLALYSASSLLSVSEDSTLVVWDVAAQRQETPEWTTRDCCERCARPFFWNLRARTSNSDVGSRQHHCRRCGRALCDACSENRSTLPRLGFEFPVRICNECHLHISDGDREPLAKFHDLKAPVTAMSLSAESKTMVTIGTDRSIKIWDLSKVLSKKALKKAQKEAEKAARKAAHK---AAGKENGSDDGSQGLYGQYPMIQSTEKLQREMIEISQCTLERADQMVWLRGRLHTSRAKSKQCFFVLRQQHYTLQCLLDVSEGTSKQMLKFISAVPKETIVDVEGKLVKSPLKIESCSQQEVELRIYQFWIVSLSDTRLPLQVEDASRPEPAEGDEEALKIRVNQDTRLDHRVLDLRTPANQAIFRLQAGVCHLFRESLNRRHFIEIHTPKIISAASEGGANVFEVTYFKGKAYLAQSPQLYKQMAIAADFDRVYTIGAVFRAEDSNTHRHLTEFVGLDLEMAFKYHYHEVLDTIAEMFVDIFKGLRDRYQPEIDTINKQYPSEPFKFLEPSLRLEYSEGVAMLRAAGVEMADDEDLSTPNEKLLGRLVKAKYDTDFYVLDKYPLAVRPFYTMPDPNNQMLSNSYDIFMRGEEIMSGAQRIHDPEYLTQRAKAHGIDISTIQAYIDSFRYGAPPHAGGGIGLERVVMLYFGLNNIRKTSMYPRDPKRLTPAMEEGLQLNEINPLEFSRVKMVYPKECRIRRISYRGKLDLTLWWMNGIKQEPIKRTCGEIPIMVKSLKCNLYGLDPEQLVERGEEMEEFGGYFVVNGNEKVIRLLIMQRRNYPIAMARNGWKNRGSMFSEFGVSLRSCRRDAQNMVLHYLTNGTVQVMITYRKEVYFVPAVLLLKALVNKSDYDIYRTLTQGCENDSFYKGCITNMLRLVQENILTQDAKEFIGDKFRWSSNADVCDQLLKRCVCVHLDSNEDKFNLMCFMVRKLFAVAKNKCALESADSTMNQELLLPGHIYLHILKEKIEGLLVGVKISIDKKMSNALDVTNAMNYFLSTGNLVSKTGLSLQQTSGFTILAEKLNFWRYLAHFRCVHRGSFFMEMRTTTVRKLLPEAWGFICPVHTPDGGPCGLLLHLSAMCEVVVQLDGRCIGWVLRYLKATGNIPPTLEICVIPRTEQNSLFPGLYIFSTPARMMRPVLNRTQTVEWIGTLEQVHMDICVIAEEATTHQELRETAMLSVLANLIPWPDFNQSPRNMYQCQMGKQTMGSPMHTFRYRADNKLYRLYPQSALVRPTSYDHFQMDEYPSGTNAVVAVISYTGYDMEDAIVLNKMSVERGFKAGVYKTETINLRVDVDGLPYIGSQGDPVCAYIQLKTVRYYSTEPAIVHEVKILGNLQQIQLTYLIRTPMIGDKFASRAGQKGICSALWPTESMPFTDSGMVPDIIFNPHGFPSRMTIGMVVESMAGKSAALHGYVHDASPFKFSEEYPSSAYFGELLQRAGYNYYGTERMYSGVDGREMEADIFFGVIYYQRLRHMVADKYQVRTTGPVDSLTRQPVKGRKRGGGIRFGEMERDSLLAHGTAFLLHDRLFNCSDKTLCATCGSIISIPIPYVFRYLAAE-MVSINMKIIRDTSRDLHRHQRNAPELHPMTVVREYTAAVNAAKLDRIFAKPFLCSLGHRDAVELLAKHPDRISGAVSASADGELRWWDLSNRKCVRALQAHDGPIRGLVGQDQTIKTILSKLAHHYNNMFATAGETVSLWEERNEPLRSFW-GVDTIYVIFSPIESLSSDRSIVLYDIREASPLRKVILEMRSNALAFNPMQAMHFTVANENYNLYTFDMRHLKKALQSHTDHVGAVLSVDYSPTGTEFVSGSYDKSVRIYRSREVYHTKRMQRVTSVMYSLDSKYILSASDEMNIRLWKAKASEQLGIQNYNETLLQRFQHHPQVKRIVRHRHIPKTLYQEKQTMVTARKRKMGMQFLQRGAYQDALSHYHAAIEGDDTNYQSFYWRATVYLALGKSKLAVEDLNRVIELKDDFLKAREQRGNILLKQGHLDEAHIDYEFVLRLEPDNPEAMIEELKNDVIHILQRVWNLKLRELRASCYESIGDIQSAITDLRPAIRSVPDNTGGYLHLAQLYYKHGDPDDSLTTIRECLKLDPDHKECYKNVKKLAKSMQEECVEKMVGLIKTKCQCASKGGSAVQICTEALALDPILCDRGEAYINQDDFKQDFAAARELDQRAGEGLKRAQKLEKSRGKRDYYKILASKREIAKAYRKLAAEWHPDQY-QGID--KKNAEKKFIDIAAAKEVLTDPDKRAKFDRGEDPLDPESGFHYTFPFMLVLFESPAGYAVFKVLDEKKVQKTDNLFKEFEDASGAAKILKLKHFQKFQDMTQALSAATAAIEGKLCKPLKKVLKKLAVSDAHETLAVADAKLGNIIKEKMDILCVANSSIQELMRCIRSQQEALITGLSQREATAMALGLAHSLSRYKLKFSPDKVDTMIIQAVSLLDDLDKELNNYVMRCKEWYGWHFPEMSKVVTDNMLYVKTVRKMGMRSNAINLDLSDILPEDQEAKIKELAEVSMGTEIAPDDVANIMHLCDEVIQMTEYRGTLYEYLKNRMTAVAPNLTVLVGELVGARLIAHAGSLLNLSKQPASTVQILGAEKALFRALKTKHDTPKYGLIYHAQMVGQSSQKCKGKASRWLAAKSALAIRVDALGEDTDTEMSLRNRANLEARLKMLEDGKLTRISLFLWPKNSTKLQFSIFVCFGIMIVGRVCTPIAPIMQKKIVDGLVSSTGLLL---LFQGANSLMANVRSYLWLGVQQYTTKATQVSLYAHLHSLSISWHLSRKTGEVLKVLDRGTSGVQNLCSYLLFQIFPALTDIVIAFGYFTYAFNWFALVAFVCMGSIVLTEWRTKFRHEMNHLENAAYAKSVDALLNFETVKYYNAEEIEIAKYQNAEWKSQSSLALLNIIQGSTSSIIGALLCAYITAGDYVLFIAYNAQLYAPLTFLGTYYRMIQQSFTDMENMFELLDVVDHHLKL--EIEFRDVCFRTVLKHISFVVPHKHTVALVGHTGSGKSTILRLLLRFYDVQSGSILIDGQNISAVSLRSHIGVVPQDTVLFNMSIRENIRYGRPSEDVEAAAAAADLHHSIFQMYETVVGERGLKLSGGEKQRVAIARTILKAPSIIVLDEATSALDTQTERNVQKALNVLENRTSIVIAHRLSTIINANQIIVLEHGEIVEQGHEELLSKKYASMWRQQQALNVPETRVTTLSNAVRVASEDTGAPTATVGIWIDAGSRYETEKTNGVAHFLEHMAFKGTGKRSQTDLELEVENAGMHLNAYTSREQTVYYAKCLKKDLARAVDIIADITQNPKLGEQEIERERGVILREMEEVEGNLQEVVFDHLHSIAYQGTPLGLTILGPTENIKSLQRQDLKDYIDTHYKGSRIVLAGAGGVDHDELVKIAEQTFGKVSNSMDSQ--APCRYTGSDIRVRDDDMPFAHIAIAVEGAGWANADNIPLMVANTMIGSWDRSHGGGANASSRLAAWAQSVKSMHSFQSFNTCYKDTGLWGLYFVADGDELDDIMIAVQEEWMRICTEATDGDVTRAKNLLKTNLLLQLDGTTPLCEDIGRQMLCYGRRIPLHELEARIDAIDADTIRNVCQTYIYDRCPVVAAVGPVEGLTEYTRIRGQMYKHSVHTLVFRSLKRSHDMFICEEGALPPIDEKAHKLRVGTKGRDEYGPVMHLVSEGRRSVSSGPLVLAGQQLTAQLKKPSIPKPTWHPPWKLYRVISGHTGWVRCVAFDPTNEWFCTGSNDRIIKIWDLASGKLKLSLTGHISGVRGLAVSQHHPYLFSCGEDKQVKCWDLEQNKVIRHYHGHLSGVYTIGLHPTIDVIITGGRDSTARVWDMRTKANIHVLSGHTNTVASVLVQATEPQVVSGSHDSTIRLWDIVAGKTRVTLTHHKKSVRALVLHPKLNMFASGAPDNIKQWMCPDGKFIQNLSGHNTIVNCLAMNEDGVLVSGGDNGSLQFWDWKTGYNFQKLTTPVQPGSIDSEAGIFAMSFDLSGTRLVTCEADKTIKIFKEDETATEETHPINWRPEIVTGSTSEYLINCSAEHYLEILGINVKAKNFLVFQGAVESIAMKNPKERTVLFEEISRSMISAEEETYQKKKGIAAERKEAQIEEAEKYQKLKEDVQVNLHLFRLFHQEEELQKKRRKDKIEAELKEKKKVEQTHRDDVELNKRKPAYIKAKEKTAHMQKKLDAAKKSLEAATKTHKSHQGIEELEHELSQVEEAYEQEDVSLEESQVKEYNRLKEKAGKMASAALQEYDSVAREQKTDQDHLDNELRKRNECEAKLKELEENQRRINKLVDLQDLKEEEKKLEAKKRLTQKFEDVASLGDAKVDKHEDARRKRKSEIVEHFKKLYPGVHDRLVNLCHPIHKKYNVALTKVLGRNMEAIVVDTEKTGRACIQYLKEQMLEAETFLPLDYIDFKPLKERLREFKDVPNVKLLYDVLKYEPLSIKKAVLYATNNALVCETAEDAAKVAFQSPDGKRYDAVALDGTYYQKNGFISGGSSDLAKRAKRWDDKDFHKLKDQKEKLQEDLREAMKTARKESDLTTIESQIKGLETRIKYSKVKQLEQSMREREGRINEIKARQNTVEDDVFRDFCEQIGVANIREYEERELHASQEREQRAELENQKNRIASHLEYER--TKDTLAVEQDQQELGRLKEIEQKQKELIEQQMEAISTLKNERQSKKIRVDEIDEEVAEIRKRLTAQQKEVTGVQKTVTQAEAKLEQKRSERHTLLQSCKLEGIRIPLIRGSMSQMYAREAMEIDYNQLRMEKEINLQRIQAPNFKAMEKLDSVKERLKDTDTEFEHARRKAKSNFELVKRERTCFEHVSNCIDEIYKSLTNNPSAQAFLGPENPEEPYLEGINYNCVAPGKRFQPMSNLSGGEKTVAALALLFAIHSYQPAPFFVLDEIDAALDNTNIGKVARFIREKTQTSFQCIVISLKEEFYGHADCLVGICPDPGECTISRIYTIDLSMSEAMLLYLGQAIREGNLAEMQNIYERSLVNLMDQYLIIIYKE--LYYRQIYADDRFSSYYNYVNLFNYILSSGPVSLDLPNQWLWEIMDDFVYQF--QSFSTFMWNVHSVLNVLHSLVQKSNINQQLEVFSKGGNPDEVAGEYGSRPLYKMLGYFSLIALLRLHSQLGDYYQAIKVLQHLELNRKGLSRVPACQVSTYYYVGFAYMMMRRYEDAIRTFSDVLVYIGRTKRNYQLRQMNNQMEKMYALLSICMVLHPQRMDESVLQQLKLKHMMKMSQGDLDTFERCPRFLSPVQWRAFRGEVVAQMNMRSFLKLYTTMPVEKLTKFVLMCFKHLMSNVVLDGEFQTGSDMDFFIDKDMIHIADTKLARKYGEFFAKQYNRFEEWYEMGPPDAILGVTEAYKKDPNPKKMNLGVGAYRDDDGKPFVLPSVRAAERQLMSKNLDKEYLPIGGLNDFCKNAAILALGDNSAVIKEDRNATVQGISGTGSLRIGAMFLDEFLKGNKTVYMPNPTWGNHIPLFKRCNFQVKQYRYYDPKTCGLDFQGALEDISSIPEGSVILLHACAHNPTGVDPRPEQWTEIEKVVRERNLFPFLDMAYQGFATGDIDRDASAVRLFAGSG-PMCLAQSFAKNMGLYGERVGAFSLICSSAEEQARCMSQIKILIRPLYSNPAVNGARIANLILSDPQLRAQWLKDVKGMADRIITMRSRLRSGLKREGSTHDWKHITEQIGMFCFTGMTAEQVTRLIKDYSVYLTKDGRISVAGISSHNVDYLARAMHEVTKHIRPSFYHALHCILMAVLMYVFVEFSKPPDQVDPFSEYGVIFTIILYLFRLLPLLALPQSLTNLFGLTLYNAFPPRVRLKVKPHEAPFLCIRVVTRGDYPGLVRENVKRNLATCLDTGIDNFVIEVVTDKEVYVTANSKIRQTVVPKSYNTTTGAMFKARALQYCLEDNVNLLADGDYILHLDEETLLTKDALRGVLNFISAGRCSFGQGLITYANERIVNWFTTSADMYRVADDLGKLRFQFNFFHKPLFSWKGSYVCTRVGAEREVSFDHGPDGSVAEDCYFSMVAFSKGYSFEFIEGALWEKSPFTISDLIQQRKRWMQGIYLVVHSAKIPWRYKVWLSCSLYAWATMPLSTSNLVLAPNFPLPCPQTFNIICAFIGALNIYMYIFGLIKSFSISRYGFFGFWLCFMLVVIAIPLNIVVENVAVVWGLLGNKHKFYPWPHYHFSGKLRPGRISTKRTVPAHIKRPDYADHPEGIPVSEQAMKGA-EIKVLNEAEQEAVRKASLLARECLDVALAAAKPGVTTDELDRLVHEAAIARNCYPSPLNYYKFPKSCCTSVNEVICHGIPDDRPLKDGDILNVDVTVYHNGYHGDLNETIFIGKVDEAAKKLVRVTYESLQKAIECCRPGVLYREIGKIIQKHVQQNGFSVVKSYCGHGIHSLFHTAPSVPHYAKNKAVGVMKAGHCFTIEPMISEGVWQDEVWPDNWTAVTTDGKRSAQFEQTLLVTDTGVDILTRRRKKNGQPWFMDQSTLRECLKERSAKRRELLARQLGAGCAENLGLLLGNDKTTATEQGVTLDDEEVMAYRDSSTFLKGTQSANPHNDYCQHFVDTGQRPQNFIRDVGIQDRFEEYPKLKELIKLKDELIHETATPPMYLKCDLLQYNLRELNGKFDVILIEPPLEEYQRSCGVTNTRFWSWEEIMKLEIEEVAAPRSFVFLWCGSSDGLDLGRQCLRKWGFRRCEDICWIKTNINNSKVKNVEPRAVFQRTKEHCLMGIKGTVRRSTDGDFIHANVDIDLIISEEPPFGMMEKPEEIFHIIEHFCLGRRRLHLFGRDLTIRPGWLTLGPELTNSNLNTEAYNAHFNTANDYLTGCTERIEALRPKSPPPKGKRVVMRVDFNVPLKDGKITNNQRILAALPSIKYCLDKGAKSVVLMSHLGRPDGQSNSKYSLAVVAEELNQLLGKKVIFLNDCCGAQIEAACADPTPGSVILLENLRFHIEEEGKGVDTAGNKIKADASKVKEFRASLTRLGDVYVNDAFGTAHRAHSSMVGVELPRRAAGFLMKKELDYFSKALDQPARPFLAILGGAKVKDKIQLIENLLDKVNEMIIGGGMAYTFLKVTRGMKIGDSLFDDDGAAIVEKLMAKAASNNVQIHLPSDFVIADKFHEDATTGTADISNGIPDGWMGLDCGPKSVELFAGAVSRAKTILWNGPAGVFEFEKFAVGTKGLMDVVVAATDRGAVTIICGGDTATCAAKWGTEDKVSHVSTGGGASLELLEGKILPGVAALSDAMRTFGDRPVSFQLEDGGDYYYIGTEVGNYLRLFRGTLYKKYPSLWRRAVTVDERKKISQMNMSQHSAANFISLLKKSEVDDLIDGNEEKYRAAPVQENEGGGHGAKNARPSFMPAAPNNAHHLDAVPCSTPINRNRLQHKKNKSFPMLYDDLDPAMLHESAALPECLVPIRLDMEIEGSKLRDTFTWNRHEAHISPEQFAELLCDDLDLPPLLFVPQIAASMRQQIEAFPSESLLDEQTDQRVLIKLNIHVGNISLVDQFEWDMSERANSPEEFATKLCSDLGLGGEFVTAIAYSIRGQLAWHQRTYAFSEAPLAQLEMPFRAQSEAEQWC---------------------------------MGANGSTLDRDDALPSSERYLGLVNFGNTCYCNSVLQALYYCKPFREKVLEYKAKNKRTRETLLTCLADLFHNIHSHKKKTGTLAPKKFIARLRKDNEVFDNYLQQDAHEFLNYLLNTIGDLLQAESSWVHDIFQGTLVNETRCLTCETVSSKDEDFLDLSVDISPNTSISHCLRGFSSTETLRGEHKYHCEQCNSKQEAQKSLKVKKLPPILALHLKRFKYTEQQNRNTKLSWRVVFPLELRLFNTSDDALNGDRLYDLVAIVVHCGTGPNRGHYISIVKSHGLWLLFDDDMVDKIDPSTIDDFFGLTQDTPKSSESGYILFYQSKENADIDNWIELAKQCKYLPEADLKKLCNMVCQILIEENNVQPVSSPVTVCGDIHGQFYDLEELFRCGGHVPDTNYVFMGDFVDRGYYSLETFTRLLTLKAKYPKKMTLLRGNHESRQITQVYGFYDECQQKYGNANAWKYCCKVFDLLTLAAIIDGEIFCVHGGLSPEIKALDQIRTIQRNQEIPHKGAFCDLVWSDPDEVETWSCSPRGAGWLFGAKATHEFMTYNSLSLICRAHQLVHEGYKYMFEDKLVTVWSAPNYCYRCGNVAAVLEISNDQKKNPKIFNAVPDHERVIPERHAPYFLVDSASPEETQDAEAATIDELKEHTAHIQKAVAQKESRFILRILRLLPATRKKLNSKLLRKTINGFYTHDKMHREVLLSFVDAEDADTDAAQKSAHLALLPEVDVYLHLLLLVHMIDAANMERAIRCAELLKGKVEAHSRRSMDLLAAKTYFYYSRVYELDGNLSSIRGFLLKRLRTATLRSDFEGQAVLINCLMRNYLHYSLFKQAAKLVSKVTFPEMASNNEWARYLYYLGCIKAIQLYYTDAHKNLLQAIRKAPQHSALGFKQTVYKLAVTVELLLGDIPDRTTFRQPALRKSLAPYFQLTQAVRTGNLGLFNQVLESYGARFQADHTYTLIIRLRHNVIKTGVRMINLSYQRISLADVAAKLQLGSAEDAEFIVAKAIRDGVIEATIDHDKGYVQSAENIDVYCTGEPQSQFDQRISFCLDIHNQSIKAMRFPPKSYNKDLESAEERREREQQDMEYAKEEDDDTFQVAVRLTLALAAMAARTLWQSAVTDMIENFRDSQPLLFEFLARLPEEATAGYIYYTSVLSLCQSALASNLRITAMRTVPANLCITLL-DLTDDHACDAILSFLQHPDGHYPKLMGDLLEQVVKCGPIVETKRACGDQDSIYSLLTGMGEMHTNLILASLLPDSSNRTEMLLKMLLDCVGTPGQYPSEEIISRIPITFWHILLDELARVEPSTQMAKQLQPVYEQLVKMLRKSQLPDPGTMDLDEKEDLRCYRQDIAD--CYMYIATMLPAPVFYFFISALDTAKNAKVIEACLFALNAIGDMADSEDDSPVVGAVLALLPRIPAGDEVLSQVMTAVGIFAEENIGPLVHLLLRGLQQTSASASMALKDLARTHGDRLAPAANDILQAIAVLKHRDRVRLVAIVGHVVSALSSEQALTSLSALMAPFVQQLNEITNLVEQLMPLFKLIAAKY-SCDAEVVSNLAECIRKAVPVLELEVVLSELLSMCGALLLGAATSVDLHGCTDAVESFYKLAVLFKKFSLDDLIQLCPEQFTYRALTQFVNASERSPVKEALENIIAQLVQNIRVSRNFIEAEADVFLALNKTAAALQRFVSSPESRQNFARQILRERSNKRMLCKVI--IDFTLKVKVKWKEVY-DVEVHLPEVFRAQLFALTGVLPERQKVMFKGAILKDS-WGATILMMGTKEELPQPTEKTVFMEDMDDSEISTALKLPTGLNNLGNTCYMNAVVQCFKTVPELTDLAKFTGTITGALRDLYRSMESYSTAPIVLLQALHTMFPRFAEKGEHGQQDANECWTEMMRMLQNLIDQLFGGKLVALQCTESEEESTEDFLQLSCFIS-NEVKYLIAGLKLRM-QETITKMSPTLNRDASYKTSKISRLPAYLTINLVRFYYKERESVNAKILKNVVFPMMLDVYELCSTDLQQKLSPQREKFKKWDNEFSFSEDGSNNSGFYQLQAVLTHKGRSSSSGHYVGWVRREWFKCDDDVVVSEEEILKLSGGGDWHVAYVLLYGPRVLAGLINEKLPKELLLKIFSFLDIVSLCRCAQVSKEWNVLAMDGSNWQNIDLFSFQRDVTYDVVSYIAQRCGGFLRRISLRGCQNVPDQALSVFAQYCHNIEQVLTNCHKLSDDSVVSLSMSLHVDSCVELTDRSLRFF----NRLRVIDISWCRKITGQGIGTIAG--DQLLRFTAKGCLDNEAIIKLATKLQVLNLQCCSFLTDSAVIAVAQNCPDLRHLCVSGCSLLTDASPQALAGGCLHTLEMANCQRCGDAGLAPLLKACHDLRRLDLEECNLITDSTLNHVAAFCPLMEQLTLSHCDQITDQGVHKLAVQCIEIDNCPFISDTSLEYLADHLRRVELYDCQLITQDAIGKFQPEVRLHTYFAPATPRQRYCRCCVIVSSKAARVFCPDHPGANLIEDYRAGDMICPQCGLVVGDRIVDVGTEWRVFQNEKSSNDPTRVGAAENPLLGGSDLSTIIGRTGDASDESGNAKYANRKTMSASDRALIGAFREISAMGDRINLPKTIMDRSNLLFKQVHDGRSLKGRSNDAIASACLYIACRQEGVPRTFKEICAVSKVSKKEIGRCFKLILKALETSVELITTGDFMSRFCSNLALPPSVQKAATHIARKAVEMDIVAGRSPISVAAAAIYMASQASAEKKSQKDIGDIAGVAEVTIRQSYKQMYPKAAQLFPEDFKPANESPWVEKYRPEKFTEIVGNEETVARLEVFSRQGNMPNIILCGPPGVGKTTTILCLARLLLGNSFKDAVLELNASNDRGIDVVRNKIKMFAQTKI---------IILDEADSMTEGAQQALRRTMENYSKTTRFALACNTSDKIIEPIQSRCAVVRFGKLNDAQILAKVIDVCRKENISYTEDGLEAIVYTAQGDMRQAIGNLQSTHVGFGHVNAKNVFKVCDEPHPLIIKEIIEFCSKGDIDEAYTRMKTLYSLGYAAEDIISNMFRVTKSHGLAEFVKLEFVKQIGLTHMTILQGLGSLLQLSSLLANLCLIVQDKKYMKKEHSLVKPYQGSGMNMPNWDFTGTTMVSSNYIRLTRDSQSQQGSIWNKVPWEIQIQFKVYGSGKDLYGDGFAIWYTKDPLQPGPVFGSRDFQGLGIFLDTYANQNGHHNHGHPYISAMVNNGSLSYDHDRDGTHTELAGCEAKFRNSEYDTSVSIRYEHDTLVVSTDIMGKKEWKECFRVSGVRLPTKYHFGVSAATGDLSDNHDVIGIKVFELDEAREFILPQAAPHRDHIDDAMSGTKFFFVVLFSMLFLMFYFYQKHQENARKRFYRDRDAFDEIRDKNCRIQDINELFLPNDTVTHVPNIKQLNIDPVFPNRTNLLHIHNMAISKAFFFSFILQRAKDDEPGFMYYFMSVIADVAANRFINASAIYYAPNMSFTPSYKGFFNKTMPLFAPRAFRSDDFNDPYHLEGTSTLNTIEAIDLGAISLNYSSDQYRINEWYSAWLPDLTKRQDSKTTYTVQITGNNDTFVWHGPPAGNDNPGPVKWVRPYFDCTRSDKWVYGATSPIPDIYPRHTQWRHIEIPRYVAVSVMELDFERIDINQCPFGPGN-PRPNYFAGTSRCKNDTTDCEPVHGYGFRRGGYQCRCKPGFRRPRVVRNPYHGELIERASKHEYENGYQCDKIGYIAVLTQTLNNYMAIGTRIDPLLGGDVVYGKEVQLENEARMAVRLANFVSGFLQIVDPKDLFAEFRVPDKSLTADQMIGEVMSIVIGDQKVVGAGIYFDYKAFFGPY--AWRKGRNERKYFVDDTTKIRIRYNSTGIKYDHFPLQYKAADVGYWTSPYFDCGGYHNSWMVTYAAPFFGWDSLRARLQFKGVVAVIELEQLEINQCNAFENTHKCDRRSSRCVPILGRGFQGGYKCECNQGYEYPYNDPITYFDGQIVEAPSRFERMTHVSGTDKSKYGGRFMVTALPGDGIGPELIGYVKEVFRYGGVPVDFEEVHLDSSRDDVDLLEQAIIAVKRNGVALKGNIETRHNDPNCKSRNVELRLRLGLFANIVHVTSQPGIETRHSGIDIVLIRQNTEGEYSCEEHMSIKGVVESLKVITQSKSDEIARYAFEWAKNNGRKKITCVHKANIMKLSDGLFLRCCTEISKEYPELEFDNIIIDNCSMQLVSNPKQFDVLLLPNLYGNILTNLACGITGGPGIASGRNYGREYAVFETGTRNTGKSIAGKNIANPIAMMNAGVDLLYHLNLREHAEVIATAIDKTINVDKIHTPDLGGQATTTDVVQNIIKEVQKHAMTYSGSSNEVILRDPPTDGISAVKFGQTSNQFLVASSWDGFIRLYDIQGERCRAKFDLGGPVLDTCFQGSSHVWSAGVHRSVRLFDINQGTELKAGSHEDTVRCIEYASDVSQIVSGGWDGAVKLWDPRKPIGPSSHSQDNKVYAIAIAGERIIVGTANRKVLIWDLRNMAFVLQKRDSSLKFQTRAIKAFPDKTGYVLSSIEGRVAVEYLDPSPEAQKKKYAFKCHRMKDSTKIEHIYPVNAIAFHTVHGTFATGGSDGFVNVWDGRNKKRLCQFHKFPTSISSLAFSPDGSALAIAASFQHEYRLEQNPPADQIYIRHSCLDQLKELTIVVADTGDFEAMKEYKPTDATTNPSLILQAAKLTQYQALIDEAVSYGRGPCEQLEEAMDKLFVLFGNEILKIIPGRVSTEVDARLSFDKDASINKAVKLISLYKELGVPKERVLIKLASTWEGIEAARILEKEHGIHCNMTLLFNFTQAIACAEAGVTLISPFVGRILDWYTANTDKKAFEPLEDPGVKSVTRIYNYYKKFGYKTVVMGASFRNTGEVKALAGCDLLTISPGLLKELANSNELVPHYLKAENATSIELEKISVDEKRFRWDMNEDQMATDKLSDGIRKFAADARKLEALIQEKLKMGDCVCRPLATVYAEDNEQSGTNVSMPAVFRVPIRPDLISFVHHQLLKNKRTPYAVSKEAGHQTSAESWGTGRAVARIPRVRGGGTHRAGQGAFGNMCRGGRMFAPTKTWRRWHRRVNVTQRRHAAASAISASGVTALVMAKGHAIERVNEVPLVVSDKVQEYKKTKQAVALLKSVKAWDDVEKVYKSKRQRPGKGKKRNRRYKKKCGPLVIYEKDNGIVRAFRNIPGVDTCDVNALSLFKLAPGGHAGRFIVWTESAFRKLNDIFGTFTKTSKVKKGYKLPRPMMTITDLGRLFKSEEIRGALRPKKSIIISKKNKPNPLKKIHLLGRLNPYALVEKRKTILAQQEQKSQRIQKKAALEKVAALKKKRLQSPFPKNRKLNQGAVLKKMLDAIKDLINEGNYDDIVSIYLHQAMDNSHVSLVALNLRADGFDKFRCDRNLSMGMNLTSMAKILKCAENNDIITLKAQDDADTVTFVFESQNQDKVSDFEMKLMNLDSEHLGIPETDYSVVVKMPSAEFQRICRDLSQIGDSVQLTCTKDGIRFSASGDLGTGNIQLSQTADVEKEEEAVIIDMQEAVTLTFALKYLNSFTKATPLSTQVCLSMSADVPLVVEYNMGHLRFYLAPKIDDSERNNVKVAVLGASGGIGQPLSLLLKQHPGISYLSLYDIAHTPGVAADLSHINTGSKVKGFVGNDQLKAALEGIEIVVIPAGVPRKPGMTRDDLFNTNASIVRDLADACAQTCPKAMLAIISNPVNSTVPIASETFKKRGVYDPKRIFGVTTLDVVRANTFIAEAKGLDPVSMSVPVVGGHAGITIIPLVSQASPKVDFPQDQLEKLTKRIQEAGTEVVQAKAGAGSATLSMAFAGARFVFSLVSAIQGKNIVECAYVKSDIGDAGFFSTPLLLGKNGMEKNLGLGKLSKFEEKMVTDAMDELKKSVKKGIDFANKEYEWLLKFEVNDIVEQLVIAECSKRFPL-ARSDKFIM-QSKVVATLTGDSISHADISLRL-PKHSQRTIVQNDAQWKLQQIQDAGNHLMQAMNLLRFKFTSGQEVRNLMSDVMTCVGRGRACLVVPKKRTIEEIMQSRNMKSLQPPLPNDVAVSFYIQSYKLVFAVYHV-QKDPQKF-DAECSVPWLSEVLVLFTVALQLCQQLKDKVEVFQYNDFLPMFIPLFLEFNAPISEISDLLGVCDVCFKDAESVL--NGFVSILVCLFCERLESATVQPVAFN--VLYNFYEGLRVDVFCSLLRVAAGSVPEVFQDVSVTKKWLPVDKARQVYRNIHSALSDMPLRVMVELLSTYQDHDAAEANQDAIKCIAFAISDPNTYLMDHLIPLKPIKALENQPINELLKIFVYGKLSEYREFYRKHKEVV-EQLGLDHEKNVEKMRYLTFMYLAEKNQEISFDDIKREVEI--DDVEGFTINVLRTKLVTAKVNQPNQKVIVVSTMHRSFMKNEWEQLREILRLEKVEQSTQQFLEYLG-TR-RKKEVEELAEEAKAIDEGEERAVIEESPQFKELVNILVEWINDELAKHRII--VKNIEEDLYDGQILHKLLEQLTNSRIDVVEMTQNEEGQREKLKVVLERASQALGLKWSVDAVHSKNVVAIVHLLVALARHFRAPVRARLPENVVVSVVSVTKR-EQLTRTYDEYGMKVE-RDAFDQLFDHAPDKLTVVKKSLLTFVNKHLNKINIKDLD-KQFHDGFYLALLMGLLEGYFIPLYLTLTP----------------------NNKVNNVAFAFGLMKDAGLKPKARPEDIVNYDLKSTLRVLYNIFMQLSHRSTRIIKDAERDLRELLSVPPTYKVLFMQGGGTGQFAAIPLNLCPADYLVTGTWSRKAAEEAKQYVKYTRVPPASEWNLSPDAAYFYYCDNETIHGVEFPIVCDMSSNILTRPVDISKFGVIFAGAQKNLGPAGVTIVIVREDLVAVASVCPSILAYKVFAENDSLYHTPPTYAIYLLLVLKWIKSEGGVSGMARSAAKSQAIYDLIDQSGGFYCRSRTNIPFRI----RSNDALEKKFLKEAEMIQLKGHRSIGGIRASVFNAMDVVQAMKLYRERPEWQDVTPVLKISYTNTFRDCFGYLRAVISGELSERVFELTTTCADENPACYTVWVLRRRLLEHLKKDLNEEMEFMSRQIFDNQKNYQVWYHRQRLVQLRELDFIERVLDAKNYHAWQYRQWLLKTFNLWSKELNFCSSMLNEDIRNNSAWNQRYFVLKNTTGFVVEEEIKFTLDKISCNESAWNYLGGILNIYLYATLLSEALEIDIPRSPYWTFKQKLLAIIIDRFEELLEFVPHLSRFLTAQGVAYRFIVINQGDRFRFNRGALINIGYLVSRAQCDYMVMHDVDLLPLNPKLSYRFPQGDNVHIAAPHLHPKYHYSTFVGGILLMRHAVFARLNGLSNKYWGWGLEDDEFYVRAKEANVRFERPTDIGTGINDTFRHIHDARRRPRDMVRIGSQREESRKRDRVTGLNNVVFQHQGLYQVRIGEVPVDVHNVHLHCDLTITPWCEKPRMSPGETITTDGYMRGHGGLLSAVAGVVEKVNKLITVRPLKTRYNPEVGDVVVGRIVQVC--QKLWKVDVGARLHAALHLHSVNLPGGELRRKSIEDELMMSQYFIDGDLVSAEVQNVGVDGAVSLHTRNLKYGKLGQGALVTVSPSLIKRCKHLHNLA-NGVHLIIGLNGFIWVTSSKFTREAICRTRNVILCLAIHNIMLYDTSIVHAYDIS-VAQLTRHRADKLVGSWLLTCSGMALGAVFLGGLTRLTKSGLSMVDWHPLNEGRPRTAEWEAEFAKYQQFPEYKVRNKDMTLEQFKSIYWMEYIHRMWGRTVGAAFYIPAAAFWARGYFNRGMKKQIIYLGTLLAAQGLMGWYMVRSGLEEKPRVSNLRLAVHLGAAFFFYGLLFRAALHRLIPSKPLVRFSWATGLVFTTALSGALVAGIEAGLVYNSFPKMADSWIPSDILAFPKWKNFVENPTTVQFDHRLLGETVLTALYIYSRKVPLPPRARLATHAMLAAWLQVGLGITTLLTYVPTPVAVSHQVGALTLLTTLLWLTHELKLIRR-LERYGIDD------------------VTEKNACQWSKDKLHSLFSDLEIND-SIMSVIKELKKCEGEATANNRKAKLIFFYEWELELCAGS-VVKGRVEIPNLSDENDIHEVSVNVTLVDEKIKGMMRSKGTEVIRSKLDEYVSSLKMDFSQGLILPTKDSGANSTATKGKKISTKELIMEDFKCRAEELYRAFTMVQAFTKGAAGGHFQMLDTNVSGKFLKLNELEFEWRFKSWPAEHYSFVITIEEVN---------------------------------------MDPETFLEIANHVSKLKMYPYFELAHCIVTLLYLREDLGTGSQLFSRKHPLSCWVSSMFSIYAGGIFAALLLGEPVLAVLKSNQSLILATACWYLIFYSPFDIVYKFCKILPIKLAIALAKEVTRAKKVHDGVHHAAKIYPSAYIIMVIIGVVKGNGTSFLKVFERLLRGFWTPQAMEIMQPSFATKACVIASLVFVVDKKTDLISAPHSLVYFGVVVFFVYFKLSSVVLGLHDPFIPFENLACAIFFGGIWDAISRAIKDTAAKKKEMFRPDGIVQRRTADGCDKDKETRLTLMEEVLLLGLKEKEGYTSFWNDCISSGLRGCILVELGIRGRIDLEAAGMRRKSLLMRKVVVKNDAPVGDVILDEALKHIKETATPETLQNWVDYLSGETWNPLKLRYQLRNVRERLAKGLVEKGILTTEQQNFLLFNMTTHPLVDQNVKDKLIKRVQDSVLAKWVNDVHRMERRHLALLLLSHASDVLENAFNPLSDEEYEMAMRRVRDLLDMDFEAECAKSQTCDIMWGVFAAFVKMADGVPTFKCVLVGDGGTGKTTFVKRHLTGEFEKKYVATLGVEVHPIVFHTNRGAIRFNVWDTAGQEKFGGLRDGYYIQAHCAIMMFDVTSRITYKNVPNWHRDLVRVCENIPIVLVGNKVDVKDRKVKAKSIVFHRKKNLQYYDISAKSNYNFEKPFLWLARKLIGDANLEFVAMPALAPPEVQMDPEWQSKLENDMKEAQNIVLPDEEDDDLLAAQITPILRESKFRESGMLTPEEFVLAGDHLVATCPTWAWAKG-DKSYLPEDKQFLVTKNVPCSKRCRDMEEKIIEGD-EGWVDTHDEDNEDDEGVDMDDFLDEDLETAEILATRTYDLNITYDNYYRTPRLWLTGYDEKMKPLTTEQIYEDISQDFVKKTVTVENHPHIEGVPQASVHPCRHAQAMKNLIQTVEEGGGLEVHMYLIVFLKFVQAVIPTIEYDYTANFNMLSNLERKLPYDKLSANIDIVKKRLNRPFTLSEKVLYSHLDQPQSEEIVRGTSYLKLRPDRVAMQDATAQMAMLQFISSGLPKVAVPSTIHCDHLIEAQLGGDKDLSRAKDLNKEVYNFLATAGSKYGVGFWKPGSGIIHQIILENYAFPGLLMIGTDSHTPNGGGLGGLCVGVGGADAVDVMAGLPWELKCPKVIGVHLTGKMSGWTSSKDVITKLAGILTVKGGTGAIVEYFGPGVQSISCTGMGTICNMGAEIGATTSVFPFNSRMADYLASTNRRAIADAAEQVKDLLSADAGCKYDQVIEINLDSLEPHVNGPFTPDAAHPISKLGQTAKEKGWPLDVKVGLIGSCTNSSYEDMSRSAMLAKQALDHGVKSKSLFTVTPGSEQIRATIERDGQAKVLKEFGGMVLANACGPCIGQWDRKDIKKGEKNTIVTSYNRNFTSRNDANPQTHAFVTSPEMVTALAIAGRLDFNPLTDELTGSDGKKFKLKAPVGDELPRAGFDPGQDTYQAPPADGSNVKVDVDPKSQRLQLLSPFSKWDGKDLIDMVVLLKAKGKCTTDHISAAGPWLKYRGHLDNISNNMFIGAIPEESGEANKIQNRVSGSWGTVPEIARDYKAKGQPWVVIGDENYGEGSSREHAALEPRHLGGRAVIVKSFARIHETNLKKQGLLPLTFADPSDYDKIKSDDKISIVGLNGFAPGMESRGGMFKNTFQSGFLSILYSLGSKPLQIWDKKVRNGHIKRITDNDIQSLVLEIVGSNVSTTFITCPADPRETLGIRLPYIILIVKNLKKYFTFEVQILDDKNIKRRFRASNFQSTTRVKPFICTMPMRLDEGWNQIQFNLADFTRRAYGTNYVQTLRVQIHANCRLRRVYFADRLYAEDELPAEFKLYLPVQ------SYQYMQKARADIKRVTQQYRGLAETFIFNNGSSKELVCLDGTIPVRYKYNIPIRIWVLDIHPYHAPFCYVCPTPTMQIKTSQYVDESGRVYLPYLHDWNRNS-SDLIGVIQVMIMIFGEQPPVFSKSPNPYPSSNTLSITDQHIRISLLSAVESRITDRALEKSKAEEEVLRKTNEELQAGKQKLDRYMSDMERDREMESEKSEQLKQLDVDNAVTPTAPLYRQLLQAYAEESAVEDAIYYLGEGLRKGVIDLDTFLKHVREQSRKQFMLRALMQKCRQKAGLPMGRKFYVGGNWKLNGTKQSIQVICDRLKTSQSETEVCVGVPAPYIQFVRDLLPPSIHVAGQNCYKASSGAFTGELSVDMIRDCGGDSVILGHSERRNVFGENDQLIAEKCAFALQGGLVVIACIGELLEEREAGKTEEVVFRQTKAYADLIKDWKNVVIAYEPVWAIGTGKTATPEQAQEVHAKLREWLSKNVSEEVGLNIRIIYGGSVTAANCKELAQKPDVDGFLVGGASLKPEFVDIVNAKKMATALSKKRRFVADGVFNAELNEFLRRELAENGYSGVEVRNGAMKTDIIIMATRTQDVLGEKGRKIRELTAVVQKRFNFKEGTVNLFAEKVSARGLCAITQCESLRYKLIGGLAVRRACYSVLRCIMEAEAMGCEVVVSGKLRGQRAKSMKFVEGLMIHSGDPTNHYVETAVRHVLLKQGVLGIKVKIMHPFDAQGKRGPALMLPDKVTVMVPKYEDDNVETRSDNKDNAASKIEQLKQWSLSTYKCTRQILAEKMGKGTRTVDGELEANIELLRETHQKYLNILRLAKLLTTHFNTVATQAALGECFSDLAQK---ELQQEFLYNAETQKNLSKNGDTLLGALNFFVSSLSTLCNKTIEDTLITIRHYENARLEFDAYRCEARANFKEKYERLRGDVQIKMKFLHENKVKVMHKQLLLLHNAVSAY-FSGNQSSLEATLKQFNISWLEQ-MVDLVLDRDIRIWVFLPIVVITFLVGIVRHYVSILLTSSRKAELQQVYDSQALIRVRYLRENGKYLPARGFFMRKHFFNDEETGWLK-TQKRAPPMNNPMSDPSMMSEMLKGNLTNVLPMIVIGGWINWTFSGFLTTKVPFPLTLRFKPMLQRGIELVSLDASWVSSASWYFLNVFGLRSIYTLVLGEDNAADSTRAMQDSMVPQAAAMPQDPKAAFKAEWEALEVVDHKWALTGIEEMAQYKGAASEAGRAMQILKRRERQKEEVELKRQKIEQEMRVS-MGDKFSSHFDAVEAQIKSATVGLVTLNEMKAKQQDAVKEREKRLAQKELEEKQRAEDQKKAQKEKQKKAIQALSFNLDEIKVKKNPDVDTSFLPDREREEKERMIREELRQEWKDRQRALKEENIQITFSYWDGSGHRRVVDMKKGNSIYQFLQRCLDSLRKEFYELRVVSADQLMYIKEDLIIPHHYTFYDFIVTKARGKSGPLFAFDANEDIRMTSDASKEKEESHAGKVLLRSWYERNKHIFPASRWEPYDPTKCYDKYTIKDKKKNKPMVPETLLKQRKHNAELRQQRLLAAAAKKKAARARRVLAFRRAEQYVREYRRIETSEKNNRLVAKVNGNFFVPDEPKVAIVIRIRGITGVSPKPRKVMQLFRLRQINNAMFVRLNKATINMLRLAEPYLAWGYPNLKTVRDLIYKRGFGRVNGRRVPLIDNSIIEEKLGKYGIICMEDLVHEIYTVGPNFKQVVNFLWHFKLNNPKGGWRKKTTHFVEGGDYGNRETLINSLLRKMIVRLGAEDLWKSVTSVSNAGRKRGRASGHSKKASKDLNRGQKIGSGRINMLWPGLNAPIFRGREVVERQQLPPDPEREKRLIELRDKQQNFRRIRLTPLERGWSGTRAPGRSLGPPDTPEDIDIKDFDSVVLDLRLVSNMTSHLGRVRRHKAVVAVGNGKGLLGFATGRGPDGKTALRKAKNRALLRLCHYPMFEETVLHDFFSEYGCTRIVVKQKERGYGLKCHRAIMSLCKLIGIKNHVLFVAYSNIYINAIEKSFIV-LRYSRSKFDFADEKRLHVVEFRPEQLYFPRIVASPKTVRKEDEIDPYEELDINMIVSEGRMIYARKKAEPFYTRLPGWEVHLKKTDNLKNQRQVRLRLMKKYGALKSFLNIRKPLNQIGPRKSDEKYKWTNKQRLLIFASRGITYRDRHLMNSFRTMLAHSKEECKFE--KKDINEVAEMKNCNKVMYMENRRRSDTYMWLANMQTGPTLKFLVQNIHTMEELKFSGNCLRGSRPFLSFDPAFDSHPLVKEVLAQTFGTPAYHPRSQPFFDHVFVFRLLDKRIWFRNYQIVEE-DGSLVEIGPRFCLNLVKIFDGPFSGAVIYTNPHYVAPNKMRRLAQKE--NKYMYMDVFETKNTFYRMAVGKNKGLSKGGKKGLKKKIVDPFSRKDWYDVKAPSMFSIRNVGKTLVNRTQGTKIASDGLKGRVYEVSQADLQTGEDAFRKFKLVCEEVQGRHCLTNFHGMDLTTDKLRSMVKKWQTLIEAQVDVRTTDGFVLRLFCIGFTKKAQNQVKKTCYAQHAQVRAIRRKMVEIMHREVSSSDLKEVVNKLIPEAIGKDIEKNCIMIYPLHDVHIRKVKVLKKPKFDMGKLLEMHGEGKGSGGGDGMAVDRPDNYEPPVLEDVVKIGIIGGSGLE-DPELLKEDTPYGKPSDA-LISGKIDGIDVVILSRHGRRHTINPSNVNYRANLFALK-QEGCSHILVTTACGSLKEEVRPGNFTPNTFIDRTRIQTFYVCHLPMTKPFLSKLLSEHPKGTVVCIEGPRFSTVAESNVFRQWGADLVNMTVVPEVVLAHELGIPYAALAITTDYDCWR-E--ETVDVEKVSTLKLAADGACRILRATLPKVLKIAVMMRVLRHEEFEKGCLAACNGRYDGFWSKTMVGYGSEDDHFAMELIYNYGVDKYKKGNEFKGIVIQMSNVLKRAQENRWTVQQENDKSYVEAPGGFKFYVEEDSS-ERKDPVRQVIYSCTNVEKTRRFWVTMLGCEVVESGDDFLEVAYDKSKTSLRFEKIFEPIDRGEAYGRVAFACPRNQLPEIEAQVKNADGTVITPLVSLETPGKASVEVVILGDPDGHEICFVGDEGFHELSKEDPEASQVLQEKMK--EYDAYVEKVQRRDRRFRLPPLPTPADLLRFYRLRATKQLSQNFLLDPICSKFVRSSGHVIEVGPGPGCLTRPIFEQGAESVVVIEKDKRFMAALELLANATN-NKLKIVHGDVLNSKLEDLI-PAEKAKPWEGIHLIGNLPFSISTILIIKWLHMISRKSSAWQYGRVRMTLSFQKEVAERITSPERCRLSVIAQGCYVKNGGAFVPPPEVDVNIVRFVPVQPIFQPFDLVEKVLRCLFNKTLKIELAQELLQRSDVDPPVLISVEDYSKICDAYAGLCKEPGLYEYDFRSPPMVPIVIEQTGRGERAYDIYSRLLKERIICLMGSIDDDIASLVVAQLLFLQSESSKKPIHMYINSPGGSVTAGLGIYDTMQYVLPPIATWCVGQAASAASLLLAAGEPGMRHSLPNSRIMVHQPSGGVSGQATDIQIHAEEILYLKRTVNLLYAKHTNQALETIESAMERDRFMNPEQAKEFGLIDTVLEQPPMRIERPVGTWLTLLPLWALTTAAPAGSLPDAALFCTGALLMRGFGCTINDMWDRDIDRQVERTRQRPLAAGRLSRWDALWFAGGQGLACLVLLHFNWNTVMLGLASVGLVVIYPVMKRFTYWPQAILAVVFNWGALLGFSATALPMYAGAFSWTLIYDTIYAHQDKRDDLLVGMKSTALRFGARTPLWLGAFSTMTTGLMGWPYYAAITLKIDNIGLLVGCIGGSLLKAAGSERQSVGRRSKILKAREPQVIENPKTSFVKGNNINQRTTQILKDIYALKKSESVFYQQRNPFEDPSPLEKLGKKDTSLFAFGSHNKKRPQNIVLGRTFDRMIIDQFEFGVENFKSLQEFKVPKIGVMLKPILVFSGESWQEMKRLKNFFIDFFKGDYIAVTGLEHVISFTILDILLRSYKVQLKKSGQKSPR-VEIEEIGPRMDLKIRRNKIASDDLFKQALRRPRELKVKRKKNMEQNDLGTSLGRVHMERQDFKLQTRKMKGLKVQILEALDDNYMYLVITREAAVVDPVNPEKVLRAVKVKLTTVLVTHHHWDHAGGNEKLLVFGGDDRIKELTDKDNVNITIGSLAQSLFTPCHTTGHVCYFIVFTGDTLFTSGCGKFFEGTAQQMQAAMLGSLPPETRVYNGHEYTVNNLKYAQHVEPDNQVKEKLLWAQKKPTIPSTIAEEKSINPFMRTSVQKHTDPVSTMNALRQEKDRFRMNRLFGKGKAKEPPPNLSNCIEVLDQRANNMDEKIKKLDAELFKYKEQMSKMREGPPKDLVKQKALRILKQKKMYESQRDNLMQQSFNMEQANFATQQLKDTKTTMEAMRLGVKEMKHEYKKVNLNEIEDLQDDLEDMLEQANEVQEALGRSYGMPEMDDEELEAELTMLNDEIALEDTSYLEQVRMVSRTRGKATLPDLPYDYNALEPVICAEIMTLHHSKHHNAYVTNYNIAAEKLQEAIQKNDTNAQIALQNAIRFNGGGHINHSIFWQNLCNPKSGEPSAELLAAIKKDFGSFEAMKDKMSAAAVAVQGSGWAWLGLNKANKQLQVAVCPNQDPLEASTGLVPLFGIDVWEHAYYIQYKNVRPDYVKAIWKVANWKDISQRFIKA-DDHIQHDVIKEALESGRDLREYSRSVEQQLKGSEDEAIRDYMHNCRDIAALHGEIASCDAILQRMESILRGFQNDLGSISSEIQSLQRQSVAMNLQLKNRQAVRGELSQFVDDFIVPEATINVILDCPVIDEDFLTQLALLDQKISFVKVQSFKEASSCQDVKDILDKLKVHAVTKIREWLLQKVFSFRKANANFQLAQNTMLKHRLFFQFLATHEREVAKEIREEYVDTISKVYLSYFKAYQTRLMKLQFEDVPDKDDLMGVDDTPKWGLFNKPSLKNRSTIFTLGSRNAVLGADLEAPVIVPHASAKSEKHYPFEQLFRSTQYALCDNAAREYLFVSEFFLLTKQGAVDMFDNILEKSMSLFAKHTESFVAECFDSIALFLCIHIIRKLKVVMHSRNVPVMDRYWDLLVKIIFPRFETILRMNIASIRDCDPSKLGSIDNRPHYITRRYAEFSAAIVSINENHPDERVTQLLGQLQIEVENFVLRMAAEFNGRKDQLIFLINNYDMMLGVLQQRTHEDSKETTNFRTLLTARQSEYVEQILTVHFGGMMTFIKESEYYVEKGQINELEKESHKVATLVRNFNSGWKKAIDDMNGDIMKTFTNFKCGTSILQEALKQLLQYYHRFNKVISQPPLCSLPVRSELINIHHLMVDIKKYKATFGYFIVVDAHSEECFHDRVVKGTKMGLTFEVAEGGFLDIDVKITGPDEKVVYNGERESSNKYTFAAYMDGVYKYCFSNAMSTMTPKTVMFTMDIGEEPKADGK---DGGENKLEDMISELHTAMTGVKHEQEYMMIRDRIHRSISESTNSRVVIWAFFENLIIIAMTLGQVYYLKRIFEVRRVVMRLSHILKLPKDYANLPKSYVKRAMAQVEWRTPNGRQFRRAVVQKPYTMNRPWTNEFMTNPPGVAIPVQVMFRGDRVEVMVGDDKGKQGTVNYIVPERNWVTVEGLNTEELPLLMGTQVKLVDPVDEKATDVEWRYTESGERVRVSVRTGRIIPIPLAYETIDYKYVDKAKDTSATELEKITFVP-KLATFEMDIMEQHGIQDDRVPHRTFWYVEQKMELLLWLSRLACTMFTVSYLLGNPYSLYQKALICNGVTSALRLHQRAPTVRLNAEFLTTILVEDSCHYLIYSLIF-LPIS-MVLLPPMLFAVLHSLQLLERAGILLQ---SRVRVFRLIAMTEIFLMPTVIIGLFCGVSLMAPFMYYRFLKLRYASMRNPYNRNVFYELKLHANQLAAPNCPMIIQRVRFIESRAGLAIAVITVYLSFPEMRPEEQPYVKLPTSLEDAKNLGRVLSRYTDDAVVLAFFCTYIFLQSFAIPGSIFLSFLSGFLFPFLLAIFLVCLCSAIGASLSYLISYSVGSRLILAWIPNMVYYIIFLRITPFLPNWFINLASPIVDVHIVPFFIGTFIGVAPPSILAIRAGISLQQLASALTWENIGLLTGFAAFSLLPVLLKLKNKFEEVQDKLTRIAIVSIDKCKPKRCRQECKKSCPVVRMGKLCIEVTPNDRIATISENLCIGCGICTKKCPFEAIQIINLPSNLERDTTHRYSANSFKLHRLPTPRPGEVLGLVGTNGIGKSTALKILAGKLKPNLGRYNDPPDWTEILGYFRGSELQNYFTRILEDHLKAVIKPQYVDQIPKAVKGTVRELLDRRNDLGKEDYLCELLELVNVMDRQIADLSGGELQRFATAMVCIQKGDIFMFDEPSSYLDVKQRLKSAQAIRGQIEATKYVIVVEHDLSVLDYLSDFICCLYGTPGCYGVVTMPFSVREGINIFLDGFVPTENLRFRESSLVFKVSDNTDEDVKRLARYEYPSMVKKMGDFELKVQGGSFTDSEIIVMLGENGTGKTTLIRMLAGRLKPDGDEDVPTLNISYKPQKISPKSTGTVRFLLHEKIRDAYQHPQFVADVMKPLLIDNIIDQEVQNLSGGELQRVALALCLGKPADVYLIDEPSAYLDSEQRLAAAKVIKRFILHAKKTGFVVEHDFIMATYLSDRVIVFDGKPSVSTTANMPQSLLVGMNRFLELLNITFRRDPNNFRPRINKLNSVKDSEQKKNGTFFFLEDEEDGEDLFGPELDRYDAAEEEMRRRDRGRLPRGLESIENLEDMKGHTVKEWVTQGPKTEIFNRFKNFLRTYKEKIRAMEQNRMSLEVDYTLAQSEQVLAFFLPEAPAEVLPIFDEAAKDIVVGMFPHYGRIHHEIRVRITDLPILEEIRTLRKIHIDQLIRTSGVVTSTTGVLPQLRMVKYDCVKCKYILGPFVQSQEVKPSSCPECQSTGPFSVNVAQTIFQDYQRVTIQESPGKVNAGRLPRSKDAILLNDLCDSCKPGDEIEITGIYSNKFEGSLNKANGFPVFATVIIANHILKKDTDEDVKEVVKLSKEELAERIIASIGPSIYGHDDIKRAIALSLFGGVSKNPGQKHRIRGDINVLLCGDPGTAKSQFLKYVQQIAPRAVYTTGQGATAVGLTAYVSPVTRDWTLEAGALVLADKGVCLIDEFDKMNDADRTSIHEAMEQQTISIAKAGIVTSLRARCTIIAAANPIGGRYDTFHQNVNLSDPILSRFDVLCVVRDERLARFVVDSHSRHHPISQDLLQKYILYAREKIEPKLDQDKISQLYSDLRRESMATGSMPITIRHLESIIRLAESHARMHLREHVDVNTAIRVMLDSFVTQKFSVMRMGLLDDVKEIFGADTLYEVLGVKKSVRSDLLKKAYRQKSLLCHPDKANEENKEEFTKKFQILCKCYEILCDEEKRKIYDETGSV--DDSFDEKKDWKSFWRTLFPTVTDSQIQDFFDKYHGSEQEREDLKRAYEKTKGDMNKIAECFIGYDEDRLTLLLAIAIGFLLLGKVGTKKLRKLEEKAEKRRLRELELQEREERAQKQAEDEYRKKVEQKKEDEQKQKEEEERRQKEEKERREHEEYLKMKAAFAVEEEGFDQEDDP-NSSQKLHEFITYINEHKVVQLENLAAQFRMKTQDCIDRVQRLLEDESLCGVIDDRGKFIAITRDELDEVARFIKVRGRVSIQELVENSNRLINLASDIMKQQEEAKAAALKHVVTMLQIPDQLDKVEQHRKRVQRKKASVEAMLKTAVQSQLEGVRTGILLLAAARDDVNDIQKDEADTIYDLSKLVQLQDVREESFRHSQTGTLMEHLKHIFNVPGSITRTQDLIQDGNLLLAHKLSDLECSRDELLYELHKQANNQASDRAMLKQYFADVERLSDELAKQLWLILKRTLNTVRKEPQVIVTALRLIMREEWAALKRQQST-GFLPPGRPKLWRKKAIETLEQSVAERLEGNQIEVRSENNMWLVRHLEVTRQLIIEDLKTVKHHCTPCFPPCFDIFNEVRMTHNCLSQRLQTIISGLVDSEYIHVLQWLNTYNSRELMGHPDLHVDVALLPDTIERLMERYLAGLRTKFEEWLRNALSDHKDWPEQDSNGYYRTETPMLIYQMITQHVEVARTVDLVSRVLRLAMNRMDYFLSQYNQLVTDYNFEDRSYTAYMIAIANNAVNMK-LRELKDNSLDYLCEEVLTDIKVMPDIMTETVTVTLADYGGDYIEKEVAASYVKAIC--EKRMSFRNYEERRAAAELEKLKKSKLDVLKLMAEVLKMKDSSLLSLEMNGSRTDAEVIAREEEAIKVDSLSVAQLKEIREKAQKHAFQAEVARMMKLIINSLYRNKEVFLRELISNASDALDKIRLLSLTDPDALKKLQDLSIRIMADKENNVLHITDTGIGMTKEDLMKNLGTIAKSGTAEFLQKVSEGSSGKDLNDLIGQFGVGFYSAFLVADRVAVASKHNDDPIQHVWESNAAEFSVADDPRGDTLKRGTTVSLYMKDEAKDFLEHDTLKKLIEKYSQFINFNIYLWSSKT-VTEEVTESEKPAEE-----ADDDEAKVEDAKEP--LKTKKVDKTVWDWELINSAKPIWTRKEKEVSDEEYNEFYKAVTRDSQNPLAKTHFTAEGELTFKSLLFVPSKQPQDSFNRYGQKTDHIKLYVRRVFITDDFQDMLPNYLSFLRGVVDSDDLPLNVSRENLQQHKLLKVIKKKLVRKALEMFRKISEEEYVKFWKEYSTNIKLGVIEDSANRSRLAKLLRFPSSLDSTDKLVSLGDYVQRMKEKQTAIYYIAGNGMDEVKKSPFVERLLKRGYEVLYLTEPVDEYAISSLTEFEGKKFQNVAKEGLSLDD---NKDIREAMEKEFEPLTKWLSETALKDKISKAVISERLVETPMALVASQFGWTGNMERIVAAQTHMKENDPQKSFYMTQKKTLEINPRHPLIKELLRRVDDSPSDEMAKYLTEMMFETATLRSGFQLNDNAQFASNVERMLRKMMGVSEDAQVDAELGRRGEVKPLSAQEMKMIVISEIIQELVQAHNDKRDVNLNRVKYDVSARYGLKSQPKLVDIIAAIPPQFKQILLPKLKAKPVRTASGIAVVAVMCKPHRCPHINYTGNICVYCPGGPDSDFEYSTQSYTGYEPTSMRAIRARYDPFLQTRHRVEQLKQLGHDVDKIEFIVMGGTFMSLPESYRDYFIRNLHDALSGHTSANVDEAVKYSERSKTKCIGITIETRPDYCLQRHLTDMLRYGCTRLEIGVQSVYEDVARDTNRGHTVQAVCETFHIAKDCGFKVVTHMMPDLPNVDFERDVLQFVELFKNPSFRMDGLKIYPTLVIRGTGLYELWKTGRYKSYPPALLVDLIAKIFSLVPPWVRIYRVQRDIPMPLVSSGVENGNLRELALARMKDLGLVCRDIRTREVGIQEIHHKVVPYHIELVRRDYVANGGWETFLAYEDPQQDILIGLLRLRKCTEQTYRPELLGQCSIVREHVYGSVVPVHSRDPSKFQHQGFGTLLMEEAERIAREEHCSKKIAVISGVGTRNYYRKLGYELDGPYMSR

'Varroa_jacobsoni' PGQRVNVLSKELMTSVVLISGKKGCFIAGADITMLEQCRSEAKNLKPIVAAIMGSCLGGGLETALACRYR-IAVEEPKTTLGLPEVMLGVLPGGGGTQRLPKLIQLPTALDMMLTGKSLHAKKAKKVGLIDAIVKPLGPGLYLEEVAARDLATGELKIRARPLTERIVRDM-IFDKARGQVMKLTNGLYPAPLKILDAVRAGLEKQNFAELCTKESKGLMGLYHGQVHCKKNAFGKPTENIAVLGAGLMGAGICQVSLKDFNRVVMKDGLVRGQNQIKKKKIQKDRLMSTLLPTLDYSDMIIEAVFEDIHVKHKVVKEVEAEHCVFASNTSALPIAKISEVSKRPEKIIGMHYFSPVEKMQLLEVITTDKTSKDTAATAVDVGLRQGKVVIVVKDGPGFYTTRILAPMMSEAMVLLMEGCQVKELDKLKAFGFPVGAATLLDEVGIDVGAHIAEGVFGDRKEMVNNFLGRKSGKGCYIYRAVNPIQRKYTTEQVQFRLATRFMNEAVMCLQEGILANPVEGDIGAVFGLGFPPNRGGPFQFIDTYGADKIVNMRQFQEFEPCQLLLDHA-NDPLKKFH???????????????????????????????????????????????????????????????????????????????????????????????????????????????????????????????????????????????????????????????????????????????????????????????????????????????????????????????????????????????????????????????????????????????????????????????????????????????????????????????????????????????????????????????????????????????????????????????????????????????????????????????????????????????????????????????????????????????????????????????????????????????????????????????????????????????????AKVLNRLEAFREHEMAVTRDYISQPRMIYKTVCGVNGPLVILDQVKFPKFAEIVQLVLADGTPRTGQVLEVSGDRAVVQVFEGTSGIDAKNTVCEFTGDILRIPVSEDMLGRVFNGSGKPIDKGPPVLAEDFLDIQGQPINPWSRIYPEEMIQTGISAIDVMNSIARGQKIPIFSAAGLPHNDIAAQICRQGGLVKRPQKSVMDDNFAIVFAAMGVNMETARFFKQDFEENGSMDNVCLFLNLANDPTIERIITPRLALTTAEFLAYQCEKHVLVILTDMSSYAEALREVSAAREEVPGRRGFPGYMYTDLATIYERAGRVEGRNGSITQIPILTMPNDDITHPIPDLTGYITEGQIYVDRQLHNRQVYPPINVLPSLSRLMKSAIGEGFTRKDHADVSNQLYACYAIGKDVHAMKAVVGEEALSPEDMLYLEFLGKFEKNFISQGRYENRTIFESLDIGWNLLRIFPKEMLKRIQHSLLAEFYPRAEAKTGLFEDPNGFYLLKENAITQAEQLIAEAMYRKRKMVQIFDDLSDCLCRVADLAEFVKVGHPQGRYAQAAEHASLAISSLVEKLNTNRELYSALRSVIENG-DIVPTTAEQHVGRLFLFDFEQCGIHLDEERRQRVVALNDHILYVGGQFLQNSHRPRYVRQSGMPENLLVVSGLQADCSNELVREAAYRIYLYPDDHQLGLLDELLRSRHELARLCGFDTYAHRVLKGSIAETPENVTFLSYLSAELGPRAERDYQEMMGMKPWDVPYFTAYLSLASCMEGLDMIFNALYGINLEVVGELWHSSVVKLVVKNLASHMGVIYCDLFERPGKPHQDCHFTIQGGRRSDGSYQIPKVVLMLNLPPPLLTPSLMDNLFHEMGHAMHSMLARTEYQHVTGTRCATDLAEVPSILMEYFASDPRVVSKFARHYRTGELMPAEMAASLDASRVIFQASETQLQVFYAFVDHEYHSKYPLNTTEILRDVQNKHFGVKYVDNTAWQLRFGHLVGYGAKYYSYLMSRAVAATFWHRAFNADPFSRCVGTYREEVLAHGGALPPAQLIQNFLLAESLIRDIMPSDAKKKRDAKKKEALKNRNNPDATNGEDEMDEVTKKFEEDMKLNAAARAVTGVLSIHPRSRDIKIENLSITFHGWEVLQDTKLELNCCRRYGLIGLNGCGKSTLLSAIGRRELPIQECLDIYHLTRECPPSEKTALQMVLDVDKERARLEKLAEELAASDDDTSQEQLMDVYERLDAMSADTALAKASYILHGLGFTQSMMHKKCKDFSGGWRMRIALARALYVKPHILLLDEPTNHLDLDACVWLEEELKTYSRILILISHSQDFLNGVCTNIIHMNLRKLEYYGGNYDQFVITRNEMLENQMKRYNWEQAQMSHMKDYIARFGHGSAKLARQAQSKEKTLAKMVAGGLTDKVVYDKTVSFYFPSCGTIPPPVIMVQNVSFRYTDKTPFIYKNLEFGMDLDTRVALVGPNGAGKSTLLKLLCGALVPTDGIIRTHSHLKIARYHQHLHESLDVDLSALEYMMKSFPDVREKEEMRKIIGRYGLTGRQQVCPIRQLSDGQKCRVVFAWLAWQVPHMLFLDEPTNHLDMETIDALAEAINNFEGGMVLVSHDFRLISQVANEIWVCENQTVTKWRGDIKTYKQHLKNKVMKEMEKMVLADLGRRITSALRNLSTATVINQEVLDSMLKEICAALLESDINVRLVKQLRENVKAAIDIDEMAVGLNRRKVVQSAVFKELVKLVDPGVRAWQPSKGRSNVIMFVGLQGSGKTTTCTKLAYYYMKKGWKTALVCADTFRAGAFDQLKQNATKARIPFYGSYTEVDPVVIAADGVSKFKAEHFEIIIVDTSGRHKQEDSLFEEMLEVSNAVSPDNVIFVMDASIGQACELQARAFKEKVDVASVIITKLDGHAKGGGALSAVAATRSPVIFIGTGEHIDDFEPFRVKPFIQKLLGLGDIEGLIDKVNELKLDENHELIEKLKHGEFTLRDMYEQFQNIMKMGPFNQIMGMIPGFSADFMSKGNEQESMARLKRLMTMMDSMTDEELDDREGAKLFARQQTRITRVARGSGCSTFEVHELLNQYTKFAAMVKKMGGMKGLFKGNDLARNVNPAQMNKLSAEMAKMIDPRVLQQMGGFSGIQNMMRQMNASSLMQDGTFIKLKKLYSMKKGTLNINSLFENDPSRAEKYTIKLADGDETLLIDYSKNLIDDEILVNLVELAKNREVELMRAKLFSGEKINFTENRSVLHVALRNRSNRPITADGEDVMPKVNAVLEHMKAFCQQVISGEWKGYTGKKITDVVNIGIGGSDLGPLMVTEALKPFQVGPRVHFVSNVDGTHLFETLKKVDSETTLFIIASKTFTTQETITNAESTKQWFLDKAGDKAHVSKHFVALSTNKPKVEAFGIDAANMFEFWDWVGGRYSLWSAIGLPIALFIGMPNFEKLLAGAHFMDEHFRTTPLDKNVPVILAMVGVWYINFFGAESHCLLPYDQYLHRFAAYFQQVDMESNGKYVQRNGERVDYQTGPILWGEPGTNGQHAFYQLIHQGNRLIPCDFIAPVKTHNPIRGGVHHKILLANFLAQTEALMKGKSEQEAKSELKASGLSEEVLERILPHKVFLGNRPTNSIIVQQVTPFTLGALIAMYEHKIFVQGVIWNINSYDQWGVELGKQLAKRIELELNGKDPVSSHDPSTNQLINFINSYNTNVSYQDRDKPAQVRQSNITASKAVCDAVRTSLGPRGMDKMIQAVSGDVTITNDGATILQQMQVLHPAAKMLVELSKAQDIEAGDGTTSVVVIAGSLLDAASKLLLRGIHPTIISEAFQAAAKECVDILSCLAIPIELSDRESLLQSATTSLCSKVVSQHSDVLAPMAVDAVLKVIDPNNVDLRDIKIIKKLGGTVEDTELIDGLVFTEKLAGGNSPHRVEKAKIGLIQFCISPPKPNMDHQVIVSDYTVMDRVLREERAYLLNIVKVVKKAGCNVLLIQKSILRDAVSDLALHFLAKMKIMVIKDIERDDIEFISKSLGCRPIASLDHFVPEALGSAELVEEVTSAKYVKVTGVANP---KTVSLLLRGSNKLVLEEADRSIHDALCVVRCLVKKRALVPGGGAPEIELSLRLAERAREIEGLHSYCYRAFADALEIIPYTLAENAGLNPIQTVTELRNRHAQDKRTYGINVRRGCVTDILEENVLQPLLVSTSAITLAAECVRSILKIDDIVQTVRMSKRDVVYLWDPDVGNFHYGPGHPMKPQRIAVTHSLVLNYNLHTKMRIYRPYRANPHDMCKFHSEEYVNFIERVTPKNIQTFSKSLTHFNVGDDCPVFDGLYDFCSMYTGASIDGAWRLNNKSCDIAINWSGGLHHAKKFEASGFCYINDIVVAILELLKYHARVLYIDIDVHHGDGVQEAFYLTDRVMTVSLHKYGAYFFPGTGDMYEVGAESGKYYALNVPLKEGECFLNLKYVFKSVISSVIEHYNPGAIVLQCGADSLAGDRLGCFNLSIKGHGECVRFVRDLNIPLLVLGGGGYTLRNVARAWTNETAILVDEQVSSEIPFNEYLEFFAPDFSLYPD-------ENANSKQYLEQIIKYTTENLRCLDHAPSVQMQDVPPELADLGDDQEKRETTKEHPAEFYD---------------MSDAEEDYELEYSEDDDSQPDVDLENQYYNSKALKEDDPQAALQSFQKVLDLEGGQKGDYGFKALKQMVKINFQLGEFKEMMSRYKQLLTYIRTAVTRNYSEKSINSILDYISTSKKMDLLQEFYEVTLEALRDAKNDRLWFKTNTKLGKLYLDREEWNRLARILRQLHLSCQNVDGSDDLRKGTQLLEIYALEIQMYTSQKNNKELKKLYEASLQIKSAIAHPLIMGVIRECGGKMHLREGEYNSAHTDFFEAFKNYDESGSPRRTTCLKYLVLASMLMQKEINVLDSQEAKPYKDDPEIVALTDLVDAYQAHDISRFESIVSPHKESIMKDAFIKEHIEQLLLNIRRQVLIRLIRPYTRITISFISRELNIPSAEVESLLVSCILDNTINGRIDQVKQVLELNP-SPTDRRYTAMEKWAAQVQLIQSTVVGKLAQLGTLNPVQILNQQAEEEKAENARLSSFVGAIAIGDLLKSTLGPKGMDKILLCETSRDSKVEVTNDGATILKAIGIDNPAAKVLVDISKTQDDEVGDGTTSVAVLAAQLLQEAEKLVGMRLHPQTIIAGWRKAVVAARAALEEFSQNRSNDEAQFRIDVLNIARTTLGSKILSQHKDFFAQLAVDAVMRLKGKSNLDAIHIIKKLGGSMLDSHLEQGFLLDKKPGLNQPKRVEKAQILIANTPMDSDKIKAGKLRDECFSRVVVAELEDAEKLKMKRKVDAILAHKCNVFINRQLIYNYPEQLFADAGVMAIEHADFDGIERLALVTGGEIVSTFTSPESVRLGTCDVIEEVMIGEDKLLKFSGVPLGEACTIVLRGATQQILDEAERSLHDALCVLAFVVKEKKICYGGGSAEMLMAAAVDSVAQTTPGKEALAIEAFARALRQLPTIIADNAGLDSAQLVSELRAAHANGQSTFGINIADAKIDDMEKLGVTEAFVVKRQVLLSASEAAEMILRVDSIIKDAPRKRVPDRSHMAASIKPSGKPQLLCRLDGHTDTVNQVVLIGDADAVISVSDDRTIRVWARRDTGQYWPSVCHTMPSLASAMDYDPQLRRLFVAMDNGSITEFELADDLNKITYRRSYIAHQQRVTSMKFSPVTEWLISAGKDKYFQWHCTETGRRLGAFLGSAWCTTVALDQASRHAFVGDYSGEITMLKLTETSYQPVTTLKGHSGSVQSLLWDERRRLLISGGFDQIIIVWDIGGGKGTAYELSGHRARITGLALYSASSLLSVSEDSTLVVWDVAAQRQETPEWTTRDCCERCARPFFWNLRARTSNSDVGSRQHHCRRCGRALCDACSENRSTLPRLGFEFPVRICNECHLHISDGDREPLAKFHDLKAPVTAMSLSAESKTMVTIGTDRSIKIWDLSKVLSKKALKKAQKEAEKAARKAAHK---AAGKENGSDDGSQGLYGQYPMIQSTEKLQREMIEISQCTLERADQMVWLRGRLHTSRAKSKQCFFVLRQQHYTLQCLLDVSEGTSKQMLKFISAIPKETIVDVEGKLVKSPLKIESCSQQEVELRIYQFWIVSLSDTRLPLQVEDASRPEPAEGDEEALKIRVNQDTRLDHRVLDLRTPANQAIFRLQAGVCHLFRESLNRRHFIEIHTPKIISAASEGGANVFEVTYFKGKAYLAQSPQLYKQMAIAADFDRVYTIGAVFRAEDSNTHRHLTEFVGLDLEMAFKYHYHEVLDTIAEMFVDIFKGLRDRYQPEIDTINKQYPSEPFKFLEPSLRLEYSEGVAMLRAAGVEMADDEDLSTPNEKLLGRLVKAKYDTDFYVLDKYPLAVRPFYTMPDPNNQMLSNSYDIFMRGEEIMSGAQRIHDPEYLTQRAKAHGIDISTIQAYIDSFRYGAPPHAGGGIGLERVVMLYFGLNNIRKTSMYPRDPKRLTPAMEEGLQLNEINPLEFSRVKMVYPKECRIRRISYRGKLDLTLWWMNGIKQEPIKRTCGEIPIMVKSLKCNLYGLDPEQLVERGEEMEEFGGYFVVNGNEKVIRLLIMQRRNYPIAMARNGWKNRGSMFSEFGVSLRSCRRDAQNMVLHYLTNGTVQVMITYRKEVYFVPAVLLLKALVNKSDYDIYRTLTQGCENDSFYKGCITNMLRLVQENILTQDAKEFIGDKFRWSSNADVCDQLLKRCVCVHLDSNEDKFNLMCFMVRKLFAVAKNKCALESADSTMNQELLLPGHIYLHILKEKIEGLLVGVKISIDKKMSNALDVTNAMNYFLSTGNLVSKTGLSLQQTSGFTILAEKLNFWRYLAHFRCVHRGSFFMEMRTTTVRKLLPEAWGFICPVHTPDGGPCGLLLHLSAMCEVVVQLDGRCIGWVLRYLKATGNIPPTLEICVIPRTEQNSLFPGLYIFSTPARMMRPVLNRTQTVEWIGTLEQVHMDICVIAEEATTHQELRETAMLSVLANLIPWPDFNQSPRNMYQCQMGKQTMGSPMHTFRYRADNKLYRLYPQSALVRPTSYDHFQMDEYPSGTNAVVAVISYTGYDMEDAIVLNKMSVERGFKAGVYKTETINLRVDVDGLPYIGSQGDPVCAYIQLKTVRYYSTEPAIVHEVKILGNLQQIQLTYLIRTPMIGDKFASRAGQKGICSALWPTESMPFTDSGMVPDIIFNPHGFPSRMTIGMVVESMAGKSAALHGYVHDASPFKFSEEYPSSAYFGELLQRAGYNYYGTERMYSGVDGREMEADIFFGVIYYQRLRHMVADKYQVRTTGPVDSLTRQPVKGRKRGGGIRFGEMERDSLLAHGTAFLLHDRLFNCSDKTLCATCGSIISIPIPYVFRYLAAE-MVSINMKIIRDTSRDLHRHQRNAPELHPMTVVREYTAAVNAAKLDRIFAKPFLCSLGHRDAVELLAKHPDRISGAVSASADGELRWWDLSNRKCVRALQAHDGPIRGLVGQDQTIKTILSKLAHHYNNMFATAGETVSLWEERNEPLRSFW-GVDTIYVIFSPIESLSSDRSIVLYDIREASPLRKVILEMRSNALAFNPMQAMHFTVANENYNLYTFDMRHLKKALQSHTDHVGAVLSVDYSPTGTEFVSGSYDKSVRIYRSREVYHTKRMQRVTSVMYSLDSKYILSASDEMNIRLWKAKASEQLGIQNYNETLLQRFQHHPQVKRIVRHRHIPKTLYQEKQTMVTARKRKMGMQFLQRGAYQDALSHYHAAIEGDDTNYQSFYWRATVYLALGKSKLAVEDLNRVIELKDDFLKAREQRGNILLKQGHLDEAHIDYEFVLRLEPDNPEAMIEELKNDVIHILQRVWNLKLRELRASCYESIGDIQSAITDLRPAIRSVPDNTGGYLHLAQLYYKHGDPDDSLTTIRECLKLDPDHKECYKNVKKLAKSMQEECVEKMVGLIKTKCQCASKGGSAVQICTEALALDPILCDRGEAYINQDDFKQDFAAARELDQRAGEGLKRAQKLEKSRGKRDYYKILASKREIAKAYRKLAAEWHPDQY-QGID--KKNAEKKFIDIAAAKEVLTDPDKRAKFDRGEDPLDPESGFHYTFPFMLVLFESPAGYAVFKVLDEKKVQKTDNLFKEFEDASGAAKILKLKHFQKFQDMTQALSAATAAIEGKLCKPLKKVLKKLAVSDAHETLAVADAKLGNIIKEKMDILCVANSSIQELMRCIRSQQEALITGLSQKEATAMALGLAHSLSRYKLKFSPDKVDTMIIQAVSLLDDLDKELNNYVMRCKEWYGWHFPEMSKVVTDNMLYVKTVRKMGMRSNAINLDLSDILPEDQEAKIKELAEVSMGTEIAPDDVANIMHLCDEVIQMTEYRGTLYEYLKNRMTAVAPNLTVLVGELVGARLIAHAGSLLNLSKQPASTVQILGAEKALFRALKTKHDTPKYGLIYHAQMVGQSSQKCKGKASRWLAAKSALAIRVDALGEDTDTEMSLRNRANLEARLKMLEDGKLTRISLFLWPKNSTKLQFSIFVCFGIMIVGRVCTPIAPIMQKKIVDGLVSSTGLLF---LFQGANSLMANVRSYLWLGVQQYTTKATQVSLYAHLHSLSISWHLSRKTGEVLKVLDRGTSGVQNLCSYLLFQIFPALTDIVIAFGYFTYAFNWFALVAFVCMGSIVLTEWRTKFRHEMNHLENAAYAKSVDALLNFETVKYYNAEEIEIAKYQNAEWKSQSSLALLNIIQGSTSSIIGALLCAYITAGDYVLFIAYNAQLYAPLTFLGTYYRMIQQSFTDMENMFELLDVVDHHLKL--EIEFRDVCFRTVLKHISFVVPHKHTVALVGHTGSGKSTILRLLLRFYDVQSGSILIDGQNISAVSLRSHIGVVPQDTVLFNMSIRENIRYGRPSEDVEAAAAAADLHHSIFQMYETVVGERGLKLSGGEKQRVAIARTILKAPSIIVLDEATSALDTQTERNVQKALNVLENRTSIVIAHRLSTIINANQIMVLEHGEIVEQGHEELLSKKYASMWRQQQALNVPETRVTTLSNAVRVASEDTGAPTATVGIWIDAGSRYETEKTNGVAHFLEHMAFKGTGKRSQTDLELEVENAGMHLNAYTSREQTVYYAKCLKKDLARAVDIIADITQNPKLGEQEIERERGVILREMEEVEGNLQEVVFDHLHSIAYQGTPLGLTILGPTENIKSLQRQDLKDYIDTHYKGSRIVLAGAGGVDHDELVKIAEQTFGKVSNSMDSQ--APCRYTGSDIRVRDDDMPFAHIAIAVEGAGWANADNIPLMVANTMIGSWDRSHGGGANASSRLAAWAQSVKSMHSFQSFNTCYKDTGLWGLYFVADGDELDDIMIAVQEEWMRICTEATDGDVTRAKNLLKTNLLLQLDGTTPLCEDIGRQMLCYGRRIPLHELEARIDAIDADTIRNVCQTYIYDRCPVVAAVGPVEGLTEYTRIRGQMYKHSVHTLVFRSLKRSHDMFICEEGALPPIDEKAHKLRVGTKGRDEYGPVMHLVSEGRRSVSSGPLVLAGQQLTAQLKKPSIPKPTWHPPWKLYRVISGHTGWVRCVAFDPTNEWFCTGSNDRIIKIWDLASGKLKLSLTGHISGVRGLAVSQHHPYLFSCGEDKQVKCWDLEQNKVIRHYHGHLSGVYTIGLHPTIDVIITGGRDSTARVWDMRTKANIHVLSGHTNTVASVLVQATEPQVVSGSHDSTIRLWDIVAGKTRVTLTHHKKSVRALVLHPKLNMFASGAPDNIKQWMCPDGKFIQNLSGHNTIVNCLAMNEDGVLVSGGDNGSLQFWDWKTGYNFQKLTTPVQPGSIDSEAGIFAMSFDLSGTRLVTCEADKTIKIFKEDETATEETHPINWRPEIVTGSTSEYLINCSAEHYLEILGINVKAKNFLVFQGAVESIAMKNPKERTVLFEEISRSMISAEEETYQKKKGIAAERKEAQIEEAEKYQKLKEDVQVNLHLFRLFHQEEELQKKRRKDKIEAELKEKKKVEQTHRDDVELNKRKPAYIKAKEKTAHMQKKLDAAKKSLEAATKTHKSHQGIEELEHELSQVEEAYEQEDVSLEESQVKEYNRLKEKAGKMASAALQEYDSVAREQKTDQDHLDNELRKRNECEAKLKELEENQRRINKLVDLQDLKEEEKKLEAKKRLTQKFEDVASLGDAKVDKHEDARRKRKSEIVEHFKKLYPGVHDRLVNLCHPIHKKYNVALTKVLGRNMEAIVVDTEKTGRACIQYLKEQMLEAETFLPLDYIDFKPLKERLREFKDVPNVKLLYDVLKYEPLSIKKAVLYATNNALVCETAEDAAKVAFQSPDGKRYDAVALDGTYYQKNGFISGGSSDLAKRAKRWDDKDFHKLKDQKEKLQEDLREAMKTARKESDLTTIESQIKGLETRIKYSKVKQLEQSMREREGRINEIKARQNTVEDDVFRDFCEQIGVANIREYEERELHASQEREQRAELENQKNRIASHLEYER--TKDTLAVEQDQQELGRLKEIEQKQKELIEQQMEAISTLKNERQSKKIRVDEIDEEVAEIRKRLTAQQKEVTGVQKTVTQAEAKLEQKRSERHTLLQSCKLEGIRIPLIRGSMSQMYAREAMEIDYNQLRMEKEINLQRIQAPNFKAMEKLDSVKERLKDTDTEFEHARRKAKSNFELVKRERTCFEHVSNCIDEIYKSLTNNPSAQAFLGPENPEEPYLEGINYNCVAPGKRFQPMSNLSGGEKTVAALALLFAIHSYQPAPFFVLDEIDAALDNTNIGKVARFIREKTQTSFQCIVISLKEEFYGHADCLVGICPDPGECTISRIYTIDLSMSEAMLLYLGQAIREGNLAEMQNIYERSLVNLMDQYLIIIYKE--LYYRQIYADDRFSSYYNYVNLFNYILSSGPVSLDLPNQWLWEIMDDFVYQF--QSFSTFMWNVHSVLNVLHSLVQKSNINQQLEVFSKGGNPDEVAGEYGSRPLYKMLGYFSLIALLRLHSQLGDYYQAIKVLQHLELNRKGLSRVPACQVSTYYYVGFAYMMMRRYEDAIRTFSDVLVYIGRTKRNYQLRQMNNQMEKMYALLSICMVLHPQRMDESVLQQLKLKHMMKMSQGDLDTFERCPRFLSPVQWRAFRGEVVAQMNMRSFLKLYTTMPVEKLTKFVLMCFKHLMSNVVLDGEFQTGSDMDFFIDKDMIHIADTKLARKYGEFFAKQYNRFEEWYEMGPPDAILGVTEAYKKDPNPKKMNLGVGAYRDDDGKPFVLPSVRAAERQLMSKNLDKEYLPIGGLNDFCKNAAILALGDNSAVIKEDRNATVQGISGTGSLRIGAMFLDEFLKGNKTVYMPNPTWGNHIPLFKRCNFQVKQYRYYDPKTCGLDFQGALEDISSIPEGSVILLHACAHNPTGVDPRPEQWTEIEKVVRERNLFPFLDMAYQGFATGDIDRDASAVRLFAGSG-PMCLAQSFAKNMGLYGERVGAFSLICSSAEEQARCMSQIKILIRPLYSNPAVNGARIANLILSDPQLRAQWLKDVKGMADRIITMRSRLRSGLKREGSTHDWKHITEQIGMFCFTGMTAEQVTRLIKDYSVYLTKDGRISVAGISSHNVDYLARAMHEVTKHIRPSFYHALHCILMAVLMYVFVEFSKPPDQVDPFSEYGVIFTIILYLFRLLPLLALPQSLTNLFGLTLYNAFPPRVRLKVKPHEAPFLCIRVVTRGDYPGLVRENVKRNLATCLDTGIDNFVIEVVTDKEVYVTANSKIRQTVVPKSYNTTTGAMFKARALQYCLEDNVNLLADGDYILHLDEETLLTKDALRGVLNFISAGRCSFGQGLITYANERIVNWFTTSADMYRVADDLGKLRFQFNFFHKPLFSWKGSYVCTRVGAEREVSFDHGPDGSVAEDCYFSMVAFSKGYSFEFIEGALWEKSPFTISDLIQQRKRWMQGIYLVVHSAKIPWRYKVWLSCSLYAWATMPLSTSNLVLAPNFPLPCPQTFNIICAFIGALNIYMYIFGLIKSFSISRYGFFGFWLCFMLVVIAIPLNIVVENVAVVWGLLGNKHKFYPWPHYHFSGKLRPGRISTKRTVPAHIKRPDYADHPEGIPVSEQAMKGA-EIKVLNEAEQEAVRKASLLARECLDVALAAAKPGVTTDELDRLVHEAAIARNCYPSPLNYYKFPKSCCTSVNEVICHGIPDDRPLKDGDILNVDVTVYHNGYHGDLNETIFIGKVDEAAKKLVRVTYESLQKAIECCRPGVLYREIGKIIQKHVQQNGFSVVKSYCGHGIHSLFHTAPSVPHYAKNKAVGVMKAGHCFTIEPMISEGVWQDEVWPDNWTAVTTDGKRSAQFEQTLLVTDTGVDILTRRRKKNGQPWFMDQSTLRECLKERSAKRRELLARQLGAGCAENLGLLLGNDKTTATEQGVTLDDEEVMAYRDSSTFLKGTQSANPHNDYCQHFVDTGQRPQNFIRDVGIQDRFEEYPKLKELIKLKDELIHETATPPMYLKCDLLQYNLRELNGKFDVILIEPPLEEYQRSCGVTNTRFWSWEEIMKLEIEEVAAPRSFVFLWCGSSDGLDLGRQCLRKWGFRRCEDICWIKTNINNSKVKNVEPRAVFQRTKEHCLMGIKGTVRRSTDGDFIHANVDIDLIISEEPPFGMMEKPEEIFHIIEHFCLGRRRLHLFGRDLTIRPGWLTLGPELTNSNLNTEAYNAHFNTANDYLTGCTERIEALRPKSPPPKGKRVVMRVDFNVPLKDGKITNNQRILAALPSIKYCLDKGAKSVVLMSHLGRPDGQSNSKYSLAVVAEELNQLLGKKVIFLNDCCGAQIEAACADPTPGSVILLENLRFHIEEEGKGVDTAGNKIKADASKVKEFRASLTRLGDVYVNDAFGTAHRAHSSMVGVELPRRAAGFLMKKELDYFSKALDQPARPFLAILGGAKVKDKIQLIENLLDKVNEMIIGGGMAYTFLKVTRGMKIGDSLFDDDGAAIVEKLMAKAASNNVQIHLPSDFVIADKFHEDATTGTADISNGIPDGWMGLDCGPKSVELFAGAVSRAKTILWNGPAGVFEFEKFAVGTKGLMDVVVAATDRGAVTIICGGDTATCAAKWGTEDKVSHVSTGGGASLELLEGKILPGVAALSDAMRTFGDRPVSFQLEDGGDYYYIGTEVGNYLRLFRGTLYKKYPSLWRRAVTVDERKKISQMNMSQHSAANFISLLKKSEVDDLIDGNEEKYRAAPVQENEGGGHGAKNARPSFMPAAPNNAHHLDAVPCSTPINRNRLQHKKNKSFPMLYDDLDPAMLHESAALPECLVPIRLDMEIEGSKLRDTFTWNRHEAHISPEQFAELLCDDLDLPPLLFVPQIAASMRQQIEAFPSESLLDEQTDQRVLIKLNIHVGNISLVDQFEWDMSERANSPEEFATKLCSDLGLGGEFVTAIAYSIRGQLAWHQRTYAFSEAPLAQLEMPFRAQSEAEQWCPFLETLTDQEMEKKIRDQDRNTRRMRRLANTGWMGANGSTLDRDDALPSSERYLGLVNFGNTCYCNSVLQALYYCKPFREKVLEYKAKNKRTRETLLTCLADLFHNIHSHKKKTGTLAPKKFIARLRKDNEVFDNYLQQDAHEFLNYLLNTIGDLLQAESSWVHDIFQGTLVNETRCLTCETVSSKDEDFLDLSVDISPNTSISHCLRGFSSTETLRGEHKYHCEQCNSKQEAQKSLKVKKLPPILALHLKRFKYTEQQNRNTKLSWRVVFPLELRLFNTSDDALNGDRLYDLVAIVVHCGTGPNRGHYISIVKSHGLWLLFDDDMVDKIDPSTIDDFFGLTQDTPKSSESGYILFYQSKENADIDNWIELAKQCKYLPEADLKKLCNMVCQILIEENNVQPVSSPVTVCGDIHGQFYDLEELFRCGGHVPDTNYVFMGDFVDRGYYSLETFTRLLTLKAKYPKKMTLLRGNHESRQITQVYGFYDECQQKYGNANAWKYCCKVFDLLTLAAIIDGEIFCVHGGLSPEIKALDQIRTIQRNQEIPHKGAFCDLVWSDPDEVETWSCSPRGAGWLFGAKATHEFMTYNSLSLICRAHQLVHEGYKYMFEDKLVTVWSAPNYCYRCGNVAAVLEISNDQKKNPKIFNAVPDHERVIPERHAPYFLVDSASPEETQDAEAATIDELKEHTAHIQKAVAQKESRFILRILRLLPATRKKLNSKLLRKTINGFYTHDKMHREVLLSFVDAEDADTDAAQKSAHLALLPEVDVYLHLLLLVHMIDAANMERAIRCAELLKGKVEAHSRRSMDLLAAKTYFYYSRVYELDGNLSSIRGFLLKRLRTATLRSDFEGQAVLINCLMRNYLHYSLFKQAAKLVSKVTFPEMASNNEWARYLYYLGCIKAIQLYYTDAHKNLLQAIRKAPQHSALGFKQTVYKLAVTVELLLGDIPDRTTFRQPALRKSLAPYFQLTQAVRTGNLGLFNQVLESYGARFQADHTYTLIIRLRHNVIKTGVRMINLSYQRISLADVAAKLQLGSAEDAEFIVAKAIRDGVIEATIDHDKGYVQSAENIDVYCTGEPQSQFDQRISFCLDIHNQSIKAMRFPPKSYNKDLESAEERREREQQDMEYAKEEDDDTFQVAVRLTLALAAMAARTLWQSAVTDMIENFRDSQPLLFEFLARLPEEATAGYIYYTSVLSLCQSALASNLRITAMRTVPANLCITLL-DLTDDHACDAILSFLQHPDGHYPKLMGDLLEQVVKCGPIVETKRACGDQDSIYSLLTGMGEMHTNLILASLLPDSSNRTEMLLKMLLDCVGTPGQYPSEEIISRIPITFWHILLDELARVEPSTQMAKQLQPVYEQLVKMLRKSQLPDPGTMDLDEKEDLRCYRQDIAD--CYMYIATMLPAPVFYFFISALDTAKNAKVIEACLFALNAIGDMADSEDDSPVVGAVLALLPRIPAGDEVLSQVMTAVGIFAEENIGPLVHLLLRGLQQTSASASMALKDLARTHGDRLAPAANDILQAIAVLKHRDRVRLVAIVGHVVSALSSEQALTSLSALMAPFVQQLNEITNLVEQLMPLFKLIAAKY-SCDAEVVSNLAECIRKAVPVLELEVVLSELLSMCGALLLGAATSVDLHGCTDAVESFYKLAVLFKKFSLDDLIQLCPEQFTYRALTQFVNASERSPVKEALENIIAQLVQNIRVSRNFIEAEADVFLALNKTAAALQRFVSSPESRQNFARQILRERSNKRMLCKVI--IDFTLKVKVKWKEVY-DVEVHLPEVFRAQLFALTGVLPERQKVMFKGAILKDS-WGATILMMGTKEELPQPTEKTVFMEDMDDSEISTALKLPTGLNNLGNTCYMNAVVQCFKTVPELTDLAKFTGTITGALRDLYRSMESYSTAPIVLLQALHTMFPRFAEKGEHGQQDANECWTEMMRMLQNLIDQLFGGKLVALQCTESEEESTEDFLQLSCFIS-NEVKYLIAGLKLRM-QETITKMSPTLNRDASYKTSKISRLPAYLTINLVRFYYKERESVNAKILKNVVFPMMLDVYELCSTDLQQKLSPQREKFKKWDNEFSFSEDGSNNSGFYQLQAVLTHKGRSSSSGHYVGWVRREWFKCDDDVVVSEEEILKLSGGGDWHVAYVLLYGPRVLAGLINEKLPKELLLKIFSFLDIVSLCRCAQVSKEWNVLAMDGSNWQNIDLFSFQRDVTYDVVSYIAQRCGGFLRRISLRGCQNVPDQALSVFAQYCHNIEQVLTNCHKLSDDSVVSLSMSLHVDSCVELTDRSLRFF----NRLRVIDISWCRKITGQGIGTIAG--DQLLRFTAKGCLDNEAIIKLATKLQVLNLQCCSFLTDSAVIAVAQNCPDLRHLCVSGCSLLTDASPQALAGGCLHTLEMANCQRCGDAGLAPLLKACHDLRRLDLEECNLITDSTLNHVAAFCPLMEQLTLSHCDQITDQGVHKLAVQCIEIDNCPFISDTSLEYLADHLRRVELYDCQLITQDAIGKFQPEVRLHTYFAPATPRQRYCRCCVIVSSKAARVFCPDHPGANLIEDYRAGDMICPQCGLVVGDRIVDVGTEWRVFQNEKSSNDPTRVGAAENPLLGGSDLSTIIGRTGDASDESGNAKYANRKTMSASDRALIGAFREISAMGDRINLPKTIMDRSNLLFKQVHDGRSLKGRSNDAIASACLYIACRQEGVPRTFKEICAVSKVSKKEIGRCFKLILKALETSVELITTGDFMSRFCSNLALPPSVQKAATHIARKAVEMDIVAGRSPISVAAAAIYMASQASAEKKSQKDIGDIAGVAEVTIRQSYKQMYPKAAQLFPEDFKPANESPWVEKYRPEKFTEIVGNEETVARLEVFSRQGNMPNIILCGPPGVGKTTTILCLARLLLGNSFKDAVLELNASNDRGIDVVRNKIKMFAQTKI---------IILDEADSMTEGAQQALRRTMENYSKTTRFALACNTSDKIIEPIQSRCAVVRFGKLNDAQILAKVIDVCRKENISYTEDGLEAIVYTAQGDMRQAIGNLQSTHVGFGHVNAKNVFKVCDEPHPLIIKEIIEFCSKGDIDEAYTRMKTLYSLGYAAEDIISNMFRVTKSHGLAEFVKLEFVKQIGLTHMTILQGLGSLLQLSSLLANLCLIVQDKKYMKKEHSLVKPYQGSGMNMPNWDFTGTTMVSSNYIRLTRDSQSQQGSIWNKVPWEIQIQFKVYGSGKDLYGDGFAIWYTKDPLQPGPVFGSRDFQGLGIFLDTYANQNGHHNHGHPYISAMVNNGSLSYDHDRDGTHTELAGCEAKFRNSEYDTSVSIRYEHDTLVVSTDIMGKKEWKECFRVSGVRLPTKYHFGVSAATGDLSDNHDVIGIKVFELDEAREFILPQAAPHRDHIDDAMSGTKFFFVVLFSMLFLMFYFYQKHQENARKRFYRDRDAFDEIRDKNCRIQDINELFLPNDTVTHVPNIKQLNIDPVFPNRTNLLHIHNMAISKAFFFSFILQRAKDDEPGFMYYFMSVIADVAANRFINASAIYYAPNMSFTPSYKGFFNKTMPLFAPRAFRSDDFNDPYHLEGTSTLNTIEAIDLGAISLNYSSDQYRINEWYSAWLPDLTKRQDSKTTYTVQITGNNDTFVWHGPPAGNDNPGPVKWVRPYFDCTRSDKWVYGATSPIPDIYPRHTQWRHIEIPRYVAVSVMELDFERIDINQCPFGPGN-PRPNYFAGTSRCKNDTTDCEPVHGYGFRRGGYQCRCKPGFRRPRVVRNPYHGELIERASKHEYENGYQCDKIGYIAVLTQTLNNYMAIGTRIDPLLGGDVVYGKEVQLENEARMAVRLANFVSGFLQIVDPKDLFAEFRVPDKSLTADQMIGEVMSIVIGDQKVVGAGIYFDYKAFFGPY--AWRKGRNERKYFVDDTTKIRIRYNSTGIKYDHFPLQYKAADVGYWTSPYFDCGGYHNSWMVTYAAPFFGWDSLRARLQFKGVVAVIELEQLEINQCNAFENTHKCDRRSSRCVPILGRGFQGGYKCECNQGYEYPYNDPITYFDGQIVEAPSRFERMTHVSGTDKSKYGGRFMVTALPGDGIGPELIGYVKEVFRYGGVPVDFEEVHLDSSRDDVDLLEQAIIAVKRNGVALKGNIETRHNDPNCKSRNVELRLRLGLFANIVHVTSQPGIETRHSGIDIVLIRQNTEGEYSCEEHMSIKGVVESLKVITQSKSDEIARYAFEWAKNNGRKKITCVHKANIMKLSDGLFLRCCTEISKEYPELEFDNIIIDNCSMQLVSNPKQFDVLLLPNLYGNILTNLACGITGGPGIASGRNYGREYAVFETGTRNTGKSIAGKNIANPIAMMNAGVDLLYHLNLREHAEVIATAIDKTINVDKIHTPDLGGQATTTDVVQNIIKEVQKHAMTYSGSSNEVILRDPPTDGISAVKFGQTSNQFLVASSWDGFIRLYDIQGERCRAKFDLGGPVLDTCFQGSSHVWSAGVHRSVRLFDINQGTELKAGSHEDTVRCIEYASDVSQIVSGGWDGAVKLWDPRKPIGPSSHSQDNKVYAIAIAGERIIVGTANRKVLIWDLRNMAFVLQKRDSSLKFQTRAIKAFPDKTGYVLSSIEGRVAVEYLDPSPEAQKKKYAFKCHRMKDSTKIEHIYPVNAIAFHTVHGTFATGGSDGFVNVWDGRNKKRLCQFHKFPTSISSLAFSPDGSALAIAASFQHEYRLEQNPPADQIYIRHVCLDQLKELTIVVADTGDFEAMKEYKPTDATTNPSLILQAAKLTQYQALIDKAVSYGRGPCEQLEEAMDKLFVLFGNEILKIIPGRVSTEVDARLSFDKDASINKAVKLISLYKELGVPKERVLIKLASTWEGIEAARILEKEHGIHCNMTLLFNFTQAIACAEAGVTLISPFVGRILDWYTANTDKKAFEPLEDPGVKSVTRIYNYYKKFGYKTVVMGASFRNTGEVKALAGCDLLTISPGLLKELANSNELVPHYLKAENATSIELEKISVDEKRFRWDMNEDQMATDKLSDGIRKFAADARKLEALIQEKLKMGDCVCRPLATVYAEDNEQSGTNVSMPAVFRVPIRPDLISFVHHQLLKNKRTPYAVSKEAGHQTSAESWGTGRAVARIPRVRGGGTHRAGQGAFGNMCRGGRMFAPTKTWRRWHRRVNVTQRRHAAASAISASGVTALVMAKGHAIERVNEVPLVVSDKVQEYKKTKQAVALLKSVKAWDDVEKVYKSKRQRPGKGKKRNRRYKKKCGPLVIYEKDNGIVRAFRNIPGVDTCDVNALSLFKLAPGGHAGRFIVWTESAFRKLNDIFGTFTKTSKVKKGYKLPRPMMTITDLGRLFKSEEIRGALRPKKSIIISKKNKPNPLKKIHLLGRLNPYALVEKRKTILAQQEQKSQRIQKKAALEKVAALKKKRLQSPFPKNRKLNQGAVLKKMLDAIKDLINEGNYDDIVSIYLHQAMDNSHVSLVALNLRADGFDKFRCDRNLSMGMNLTSMAKILKCAENNDIITLKAQDDADTVTFVFESQNQDKVSDFEMKLMNLDSEHLGIPETDYSVVVKMPSAEFQRICRDLSQIGDSVQLTCTKDGIRFSASGDLGTGNIQLSQTADVEKEEEAVIIDMQEAVTLTFALKYLNSFTKATPLSTQVCLSMSADVPLVVE------------------RNNVKVAVLGASGGIGQPLSLLLKQHPGISYLSLYDIAHTPGVAADLSHINTGSKVKGFVGNDQLKAALEGIEIVVIPAGVPRKPGMTRDDLFNTNASIVRDLADACAQTCPKAMLAIISNPVNSTVPIASETFKKRGVYDPKRIFGVTTLDVVRANTFIAEAKGLDPVSMSVPVVGGHAGITIIPLVSQASPKVDFPQDQLEKLTKRIQEAGTEVVQAKAGAGSATLSMAFAGARFVFSLVSAIQGKNIVECAYVKSDIGDAGFFSTPLLLGKNGMEKNLGLGKLSKFEEKMVTDAMDELKKSVKKGIDFANKEYEWLLKFEVNDIVEQLVIAECSKRFPL-ARSDKFIM-QSKVVATLTGDSISHADISLRL-PKHSQRTIVQNDAQWKLQQIQDAGNHLMQAMNLLRFKFTSGQEVRNLMSDVMTCVGRGRACLVVPKKRTIEEIMQSRNMKSLQPPLPNDVAVSFYIQSYKLVFAVYHV-QKDPQKF-DAECSVPWLSEVLVLFTVALQLCQQLKDKVEVFQYNDFLPMFIPLFLEFNAPISEISDLLGVCDVCFKDAESVL--NGFVSILVCLFCERLESATVQPVAFN--VLYNFYEGLRVDVFCSLLRVAAGSVPEVFQDVSVTKKWLPVDKARQVYRNIHSALSDMPLRVMVELLSTYQDHDAAEANQDAIKCIAFAISDPNTYLMDHLIPLKPIKALENQPINELLKIFVYGKLSEYREFYRKHKEVV-EQLGLDHEKNVEKMRYLTFMYLAEKNQEISFDDIKREVEI--DDVEGFTINVLRTKLVTAKVNQPNQKVIVVSTMHRSFMKNEWEQLREILRLEKVEQSTQQFLEYLG-TR-RKKEVEELAEEAKAIDEGEERAVIEESPQFKELVNILVEWINDELAKHRII--VKNIEEDLYDGQILHKLLEQLTNSRIDVVEMTQNEEGQREKLKVVLERASQALGLKWSVDAVHSKNVVAIVHLLVALARHFRAPVRARLPENVVVSVVSVTKR-EQLTRTYDEYGMKVE-RDAFDQLFDHAPDKLTVVKKSLLTFVNKHLNKINIKDLD-KQFHDGFYLALLMGLLEGYFIPLYLTLTP----------------------NNKVNNVAFAFGLMKDAGLKPKARPEDIVNYDLKSTLRVLYNIFMQLSHRSTRIIKDAERDLRELLSVPPTYKVLFMQGGGTGQFAAIPLNLCPADYLVTGTWSRKAAEEAKQYVKYTRVPPASEWNLSPDAAYFYYCDNETIHGVEFPIVCDMSSNILTRPVDISKFGVIFAGAQKNLGPAGVTIVIVREDLVAVASVCPSILAYKVFAENDSLYHTPPTYAIYLLLVLKWIKSEGGVSGMARSAAKSQAIYDLIDQSGGFYCRSRTNIPFRI----RSNDALEKKFLKEAEMIQLKGHRSIGGIRASVFNAMDVVQAMKLYRERPEWQDVTPVLKISYTNTFRDCFGYLRAVISGELSERVFELTTTCADENPACYTVWVLRRRLLEHLKKDLNEEMEFMSRQIFDNQKNYQVWYHRQRLVQLRELDFIERVLDAKNYHAWQYRQWLLKTFNLWSKELNFCSSMLNEDIRNNSAWNQRYFVLKNTTGFVVEEEIKFTLDKISCNESAWNYLGGILNIYLYATLLSEALEIDIPRSPYWTFKQKLLAIIIDRFEELLEFVPHLSRFLTAQGVAYRFIVINQGDRFRFNRGALINIGYLVSRAQCDYMVMHDVDLLPLNPKLSYRFPQGDNVHIAAPHLHPKYHYSTFVGGILLMRHAVFARLNGLSNKYWGWGLEDDEFYVRAKEANVRFERPTDIGTGINDTFRHIHDARRRPRDMVRIGSQREESRKRDRVTGLNNVVFQHQGLYQVRIGEVPVDVHNVHLHCDLTITPWCEKPRMSPGETITTDGYMRGHGGLLSAVAGVVEKVNKLITVRPLKTRYNPEVGDVVVGRIVQVC--QKLWKVDVGARLHAALHLHSVNLPGGELRRKSIEDELMMSQYFIDGDLVSAEVQNVGVDGAVSLHTRNLKYGKLGQGALVTVSPSLIKRCKHLHNLA-NGVHLIIGLNGFIWVTSSKFTREAICRTRNVILCLAIHNIMLYDTSIVHAYDIS-VAQLTRHRADKLVGSWLLTCSGMALGAVFLGGLTRLTKSGLSMVDWHPLNEGRPRTAEWEAEFAKYQQFPEYKVRNKDMTLEQFKSIYWMEYIHRMWGRTVGAAFYIPAAAFWARGYFNRGMKKQIIYLGTLLAAQGLMGWYMVRSGLEEKPRVSNLRLAVHLGAAFFFYGLLFRAALHRLIPSKPLVRFSWATGLVFTTALSGALVAGIEAGLVYNSFPKMADSWIPSDILAFPKWKNFVENPTTVQFDHRLLGETVLTALYIYSRKVPLPPRARLATHAMLAAWLQVGLGITTLLTYVPTPVAVSHQVGALTLLTTLLWLTHELKLIRR-L--------------------------TEKNACQWSKDKLHSLFSDLEIND-SIMSVIKELKKCEGEATANNRKAKLIFFYEWELELCAGS-VVKGRVEIPNLSDENDIHEVSVNVTLVDEKIKGMMRSKGTEVIRSKLDEYVSSLKMDFSQGLILPTKDSGANSTATKGKKISTKELIMEDFKCRAEELYRAFTMVQAFTKGAAGGHFQMLDTNVSGKFLKLNELEFEWRFKSWPAEHYSFVITIEEVS---------------------------------------MDPETFLEIANHVSKLKMYPYFELAHCIVTLLYLREDLGTGSQLFSRKHPLSCWVSSMFSIYAGGIFAALLLGEPVLAVLKSNQSLILATACWYLIFYSPFDIVYKFCKILPIKLAIALAKEVTRAKKVHDGVHHAAKIYPSAYIIMVIIGVVKGNGTSFLKVFERLLRGFWTPQAMEIMQPSFATKACVIASLVFVVDKKTDLISAPHSLVYFGVVVFFVYFKLSSVVLGLHDPFIPFENLACAIFFGGIWDAISRAIKDTAAKKKEMFRPDGIVQRRTADGCDKDKETRLTLMEEVLLLGLKEKEGYTSFWNDCISSGLRGCILVELGIRGRIDLEAAGMRRKSLLMRKVVVKNDAPVGDVILDEALKHIKETATPETLQNWVDYLSGETWNPLKLRYQLRNVRERLAKGLVEKGILTTEQQNFLLFNMTTHPLVDQNVKDKLIKRVQDSVLAKWVNDVHRMERRHLALLLLSHASDVLENAFNPLSDEEYEMAMRRVRDLLDMDFEAECAKSQTCDIMWGVFAAFVKMADGVPTFKCVLVGDGGTGKTTFVKRHLTGEFEKKYVATLGVEVHPIVFHTNRGAIRFNVWDTAGQEKFGGLRDGYYIQAHCAIMMFDVTSRITYKNVPNWHRDLVRVCENIPIVLVGNKVDVKDRKVKAKSIVFHRKKNLQYYDISAKSNYNFEKPFLWLARKLIGDANLEFVAMPALAPPEVQMDPEWQSKLENDMKEAQNIVLPDEEDDDLLAAQITPILRESKFRESGMLTPEEFVLAGDHLVATCPTWAWAKG-DKSYLPEDKQFLVTKNVPCSKRCRDMEEKIIEGD-EGWVDTHDEDNEDDEGVDMDDFLDEDLETAEILATRTYDLNITYDNYYRTPRLWLTGYDEKMKPLTTEQIYEDISQDFVKKTVTVENHPHIEGVPQASVHPCRHAQAMKNLIQTVEEGGGLEVHMYLIVFLKFVQAVIPTIEYDYTANFNMLSNLERKLPYDKLSANIDIVKKRLNRPFTLSEKVLYSHLDQPQSEEIVRGTSYLKLRPDRVAMQDATAQMAMLQFISSGLPKVAVPSTIHCDHLIEAQLGGDKDLSRAKDLNKEVYNFLATAGSKYGVGFWKPGSGIIHQIILENYAFPGLLMIGTDSHTPNGGGLGGLCVGVGGADAVDVMAGLPWELKCPKVIGVHLTGKMSGWTSSKDVITKLAGILTVKGGTGAIVEYFGPGVQSISCTGMGTICNMGAEIGATTSVFPFNSRMADYLASTNRRAIADAAEQVKDLLSADAGCKYDQVIEINLDSLEPHVNGPFTPDAAHPISKLGQTAKEKGWPLDVKVGLIGSCTNSSYEDMSRSAMLAKQALDHGVKSKSLFTVTPGSEQIRATIERDGQAKVLKEFGGMVLANACGPCIGQWDRKDIKKGEKNTIVTSYNRNFTSRNDANPQTHAFVTSPEMVTALAIAGRLDFNPLTDELTGSDGKKFKLKAPVGDELPRAGFDPGQDTYQAPPADGSNVKVDVDPKSQRLQLLSPFSKWDGKDLIDMVVLLKAKGKCTTDHISAAGPWLKYRGHLDNISNNMFIGAIPEESGEANKIQNRVSGSWGTVPEIARDYKAKGQPWVVIGDENYGEGSSREHAALEPRHLGGRAVIVKSFARIHETNLKKQGLLPLTFADPSDYDKIKSDDKISIVGLNGFAPGMESRGGMFKNTFQSGFLSILYSLGSKPLQIWDKKVRNGHIKRITDNDIQSLVLEIVGSNVSTTFITCPADPRETLGIRLPYIILIVKNLKKYFTFEVQILDDKNIKRRFRASNFQSTTRVKPFICTMPMRLDEGWNQIQFNLADFTRRAYGTNYVQTLRVQIHANCRLRRVYFADRLYAEDELPAEFKLYLPVQERCINSSYQYMQKARADIKRVTQQYRGLAETFIFNNGSSKELVCLDGTIPVRYKYNIPIRIWVLDIHPYHAPFCYVCPTPTMQIKTSQYVDESGRVYLPYLHDWNRNS-SDLIGVIQVMIMIFGEQPPVFSKSPNPYPSSNTLSITDQHIRISLLSAVESRITDRALEKSKAEEEVLRKTNEELQAGKQKLDRYMSDMERDREMESEKSEQLKQLDVDNAVTPTAPLYRQLLQAYAEESAVEDAIYYLGEGLRKGVIDLDTFLKHVREQSRKQFMLRALMQKCRQKAGLPMGRKFYVGGNWKLNGTKQSIQVICDRLKTSQSETEVCVGVPAPYIQFVRDLLPPSIHVAGQNCYKASSGAFTGELSVDMIRDCGGDSVILGHSERRNVFGENDQLIAEKCAFALQGGLVVIACIGELLEEREAGKTEEVVFRQTKAYADLIKDWKNVVIAYEPVWAIGTGKTATPEQAQEVHAKLREWLSKNVSEEVGLNIRIIYGGSVTAANCKELAQKPDVDGFLVGGASLKPEFVDIVNAKKMATALSKKRRFVADGVFNAELNEFLRRELAENGYSGVEVRNGAMKTDIIIMATRTQDVLGEKGRKIRELTAVVQKRFNFKEGTVNLFAEKVSARGLCAITQCESLRYKLIGGLAVRRACYSVLRCIMEAEAMGCEVVVSGKLRGQRAKSMKFVEGLMIHSGDPTNHYVETAVRHVLLKQGVLGIKVKIMHPFDAQGKRGPALMLPDKVTVMVPKYEDDNVETRSDNKDNAASKIEQLKQWSLSTYKCTRQILAEKMGKGTRTVDGELEANIELLRETHQKYLNILRLAKLLTTHFNTVATQAALGECFSDLAQK---ELQQEFLYNAETQKNLSKNGDTLLGALNFFVSSLSTLCNKTIEDTLITIRHYENARLEFDAYRCEARANFKEKYERLRGDVQIKMKFLHENKVKVMHKQLLLLHNAVSAY-FSGNQSSLEATLKQFNISWLEQ-MVDLVLDRDIRIWVFLPIVVITFLVGIVRHYVSILLTSSRKAELQQVYDSQALIRVRYLRENGKYLPARGFFMRKHFFNDEETGWLK-TQKRAPPMNNPMSDPSMMSEMLKGNLTNVLPMIVIGGWINWTFSGFLTTKVPFPLTLRFKPMLQRGIELVSLDASWVSSASWYFLNVFGLRSIYTLVLGEDNAADSTRAMQDSMVPQAAAMPQDPKAAFKAEWEALEVVDHKWALTGIEEMICYKGAASEAGRAMQILKRRERQKEEVELKRQKIEQEMRVS-MGDKFSSHFDAVEAQIKSATVGLVTLNEMKAKQQDAVKEREKRLAQKELEEKQRAEDQKKAQKEKQKKAIQALSFNLDEIKVKKNPDVDTSFLPDREREEKERMIREELRQEWKDRQRALKEENIQITFSYWDGSGHRRVVDMKKGNSIYQFLQRCLDSLRKEFYELRVVSADQLMYIKEDLIIPHHYTFYDFIVTKARGKSGPLFAFDANEDIRMTSDASKEKEESHAGKVLLRSWYERNKHIFPASRWEPYDPTK-------------KPMVPETLLKQRKHNAELRQQRLLAAAAKKKAARARRVLAFRRAEQYVREYRRIETSEKNNRLVAKVNGNFFVPDEPKVAIVIRIRGITGVSPKPRKVMQLFRLRQINNAMFVRLNKATINMLRLAEPYLAWGYPNLKTVRDLIYKRGFGRVNGRRVPLIDNSIIEEKLGKYGIICMEDLVHEIYTVGPNFKQVVNFLWHFKLNNPKGGWRKKTTHFVEGGDYGNRETLINSLLRKMIVRLGAEDLWKSVTSVSNAGRKRGRASGHSKKASKDLNRGQKIGSGRINMLWPGLNAPIFRGREVVERQQLPPDPEREKRLIELRDKQQNFRRIRLTPLERGWSGTRAPGRSLGPPDTPEDIDIKDFDSVVLDLRLVSNMTSHLGRVRRHKAVVAVGNGKGLLGFATGRGPDGKTALRKAKNRALLRLCHYPMFEETVLHDFFSEYGCTRIVVKQKERGYGLKCHRAIMSLCKLIGIKNLYAKVDTRNTNLLSLTRAFLIALMYVRSKFDFADEKRLHVVEFRPEQLYFPRIVASPKTVRKEDEIDPYEELDINMIVSEGRMIYARKKAEPFYTRLPGWEVHLKKTDNLKNQRQVRLRLMKKYGALKSFLNIRKPLNQIGPRKSDEKYKWTNKQRLLIFASRGITYRDRHLMNSFRTMLAHSKEECKFE--KKDINEVAEMKNCNKVMYMENRRRSDTYMWLANMQTGPTLKFLVQNVHTMEELKFSGNCLRGSRPFLSFDPAFDSHPLVKEVLAQTFGTPAYHPRSQPFFDHVFVFRLLDKRIWFRNYQIVEE-DGSLVEIGPRFCLNLVKIFDGPFSGAVIYTNPHYVAPNKMRRLAQKE--NKYMYMDVFETKNTFYRMAVGKNKGLSKGGKKGLKKKIVDPFSRKDWYDVKAPSMFSIRNVGKTLVNRTQGTKIASDGLKGRVYEVSQADLQTGEDAFRKFKLVCEEVQGRHCLTNFHGMDLTTDKLRSMVKKWQTLIEAQVDVRTTDGFVLRLFCIGFTKKAQNQVKKTCYAQHAQVRAIRRKMVEIMHREVSSSDLKEVVNKLIPEAIGKDIEKNCIMIYPLHDVHIRKVKVLKKPKFDMGKLLEMHGEGKGSGGGDGMAVDRPDNYEPPVLEDVVKIGIIGGSGLE-DPELLKEDTPYGKPSDA-LISGKIDGIDVVILSRHGRRHTINPSNVNYRANLFALK-QEGCSHILVTTACGSLKEEVRPGNFTPNTFIDRTRIQTFYVCHLPMTKPFLSKLLSEHPKGTVVCIEGPRFSTVAESNVFRQWGADLVNMTVVPEVVLAHELGIPYAALAITTDYDCWR-E--ETVDVEKVSTLKLAADGACRILRATLPKVLKIAVMMRVLRHEEFEKGCLAACNGRYDGFWSKTMVGYGSEDDHFAMELIYNYGVDKYKKGNEFKGIVIQMSNVLKRAQENRWTVQQENDKSYVEAPGGFKFYVEEDSS-ERKDPVRQVIYSCTNVEKTRRFWVTMLGCEVVESGDDFLEVAYDKSKTSLRFEKIFEPIDRGEAYGRVAFACPRNQLPEIEAQVKNADGTVITPLVSLETPGKASVEVVILGDPDGHEICFVGDEGFHELSKEDPEASQVLQEKMK--EYDAYVEKVQRRDRHFRLPPLPTPADLLRFYRLRATKQLSQNFLLDPICSKFVRSSGHVIEVGPGPGCLTRPIFEQGAESVVVIEKDKRFMAALELLANATN-NKLKIVHGDVLNSKLEDLI-PAEKAKPWEGIHLIGNLPFSISTILIIKWLHMISRKSSAWQYGRVRMTLSFQKEVAERITSPERCRLSVIAQGCYVKNGGAFVPPPEVDVNIVRFVPVQPIFQPFDLVEKVLRCLFNKTLKIELAQELLQRSDVDPPVVISVEDYSKICDAYAGLCKEPGLYEYDFRSPPMVPIVIEQTGRGERAYDIYSRLLKERIICLMGSIDDDIASLVVAQLLFLQSESSKKPIHMYINSPGGSVTAGLGIYDTMQYVLPPIATWCVGQAASAASLLLAAGEPGMRHSLPNSRIMVHQPSGGVSGQATDIQIHAEEILYLKRTVNLLYAKHTNQALETIESAMERDRFMNPEQAKEFGLIDTVLEQPPMRIERPVGTWLTLLPLWALTTAAPAGSLPDAALFCTGALLMRGFGCTINDMWDRDIDRQVERTRQRPLAAGRLSRWDALWFAGGQGLACLVLLHFNWNTVMLGLASVGLVVIYPVMKRFTYWPQAILAVVFNWGALLGFSATALPMYAGAFSWTLIYDTIYAHQDKRDDLLVGMKSTALRFGARTPLWLGAFSTMTTGLMGWPYYAAITLKIDNIGLLVGCIGGSLLKVARVDQPKTRKGSKILKAREPQVIENPKTSFVKGNNINQRTTQILKDIYALKKSESVFYQQRNPFEDPSPLEKLGKKDTSLFAFGSHNKKRPQNIVLGRTFDRMIIDQFEFGVENFKSLQEFKVPKIGVMLKPILVFSGESWQEMKRLKNFFIDFFKGDYIAVTGLEHVISFTILDILLRSYKVQLKKSGQKSPR-VEIEEIGPRMDLKIRRNKIASDDLFKQALRRPRELKVKRKKNMEQNDLGTSLGRVHMERQDFKLQTRKMKGLKVQILEALDDNYMYLVITREAAVVDPVNPEKVLRAVKVKLTTVLVTHHHWDHAGGNEKLLVFGGDDRIKELTDKDNVNITIGSLAQSLFTPCHTTGHVCYFIVFTGDTLFTSGCGKFFEGTAQQMQAAMLGSLPPETRVYNGHEYTVNNLKYAQHVEPDNQVKEKLLWAQKKPTIPSTIAEEKSINPFMRTSVQKHTDPVSTMNALRQEKDRFRMNRLFGKGKAKEPPPNLSNCIEVLDQRANNMDEKIKKLDAELFKYKEQMSKMREGPPKDLVKQKALRILKQKKMYESQRDNLMQQSFNMEQANFATQQLKDTKTTMEAMRLGVKEMKHEYKKVNLNEIEDLQDDLEDMLEQANEVQEALGRSYGMPEMDDEELEAELTMLNDEIALEDTSYLEQVRMVSRTRGKATLPDLPYDYNALEPVICAEIMTLHHSKHHNAYVTNYNIAAEKLQEAIQKNDTNAQIALQNAIRFNGGGHINHSIFWQNLCNPKSGEPSAELLAAIKKDFGSFEAMKDKMSAAAVAVQGSGWAWLGLNKANKQLQVAVCPNQDPLEASTGLVPLFGIDVWEHAYYIQYKNVRPDYVKAIWKVANWKDISQRFIKA-DDHIQHDVIKEALESGRDLREYSRSVEQQLKGSEDEAIRDYMHNCRDIAALHGEIASCDAILQRMESILRGFQNDLGSISSEIQSLQRQSVAMNLQLKNRQAVRGELSQFVDDFIVPEATINVILDCPVIDEDFLTQLALLDQKISFVKVQSFKEASSCQDVKDILDKLKVHAVTKIREWLLQKVFSFRKANANFQLAQNTMLKHRLFFQFLATHEREVAKEIREEYVDTISKVYLSYFKAYQTRLMKLQFEDVPDKDDLMGVDDTPKWGLFNKPSLKNRSTIFTLGSRNAVLGADLEAPVIVPHASAKSEKHYPFEQLFRSTQYALCDNAAREYLFVSEFFLLTKQGAVDMFDNILEKSMSLFAKHTESFVAECFDSIALFLCIHIIRKLKVVMHSRNVPVMDRYWDLLVKIIFPRFETILRMNIASIRDCDPSKLGSIDNRPHYITRRYAEFSAAIVSINENHPDERVTQLLGQLQIEVENFVLRMAAEFNGRKDQLIFLINNYDMMLGVLQQRTHEDSKETTNFRTLLTARQSEYVEQILTVHFGGMMTFIKESEYYVEKGQINELEKESHKVATLVRNFNSGWKKAIDDMNGDIMKTFTNFKCGTSILQEALKQLLQYYHRFNKVISQPPLCSLPVRSELINIHHLMVDIKKYKATFGYFIVVDAHSEECFHDRVVKGTKMGLTFEVAEGGFLDIDVKITGPDEKVVYNGERESSNKYTFAAYMDGVYKYCFSNAMSTMTPKTVMFTMDIGEEPKADGK---DGGENKLEDMISELHTAMTGVKHEQEYMMIRDRIHRSISESTNSRVVIWAFFENLIIIAMTLGQVYYLKRIFEVRRVVMRLSHILKLPKDYANLPKSYVKRAMAQVEWRTPNGRQFRRAVVQKPYTMNRPWTNEFMTNPPGVAIPVQVMFRGDRVEVMVGDDKGKQGTVNYIVPERNWVTVEGLNTEELPLLMGTQVKLVDPVDEKATDVEWRYTESGERVRVSVRTGRIIPIPLAYETIDYKYVDKAKDTSATELEKITFVP-KLATFEMDIMEQHGIQDDRVPHRTFWYVEQKMELLLWLSRLACTMFTVSYLLGNPYSLYQKALICNGVTSALRLHQRAPTVRLNAEFLTTILVEDSCHYLIYSLIF-LPIS-MVLLPPMLFAVLHSLQLLERAGILLQ---SRVRVFRLIAMTEIFLMPTVIIGLFCGVSLMAPFMYYRFLKLRYASMRNPYNRNVFYELKLHANQLAAPNCPMIIQRVRFIESRAGLAIAVITVYLSFPEMRPEEQPYVKLPTSLEDAKNLGRVLSRYTDDAVVLAFFCTYIFLQSFAIPGSIFLSFLSGFLFPFLLAIFLVCLCSAIGASLSYLISYSVGSRLILAWIPNMVYYIIFLRITPFLPNWFINLASPIVDVHIVPFFIGTFIGVAPPSILAIRAGISLQQLASALTWENIGLLTGFAAFSLLPVLLKLKNKFEEVQDKLTRIAIVSIDKCKPKRCRQECKKSCPVVRMGKLCIEVTPNDRIATISENLCIGCGICTKKCPFEAIQIINLPSNLERDTTHRYSANSFKLHRLPTPRPGEVLGLVGTNGIGKSTALKILAGKLKPNLGRYNDPPDWTEILGYFRGSELQNYFTRILEDHLKAVIKPQYVDQIPKAVKGTVRELLDRRNDLGKEDYLCELLELVNVMDRQIADLSGGELQRFATAMVCIQKGDIFMFDEPSSYLDVKQRLKSAQAIRGQIEATKYVIVVEHDLSVLDYLSDFICCLYGTPGCYGVVTMPFSVREGINIFLDGFVPTENLRFRESSLVFKVSDNTDEDVKRLARYEYPSMVKKMGDFELKVQGGSFTDSEIIVMLGENGTGKTTLIRMLAGRLKPDGDEDVPTLNISYKPQKISPKSTGTVRFLLHEKIRDAYQHPQFVADVMKPLLIDNIIDQEVQNLSGGELQRVALALCLGKPADVYLIDEPSAYLDSEQRLAAAKVIKRFILHAKKTGFVVEHDFIMATYLSDRVIVFDGKPSVSTTANMPQSLLVGMNRFLELLNITFRRDPNNFRPRINKLNSVKDSEQKKNGTFFFLEDEEDGEDLFGPELDRYDAAEEEMRRRDRGRLPRGLESIENLEDMKGHTVKEWVTQGPKTEIFNRFKNFLRTYKEKIRAMEQNRMSLEVDYTLAQSEQVLAFFLPEAPAEVLPIFDEAAKDIVVGMFPHYGRIHHEIRVRITDLPILEEIRTLRKIHIDQLIRTSGVVTSTTGVLPQLRMVKYDCVKCKYILGPFVQSQEVKPSSCPECQSTGPFSVNVAQTIFQDYQRVTIQESPGKVNAGRLPRSKDAILLNDLCDSCKPGDEIEITGIYSNKFEGSLNKANGFPVFATVIIANHILKKDTDEDVKEVVKLSKEELAERIIASIGPSIYGHDDIKRAIALSLFGGVSKNPGQKHRIRGDINVLLCGDPGTAKSQFLKYVQQIAPRAVYTTGQGATAVGLTAYVSPVTRDWTLEAGALVLADKGVCLIDEFDKMNDADRTSIHEAMEQQTISIAKAGIVTSLRARCTIIAAANPIGGRYDTFHQNVNLSDPILSRFDVLCVVRDERLARFVVDSHSRHHPISQDLLQKYILYAREKIEPKLDQDKISQLYSDLRRESMATGSMPITIRHLESIIRLAESHARMHLREHVDVNTAIRVMLDSFVTQKFSVMRMGLLDDVKEIFGADTLYEVLGVKKSVRSDLLKKAYRQKSLLCHPDKANEENKEEFTKKFQILCKCYEILCDEEKRKIYDETGSV--DDSFDEKKDWKSFWRTLFPTVTDSQIQDFFDKYHGSEQEREDLKRAYEKTKGDMNKIAECFIGYDEDRLTLLLAIAIGFLLLGKVGTKKLRKLEEKAEKRRLRELELQEREERAQKQAEDEYRKKVEQKKEDEQKQKEEEERRQKEEKERREHEEYLKMKAAFAVEEEGFDQEDDP-NSSQKLHEFITYINEHKVVQLENLAAQFRMKTQDCIDRVQRLLEDESLCGVIDDRGKFIAITRDELDEVARFIKVRGRVSIQELVENSNRLINLASDIMKQQEEAKAAALKHVVTMLQIPDQLDKVEQHRKRVQRKKASVEAMLKTAVQSQLEGVRTGILLLAAARDDVNDIQKDEADTIYDLSKLVQLQDVREESFRHSQTGTLMEHLKHIFNVPGSITRTQDLIQDGNLLLAHKLSDLECSRDELLYELHKQANNQASDRAMLKQYFADVERLSDELAKQLWLILKRTLNTVRKEPQVIVTALRLIMREEWAALKRQQST-GFLPPGRPKLWRKKAIETLEQSVAERLEGNQIEVRSENNMWLVRHLEVTRQLIIEDLKTVKHHCTPCFPPCFDIFNEVRMTHNCLSQRLQTIISGLVDSEYIHVLQWLNTYNSRDLMGHPDLHVDVALLPDTIERLMERYLAGLRTKFEEWLRNALSDHKDWPEQDSNGYYRTETPMLIYQMITQHVEVARTVDLVSRVLRLAMNRMDYFLSQYNQLVTDYNFEDRSYTAYMIAIANNAVNMK-LRELKDNSLDYLCEEVLTDIKVMPDIMTETVTVTLADYGGDYIEKEVAASYVKAIC--EKRMSFRNYEERRAAAELEKLKKSKLDVLKLMAEVLKMKDSSLLSLEMNGSRTDAEVIAREEEAIKVDSLSVAQLKEIREKAQKHAFQAEVARMMKLIINSLYRNKEVFLRELISNASDALDKIRLLSLTDPDALKKLQDLSIRIMADKENNVLHITDTGIGMTKEDLMKNLGTIAKSGTAEFLQKVSEGSSGKDLNDLIGQFGVGFYSAFLVADRVAVASKHNDDPIQHVWESNAAEFSVADDPRGDTLKRGTTVSLYMKDEAKDFLEHDTLKKLIEKYSQFINFNIYLWSSKT-VTEEVTESEKPAEE-----ADDDEAKVEDAKEP--LKTKKVDKTVWDWELINSAKPIWTRKEKEVSDEEYNEFYKAVTRDSQNPLAKTHFTAEGELTFKSLLFVPSKQPQDSFNRYGQKTDHIKLYVRRVFITDDFQDMLPNYLSFLRGVVDSDDLPLNVSRENLQQHKLLKVIKKKLVRKALEMFRKISEEEYVKFWKEYSTNIKLGVIEDSANRSRLAKLLRFPSSLDSTDKLVSLGDYVQRMKEKQTAIYYIAGNGMDEVKKSPFVERLLKRGYEVLYLTEPVDEYAISSLTEFEGKKFQNVAKEGLSLDD---NKDIREAMEKEFEPLTKWLSETALKDKISKAVISERLVETPMALVASQFGWTGNMERIVAAQTHMKENDPQKSFYMTQKKTLEINPRHPLIKELLRRVDDSPSDEMAKYLTEMMFETATLRSGFQLNDNAQFASNVERMLRKMMGVSEDAQVDAELGRRGEVKPLSAQEMKMIVISEIIQELVQAHNDKRDVNLNRVKYDVSARYGLKSQPKLVDIIAAIPPQFKQILLPKLKAKPVRTASGIAVVAVMCKPHRCPHINYTGNICVYCPGGPDSDFEYSTQSYTGYEPTSMRAIRARYDPFLQTRHRVEQLKQLGHDVDKIEFIVMGGTFMSLPESYRDYFIRNLHDALSGHTSANVDEAVKYSERSKTKCIGITIETRPDYCLQRHLTDMLRYGCTRLEIGVQSVYEDVARDTNRGHTVQAVCETFHIAKDCGFKVVTHMMPDLPNVDFERDVLQFVELFKNPSFRMDGLKIYPTLVIRGTGLYELWKTGRYKSYPPALLVDLIAKIFSLVPPWVRIYRVQRDIPMPLVSSGVENGNLRELALARMKDLGLVCRDIRTREVGIQEIHHKVVPYHIELVRRDYVANGGWETFLAYEDPQQDILIGLLRLRKCTEQTYRPELLGQCSIVREHVYGSVVPVHSRDPSKFQHQGFGTLLMEEAERIAREEHCSKKIAVISGVGTRNYYRKLGYELDGPYMSR

'Dermanyssus_gallinae' PGSKVNTLSQEVMASAVLISGKKGCFIAGADITMLEKCKTEAKKLKPVVAAIMGSCLGGGLETALACRYR-IAVEDPKTTLALPEVMLGILPGGGGTQRLPLYVQLPTALTMMLTGQNMRADKAKKAGLVDVTVKPLGPGLYLEEVAARDIASGKLKIRVRPLSERLVRNT-IFNKAKAQVMKNTNGLYPAPLRILQVVRTGIEKQNFAELCTPECKSLMGLYHGQVQCKKNAFGQPTQNVAVLGAGLMGAGICQVTLKDFNNVIMKDGLVRGHNQIKKKKIEKDKLMSKLLPTLDYSDMVIEAVFEDLAVKHKVVKEVEAPHCIFASNTSALPITKIAEASKRPEKVIGMHYFSPVDKMMLLEIITTDKTSKDTAAAAVDVGLRQGKVVIVVKDGPGFYTTRILAPMMSEAMVLLMEGCQVKELDKLKDFGFPVGGATLLDEVGVDVGAHIAESVFGDRKDMVSNFLGRKSGKGCYIYRPVNPLQRKYTPEQLQFRLATRFINEAIMCLQEGVLANPVEGDVGAVFGLGFPPNRGGPFQFVDTYGAAKIVDMKQFQDFQPCQLLLDHA-KDSSKKFHMVTIVIIDPAKTLKILGYTCMLYNIEASFSICVFIDFVLLASESAKLPLRTPATALTRKENAKITNIQDGAYACVSLQLNIVISSISVVSSVETDLVASKKEFFAKMKCLLQTCLIRDTIAGTLASAIYSWLYQYNKLYYGVGSNDQRISFELQSLFTLLYIYRDNRKQYTSYETSQASTRITMHALKILSGIKKVSGGALEDSMLVAGVAFKKTFSYAGFEMQPKQYTSPKIALLNIELELKAERDNAEIRVENVEEYQKIVDAEWNILYDKLAKIHASGAKVVLSRLPIGDVATQYFADRDMFCAGRVAEEDLHRTMKACGGCVLTTVQDLKNENLGSCEHFDEVQIGGERYNIFKGCPNSKTVTMILRGGAEQFIEETERSLHDAIMIVRRAVKNDAVVAGGGAIEMELSKYLRDYSRSVAGKEQLLVAAFAKALEVIPRQLCDNAGFDATNILNRLRERHAKGEKWSGVDINAEDIADNLVACVWEPAVVKVNALTAATEAACLILSVDETIKAPQSNTDPSAGRPF------------------------------------------------------------------------------------------------------------------------------------------------------------------------------------------------GLVKRAGKSVMDDNFAIVFAAMGVNMETARFFKQDFEENGSMDNVCLFLNLANDPTIERIITPRLALTTAEFLAYQCEKHVLVILTDMSSYAEALREVSAAREEVPGRRGFPGYMYTDLATIYERAGRVEGRHGSITQIPILTMPNDDITHPIPDLTGYITEGQIYVDRQLHNRQVYPPINVLPSLSRLMKSAIGEGFTRKDHADVSNQLYACYAIGKDVQAMKAVVGEEALSPEDLLYLEFLTKFEKNFISQGRYENRNIFESLDIGWNLLRIFPKEMLKRIQHSLLAEFYPRTDAKTGLFGEPTGFYVLKDNAIQQAEELVAEATSRKRKMVHIFDDLSDCLCRVADLAEFVRVGHPQGRYNDAAKHASLAISSLVEKLNTNLDLYSALRSVVENG-DISASSTEKHVERLFLFDFEQCGIHLDEQRRQEVVALNDLILHVGGYFLQNSQNPRYVPKSMVPENILLVNGLQADCSNELVRQAAYRMYLSPDDQQQKLLDQLLEARHRLAQLCGFETYAHRVLKGSIVETPENVTFLSYLSAELKPRAERDYQEMLTMKAWDIPYYTAYLSLASCMEGLDMIFHELYGVRLEVLLLITHSSRMLLIT--SSSPMGVIYCDLFERPGKPHQDCHFTIQGGRRSDGTYQTPKVVLMLSLPPPLLTPPVMDNLFHEMGHAMHSMLARTEYQHVTGTRCATDLAEVPSILMEYFASDPRVVLRFARHYETGRPMPSYMATSLEASRVVFQASETQLQVFYAFVDHEYHSKYPLSTTEILRNVQNKHFGVKYVDDTAWQLRFGHLVGYGAKYYSYLMSRAVAASFWHRVFKADPLSRSSGTYRNEVLAHGGALPPAQLVQDFLLAESLIADI----------------------------------------------------------------------------------------------------------------------------------------------------------------------------------------------------------------------RMRIALARALYVKPHILLLDEPTNHLDLDACVWLEEELKTYSRILILISHAQDFLNGVCTNIIHMNLRKLEYYGGNYDQFITTRAETLENQMKRYNWEQAQMSHMKDYIARFGHGSAKLARQAQSKEKTLAKMVAGGLTEKVVNDKTVNFYFPSCGPIPPPVIMVQNVSFRYSDDTPLIYKNLEFGMDLDTRVTLVGPNGAGKSTLLKLLCGALVPTDGIIRTHSHLKIARYHQHLHETLNFDLSALEYMLQCFPDVREKDEMRKIIGRYGLTGRQQVCPIRQLSDKQKCRVVFAWIAWQVPHMLFLDEPTNHLDKETIDALADAINNFEGGMVLVSHDFRLISQMAKEIWVCENQTVTKWAGDIKSYKQHLKTKVLKDMVLMVLADLGRRITGALRSLSTATVINQEVLDSMLKEICAALLESDINVRLVKQLRENVKAAIDIEEMAVGLNRRKVVQSAVFKELVKLVDPGVKAWQPSKGRNNVIMFVGLQGSGKTTTCTKLAYHYMKKGWKTALVCADTFRAGAFDQLKQNATKARIPFYGSYTEVDPVVIAADGVSKFKAENFEIIIVDTSGRHKQEDSLFEEMLEVSNAVSPDNVIFVMDASIGQACELQARAFKEKVDVASVIITKLDGHAKGGGALSAVAATKSPVIFIGTGEHIDDFEPFRVKPFIQKLLGLGDIEGLIDKVNELKLDENHELIEKLKHGEFTLRDMYEQFQNIMKMGPFNQIMGMIPGFSADFMSKGNEQESMARLKRLMTMMDSMTDEELDDRDGAKLFAKQQTRITRVARGSGCSTYEVHELLNQYTKFAAMVKKMGGMKGLFKGNDLARNVNPAQMNKLSAEMAKMIDPRVLQQMGGFSGIQNMMRQMNASVLTEDPAYLKLQQLYESKGKTLRLNDLFKADATRAEKYTLKLSEGPETLLLDYSKNLVDGEILAALFELAKNRQVEGMRDRMFAGDKINFTENRSVLHVALRNRSNRPIQVDGADVMPGVNAVLEHMRTFSLKVISGDWKGYTGKKITDVINIGIGGSDLGPLMLCVILGYFFVGPRVHFVSNVDGTHLFETLKKVDPETTLFIVASKTFTTQETITNAESAKAWFLGKASDKAHVAKHFVALSTNKAKVEAFGIDAANMFEFWDWVGGRYSLWSAIGLSIALFVGMANFEKLLAGAHFMDEHFRTAPLDRNAPMILALLGIWYINMFGAESHCLLPYDQYLHRFAAYFQQGDMESNGKYVQRDGTRVAHQTGPIVWGEPGTNGQHAFYQLIHQGTKLIPCDFIAPAKTHNPVQGGLHHKILLANFLAQTEALMKGKSSDEARTELEASGLSGDTLNKILPHKVFQGNKPTNSIMVQQVTPFTLGALIAMYEHKIFVQGVIWDINSYDQWGVELGKQLAKVIQPELDGKDPVSSHDSSTNQLINFIKSFN??????????????????????????????????????????????????????????????????????????????????????????????????????????????????????????????????????????????????????????????????????????????????????????????????????????????????????????????????????????????????????????????????????????????????????????????????????????????????????????????????????????????????????????????????????????????????????????????????????????????????????????????????????????????????????????????????????????????????????????????????????????????????????????????????????---------------------------------------------------YRANPHDMCKFHSEEYVNFIERVTPKNIQTFSKSLTHFNVGDDCPVFDGLYDFCSMYTGASIDGAWRLNNKTCDIAINWSGGLHHAKKFEASGFCYINDIVIAILELLKYHARVLYIDIDVHHGDGVQEAFYLTDRVMTVSLHKYGAYFFPGTGDMYEVGAESGKYYALNVPLKEGIDDASYYQVFKSVISSVIEHYKPGAIVLQCGADSLAGDRLGCFNLSIKGHGECVRFVRNLNIPLLVLGGGGYTLRNVARAWTNETAILVDEQVSAEIPYNEYLEFFAPDFSLYPD-------ENANSKQYLEAIIKYTTENLRCLDHAPSVQMQDIPPDLADLGDDQVKRETTKEHPGEFFD---------------MSDAEEDYELEYSEDDDSQPDVDLENQYYNSKALKEDDPQAALQSFQKVLDLEGGQKGDYGFKALKQMVKINFQLGHYEEMMARYKQLLTYIRTAVTRNYSEKSINSILDYISTSKQMQLLQEFYEVTLDALRDAKNDRLWFKTNTKLGKLYLDREDWQRLSRILRQLHLSCQNDDGSDDLRKGTQLLEIYALEIQMYTGQKNNKELKKLYEASLQIKSAIAHPLIMGLIRECGGKMHLREGEYNSAHTDFFEAFKNYDESGSPRRTTCLKYLVLASMLMQKEINVLDSQEAKPYKDDPDIVALTDLVDAYQAHDIGRFEAIVSPHKESIMKDAFIKEHIEQLLLNIRRQVLIRLIRPYTRITISFISRELKIPSADVESLLVSCILDNTIHGRIDQVRQVLELNP-SPIDRRYAAMEKWSAQVQLIINTVCHKL???????????????????????????????????????????????????????????????????????????????????????????????????????????????????????????????????????????????????????????????????????????????????????????????????????????????????????????????????????????????????????????????????????????????????????????????????????????????????????????????????????????????????????????????????????????????????????????????????????????????????????????????????????????????????????????????????????????????????????????????????????????????????????????????????????????????????????MAASINPSGKPQLLCRLEGHSDAVNQVALIGDADAVISVSEDRTVRVWARRDTGQYWPSVCHTMPSAASALDYEPTSRRLFVAMDNGSITEFELADDLNKITFKRSYIAHQQRVTSVKFSHITGWLLSAGKDKYFQWHCTETGRRLGAFQGSAWCTTVELDSASRHAFIGDYSGHVTMLKLTETNYQPVTTLRGHSGSIQALLWDERRQLLISGSFDQVIIVWDIGGGKGTAYELSGHRARVTGLALFGPSSLLSVSDDASLVVWDIGAQRQETPEWSERDCCERCARPFFWNVRAMLDQKQVGLRQHHCRRCGRALCDRCSANRSTLPRLGFEFPVRICDECHLHISDRDRQSLATFHDLKAPVTAMTLLQPAKIMATVGSDRSIKIWDLSKVL----------------------------------DVSAGLYGKYPLIQSKEKIDREILKVCQCTPKFADQIVWLRGRLHTSRAKGKQCFFVLREQHFTLQCLLSVGETTSKQMVKFVANVTKESLVDVQGKLVKSPSRIESCSQQEVELQVHQFWVVSLSEARLPLQVEDAARPECADDDEEALKIRVNQDTRLDNRVLDLRTPANQAIFRLQAAICHLFRESLNRRGFTEIHTPKIISAASEGGANVFEVTYFKGKAYLAQSPQLYKQMAIAADFDKVYTIGAVFRAEDSNTHRHLTEFVGLDLEMAFKYHYHEVLDTIADMFVDMFKGLRDRFQPEIEAIGRQYPAEPFKFLEPTLRLEYAEGVAMLRAAGVEMADDEDLSTPNEKLLGRLVKAKFDTDFYVLDKFPLAVRPFYTMPDPTDMKWSNSYDIFMRGEEIMSGAQRIHDPEFLTERAKAHAIDLSTIQSYIDSFKYGAPPHAGGGIGLERVAMLYLGLDNIRKTSMFPRDPKRLTPALDEGLPVEQIDPLEFSRVRIMYPKECRTRRVTYRGKLDVTFWWVNGLKQEPIKRTCGEIPIMVRSRKCNLYGLNPKEIVARGEEMEEFGGYFIVNGNEKVIRLLIMQRRNYPIAMARNGWKNRGSMFSEFGVSLRSCRRDGQNMVLHYLINGTVQVMLTNRKEIYFIPVILLLKALTDKSDYEIYKALTKSCEKDTFYKGCISNMLRLVQENILTQDAKEFIGSNFRWASNADVCDKLLDKCVCVHLDNNEDKFNLICFMVRKLFAVAKNKCAIESADSTMNQEVLLPGHIFLGVLKEKMEGILTGVKLSIDKKMSSALDLTNGMNYFLSTGNLVSKTGLGLQQTSGFTIIAEKLNFWRYLAHFRCVHRGSFFMEMRTTTVRKLLPEAWGFLCPVHTPDGGPCGLLLHLTAMCEMVVQLDGKCIGWVLRYFKASGNIPSTLEICVIPRTGTNSLYPGLYLFSTPARMMRPVLNRARCVEWIGTLEQVHMDICVVAEEATTHQELRETAMLSVLANQIPWSDFNQSPRNMYQCQMGKQTMGSPMHTFRNRADNKLYRLYPQSALVRPTAYDFYKMDEYPSGTNAIVAVISYTGYDMEDAIV-----------------------------------------------------------------------------------------------------------------------------------------------------------------------------------------------------------------------------------------------------------------------------------------------------------LRETSRDLHRHQRNEPELHPMATAREYTAAVNAAKLDRIFAKPFLCSLGHRDAVELLAKHPERISGAVSASNDGELRWWDLTHRKCVRALQAHDGPVRGLAGQDQSIKTILSKLAHHYSTTFATAGETVSLWEERNEPLRNLW-GIDTVYVTFSPIESLSSDRSIVLYDIREASALRKVVLEMRSNALSFNPMQAMHFTVANENYNLYTFDMRNLRRALQSHTDHVGAVLSVDYSPTGTEFVSGSYDKTVRIYRSREVYHTKRMQRVTSVLYSLDAKYVLSASDEMNIRLWKAKASEQLGVQNYNDTLLQRFGQHPQVKRIIRHRHVPKSLHQEKQVMLASRKRK???????????????????????????????????????????????????????????????????????????????????????????????????????????????????????????????????????????????????????????????????????????????????????????????????????????????????????????????????????????????????????????????????????????????????????????????????????????????????????????????????????????????????????????????????????????MLVLFESPAGFAVFKVLDEKKVQKTENLFREFEDASGAAKILKLKHFQKFDDMTQALSAATAAIEGKLCKPLKKVLKKLVASDAHETLAIADAKLGSIIKEKMDISCVANSSIQELMRCIRSQQEALITGLSQKEVTAMALGLAHSLSRYKLKFSPDKVDTMIIQAVSLLDDLDKELNNYVMRCKEWYGWHFPEMSKVVTDNMLYVKTVRKMGMRSNAINMDLSDILPEDQEAKIKELAEVSMGTEIAPDDVENIKHLCDEVIQMTDYRATLYEYLKNRMAAVAPNLTVLVGELVGARLIAHAGSLLNLSKQPASTVQILGAEKALFRALKTKHDTPKYGLIYHAQMVGQSSQKCKGKASRWLAAKAALAIRVDALGDDADTEMSIRNRANLEARLKMLEEGKLTRISPFLWPKNNVKLQLAVLVCFATLVLGRVLNPISPIMQKKIVDGLSASTKITI---MVQGSNSWLTNLRSFLWISIQQFASKTTQIGLYSHLHSLSLGWHLSRKTGEVLKILDRGVASVDALLSYFLFQILPAFADILIAFGYFTYAFNWFGLIALSCAVSICITEWRIKFRREMNQLDNAAQAKGVDALLNFETVKYYNAEEIEISKYQKAEWKSSASLSLLNVVQGSILTTIGSLLCAYMTAGDYVLFLMYSAQLYTPLNFLGTYYRVIQRSFIDMENMFELLDVVDHHLKV--EIEFRDVCFRPILKHVSFVVPHGHTVALVGHTGSGKSTVLRLLLRFYDVSSGSILIDGQNIAGVSLRSAIGVVPQDTVLFNTSIRENIRYGRPSDEVELAAQAADMHQSIVQMYDTVVGERGLKLSGGEKQRVAIARTILKAPAIVVLDEATSALDTRTERHVQKALAVVQNRTSVVIAHRLSTVVNADQIIVLEHGEIVEQGHRELLTKKYAAMWFAQLEFNMPETRVTTLANGVRVASEDNGAPTATVGIWIDAGSRYETEKNNGVAHFLEHMAFKGTGKRTQTQLELEVENAGMHLNAYTSREQTVYYAKCLRKDLANAVDIVADITQNPKLGEQEIERERGVILREMEEVEGNLQEVVFDHLHAVAYQGTPLGLTILGPTKNIKSLQRQDLKDYIDTHYTGSRIVLACAGGVDHDELVKLAEQHFGKVGTGFDQQ--SPCRYTGSEVRVRDDDMPFAHVAIAIEGAGWTNPDNIPLMVANTMIGSWDRSHGGGANASSRLAAVAATDRTMHSFQSFNTCYKDTGLWGVYFVANGDELDDCMSAVQNEWMRICTECVEADVTRAKNLLKTNLLLQLDGTTPLCEDIGRQMLCYGRRIPLHELEARIDAVTANTVRDVALKYIYDRCPVVAAVGPVSGLIDYVRIRSQMYKHSVHTLVFRSLKRSHDMFLCDEGALPPVDDRAHRLRVATKARDDYGPVMHLVSEGQRASATGALMLAGSQLTAQLKKPSIPKPQWHPPWKLYRVISGHTGWVRCVAFDPTNEWFCTGSNDRIIKIWDLAS---------------------------------QVKCWDLEQNKVIRHYHGHLSGVYTIGLHPTIDIIVTGGRDSTARVWDMRTKANIHVLGGHTNTVASVLVQATEPQVVSGSHDSTIRLWDIVAGKTRVTLTHHKKSVRALVLHPKLNMFASGAPDNIKQWMCPDGKFIQNLSGHNTIVNCLAMNDDGVLVSGGDNGSLQFWDWKTGYNFQKMTTPVQPGSIDSEAGIFAMGFDLSGTRLVTCEADKTIKVFKEDENASEETHPINWKPEIVTGSTSEYLINCSAEHYLEALGINVKAKNFLVFQGAVESIAMKNPKERTALFEEISRS--------------------------------------------------EELQKKRRKEKIENELKEKKKVEQTHRDDVELNKRKPAYIKAKEKTTHMQKKLDAAKKSLEAATKTHKSHQGIEELEHELAQVEEAFEQEDVSLEESQVKEYNRLKEKAGKMASAALQEYDSVAREQKTDQDHLDNELRKRNECEAKLKELEENQRRVNKLVDLHDLKEEEKKLEAKRRLTEKFEDVASLGDAKVDKHEDTRRKRKSEIVEHFKKLYPGVHDRLVNLCHPIHKKYNVALTKVLGRNMEAIVVDTEKTGRACIQYLKEQMLEAETFLPLDYIDFKPLKERLREFKDVPNVKLLYDVLKYEPLSIKKAVLYATNNALVCETAEDAAKVAFQSPDGKRYDAVALDGTYYQKNGFISGGSSDLAKRAKRWDDKDFHKLKGEHTKLQEELREAMKTARKESDLTTIESQIKGLETRIKYSKVKQLEHSMREREVRINEIKSRQNTVEDDVFHDFCEQIGVANIREYEERELHASQEREQRAELENQKNRIASHLEYER--TKDTLAVEQDQQELARLKDIEQKQKELIEQQMEAISALKNERQSKKMKVDEIDEEVAEIRKRLTAQQKEVTSVQKTVTQAEARLEQKRSERHTLLQSCKLEGIRIPLIRGSMAQMYEREALQIDYSQLRLEKEINLQRIQAPNFKAMEKLDSVKERLKDTDTEFEHARRKAKSNFELVKRERTCFEHVSNCIDEIYKSLTNNPSAQAFLGPENPEEPYLEGINYNCVAPGKRFQPMSNLSGGEKTVAALALLFAIHSYQPAPFFVLDEIDAALDNTNIGKVARFIREKTQTSFQCIVISLKEEFYGHADCLVGICPDPGECTISRIYTIDLSVSEAMLVYLGEAIREGNLVEMQNLYANSLATLMHNYVHTIYKE--LYYRQIYADDRFNSYQNYVNLFNYILSSGPVSLELPNQWLWEIMEDFVYQF--QSFSTFMWNVHSVLNVLHSLVQKSNINQQLEVFSKGGNPDEVAGEYGSRTLYKMLGYFSLIALLRLHSQLGDYFQAIKVLQHLELNRQGLSRVPACQVSTYYYVGFAYMMMRRYEDAIRTFCNVLMYISRTKRSYQQRQMISQTDKMYSLLSICYVLHPQRMDESVMAQLKAKHMLKMTQGNMEVFERCPRFLSPVQWNAFRGEIEAQRTIRSYLKLYTTMPVEKLTKFFLMCFKHLMSNVVLEGEFQTGSDMDFFIDKDMIHIADTKIAPKYGEFFAKQFNKFEEMYE----------------------------AYRDDNGKPFVLPSVRMAEQELMTKNLDKEYLPIGGLGEFCNNAAKLALGDDSPVVKDGRNATVQGISGTGSLRIGAMFLDEFLKGNKSVYMPNPTWGNHIPLFKKCNFQVKQYRYYDPKTCGLDFDGALEDISNIPEGSVILLHACAHNPTGVDPRPEQWVEIEKVIRSRNLFPFLDMAYQGFATGDIDRDAMAVRLFAKSG-PMCLSQSFAKNMGLYGERVGAFSLICESKEEMSRCMSQLKILIRPLYSNPPVNGARIANLILSDAKLRAQWLADVKLMADRIISMRTRLRDGLKREGSTRDWKHITDQIGMFCFTGMTPDQVAKLTKDFSVYLTKDGRISVAGISSNNVDYLAHAIHNATKRIIPKLRHVAHCVLMATLIYLFVKWSRPPDSVDPFAEYGVVFTIILYLFRLLPLLALPQSLTNLLGLTLYNAFPPKVRLKVAAQEAPFLCIRVVTRGDFPALVRHNVDRNLATCLDLGIENFAVEVVTDKAVYLASHPKIRQTIVPPDYRTRTNAMFKARALQYCLEDDVNVLADNDYILHLDEETLLTRDAVRGVLNFISSGQHPFGQGLITYANERIVNWVTTMADTYRVADDLGKLRFQFNCFHKPLFSWKGSYVCTRAGAERAVSFDHGPDGSVAEDCYFSMVAYARGYSFEFIEGALWEKSPFTISDLIQQRKRWLQGIYLVVHSSKIPWRFKVWLACSLYAWATMPLSTSNLFLAPNFPLPCPQTFNVTCAFIGALNLYMYIFGVLKSFSIKRLGFFGFFMCLALVVVAIPVNIFVENVAVVWGLLGKKHKFYPWPNYRYTGKLRAGRVTPKRPVPEHIQRPDYADHPEGVPISEQKMKGA-EIKVLSKAEQDGVRRASLLARECLDVALAAAKPGVTTDELDRLVHEAAIERDCYPSPLNYYMFPKSCCTSVNEVICHGIPDDRPLEDGDILNVDVTVYHNGYHGDLNETVFIGKVDDNAKKLVRVTYEALQKAIDACKPGMLYRDIGNIIQRHVQQNGFSVVRSYCGHGIHSLFHTAPSVPHYAKNKAVGVMKPGHCFTIEPMISEGNWHDTVWPDNWTAVTTDGKRSAQFEQTLLVNE---------------------STLRESLKERSARRRELLAQQLGAGCADNLGLLLGNDKTPTVGQGTQPDDDEVMAYRDSSTFLKGTQSANPHNDYCQHFVDTGQRPQNFIRDVGIQDRFEEYPKLKELIRLKDDLIRETATPPMYLKCDLLQYNLRELNGKFDVILIEPPLEEYQRSCGVTNTRFWSWEEIMKLEIEEVAAPRSFVFLWCGSSDGLDLGRQCLRKWGFRRCEDICWIKTNISNSKAKNVEPRAVFQRTKEHCLMGIKGTVRRSTDGDFIHANVDIDLIISEEPPFGMMEKPEEIFHIIEHFCLGRRRLHLFGRDLTIRPGWLTLGPELTNSNLNTDAYNAHFNTANDYLTGCTERIEALRPKSPPPKGKRVVMRVDFNVPLKDGKITNNQRIVAALPSIKHCLDKGAKSVVLMSHLGRPDGTANPKFTLAPVAGELKKLLEKEVTFLKDCTGSEVEAACADPAPGTVILLENLRFHVEEEGKGVDAAGNKIKADPAKVKEFRASLTKLGDVYVNDAFGTAHRAHSSMVGVELPQRAAGFLMMKELEYFSKALDEPARPFLAILGGAKVKDKIQLIENLLDKVNEMIIGGGMAYTFLKVSSGMKIGGSLFDEDGAAIVEKLLKKAADKKVQIHLPVDFITADKFAEDAATGSADVDGGIPDGWMGLDCGPKSVALFVEAVGRAKTIVWNGPVGVFEFDKFAAGTKAVMDAVVSATGRGVVTIIGGGDTATCAAKWGTEDKVSHVSTGGGASLELLEGKVLPGVAALTDAMRTFGDRPHSFQLEDGGEYYYIGTEVGNYLRLFRGTLYKKYPSLWRRAVTVEERKKISQMNMSQHSAANFISLLKKSEVDDLIDGNEEKYRAAPVQENDGGAHGAKNARPSFMPAAPNNAHHLDAVPCSTPINRNRLQHKKNRSFPMLYDDLDPAMLHESAALPECLVPIRLDMEIEGSKLRDTFTWNRHEAHISPEQFAELLCDDLDLPPLLFVPQIAASMRQQIEAFPSESLLDEQTDQRVLIKLNIHVGNISLVDQFEWDMSERANSPEEFAAKLCSDLGLGGEFVTAIAYSIRGQLAWHQRTYAFSEAPLAQLEMPFRPQSEAEQWCPFLETLTDQEMEKKIRDQDRNTRRMRRLAHTAW--------ERIDALPSSERYLGLVNFGNTCYCNSVLQALYYCKPFREKVLEYKAKNKRTRETLLTCLADLFHNIHSHKKKTGTLAPKKFIARLRKDNEVFDNYLQQDAHEFLNYLLNTIGDLLQAENSWVHDIFQGTLVNETRCLTCETVSSKDEDFLDLSVDISPNTSISHCLRCFSSTETLRGEHKYHCEQCNSKQEAQKSLKVKKLPPILALHLKRFKYTEQQNRNTKLSWRVVFPLELRLFNTSDDALNGDRLYDLVAIVVHCGTGPNRGHYISIVKSHGLWLLFDDDLVDKIDPATIDDFFGLTQDTPKASESGYILFYQSKE??????????????????????????????????????????????????????????????????????????????????????????????????????????????????????????????????????????????????????????????????????????????????????????????????????????????????????????????????????????????????????????????????????????????????????????????????????????????VDGSGPEETQDAEAATIDELKEHTAHIQKAVAQKESRFILRILRLLPATRKKLNSKLLRKTINGFYTHDKAHRELLLSFVDAEDTDADAVQKSAHLALLPEVDVYLHLLLLVHMVDAANMERAVRCAELLKSKIETHSRRSMDLLAAKTFFYYSRVYELDGKLCYCISFLLKRLRTATLRSDFEGQAVLINCLMRNYLHYSLFKQAAKLVSKVSFPEMASNNEWARYLYYLGCIKAIQLYYTDAHKNLLQAIRKAPQHSALGFKQTVYKLAVTVELLLGDIPDRTIFRQPALRKSLAPYFQLTQAVRTGNLGLFNQVLESYGARFQADHTYTLIIRLRHNVIKTGVRMINLSYQRISLADVAAKLQLGSAEDAEFIVAKAIRDGVIEATIDHDKGYVQSAENIDVYCTGEPQAQFDQRISFCLDIHNQSIKAMRFPPKSYNKDLESAEERREREQQDMEYAKEEDDDSYQVAVRLTVALAAFAALTMWKSAVTDMTENFRESHPLLFEFLANLPDQVVRSLTLSSVVLSLCHSALLSPLRQAALRAVPLNFINALL-DISDEPACEAVLSLAQHQDGNQPKLMMTWLVQITKCVPVVQSKQAARDYDSIYSMLTGVADTHASLILKG-LRDTETQMEMLVKMLLDCVGTPGVYPAEETLSRVALPFWHTLLDEHNRLLP-AETAKNLQPVYEQLVRLLNKSQLPDPDCLDSDEREELRCYRQDITD--CYMCIMTVLPVQLIRFFMTELETANNSRIIEACLFAINSVGDVIDGDEDVGIVSAILEILPRVPASDEIQSQVMTAVGTFADENIGPLVHILLGGLQRTSAAASMALKDVARAHGDRLEPAANDILQAIRVLKHRDRQRLVSIVGHVASALSSDQALTSLTALMAPFVLQMNEMVNLAEQLLPLFKQIATKYANSDCEVVSSLAECIRKMVPILEIDVILLELLTICGGLLINTTTSCS----TELVEAFYRMGTLLKKLSPDDLIQLCPEHHTFKSLTQFVIATERSEIKEALEGIVIQLIQNMCVSRQYITCQADIFLALSRSAAALHRFARTADSRQNFVRQILRERSNKRILINVV--SDFTVQVKVKWKDSY-DVHLHLPDVFRAQLFALTGVLPERQKIMCKGSILKDS-WGATILMMGTKEELPQPVEKTLFVEDMDDSEISAALKLPTGLTNLGNTCYMNAVVQCFKTVPELTELDKFVVSITAALGSLYRSMDKATRAPLVLLQALQTAFPRFAEKGEHGQQDANECWTEMMRMLQSLVDQIFGGKLVSLKCTESEEESTEDFLQLSCFID-KDVKYLMAGLKLRM-RETITKMSPVLKRDAAYKTSKISRLPAYLTVNLVRFFYKERESINAKILKDVKFPMMLDVYELCSNDLQEKLLPQREKFKQWDDEFSFEDDGSNNSGYYQLQAVLTHKGRSSSSGHYVGWIRREWFKCDDQVVVPEEEILKLSGGGDWHVAYVLLYGPRLLVGLINKRLPKELLLKIFSFLDVVSLCRCAQVSKEWNVLAMDGSNWQSIDLFSFQRDVTSEVVAYIARRCGGFLRRISLRGCQGVPDDSLYVFAQHCRNIEQVLSNCHKLTDDSVLALALSLHIDSCVELTDLSLGSF----KHLRVVNISWCRKITGQGIGMIAG--EHLLRFTAKGCLDNEAIVKLATKLQVLNLQCCSFLADSAVIAVAQNCPDLRHLCVSGCTQLTDAAPQALATGCLHTLEMANCTRCGDAGLIPLVKACHDLRRLDLEECALVTDSSLNHVAAFCPLMEQLTLSHCDQITDQGVHKLALQCIEIDNCPFISDTSLEYLADHLRRVELYDCQLITQDAIGKFQPDVRLHTYFAPATPRQRYCRCCVIVSSKGARVFCPDHPNANLIEDYRAGDMICPQCGLVVGDRIVDVGTEWRVFQNEKSSNDPTRVGAAENPLLSGSDLSTIIGRTGDASDDQGNAKYANRKTMSASDRALIGAFREISAMGDRINLPKTIMDRSNLLFKQVHDGRSLKGRANDAIASACLYIACRQEGVPRTFKEICAVSKVSKKEIGRCFKLILKALETSVELITTGDFMSRFCSNLALPSAVQKAATHIARKAVEMDIVAGRSPISVAAAAIYMASQASPDKKSQKEIGDIAGVAEVTIRQSYKQMYPKAAQLFPEDFKP????????????????????????????????????????????????????????????????????????????????????????????????????????????????????????????????????????????????????????????????????????????????????????????????????????????????????????????????????????????????????????????????????????????????????????????????????????????????????????????????YVKKEHSLVKPYQGSGMTMPNWDFTGSTMISSNYVRLTSDTQSQQGSIWNKVPWEIHLQFKVHGHGKDLFGDGFAIWYTKDPLQPGPVFGSRDFQGLAVFLDTYANQNGHHNHAHPYISAMVNNGTLSYDHDRDGTHTELAGCEAKFRNSDYDTLISVRYEHDTLVVSTDIMGQKEWKECFRVSGVRLPTKYHFGVSAATGDLSDNHDIISIKVFELDELREFIAPQAAPHRDHVDDSMSGTKFFFLMLFLMLVLVCFFYQRRQEDARKRFYRDRDAFDEIRDKNCRIQDINELFLPNDTVTHVPNIKRLNIDPVFPNRTNLLHIHNMAISKAFFFSFILQRAKDDQPGFMYYFMSVISDVAANRFINASAIYYAPNMSFTPSYKGFFNKTMPLFAPRAFRSDDFNDPYHLEGTSTLNTIEAIDLGAISQNYSSDQYRINEWYSSWLPDLTKRQDSKTTYTVQITGNNDTFVWHGPPAANDNPGPVKWVRPYFDCGRSDKWVYGATSPIPDIYPRHTQWRHIEIPRYVAVAAMELDFERIDVNQCPFGPGN-PRPNYFAGTSRCKNDTTDCEPVHGYGFRRGGYQCRCKPGFRRPRMVRNPYHGELIERASKQEYEKGYQCDKIGYIGVLTQNLNNYMAIGTRMDPALGGNVLYGKEVQLENEARMAVRLANFVSGFLQIVDPKDLFAEFRVPDKPLTADQMIGEVMSIVIGDQKVVGAGVYFDYKAFFGPY--AWRQGRNERKYFVDDTTKIQIRYNSSGIKYDHYPLQYKAADVGYWTSPYFDCGGYHNSWMVTYAVPFFGWDSLRARLQFRGVVAVIELEQLEINQCNAFENTHKCDRKSSRCVPILGRGFQGGYKCECNQGYEYPYNDPITYFDGQIVEAPSRFERMTHVSGTDKSKYGGRYMVTALPGDGIGPELIGYVKEVFRYGGVPVDFEEVHLDSSRDDIDLMEQAITAVKRNGVALKGNIETRHNDPNCKSRNVELRLRLGLFANIVHVTSQPGIETRHKDVDIVLIRQNTEGEYSSEEHTSIKGVVESLKVITKARSDEIARYAFEWAKNNGRKKISCVHKANIMKLSDGLFLQCCTETAKEYPEIEFDNIIIDNCSMQLVANPHQFDVLLLPNLYGNILTNLVCGITGGPGIASGRNYGREYAVFETGTRNTGKSIAGKNIANPIAMMNAGVDLLYHLGLTEHAEIISTAIDKTLNVDRVHTPDLGGQATTTEVVQNIVKEVQKHSMTYSASSNEVLLRDPPTDGISAVKFGQTSNQFLVASSWDGFVRLYDIHGDRVRAKFEHGSPVLDTCFQGSSHVWSAGSDGGVRLFDVNQGTELKAGSHDDTVRCIEYASDVSLVVSGGWDGAVKLWDARRPVGASSHTQDNRVYAVAIAGERIIVGTANRKVLIWDLRNMAFVLQKRDSSLKFQTRAIKAFPDKTGYVLSSIEGRVAVEYLDPSPEAQKKKYAFKCHRMKDVTKVEHIYPVNAIAFHMIHGTFATGGSDGFVNVWDGRNKKRLCQFHKFPTSIASLAFSPDGSALAIASSFMHEYRLEPNQPPDQIFIRHV--------------------MKEYKPTDATTNPSLILQAAKLPQYADLIDKAVSYGRSPLEQLEEARDKLFVLFGSEILKIVPGRVSTEIDARLSFDKDASIAKALKLIALYKELGVPKERILVKLASTWEGIEAARVLEQEHGIHCNMTLLFNFTQAVACAEAGATLISPFVGRILDWYVANTDKKSFEPQDEPGVKSVTSIYNYYKKFGYKTVVMGASFRNVGEIRALAGCDLLTISPSLLKELANSDEPVVQHLQAEKAASLPLEKIEVDEKRFRWDMNEDQMATEKLSDGIRKFSADARKLEVLIQEKLKMGDSVCRPLATVYTDTNEASGTNAGMPAVFRSPIRPDLISFVHHQLLKNKRTPYAVSKEAGHQTSAESWGTGRAVARIPRVRGGGTHRSGQGAFGNMCRGGRMFAPTKTWRRWHRRVNVTQRRHAAASAISASGVTALVMAKGHAIEQVSEVPFVVSDKVQDYQKTKQAVALLKSVNAWPDIEKVYKSKRLRPGKGKRRNRRYKKKCGPLVIYEKDNGIVRAFRNIPGVDTCDVNALSLFKLAPGGHAGRFIIWTESAFRKLTEIFGTFTKNSKVKKGYKLPRPMMTITDLGRLLKSEEIRSSLRPRKSIILKKTNKPNPLKKVHLMDRLNPYAIVEKRHRILTLEKEAGKNLKAVRAKERMKAAKQKRLANPFPKNRKLVQGAVLKKTLDAIKDLINEGTWDCSASGISLQAMDNSHVSLVALNLRADGFDKFRCDRNISMGMNLASMAKILKCAENNDVITIKAQDDADTVTFVFEAQNQEKVSDFEMKLMNLDSEHLGIPDTDYSVVVKMPSSEFQRICRDLSQIGDSVQLTCTKDGIRFSASGDLGTGNIQLSQTADVEKEEEAVIIEMQEAVTLTFALKYLNSFTKATPLSPQVSLSMSADVPLVVDYLRLFLRILFVPK-----RSNVKVCVLGASGGIGQPLSLLLKQHPGISYLSLYDIAHTPGVAADLSHINTGAKVKGFVGAEQLKAALEGVEVVVIPAGVPRKPGMSRDDLFTTNASVVRDLATACAEVCPKAMFAIISNPVNSTVPIASEVFKKHGVYDPTRIFGVTTLDVVRANTFIAEAKGLDPVSMSVPVVGGHAGITIIPLVSQASPKVDFPQDQLEQLTKRIQDAGTEVVQAKAGAGSATLSMAFAGARFVFSLVSAINGKNVVECAFVKSDIGDAGFFSTPLVLGKNGMEKNLGLGKLSPFEEKMVADAMPELKKSVQKGVSH---EYEWLLKFEVNDIVEQLVIAECSKRFPL-ARSDKFVM-QSKVVATLMGDSISHADITLRL-PKHSQRTIVQNDAQWKLQQIQDAGNHLMQAMNLLRFKFSSGQEVRNLMAEVMGCLGRGRACLVVPKKRTIEEIMQSRNMRSLQPPLPNDVAVSFYIQSYKLVFAVYHV-QKDSQKF-DAECSVPWLSEALVLFTVALQLCQQLKDKVEVFQYNDFLPMFIPIFLEHDAPLPEISELIRTCDVCFKDAESIL--NGFVSILVCEFCRRLESASTPKVAFN--VLYNFFEGLRVDVYCSLIKVAGRSVTEVFADVGPIKTWLHVSKVRDVYRNLHRELSDMALRVMVELLSTYSEHDAAEAAEDAERCIASTLADPNAFLMDHLLPLKPIKALEGKPIHDLLKIFIFDKLSAYQDFYKKNKAFV-EGLGLDHESNVEKMRLLTFMLMAEKQREISFDDIARELEV--TDVEEFTIRALKTKLVSAKLNQMTQKVVVISTMHRTFGMSEWTQLRDILRLEKVEQSTQQ-------------NVEELAEEAKAIDEGEERAVIEESPQFKELVNILIEWINDELAKHRII--VKNIEEDLYDGQILHKLLEQLTNSRIDVVEMTQNEEGQREKLKVVLERASQALGLKWSVDAIHSKNVVAIVHLLVALARHFRAPVKARLPENVTVSVVSVTKR-EQLTRTYDEYGMKVE-RDAFDQLFDHAPDKLVVVKKSLLTFVNKHLNKIKFEDLD-KQFHDGIYLALLMGLLEGYFVPLYLMLTP----------------------KNKVNNVAFAFGLMKDAGLKPKARPEDIVNYDMKSTLRVLYNIFMK??????????????????????????????????????????????????????????????????????????????????????????????????????????????????????????????????????????????????????????????????????????????????????????????????????????????????????????????????????????????????????????????????????????????????ILYRNRAEWADVTPILRISYSEKFQDCFGYLRAVISGELSERVFELTTTCAGENPACYTVWVLRRTLLQHLNKDYRDEMRFMSRMILDNQKNYQVWYHRQRLVKWLELAFLDEILDSKNYHAWQYRQWLLTTFNLWDDELDFTTALLIDDLRNNSAWNQRYFVISKTTGFVVEREIRFTQEKILVNESAWNYL----------------------------------???????????????????????????????????????????????????????????????????????????????????????????????????????????????????????????????????????????????????????????????????????????????????????????????????????????????????????????????????????VSPGTTITTDGYMRGHGGLLSAVAGVVEKVNKLITVRPLKTRYNPEVGDVVVGRIVQVC--QKLWKVDVGGRLFAALHLSSVNLPGGELRRKSIEDERMMSQYLIDGDLVSAEVQNVGVDGGVSLHTRNLKYGKLGQGTLVKVSPSLIKRCKHFHNLP-NGVHLIIGLNGFIWVTSSKFTREAICRTRNVILCLAKYNLMLFDTPIVYAYEIS-LVQLTRHRSDKLVGGWLLACGGMAFGAVILGGVTRLTKSGLSMVDWHPLNEGRPRTAEWEAEFAKYQQYPEYKARNKDMTLEQFKSIYWMEYIHRMWGRSIGAVFYVPATIYWFLGYFSPAVKKRVVVLGGLLAAQGLMGWYMVRSGLQEKPRVSNLRLAAHLGTAFVFYSLLFRSALQHLLPDAAVVRFVRAKALVFITALSGALVAGIEAGLVYNSFPKMADRWIPSDILALPTWKNFFENPTTVQFDHRLFGETVITALYIHSRKVPLPRRARLATHAMLAAWIQVGLGISTLLTFVPTPLAAAHQAGALTLLTTLLWLTHELKLVKK-IAKWGEGDPRWIVEERPDATNVNNWHWTEKNACQWSRDKLMALFTDLEITD-TVMCVIKQVKKCEGEATANNRKAKLIFFYEWELELCAGS-LVKGRVEIPNLSDENNIDEVAVNIVLVDEKLKAMMRSKGTEQIRAKLEEYVSSLKSDFSQGLILPTKDSGANSTSSKGKEFSTSELSMEEFKCTADELYRALTMVQAFTQGPVGGSFQILDTNVNGKFTKLNELEFEWRFKSWPAEHYSKVITIEQANDCSLLQQGVPTDEVDRTRGGWQRYYFDAIKRTFGFGAILMDPETFLEIANQVSKLKMYPYFEIAHCVVTLLYLREDLGTGSYA-SR--------VSMFSIFAGAMFAALLLGEPVLAAFKSNQSLVLATGVWYLIFYSPFDIVYKLSKVLPIKLVLTLAKEVTRAKKVHDGVHHAAKLYPNGYLIMVIIGVVKGNGSSFLKVFERLLRGLWTPLAMEIMQPSFATKACVVASLVFVIDKKTDLISAPHSLVYFGVVCFFLYFKLSSVVLGLHDPFTPFENLVCAVFFGGIWDAISRALKDAASKKKEMFRPDGIVQRRTADGGDNDKETRMTLMEEVLLLGLKEKEGYTSFWNDCISSGLRGCILVELGIRGRIDLEAAGMRRKSLLMRKVVVKNDAPVGDVILDEALKHIKETTTPETLQNWVDYLSGETWNPLKLRYQLRNVRERLAKGLVEKGILTTEKQNFLLFDMTTHPLEDQNVKDKLIKRVQDSVLSKWVNDVHRMERRQLALLLLSHASDVLENAFNPLSDEEYELAMRRVRDLLDMDFEAECAKSQSCDIMWGVFAAFVKMADGVPTFKCVLVGDGGTGKTTFVKRHVTGEFEKKYVATLGVEVHPILFHTNRGAIRFNVWDTAGQEKFGGLRDGYYIQAHCAIMMFDVTSRITYKNVPNWHRDLVRVCENIPIVLVGNKVDVKDRKVKAKAIVFHRKKNLQYYDISAKSNYNFEKPFLWLARKLIGDANLEFVAMPALAPPEVTMDPEWQSKLENDMKEAQNTSLPDDEDDDLLAAQMTPVLKESKFRESGMLTPEEFVAAGDHLVATCPTWAWARG-DKTYLPEDKQFLVTKNVPCHKRCRDMEERVIEED-EGWVDTHDNEDDDEAGVDMDAFLDEDAEAVDILATRTYDLNITYDNYYRTPRLWLTGYDENCRPLTTEELYEDISQDFAKKTVTVENHPHIEGLPQASVHPCRHAQAMKNLIQTVEDGGGLEVHMYLIVFLKFVQAVIPTIEYDYTANFNMMSNLEKKLPYDKLCANVDVVKKRLNRPLTLSEKILYSHLDQPQSEEIVRGQSYLRLRPDRVAMQDATAQMAMLQFISSGLPKVAVPSTIHCDHLIEAQLGGVKDLARAVDINKEVYSFLASAGAKYGVGFWKPGSGIIHQIILENYAFPGLLMIGTDSHTPNGGGLGGLCIGVGGADAVDVMAGIPWELKCPNVIGVHLTGKMSGWTSSKDVITKLAGILTVKGGTGAIVEYFGPGVQSISCTGMGTICNMGAEIGATTSVFPFNSRMADYLASTNRRDIADAAESVKDLLTSDSGCKYDQVIEINLDTLEPHVNGPFTPDAAHPISQLGKVAKEKGWPMDVKVGLIGSCTNSSYEDMSRSAMLAQQALDHGLKSKSLFTVTPGSEQIRATIERDGQAKVLKEFGGMVLANACGPCIGQWDRQDTKKGEKNTIVTSYNRNFTSRNDANPQTHAFVTSPEMVTALSIAGRLDFNPLTDELTGSNGQKFKLKPPVGDELPRAGFDPGENTYQGPPPDGSSLTVDVDPKSQRLQLLSPFDKWSGSDLKDMVVLLKAKGKCTTDHISAAGPWLKFRGHLDNISNNMFIGAIPEESGEANKVQNRLTGAWGAVPDTARHYKAQNVPWVVIGDENYGEGSSREHAALEPRHLGGRAIIVKSFARIHETNLKKQGLLPLTFADPADYDKIKSDDRISIVGLDSFAPGMESRAGMFKNTFQSGFLSILYSLGSKPLQIWDKKVRNGHIKRITDNDIQSLVLEIAGSNVSTAFITCPADPRETLGIKLPYIILIVKNLKKYFTFEVQILDDKNIKRRFRASNFQSTTRVKPFICTMPMRLDEGWNQIQFNLADFTRRAYGTNYVETLRVQIHANCRLRRVYFADRLYAEDELPAEFKLYLPVQ??????????????????????????????????????????????????????????????????????????????????????????????????????????????????????????????????????????????????????????????????????????????????????????????????????????????????????????????????????????????????????????????????????????????????????????????????MGRKFYVGGNWKMNGVKASIQKICEQLKNAQPCTEVCVGVPAPYLQLVRDELPANFHVAAQNCYKAASGAFTGELSVDMIKDCGCDTVILGHSERRNVFGEKDQLIAEKCAFALNNGLTVIACIGELLEEREANKTEEVVYRQIKAYADLIKEWKNVVIAYEPVWAIGTGKTATPDQAQEVHAKLRQWLTNNVSEEVGLNTRILYGGSVTAANCKELAQKADVDGFLVGGASLKPEFVQIINAKQMSTAVSKKRKFVADGVFNAELNEFLRRELAENGYSGVEVRTGTMKTDIIIMATRTQDVLGERGRKIRELTAVVQKRFGFKEGSVNLFAEKVSSRGLCAITQCESLRYKLIGGLAVRRACYSVLRCIMEAEAMGCEVVVSGKLRGQRAKSMKFVEGLMIHSGDPTNHYVETAVRHVLLKQGVLGIKVKIMHPYDPLGKRGPALPLPDKVTVTASKHEDDNVEIRSE-KDNTG???????????????????????????????????????????????????????????????????????????????????????????????????????????????????????????????????????????????????????????????????????????????????????????????????????????????????????????-MVDLVLDRDIRIWVFLPIVVITFLVGIVRHYVSILLTSTRKAEIQQVYDSQALIRCRYLRENGKYLPAKGFMMRKHFFNDEDTGWLK-TQKRASPMSNPMHDPGMMTDMLKGNLTNVLPMIMIGGWINWTFSGFLTTKVPFPLTLRFKPMLQRGIELVSLDASWVSSASWYFLNVFGLRSIYALVLGEENAADSTRAMQDQMTGPAMQMPQDPKAAFKAEWEALEVVEHKWVLAGVEEMVIYKGAASEAGRAMQILKRRERQKEDVELKRQRIEHEMRVS-MGDKFSSHFDAVEAQIKSATVGLVTLDEMKAKQENAVKEREKRLAQKEQEEKQRAEDKKWAQKEKQKKAIQALSFNLDEIRVKKNPDVDTSFLPDREREEQERLIREELRQEWKDKQKTLKEESIQITFSYWDGSGHRRVVDMKKGNSIYQFLQRCLDSLRKDFYELRVVSADQLMYIKEDLIIPHHYTFYDFIVTKARGKSGPLFAFDANEDIRMTSDASKEKEESHAGKVLLRSWYERNKHIFPASRWEPYDPTKCYDKYTIKDKKKNKPTVPETLLKERKFNAELRQQRLLAAAGKKKAARARRVLAFRRAEQYVSEYRRMAESEKSNRLVAKVNGNFFVPDEPKVAIVVRIRGITGVSPKPKKVMQLFRLRQINNAMFVRLNKATINMLRIAEPYLAWGYPNLKTVRDLIYKRGFGRVNGRRVPLTDNVIIEEKLGKYGIICMEDLVHEIYTVGPNFKQAVNFLWHFKLNNPKGGWRKKTTHFVEGGDYGNRETLINSLLRKMVVKISADDLWRGVTSVSNAGRKRGRASGSSRKFAKNLNKGQTIGFGKANMVWPGLNVPVIRGREVVQQQALPPDPERQQRLLAIRDKQHIFQRAKRGPLERGWSGAKAPGRSLGPPDPIADQKFDDFKSVIIQLRTISIMRGYRGRIRRHKAVVIVGNGAGLCGLGIAKAPEVRVALRKAKNRAIRRQCYFQRYEDTVLHDFISEVAFTRISVKKKQRGFGLVCHRAIREVCKCVGIKDLYAKVDSRCTRPVPTIRAFLLGLHRQRTHQEIADEKRLHVVEYRPENNYFPRIVASPQHVRTEEEVDPYEYLDMDLITSKGRLLVQKPKYEPFYRRLPCWQIHLKKTDNLKNDRIIRQRLILKYGSLKSFLTVREAKALAGG????????????????????????????????????????????????????????????????????????????????????????????????????????????????????????????????????????????????????????????????????????????????????????????????????????????????????????????????????????????????MAVGKNKGLSKGGKKGLKKKIVDPFSRKDWYDVKAPSMFNVRNVGKTLVNRTVGTKIASEGLKGRVYEVSQADLHSGEDAFRKFKLVCEEVQGRHCLTNFHGMDLTTDKLRSMVKKWQTLIEAQVDVRTTDGFVLRLFCIGFTKKAQNQVKKTCYAQHAQVRAIRRRMVETMQREVASCDLKEVVNKLIPESIGKDIEKSCNYIYPLHDVHIRKVKVLKKPKFDMGKLLEMHDEGKGPSGGDGMAVDRPDNFEPPVLDEVVKIGIIGGSGLE-RPNLLKEVTPYGKPSDA-LITGKIDGVDVVILSRHGRGHTINPSNVNYRANLFALK-QEGCTHIIATTACGSLKEEVHPGALTPRSFIDRTREQTFYVCHIPMVKPFLMELISKHPSGVIVCIEGPRFSSRAESVLFRQWGADLVNMTLVPEVVLAQELGVPYAALAIVTDYDCWR-D--DAVDVSKVATLKDAADGVCRLLRAVLPKINKIAIFMRVLRHEEFETGCKAACNGRYDGFWSKTMIGYGPEDTHFVMELTFNYGVDTYRKGNEFRGIVIEDCGIIKRCEELKYATEKDRNRTVLNGPGGHKFYIVDKTS-K-EDPVKQVIYSCTDVAKTSKFWVNTLGCRLLNTGDDFLEVAYDEAKTSLRFEKISEAMDRGEAYGRIAFACPKSHLPELESQVKNGGGSVITPLVSLSTPGKASVEVVILGDPDGHEICFVGDEAFRELSKEDPTSLKVLQEHMA--EYEAFLKKTSARPHQD????????????????????????????????????????????????????????????????????????????????????????????????????????????????????????????????????????????????????????????????????????????????????????????????????????????????????????????????????????????????????????????????????????????????????QMVPIVIEQTGRGERAYDIYSRLLKERIICLMGPINDDVASVVVAQLLFLQAEAPKQPIHMYINSPGGNVTAGLGIYDTMQYVTPPIATWCVGQACSAASLLLAAGEAGMRHSLPNSRIMVHQPSGGISGQATDIQIHAEEILFLKKRVNLIYAKHTKQPIETIDAMMERDRFMSPEQAQELGLIDRVMERPPMRLDRPVGTWLTLLPLWALAAAAPAGSLPDLGIFCTGALLMRSFGCTINDMWDRDIDRQVQRTRQRPLAAGDISRWDALWFAGGQGLACLVLLQLNWHTVMLGLASVGFVVIYPLMKRFTYWPQAMLALVFNWGALLGFSATAIPMYAAAFSWTLIYDTIYAHQDKKDDILVGMKSTALRFGERTPLWLGAFSTMATALMAWPYFAAVTLKIDNIGLLVGCIGGTLFKIARVDKPKTRKGSRILKSKEPLLIENAKTSFVKGANINQPTVQILKDIYTLKKTESVFYQKKHPFEDATPLERLAKKDTSLFAFGSHNKKRPQNIVLGRCFDATILDQFEFGVQNYKALHEFKVAKIGIMVKPVLVFAGEAWQEMKRLKNFLTDFFRGDYIGLSGIEHVISFTASDVLLRSYRIQLKKSGLKTPR-VEVEEIGPRMDLKVRRCKIASDDLFKQALRRPKELKAKKKKNLEQDDLGTALGRVHMERQDFRLQTRKMKGLKVEILPALQDNYMYLVITREAAVVDPVNPQKVFEAVKVKLLTVLTTHHHSDHAGGNDQIVVYGGDDRIKQLTHKDDFTIRIGQMVRTILTPCHTSGHVCYYIVFTGDTLFTAGCGKFFEGTAKQMLAAMLGALPFETRVYNGHEYTVNNLKFALHVEPNNDAKRKLSWAKDEPTIPSTIAEEKSFNPFMRTAVQRHTDPIAAMDSLRREKDTFRMNRLFGKAKAKEPPPNLSDCIQTVDQRANNMDEKIKKLEIELVKYKEQMAKMREGPAKNLVKQKAMRILKQKKMYESQRDNLMQQSFNMEQTNFATQQLKDTKVTVEAMKIGVKEMKQEYKKVNLNEIEDIQDDLEDMLDQANEVQETLGRSYGMPEIDDEELESELAMLNDEIALEDTSYLD----MQAARAKATLPDLPYDYNALEPVISAEILKVHHDKHHLAYVNNFNMLKEKFEEAVQKGDLTAQFSLAGAYRFNYGGHLNHSIYWQVLCSAKSGEPSADLLAAINRDFGSLDVMKEKVSAAAIGVQGSGWSWLCYNKATKKLQCVTSANQDPLEATTGLVPIFCIDVWEHAYYLQYKN--------------------------?????????????????????????????????????????????????????????????????????????????????????????????????????????????????????????????????????????????????????????????????????????????????????????????????????????????????????????????????????????????????????????????????????????????????????????????????????????????????????????????????????????????????????????????????????????????????????????????????????????????????????????????????????????????????????????????????????????????????????????????????????????????????????????????????????????????????????????????????????????????????????????????????????????????????????????????????????????????????????????????????????????????????????AYFIVVDAHSEECFHDRVAKGTKMGLTFEVAEGGFLDIDVKITGPDDKVVYNGERESSNKYTFAAYMDGQYKYCFSNSMSTLTPKTVMFSMDIGEEPSDDAKAGADAHENKLEDMIRELHTAMTGVKHEQEYMMIRDRIHRSISESTNSRVVIWAFFENLVIIAMTLGQVYYLKRIFEVRRVVMRLTQILKLPKDYANLPESYVNRVSTKIEWKTPKGRQYRKAVLKKPYGMDRPWTQEWQHNKVGTYPQPKILFRGDLVEILVGDDKGKQGTINYIVPERNWVTVEGLNTVTYPSLQRTLVLLVDPADERPTRIEWRYTEEGKRVRVSLRTGRIIPIPAAEATIDYKYRDKPKDTSAAELAQITFIP-KLATFEMDIMEQHGIEDDRVPARTFWYVEQKLEFILLLSRLASVVFAVLCMLGNPYSFYQKAIICNGFTSALRLHHRMPTVRLNAEFLGAVLLEDACHYLIYSLIF-LPIS-MVLLPPTLFATLHSLQLLDRAGTLHQ---NRVKVFRLIAMTEIFLMPTVVIGLFWGVSLMAPFMYYRFLKLRYASVRNPYTRNVFYELRLMAQQLAAPSCPAVVSRIQFISSRADLAIVSIAVYLSFPEIRPEERPYVKLPTSLEDAKNLGNVLSNYTDDQVLLAFFCTYIFLQSFAIPGSIFLSFLSGFLFPFLLAIFLVCLCSALGASLCYLISYSVGTRLVMHCMPNILYYLIFLRITPLLPNWFINIASPIVGVHLLTFFIGTFIGVAPPSILAIRAGISLQQLATAFTWENMLLLLGFAVLSLVPVLFKFKKKFEEVQEKLTRIAIITEDKCKPKRCRQECKKSCPVVRMGKLCIEVTPNDKIAFISENLCIGCGICVKKCPFEAITIINLPSNLERETTHRYGPNSFKLHRLPTPRPGEVLGLVGTNGIGKSTALKILAGKLKPNLGRYNEPPDWTDILAHFRGSELQNYFTRLLEDELKAVIKPQYVDQIPKAIKGTVKELLAKKNDLGKQDHLCDLLELVNVKDRQIADLSGGELQRFATAMVCIQRGDIFMFDEPSSYLDVKQRLKAAVAIRGQLEATKYVIVVEHDLSVLDYLSDFICCLYGTPGCYGVVTMPFSVAEGINIFLDGYVPTENLRFRESALKFKVSDNTDEEVKRLARYEYPSMSKTMGDFELVVEGGSFTDSEIIVMLGENGTGKTTLIRLLAGRLKPDGDEEVPTLNISYKPQKISPKSTGSVRSLLHEKIRDAYQHPQFVADVMKPLLIDNIIDQEVQNLSGGELQRVALALCLGKPADVYLIDEPSAYLDSEQRLVAAKVIKRFILHAKKTGFVVEHDFIMATYLADRVIVFEGRPSVSSRATVPQSLLVGMNRFLELLNITFRRARNNFRPRINKLNSVKDSEQKRNGTYFFLEDEEDGEDLFGPELDRYDVAEEEMRRRDR--VPVPGESIENLEDMKGHTVKEWVTQGPKTEIFNRFKNFLRTYKEKIRAMEQNRMSLEVDYTLAQSEQVLAFFLPEAPAEVLPIFDEAAKDIVVGMFPHYSRIHPEIRVRITDLPILEEIRTLRKIHIDQLIRTSGVVTSTTGVLPQLRMVKYDCVKCKYILGPFVQSQEVKPSSCPECQSTGPFAINVSQTIFQDYQRITIQESPGKVNAGRLPRSKDAILLNDLCDSCRPGDEIELTGIYSNKFEGSLNKANGFPVFATVIIANHILRKDTDDDVKEVVKLSKEELAERIMASIGPSIYGHDDIKRAIALSLFGGVSKNPGQKHRIRGDINVLLCGDPGTAKSQFLKYVQQIAPRAVYTTGQGATAVGLTAYVSPVTRDWTLEAGALVLADKGVCLIDEFDKMNDSDRTSIHEAMEQQTISIAKAGIVTSLRARCTIIAAANPIGGRYDTFHQNVNLSDPILSRFDVLCVVRDERLARFVVDSHARHHPINQDLLQKYILYAREKIEPKLDQDKIAQLYSDLRRESMSTGSMPITIRHLESIIRLAESHARMHLREHVDVNMAIRVMLDSFVTQKFSVMRMGLIDDIQQSFGSENLYDVFGVPKTADADSIKKAYRRKSLLCHPDKAGQEDRDEFTRKFQLLSKCYEILRDPEKRKIYDETGEV--DDSLNSTSDWQGYWRRMFPKVTVTQITDFMNRYIGSDTEREDLKHIYEKCKGDMNKISEYHIGYEEDRL????????????????????????????????????????????????????????????????????????????????????????????????????????????????????????????????????????????????????????????????????????????????????????????????????????????MEMEVYVEAKHTALKHVITMLQMPDQLDKVEQHRKRVQRKKASVEAMLKTAVQSQLEGVRTGLSLLATCLDDASDIRKEEADAIYELAKLLQLQDVREESIRHSQTGTLMEHLKHIFNVPGSVTRTQDLIQDGKLLLAHKLSDLECSRDELLYELHKQANNQASDRAMLKQYFGDVERLSDDLAKQLWLILKRTLNTVRKEPQVIVTALRLIMREEWAALKRQEST-GFLPPGRPKLWRKKAMETLEQSVAERLEANQIEGRSENKMWLVRHLEVTRQLIIDDLKTVKHHCTPCFPPSFDIFNEVRMTHNCLSQRLQTIIAGLVDSEYIHVLSWLNTYNSRELMSHPDIHVDVALLPDTIEKLMERYLAGLHTKFDEWLRNALNDHKDWPEQDTDGYYRTEAPMLIYQMITQHIEVARTVKLVSRVLKLAMDHMGKFLKDYTDYVTEYNFEDRSYTAYMIAIANNSVYMK-LSDLKRNALSYLCEEVLTDIKLMEDIMTETVTVTLADYGEDYIEKQVASSYVKAIC--EKRMSFKNYEERKSAAEFEKLRNSELEVLKLMAEVLKMKDSSLLSLEMNG-------------------------------------------MMKLIINSLYRNKEVFLRELISNASDALDKIRLLSLTNPDALKALQDLKIRIMADKENNVLHITDTGIGMTKEDLMKNLGTIAKSGTAEFLQKVSDGSGGTDLNDLIGQFGVGFYSAFLVADRVAVASKHNDDPVQHVWESNASEFSVTEDPRGDTLKRGTTVSLYMKDEAKDFLEHDTLKKLIEKYSQFINFNIYLWSSKT-VTEEIAEAE--TTE-----ADDDDAKVEDEKEP--PKTKKVEKTVWDWELVNSAKPIWTRKEKDVSDEEYNEFYKTVTRDSQNPLARTHFTAEGELTFKSLLFVPQRQPQDSFNRYGQKTDNIKLYVRRVFITDDFQDMLPNYLSFLRGVVDSDDLPLNVSRENLQQHKLLKVIKKKLVRKALEMFRKISDEDYEKFWKEYSTNIKLGVIEDSANRSRLAKLLRFPSSNDSTGKLVSLSEYVERMKEKQTAIYYVAGGSLDEVQKSPFVERLLKRGYEVLYLTEPVDEYAISSLTEFEGKKFQNVAKEGLSLDD---NKDVREALEKEFEPLTKWLTETGLKDKISKAVVSERLVETPMALVASQFGWTGNMERIVSAQTHMKENDPQRQFYLGQKKTLEINPRHPLIKDLLRRVDDSPSDETAKYLTEMMFDTATLRSGFQLGDHAQFASNVEKMLRKMMGVSEDAQIDAEPGRRGEVKELSAEEQKMAVISEIIQELVSAHHERRDVNLNRVKGDASSRHGLKSQPKLVDIIAAIPPQFKQLLLPKLKAKPVRTASGIAVVAVMCKPHRCPHIAYTGNICVYCPGGPDSDFEYSTQSYTGYEPTSMRAIRARYDPFLQTRHRVEQLKQLGHDVDKIEFIVMGGTFMSLPEDYRDFFIRNLHDALSGYTSANVDEAVKYSERSKTKCIGITIETRPDYCLQRHLSDMLRYGCTRLEIGVQSVYEDVARDTNRGHTVKAVCETFHMAKDAGFKVVTHMMPDLPNVDFERDVLQFVELFKNPDFRMDGLKIYPTLVIRGTGLYELWKTGRYKSYPPALLVDLIAKILSLVPPWVRIYRVQRDIPMPLVSSGVENGNLRELALARMKDLGLVCRDVRTREVGIQEIHNKVLPYQIELIRRDYVANGGWETFLAYEDPQQDILVGLLRLRKCTEQTFRPELVGQCSIVREHVYGSVVPVHSRDPSKFQHQGFGTLLMEEAERISRDEHGSTKMVVISGVGTRNYYRKLGYELDGPYMSK

'Tropilaelaps_mercedesae' PGARVNVLSKELMASVVLISGKKGCFIAGADITMLEECQSEAKRLKPVVAAIMGSCLGGGLETALACRYR-IAVEDSKTVMALPEVMLGILPGGGGTQRLPRLIQLPTALDMMLTGKNIRAAKAKKMGLIDATVKPLGPGIYLEEIAARNLASEKLKIRVRPLTERLVRDM-IFNKAREQVMKLTNGLYPAPLRILDVVRAGIEKQGFAELCTKEARSLMGLYHGQVQCKKNAFGKPTDNVAILGAGLMGAGICQVSLKDFNKVVMKDGLVRGQNQIKKKKIGKDKLMSKLLPSLDYSDMIIEAVFEDINVKHKVVKEVEAAHCIFASNTSALPITKIAEASKRPDKVVGMHYFSPVEKMMLLEIITTDKTSKDTAAAAVDVGLRQGKVVIVVKDGPGFYTTRILAPMMCEATVLLMEGCKVKELDKLKEFGFPVGGATLLDEVGVDVGAHIAEGVFGERKEMVSNFLGRKSGKGCYIYRPVNPIQRRYTVEQMQYRLATRFINEA------------------------------------------------------------------------MQRPIILLQEGTESQQGKTHVMSNINACQAISDAVRTTLGPRGMDKLMVDSRGKTVISNDGATIMKQLDIVHPAARTLVDIAKSQDSEVGDGTTSVVLLAGEFLKQAKPYIEEGLHPQIIAKAYRKASKMAIEKIHEIAVKVDKGEMKALLEKCAMTTLSSKLVAAKKEFFAKMVVDAVLQLDELLPLNMIGIKKVSGGALEDSLLVSGVAFKKTFSYAGFEMQPKQYNQPKIALLNIELELKAERDNAEIRVSNVEEYQKIVDAEWNILYDKLAKIHASGAKVVLSKLPIGDVATQYFADRDMFCAGRVAEEDLRRTMKACGGCVLTTVQDLRDSNLGSCERFEEVQIGGERYNIFKGCPNSKTVTLILRGGAEQFIDETERSLHDAIMIVRRAVKNDAVVAGGGAIEMELSKYLRDHSRTVAGKEQLLIAAFAKALEVIPRQLCDNAGFDATNILNRLRERHAKADRWAGVDVNSEDVADNLAACVWEPAVVKVNALTAATEAACLILSVDETIKAPQSNTDPSVGRPFSKTLNRENAFREHGMAVTRDYISQPRMIYKTVCGVNGPLVILDQVKFPKYAEIVQLVLADGTPRTGQVLEVSGDRAVVQVFEGTAGIDAKNTVCEFTGDILRIPVSEDMLGRVFNGSGKPIDKGPPVLAEDFLDIQGQPINPWSRIYPEEMIQTGISAIDVMNSIARGQKIPIFSASGLPHNNIAAQICRQGGLVKRPEKSVKDDNFAIVFAAMGVNMETARFFKQDFEENGSMDNVCLFLNLANDPTIERIITPRLALTTAEFLAYQCEKHVLVILTDMSSYAEALREVSAAREEVPGRRGFPGYMYTDLATIYERAGRVEGRNGSITQIPILTMPNDDITHPIPDLTGYITEGQIYVDRQLHNRQVYPPINVLPSLSRLMKSAIGEGFTRKDHADVSNQLYACYAIGKDVQAMKAVVGEEALSPEDMLYLEFLGKFEKNFISQGRYENRTIFESLDIGWNLLRIFPKEMLKRIQHSLLAEFYPRAEAKTGLFGDPTGFILLKENAIAKAEELTAEATSRRRKMVQIFDDLSDCLCKVADLAEFVRVGHPQVRYAHAAEDASLAISSLVEKLNTNRELYSALRSVIEKG-DIVPTTAEQHVGRLFLFDFEQCGIHLDEQRRQQVVALNDHILFVGGRFLQDSHQPRYILKSSMPENILVVSGLQADCSNEVVREAAYRIYFRPDEHQLHLLDELLESRHRLARLCGFDTYAHRVLKGSIAGTPENVKFLSYLSTELKPRSQRDYQEMLGMKAWDVPYYTAYLSLANCMEGLDMLFNALYGIKLEVVGELWHSSVVKVAVRDPSPTMGVIYCDLFERPGKPHQDCHFTIQGGRRSDGSYQTPKVVLMLNLPPPLLTPSLMDNLFHEMGHAMHSMLARTEYQHVTGTRCATDLAEVPSILMEYFASDPRVVSQFARHYRTGEPMPAEMAANLEASRVIFQASETQLQVFYAFVDHEYHSKYPLSTTDVLREVQNRHFGVGYVENTAWQLRFGHLVGYGAKYYSYLMSKAVAATFWHRVFHTNPFSRSAGTYREEVLAHGGALPPAQLIENFLLAESLIRDIMPSDAKKKRDAKKKEALKNRNNPDTPNGEDEMDEVTRKFDEDMKLNAAARAVTGVLSIHPRSRDVKIENLSITFHGWEVLQDSKLELNCGRRYGLIGLNGCGKSTLLSAIGRRELPVQDCLDIYHLTRECPPSEKTALQMVLDVDKERARLERLAEELASAEDDTSQEQLMDVYERLDNMSADTAQGKASYILHGLGFTQAMMSKKCKDFSGGWRMRIALARALYVKPHILLLDEPTNHLDLDACVWLEEELKSYSRILILISHSQDFLNGVCTNIIHMNLRKLEYYGGNYDQFVITRSEVLENQMKRYNWEQLQIAHMKDYIARFGHGSAKLARQAQSKEKTLAKMVAGGLTDKVVYDKTVSFYFPSCGTIPPPVIMVQNVSFRYSIDTPLIYKNLEFGMDLDTRVALVGPNGAGKSTLLKLLCGALVPTDGIIRTHSHLKIARYHQHLHESLDVDLSALEYMMKSFPDVREKEEMRKIIGRYGLTGRQQ---------------------------------------------------------------------------------------------------------MVLADLGRRITSALRNLSTATVINQEVLDSMLKEICAALLESDINVRLVKQLRENVKAAIDIEEMAVGLNRRKVVQSAVFKELVKLVDPGVKSWHPSKGRSNVIMFVGLQGSGKTTTCTKLAYYYMKKGWKTALVCADTFRAGAFDQLKQNATKARIPFYGSYTEVDPVVIAADGVAKFKTEHFEIIIVDTSGRHKQEDSLFEEMLEVSNAVTPDNVIFVMDASIGQACELQARAFKEKVDVASVIITKLDGHAKGGGALSAVAATRSPVIFIGTGEHIDDFEPFRVKPFIQKLLGLGDIEGLIDKVNELKLDENHELIEKLKHGEFTLRDMYEQFQNIMKMGPFNQIMGMIPGFSADFMSKGNEQESMARLKRLMTMMDSMTDEELDDREGAKLFLKQQTRITRVARGSGCTTFEVHELLNQYTKFAAMVKKMGGMKGLFKGNDLARNVNPAQMNKLSVEMAKMIDPRVLQQMGGFSGIQNMMRQMNASILTEDAAFIELKKLHSTKGKSLNLNTLFKDDLSRAGKYTIKLSEGPETLLIDYSKNLIDDEIFTTLLELAKNRQVETMRDRMFSGEKINFTENRSVLHVALRNRSNRPIEVDGADVMPGVNAVLEHMKIFCQQIISGEWKGYTGKKITDIVNIGIGGSDLGPLMVTEALKSFQVGPRAHFVSNVDGTHLFETLKKVDPETTLFIIASKTFTTQETLTNADSAKAWFLGKAGDKAHVAKHFVALSTNKAKVESFGIDPTNMFEFWDWVGGRYSLWSAIGLSIALFIGVPNFEKLLAGAHFMDEHFRTAPLEKNVPVILAVLGVWYINMFGAESHCVLPYDQYLHRFPAYFQQGDMESNGKYVQRNGVQVSYQTGPIVWGEPGTNGQHAFYQLIHQGTKLIPCDFIAPVKTHNPVQGGLHHKILLANFLAQTEALMKGKSSQEAKAELEASGINGEELAKILPHKVFQGNKPTNTIIVQQVTPFTLGALIAMYEHKIFVQGVIWDINSYDQWGVELGKQLAKVIQPELDGKDPVSSHDASTNQLINFIKSFNKNVSYQDRDKPAQVRQSNITAAKAVCDAIRTSLGPRGMDKMIQAVNGDVTITNDGATILQQMQVLHPAAKMLVELSRAQDVEAGDGTTSVVVIAGSLLDAASKLLIRGMHPTIVSDAFQVAAKECVDILSNLAISVELSDRESLLKSATTSLSSKVVSQHSDILAPMAVDAVLKVIDPNNVDLRDIRIIKKLGGTVEDTELIDGLVFTEKLAGGNAPHRMEKAKIGLIQFCISPPKPNMDHQVIVSDYSAMDRVLREERAYLLNIVKVVKKAGCNVLLIQKSILRDAVSDLALHFLAKMKIMVIKDIERENIEFITKSLGCRPIASLDHFVPESLGSAELVEEVTSAKYVKVTGVANP---KTVSLLLRGSNKLVLEEADRSIHDALCVIRCLVKKRALVAGGGAPEIELSLRLAERARQIEGLHSYCYRAFADSLEIIPYTLAENAGLNPIQTVTELRNRHAKGERTCGINVRRGCVSDITKENVLQPLLVTTSAITLAAECVRSILKIDDIVQTVRMSKRDVVYLWDPDVGNFHYGPGHPMKPQRIAVTHSLVLNYNLHTKMRIYRPYRANPHDMCKFHSEEYVNFIERVTPKNIQTFSKSLTHFNVGDDCPVFDGLYDFCSMYTGASIDGAWRLNNKSCDIAINWSGGLHHAKKFEASGFCYINDIVVAILELLKYHARVLYIDIDVHHGDGVQEAFYLTDRVMTVSLHKYGAYFFPGTGDMYEVGAESGKYYALNVPLKEGIDDASYYQVFKSVISSVIEHYKPGAIVLQCGADSLAGDRLGCFNLSIKGHGECVRFVRDLNIPLLVLGGGGYTLRNVARAWTNETAILVDEQVSPEIPYNEYLEFFAPDFSLYPD-------ENANSKQYLEAIIKYTTENLRCLDHAPSVQMQDVPPDLADIGDDQEKRDSTKEHPAELFD---------------MSDAEEDYELEYSEDDDSQPDVDLENQYYNSKALKEDDPQAALQSFQKVLDLEGGQKGDYGFKALKQMVKINFQLGHYEEMMSRYKQLLTYIRTAVTRNYSEKSINSILDYISTSKRMQLLQEFYEVTLDALRDAKNDRLWFKTNTKLGKLYLDREEWPRLARILRQLHLSCQNVDGSDDLRKGTQLLEIYALEIQMYTSQKNNKELKKLYEASLQIKSAIAHPLIMGVIRECGGKMHLREGEYNSAHTDFFEAFKNYDESGSPRRTTCLKYLVLASMLMQKEINVLDSQEAKPYKDDPEIVALTDLVDAYQAHDISRFESIVSPHKESIMKDSFIKEHIEQLLLNIRRQVLIRLIRPYTRITISFISRELNIPSAEVESLLVSCILDNTIHGRIDQVKQVLELHP-SPTDRRYTAMEKWAAQVQLIQNTVPMLLAHLGTLNPVQILNEQAEEEKAENARLSSFVGAIAIGDLLKSTLGPKGMDKILLCETSRDSKVEVTNDGATILKAIGIDNPAAKVLVDISKTQDDEVGDGTTSVAVLAAQLLQEAEKLIAMRLHPQTIIAGWRQAVVAARTALEDFSQDRSQNETQFRIDVLNIARTTLGSKILAQHKDFFAQLAVDAVTRLKGKCNLDAIHIIKKLGGSMLDSYLEQGFLLDKKPGMNQPRRVENAQILIANTPMDSDKIKVFGSRMKCDSIAKVAELEEAEKLKMKRKVDSILAHKCNVFINRQLIYNYPEQLFADAGVMAIEHADFDGIERLALVTGGEIVSTFTSPEAVQLGTCELIEEVMIGEDKLLRFSGVPVGEACTVVLRGATQQILDEAERSLHDALCVLASVVKEKKICYGGGSGEMLMAAAVEKLAQTTPGKEALAIEAFARALRQLPTIIADNAGLDSAKLVAELRAAHANGQNTFGINIVDAKVDDMQKLGVTEAFVVKRQVLLSAAEAAEMILRVDSIIKDAPRKRVPDKSH----MKSTDPFPFLCTLQQHRPYVGQVVLIGNADAVISVSDDRTIRVWARRDTGQYWPSVCYTMPAAASALNYDQPSRRLFVAMDNGSITEFELADDLNKIIFRRSYIAHQQRVTSIKFSPTTEWLLSAGKDKYFQWHCTETGRRLGAFQGSAWCTTVELDEASRHAFIGDYSGHVTMLKLTETSYQPVTTLRGHSGSVQSLLWDERRNLLISGSFDQVIIVWDIGGGKGTAYELSGHRARITGLALYSASSLLSVSEDSTLVVWDVAAQRQETPDWNERDFCERCARPFFWNVRAMLDQKQVGLRQHHCRRCGRALCDRCSANRSALPRLGFEFPVRICDECHLHISDGDRQPLASFHDLKAPVTAMSLSAASKTLVTVGTDRSIKIWDLSKVLSKKALKKAQKEAEKAAKKAAKKAERAAGKEDQSGDVSEGFYGQYHMIQSTDTPQREIIKVSQCTLQLADETIWLRGRLQTSRAKGKQCFFILREQQFTLQCLLAVGEKTSRQMVKYIANISRESLVDVQGKLVKSPSKIESCSQQEVELQVLQFWVVSLSDARLPLQVEDAARPEPAEGDEEGLKIRVNQDTRLDNRILDLRTPANQAIFRLQAGVCHLFRESLNRRGFIEIHTPKIISAASEGGANVFEVTYFKSKAYLAQSPQLYKQMAIAADFDKVYTIGEVFRAEDSNTHRHLTEFVGLDLEMAFKYHYHEVVDIIAAMFVDMFKGLRDRYQPEIDAINKQYPAEPFKFLDPSLRLEYAEGVAMLRAAGVEMADDEDLSTPNEKLLGRLVKAKYDTDFYVLDKFPLAVRPFYTMPDPTNEKWSNSYDFFMRGEEIMSGAQRIHDSEFLTRRAKAHGIDISTIQSYIDSFKYGAPPHAGGGIGLERVAMLYLGLDNVRKTSMFPRDPKRLTPAMEKGLQLNEIDPLEFSRVKIMYPKECRIRRVTYRGKLDLTFWWVNGLKQEPVKRSCGEIPIMVKSRKCNLAGLNPQQTVGHGEEMEEFGGYFVVNGNEKVIRLLIMQRRNYPIAMARNGWKNRGAMFSEFGVSLRSCRRDGQNMVLHYLTNGTVQVMITFRKEVYFVPALLLLKALVDKSDYEIYKALTKGCENDSFYKGCITNMQRLVQENIMTCDAKEFIGDKFRWASNAYVCDKLLDRCVCVHLDSNEDKFNLMCFMVRKLFAVAKNRCALESADSTMNQEVLLPGHIFLSVLKEKIEGLLIGVKLSIDKKMSSAMDVTNAMNYFLSTGNLVSKTGLSLQQTSGFTIIAEKLNFWRYLAHFRCIHRGSFFMEMRTTTVRKLLPEAWGFICPVHTPDGGPCGLLLHLSSMCEIVVQLDGKCVGWVIRYLKVSGDIPPTLEICVIPKTEQNSLFPGFYMFSTPARMMRPVFNRTQTIEWIGTLEQVHMDICVISEEATTHQELRETAMLSVLANQIPWPDFNQSPRNMYQCQMGKQTMGSPMHAFRNRADNKLYRLYPQSALVRPTAYDHFKMDEYPSGTNAIVAVISYTGYDMEDAIVVNKMSVERGFKTGVYKTETINLRVDVDGLPYIGCHGDPVCAYIQLKTVKYHSTEPATIHEVKILGNLQQIQLTYLVRTPMIGDKFASRAGQKGICSTLWPTESMPFTDSGMVPDIIFNPHGFPSRMTIGMVVESMAGKSAALHGYVHDASPFKFSEENPSSAYFGDLLRRAGYNYHGTERMYSGVDGREMDADIFFGVVYYQRLRHMVADKYQVRTTGPIDSLTRQPVKGRKRGGGIRFGEMERDSLLAHGTAFLLHDRLFNCSDKTLCIACGSVLSVPIPYVFRYLVAE-MVSVNMKIIRETSRDLHRHQRNAPELHPLAITREYTAAVNAAKLDRIFAKPFLCSLGHRDAVELLAKHPDRISGAVSASADGELRWWDLTNRMCVRSLQAHDGPIRGLTGQDQSIKTILSKLTHHYSTTFATAGETVSLWEERNEPLRSFW-GIDTVYVYFSPIESLSSDRSIVLYDIREASPLRKVVLEMRCNALAFNPMQAMHFTVANENYNLYTFDMRSLNKALQSHTDHVGAVLSVDYSPTGTEFVSGSYDKTVRIYRSREVYHTKRMQRVTSVMYSLDAKYILSASDEMNIRLWKAKASEQLGIQNYNATLLERFGYHPEVKRIVRHRHVPKQLYQEKQTMVAAKKRKMGMQLLQKGAYQDALSHYHAAIEGDDRNYQSYYWRATVYLALGKSKLAVEDLDRVVELKDDFVKAREQRGNILLKQGHLDEAHIDYEFILRLDPHNVEAIIEELKNDVIHILQRVWNLKFREMRASCYEAIGDIQAAITDLRPAIRSVPDNTGGYFRLAELYRKFGEPDDALNTIRECLKLDPDHKECYKNIKRLVKSMQEECVEKIVTLIRNKCQCASKGGNAVKICSEALQLDPILCDRGEAYINQDDFAQDFAAARELDSRAAEGLKRAQKLEKAQGRRDYYKILANKGEISKAYRKLAAKWHPDQY-QGDD--KKNAEKKFIDIAAAKEVLTDPEKRAKFDRGEDPLDPESGFHYTFHFMLVLFESPAGYAIFKVLDEKKVQKTDNLFKEFEDASGAAKVLKLKHFQKFEDMTQALSAATGAIEGKLCKPLKKVLKKLAANQAHETLAVADAKLGSIIKEKMDISCVANSSIQELMRCIRSQQEALITGLSQKEVTAMALGLAHSLSRYKLKFSPDKVDTMIIQAVSLLDDLDKELNNYVMRCKEWYGWHFPEMSKVVTDNMLYVKTVRKMGMRSNAINMDLSDLLPEDQEAKVKELAEVSMGTEIASDDVENIKHLCDEVIQMTEYRATLYEYLKNRMTAVAPNLTVLVGELVGARLIAHAGSLLNLSKQPASTVQILGAEKALFRAFKTKHDTPKYGLIYHAQMVGQSSQKCKGKASRWLAAKAALAIRVDALGDDTGTEMSLKNRANLEARLKMLEEGKLTKISKYVWPKERPDIRRTVTLALGILVTAKLVNVSVPFIFKYLIDFLNQTTGLVLAYGLARAGSAGLNELRNAVFASVAQHSIRSMGKRLFMHLHDLDLSFHLQRQTGALSKAMDRGTRGINFVLTALVFNVVPTIFEVALVSTILWYKCGQFAVVTLGCIGTLAVTQWRTQFRVDMNKADNRAGSRAIDSLLNYETVKYFSNEKYELSDYERASLKTTVSLAGLNFGQNAIFSAAVMYLACQLTVGDLVMVNGLLFQLSLPLNFLGSVYREVRQSLIDMQTMFALSEIKTKEIIISREIEFDNVTFQKILDGVSFSIPTGKKVALVGGSGSGKSTTVRLLFRFFDPSNGRVLINGQDIRDVSLRHGIAVVPQDAVLFHDTIHFNLSYGDLTAEVENAAKMAEIHDSIKAWYDTQVGERGLKLSGGEKQRVAIARAILKDAPILIFDEATSSLDSITEHKIMMALRAASGKTTLCIAHRLSTIVDADQIYVLRNGSIIEAGHQTLLASFYAHLWNQQHSQNIPETRVTTLANGIRVATEDNGAPTATVGIWIDAGSRYESEKNNGVAHFLEHMAFKGTGKRSQTELELEVENAGMHLNAYTSREQTVYYAKCLTKDLARAVDIIADITQNPKLGEQEIERERGVILREMEEVEGNLQEVVFDHLHSVAYQGTPLGLTILGPTANIKSITRQDLKDYIDCHYKGPRIVLAGAGGVDHDELVKIAEQTFGKVSASPENF--VPCRYTGSDVRVRDDDMPFAHVAIAIEGAGWTNPDNIPLMVANTMIGSWDRSHGGGANASSKLASLAATARSLHSFQSFNTCYKDTGLWGLYFVADGDELDDIMFAVQDEWMRICLSATESDATRAKNLLKTNLLLQLDGTTPICEDIGRQMLCYGRRIPLPELEARIDAVDAKAIRDVCLKYIYDRCPVVAAVGPVEGLTDYVRIRGQMYKHSVHTLVFRSLKRSHDMFLCDEGALPPVDETAQKFRIGTKARDEYGSVMHLVSEGRRATASGALVLAGQQLTAQLKKPSIPRPQWHPPWKLYRVISGHTGWVRCVAFDPTNEWFCTGSNDRIIKIWDLASGKLKLSLTGHISGVRGLAVSQHHPYLFSCGEDKQVKCWDLEQNKVIRHYHGHLSGVYTIGLHPTIDVIVTGGRDSTARVWDMRTKANIHVLSGHTNTVASVLVQATEPQVVSGSHDSTIRLWDIVAGKTRVTLTHHKKSVRALVLHPKLNMFASGAPDNIKQWMCPDGKFIQNLSGHNTIVNCLAMNEDGVLVSGGDNGSLQFWDWKTGYNFQKLTTPVQPGSIDSEAGIFAMGFDLSGTRLVTCEADKTIKIFKEDETASEETHPINWRPEIVTGSTSEYLINCSAEHYLEFLGINVKAKNFLVFQGAVESIAMKNPKERTVLFEEISRSMMVAEEETYQKKKGIAAERKEAQIEEAEKYQKLKEDVQVNLHLFRLFHQEEELQKKKRKDKIEAELKEKKKVEQNHRDDVELNKRKPAYIKAKEKTAHMQKKLDAAKKSLEAATKTHKSHQGIEELEHELSQVEEAFEQEDVSLEESQVKEYNRLKEKAGKMASAALQEYDSVAREQKTDQDHLDNELRKRNECEAKLKELEENQRRVNKLVDLHDLKEEEKKLEAKKRLTQKFEDVASLGDAKVDKHEDARRKRKSEIVEHFKKLYPGVHDRLVNLCHPIHKKYNVALTKVLGRNMEAIVVDTEKTGRACIQYLKEQMLEAETFLPLDYIDFKPLKERLREFRDVPNVKLLYDVLKYEPLSIKKAVLYATNNALVCETAEDAAKVAFQSPDGKRYDAVALDGTYYQKNGFISGGSSDLAKRAKRWDDKDFHKLKDQKEKLQEDLREAMKTARKESDLTTIESQIKGLETRIKYSKVKQLEQSMREREGRINEIKSRQNTVEDDVFRVFCEQIGVANIREYEERELHASQEREQRAELENQKNRIASHLEYER--TKDTLAVEQDQQELARLKDIEQKQKELIEQQMETISALKNERQSKKMKVDEIDEEVAEIRKRLTAQQKEVTGVQKTVTQAEARLEQKRSERHTLLQSCKLEGIRIPLIRGSMSQMYAREALQINYSHLRMEKEINLQRIQAPNFKAMEKLDSVKERLKDTDTEFEHARRKAKSNFELVKRERTCFEHVSNCIDEIYKSLTNNPSAQAFLGPENPEEPYLEGINYNCVAPGKRFQPMSNLSGGEKTVAALALLFAIHSYQPAPFFVLDEIDAALDNTNIGKVARFIREKTQTSFQCIVISLKEEFYGHADCLVGICPDPGECTISRIYTIDLSMSEAMLLYLGQAIRDGNIAEMQNIYEMSLVNLMDQYLIIIYKE--LYYRQIYAEERFSSYYNYVNLFNYILSSGPVSLDLPNQWLWEIMDDFVYQF--QSFSTFMWNVHSVLNVLHSLVQKSNINQQLEVFSRGGNPDEVAGEYGSRSLYKMLGYFSLIALLRLHSQLGDYYQAIKVLQHLELNRKGLSRVPACQVSTYYYVGFAYMMMRRYEDAIRTFSNVLVYIGRAKRSYQQRQMNNQTEKMYALLSICMVLHPQRMDESVLQQLKLKHMLKMSQGDIDTFERCPRFLSPVQWRAFRGEVLAQMTIRSFLKLYTTMPVEKLTKFVLMCFKHLMSNVVLDGEFQTGSDMDFFIDKDMIHIADTKIARKYGEFFAKQYNRFEEWYEMGPPDAILGVTEAYKKDSNPKKMNLGVGAYRDDNGKPYVLPSVRAAEQQLMLKSLDKEYLGIAGLAEFCKNSAALALGANSAVTKEGRNATVQGISGTGSLRIGAIFLDEFLKGNKTVYMPNPTWGNHIPLFKKCNFQVKQYRYYDPKTCGLDFPGALEDISRIPEGSVILLHACAHNPTGVDPRPEQWTEIEKVVRKRNLFPFLDMAYQGFATGDIDRDAMAVRLFAASG-PMCLSQSFAKNMGLYGERIGAFTLICDSAEEAARCMSQIKILIRPLYSNPPVNGARIANLILSDAQLRSQWLIDVKEMANRIISMRTRLRDGLKREGSSRDWKHITDQIGMFCFTGMTPEQVSKLTRDYSVYLTKDGRISVAGISSNNVDYLAHAMHNVTKRT-PGIFHLLHCMLMATVVFAFMNFSKPPDEVDPFSEYGVIFTIILYLFRLLPLLALPQSLTNLFGLTLYNAFPPKVRLKVKPHDAPFLCIRVVTRGDYPALVRENVERNLATCLETGINNFVIEVVTDKEVYVAVNSKIRQTVVPKSYNTSTGAMFKARALQYCLEDSVNSLAEGDYILHLDEETLLTKDALRGVINFISAGRHPFGQGLITYANERVVNWFTTSADMYRVADDLGKLRFQFNFFHKPLFSWKGSYVCTRFGAERDVSFDHGPDGSVAEDCYFSMVAYAKGYSFEFIEGALWEKSPFTVGDLIQQRKRWLQGIYLVVHSGKIPRRFKIWLSCSLYAWATMPLSTSNLLLAPTFPLPCPQAFNVVCAFIGALNIYMYVFGLIKSFSIRRHGFFGFWLCLVLVVLAIPLNIVVENVAVIWGLLGDKHKFYPWPHYHFSGKLRPGRITPKRPVPAHIARPDYADHPEGVPISEEAMKGA-EIKVLSKTEQEGVRKASVLARECLDVALAAAKPGVTTDELDRLVHEAAIARNCYPSPLNYYKFPKSCCTSVNEVICHGIPDERPLEDGDILNVDVTVYHNGFHGDLNETVFIGKVDEAAKKLVRVTYDALQKAIEACRPGVLYRDIGNIIQKHVQQNGFSVVKSYCGHGIHSLFHTAPSVPHYAKNRAVGVMKPGHCFTIEPMISEGAWHDEVWPDNWTAVTTDGKRSAQFEQTLLVTDRGVEILTSRREKNGQPWFMDQSTLRDSLKERSAKRRKLLAQQLGAGCAENLGLLLGNDKTSTTKQGTAPQEEEVMAYRDSSTFLKGTQSANPHNDYCQHFVDTGQRPQNFIRDVGIQDRFEEYPKLKELIKLKDELIRETATPPMYLKCDLLQYNLCELNGKFDVILIEPPLEEYQRSCGVTNIRFWSWEEIMKLEIEEVAAPRSFVFLWCGSSDGLDLGRQCLRKWGFRRCEDICWIKTNISNSKVKNVEPRAVFQRTKEHCLMGIKGTVRRSTDGDFIHANVDIDLIISEEPPFGMMEKPEEIFHIIEHFCLGRRRLHLFGRDLTIRPGWLTLGPELTNSNLNTEAYNAHFNTANDYLTGCTERIEALRPKSPPPKGKRVIMRVDFNVPLKDGKITNNQRIVAALPSVMHCLDKGAKSVVLMSHLGRPDGQPNENYTLGPVAEELKKLLVKPVIFLKDCCGSEVEAVCADPSPGSVILLENLRFHVEEEGKGVDASGTKVKADPSKVKAFRASLTKLGDVYVNDAFGTAHRAHSSMVGVELPQRAAGFLMMKELQYFSKALDKPARPFLAILGGAKVKDKIQLIENLLDKVDEMVIGGGMAYTFLKVNRGMQIGNSLFDEDGAAIVEKLLGKAAANNVKIHLPLDFITADKFHEDAATGTADISSGIPDGWMGLDCGPKSVELFAGAVARAKTIVWNGPAGVFEFDKFATGTKAMMDSIVGATARGAITIIGGGDTATCAAKWGTEDKVSHVSTGGGASLELLEGKVLPGVAALSDAMRTFGDRPVSFQLEDGGDYYYIGTEVGNYLRLFRGTLYKKYPSLWRRAVTVEERKKISQMNMSQHSAANFISLLKKSEVDDLIDGNEEKYRAAPVQENEGGGHGAKNARPSFMPAAPNNAHHLDAVPCSTPINRNRLQHKKNRSFPMLYDDLDPAMLHESAALPECLVPIRLDMEIEGSKLRDTFTWNRHEAHISPEQFAELLCDDLDLPPLLFVPQIAASMRQQIEAFPSDSLLEEQTDQRVLIKLNIHVGNISLVDQFEWDMSERANSPEEFAAKLCSDLGLGGEFVTAIAYSIRGQLAWHQRTYAFSEAPLGQLEMPFRAQSEAEQWCPFLETLTDQEMEKKIRDQDRNTRRMRRLANTG-MGANGSTIERDDVLPSSERYLGLVNFGNTCYCNSVLQALYYCKPFREKVLEYKAKNKRTRETLLTCLADLFHNIHSHKKKTGTLAPKKFIARLRKDNEVFDNYLQQDAHEFLNYLLNTIGDLLQAESSWVHDIFQGTLVNETRCLTCETVSSKDEDFLDLSVDISPNTSISHCLRGFSSTETLRGEHKYHCEQCNSKQEAQKSLKVKKLPPILALHLKRFKYTEQQNRNTKLSWRVVFPLELRLFNTSDDALNGDRLYDLVAIVVHCGTGPNRGHYISIVKSHGLWLLFDDDIVDKIDPSTIDDFFGLTQDTPKSSESGYILFYQSKEKSDVDHWIELGKQCRYLPEADLKKLCNMVCQILIEENNVQPVSSPVTVCGDIHGQFYDLEELFRCGGQVPDTNYVFMGDFVDRGYYSLETFTRLLTLKAKWPKKMTLLRGNHESRQITQVYGFYDECQQKYGNANAWKYCCKVFDLLTLAAIIDGEIFCVHGGLSPEIKALDQIRTIQRNQEIPHKGAFCDLVWSDPDEVDTWSCSPRGAGWLFGARATHEFMGYNALSLICRAHQLVHEGYKYMFDDKLVTVWSAPNYCYRCGNVAAVLEISDDQRKNPKIFNAVPDHERVIPERHAPYFLVDPVQPDESQDAEAATIDELKEHTAHIQKAVAQKESRFILRILRLLPATRKKLNSKLLRKTINGFYTHDKVHRELLLSFVDAEDTDMDPTQKTAHLALLPEVDVYLHLLLLVHMLDAANMERAVRCAELLKGKVEAHSRRSMDLLAAKSYFYYSRVYELDGNICMIRGFLLKRLRTATLRSDFEGQAVLINCLMRNYIHYSLFKQAAKLVSKVTFPEMASNNEWARYLYYLGCIKAIQLYYTDAHKNLLQAIRKAPQHSALGFKQTVYKLAVTVELLLGDIPDRTTFRQPALRKSLAPYFQLTQAVRNGNLGLFNQVLESYGSRFQADHTYTLIIRLRHNVIKTGVRMINLSYQRISLADVAAKLQLGSAEDAEFIVAKAIRDGVIEATIDHDKGYVQSAENIDVYCTGEPQAQFDQRISFCLDIHNQSIKAMRFPPKSYNKDLESAEERREREQQDMEYAKEEDDDTFQIAIRLTLALAALAARTLWQSSVTDMIENFRDSQPLLFEFLARLPEETAQGAILGTSVLLLCQSALASSLRMTAMRAIPVNLCIALL-DIVDEHACDAILSFLQHPDGHYPKMMADLLEQVTRCGPVVDAKRACGDHESLYSLLAGIGEIHTNLILENLLPDSTRRIELLLKILLDCVGTSGQYPSEETLSRIPITFWHILLDELGRVEPNTQMAKQLQPVYEQLVKLLRKCQLSDPGTMDSDEKEDLRCYRQDIAD--CYMSIATMLSSPVFYFFISALETAKNSKLIEACLFALNAIGDMADSEEDAPVVDAVLALLPRVPAGDEVLSQVMTAVGIFAEENIGPLVHLLLRGLQQTSAAASMALKDLARAHGDRLAPAANDILQAIAVLKHRDRVRLVAIVGHVVSALSSEQALMSLSALMAPFVQQLNEMTNLVEQLLPLFKLIAVKYSSCDPEVVSNLAECIRKAVPVLEVEVILSELLSMCGALLINATTFIDLHGCTDTVESFYKLGTLLKKFSLDDLVQLCPPLINIRCATQFVNASERSPIKEALENIIAQLIQ----------------------------------------------------------------KVKVKWKEVY-DVEVDLPEVFRAQLFALTGVIPDRQKVMFKGAILKDS-WGVTVLMMGTKDDVPQPTEKTVFMEDMDDSEISTAFKLPTGLTNLGNTCYMNAVVQCFKTVPELTGLEKFTGTLTSALRDLYRNMGSCTTAPLVLLHALHTLFPRFAEKGEHGQQDANECWTEMMRMLQNLIDQLFGGKLVSLKCMESEEESTEDFLQLSCFIS-SDVRYLIAGLKLRM-QESITKMSSTLNRDAVYKTSKICRLPAYLTVNLVRFFYKEKQSVSAKILKNVAFPMMLDVYELCETDLQQRLLPQREKFKKWDCEFSFEDDGSNNSGFYQLKAVLTHKGRSSSSGHYVAWIRSEWFKCDDDVVVPKEEILKLGGGGDWHVAYVLLYGPRILDGLINKKLPKELLLKIFSFLDIVSLCRCAQVSKEWNVLAMDGSNWQNIDLFSFQRDVTYDVVSYIAQRCGGFLRRISLRGCQNVPDQALSVFAQHCHNIEQVLTNCHKLSDDSVVSLSMSLHVDSCVELTDRSLRFF----NRLRVIDISWCRKITGQGIGMVAG--EQLLRFTAKGCLDNEAIIKLASKLQVLNLQCCPFLTDSAVVAVSQNCPDLRHLCVSGCTLLTDASPQALAVGCLHTLEMANCQRCGDAGLAPLLKACHDLRRLDLEECNLITDSTLNHVAAFCPLMEQLTLSHCDQITDQGVHKLAIQCIEIDNCPFISDTSLEYLADHLRRVELYDCQLITQDAIGKFQPEVRLHTYFAPATPRQRYCRCCVIASSKGARVYCPDHPGANLIEDYRAGDMICPQCGLVVGDRIVDVGTEWRVFQNEKSSNDPTRVGAAENPLLGGSDLSTIIGRTGDASDESGNAKYANRKTMSASDRALIGAFREISAMGDRINLPKTIMDRSNLLFKQVHDGRSLKGRSNDAIASACLYIACRQEGVPRTFKEICAVSKVSKKEIGRCFKLILKALETSVELITTGDFMSRFCSNLALPPSVQKAATHIARKAVEMDIVAGRSPISVAAAAIYMASQASAEKKSQKEIGDIAGVAEVTIRQSYKQMYPKAAQLFPEDFKPANEAPWVEKYRPENFSEIVGNEETVSRLEVFSRQGNVPNVILCGPPGVGKTTTILCLARLLLGSSFKDAVLELNASNDRGIEVVRNKIKMFAQTK-------------------------ALRRTMEVYSKTTRFALACNTSDKIIEPIQSRCAVIRFGKLSDAQVLAKVIYICRKENISYTEDGLEAIVYTAQGDMRQAIGNLQSTHVGLGHVNGENVFKVCDEPHPLIIKEIIEFCAKGDINEAYARMKTLHSMGYAAEDIISNMFRVTKSHELAEYIKLEFIKQIGLTHMTILQGLGSLLQLSALLANLCLVVLDKKYVKKEHSLVKPYQGSGMNMPNWDFTGTTMVSSNYIRLTRDVQSQQGSIWNKVPWEVQIQFKVHGTGKDLFGDGFAIWYTKDALQPGPVFGSRDFQGLGVFLDTYANQNGHHNHAHPYISAMINNGSLTYDHDRDGTHTELAGCEAKFRNSDYDTSISIRYEHDTLVVSTDIMGKKEWNECFRVTGVRLPTKYHFGVSAATGDLSDHHDIIGIKVFELDELREFIVPQAAPHRDHIDDSMSGTKFFFVVLFSMLFLMLYFYQKHQEKARKRFYRDQDSFDAIRDRNCRIQDINELFLPNDTVTHVPNIKRLNIDPVFPNRTNLLHIHNMAISKAFFFSFILQRAKDDEPGFMYYFMSVISDVAANRFINASAIYYAPNMSFTPSYKGFFNKTMPLFAPRAFRSDDFNDPYHLEGTSTLNTIEAIDLGAISQNYSSDQYRINEWYSAWLPDLTKRQDSKTTYTVQITGNNDTFVWHGPPAANDNPGPVKWVRPYFDCERSDKWVYGATSPIPDIYPRHTQWRHIEIPRYVAVSVMELDFERIDINQCPFGPGN-PRPNYFAGTARCKNDTTDCEPVHGYGFRRGGYQCRCKPGFRRPRIVRNPYHGELIERASKQEYENGYQCDKIGYIGVLTQNLNNYMAIGTRIDPFLGGDVVYGKDVQLENEARMAVRLANFVSGFLQIVDPKDLFAEFRVPDKPLSADQMIAEVMSIVIGDQKVVGAGVYFDYKAFFGPY--AWRQGRNERKYFVDDTTKIQIRYNSSGIKYDHFPLQYKAADVGYWTSPYFDCGGYHNSWMVTYAVPFFGWDSLRARLQFKGVVAVIELEQLEINQCNAFENTHKCDRKSSRCVPILGRGFQGGYKCECLQGYEYPYNDPITYFDGQIVEAPSRFERMTHVSGTDKSKYGGRFMVTALPGDGIGPELVGYVKEVFRYGGVPVDFEEVHLDSSRDDVDLLEQAITAVKRNGVALKGNIETRHNDPNCKSRNVELRLRLGLFANIVHVTSQPGIETRHSGIDIVLIRQNTEGEYSCEEHMSIKGVVESLKVITQLKSDEIARYAFEWAKNNGRKKITCVHKANIMKLSDGLFLRCCTEIAKDYPEIEFDNIIIDNCSMQLVSNPKQFDVLLLPNLYGNILTNLACGITGGPGIASGRNYGRDYAVFETGTRNTGKSIAGKNIANPIAMMNAGVDLLYHLNLREHAEVIASAIDKTINVDKIHTPDLGGQATTTEVVQNIIKEVQKSAMTYSGSSNEVILRDPPTDGISAVKFGQTSNQFLVASSWDGFVRLYDIQGERCRAKFDHGEPVLDTCFQGSGYVWSAGSDQSVRLFDINQGTELKAGSHDDAVRCIEYASDVSQIVTGGWDGAVKLWDPRRPVAPSSHSQDNKVYAIAIAGERIIVGTANRKVLIWDLRNMAFVLQKRDSSLKFQTRAIKAFPDKTGYVLSSIEGRVAVEYLDPSPEAQKKKYAFKCHRMKDSTKMEHIYPVNAIAFHTIHGTFATGGSDGFVNVWDGRNKKRLCQFHKFPTSISSLAFSPDGSALAIASSFQHEYRLESNPPADQIYIRHV--------------------MREYKPTDATTNPSLILQAAKLPQYAALIDKAVSYGKSPCQQLEEAMDKLFVLFGNEILKIVPGRVSTEVDARLSFDKDASIAKALKLISLYEELGISKERVLIKLASTWEGIQAARVLEEQHGIHVNMTLLFNFTQAIACAEAGATLISPFVGRILDWYVANTDKKSFEPLEDPGVKSVTKIYNYYKKFRYNTVVMGASFRNTGEVKALAGCDLLTISPGLLKELANSNEAVPQHLKAENAANLQLEKISVDEKRFRWDMNEDQMATEKLSDGIRKFAADARKLEALIQEKLKMGDSVCRPLATVYAESNEASGTNAGMPAVFRAPIRPDLISFVHHQLLKNKRTPYAVSKEAGHQTSAESWGTGRAVARIPRVRGGGTHRSGQGAFGNMCRGGRMFAPTKTWRRWHRRVNVTQRRHAAASAISASGVTALVMAKGHAIEKINEVPLVVSDKIQDYRKTKQAVGLLKNIKAWDDVEKVYKSKRLRPGKGKRRNRRYKKKCGPLVIYEKDNGIVRAFRNIPGVDTCDVNALSLFKLAPGGHAGRFIIWTESAFRKLNDIFGTFNKASKVKKGYKLPRPMMTITDLGRLFKSEEIRKELRQKKSIIISKKNKPNPLKRIHLLGRLNPYAIVEKRNYALTLAAESVKSAQKKRRQEKLKTLKMKRLQNPFPKNRKLNQGAVLKKTLDAIKDLINEGTWDCSASGVSLQAMDNSHVSLVALNLRADGFDKFRCDRNLSMGMNLASMAKILKCAENNDIITLKAQDDADTVTFVFESQNQDKVSDFEMKLMNLDSEHLGIPDTDYSVVVKMPSAEFQHICRDLSQIGDSVQITCTKDGIRFSASGDLGTGNIQLSQTADVEKEEEAVIIEMQEAVTLTFALKYLNSFTKATPLSAQVCLSMSADVPLVVEYNMGHLRFYLAPKIDDSEHHNVKVAVLGASGGIGQPLSLLLKQHPGISSLSLYDIAHTPGVAADLSHINTTAQVQGFVGADQLKDALKGMEIVVIPAGVPRKPGMSRDDLFNTNAGIVRDLADACAQVCPKAMLAIIANPVNSTVPIASETFKKRGVYDPARIFGVTTLDVVRANTFIAEAKGLDPMSLSVPVVGGHAGITIIPLVSQAEPKVEFPQDQLEKLTKRIQEAGTEVVQAKAGAGSATLSMAFAGARFVFSLVSAIKGKNVVECAYVKSDIGDAGYFSTPLILGKNGMEKNLGLGKLSKFEEEMVAKAMDELKKSVKKGVDFVSKEYEWLLKYEVNDIVEQLVIAECSKRFPL-TRSDKFVM-QSKVVATLTGDSISHADITLRL-PKHSQRTIVQNDAQWKLQQIQDAGNHLMQAMNLLRFKFTSGQEVRNLMSDVMTCVGRGRACLVVPKKRTIEEIMQSRNMKSLQPPLPSDVAVSFYIQSYKLVFAVYHV-QKDSQKF-DAECHVPWLSEVLVLFTVALQLCQQLKDKVEVFQYNDFLPMFIPLFLEHNAPISEISELIGACNVCFKDAESVL--NGFVSILVCLFCERLESTTTQPVSFN--VLYNFYEGLRVEVYCSLLRVAAGAVADVFQDVSVTKRWLPVEKARNVYRNIHAALSDMPLRVMVELLSTYEEHDAKEATQDAIKCISFAMGDPNTYLMDHLIPLKPIKALEGQPIHELLKIFVYGKLSEYREFYRKHKDVV-DQLQLDHEKNVEKMRYLTFMYLAEKNQEIPFDDIRREVEI--DDVEAFTINVLGTKMVTAKVNHLTQKVIVVSTMHRSFMRNEWEQLRDILRLEKVEQSTQQFLDYLG-TR-RKKEVEDLAEEANAIDEGEERAVITESPQFEELINILIEWINDELAKHRII--VKNVEDDLYDGQILHKLLEQLTNSRIDVVEMTQNEEGQREKLKVVLERASQALGLKWSVNAIHSKNVVAIVHLLVALARHFRAPVRARLPENVVVNVVSVTKR-EQLTRTYDEYGMKVE-RDAFDQLFDHAPDKLTIVKKSLLTFVNKHLSKIYVDDLD-REFHDGVKLAFLMGLLEGYFIPLYLILKP----------------------DNKVNNVTFAFGLMKDAGLKPKARPEDIVNYDLKSTLRVLYNIFMQLSHRGAKIIKDAEKDLRELLSIPLSYKVLFMQGGGTGQFAAVPLNLCPADYLVTGAWSQKAAKEAGQYVKYTKIPPASEWKLSPDSAYFYYCDNETIHGVEF-LVCDMSSNILTRPVDVSKFGVIFAGAQKNLGPAGVTIAIVREDLLSSASVCPSVLSYKTTAENDSLYNTPPTYAIYLLLVLKWIKSEGGVAEMARSEKKSQGLYDLIEQSRGFYFRSRITIPFRI----RCDDALEKKFVKEAEMIQLKGHRSVGGIRASMFNAMDVSQAARLYRDRPEWQDVKPVLKISYTEAFKDCFGYLCAVISEELSERVFELTTTCADENPACYTVWVLRRKLLEHLKKDLHEEMDFMSRQIFDNQKNYQVWYHRQRLVQLLELEFIDKILDAKNYHAWQYRQWLLKTFNLWDDELNFCTAMLKEDIRNNSVWNQRYFVLKNTTNFVVEDELKFTLDRISCNESAWNYLGGMLNIYLYATLLSEAQEIDIPRSSYWTFRQRMLAVVIDRFEELLEFVPHMSRFLTAQGVAHKFFLINQGDRLRFNRGALINIGYLASRAQCDYMVMHDIDLLPLNSKLSYRYPDKDNVHLAAPHLHPKYHYSTFVGGILLMRHEVFAQLNGLSNKYWGWGLEDDEFYIRAKEAKIQLERPHNIGSGVNDTFRHIHDARRRPRDMVRIGAQREESRKRDRITGLHNVVFQLQGVYRMQIAGVPVEVHNVQLHCDLNATPWCEKPRVSPGTTITTDGYMRGHGGLLSAVAGVVEKVNKLITVRPLKTRYNPEVGDVVVGRIVQVC--QKQWKVDVGGRLNAALHLHSVNLPGGELRRKSIEDELMMSQYLIDGDLVSAEVQNVGVDGGVSLHTRNLKYGKLGQGALVRVSPSLVKRCKHFHNLP-NGVHLIIGLNGFIWVTSSKFAREAICRTRNVILCLAKHNIMLYDTPIVYAYDIS-VAQLTRHRADKLVGGWLITCSGMVFGAVLLGGITRLTKSGLSMVDWHPLNEGRPRTAEWEAEFAKYQQFPEFKIRNKDMTMEQFKSIYWMEYIHRMWGRSIGAVFYIPAAVFWMIGYLRKGMKQRIVYCGALLAAQGLMGWYMVRSGLEEKPRVSNLRLAAHLGTAFVLYSLLLRTGFDHILPNESLVRFSRAMGLVFCTALSGALVAGIEAGLVYNSFPKMADRWIPSDILALPKWKNFFENATTVQFDHRLLGEMVLTALYIHSRKVPLPPRARLATHTMLAAWLQVGLGITTLLTYVPTPIAVSHQAGALTLLTTLLWVTHELKLIQR-LSR------------------------TEKNACQWSKDKLNSLFADLEIND-SIMCVVKELKKCEGEATANNRKAKLIFFYEWDLELCAGS-VVKGRVEIPNLSDENDIDEVTVNVILVDEKIKGMMRSKGIEVIRSKLEEYVSSLKTDFSQGLILPTKDSGVNTTATKGKTISTKQLTMEEFKCTADELYRAFTMVQAFTRGPAGGRFQMLDTNVEGKFVRLNELEFEWRFKSWPAEHYSLVLSIEQVS---------------------------------------MDPETFLEIANHVSKLKMYPYFEIAHCVVTLLYLREDLGSGSHLFSRKHPLSCWVSSMFSIYAGGIFAAFLLGEPVIAVFKSNQSLLLATACWYLIFYSPFDVVYKFCKILPIKLVIALAKEVTRAKKVHDGVHHAAKLYPNGYIIMIIIGVVKGNGSSFLKMFERLLRGFWTPQAMELMQPSFATKACAIAALVFVVDKKTDLISAPHSLVYFGVVCFFLYFKLSSVVLGLHDPSIPFENIACAMFFGGIWDAITKAIKDAATKKKDMFRPDGIVQRRTADGCDNDKETLLTLME----------EGYTSFWNDCISSGLRGCILVELGIRGRIDLEAAGMRRKSLLMRKVIVKNDAPVGDVILDEALKHIKETATPETLQNWVDYLSGETWNPLKLRYQLRNVRERLAKGLVEKGILTTEKQNFLLFDMTTHPLVDQNVKDKLIKRVQDSVLAKWVNDVHRMERRHLALLLLSHASDVLENAFNPLSDEEYEMAMRRVRDLLDMDFEAECAKSQTCDIMWGVFAAFVKMADGVPTFKCVLVGDGGSGKTTFVKRHLTGEFEKKYVATLGVEVHPIVFHTSRGTIRFNVWDTAGQEKFGGLRDGYYIQAHCAIMMFDVTSRITYKNVPNWHRDLVRVCEGIPIVLVGNKVDVKDRKVKAKSIVFHRKKNLQYYDISAKSNYNFEKPFLWLARKLIGDTNLEFVAMPALAPPEVQMDPEWQAKLEDDMKQAQNTVLPDEEDDDLLAAQITPILKESKFRESGMLTPEEFVAAGDHLVATCPTWAWAKG-DKSYLPEDKQFLVTKNVPCYKRCRDMEEKIIEDD-EGWVDTHEKEDDDEEGVDMDAFLDEDSEAAGILSTRTYDLNISYDNYYRTPRLWLTGYDEAFRPLTTEELYEDISQDFAKKTVTVENHPHIEGLPQASVHPCRHAQAMKNLIQTVEEGGGLQVHMYLIVFLKFVQAVIPTIEYDYTANFNMMSVLEKKLPYDKLSDNINIVKKRLNRPLTLSEKILYSHLDQPESEEIVRGQSYLKLRPDRVAMQDATAQMAMLQFISSGLPKVAVPSTIHCDHLIEAQVGGDKDLSRAKDINKEVYNFLATAGAKYGVGFWKPGSGIIHQIVLENYAFPGLLMIGTDSHTPNGGGLGGLCIGVGGADAVDVMANIPWELKCPKVIGVHLTGKMSGWTSSKDVITKLAGILTVKGGTGAIVEYFGPGVQNISCTGMGTICNMGAEIGATTSVFPFNSRMADYLASTKRRDIADAAEKVKDLLSADSGCKYDQIIEINLDTLEPHVNGPFTPDAAHPVSKLGQTAKEKGWPLDVKVGLIGSCTNSSYEDMSRSAMLAKQALDHGLKAKSLFTVTPGSEQVRATIERDGQAKVLKEFGGMVLANACGPCIGQWDRKDIKKGDKNTIVTSYNRNFTSRNDANPQTHAFVTSPEMVTALAIAGRLDFNPLTDDLVAADGKKFKLKPPVGDELPRSGFDPGEDTFQAPPSDGSNVKVDVDPKSQRLQLLAPFDKWNGKDLTDMVVLLKAKGKCTTDHISAAGPWLKFRGHLDNISNNMFIGAIPEESGEANKVQNRLSGKWGGVPETARDYKAKGQPWVVIGDENYGEGSSREHAALEPRHLGGRAIIVRSFARIHETNLKKQGLLPLTFADPADYDKIKSDDQISIVGLKDFAPGMESRGGMFKNTFQSGFLSILYSLGSKPLQIWDKKVRNGHIKRITDNDIQSLVLEIAGSNVSTAFITCPADPKETLGIRLPYIILIVKNLKKYFTFEVQILDDKNVKRRFRASNFQSTTRVKPFICTMPMRLDDGWNQIQFNLADFTRRAYGTNYVQTLRVQIHANCRLRRVYFADRLYAEDELPAEFKLYLPVQGLSASHRYQYMLKARNDIKRVIGKYRALAETFIFNDGSSKELVCLDGTIPVRYKYNIPIRVWVLDTHPYHAPLCYVCPTPTMQIKVSQYVDESGRVYLPYLHEWNSNL-SDLTGVIGVMVVVFGETPPVFSKGPSPYPTSNTLSITDEDIRISLLSAVESRITERALEKSKAEEDVLRKTNEDLQAGKQKLTRYMADMERDREMESEKMDQLKQLDVDSAVTTTAPLFRQLLQAYAEESAVEDAIYYLGEGLRKDVIDLDTFLKHVREQSRKQFMLRALMQKCRQKAGLPMGRKFYVGGNWKMNGTMETIQEICCRLKNSEPKTEVCVGVPAPYLQFVRNIVSSKIHIVAQNCYKVPSGAFTGELSVEMIKDCCSDGVLLGHSERRNVFGEKDELIAEKCALVLDNKLEVIACIGELLEEREAGKTEEVVFRQLKAYADKIKDWARVVIAYEPVWAIGTGKTATPDQAQEIHAKLRDWLSKNVSEEVSLNTRIIYGGSVTAANCKELAQKPDIDGFLVGGASLKPEFIDIINAKKMSSTVSKKRRFVADGVFNAELNEFLRRELAENGYSGVEVRNGATKTDIIIMATRTQDVLGEKGRKIRELTAVVQKRFNFKEGTVNLFAEKVSARGLCAITQCESLRFKLVGGLAVRRACYSVLRCIMEAEAMGCEVVVSGKLRGQRAKSMKFVEGLMIHSGDPTNHYVETAVRHVLLKQGVLGIKVKIMHPYDLQGKRGPALMLPDKVIVTVPKDEDDNVGINSDNKENAISKIEQIKQWSLSTYKCTRQILAEKMGKGTRTVDGELEANIELLRETHQKYLNILRLAKLLTTHFNTVATQAALGECFSDLAQK---ELQQEFLYNAETQKNLSKNGETLLGALNFFVSSLSTLCNKTIEDTLITIRHYENARLEFDAYRCEARANFKEKYERLRGDVQIKMKFLHENKVKVMHKQLLLLHNAVSAY-FSGNQSSLEATLKQFNISWLEQ-MVDLVLDRDIRIWVFLPIVVIMFFVGIVRHYVSILLTSSRKAELQQVYDSQALIRVRYLRENGKYLPARGFFMRKHFFNDEESGWLK-TQKRPPPINNPMSDPGMMSEMLKGNLTNVLPMIVIGGWINWTFSGFLTTKVPFPLTLRFKPMLQRGIELASLDASWVSSASWYFLNVFGLRSIYALVLGEDNAADSTRAMQDSMVPQAAAMPQDSKAAFKAEWEALEVVDHKWALAGIEDMLEQPGAASEAGRAMQILKRRERQKEEVELKRQKIEQEMRVS-MGDKFSSHFDAVEAQIKSATVGLVTLDEMKAKQEDAVKEREKRLAQKELEEKRRAEDKKRAQKEKQKKAIQALSFNFDDIRVKKNPDVDTSFLPDREREEAERMIREELRQEWKDKQKALKEENIQITFSYWDGSGHRRVVDMKKGNSIYQFLQRCLDALRKEFYELRVVSADQLMYIKEDLIIPHHYTFYDFIVTKARGKSGPLFAFDASEDIRMTSDASKEKEESHAGKVLLRSWYERNKHIFPASRWEPYDPTKCYDKYTIKDKKKNKSAVPETLLKQRKHNAELRQQRLLAAASKKKADRARRVLAFRRAEQYVREYRRIEASEKNNRLVAKVNGNFFVPDEPKVAIVIRIRGITGVSPKPKKVMQLFRLRQINNAMFVRLNKATINMLRIAEPYLAWGYPNLKTVRDLIYKRGFGRVNGRRAPLTDNAIIEEKLGRYGIICMEDLVHEIYTLGPNFKQVVNFLWHFKLNNPKGGWRKKTTHFVEGGDYGNRETLINSLLRKMIVKVDAKDLWKSVTSVSNAGRKRGRASGNSKKTSKDLNKGQKIGEGTVNMVWPGLNAPIVRGREIVKQGSLPPDLERTKRILALRDKQPNFRRLKLNPMERGWSGTKAQGRSLGPPDPIGGSEFPDFNSVILSLRLVSHMTGHLGRVRRHKAIVAVGNGKGLLGFADAKASVAKSALRRAKNKAFQRLCFYERFKDTVLHDFITEYGCTRIVVTKKERGFGLICHRALRDLCKLIGIKDMYAKVDTRNTNMLHLIRAFLLGLQKQKSHQDIAEEKRLHVVEFRPERSFFPEIIASPKNVRTSDEIGSYEELDINRYISGGKMIIQKPKKMPFYTRLPGWEIHLRKTDNLKNERMVRLRIFRKYGELKSFLNVRQPLMQKGARSSEGKYKWTNKQRLLIFASRGITYRDRHLMNSLRSLLAHSKEEVKFE--KKDINEIAEMKNCNKVMYMENRRKSDTYMWLANMRTGPTLKFLIQNVSTMEELKFSGNCLRGSRPLLSFDPTFNNNPLVKEVLAQTFGTPAYHPRSQPFFDHVFVFRLLDKRTWFRNYQIVEE-DGSLVEIGPRFCLNLIKIFDGPFSDVVIYTNPHYVAPNKIRRLAKKD--SRYVHLDVFETKHAYYRMAVGKNKGLSKGGKKGLKKKIVDPFTRKDWYDVKAPSMFAVRNVGKTLVNRTQGTRIASDGLKGRVYEVSQADLQTGEDAFRKFKLVCEEVQGRHCLTNFHGMDLTTDKLRSMVKKWQTLIEAQVDVRTTDGFVLRLFCIGFTKKAQNQVKKTCYAQHAQVRAIRRKMVETMQREVASCDLKEVVNKLIPEAIGKDIEKCCNTIYPLHDVHIRKVKVLKKPKFDMGKLLEMHGEGKGAGGGDGMAVDRPDNYEPPVLEDVAKIGIIGGSGLE-RPDLLKQKTPYGEPSDA-LITGKINEIDVVILSRHGRRHTINPSNVNYRANLFALK-LEGCTHILVTTACGSLKEEVRPGNFTPDSFIDRTRTQTFYVCHLPMTKPFLSDLLSKHPKGVVVCIEGPRFSTRAESAVFRQWGADLVNMTIVPEVVLAQELGIPYAALAIATDYDCWS-N--DTVDVQKVATLKKAADEACHILKTVLPKILNIAVLMRVLRHEEFETGCQAACNGRYDGFWSKTMVGYGSEDDHFVMELTYNYGVNSYKMGNDFRGILIQSDEILKRAALHKCPVELEKGRTYLKSPGGYKFYVDNLKS-E-GDPVKQVIYNCTNLAKTRRFWEDILWCEVLDSGDDFLEVTYDKSKTSLRFEKIIEPIDRAEAYGRVAFSCPRGQLEDIESQVKNSNGVVITPLVTLPTPNKPPVEVVILGDPDGHEICFVGDEAFRELSKEDPKSERMLEEKMA--EYDAFIKKTAATGRRFRLPPLPTPADLLRFYRLRALKQLSQNFLLDPICAKVVRSSGHVIEVGPGPGCLTRPILEQGAESVVVIEKDRRFLPTLKLLADATN-NRVKVVHGDVLLQELHNLI-PPEKAEPWEGIHLIGNLPFSISTILIVRWMKMVSERTGAWKFGRTRMTLTFQKEVADRITCPERCRLSVLVQGCYTKSGGSFVPPPMVNVGVVRVVPIEPLFQPFDIVEKVLRCLFNKEIRNDLVPEILKMANVDPTLFLDIEEFSRICDVYAKLCDEPGFSGFDYRGNPLIPIVIEQTGRGERAYDIYSRLLKERIICLMGPINDEIASVVVAQLLFLQSESPKKPIHMYINSPGGSVTAGLGIYDTMQYVLPPVATWCVGQACSAASLLLTAGEPGMRHSLPNSRIMVHQPSGGVSGQATDIQIHAEEILYLKRKVNLLYAKHTNQSIETIDAAMERDRFLSPEQSKEFGLIDTVLEQPPVRVERPVGTWLTLLPLWALTSAAPAATLPDAVLFCTGAFLMRGFGCTVNDMWDRDIDRQVERTRYRPLAASKLSRWDAFWFAGGQGLAALVLLQLNWHTVCLGLASLGIVVVYPLMKRFTYWPQAVLAIVFNWGVLLGFSAAALPMYLAAFSWTVIYDTIYAHQDKKDDLVVGIKSTALRFGTRTPIWLSAFTAMTTGLMGWPYFIAVTLKIDNIGLLVGCIGGTLLKIARVDRPKTRKGSKIVKAREPQLIENPKTSFIRAGNINQRTTQILKDLYTLKKTESVFYQKKNPFDDAVPLEKLAKNDTSLFALGSHNKKRPQNIVLGRTFDRMVMDQFEFGVENYKSLEEFKVPKISIMVKPILVFAGESWQELKRLKNFLIDFFKGDYIGVSGLEHVISFTALDILLRSYKVQLKKSGQTTPR-VEIYEMGPRMDLKLRRNKIASDDLFKQALRRPKELKAKKKKNLETDDLGTSFGRIHMERQDFKLQTRKMKGLKVRILEAFSDNYMYLVITKEAAVVDPAEPQKVISAVEVKLTTVLTTHHHSDHAGGNEQLLVYGGDARIKKLTHRDDMKITIGSFVQTLLTPCHTSGHVCYFIVFTGDTLFTSGCGKFFEGTAQQMQAAMLAALPPETRVYNGHEYTVSNLKFALHVEPGNKVSDKLSWAKFQPTIPSTIGEELTYNPFMRTDVQAHTDPVSTMAALRLEKDNFKMNRLFGKAKAKEPSPSLTDCISTVDQRANNVEEKIKKLDIELVKYKEQMSKMREGPAKNMVKQKAMRILKQKKMYETQRDNLMQQSFNMEQANFATQQLKDTKITVEAMKLGVREMRHEYKKINLNEIEDLQDDLEDMLEQANDVQETLGRPYGMPEIDDDELEAELAVLNDEIALEDTSYLDPVRSLSRSRGKATLPDLPYDYNALEPVISAEIMQLHHSKHHNAYVTNYNVAAAKLQEAVQKGDITAQVALQSALRFNGGGHVNHSIFWQNLCNPKTGEPSADLLAAIKRDFSSLEAMKEKVSASAVGVQGSGWAWLGYNKTTKRLQVTTCPNQDPLEATQGLIPLFGIDVWEHAYYLQYKNIRPDYVKAIWKVANWKDISQRFATAQDDHIQHDVIKEALESGRDLREYSRSVEQQLKGSEDEAIRDYMHNCRDIAALHGEILSCDAILQHMESILRGFQNDLGSISSEIQSLQRQSVAMNLQLKNRQAVRGELSQFVDDFIVPEATINVILDCPVTDEDFLIQLALLDQKISFVKVQSFKEASSCQDVKDILDKLKVRAVTKIREWLLQKVFSFRRANANFQLAQNTMLKHKRFFQFLATHEREVAKEIREEYVDTISKVYFSYFKAYQGRLMKLQFEDVPDRDDLMGVDDTPRWGLFNKPSLKNRSTIFTLGNRNAVLSSELEAPVIVPHASAKSEKHYPFEQLFRSMQYAFCDNAAREYLFVSEFFLLTKQGAADMFDNILEKSMTLFAKHTESFVADCFDSIALFLCIHIVHKLKVIMHVRNVPAMDRYWDLLAKIIFPRFETILRINITSIRDCDPSKLGSIDNRPHYITRRYAEFSAAIVSINENHPEERVSQLLGQLQIEVENFILRMAAEFNGRKDQLIFLINNYDMMLGVLQQRTHEDSKETTNFRGLLTARQSEYVEQILTVHFGGMITFIKESEYYLEKGQTEKLEKESQQVATLVRTFNSGWKKAIDNMNADIMKTFTNFKCGTSILQEALKQLLQYYHRFNKVLSQPPLCSLSVRSELINIHHLMVDIKKYKATFGYFIVVDAHSEECFHDRVVKGTKLGLTFEVAEGGFLDIDVKITGPDDKVVYNGERESSNKYTFAAYMDGQYKYCFSNAMSTMTPKTVMFTMDIGEEPKDDAK--GDGSENKLEDMISELHTAMTGVKHEQEYMMIRDRIHRSISESTNSRVVIWAFFENLVIIAMTLGQVYYLKRIFEVRRVVMRLSLILKLPKDYANLPESYVKRTMEQVEWRTPKGRQYRKAVIQKPYTMNRPWTDEFRNNRPGVKISVKAMFRGDRVEIMVGDDKGKLGTVNYIVQERNWVTVEGLNTRELPLIMGSQVKLVDPLDEKATDVQWRYTGSGQKVRISLRTGRIIPIPLAHETIDYKYIDRSNDTTSAELTKITFIP-KLATFEMDIMEQQGIEDNRVPQRTFWYVEQKMELLLWLSRLACAIFTFLYLLGNPFSLYQKALICNGVTSALRLHQRSPTVRLTAEFLGSVLIEDSCHYLIYSMIF-LPIS-MVLLPPMLFALLHSLQLLSRAGILHQ---SRVKAFRLIAMTEIFLFPTVVLGLFWGVSLMTPFMYYRFLKLRASSHRNPYTRNVFYELKLHATQFAVPNCPSIVSRVRFIESRADIAVVIIAVYLSFPEMRPEERPYVKLPTSLEDAKNLGRVLSNYTDKTVLLAFFCTYIFLQSFAIPGSLFLSFLSGFLFPFLLAITLVCLCSAIGASLCYLISYNVGRRLVLHYIPNMIYYIIFLRITPFLPNWFINITSPIVDVAIFPFFVGTFIGVAPPSILAIRAGTSLQQLASAFTWENILLLTGFAALSLVPVLFKLKSKFEEVQDKLTRIAIVSTDKCKPKRCRQECKKSCPVVRMGKLCIEVTPNDKIATISENLCIGCGICIKKCPFEAIQIINLPSNLERDTTHRYSVNSFKLHRLPTPRPGEVLGLVGTNGIGKSTALKILAGKLKPNLGRYNDAPDWTEILAYFRGSELQNYFTRILEDHLKAIIKPQYVDQIPKAVKGTVRELLDKKNDLGKEADLCELLELVNVMDRQIVDLSGGELQRFATAMVCIQKGDIFMFDEPSSYLDVKQRLKSAEAIRGQIEATKYVIVVEHDLSVLDYLSDFICCLYGTPGCYGVVTMPFSVREGINIFLDGFVPTENLRFRESSLVFKVSDNTDEDVKRIARYEYPSIMKKMGDFELQVRGGSFTDSEIIVMLGENGTGKTTLIRMLAGRLKPDGDEEVPTLNISYKPQKISPKSTGTVRYLLHEKIRDAYQHPQFVADVMKPLLIDNIIDQEVQNLSGGELQRVALALCLGKPADVYLIDEPSAYLDSEQRLAAAKVIKRFILHAKKTGFVVEHDFIMATYLADRVIVFDGKPSVSTVANEPQSLLVGMNRFLELLDITFRRDPNNYRPRINKLNSVKDSEQKRSGTFFFLEDEEDGEDLFGPELDRYDAAEEEMRRRDRGRLPRGLESIENLEDMKGHTVKEWVTQGPKTEIFNRFKNFLRTYKEKIRAMEQNRMSLEVDYTLAQSEQVLAFFLPEAPAEVLPIFDEAAKDIVVGMFPHYGRIHHEIRVRITDLPILEEIRTLRKIHIDQLIRTSGVVTSTTGVLPQLRMVKYDCAKCKYILGPFVQSQELKPSSCPECQSTGPFTINVAQTIFQDYQRITIQESPGKVNAGRLPRSKDAILLHGLCDSCKPGDEIEITGIYSNKFEGSLNKANGFPVFGTVIIANHILKKDTDEDVKEVVKLSKEELAERIMASIGPSIYGHDDIKRAIALSLFGGVSKNPGQKHRIRGDINVLLCGDPGTAKSQFLKYVQQIAPRAVYTTGQGATAVGLTAYVSPVTRDWTLEAGALVLADKGVCLIDEFDKMNDADRTSIHEAMEQQTISIAKAGIVTSLRARCTIIAAANPIGGRYDTFHQNVNLSDPILSRFDVLCVVRDERLARFVVDSHCRHHPINQDLLQKYILYAREKIEPKLDQDKIAQLYSNLRRESMATGSMPITIRHLESIIRLSESHARMHLREHVDVNMAIRVMLDSFVTQKFSVMRMGLLDDAKETFGANTLYDVFGVEKTVAADRLKKAYRKKSLLCHPDKAKQGHKEEFTKKFQILCKCFEILQDNNKRKLYDETGSV--DDSFNADKDWEAYWRCLFPKITVNQIQEFIDKYHGSEEEREDLKRSYEKSKGDMNKIAEYLIGYNEDRLTVLLLAATCALLLGKIGTKKLRKLEEKAERRRLRELELQEREERQQKQAEDERRRKEEQKKEDEEKRKEEEQRKQEEERERHEHEEYLKMKAAFAVEEEGYDEKDDL-NSSQKLSEFIAYVNDQKVVQLEDLAARFRLKTQDCIDRIQRLLEDGSLCGVIDDRGKFISITREELEEIARFIKIRGRVSIQELVENSNRLINLASIIMKQQEEAKALGLKHVLSQYQLPRESQFVEQHRKRVQRKKASVEAMLKTAVQSQLEGVRTGLLLLSAARDDVNDIQKDEADTIYGLSKLVQLQDVREESFRHSQTGTLMEHLKHIFNVPGSVARTQDLIQDGRLLLAHKLSDLECSRDELLYELHKQANNQASDRAMLKQYFADVERLSDELAKQLWLILKRTLNTVRREPQVIVTALRLIMREEWAALRRQEST-GFLPPGRPKLWRKKAMETLEQSVAERLEANQIEIRSENKMWLVRHLEVTRQLIIEDLKTVKHHCTPCFPSILDIFNEVRMTHNCLSQRLQTIIAGLVDSEYIHVLQWLNAYQSRELMGHPDLHVDMALLPDTIERLMERYLAGLNTKFEEWLRNALSDHKDWPEQDSHGYYRTEAPMLIYQMITQHVEVARTVTLVSRVLTLAMEQMNKFLSDYNQLVTDYNFEDRSYTAYMIAIANNAVNMK-LRELKENSLDYLCEEVLTDIKVMPDIMTETVTVTLADYGNDYIEKEVASNYVKAIC--EKRMSFRNYEERRSAAELEKLKNSKLDILKLMAEVLKMKDSTLLSLEMNRSRTDAEVVAREEEAIKLDSLSVAQLKEIREKAQKHAFQAEVARMMKLIINSLYRNKEVFLRELISNASDALDKIRLLSLTNPDALKALQDLSIRIMADKENNVLHITDTGIGMTQEDLMKNLGTIAKSGTAEFLQKVSEGGGGTDLNDLIGQFGVGFYSAFLVADRVAVASKHNDDPNQHVWESNASEFSIADDPRGDTLKRGTTVSLYMKDEAKDFLEHDTLKKLIEKYSQFINFNIYLWSSKT-VTEEVIEGEKPSEETAT-LDDDEEAKVEDAKEA--PKTKKVEKTVWDWELINSAKPIWTRKEKEVTDEEYNEFYKTVTRDNQDPLTRTHFTAEGELTFKSLLFVPQKQPQDSFNKYGQKTDHIKLYVRRVFITDDFQDMLPNYLSFLRGVVDSDDLPLNVSRENLQQHKLLKVIKKKLVRKALEMFRKIPETEYTKFWKEYSTNIKLGIIEDSANRSRLAKLLRFPSSLDSLDKLVSLSEYVQRMKDKQSAIYYIAGMGLDDVKKSPFVERLIKREYEVLYLTEPVDEYAISSLTEFEGKKFQNVAKEGLSLDD---NKEIREALEKEFEPLTKWMTETALKDKISKAVISERLVETPMALVASQFGWTGNMERIVAAQTHMKENDPQRMFYMGQKKTLEINPRHPLIKELLRRVDDSPSDETAKYLTEMMFETATLRSGFQLNDNVRFASNVERMLRKMMGVSEDAQVDAEPGRRGEVKELSPQELKMVVISEIIHELVRAHNDKRDVNLNRVKCDTSSRYGLKSQPKLVDIIAAIPPQFKQILLPKLKAKPVRTASGIAVVAVMCKPHRCPHINYTGNICIYCPGGPDSDFEYSTQSYTGYEPTSMRAIRARYDPFLQTRHRVEQLQQLGHDVDKVEFIVMGGTFMSLPDDYRDFFIRNLHDALSGHTSASVDEAVKYSERSKTKCIGITIETRPDYCLKRHLSDMLRYGCTRLEIGVQSVYEDVARDTNRGHTVRAVCETFHMAKDCGFKVVTHMMPDLPNVGFERDVLQFVELFKNPDFRMDGLKIYPTLVIRGTGLYELWKTGRYKSYPPALLVDLIAKILSLVPPWVRIYRVQRDIPMPLVSSGVENGNLRELALARMKDLGLVCRDVRTREVGIQEIHNKVVPYQIELVRRDYVANGGWETFLAYEDPQQDILVGLLRLRKCTDQTYRPELVGQCSIVREHVYGSVVPVHSRDPSKFQHQGFGTLLMEEAERISRDEHGSAKIAVISGVGTRNYYRKLGYELDGPYMSK

Speleorchestes ??????????????????????????????????????????????????????????????????????????????????????????????????????????????????????????????????????????????????????????????????????????????????????????????????????????????????????????????????????????????????????????????????????????????????????????????????????????????????????????????????????????????????????????????????????????????????????????????????????????????????????????????????????????????????????????????????????????????????????????????????????????????????????????????????????????????????????????????????????????????????????????????????MQAPIILLKEGTENAQGKSMVLTNINACQAVAEAVRTTLGPRGMDKLIVDSKGSVTISNDGATILKKLDIVHPASKTLVDISKSQDAEVGDGTTSVVLFGAEFLKQAKPFVEEGLHPQVIIKSYRKACNLAIEKLKELAVTIKETKKRQVLEKCAATTLSSKLVAHQKDFFSKMVVDAVCQLDELLPLEMIGIKKVQGGALEESVLVSGVAFKKTFSYAGFEMQPKSYKNPKIALLNIELELKAERDNAEVRVDSVQEYQNIVDAEWNILYEKLRKIHESGAKVVLSKLPIGDVATQYFADRDLFCAGRVQEEDLKRTMKACGGAILTTVADLTDANMGTCADFEEIQIGGERYNIFKGCPNAKTVTFILRGGAEQFIEETERSLHDAIMIVRRALKNDAVVAGGGAIEMELSKHLRDYSRSVAGKEQLLIAAFAKAFEVIPRQLCENAGFDATNILNKLRQKHAQGGVWFGVDVYTEDISDNFAACVWEPALIKTNAIVAATEAACLILSVDETIKSPKAQSETSAARRM??????????????????????????????????????????????????????????????????????????????????????????????????????????????????????????????????????????????????????????????????????????????????????????????????????????????????????????????????????????????????????????????????????????????????????????????????????????????????????????????????????????????????????????????????????????????????????????????????????????????????????????????????????????????????????????????????????????????????????????????????????????TGLFSDANGFYLLKENAILSAERLVREALSRKRKMVEIFDDLSNSLCLVADLAEFVRVGHPELRFQQAAEQTSIFISAEVERLNTNLELYVALREVVETGKDVAPTTLDNFVAKLFLFDFEQSGIHLDDDRRKEVVRLNESILHVGSYFVNNTNQPRTVPRSKLPEEVCIVNSLYPESENELLREAAYRIYLFPDDHQENLLQQLLLARKQLASLCGFKSYAHRAVKGSIADTPEVVEFIDCVTEKLMPLALRDYEAMFKLKPWDVAFYTPYFSIGACMDGLNLVFRELYQVQLNVCGEVWHKDVIKLSVSPSDEPLGFIYCDFLERAGKPNQDCHFTIQGGCLKGGVRQLPIVVLMLSLPPSLLNPHMVDNLFHEMGHAMHSMLGQTRYQHVTGTRCSTDLAEVPSILMEYFASDPRVVSRFAKHYVTGEEIPKNLLSSWIKSKKVFSASDTQLQVFYAALDQAYHSEDPLDTTAVLADLQNKYYGLPHVPYTAWQLRFGHLVGYGAKYYSYLVSRAVASSIWEKHFKADPLNFDSGKFYDQVLRHGGGKPAKEIVEGVLLANAIIDDV????????????????????????????????????????????????????????????????????????????????????????????????????????????????????????????????????????????????????????????????????????????????????????????????????????????????????????????????????????????????????????????????????????????????????????????????????????????????????????????????????????????????????????????????????????????????????????????????????????????????????????????????????????????????????????????????????????????????????????????????????????????????????????????????????????????????????????????????????????????????????????????????????????????????????MVLADLGRKITNALRSLGNATIINEDVLNSLLKEVCTALLEADVNIRLVKQLRENVRSVIDFEEMAAGLNKRRMIQMAVFKELVKLVDPQVKAWQPVKGRPNVVMFVGLQGSGKTTTCTKLAYYYQKKGWKTALVCADTFRAGAFDQLKQNATKARIPFYGSYTEVDPVVIAAEGVEKFRNENFEIIIVDTSGRHKQEASLFEEMLAISQAVDPHLVIYVMDASIGQACEAQAKAFKSKVDVGAVIVTKLDGHAKGGGALSAVAATQSPIIFIGTGEHIDDFEPFKVKPFVSKLLGMGDIEGLLDKVNDLKLEENTELIEKLKHGEFTLRDMYEQFTNIMKMGPFNQILSMIPGFGPDLMKGASEQESMARLKRLMTIMDSMSDKELDSSEGEKLFSRQPTRIARVARGAGVTQREVQELLTQYKKFAQVVKKMGGIKGLFKGGDLSKNVNQAQMAKLNQQMAKMMDPRVLQHMGGMQGLQSMMKQLQGA?????????????????????????????????????????????????????????????????????????????????????????????????????????????????????????????????????????????????????????????????????????????????????????????????????????????????????????????????????????????????????????????????????????????????????????????????????????????????????????????????????????????????????????????????????????????????????????????????????????????????????????????????????????????????????????????????????????????????????????????????????????????????????????????????????????????????????????????????????????????????????????????????????????????????????????????????????????????????????????????????????????????????????????????????????????????????????????????????????????????????????????????????????????????????????????????????????????????????????????????????????????????????????????????????????????????????????????????????????????????????????????????????????????????????????????????????????????????????????????????????????????????????????????????????????????????????????????????????????????????????????????????????????????????????????????????????????????????????????????????????????????????????????????????????????????????????????????????????????????????????????????????????????????????????????????????????????????????????????????????????????????????????????????????????????????????????????????????????????????????????????????????????????????????????????????????????????????????????????????????????????????????????????????????????????????????-------MDNDDEDYDLVYSEDSNSEPDVDLENQYYNSKALKEDDPKAALQSFQRVLDLEAGEKGEWGFKALKQMIKINFRLGNYAEMMARYKQLLTYIKNAVTRNYSEKSINSILDYISTSKQMELLQEFYETTLDALKDAKNDRLWFKTNTKLGKLYYDRGEFNKLAKILKQLHQSCQTDDGSDDLKKGTQLLEIYALEIQMYTAQKNNKKLKKLYEQSLHIKSAIPHPLIMGVIRECGGKMHLREGEYEAAHTDFFEAFKSYDESGSPRRTTCLKYLVLANMLMKSGINPFDSQEAKPYKNDPEILAMTNLVSAYQNNDIAEFEKILKNNRRTIMDDPFIKEHIEDLLRNIRTQVLIKLIKPYTRIHIPFISKELNIDEVEVENLLVSCILDNTIQGRIDQVNQVLELNRAGQSGCRYAALDKWTLQLSSLHQTILNKM?????????????????????????????????????????????????????????????????????????????????????????????????????????????????????????????????????????????????????????????????????????????????????????????????????????????????????????????????????????????????????????????????????????????????????????????????????????????????????????????????????????????????????????????????????????????????????????????????????????????????????????????????????????????????????????????????????????????????????????????????????????????????????????????????????????????????????????????????????????????????????????????????????????????????????????????????????????????????????????????????????????????????????????????????????????????????????????????????????????????????????????????????????????????????????????????????????????????????????????????????????????????????????????????????????????????????????????????????????????????????????????????????????????????????????????????????????????????????????????????????????????????????????????????????????????????????????????????????????????????????????????????????????????????????????????????????????????????????????????????????????????????????????????????????????????????????????????????????????????????????????????????????????????????????????????????????????????????????????????????????????????????????????????????????????????????????????????????????????????????????????????????????????????????????????????????????????????????????????????????????????????????????????????????????????????????????????????????????????????????????????????????????????????????????????????????????????????????????????????????????????????????????????????????????????????????????????????????????????????????????????????????????????????????????????????????????????????????????????????????????????????????????????????????????????????????????????????????????????????????????????????????????????????????????????????????????????????????????????????????????????????????????????????????????????????????????????????????????????????????????????????????????????????????????????????????????????????????????????????????????????????????????????????????????????????????????????????????????????????????????????????????????????????????????????????????????????????????????????????????????????????????????????????????????????????????????????????????????????????????????????????????????????????RRETKLDIHKVQRNDPALHPLELPREYKRALNAVKLDRMFAKPFIGALGHSDVVQSLMKHPTRLSVMVSGSCDGQIKLWNIPTGKCIRTIQASNSD-EELQDDSSNIKTVIANIDAHYKPLLMTCGERVDVWEERSEPLVSYW-NIDTIYVKFNPVEASGSDRSLILYDIRQKEPLRKVILALRTNALCWNPMEGMHFTAANEDYDCYTFDMRKLSEPLIIHKDHVQAVIDLDYAPTGKEFVTGSYDKTIRIFHSREVYCTKRMQRLTSVIWSLDNKYVVSASDEMDIRVWKSNASEKLGPKKYQDKLKEKFAHHPEIRRIANNRHVPKSVYKEHKIIERSQLRK--MTLLAKGQYGDALSHFHAAVEGDPKNYLTYFKRATVYLAIGKHRSALDDLNEAVNLKPDFHAARLQRGSALLKSGRLDEAHIDLEAVLRDDPTNQEASIEPIKYEAVDLLTKLWDVKLRELRSQSFEKLGDYVNAISDLRASTKMRADDTDGFLKLSKLHYELGEADESLSTIRECLRLDPDHKECYKKVKKLANAMTEDCVDKIVSQIKAKCHCLNKGKDGIKICTEAIQASPIYCDRADLYIHNQLYESDYRKAAQLDERAKEGIQRAGKLLKQSQRRDYYKILATKKEIMKAYRKLAQKWHPDNFASEDETQRKLAEKKFIDIASAKEVLTDEEKRQKFDMGEDPLDPEAQ-------????????????????????????????????????????????????????????????????????????????????????????????????????????????????????????????????????????????????????????????????????????????????????????????????????????????????????????????????????????????????????????????????????????????????????????????????????????????????????????????????????????????????????????????????????????????????????????????????????????????????????????KNVWPKERPHIRRRVLIALGLLVLAKVANVQVPFLFKYAVDYLNEKTGILIGYGIARAGASLMNELRNAIFAKVAHDSIRRVATSVFLHLHNLDLSFHLGRQTGALSKAIDRGTRGIQFVLSALVFNVVPTIFEVGLVSTILYIKSGEYALLTLGCIGTVITTQWRTKFRIAMNKAENEAGTQAIDSLINYETVKYFGNEKYELRKFEDASLKTTTSLAFLNFGQNAIFSAAIMIMATQMTVGDLVLVNGLLFQLSLPLNFLGSVYREVRQSLIDMQTMFSLLRVSSKPLLATPKVKFNDVHFSPILRGLSFEVPAGKKVALVGGSGSGKSTVVRLLYRFFDPKNGSVSIAGKDIRDVSVRQSIAVVPQDAVLFHNTILYNIRYGNLKAEAIEASRMAELHDSIERWYETQVGERGLKLSGGEKQRVAIARAILKNSPILVFDEATSSLDSITEHKIMNALKASQNRTSICIAHRLSTVVDADLIYVLKDGRVLEEGHLSLVQSFYAYLWTRQHQVNVPETLVTEIENGIRVATEDSGLQTATVGVWIDAGSRWETTENNGVAHFLEHMAFKGTQKRTQTDLELEVENMGAHLNAYTSREQTVYYAKCLSEDIPKAIEILADIIQNSKLGEAEIERERGVILREMQEVETNLQEVVFDHLHSVAYQGTPLGLTILGPTENIKKINRQDLLDYINTHYKAPRM-------------------HFGKVPALYQGT--KHCRFTGSDIRIRDDDMPFVHCAIAVEGCGWENPDNIPLMVANTLIGSWDRSMGGGTNVSNYLALVAGNQQLAYSFQSFNTCYKDTGLWGVYFVSDKMKCHEFVWHLQKMWMTLCTDVKEAEVERAKNLLKTNMLLQLDGSTPICEDIGRQMLCYGRRIPLPELIARIDAVSVDTLRDV?VKISVDKCSVTGAGSPFLSL-----------????????????????????????????????????????????????????????????????????????????????????????????????????????????????????????????????????????????????????????????????????????????????????????????????????????????????????????????????????????????????????????????????????????????????????????????????????????????????????????????????????????????????????????????????????????????????????????????????????????????????????VIGSSSEFRIDVNKQEYLEELGINVKAKNFLVYQGAVESIAMKNPREITALFEEISHSMDRSDEELYVKKKGIAAEKKEAQGEEAKKYQQLKEDLQVELQLFKLYHIQEEMEKKKKKEKIESDIKEKKQIDRNIRDELNLNKKRPSYIKAKENASHIEKKLETAKKSLNQAKKAHQNHEKIEELEEELDRVKKEFIAEDLELQEEQRREYDRLKDEAARTSAKYLKDLDSLEREQKADGDKLENETRRQNEIEAKIRERQENIKRIEKLKDLKELEQKEHEIDAKERITKELESLSQLGDAKVDRHEQERRKKKEEVVDHLKKLYPGIYGRMINLCKPIHSRYNMAVTKVMGKSMEAIVVDTEKTARQCIQYLKDQMLEPETFLPLDYITCKEVIERLRSIQHPRNVKLVYDCLKYDPPAIKRAVLHATNNALVCETPEDASIVAFDLGDGRRYDAVALDGTFYQKCGFISGGSAELERRARRWDEKDIYKLKISKEKLTEELKEAIKKTRKESDLMVMQSQLNGLKTRLKYCTIDEIKSRMAKREERINEIRDSMNNVEDQIFADFCRQLGVQNIRQYEERQSKDRQEKARRLQYENEIHSLQSRKEYEK--SKNTLEVKEEEKNLEQARETERREMKSIEEEMNKVEEYKNQKIEKKTQCDRIEDEISEIKRGLSGIQKEISTIQKALMQIECRLESKKADRHAILLHCKMECIDLPLEDGSLQKSYQADNIRPDFGILEMQSEINMRKIQAPNMRALERLEGVKDKLKETDNELNNLRKTAKTNFERVKRQRECFESVAQKVDSIYKSLTNNASAQAFLVPENPEEPYLEGINYNCVAPGKRFQPMSNLSGGEKTVAALALLFAIHSFKPAPFFVLDEIDAALDNTNIGKVARFIREKTESQFQCIVISLKEEFYGHADALIGVAPDPGDCTISRVFTVDLSVPDLVLKLLKKAITERNVFEIQNLYENTFVTLTEKHFLILYKE--LYYRHIYAENRFESYYNYCHLFNYILSAAPVPLELPNQWLWDIIDEFIYQF--QSFSLFVWNVHSVLNVLHSLVDKSNINEQLKEVNANRDPYAVTGVFGRQPLYQTLGYYALIGLLRLHSLLGDYYQAIKVLENIDLNRT--ARILACQMTTFYYVGFAYMMMRRYSDAIRTFSNMLVYLQRTRRTLQAEMIEKQTDQMYRLLTICLVLQPQRIDESVASVLQEKNMIKLQRGDLDEFQNCPKFVSPVQLEVFMKEVTQHLTIRSYLKLYTTMPISKLAAFMLLCFKHKMQNLVLEGEFQTGSDVDFYIDKDMI---------------------------MGPPDPILGVTEAYKRDTNPKKINLGVGAYRDDNGKPYVLPSVLMAEEQMMAAKLDKEYAPISGSPEFCAEAAKLAFGSDSDVIKEGLNATVQGISGTGSLMIGGIFLQAFWPHNKEIYLPTPTWGNHIPLFKRSGFAVKQYRYYDPKTCGFDFDGALQDLAKMPEKSVVLLHACAHNPTGVDPKPEQWQEMSKVIKSRNLLPFFDMAYQGFASGDIDRDAQALRLFVREGHRVLLAQSFAKNMGLYGERCGAFSLIASSKEEQAKVLSQLKILIRPTYSNPPIHGARIAQRILSTPELRKQWLVDVKGMADRIISMRTMLRKGLEKEGSSRNWQHVTDQIGMFAFTGMNAEQVERIIKEFSVYLTKDGRISMAGVTSKNVEYLAHAMHQVTKQLTSRQKHILHCCLYCCVILGFEYASDDPEALDPFEAYGTFMALFLYFLRFLALLSLPQVICNALGLTLYQAFPSKVEAKGSPLVAPFICIRTVTRGMFPDLVKQNVSRNMNTCLAVGLENFVIEIATDRPLNIQKHPRIREVVVPPEYKTKNGSLFKARALQYCLEEDVNILGDDDWIVHLDEETILTENAVRGILNFVYSKKHEFGQGLITYANEEVVNWLTTLADTCRVADDMGKLRFQFYMFHRPLFSWKGSYVVSKFKAERDVSYDHGPDGSVAEDCYFSMIAYKKGYTFDFIEGELWEKSPFTIKDFLQQRKRWLQGIFLVVHSDKIPIQNKIFLAMSLYAWMTMPLATLSLFLSTFYPLPPTAWCNFLSAFVGATWIYMYIFGVFKSFSLMRLGVKRFIICIIGALCAIPFNIVIENVAVIWGLFGSKHKFY---NYNFTGNLRPWPQTPMRKVPDTIERPDYADHPDGRSKCEEQVKGSGVIKVLDEEEIEGVRLASKLAREVLDEAYKAAGVGVTTDEIDRIVHEACIDRECYPSPLNYYNFPKSCCTSVNEVVCHGIPDTRPLQDGDLLNVDITVFHRGFHGDLNETFFVGNVDAESKRLVKATWECLQKAIAIVKPGEKYREIGNVIEKHARANGFSVVRTYCGHGIHRLFHTTPNVPHYAKNKAVGVMKPGHVFTIEPMINMGNWRDTTWPDDWTAVTLDGRRSAQFEQTLLVTETGVDILTRRRDKNGQPYFMDQSDVMTALRRLSKKRRELLVKQFGTGTFENLAKLLGNGMEP---DTDESEIDTLLTYTDSSTFLKGTQSANPHNDYCQYFVDTGQRPQNFIRDVGLQDRFEEYPKLKELIRLKDELISRTATPPMYLKCDLRNFNLSELGSKFDVILVEPPLEEYQRTAGVINMDLYAWDDIINLPIETVAAQRSFIFLWCGSSDGLDLGRQCLKKWGFRRCEDICWIKTNLKHGHSKNLEPKAIFQRTKEHCLMGIKGTVRRSTDGDFIHANVDIDLIITEEPEYGCIDKPEEIFHIIEHFCLGRRRLHLFGRDSTIRPGWLTVGPALTNSNFHAETYANYFTTPNGHLTGCTDRIEALRPKSPPPKDKRVLIRVDFNVPMKDGKITNNQRITAALPTIQHCLKNGAKAVILMSHLGRPDGQKMEKFSLRPVAVEVGKLLNTNVSFLDDCVGQAVEQACANPSSGSVILLENLRFHVEEEGKGVGPNGEKLKASDSDVAKFRESLTKLGDVYVNDAFGTAHRAHSSMVGVNLPQKAAGFLMKKELEYFAKALENPEKPFLAILGGAKVKDKIQLIENLLDKVNEMIIGGGMAFTFLKVLNNMKIGKSLFDEEGAGIVQKLMDKAKSKNVKVHLPVDFVTGDKFAEDATPGYANVEQGIPDDQMGLDIGPKSVEAFAAVVQRAKTVVWNGPPGVFEFDKFANGTKGMMNAVVAATKSGATTIIGGGDTATCCAKYNTEDKVSHVSTGGGASLELLEGKVLPGVAALTDV------------------------------------------------VTNEERKKITALGLGPHGLATNITLLKASEVDEIFSGFDEKYKALSVSTEPTVPREPKQRKQNWNISMPNSSHHLDAVPCSTPVYRSRLAHKKVRTFPLLYDDLNPEQLQENANQPEVLIPIRLDMEIEGHKLRDTFTWNKNEQIITPEQFAEVLCDDLDLPAASFVPAIAQSIRTQIEAFPTDNILEEQTDQRVVLKLNIHVGNISLVDQFEWDMSEKDNSPEGFALKLCQELGLGGEFVTAIAYSIRGQLSWHQRTYAFSEAPLPTVEVPFRNPSDADQWCPFLETLTDAEMEKKIRDQDRNTRRMRRLANTAW????????????????????????????????????????????????????????????????????????????????????????????????????????????????????????????????????????????????????????????????????????????????????????????????????????????????????????????????????????????????????????????????????????????????????????????????????????????????????????????????????????ISDLDRQIEQLRRCETIKESEVKALCAKAREILVEESNVQRVDAPVTVCGDIHGQFYDLKELFKVGGDVPETNYLFLGDFVDRGFYSVETFLLLLALKVRYPDRITLIRGNHESRQITQVYGFYDECLRKYGSITVWRYCTEIFDYLSLSAIIDGKIFCVHGGLSPSIQTLDQIRVIDRKQEVPHDGPMCDLLWSDPEDTQGWGVSPRGAGYLFGSDVVQQFNATNNIDMICRAHQLVMEGFKWHFNETVLTVWSAPNYCYRCGNVAAILELDENLQRDFTIFEAAPQEARGIPCKKQPYFLMKPAE--SMADPDLQTIEDVKEQARQIEKSVNTKEPRFMLRVLRSLVTTRKKLNARVLRKVVSGFYTHSPQQRDELLAFIEPMDTDAASPSKTEKLPLTPEVDIYLHLLVLLYLVDLSKKQEAVKCADLLMKKVEGQNRRTLDLLAAKCYFFYMRAYELTNQLNKIKSILHQRLRTATLRNDHEGEAVLLNCLLRIYLHYNLYDQAAKLVSRSTFPDTASNNESARHLYYLGRIKAIQLEYSEAHKNLLQAIRKAPQNTAIGFKQTVHKLAVTVELLLGDIPDRSLFRQAHLRRPLFPYFQLTQAVRAGNLAQFAQVLLRYGPQFQQDHTYTLIIRLRHNVIKTGIRMLNASYSRISLNDVARKLALDSAEDAEFIASKAIKDGVIEAMIDHDQGYLQSKDSIDIYSTGEPLSAFHERITFCLDLYNSSVKAMRFPPKSYSKELESAEERREREQQDLEYAKEDDDDEF??????????????????????????????????????????????????????????????????????????????????????????????????????????????????????????????????????????????????????????????????????????????????????????????????????????????????????????????????????????????????????????????????????????????????????????????????????????????????????????????????????????????????????????????????????????????????????????????????????????????????????????????????????????????????????????????????????????????????????????????????????????????????????????????????????????????????????????????????????????????????????????????????????????????????????????????????????????????????KVKVKWKETYNDVELNTVTLFKEILFSLTGVQPDRQKLLLKGKEIKND-WGMMLLMMGTPDPLPQPAEKPRFLEDMDESEVASALDLPAGLNNLGNTCYINATVQCLKTVPELRELNAFTGALIAKLRDLYRNMDNTEIAAFFMLSAIHAAFPRFAEKGENGQQDANECWTELLRVLQSVIDQYFGGTFVEMRCDEAPDESTENFLQLSCFIS-QEVKYLQSGLKLRL-QETLTKRSPTLNRDAKYKTSKICRLPAYLTVQFVRFFYKERESVSAKILKDIKFPLVLDVFELCSESLQQKLLPMRAKFKELEDLYQFPDDGSNNSGYYSLQAVLTHKGRSSSSGHYVAWIRREWFKCDDDVIVSEEEITKLSGGGDWHTAYVLLYGPRQLEALINKKLPKELLLRIFSYLDVVSLCRSAQVSKAWNVLALDGSNWQRVDLFNFQTDIEGPVVENISRRCGGFLKKLSLRGCRSVTDASLKTFAQNCNNIEDLLNDCKKLTDSTCQYLSLHLNVGSCSEITDLSLKALGEGCRNLEEINISWCEQITKEGVEHLAKGCPKLRAFIAKMCINDDAVKVLTWNLEVINLYGCSTITDEATISISQNCPRLCYLSVSNCPHLTDMTLNSLSQGCLKTLEVAGCSQFTDAGFQALARNCRLLENLDLEECVLITDNTLAYLAAGCPNLRKLSLSHCELITDDGIRQIGLSVLELDNCPLITDSSLEHLISCLQRIELYDCQLITRAGIRRLRPDLKVHAYFAPVTPRQRYCRCCVIL?????????????????????????????????????????????????????????????????????????????????????????????????????????????????????????????????????????????????????????????????????????????????????????????????????????????????????????????????????????????????????????????????????????????????????????????????????????????????????????????????????????????????????????????????????????????????????????????????????????????????????????????????????????????????????????????????????????????????????????????????????????????????????????????????????????????????????????????????????????????????????????????????????????????????????????????????????????????????????????????????????????????????????????????????????????????????????????????????????????????????????????????????????????????????????????????????????????????????????????????????????????????????????????????????????????????????????????????????????????????????????????????????????????????????????????????????????????????????????????????????????????????????????????????????????????????????????????????????????????????????????????????????????????????????????????????????????????????????????????????????????????????????????????????????????????????????????????????????????????????????????????????????????????????????????????????????????????????????????????????????????????????????????????????????????????????????????????????????????????????????????????????????????????????????????????????????????????????????????????????????????????????????????????????????????????????????????????????????????????????????????????????????????????????????????????????????????????????????????????????????????????????????????????????????????????????????????????????????????????????????????????????????????????????????????????????????????????????????????????????????????????????????????????????????????????????????????????????????????????????????????????????????????????????????????????????????????????????????????????????????????????????????????????????????????????????????????????????????????????????????????????????????????????????????????????????????????????????????????????????????????????????????????????????????????????????????????????????????????????????????????????????????TLEQLRKMTIVVADTGDFESMKRFKPTDATTNPSLILQAAKLPQYQKLIDEAVEFGKNNGNDLEATMDMLFVLFGCEILKIIPGRVSTEVDARLSFDVQGSVNKALNIIKLYEKRGISKDRILIKLATTWEGIKAAEILERDHGIHCNMTLLFNFAQAVAAADAGVTLISPFVGRIYDWFVKSTGVKVYEGYDDPGVKSVTRIYNYYKKYGYKTVVMGASFRNTSEIKCLAGCDLLTISPSLLEQLNETGGNLTQALSVEKAKAAELERRTFDEKTFRWELNEDEMATDKLSEGIRKFAIDARKLEDLIRSKLN??????????????????????????????????????????????????????????????????????????????????????????????????????????????????????????????????????????????????????????????????????????????????????????????????????????????????????????????????????????????????????????????????????????????????????????????????????????????????????????????????????????????????????????????????????????????????????????-----------------------------------MDTSHVALVSVKLKAEGFDMYRCDRNQVLGTNLNSLSKVLKCANNDDTITLRAQEDGDTLTMIFEAKDESELHEYEVKLMNLDTEHLNIPVTSYQVTAKLPSAKFQRICRDLSQIGDSVTISCAKDGIRFSTQGDIGSCTIRLKQTTDPDKPEEAVSIKMTEPIIQSFALKYLDKFAKATPLSSHVLLCMSPDIPLTVDYIIGSIRYFLAPKIDDDDADLNKVAVLGASGGIGQPLSLLLKQNKLVSHLSLYDVANTYGVAADLSHMNTRARVTGHLGPDQLDEALQGCDVVIIPAGVPRKPGMTRDDLFNTNASIVRDLTDACARNCPNALVGIISNPVNSTVPIASKVYEKRGV-DASRIFGVTTLDVVRANAFVAELKGLDPEQVNVPVIGGHAGITIIPLISRATPSVSFPQDQLDALTKRIQDAGTEVVKAKAGTGSATLSMAFAGARFAISLLEAINGKNVVECAYVRSNVTEATFFSTPITLGKNGIAQNHGLGKLSPYEEELVKAAIPELKASIKKGEDFVK-????????????????????????????????????????????????????????????????????????????????????????????????????????????????????????????????????????????????????????????????????????????????????????????????????????????????????????VP-FIDLFASIGAEISDLRDIINVCDSCFKDVESVL--NSIVS-LLIAFCQKLNA-ADPKVCIR--VLQNLYEGIKYEVYLALVEVAGDQIHLVFNDINKLKSTFGVDRIQRLLRQLHRVLSELASKVMIELLSTYTEEHASHAKDDAQRCIVSFIADPGTFLMDHLLTLKPVKYLEGEKIHDLLTIFVSDKLSSYINFYNANKNFV-DSLGLNHEQNLQKMRLLTFMQMAETKKEIPFQAIQEELQIESDQVEEFVIEVLRTKLVRAKVDQVNKRVLVTSTMHRTFGRPQWEKLRDTLNLAHVQRTVHT--------------------------EYEERSMIEDQPSFQELVKILVNWINDELNDQRII--VKDLEEDLFDGQILGKLVEKLSGIKLDVVEVTQNEEGQKHKLRTVLDAVNKLLGVKWSVEGIHGKNLVQIIHLLVALIRHYRAPI--RLPQNVSVNLVVVQKR-EQLTESYDELGMRVEPRDAFDTLFDHAPDKLQVVKRSLVTFVNRNLNRINIEELDPNQFSDGLLLCFLMGMLEGYFVPLGLFTTP-TDPIEDVTGKETALKPENYINTSKLHNVNIAFELMEEAGI---------------------------?????????????????????????????????????????????????????????????????????????????????????????????????????????????????????????????????????????????????????????????????????????????????????????????????????????????????????????????????????????????????????????????????????????????????????????????????????????????????????????????????????????????????????????????????????????????????????????????????????????????????????????????????????????????????????????????????????????????????????????????????????????????????????AVIVDRFHELLRFVPHISQFLRRQGVNFKIYVINQVDSLRFNRASLINVGFLLSRHDSDYIAMHDVDLLPLNDKLSYKFPTDGPFHVASPDLHPKYHYKTFVGGILLLTREHFELVDGLSNKYWGWGLEDDEFYVRLKEAGLTIKRPQGINTGPDNTFLHIHDRNHRKRDTAKILNQKQETRKRDRETGLSTVQFEIDSEHELKVDGYVCKVINVKLICDYKLTPWCDFKN???????????????????????????????????????????????????????????????????????????????????????????????????????????????????????????????????????????????????????????????????????????????????????????????????????????????????????????????????????????????????????????????????????????????????????????????????????????????????????????????????????????????????????????????????????????????????????????????????????????????????????????????????????????????????????????????????????????????????????????????????????????????????????????????????????????????????????????????????????????????????????????????????????????????????????????????????????????????????????????????????????????????????????????????????????????????????????????????????????????????????????????????????????????????????????????????????????????????????????????????????????????????????????????????????MDPESFLEMANAVTKLKMYPWFEVAHCIISCLYIREDLGQGAHAFSRKHPFALWVSCMTSIFAGGILANLLLGEPILGVLKNNNSILLATGVWYLIFYSPLDLCYKLCKFFPVKLVVAAMKEVTRCKKVHDGVVHAAKIYPNGYLIMVIVGTVKGNGAAFLKILERILRGSWTPNAIEFMVPTFPTKASILASVIFIVEKKTELITAPHAIVYLGIVICFIYFKLSSMLLAISDPFLPFENVFCALFMGGLWDALANMG-----KKKDMDQENKLIRRRNVSDDFDSKETRLTLMEEVLLLGLKDKEGYTSFWNDCISTGLRGCILVELALRGRIELEKAGMRRRSLLLRKVLLKNGHPTGDVLLDEALKHIKDTQPPETLQSWIDYLSGETWNPLKLKYQLKNVRERLAKNLVEKGVLTTEKQNFLLFDMTTHPLVDSASKNKLIKKVQDAVLGKWVNDPHRMNKRVLALILLAHASDVLENAFAPLSDDDYEVAMKRVRELLDLDMEAESLKPGTNETMWGVFAAFVK??????????????????????????????????????????????????????????????????????????????????????????????????????????????????????????????????????????????????????????????????????????????????????????????????????????????????????LTEWFTPVLKESKFRETGMLTPEEFVIAGDHLVHHCPTWVWCAG-DKDYLPSDKQYLSTRRVPCHKRCREMEEKIIDPE-GGWVDTHTQDDDDEEPEDMDDFIDEVDPTINIVATRTYDLNITYDNYYRTPRLWLSGYNENQQPLTIEEMYQDISQDHAKKTVTMESHPHIPGPHMASVHPCRHAEVMKKIIETMEENGRLQVHQYLIVFLKFVQAVIPTIEYDYTQNFSM??????????????????????????????????????????????????????????????????????????????????????????????????????????????????????????????????????????????????????????????????????????????????????????????????????????????????????????????????????????????????????????????????????????????????????????????????????????????????????????????????????????????????????????????????????????????????????????????????????????????????????????????????????????????????????????????????????????????????????????????????????????????????????????????????????????????????????????????????????????????????????????????????????????????????????????????????????????????????????????????????????????????????????????????????????????????????????????????????????????????????????????????????????????????????????????????????????????????????????????????????????????????????????????????????????????????????????????????????????????????????????????????SILVVMHYKYQDRSKRDINNAINHYKNLIEKFTFANGTTRDLVCLDGTIPVRYRYNIPVCIYLLDNYPYSAPMCYVRPTHDMTIKQSKHVDASGRIYLPYLSDWKDTT-SDLLGVIQVMIIVFGELPPVYSK-PPPYP--GTGTITEEHIRASLLTAVEDKLKSRLREQKQAEIDVLKRTSDELSKGKQRIEDLISKMEKDVSVQEDKERQLTDLNIDESFGPTMPLYKQLLDAFAEENAIVDAIYYLGEGLRKGSIDLEVFLKHTRELSRRQFFLRALMQRCREKAGLT???????????????????????????????????????????????????????????????????????????????????????????????????????????????????????????????????????????????????????????????????????????????????????????????????????????????????????????????????????????????????????????????????????????????????????????????????????????????????????????????????????????????????????????????????????????????????????????????????????????????????????????????????????????????????????????????????????????????????????????????????????????????????????????????????????????????????????????????????????????????????????????????????????????????????????????????????????????????????????????????????????????????????????????????????????????????????????-------DPAIRVWVFLPIVVITFFVGILRHYVSILISSQKKIELQQVQDSQALIRARLLRENGKYIPKQSFLMRRHFFNNDENGFFKIQQKRPSTQPNPMTDPSMMTDMLKGNVTNVLPMILIGGWINWTFSGFVTTRVPFPLTIRFKPMLQRGIELMSLNASWVSSASWYFLNVFGLRSIYALVLGENNAADQTRAMQEQMSGAAQAVPADPKQAFKAEWEALEIWDHQWALRGVDM----------AGRALQIQRRREKEREDLEIRKKKIEEETRIGKIDNKFATHFDAIEQQLKSSTIGLVTLEEMRAKQEDVVKEREKQLAQKQSEEQRRAEEKRKLEKERQKRQIATLSFKFDDLKITKNPEVDTSFLPDREREEEEKRLREQLRQEWQEKQERIKNEEVDITFSYWDGSGHRRSVRMKKRNTIYDFLLKCLELLKKEFHELRAVTADQLMYVKEDLIIPHHYSFYDFIVTKARGKSGPLFSFDVHDDIRLLHDATVEKDESHAGKVLLRSWYERNKHIFPASRWEPYDPTKNYSKYTISDKGKKLRPLPEYVLRAMKRRQRKRKKALLTRVKTRK-RKLLKAEYINRARKYEAEYERQKKAIIQNHRMARKHDNFYVPPEPKLALVIRIRGINGVPPKPRKVLQLFRLRQINNATFVKLNKATLNMLRIAEPYIAWGYPSLKTVRRLIYKRGFLKVNGRRVPLVTNETIQRHLGRQKIICLEDIVHEIFTVGPSFKRVNRFLWHFKLSNPRGGWRNKTRHYVEG----------------VITRTTAEQLWKGVTSVSNAGKKRGRGRGAGRKAIKDLNRGQTIGVGRINMLFPGLNAPIIRGRETLSQRSLPPDPEFDKKLIEIREKQSAFRVREMHDLDRGFSGAFMPGRWIGPPDPVNGEPFEGFDTKALKVKQHNIMCP-LGRRRVCDVVVAVGNKNGVAGFSEVTGKDQRQSLRKARSQAGQRLVHIPLFEGTVIHDFYSKYHCTELFVYKRPPNFGVEGHKVIRALCEVIGIKDLTVVTEGEPRNVLAMTKAFFLGLIKQKTFQEMADEKRLHVVEFDYDKDNYPVIVASPSDVRQKHEIGSEEVLDFQMYLHNGHVLDNPPPRPPPYIGTHGWEFFIKMQSTYKARARSRVQLIAQYGELKSHLNVWEAELRQKHRLSDEVGEWKNKQRLLIVAARGITARDRHLLLNLAESMPHSKREPKVE--KNDLNEICEMRNCNKCIYFETMKKRDTYMWISSVPNGPSIRFLVENIHTMEELRMTGNCLKASRPILSFDQHFENSPLMKEVFTQTFGTPQFHPKSQPFFDHVFSFSWLDNRVWFRNYQIVDEEKGTLAEIGPRFVLNPIKILEGSFRGEVLWSNPHYVTP-----------------------------MAVGKNKG-SQKGKKGGKKKVADPFARKEWYDVKAPATFSVRQVGKTLVNRTQGTRIASDSLKGRVFESSLADLNNDEIAYRKFRLIAEEVQGRIILTNFYGMDLTTDKLRSMVKKWQTLIEAVVDVRTTDNYLLRLFCIGFTRKSPNQVKKTCYAQTTQVRAIRKKMTDIILREVGRSDLREVVTKLIPDAIGKDIEKACQSIYPMHDVMIRKVKVLKRPKFDLGKLLEMHSDGKGKAAAGTKKVDRPDGYEPPV----????????????????????????????????????????????????????????????????????????????????????????????????????????????????????????????????????????????????????????????????????????????????????????????????????????????????????????????????????MKVLRHEEFKEGCQAACNGPYDGQWSKTMIGYGAEDKHFVMELTYNYGIGSYKKGDDFSSITIADSEILKRV----------NGQTVLFSPDQYQFNIAGSEQKNSSDPVQKVTLASSNLERTIEYWNEMLQMPIVERHEKSVQFAYAEDQARLEFINGGKEIDHGKAYGRIAFSCPTSHLKPIEAKMKSSNQTILTPYTELDTPGKATVAVVILADPDGYEICFVGDEAFSALSQVDPKADDLLNKAIEEDKSDDWYAKKNKQKPAA????????????????????????????????????????????????????????????????????????????????????????????????????????????????????????????????????????????????????????????????????????????????????????????????????????????????????????????????????????????????????????????????????????????????????TLIPMVVEQTGRGERAYDIYSRLLKERIICLMGPINDDVSSLVVAQLLFLQSESSKKPIHMYINSPGGVVTAGLGIYDTMQYILPPVATWCVGQAASMASLLLAAGAPGMRHSLPHSRIMLHQPLGSASGQATDIRIHAEEILFIKSLINGLYAKHTKQPVEVIENALERDHFMRPDQAKDFGIIDQVLEHPPMRLDKPTGSWLLLLPFWSIGLATPAGHLPDLALFTAGAVLMRGAGCTINDMWDKDFDRHVERTKNRPLAAGHLTMQDAWFFLGGQGLALMVLLQFDWNSILLGSGSLLLVSTYPLFKRFTYWPQLILGMTFNWGALLGYSVTVLPLYTAGIFWTLIYDTIYAHQDKSDDLMIGLKSTAIKFGDSTKTWLSAFSVMVSNLQSWPYFLALSLDINTLGSILGIFGSAFFS?????????????????????????????????????????????????????????????????????????????????????????????????????????????????????????????????????????????????????????????????????????????????????????????????????????????????????????????????????????????????????????????????????????????VQILEALSDNYMYLLIHREACVVDPVEPGKVVDAINVALKAVLTTHHHWDHAGGNQELAVYGGDDRVQALTQEGQ-QINVGSMITCMKTPCHTSGHICYFVVFTGDTLFQAGCGRFFEGTGEQMYQALLGSLPSHTLVYCGHEYTVNNLKYAKHVEPNNAVGERMAWAKGQPTVPSTIAEEKTFNPFMRVSVKKHTDPIEVMTFLRKEKDHFRMNRLFGRGKPKEPPPNLTDVIANVDSRAESVDKKIARLDQDLVKYKDQMKKMRDGPAKNAIKQKALRVLKQRKMYETQRENLLQQSFNMEQTNFATQMLKDTHTTVAAMRTGVKEMKQAYKKVNIESIEDLQDELEDMLDQANEVQDVLGRSYGCPDVDEEELEAELEALGDELAADDTSYLD????????????????????????????????????????????????????????????????????????????????????????????????????????????????????????????????????????????????????????????????????????????????????????????????????????????????????????????????????????????????????????????????????????????????????????????????????????????????????????????????????????????????????????????????????????????????????????????????????????????????????????????????????????????????????????????????????????????????????????????????????????????????????????????????????????????????????????????????????????????????????????????????????????????????????????????????????????????????????????????????????????????????????????????????????????????????????????????????????????????????????????????????????????????????????????????????????????????????????????????????????????????????????????????????????????????????????????????????????AYYITIDAHDENCFFDKVTTGTKMGLTFEVIEGGFLDIDVKITGPDQKVIHEEVRASSGKYTFAAHMDGVYTYCFGNKMSTMTPKVVMFSMDVGDAPAPDAAHDGDANHNKLEEMIKELSHALTAVKHEQEYMAVRDRIHRSINESTNSRVVLWAFFEALVLVAMTLGQVYYLKRFFEVRRVV?????????????????????????????????????????????????????????????????????????????????????????????????????????????????????????????????????????????????????????????????????????????????????????????????????????????????????AMNKTDAVLMVTRFCTVLFTFMYLIPSPYACYQKALIANAATSALRLHQRLPRFQMTREFIALVLLEDSCHYLLYSIIF-LPIT-LSLLPIALFAFLHLVTLFDKAGSIVE---NQRSILQTISLCEIFLMPCIVLGIFSGVSLFAPFLYYRFVYLRYASRRNPYTRQMFHELRIATESLIYPSCPQIIRGISLMEGLAP????????????????????????????????????????????????????????????????????????????????????????????????????????????????????????????????????????????????????????????????????????????????????????????????????????????????????????????????????????????????????????????????????????????????????????????????????????????????????????????????????????????????????????????????????????????????????????????????????????????????????????????????????????????????????????????????????????????????????????????????????????????????????????????????????????????????????????????????????????????????????????????????????????????????????????????????????????????????????????????????????????????????????????????????????????????????????????????????????????????????????????????????????????????????????????????????????????????????????????????????????SEDGEDLFGPELDRYDQAEAEMRRRDRG-MRRGLEEIADLENTRGMSIKEWIAQGPRKEIHHRFRSFLLSFKERIRQMEENKSSFEVNYHLAAQEYVLAYFLAEAPQQMLEIFNEAAKEVVLSMYKNYDRIAKEIFVRITDLPLIEDIRALRITHLNGLIRTHGVVTSTTPVLPQLSLIKYDCQKCGFLLGPFVQHQEVKPGSCPECQSLGPFALNMEETIYQNYQRIYVQESPGQVNAGRIPRSKEAILLGDLCDSCRPGDEIELIGVYTNTYDGSLNIAHGFPVFSTVIMANHIRKKDTDEDIKQITKLSKDIIIDRIVASIAPSIYGHKNIKRAIACALFGGESKDPGQKHRVRGDINVLLCGDPGTAKSQFMKYISKIAPRAIFTTGQGASAVGLTAFVSPVTKEWTLEAGALVLADKGICLIDEFDKMSDQDRTSIHEAMEQQSISISKAGIVATLQARCAVIAAANPIGGRYDTFNDNVDLTEPIISRFDIIQVVRDEKLARFVVRSHMKHHPIPQDLLRKYITFAKQRVHPKLDREKIAKLYAELRRESLRTGSIPITVRHVESIIRCSEALAKMQLHDYVDVNIAIRIILESFITQKFSVMK??????????????????????????????????????????????????????????????????????????????????????????????????????????????????????????????????????????????????????????????????????????????????????????????????????????????????????????????????????????????????????????????????????????????????????????????????????????????????????????????????????????????????????????????????????????????????????????????????????????????????????????????????????????????????????????????????????????????????????????????????????????????????????????????????????????????????????????????????????????????????????????????????????????????????????????????????????????????????????????????????????????????????????????????????????????????????????????????????????????????????????????????????????????????????????????????????????????????????????????????????????????????????????????????????????????????????????????????????????????????????????????????????????????????????????????????????????????SRTDDETLAREEEAIKLDGLSVKQMKEIRDRAEKYAFQAEVSRMMKLIINSLYRNKEIFLRELISNASDALDKIRVYSLTDRSALDTKSELEIKIKADKDSHMLHITDSGIGMTKEELVKNLGTIAKSGTAEFLQKVSEASELQNLNDLIGQFGVGFYSAFLVADRVIVTSKSNDDPVQHIWESDSAQFSIVEDPRGDTLKRGTQVSLVLKEEARDFLEVDTLRNLIQKYSQFINFPIYLWSSKTEIVDEPIEDE----EEEKKDEVDEEAKVEEAKEEKKPKTKRVEKTVWDWELINTAKPIWQRKPDDITDDEYNEFYRALTKDHQDPLTRIHFTAEGEVTFKSLLYVPTLQPSESFNRYGTRQDHIKLYVRRVFITDDFQEMMPSYLSFIRGVVDSDDLPLNVSRETLQQHKLLKVIRKKLVRKALDMIKKIPADKYDQFWKEYSTNMKLGIIEDATNRQRLAKLLRFHTSSLSPDKWTSLNDYVKRMKPKQEYIYYVAGASYDEVSRSPFVERLLKKGYEVLFLTDAVDEYSISNLPEFEGKKFANVAKEGLKLDADEKAKEKQEALEREFEPLTKWLETKALKSRVTKVKVSHRLYESPAVLVASQFGWTGNMERLAKSNAHSKTQDATRDYYLSQKKILEINPRHPIVRELLRRINDDEDDMTARQMSLLMFETATLRSGYMLEDTEDFGRRVEKLLRKSLGVDE--------GKKRKDAKLSKNELMMLTVGEVIQELIKSHHEGKDVNLTKLKGQIASKYGLESQPRLVDIIAAVPSDYRKILLPKLRAKPIRTASGIAVVAVMCKPHRCPHINMTGNICVYCPGGPDSDFEYSTQSYTGYEPTSMRAIRARYDPFLQTKSRLDQLRQLGHDVDKVEFIVMGGTFLALPEDYRDFFIRNLHDALSGHHSQSVAEAVRHSEKSRVKCIGITIETRPDYCLRRHLNDMLLYGCTRIEIGVQSVYEDVARDTNRGHTVKAVQESFQLAKDCGFKVVSHMMPNLPNVDLERDLAQFVEYFENPAFRSDGLKIYPTLVIRGTGLYELWKTGRYRSYPPSVLVDLLAQILALVPPWTRVYRVQRDIPMPLVTSGVEHGNIRELALARMADYGTKCRDVRTREVGIQEIHHKIRPYEVELVRRDYVANGGWETFLSYEDVEQDILIGLLRLRQCSVDTFRPELKGGVSIVREHVYGSVVPVSSKDPTKFQHQGFGTLLMAEAERIAIEEHGSWKISVISGVGTRNYYRKLGYELDGPYMSK

'Osperalycus_tenerphagus' --------------------------------------------------------------------------------------------------------------------------------------------------------------------------F-IFNKAREQVMKMTKGLYPAPLKILDVVKTGLEKRGFAELSTLHSKALIGLYHGQVLCKKNRFGKPTKTIAVLGAGLMGAGICQVSIDKGLDTIMKDGLARGETQIKKRRLDRDLYMSRLYSQTDYSDMVIEAVFEDLNIKHKVIQEVEQDDCIFASNTSALPISKIAEASKRPDKVIGMHYFSPVDKMMLLEVITTDKTSPETAAAAIQVGLKQGKVVITVKDGPGFYTTRILAPVLSEAIRLLQEGVKPKELDNLRSFGFPVGAATLIDEVGIDVASHVAEKVFVERKEMVAGFLGRKSGKGFYVYRNMNDILKRYTQEELQMRLAVRFVNEAVLCLQEGILANPLEGDIGAVFGLGFPPFLGGPFRYLDNHGADKIVAMHKFAEFKPCQLLLDHA-KDTSKKFHMQAPIILLKEGTENTQGRNQVVSNINACQAIAEAVRTTLGPRGMDKLIVDSKGSVTISNDGATILKQLEVVHPAAKTLVDIARSQDAEVGDGTTTVAILAAEFLKQCKPFVEEGLHPQIIIKSFRKGCQLAISKINEIAVTI-QDKRRAILEKCAATTLSSKMIAQQKEYFAKMVVDAVMLLDELLPLDMIGIKKVQGGALEESKLIAGVAFKKTFSYAGFEMQPKKYQNLKIALLNVELELKAERDNAEVRVDSVKEYQNIVDAEWNILYDKLKKIHESGAKVVLSKLPIGDVATQYFADRDMFCAGRVPEDDLKRTMRACGGSILTTVYDLKDENLGTCELFEEMQMGGERYNLFTGCPNTKTVTIILRGGADQFIEETERSLHDAIMIVRRALKNDAVVAGGGAIEMELSKYLRDYSRTIAGKEQLLIGVMAKAFEVIPRQLCDNAGFDATNVLNKLRQKHASGGVWYGVDIFNEDIADNFEHCVWEPAVVKSNAIISATEAACLILSVDETIKNPKSSNEMTAARAMVNEMNEGAGRNQHSLAVTRDYISQPRLTYKTVSGVNGPLVILDDVKFPKFAEIVNLVLSDGTKRTGQVLEVSGSKAVVQVFEGTSGIDAKHTVCEFTGDILRTPVSEDMLGRVFNGSGKPIDRGPPVLAEDYLDIMGQPINPESRTYPEEMIQTGISAIDVMNSIARGQKIPIFSAAGLPHNEIAAQICRQGGLVKAPGKSVIDENFAIVFAAMGVNMETARFFKQDFEENGSMENVCLFLNLANDPTIERIITPRLALTTAEFLAYQCNRHVLVILTDMSSYAEALREVSAAREEVPGRRGFPGYMYTDLATIYERAGRVEGRDGSITQIPILTMPNDDITHPIPDLTGYITEGQIYVDRQLHNRQIYPPINVLPSLSRLMKSAIGEGMTRKDHADVSNQLYACYAIGKDVQAMKAVVGEEALTSEDLLYLEFLGKFEKNFISQGSYENRTVFESLDIGWQLLRIFPKEMLKRIPQNLLAEFYPRGGQATGLFNDPNGFYLLRENAVIEAEHLVKEALSRQRKMVQIFDELSNALCKVADLAEFIRIGHPSERFQMCAEQASMAISAEVERLNTHRPLYDELRRVTLES-DNLPTTLDSHVAKLFLFDFEQSGIHLDSADRKVVVQLNEHILHVGSYFMNNTNQPRTVAKSKLPEEIAIVTGLFADFDNELLREAAYRIYLHPDDHQDQLLSQLLRSRLKLAKICGFESYAHRAVKHSIAENPEMIQFLDILNERIRPLADRDYKEMLMLRPWDVPYFTPYFSLGACMDGLNTIFGHLYGVRLQVAGETWHKDVVKLSVVDSSELLGYIYCDLFERATKPHQDCHFTIQGGCLGDGTYQVPIVVLMLSLPPSLLTPHMVDNLFHEMGHAMHSMLARTPYQHITGTRCSTDLAEVPSILMEFFASDPRVVSKFAKHYITGEPIPDEWLATWIQSKRVFTASDTQLQVFYAALDQAYHSEKHVSTTDVLAAIQNRYYGLPHIPNTAWQLRFAHLVGYGAKYYSYLVSRAVAAAIWKKLFAADPLSACAGRFKDEVLAHGGGKPARDIAEGVLIASSIVDDV????????????????????????????????????????????????????????????????????????????????????????????????????????????????????????????????????????????????????????????????????????????????????????????????????????????????????????????????????????????????????????????????????????????????????????????????????????????????????????????????????????????????????????????????????????????????????????????????????????????????????????????????????????????????????????????????????????????????????????????????????????????????????????????????????????????????????????????????????????????????????????????????????????????????????MVLADLGRKITSALRSLGNATIINKDVLDSLLKEICTALLEADVNIRLVKQLRENVRQVIDFDEMAAGLNKRRMIQMVVFKELVKLVDPGVKAWQPTKGRCNVVMFVGLQGSGKTTTCTKMAYYYVRKGWKCALVCADTFRAGAFDQLKQNATKARIPFYGSYTESDPVVIASEGVDKFMNEGFEIIIVDTSGRHKQESSLFEEMLAISTAVQPNLIIYVMDASIGQACEAQARAFKSKVDVGAVIVTKLDGHAKGGGALSAVAATQSPIIFIGTGEHIDDFEQFKVKPFVSKLLGMGDIEGLIDKVNELKLEENEELIEKLKHGEFTLRDMYEQFTNIMKMGPFNQLLSMIPGFGADLMKGASEAESMSRLKRLMTIMDSMSDSELDSREGAKLFTKQPPRIARVARGSGVTQKEVQELLTQYTKFSAVVKKMGGIKGLFKGGDMAKNVNPTQMAKLNQQMAKMMDPRLLQQMGGMSEVLGIYKKMHGF------------------------------------------------LLFDYSKNLVNDDVLKLLFDLARSRKVEEMRDKMFAGEKINFTEGRAVMHVALRNKSNAVTLVDGKDVMPDVNRVLDQMRRFSVALISGSWTGYTGKKITDVVNIGIGGSDLGPLMVAEALKAYQIGPNVHFVSNIDGTHLTTVLRRLNPETTLFIIASKTFTTQETITNAVTARDWFLKTAGGKSAVAKHFVALSTNTEKVKEFGIAESSIFEFWDWVGGRYSLWSAIGLSIACHIGFDNFRQLLDGAHWIDNHFKTTTLEKNIPVIMALLGIWYIDFFGAETLAILPYDQYMHRFAAYFQQGDMESNGKYVTRNGEPVNYSTGPIVWGEPGTNGQHAFYQLIHQGTKLIPCDFIAPVKTLNPVRDGVHHKILLANFLAQTEALMKGKTRGEAEAELKKKGMKDEEIAKILPHKVFEGNRPTNSIVIDKLRPFNLGALIALYEHKIFVQGVIWDINSYDQWGVELGKELAKAIEPELDSAETITSHDSSTNLLINHIRRVG-------------------------------------MDKMIQSANGDVTITNDGATILKQMQVLHPAAKMLVELSKAQDVDAGDGTTSVVVIAGSLLEAASKLLAKGIHPTTISDAFQNAAKTSTKILSSLAIPVHLTDIESLVRVAATSLNSKVVSQYSSLLAPIAVNAVLRVKDNDNVD-------??????????SLIEGLLLPLRFANDFGAKKVEKAKIGLIQFCISPPKTDMDNQVIISDYTQMDRVLKEERNYILNIIKQIKKTGCNVLLIQKSILRDALNDLALHFLGKTKIAVIKDIEREDIEFICKSLGCRPIASLDHFTSENLVSADLVEEIPGAKFVKITGISAGSHNKTVNILVRGSNRMIIDEAERSLHDALCVIRCLVKNAALVPGGGAPEIELSLRLSELSRSMTGLEAVCYRAFAEALEVIPFTLAENAGLNPIGTVTELRQRHAAGEKNTGINVRKGSVTDILEENVVQPLLVTSSAITLAAETTRSILKIDDVINTVRMSPSNVVYFWDPEVGNFHYGPGHPMKPHRLAVTHSLVLNYGLYKKMNVYKPYRASFHDMCRFHSEDYIDFLKRVTPSNVANFTKHLSQFNVGDDCPVFEGLYDFCSRYCGASLQGAVQLNNHCCDIAINWSGGLHHAKKFEASGFCYVNDIVIAILELLKYHPRVLYIDIDIHHGDGVQEAFYLTDRVMTVSFHKFGNYFFPGTGDMYETGSEAGRYYSVNVPLKEGIDDHTYSTVFKPVVSDVIQFYQPTAIVLQCGADSLAADRLGCFNLSIKGHGECVKFVKNLNLPLLVLGGGGYTLRNVSRCWTYETSLLLEEQISNEIPYSEYFEYFAPDFSLLLDKPCGETTHNANSRVYLDSIVKYVHENLKCVAHSPSVQMHQTPSDWLDLSENPIHLLSERESENEFYDREEGHRNS-------MCADEEDYDLEYSEDSNSEPDVDLENQYYNSKSLKADTPLVALASFQKVLDLE-GDKGDWGFKALKQMIKINFKLGNYPEMMARYKQLLTYIKNAVTRNYSEKSINSILDYISTSKQMELLQEFYETTLEALKDAKNDRLWFKTNTKLGKLYFDRGEFNKLAKILKQLHQSCQNDDGSDDLKKGTQLLEIYALEIQMYTAQKNNKKLKKLYEQSLHIKSAIPHPLIMGVIRECGGKMHLREGEYEKAHTDFFEAFKNYDESGSPRRTTCLKYLVLANMLMKSGINPFDSQEAKPYKNDPEILAMTNLVSAYQNNDINEFEKILKNNRRTIMDDPFIKEHIEDLLRNIRTQVLIKLIKPYTRIHIPFISKELNIDVSEVENLLVSCILDNTIQGRIDQVNSVLELNKGSQSAARYNALDKWTNQLSSLHQSIVNKMIRQMSLNPVQILKGDADEEKGELARLSSFVGAIAVGDLVKSTLGPKGMDKILLCQGRGEGKVEVTNDGATILRSIGVDNAAAKVLVDISKTQDDEVGDGTTSVAVLAAELLKEAEQLIAMKLHPQTIIAGWRKATQVARDALEAVAIDVSDDEEVFRKHLINISKTTLGSKILSRHNDFFSKLAVDAVLRLKGSGNLDAIQVIKKLGGTLEDSFLDAGFLLDKAPGHGQPRRVEKARILIANTPMDTDKIKVFGSRVRVDSVAKVAEMELAEKEKMRDKVNLILKHNINVFLNRQLIYNYPEQLFADAGVMAVEHADFDGIERLALVTGAEIVSTFGNPDKVKLGTCDLIEEVMIGEDRLLKFSGVPLGEACTVVLRGATQQILDEAERSLHDALCVLSQTVKEPRIIYGGGAAEMLMANAVGDLAAKTPGKESFAMESFAKALRQLPTIIADNGGHDSAQLISELRAAHAKGQHSYGLNMDVGLVDDMAKLNVIEAYVVKRQVLISAAEAAEMILRVDNIIKAAPRKRNPDHLFMAAEIRPPGRLQLVSKLEGHQDVVNMAIILRDEEGLISISDDKTVRIWLKRDVGSYWPSVCHIMPSAATAMDFNHETRSLFVGMENGSISQFSVSDDFNRITHDRNYLAHQARVTAVIFSLVTEWVLSVGRDKYFQWHCSETGRRLGGFGCSSWCTALQFDVQSKHVFIGDYSGQVTMLKIEETGYKPVTTLKGHSGCIRRLEWDADRSMLFSASSDQVIICWDIGGKRGTAYELQGHHNKVTSLCYAAASKLLSGGEDCAIIAWDMKAKRKETPEWAESDFCQRCSRPFFWNIKAMVDQKIIGLRQHHCRKCGKAVCDACSTNRTTIPVIGYEFKVRVCDECHSVITDADRVSLATFHESKHSIAHMDLDESRCQLLTAGADRVIKIWDVTRLLSKKAQKKAAKQVEKAAKKAEKDAKQASSLVNSNQIMMDNRYGKMAMVQSSQKTDRVLVSVMSLTRDIVDKKVWLRGRLHTSRSKGKQTFFVVRQQQFSVQCLVSVSDIVSKAMVKFAATITKESIIDVEGVVKSVPMKIESCSQQDVEIHVEQLFVVSNSEPKLPLQIEDAARPETEGDEEGGLAIR--------------------AIFRLEAGVCKLFRETLEKRGFVEIHTPKIISAASEGGANVFEVSYFKGSAYLAQSPQLYKQMAIAADFGKVYTIGAVFRAEDSNTHRHLTEFVGLDLEMAFNYHYHEVLDVIGEMFVDIFKGLQSMYATEIATVNRQYPAEPFEWIEPSLRLEYKEGVAMLGAAGVEMGDEEDLSTANEKLLGKLVKQKYGTDFYILDKYPMAVRPFYTMPSPDDPKYSNSYDMFMRGEEIISGAQRIHDSELLTERAKAHGIILEKIESYIDAFRYGCPPHAGGGIGLERVTMLFLGLDNIRKTSLFPRDPRRLTPMLEEGIHIQNLRPLELDRVRVVYPSECRMRGTTYKGKLVLNFWSLNGNMQDVFEEVVGEIPIMVKSDKCNLAKMTPKQLIEKREDAEELGGYFIVNGNEKLIRMLTAQRRNYPLALSRKSWKEAGQFFSEYGVSMRCVRNDGANMVLHYLTNGTAKLRFFYQAQPIFLPLVMVLKALCDVSDQYIYNELIRGKEDDTFFKSCSANMLRLVQEGLFTQQVKKYIGERFRWYTDEEVTHFLSKESIAVHLNSNVDKFNLLVFMTRKLFAVAKGENALENADNPMFHEVYMSGHIYYTLFLERLSMFLSSVKSVIDRTIKKSLEIVRPLEYLLSTGNLLSKSGLGLLQRSGLSVMAEKINFWRFLSHFKSVHRGAFFAQMKTTACRKLYPEAWGFLCPVHTPDGAPCGLLNHFSEMCVLTVSLDGKLLGYLLRVMKVKCKIPQTLEIGFVQKTGKATQYPGVFLFSTPARMMRPVFNLTQSVELIGTFEQVYLDICVVGEEATTHQELRENGILSVLASQIPYPDFNQSPRNMYSCQMGKQTMGTPSHTLRFRCDTKMYDITPQSPLVRPTMHDHYHLDDYPLGTNAVVAVISYTGYDMEDAIILNKAAVERGFKHGVYKTQVVDLKVDVDGLPMIGSYDDPVCSYITAKVEKFKSTEVAYIYDVKLLGSLQKIAIMYWIRRPIIGDKFANRHGQKGICSIKWPQENMPFTESGLTPDIIFNPHGYPSRMTIGMMIESMAGKSAAIHGTIHDATPFQFSEDNVASDYYGKLLEAAGFNYYGTERMYSGVDGRELTADIFMGIVYYIRLRHMVGDKYQVRSTGPIDTLTHQPVKGRKRCGGIRLGEMERDSLLAHGTSFLLQDRLFHCSDKSSCVRCGSIISVTLPYVLRYLVSE-LASVNIKVMRETKYDIHKIPRNDPKLHPFEVAREYQRAMNAVKLDRVFAKPFLGSLGHSDVVNCLMKHQHSLSILVSGSCDGQVKIWNIAERKCLRTIQAHNSIVRSLCDDDVDIDTIIAKMDHHFKPLLMTVGEKVDLWEERQEPLRSYW-GVDSVQVKFNPVESAASDRSIILYDIRKANPLRKVVLDMRSNAICWNPMEAFHFTVANENYDLYTFDMRKLRIPIQIHKDHVSAVIDLDYSPTGKEIVSGSYDKTIRIFRSREVYHTKRMQRLTSVVWSLDNKYIVSASDEMDIRLWKSDASEKLGLKRYQSKLKEKFGNHPEIKRIARHRHVPKHIYREQRIMLESRKKKAKQIVLSNREYIDLTDEIRAKIEGCSKLYLSIHWWLLYQDALSKHRAALDDLNEVVLLKPDFAAARQQRGSALLKQGRLDEAHIDYEYLLREDPANEEAMLESLKRDAVDLLTKLWDVKLREMRSMAYEKLGDLMNAISDLRATTKMRADNTEGYLKLSKLHYDLGEAEESLTTIRECLKLDPDHKLCYKKVKKLAKGMFDECLEKMVHLIKSRCHCLNKAGDGLTVCSEALRLSPVLCDRADILIQSEDLDNDYQLAHNIDKRAKEGIQRVQKLLKQSKKRDYYKILANKKEIMRAYRKLAKKWHPDSC-EGED--KEMAEKRFMDIAAAKEVLTDKVKRHKFDNGEDPLDHDEQFTFRFHYMLVLFETASGYAVFKLLDEKKLQKTDNLFKAFQSAEKAGKIISLKHFEKFHDTTEALSSSTALIEGKMSKKLKKMLKKVVVKGDGDSLAVADAKLGNTIKEKLSINCVTSTAVQELMSCIRSQVENLIPEWSPDDESAMQLGLSHGIGRYKLKFSPDKVDTMIIQAVSLLDDLDKELNNYIMRCKEWYGWHFPELGKVVTDNTAYVKTILQLGMRSNATNADLTDILPEEVETKVKQLAEVSMGTDIADEDIFNISHLCENILELQEYRSQLFEYLKNRMMAIAPNLTILVGELVGARLISHAGSLISLAKHPSSTVQILGAEKALFRALKTKHDTPKYGLIYHAQLIGQSNPKIKGKMSRMLAAKASLACRVDALGEESSVELGTEHRAKLEARLKFLEEGGMRRISGYVWPKEKSDIRKRVVISVALLIFAKVLNSYVPFIFKYAVDYLNLHTGLVVGYGIARAGSSLFNELRNAVFAKVAHDSIRRVARNVFLHLHNLDLNFHLNRQTGALSKAIDRGSRGINFVLSALVFNVVPTIFEVGLVSSILYYKCGQYAAVTLGCIGTLVVTQWRTKFRVEMNKAENDAGAKVVDSLINYETVKYFNNEQYELKKFEHASLKTATSLALLNFGQSVIFSASIMVLAANMTVGDLVMVNALLFQLSLPLNFLGSVYREVRQSLIDMQTMFSLMNIKNKPIWVSPEVRFEDVSFQPILDGLSFEVSAGQKIAIIGGSGSGKSTIVGLLYRFFDPLQGRICVAGKDIKNVSLRKAIAIVPQDPVLFHNTILYNLHYGNFTEDVYQASRMAELHDKILRWYETQVGERGLKLSGGEKQRVAIARAILKNSPILVFDEATSSLDSITEYKIMTALRAAENRTSICIAHRLSTVVDADQILVLDNGRIVEKGHLSLVSSLYASLWYKQHELNVPETRVTELDNGLRVATEDSGIQTCTVGVWIDAGSRWETPKTNGVAHFLEHMAFKGTAKRSQTQLELEVENMGAHLNAYTSREQTVYYAKCLSKDLPKAVEILSDITQNSKFGEAEIERERGVILREMQEVETNLQEVVFDHLHSVAYQKTPLGMTILGPTENIKSIKRDDLVTYIKTHYKAPRIVLAGAGGIDHDELVKLANEHFGKVPHTYEDV--NPCRYTGSEIRVRDDSMPLAHVAIAVEGAGWENPDNIPLMVANTIIGSWDRSHGGG--???????????-????-------------------------------------------------------------------------------------------------------------------------------------????KHSVHTLVFRSLKRTHDMFVANHGDLPPIDEHVEQHTFNIKARDQFGPVMHLVQQNSKLTTKNAVVLSSGTAVIPRRAPTMPKPEWHPPWKLMRVISGHIGWVRCLAVEPGNMWFASGSNDRIIKIWDLASGKLKLSLTGHISGVRGLAVSARQPYLFSCGEDKTVKCWDLEYNKVIRHYHGHLSGVYTLAVHPTIDILITGGRDAVARVWDMRTKAQIHCLSGHTNTVATLQCQATEPQVLTGSHDTTVRLWDLVAGKTRVTLTHHKKSVRALVLHPKLNMFASGAPDNIKQWRCPDGNFIQNLSGHNAIVNCLAMNGDNVLVSGGDNGTMFFWDWRTGYNFQRHSAPPQPGSIESESGIFTMTFDQSSARLITGEADKTIKIYKEDENATEETHPINWKPDIVVGSSSDYRIDVNKQDYLEKLGINVKARNFLVYQGAVESIAMKNPREITSLFEEISHSMDKSEEELFIKKKGIAAEKKEAQGEEAKKYQDMKDELQVQLQLFKLNYLQKDKDKKKKKLAIDEEIKGKKNIEKQLREELEVNKKRPAYIKAKENKSHIEKKLDTAKKSLAAARRAQTNHREIEDLEKELAETIEKFIAEDVELREEQKQRYNELKKTAAEQSAKYLKDLDSLEREHKADSDRLEAEQRKKNEIESRIREQDENKKRIEKLEDLAELKDKETEIKAKERINTELESICELGDAKVDRHGEERRKKKTEIVDHLKKLYPGVYDRLLNLCKPIHKRYNMAVTKVMGKSMEAIVVDSEKTGRLCIQYLKDQMLEPETFLPIDYIEVKQVRERLRNIQHPKGVKLVYDVIKYDPPPIKRAILFATNNALVCETADDANIVAFDLGDGKRYDAVSLDGTFYQKCGFISGGSAELERRARRWDEKEIHKLKYQKEKLTEELKDQLKKTRKESDLMVIQSQIKGLETRLKYCDIEEIRKRMHSREAKINDIRDKMNTVEDRVFSDFCEELGVENIRQYEERQTKASQERERKLQLENDRNNIENRLAYERGYAANLGGVKDEEEHLEKAKQKEREEMRAIEVEMNKIEQLKNDRLSQRQDCDRVEDEIGEKKRNLSNVQKEVSAVQKAQMTTECKMESRKADRHAVFLHCKMECIDLPLLAGNVQKSYENDNIKPDFSKLEMQEDIKLMKFQAPNMRALERLDGVKERLKETDTELSNLRKNAKAAFEKVKRDRACFEAVAQRVDSIYKSLANNASAQAFLVPENPEEPYLEGINYNCVAPGKRFQPMSNLSGGEKTVAALALLFAIHSFKPAPFFVLDEIDAALDNTNIGKVARFIRDRTESAFQCI---------------------PGDCTISRVFTVDLSVPDLVLLLLKKAITECNVFEIQNLYENSFVKLTDTYFLIFYKE--LYYRHIYADHRFESYFNYCRLFNHILSANPVSLELPNQWLWDIIDEFIYQF--QSFSLFVWNVHSVLNVLHSLVDKSAINEQLKEFNSGRDPDLVSGDFGRQPLYKMLGYFSLIGLLRLHSLLGDYYQAVKVLENIDLNRQPLARVLACQMTTFYYVGFAYMMMRRYSDAIRTFANILVYLQRTRRTLQIEMIEKQTDQMYLLLAICLVLHPQRIDESVLSQMQEKYMLKLQRGDLKEFETCPKFLSPVQMKVFMDEVNQQLTIRSYLKLYTTMPISKLAGFLLLCFKHKMQNLVLDGEFRSGSDVDFYIDKDMIHIADTKVARRYGDYFLRQIHKFDELYRMGPPDAILGVTDAYKKDTNSKKINLGVGAYRDDNGKPFVLPSVVAAEEAIMAKKLDKEYLPISGNAEFCQAAARLAFGDTSRVIQEGLNATVQGISGTGSLTIGAFFLRDFFTGNKEVYMPTPTWGNHIPLFKKAGFVVKQYRYYDPKTCGFDFTGALQDLAKIPEKSIILLHACAHNPTGVDPKPEQWKEISKVVKNRNLFPFFDMAYQGFASGDIDRDAAALRMFIEDGHEVALAQSFAKNMGLYGERVGAFTMVGATKEETAKILSQLKIIIRPTYSNPPLHGARIAQLILTDASLRKQWLKDVKGMADRIISMRTKLRDSLQKEGSTRNWQHITDQIGMFCFTGINADQVDRLTKEFSVYLTKDGRISVAGVTSNNVEYLAHAMHQVTKHLSSSTKHLLHCLLFLAVIFFFEYMSEDLDNPDPFVTYGSLLACFLYSIRFLTLLSLPQCLCNFLGLTLYNAFPEKVTLKGTPLLAPFIAIRTVTRGDFPELVGKNVHRNIQTCLNVGLENFIVEVVTDKPINLQKHPRIREVVVPNSYRTKTGALFKARALQYCLEDENNILGDGDWIVHLDEETILTENAVCGILNFVYNGKHEFGQGLITYANEEIVNWVTTLADSFRVADDLGKLRFQFYMFHRPLFSWKGSYVVTQYKAERSVSYDHGLDGSVAEDCYFSMIAYKKGYTFDFIEGEMWEKSPFTIRDFLQQRKRWMQGIFLVVHSPKIPLENKFFLAMSLYAWMTVPLSTSNLIFASLYPLPSTAWCNFLSAFVGAVSIYMYIFGVMKSFSLIRLGLKKLMLCMFGAFLTIPLNIVIENVAVIWGFFGKKHKFYPWPGFEFTGILRPFPVTPRRDVPDTIQRPDYADHPEGRSACEEAIKGTTQIKVLNDDEIEAMRVACKLAREVLDVAYSAIDVGVTTDEIDRMVHEACIDRDCYPSPLNYYEFPKSCCTSVNEVICHGVPDLRPLQDGDIVNVDITVYHEGFHGDLNETFFVGNVSDESKKLVRVTWECLQKAIEIVKPGERYREIGNVIQKHAQQHGLSVVRTYCGHGIHRLFHTAPNVPHYAKNKAVGVMKPGHCFTIEPMIAEGTWRDVTWPDDWTAVTTDGKRSAQYEQTLLVTDTGCEILTRRRDRDGQPYFMDLYFVRRILKKRSQKRRVLLAQQLGAGSVDNLSTILGNVDERCADLHDDDDDDELCTYTDSSTFLKGTQSANPHNDYCQHFVDTGQRPQNFIRDVGLHDRFEEYPKLKELIRLKDELIGKTATPPVYLKCDLKQFDLRELKSKFDVILIEPPLEEYQRTQGVTNNEFWTWDDVMKLEIEEVAAQRSFVFLWCGSSDGLDLGRQCLRKWGFRRCEDICWIKTNIKNGHSKNLEPKAIFQRTKEHCLMGIKGTVRRSTDGDFIHANIDIDLIITEASEYGSIEKPEEIIHIIEHFCLGRRRLHVFGRDSTIRPGWLTAGPGLTNSNFNSDTYTGYFNIPNDYLTGCTDRIESLRPKSPPPKDKRVLIRVDFNVPMTDGKISNNQRIVAALDTIKYARDNGAKSVVLMSHLGRPDGQRVDKYTLRPVAEEVGKLLNCNITFLNDCVGPEVENACANPTKGTIILLENLRFHIEEEGKGVDASGKKVKADTKAVEAFRASLTKLGDVYVNDAFGTAHRAHSSMIGVDLPQRAAGCLMKKELMYFAKALDNPDRPFLAILGGAKVKDKIPLISNLLDIVDEMIIVGGMAFTFLKVAKNMEIGTSLFDEEGAKIVNELIEKAAKKNVKMHLPIDFVIGDKFDENATVGEATIESGINQGWMGLDCGPKSVVLFAEPIERAKVIVWNGPVGVFEWDNFAKGTKSVMDMIVKATQRGTTTIIGGGDTATCCAKWGTEDKVSHVSTGGGASLELLEGKELPGVAALTNAMRTFGDRPTAFQLEENGEYYYIGSEVGNYLRMFRGSLYKRYPSLWRRLVTVEERKKISALGLGPHTLATNITLLKASEVDEIFEGKDDKYKAVSISTEPSQPREPKPKRTNWMPTLPNSSHHLDAVPCSTAIARHRLSHKKVRTFPMLYDDLDPAGIHENASQSEVLVPIRLDMEIEGHKLRDTFTWNKNETTITPEQFAEVLCDDLDLPSASFVPAIAQSIRQQIEAFSTDNLLDDQTDQRVILKLNIHVGNISLVDQFEWDMSEKENSPELFALKLCSELGLGGEFVTAIAYSIRGQLSWHQRTYAFSEAPLPTVDVPFRNQSEADQWAPFLETLTDAEMEKKIRDQDRNTRRMRRLANFALMGQNASQLEKEIGFPQNEHYFGLVNFGNTCYCNSVIQALYFCRPFRDKVLEFKTKNKRSKETLLTCLADLFHNIANQKKKTGTFAPKKFIARLRKENEVFDNYMQQDAHEFLNYLLNTIADLLSAEKGWVHDIFQGTLTNETRCLNCESLSSKDEDFLDLSVDVDQNTSITHCLRDFSNTETLSSEHKYYCENCCSKQEAQKRMRIKKLPMILALHLKRFKYMESQNRHTKLSYRVVFPLELRLFNKTSNSEGDDKLYDLVAVVIHCGSGPNRGHYISIVKSCGFWLLFDDDLVDKIEASAIEEFYGLTSDTQKNSESGYILFYQSREMPDLDKWIEIARECKYLPENDLKQLCDIVCDILLEESNIQPVSTPVTVCGDIHGQFYDLEELFRNGGQVPDTNYIFLGDFVDRGYYSLETLIRLLTLKAKWPEKITLLRGNHESRQITQVYGFYDECQSKYGNANAWKNCCRVFDLLNIAAIIDDQVFCVHGGLSPEINTLDQVRTIERNQEIPYKGAFCDLVWSDPEEVDTWSMSPRGAGWLFGAKVTHEFMHINNLKLICRAHQLVQEGYKYMFDDRLVTVWSAPNYCYRCGNVAAVLAFNDVDNREAKIFSAVPDCDRVIPPRNTPYFLMKNVEGQSTADIDHLVIEDIKEHARQIEKSVNMKETRFVLRVLRSLVTTRKKLNARVLRKIISGFYTHSAEQRDALLAFVEPMDMDAHNVAKTSQLPLLPELDVYFNLLLLLYLIDLARYDSAVKCSDQLMAKVKSHNRRTLDVLAAKCYFYHMRCYELTDQLNQIKSFLHSRLRTATLRNDFEGQAVLLNCLLRLYLHYNLYDQAAKLVSKSVFPESASNNEWARFLYYLGRIRATQLEYSEAHKNLLQAIRKAPQHTAVGFKQIVQKLAIAVELLLGDIPDRSLFRQPTLRRTLAPYFQLTQAVRSGDLARFNEVIENFGQKFQADHTFTLIIRLRHNVIKTGVRMINLSYSRIHLADIAKKLKLDSSEDAEYIAAKAIRDGVIEATINHEKGFMQSKETTDVYCTTEPQAAFHQRIAFCLDIYNQSVRAMRFPPKSYNKDLESAEERREREQQDLEYAKEDDDDGFIVQVRLCVALAALILHTIWPNAIQELIFCLQPHRILLLDLLVIIPEEFQSRTMTREQIFRLVQEFLQLELKQAVIKCYHEQLLNLVL-QVADEYAVDAITSIYSHPEMKYPNSVLKLIDKISSVDVVLQKAIQESNPDNIYSLFIQVGEAHSRLLLDAVIDRPEHKILKLMTVVLQCSATPGYFPVDETCSEQAFNFWYTLQDDIRASDQ-RKIEAIFNPLFQSLIDLLVKVQYPTESIFSDEEKESFRCYRQDIGD--SFMYCYNVLRVTFLTSLLTHFTLSRPWQHLEAVLYCLGSIAENVEIDEDVYL-KEIVRALPTIPSSPRLLATAMEMMGAFCEEMLPNVISLLILGLKSVTIYATMALKDLTRECQAVIKPLAPDILCACQELKSKERARLMASIGHV----------------------------------------------------------------------------------------------------------------------------------------------------------------------------------------------------------------------------KVKVKWKQLFPDVELNTPVLFKAQLFALTGVHPGRQKVMIKGTVINDDEWGTMLLMMGTVEELPSPKEKIVFAEDMSENELAAALDLPAGLTNLGNTCYMNATVQCLRTVPELKDLRNFQGSVTAALRDLYDAMDSATIHPLIMLQVLHVAFPRFAEKSEHGQQDANECWTEVMRMLQNFIDQYFGGTFVTMKCTEAENESIENFLQLSCFIS-HEVKYLQAGLKSRM-QENITKFSPTLNRDAVYKTSKISRLPAYLAIQFVRFFFKERESVNAKILKDIKFTLCLDVFEMCTEDLQLKLIPMRNKFKELEDKFSFVDDGSSNSAYYELQAVLTHKGRSSSSGHYVAWIKREWFKCDDDKVVTSEEILKLSGGGDWHCAYVLLYGPRVLEALINRKLPRELLLRIFSYLDVVSLCRCGQVSKAWNVLALDGSNWQIVDLFDFQTDIEGAVVENISRRCGGFLKKLSLRGCKSVTDISLRTFALNCNNIEELLNDCKKVTDSTCASLSRHLNLASCSEITDLSLKVLGEGCQHLEHINISWCDQITKYGVEQLARGCRKLRAFISKMCINDEAVVAMARYLEVINLHGCSTITDDAVQCIAQSCANLTYLCVSNCPHLTDQSLIAMAQRCLKTLEVASCSQFTDAGFQALARTCHKLENMDLEECVLITDNALFYLAAGCPCMQRLSLSHCELITDEGIRHLGNSVLELDNCPLITDASLDNLVMCLERIELYDCQLITRAGIRRLRPELKVHAYFAPVTPRQRYCRCCVIL??????????????????????????????????????????????????????????????????????????????????????????????????????????????????????????????????????????????????????????????????????????????????????????????????????????????????????????????????????????????????????????????????????????????????????????????????????????PTHIPWVEKYRPVDFADIVGNEEAVARLAVFAAQGNVPNVILSGPPGVGKTTTILCLARTMLGASYKDAVLELNASSDRGIDVIRNKIKMFAQTKVTLSAGKHKIIILDEADSMTEGAQQALRRTMEIHSSTTRFALACNMSDKIVEPIQSRCAVVRFNKLTDKQILNKITEICHKEKVNFTTDGIEAIVFTAQGDLRQAINNLQSTVDGFEKVDSENVFKVCDEPHPLMLKEMIKHCIDGNFEEAYKIIVHLHKMGFSSEDIVGNIYRVMKTYEMAEYLKLEYIKEIGLTHMRVAQGVTSLLQLSALVARLCKKTFKPPYLKKDHSLTKPYQGTGMTIPNWDFFGSTMVTTSFIRLTPDQQSRQGALWNNVV-SLPVRFVIG-----------SFPYVEDCRRSGPVFGNMDFDGLGIFLDTYANQNGVHNHGHPYISSMVNNGTLHYDHDRDGTHTELSGCEAKFRGVDHNTHILVRYENDVLTVKTNIEGKNEWKECFTASGVQLPTGYYFGVTAATGELSDNHDVISIKTFELEEDRSKILPSAAPPRDHIDDAMSGLKLFLIVVCAIIGIAVVVFQKQQETSRKRFYQTRDSFDEIVQKNCRVVDINDMFLPHSTVTHVPDIKQLGIDPIFPNRTNLLHIHNMAMSRAFFYSYILQKVDDTEPGFMYYFLSSIADVAANRFINSSAIYYGPNMAFTPSYKGFYNKTMPLFAPRAFRADDFNDPYHLRGTSTLNTIAAADLGAIDNNYTSTFYKINEWYNAWLPDLTKRHDSKTTYTVQITGTNETFVWHGPPAASDTPGPVKWVRPYFDCSRSNKWVLGASVPIPDIFPRHTGWRHIEIPLYVAVAVMELDFDRLDINQCSIGEGN-PAPNYFAGTARCKNETTQCEPIHGYGFRRGGYQCRCRPGYRLPRVVRTPYLGEIVERSTESEYRNGFQCERIGYIAVRTQNVETLTGLSRRIDPNFRGDVAFGKEEQLENQARMALRLANFISSFSQVVDPKELFAEFRVPDRPLTEDQIIGEALSAVIGDRRLQGLGVYFDRNQFFAPY--AYRLERNTRKFFVVDTMKINIRYNSSGIRYDHYPKQYQAAELGYWTSPYFDCGGFHNQWLVTYAAPFFGWDKIKSRLEFKGVVAVVKLDELDLNQCNAFKDTHKCDRKSSRCVPIPGRKYSGGYKCECLQGYEYPYNDPITYFDGQILEAPSRFDTLREFRLTTAAKYGGRYMVTMLPGDGIGPEMMNYVKTIFKVGGIPVDFEEVHLDSMHEDIDNVDEAITSIRRNGAAIKGNIETREHSKHFRSRNVELRLRLNLFANVIHCKSQPGIQTRYKNIDIVLIRQNTEGEYSCLEHESVSGVVESMKIVTKKRSEQIARYTFEYAKKYNRKKVTAVHKANIMKLSDGLFLNTARDMSQEYPDIEFNDMIIDNCSMQLVSNPHQFDVLLLPNLYGNILTNIACGLVGGPGITSGRNFGEEYAVFETGTRNTGKSVAGKNVANPLAMINASADLLEHLGLEKYAEIIRDSIYKVINEAQIHTPDMGGHNTTTDVVNYILDYKQIKYMTLSESPNEFKLDNPPTDCVQSVKFGLQSSQYLLSASWDCTVRLYDITNNQLRAKYTHSAPVLDCAFQDTYNVWSGGCDHQVKIYDFNSSSETVLGSHSAPIRCVEHAPEVNLMATGGWDACVKLWDPRTPCATGSYAQPDKVYSMAVCGDKLIVGTAGRRVLVWDFRNMGYVQQRRESSLKYQTRCIRCFPNKQGYVLSSIEGRVAVEYLDPSPELQKRKYAFKCHRNKDTTGIEVIYPVNAISFHSGYNTFATGGSDGYVNIWDGFNKKRICQFHKYPTSISSLAFSPDGTYLAIASSFLYETDEQRDIPPDSIYLRRVSLEQLRQYTVVVADTGHFETINKYKPTDATTNPSLILQAAQKSQYQHLIEDAVKFGKQHGKNLEATMDYLFVSFGCEILKIIPGRVSTEVDARLSFDVEGSVKKATNLIKLYEERGIKKERILIKLASTWEGIEAAKILEKQHGIHCNMTLLFNFAQASACADADVTLISPFVGRIYDWYVTATGKKEYEPADDPGVKSVTRIYNYYKRHGYKTVVMGASFRNINQVKGLAGCDLLTISPDLLEQLNEKNVELEQVMGVEKA----------------------------------------------------MSVAAYRPRVSVYTEKNENSGVSVKLPAVFRAPIRTDVVNFVHTNMRKNRRHPYAVSKKAGHQTSAESWGTGRAVARIPRVRGGGTHRSGQGAFGNMCRGGRRFGPTRVWRRWHRKINRNQRRYALVSAIAASGVPALVMAKGHRIEEVPEFPLVVSDKIQEYTKTKQAVRLLRKVRAWRDVERVYNSKRFRAGRGKMRNRRRIQALGPVVVYSKDSGLTRAFRNIPGIETINVEKLNLLRLAPGGHVGRFIIWTESAFRKLDDLYGTWKSESKMKRHYNLPQPKMANADVSRLLKSDEIQKVIRPRITKVIRRKVKRNPLKNPLVMRRLNPYAPVLKKYARLTDERRKWARLKKKKLGDKVKDTKKKEYKAELLKKKKLAQGNVLKKILDAIKDLVNEASWDCSPSAMSLQAMDTSHVSLVSVNLKADAFRKYRCDRNVTLGMNLGSLAKIVKCAGNDDEITIKAPDDGDKITLVFEANSDVEYSEYEIKLMNLDTEYLGIPDTPYAVTVKMPSAKFQRICRDLNQIGDAVTISCAKDGIRFSASGDLGSGSVHLSQTANADKPEESVTVKMSEPICLSFALKYLNNFSKATPLSSQVSLQLSPDVPLVVEYEIGYIRYYLAPKIDDADQRCSKVAVLGASGGIGQPLALLLKHSPLISHLSLYDIANTPGVAADLSHINTRAQLSGHLGPEQLAAALQGCDVVVIPAGVPRKPGMTRDDLFNTNASIVRDLVEACAKNCPKAMVAIISNPVNSTVPIASEVYKKRGVYDPKRIFGITTLDVVRANTFVAELKSLDPTKVNVPVIGGHAGITIIPVLSQATPPVSYPKDQLDALTKRIQDAGTEVVKAKAGAGSATLSMAYAGARFAISLLEAINGKNVVECAYVKSDVTEATYFSTPILLGKTGIEKNLGLGKLNDYEQELVKNALPELKASIKKGEDFVNKEFEWLLNEEVNVILNQLIILECCRRFPV-VRPEKYQL-MMKMNVTLSGDNITYADITMKL-HKHTIRTNIQSDAAWKLHQIQDATNHLTAALCFLKYDFKTADEVIQMINGVMSCLQKGRSSLIVPKKRTIEELESSRNMKSIKPSLPNDLAVNFHIQAHKLVCAVYHM-QKDPVK--VHDQSVPWLSEALVLFTVALQFCQQLKDKVSVFQYKDMIPTFIDILKNLGADISDLKQIIDVCDVTFKDVESVL--NSVVS-LLIAFCDKLTKPPSNKICLR--VLQNLFEGLKYQVYVSLVKVAADQILLVFNDIVKVKSSFGNDKVQRLFRLLHDALSELASKVMIELLSTYTEENASQARDDAYRCIVSFLADPNTFLMDHLLTLKPVKFLEGEPIHDLLTIFVSEKLNAYINFYNSRKDFV-DSLGLLHERNLQKMRLLTFMQMAESKKEIPFETIENELQIKASDVEAFVIDVLRTKLARARVDQVNKKVLVSSTMHRTFGRQQWQQLRDTLNLGQVEHTIKT---FLG-TR-KKK----IEEGKQAIDEYEERSMIEELSDFKELIKILVNWINDELSDQRII--VKDLEEDLYDGQILGKLVEKLSGMRLDVVEVTQNEDGQKHKLRTVLENVNRLLGVKWSVEGIHGKNLVQIIHLLVTLIRHYRPPI--RLPVNVTVNLVVVQKR-EQLTGTYDELGMRVEPRDAFDTLFDHAPDKLQIVKRSLINFVNRHLNKINIEDLDPNQFSDGLLLVFLMGSLEGYFVPLGIFTTP-TDPIADVTTKETALQPDNYVNTSKLHNINVAFQLMEDAGIRQKVRAEDIVNADLKSSLRVLYMIFTKMSHRSSQICETTEKDLRDLLNIPDNYRVLFMHGGAQGQFSAIPMNLCAADYFVTGTWSAKAAKDASKYVQYTGISDQSVWKLDPSARYLYYCDNETIHGVEFPLVCDMTSNFLTRPVDVSKYGCIFAGTQKNCGMAALNIAIIRDDLIGNMDICPPVFDWKVINDNKSVYNTPPCYAIYVTLCLKWIKKNGGVEEMKKSEQKSRLIYDVINDSNGFYVRSRMTVPFRV-GGPNGNEELERKFVVEAEMIQLKGHRTVGGMRASLFNAITSQELKPPYRTRPEWRDVEPIVAIQYSEQFADAFDYFRAILRDELSERALELTRDCITLNPSNYTIWYFRRRCLRALDKDLREELPYIEDIIMGNPKNYQVWHHRKAIVECLEKKLIQRALDPKNYHAWQHRQWVVAEFALWDDELRFTEELIDDDVRNNSAWNHRFFVVSHTNDIKLITALEFEMRNAMNIEEILNFFHKVIPSYPLHPQLDGSFSLGVNFEMRWKYPNRGVAIIVDRFDELLKFVPHMHNFLSRQRVRYKIYVMNQADNLRFNRASLINVGFLISRQECDYIAMHDVDLLPLNPDLSYAYPTVGPFHVAAPELHPKYHYKSFVGGILLLTRVHFELVNGLSNKYWGWGLEDDEFYARLKEAKLNISRPVGIKTTAKDTFLHVHDRSNRKRDTARLLNQKEVTRKRDRQTGLNTVQYEILRQNELTIDGAPCTVLNVKLICDHRITPWCVFSNILPGDVITESGFMRGHGELSASVAGAVEPINKLISVRPVKTRFNGDIGDVVVGRVIEVQ--QKRWKVETNSRLDSVLLLSSVNLPGGELRRKTAEDELMMRKYFKEGDLVVAEVQSVFQDGSLSLHTRSLKYGKLGQGVLVRVSPSLIERRKHFHNLP-FGAHIILANNGYIWISSCGFVREAIARLRNCVLVLASAKMMISDTSCTYCYEAS-VARLTKENSTRNIN----????VTFGTVVIGGVTRLTKSGLSMVDWHLFKEFPPMTAEWEREFNKYQQYPEYKLRNSNMNLEEFKWIWWMEYAHRSLGRSIGAIFFLPAVFFWYKKWFNRAAKIRVVALGSLLAFQGLLGWFMVKSGLQEKPRVSHYRLAAHLGTALLFYSLAFWSGLSHLLPSLKMLSFKKTKGLIFVTALSGALVAGLEAGLIYNSFPKMADRWIPSDLFAKPLWRNFFENSSTVQFDHRLLGELSVTACWLRSRRLPLTPRMRLAANMMMIALAQVSLGIATLLLYVPKPLAASHQAGALTLLSTALWLGHELKLVRR-IAKWGEGDPRWIVEDRPDAVNVNNWHWTEKNASQWSKDKLTELLSGVRVDDDQLGWCIDEVTSIEGEAVANNRKAKLIFFYEWVVKMVNGS-TVVGKIEVPNLSEENEPKDVEVEVSVTDRTLEALMKSKGADLIRKQFAEYIKSLREEFSQGMILPTAKGTINSAVNASSKLETAMLKLETMKCTADEFYRALTMVAAFTNGNCNGRFELYDGTVSGTFTRLKVIGQRWRLKSWPADHYSDVIEIKQKDDCTLVQIGVPKSKLDETEHGWKNFYFQRMKHTFGFGAMLMDPESFLEMANQVTKLKMFPYFELAHAIISCLYVREDLAQGSHPFSRKHPFACWASCMISIFSGLILANFLLGEPILGALKSSNQVLLATGVWYLMFYSPFDVAYKICKFLPVKIVLAAMKEVIRCKKVHDGVVHAAKIYPNGYLNMVIIGTVKGNGAAFLKVIERIYRGVWTPNAIEFLSPTFPTKASIAASIIFIIDKKTDWISAPHSLVYFGIVIFFVYFKLSAMLLGIHDPFLPFENLFCAIFLGGLWDALSRVIKSEATKKKD??????????????????????????????????????????????????????????????????????????????????????????????????????????????????????????????????????????????????????????????????????????????????????????????????????????????????????????????????????????????????????????????????????MQDGVPTFKCVLVGDGGTGKTTFVKRHLTGEFEKKYVATLGVEVHPLLFHTNRGPIRFNVWDTAGQEKFGGLRDGYYIQGQCAIIMFDVTSRVTYKNVPNWHRDLVRVCENIPIVLCGNKVDIKDRKVKAKSIVFHRKKNLQYYDISAKSNYNFEKPFLWLARKLIGDPNLEFVAMPALAPPEVQMDPEWQAKLEQEMKQAQEVSLPDDDDDDLVAEYFTPLLKESKFKETGVLTPEE------------------------------------PVPCYKRCKDIEEKVIDAD-GGWVDTHDDDDDDGVAEDMDEFMDEEDKATTIVQTRTYDLNITYDKYYQTPRLWLVGYDETQKPLAIEQMYEDISQDHAKKTVTMETHPHLPG-VMASVHPCRHAEVMKKIIQTVEDGGKLAVESYLIIFLKFVQAVIPAIEYDFTQNFTLMSRFDNCIPYEKLQANLKIVKSRLNRPLTLSEKVLYSHLDDPQNQDIERGKSYLKLRPDRVGMQDATAQMAMLQFISSGLPRVAVPSTIHCDHLIEAQIGGVKDLARAIDINKEVYNFLSTAASKYGVGFWKPGSGIIHQIILENYAFPGVLLIGTDSHTPNGGGLGGLCIGVGGADAVDVMANIPWELKCPNVIGVHLTGNMSGWTSSKDVILKVAGILTVKGGTGAIIEYFGPGVESISCTGMGTICNMGAEIGATTSIFPYNNRMRDYLIATNRKEIADLADENTELLTSDKNPKYDQVVEINLSELEPHVNGPFTPDLAHPVSKLGESAKKNGWPLDIRVGLIGSCTNSSYEDMNRAASVASQAIKHGLKTKSMFTVTPGSEQIRATIERDGQAQILRQFGGVVLANACGPCIGQWDRKDVKQGEKNTIVSSYNRNFTGRNDANPATHAFVTSPELVTALSIAGTLEFDPLNDELTGANGEKFKLKAPSGDELPNRGFDPGEDTYTPPA-DGSRVSVDVDPKSQRLQLLKPFDKWDGKDLTDMVVLIKVKGKCTTDHISAAGPWLKYRGHLDNISNNMFIGAINAENGVANKVKNLLTNEWGPVPDTARYYKS-------------????SREHAALEPRHLGGRAIIVKSFARIHETNLKKQGLLPLTFDNASDYDKIQPNDKISLLDLQKLAPG------MFKNTFQSGFLSILYSIGSNPLQIWDKKVKNGSIKRITDNDIQSLVIELSGANVATTFITCPAQARLSLGIKLPYLVMIVKNMKKYFSFEVEVMDDKQCKRRFRASDFQSQTRVKHFICTMPLRLDEGWNQIGFNLADFTKRAYGTNYVETVRVTVNANCRLRRIYFADRLYSEDELPPEFKLYLPITRLLTQAKYRYQDQAKRDVQNALQHYRNLVDKYVFPDGTVRDLLCLDGTVPVSYRYNIPVCIWLTDTHPYTAPICYVRPTHDMTIKQSKHVDGSGRIYLPYLSDWTAKG-SDLLGVIQVMIIVFGETPPVYAK-PKPYPTTNSGTITEEHIRASLLSAVEDKLKSRTREQVQAEVEVLKKTENDLNKGKTKLEEIVSRMEGEVEVEDAKDAQLAELDVDNAFGPTQPLYKQLLNAFAEENAVVDAMYYLGEGLRKGTIELEVFLKHVRELSRRQFMLRALMQKCREKAGLP-------------------------------------------------------IGVAAQNSYKVASGAFTGEISPMMIKDVGAEWVILGHSERRNVFGETDELVAEKVDHALDEGLKVIACIGELLKEREAGKTMDVVSRQMKAIADKVKDWNLVVIAYEPVWAIGTGKTATPEQAQEVHAYLREWLGTNVSKDVGESTRIIYGGSVTAANCKELAREKDIDGFLVGGASLKPDFVEIVNAKQMASGPSQKRKIVANGVFKAELDEFLRRELAEDGYSGVEVRKTTNRTEIIILATRTQSVLGEKGRRIRELTSVVQKRFGCEDGVVEMYAEKVSDRGLCAIAQCESLRYKLIGGLAVRRACYGVLRFIMESGAKGCEVVVSGKLRGQRAKSMKFVDGWMIHSGEPTNDYVDTAVRHVLLRQGVLGIKVKIMLPWDMAGKKGPKRPLPDNVKVLEAKEEQYPAQPYSEPKEEVISPMDALKEWSMTTLKCTRQLVNEKLGKCPRTVDVELEAEIEHLRDTQRKYAAILKMAQQMTTQYQLIASQITLYEMMNEMSMRESKNLGVDFRQNADTLRTVAKNGEKLILALRFFVSNLSTLANKSIEDCVFTIRSFESARLEYDAERN--GDKLRSRYEQLRDDVQVKMKFLEENKAKVMHKQLILFHNAFAAY-ASGNASALDSTLKQFSISWLEK----------------------------------------KKVELQQVQDSQALIRARLLRENGKYLPKQSFLMRRHFFNNTDNGYFYMGRNRPSVQANPMTDPTMMTDMLKGNVTNVLPMILIGGWINWTFSGFVTTRVPFPLTLRFKPMLQRGIELMSLNASWVSSASWYFLNVFGLRSIYTLVLGENNAADQTRVMQDQMSGAALSMPTDPKQAFKAEWEALEISDHQWALRNVDNMAHYKGAASEAGRALQLQKKREKAKEEMEIRKRKIEDELKINNIGNKFASHYDAIEAQLKSSTIGLVTLDEMKARQEDVVKEREKKLAQKDEERKLREIEQKKEQKERQKRQIQTLSFRFDDRKMTKNPDVDTSFLPDRAREEEDKRLREELRQQWEHKQEKLKDEDIEITFSYWDGSGHRRSVKMKKGHSIYQFLHRALEILRKEFHELRAVSADQLMYVKEDLIIPHHYTFYDFIVTKARGKSGPLFSFDVHDDIRLLGDVTVEKDESHAGKVLLRSWFERNKHIFPASRWEPYDPTKNYDKYTISDRKNKVPTVPESILKKRKLRAKQKAKNAVNAIKRRQVKKQKRSLIFRRAEQYIKEYRRQEHDVIRLKRQAKLHGNFYVPAQPKLAFVLRIRGVNGVSPKPRKVLQLFRLRQINNGTFVKLNKATINMLRIAEPFIAWGYPNLKTVRELIYKRGYGRENGQRKALTSNDIIEKKLGRYGIICMEDLVHEIYTVGAHFKQANNFLWHFKLNNPRGGWRKKTTHYVEGGDFGNREDKINNLLRNMISVLTAEQLWKGVTSVSNAGRKRGRGKGAGRKTIKDLNKGQVIGVGKVNMIWPGLNAPVVRGRELVERRELPPDKDYEEKLVKIRNEMDVHRKVRIHPLERGWSGTRMAGRWIGPPDPVAGDEFTGFDTAVLQMRPLFRMTGNFGKTKRIAVLSITGNKNGLAGFAIGKAKDARTAMRQSKNRAGQRLRYLELFESTVLHDFYSRCGATAVFVEKKPRGYGIVAHRCIKAICETIGITDLYAKTEGQTKNYINLTKAFIVGLMQQKSYQQMADEKRLHIVELREERDQYPVVLASPSSCRTDAEIPSDEHLDFHLYIYNGKVKQRDVKRPPNYMGSIGWEQYLKKRDVTKNRAKVRVALICKHYSLTSFLYQRKLHFRIPPRFSDEKVKWINKQRVLIFASRGITYRDRHLLLNLRGLLPHSKADSKME--KKDINEIAEMKNCNKCIYFENRKQADLYMWMANIPNGPSVKFLVENVHTMEELKMTGNSLKGSRPLLSFDPRFDQSPLLKELFIQIFSTPNHHPKSQPFIDHVVTFSCLDNRIWFRNYQIVDESDASLAEIGPRFILNPIKIFNGSFGGPVLWSNPSYISPNRHRAIIKKQAAEKYKHD-----------MAVGKNKGLAKSGKKGVKKKVVDPFTRKEWYDVKAPSMFNVRNVGKTLVNRTQGTKIASEGLKGRVFECSLADLQNDEIAFRKFRLIAEEVQGKIVLTNFHGMDLTTDKLRSMVKKWQTLIEANVDVRTTDGYLLRVFCIGFTRKWPNQIRKTSYAQHTQVRAIRKRMVEIIQREVTSADLKEVCNKLIPDSIAKDIEKSCQSIYPLHDVMIRKVKVLKKPKFDLGKLLEMHGEGKTTTSTEPMKVDRPEGSPPP-----IKIGIIGGTGLGRDTEFLLDETPFGVTSDGTVVEGSLQGVPVVVMARHGREHTVSPSNVNYRANLWALKHQLACTHVLVTTACGSLREQVKPGDVIPDQYIDRTRARSFYVSHIPQARPFVQDILVEHPRVTAVTIEGPRFSTLAESRLYRTWGADIVNMTTVPEAQLAAELGLPYAALALVTDYDCWHESEDESVCVELVQRLKQLSVIAKKVLAVAVKRIAQSAIMMKVLRHEEFEEGCKAACNGPYDGKWSKTMIGYGSEDQHFVVELTYNYGIGSYAMGNDFLGYTIASQTAIDRVKQHNWPSAEVDGKLVVLSPDGYKFSLLGDEARASPDPVVKVTLASSHLERSINYWHDLLGLTVHERDEKSALLSYGEHQCKLELVDIRKPVIHAKAFGKIAFSCPTTELPAIEEIMKINKQKILTPLVSLDTPGKATVHVVILADPDGHEICFVGDEAFRELSQVDQQADELLRAAIDGDKSDDWYSEKNKKKPPARLPPLPTPKDLLKLYRVRASRHLSQNFLLDKISQRIVRSAGYVLEVGPGPGNMTRHILEQGPRELYVVEKDRRFLPMLEMLADIAHPGQMKIVIGDVMDFNFEQLF-PEDAKRDWLDIRIVGNLPFNVSTPLIIKWLRQISQRTGLWRYGRTQLILTFQLEVAQRICAAQRCRLSAMCQNCSVETSTSFVPPPLVDVGVVKFTPLSPITLPFNIYEKFNRHLFHKFIRNDLSMEMFSRTDIDPCYMLSTQEIGELCKIYQQFCEESGLFDYDYRARRLIPIVIEQTGRGERAYDIYSRLLKERIICVMGPITDDLSSLVVAQLLFLQSESNKKPIHMYINSPGGVVTAGLGIYDTMQYILPPIATWCVGQACSMASLLLAAGTPNMRHSLPNSRIMVHQPSGQAIGQATDIQIHAEEILYLKKRINSLYQKHTKQDLARIEDVMERDKFMSPEEAKQFGLVDVVLEQPP????????????????????????????????????????????????????????????????????????????????????????????????????????????????????????????????????????????????????????????????????????????????????????????????????????????????????????????????????MQRVVKAKTHKGKKVLQKRQAQIIEPNKAVFIRGSTANDKMVKLMKDICLIKKPNSVFLSKKNPFENSTKIEFLTKNDSSLFMFASHSKKRPNNLVFGRTFDNHILDMVEVGVENYKPLADFKTPKISLGTKPIIIFGGEPFEEMKRFKNLLLDLFSGDAIRLTGIENVVMILAAEILVRNYRIALKKSGTKLPR-VELEEMGPRLDMVMRRTHLASDDLFKRACKHPKQTKPKKVKNIKKDPFGSTLGRIHMERQDYKLQVRKLKGLK?????????????????????????????????????????????????????????????????????????????????????????????????????????????????????????????????????????????????????????????????????????????????????????????????????????????????????MNRLFGRGAPKEPPPNLTDCIANVDSRVESIDKKILRLDAELIKYKEQMKKMREGPGKNAIKQKALRVLKQKKMYEQQRDNMHQQSFNMEQTNFATQMLKDTKTSVDAMKLGVKQMKKEYKNINIDSIENLQDELEDMMESANEVQEVLGRSYGVPDVDEEELEAELEALSDELNADDTTYLDMRAAVGAVRGKHTLPDLPYDYNALEPTISAEIMQLHHSKHHAAYVNNLNVAEEKLAEATHKNDIATIIQLESALRFNGGGHINHSIFWKNLSPKGSGDPDGELLKAIKDNFGSIENMKSQLSTAAVAVQGSGWAWLGFCPKSKRLVVKPCPNQDPLQPTTGLIPLFGIDVWEHAYYLQYKNARPDYVKAIWNVVNWKDAYSRLSQFIDVDVNDGFVEDVLQSGVDLRSYSKQVEKQLRDVENGCVADYINESGNIANLHAQITSCDQILERLEHMLCTFQADLGNICQEILTLQEQSVSLNVRLKNKQAVRSQLSQFVDDMIVPQPVICHIFDTPASEKQFLEQLHVLDHKIAFVKEQSFRDAHSCHDVAEILNSLKLKAIAKIREYVLKKIQSCKKSLSNYQIPQNALLKNKFFYQFLLTHDRGSAREIQSEYIDTMSKIYYSYFKEYLHRLVKLEYEEKPDKDDLMGGEDVSKTSLFGKPSLKNKSTIFTMGCRADVLSTDLEAPLIVPHAAQKAETKYPMESLFRSHQYALVDNACREYLFVSEFFMANAPTAHELFNAVLGKTLSLMTKHIEDQFQSSYDSIALFLCLHVVYRYRLLAHKRTVPALDSYWESVVKCLWPRFEYVVRLHVQSIKSCDATKLGMCDTRPHYITRRYAEFSAAVMTVNDTFPDERVSILLGSLQNEVENFILKMASQFQHAKDQLIFIINNHDMILGVLLEKTKEDSKESESIKLQLNKRIQDFVEELLYPHFGGMICFVKDCEAYLEKNDVEALKREEKKVTSLVKSFNVGWRKALEDISREVMNCFSNFKNGNNIQQAALTQIIQYYHRFQKIVSQAPFKNNPVRNEFINIHQLMVEVKKYKTNFAYFLTIDAHADECFFDKVTTGTKMGLTFEVVEGGFLDIDVKITGPDAKVIHNEERGSSGKYTFAAHMDGMYTYCFGNKMSTMTPKVVMFSMDVGEAPK-TGQMDTEGDHNKLEEMIKELSSSLTAVKHEQEYMAVRDRIHRSINESTNSRVVSWAFFEALVLVAMTLGQVYYLKRFFEVRRVVMRLTLILKLPKNYSNLPERYIKKQTEFVVWETPKLPNYQRRVIRWRYDIHRPWEEGFQRNAPNVFVYVEPIFRGDRVEILRGKDKGKQGLVNYIVKERNWVLVEGLNCEEKPLLINRDVALVDSTDNKPTHVEWRFDEEGNKLRVSARTGRIIPIPSAFETIDYKYREQSKDTAADEVKKVTFVP-KVCTFEMEIMQEMNISEDRVPYPMYWYIANKTDAVLWLTRLATIVFTVLYVTGSPYSYYQKALIGNGATSALRLHQRLPPFRLTREYFSMLLLEDSAHYLLYSIIF-VPIT-LVLLPIALFALLHFVVMLDRMGSMVE---RQRSILQTVSLCEIFLMPLVLVSILTGAYLFTPFIYYRFLYLRYASRRNPYTRNMFHELRLATEQM----CPLVIRPIAFLERLAPVILLAILVYYNFPKLDPSEKEHLKLPKNIDDAKKLGRVLSRYSDKTVLSGFLVTYVFLQSFAIPGSIFLSILSGFLFPFPLALFLVCLCSAVGASLCYLLSYLVGRRLVLKYLPHLLNYIIFLRTTPFLPNWFINLASPVIDVPLMPFFVGTFLGVAPPSFVAIHAGTTLHKLSSSLSWTSIIMLLLFAGLSLVPVLFKLKGKF????????????????????????????????????????????????????????????????????????????????????????????????????????????????????????????????????????????????????????????????????????????????????????????????????????????????????????????????????????????????????????????????????????????????????????????????????????????????????????????????????????????????????????????????????????????????????????????????????????????????????????????????????????????????????????????????????????????????????????????????????????????????????????????????????????????????????????????????????????????????????????????????????????????????????????DDEGEDLFGPELDVYDAVEAEMRERDKGKMRRGLESIDNLDDTRGMSVREWVSQAPKREIYNRFKNFLRTFREKIRQMEENKQSFDVDYILANDEQVLAFFLPEAPIEMLDTFNLAAKEVVLSMFPAYDRIAKEICVRITDLPLLEDLRSLRQLHLNQLIRTHGVVTSTTSVLPQLSLVKYDCQKCGYILGPFVQNQEVKPGSCPECQSLGPFSINMEETIYQNYQRITIQESPGKVSAGRIPRSKDAILIGDLCDSCRPGDEIELTGIYSNSYDGSLNIANGFPVFSTVIMANHILKKDTDEDIKKIVALSKDFIVDRIMASIAPSIYGHKSVKRAIALALFGGQPKDPAKKHRIRGDINVMICGDPGTAKSQFLKYVTKIAPRAVFSTGQGASAVGLTAFVSPVTKEWTLEAGALVLADRGVCLIDEFDKMSDRDRTSIHEAMEQQSISISKAGIVASLQARCSVIAASNPIGGRYDTFSENVDLTEPIISRFDILCIVRDEQLAKFVVRSHIKNHPIPQDLLRKYIMYAKERIHPKLDHERIARLYSELRRESELTGSIPITVRHVESIIRCAEANAKMHLRDFVDVNMAIRVILESFITQKFSVMRMGLLDDIEQYFGTRNLYDVLGVKQTASEREIKSAYRKLSLKIHPDRVQDDKKVEATKKFQVLAKVHFILSDEDKKAAYDESGIIIDEDEMDNQANWEQYWRLLFPKVTVKDIDSFLAKYVGSAEESGDLKELYLRYKGDMDKIFESAICFDEERTDVVLILAISLMVLGKIGVKKRRKLEMKAEKRAMRDRELEERDERKQRMTIEEERRKDEQRAKAEQEKREEEERKIKEEKERQELEEYLKLKQSFVVEEEGFDQDIDENEAQDALKEFIDYIKHTKVVLMEDLAARFGMRTQDVINRIQDMLAQELLVGVIDDRGKFIYITREELESVAKFIRQRGRVSIAELVESSNSLIRIQPDVEALEVEARVTAAKHVANMLQRPDQLEKVDQFKRRVMHKKASVEGMLKSAMQTQLDGVRTGLIHLQTSLQDIQDVKAKEIEETFVIPNLDKLRQVREESLRHSQYAAAMENLKHIFNVPESVQKTRDWINEGNLLLAHQLTDLENSRDDLLYELHRLPSTSAADKNMLKHYFSDVEKLSEELGKQIWLIIRLCLNSVRTEPQVIVTALRIIEREEKLALKRFDST-GFMPQGRPKQWRKRVFEILEEAVSERIAGNQFESRHENKMWLVRHLEVTRQLILEDLKVIKTACVPCFPPHYDIVKELRLYHRCLSFHLQDL-AQLEGNEYVTLLNWVQAYSAAFAATHADKAFENKLRGDLQNNLAKGYITALLQNLREDMAKTNVRSRERPDE-------------------------------------------------------------------------------------------------------------------------------------------------------------------------------------------SPTDDNVVAREEEAIQLDGFNFEQMEKMREKAEKFVFQAEVSRLMKLIINSLYTNKEIFLRELISNASDALDKIRFLSLTDKSALDAIDELVVRIKADKDNAVLHITDTGIGMTKDALITNLGTIAKSGTSDFLQKVEETGNLNELSDLIGQFGVGFYSSFLVADRVVVASKSNDHPVQHIWESESGEFSVVEDPRGDTLKRGTQVSLHLKEEAKDFLQEDTLKALIRKYSQFINFPIYLWISKTQIVEEPIEDE--TTEEEKKSEEGDEAKVEEEKEE-KPKTRKVEKTIWDWELINTSKPIWTRKPDSITDDEYHEFYKSITKDTQEPLARTHFIAEGEVTFRSLLFIPKVQPGESFNRYGTKTSDIKLFVRRVFITDDFQDMMPTYLSFIRGVVDSDDLPLNVSREMLQQHKLLKVIRKKLVRKVLDMINKIADDLYEAFWKEFSTNLKLGVIEDAANRQRIAKLLRFHSSSTSSTGWTSLGDYVTRMKEKQDHIYYVAGANWEEVSKSPFVERLLNLGYEVLYLTDPVDEYCLSNLPEFNGKKFQNVAKDGLKLDDSEKAKDKQEEYEKRFEPLLKWLTSEALSERVSSVKISQRLHDSPAALVASQFGWSGNMERLARSNAHAKSQDITRDFYLNQKKILEINPRHPIIKELLRRAEKDDLDANTRQMALLMFEVATLRSGYMLEDPIDFSRRMDKHIIKSLNVDDSVPVDDEEARKRKKNGLTVGEMTVMTVGEIIKELIVAHQENRDVNLSKLKSDISRKYGLESQPRLVDIISAVPYEYKKILVPKLRAKPIRTASGIAVVAVMCKPHRCPHINMTGNICVYCPGGPDSDFEYSTQSYTGYEPTSMRAIRAHYNPYLQTRNRLDQLRQLGHDVDKVEFIVMGGTFMALPAEYRDYFIRNLHDALSGHSSSSVDEAVKFSERSRTKCIGITIETRPDYCLKRHLSDMLDYGCTRLEIGVQSVYEDVARDTNRGHTVKAVTESFQLAKDSGFKVVAHLMPNLPNVDLERDIAQFVEFFENPAFRADGLKIYPTLVIRGTGLYELWKTGRYRSYPPSVLVDLIAQILALVPPWTRVYRVQRDIPMPLVSSGVEHGNIRELALARMADFGTKCRDVRTREVGIQEIHHKLKPYQIELIRREYVANGAWETFLSYEDVEQDILIGLLRLRKCSGETFRPELKNGCSIVREHVYGSVVPVSAKDPTKFQHQGFGMLLMEEAERIASQEHGSTKIAVISGVGTRNYYRKLGYQLDGPYMSK

'Aceria_tosichella' PDSKVNVLNQPLMEAAVIISGKPSSFVAGADINMIAQCKTETEEMKPFVAAVMGDCLGGGLELALACHYR-IAVDSPKTGFALPEVMLGLLPGAGGTQRLPQLIDIPTALTMMLTAKKLRASQAKKAGLVDMVVKPVGP--KLEEVAANQLADKTFKVRKRPLVENLARDF-VFNKAKDQVMKMTNGLYPSPLRIIEVVRGGIENKAFAELATTHSKALVGLYHGQTLCKKNRFGAPAQTVAVLGAGLMGAGICQVSIQRGHNVIMKDGLARGFDQIQKRAMEKEVTMARLQPQTDYTDLVIEAVFEDLGIKHKVVQEVEKDDCIFASNTSALPINQIAQASKRPENFIGMHYFSPVEKMQLLEVITTDKTSKRAAAIAVQAGLQQGKVVITVKDGPGFYTTRILSPVLSEAILLLQEGVTPKELDALKAYGFPVGAATLVDEVGVDVAMHVSKKVFEKRVDMVSGFKGRKSGKGFFVYRPLNEIIKKYTMEDHQMRLGTRFVNEAVLCLQEGILANPLEGDIGAVFGLGFPPFLGGPFRYVDTFGADKIVGMEKYRKFEPCSLLLEHA-RDRSKKFHMNGPIILLKEGTESDQGRAQILNNINACNLVANTIRTTLGPRGMDKLIIDGKGAVTITNDGATILKQLEVVHPVARTIVEIAKSQDAEIGDGTTSVVVLAAEILKEAKPFIEEGVHPQVIIRSFRNSLNIVLKKLDEVSVKLDNN-SDEIFIKCASTTLSSKMIAQKKAFFARLVVDAVKSLDDILPINMIGIKKVNGGALEDSQLIHGVAFKKTFSYAGFEMQPKVYENPKIALLNVELELKAERENAEIRVNNVAEYQNIVDAEWKILYDKLEKIAQSGAKVVLSKLPIGDVATQYFADRDMFCAGRVAEDDLKRTMRACGGNILTTCSDLNEQNLGKCGLFEEKQVGSERYNFFKDCPSAKTVTIIIRGGADQLMEEVDRSLHDAIMVVRRLYRKDSVVAGGGAIEMELSHHLRQQSRDVKGKEQTIMTALAKAFEIVPKQLCHNAGLNATAILSELREKHAHGELWHGVDVFSGTVANNLDLCVWEPAFSKKNSITAAIEAATMVLSIDETIKNAKSQAP-------??????????????????????????????????????????????????????????????????????????????????????????????????????????????????????????????????????????????????????????????????????????????????????????????????????????????????????????????????????????????????????????????????????????????????????????????????????????????????????????????????????????????????????????????????????????????????????????????????????????????????????????????????????????????????????????????????????????????????????????????????????????--------GFHALRDDALERANLIVNEIVQGSDNVVSLFDELSNCLCKVADLAEFIRVGHPQPRFRATAEQASIAISTLVEQLNTDRRLYDSLKKAKFND-------LDEHVAKLFLFDFEQCAINLDDDRRKKVLQLNESILKLGSIFAANANQGRLVKTEKLPQNITLINGLHADSDNEQVREFAYKTYLQPDKTQDEILTSLLRCRQDLAQICGFESYAHRAVKGSIAGSPEVVDFLDLLNDRIRHLANRDYDQMLQLKAWDVPYYSPYFSIGVCMDGLNLIFNELYGVRMQVDGELWHNDVIKLSIIESNELLGHIYCDLFERPMKQHQDCHHTIRGGCLRDGTYQLPIVVLVLSLPPALLTPSMVDNLFHEMGHAMHSMMARTKYQHITGTRCSTDLAEVPSILMEFFASDPRVLSRFARHHATGQPMPEELMTRWIKSKKVFTASETQLQVFYAALDQAYHSSNIFNTTDILARIQSQYYSIPYVENTAWQLRFSHLVGYGAKYYSYLVSRAVASAIWTRLFAENPLSRSAGQYREQVLAPGGGKPAQKIAEDVLIARSVVDSFMPSDSKKKRDAKKKEAAKVRASGNTSKG---VDELTAMLEKDLELAAQARSCTGVLGVPEQSRDIKIDNLSVTFHGVEILQDTRLELNYGRRYGLIGLNGCGKSTLFSVIGRREVPIQEQIDIYHLAREIPPLDKSALDAVLDCEKERIRLERLAEELAGQDDDDAQEQLIDIYERLDDMGADRAKAKAGFILKGLGFDKNMQLKKCKDFSGGWRMRIGLARALYLKPHLLLLDEPTNHLDLEACVWLEQELKTYKSILVIISHSQDFLNGVCTNIIHLDKTRLNYYTGNYDAFVKTREELQEHQMKRFNWEQAQISHMKDYIARFGHGSAKLARQAQSKEKTLAKMVNAGLTEKVTADKLVEFYFPSCGKIPPPVLMVQKISFRYSKSTPWIYKDLELGIDLDSRIALVGPNGCGKSTFLKLLCGEVMPEDGLIRRHSHLRIARYHQHLHEALDLDLSALDYMMKCFPDVTEKEVMRRIIGRYGLTGRQQICPMRHLSDGQRCRVVFAWLSYQVPHLLLLDEPTNHLDMETIDALADAINDFEGGMVLVSHDFRLIGQVAKEIWICENGGIRKWDKDIMQYKNHLRNKMLKERENMVLADLGSKITSALRSLGNATIINQDVLDSLLNQIVRALISADVNIQLVKKLSDNVKQVIDFDEMAQGLNKRRMIQMVVFQELVKLIDPGVKPWTPVKKKSNVIMFVGLQGAGKTTTCTKMAHYYRRKGWKCALVCADTFRAGAFDQLKQNATKACIPFYGSYEEADPVVIANEGVEMFKKEGFEIIIVDTSGRHKQETSLFEEMLSIQAAVKPHQIIFVMDASIGQACESQAAAFKSVVDVGAVIVTKLDGHARGGGALSAVAATKSPIIFIGTGEHIDEFEEFRVKPFVSKLLGMGDLEGLIDRVNELRLEENEDLMEKLKHGEFTLRDMYEQFTNIMKMGPFNQILSMIPGFEAGLLKGASEAESMSRLKRLMTMMDSMSDSELDSKEGVKLFSKQPTRLVRVAQGSGVRVQEVKELLKQYGNFAAVVKKMGGVKGLFKTGDITRNVNPNQMAKLNQQMAKMIEPKMLAQMGGVNGLQHMLKQIQTQ-LTKDEQFVKLKNYYNQHAKQMNMRKMFHDDPNRAQKFHVKLEKEEKHLLFDYSKNIINEDVMKMLIDLAKCRGVEEKRHAMVTGSKINFTENRAVLHVALRNRANRPIKVDDKDVMPDVNRVLDKMKGFCDKVISGDWKGWTGKKITDVVNIGIGGSDLGPVMVTEALKPYQRGPDVHFVSNIDGTHLATAIKKLNPETTLFIVASKTFTTQETITNATSARDWFLAAAKDKVHVAKHFVALSTNLAKVQEFGISPDSIFEFWDWVGGRYSLWSAIGLSIAVHIGFDNFCSLLEGAHYMDGHFYHAPLERNVPVIMAMLGVWYINCFGAETHAILPYDQYMHRFAAYFQQGDMESNGKYVTASGEQVDYATGPIVWGEPGTNGQHAFYQLIHQGTRLVPADFIAPVKTQNPISGGLHHTILLANFLAQTEALMRGKTESEARKELEAKGTDKEAVDKLLPHKVFPGNRPTNSIVVRKLDPFTLGALIAAYEHKIFVQGAVWAINSYDQWGVELGKELAKAIEPELKSAGDVTSHDSSTNMLINHIKANQIERKFEDKDKPSQIRQSNMKAAKAVADAVRTSLGPRGMDKMIQAANGDVIISNDGATILKQIQVLHPAAKMLVEVSQAQDVDAGDGTTSVVVLAGSLLDAAHKLLQKGIHPTVISEAFQSAAKTCREHLTNLAIPVDLNDLESLQRVASTSLNSKVVSQHSSLLAPLAVQAVLKIRQTDNVDLRNIKIIRKLGGTLDDTQLIDGLVLDSKFCSDFGSKRVEKAKIGLIQFCISPPKTDMDNQVVITDYTQMDRVLREERNYILNIVKQIKKTGCNVLLVQKSILRDALTDLALHYLGKTKIAVVKDIEREDVEFVTKTLGCRPIASLDHFTAEMLASADLVEEVTSAKYIKITGITPNPINKTVNILIRGSNKLVMEEAERSFHDALCVIRCLVKSSYLIPGGGAPEIHLSHHLTKLSHTMTGLQAVCYRSFAEALEIIPYTLADNAGLNPIGTITELRQKHANGDKNFGINMRKGTVTDMLSENVLHPLLVAVSAVTLAAETARSILKIDDLINTVRMTSKRVTYFWDPEVGNYHYGPNHPMKPHRLAVTHSLVMNYGLHKKMNIYRPYMASYHDMCRFHSDRYINFLYNVTPSNIHDYAHELQRFNVGEDCPVFEGLYSFCSRYCGASLQGAQQLNNKQCDIAINWSGGLHHAKKFEASGFCYVNDIVIAILELLKYHTRVLYIDIDIHHGDGVQEAFYLTDRVMTVSFHKYGNLFFPGTGDMYETGNESGRYYSVNVPLKEGIDDSNYAQLFKPVILDVIQYYQPTAIVLQCGADSLAGDRLGCFNLSIRGHGECVKFVKGFNLPLLVLGGGGYTLRNVSRCWTYETSLLVDEEINNEIPYSEYVQYFSPDFTLLLDKPCGDFTHNANSRLYLDTIYRHVSENLKRVAHSPSVQFQPTPGGWFSEES-------------------------MSDDNDFMCHDEEDYDLEYSEDSNSEPDVDLENQYYNSKALKDTDPNLALESFQKVLDLEKDEKGDWGFKALKQMIKINFKLGNHEEMMNRYKQLLMYIKSAVTRNYSEKSINSILDYISTSHRMDLLQEFYETTLEALKDAKNDRLWFKTNTKLGKLYFDRGDFKRLSNILKQLHSSCQNDDGTDDLKKGTQLLEIYALEIQMYTVRKNNKELKKLYNQSLHIKSAIPHPIIMGVIRECGGKMHLREGEYEKAHTDFFEAFKNYDESGSPRRSTCLKYLVLASMLMKSDINPFDSQEAKPYKNDPEILAMTNLVNAYQSNDIVGFEKILNDNRNSIMDDMFIREHIEDLLKNIRTQVLTRFVKPYTRVRLDLISKELNLTVDEVEALLVTCILDNTIEGRIDQVARVLELMKHHQNNARYSAMDKLASQLASLQTTVMSRIIKPVSLNPVQILKGEADEEQGEQARLSSFVGAYAVGDLIKSTLGPKGMDKILCGTGRDEGRVEVTNDGATILRAIGVDNPAAKVLVNIAKVQDDEVGDGTTTVAVLASELLKEAETLVSKKFHPQTIIGGWREATQVAKQALEKSARDDPQDVENFRSKLITIAKTTLSSKIISQSKDLFAKLCVDAVLRLKGSGNLNSIQIIKKLGGVLEESFLDEGFLLDKMPGNGQPKRIENAKILIANTPMDTDKIKVFASRVSVDSVAKVAELELAEKEKMKDKVNLILKHNINVFVNRQLIYNYPEQLFADANVMAIEHADFDGVERLALVTGGEIVSTFGDPEKVRLGTCDLIEEVMIGEDKLLKFSGVPLGEACTIVIRGATQQILDEAQRSIHDVLCVLATVVKEPKLVYGGGASEMLMANAIEELAKKTMGKQQFAMEAYAQALRTLPTIIADNGGYDSSQLIPQLRALHAAGDSTKGIDMNKGQVADMTELGINESLAVKRQALVSAAEAAEMILRVDNILKAAPRRRQPDHRGMASNL----IPPRLAALEGHDDVINQATILKDEDGILSISNDKTIRIWLKRERGSYWPSVCHLLPFAPTCFHFDQNLKRLFVGLENGTISEFLVTDDFNRIDHQRYFSSHQSKVTSILYSSTCRLLLSVGKDKQFHWDDAESGTRLGSHPFQNSCTAVQFDPQSKHVFVAEQNGQISMLKLEQNSCRHITTLHGHSASIKSLLWDPFNKWLFSAGADKVITCWDIGGCKGTAHELQGHRNRIGALCYSRMNKLISGGEDCAIIAWNMEAKRMEAPQWSESDDCQRCKRPFFWNFKSMYETKTIGLRQHHCRNCGKAVCGDCSQRRSTIPLLGFEYQVRVCDECFPLFSDVTRKPLAKFFSATHHVNCMDLNESKGMLLTSGYDRTITLWNLSEMS???????????????????????????????????????????????????????????????????????????????????????????????????????????????????????????????????????????????????????????????????????????????????????????????????????????????????????????????????????????????????????????????????????????????????????????????????????????????????????????????????????????????????????????????????????????????????????????????????????????????????????????????????????????????????????????????????????????????????????????????????????????????????????????????????FLDTGLMIKAIRPFHYQKLEVLYPSECRERAISYMGKLNLEFWYLNGNFMGSIDECVGQIPVMVKSKLCNLANATPKQFIRRKEDAEELGGYFIQNGNERIIRMLIAVRRNNPIGLSRNTWKDSFPFYSEFGVSMRSVGPGNQNMVLHYLTNGTAKLKVFYNARSVTMPVLMILRALVDYSDKKIFERLTAGKERQ-FFENSVITMMKLVKHGINRVDALEHLGSKLKWWSDLEAGKDLLRSSVAIHLDNDEDKFNCIALMTRKVYALAKAECAIENEDNPMFHEVYSSGQIYFMLLIERIELLLKGLKLCFDKHLEKMLTVVNPMKALVGTGNLQSQSGLGLKQAVGLSVTCEKINYLRFLSNFRAVHRGAFFAQMRTTACRKLYPEAWGFMCPVHTPDGTPCGLLNHLTIGCMVTVLVDGRLVCYVLRKYKVRGGVPNDIEICLIPKTGHPTQFPGVYIFTNIFRLVRPVINALNDYEFIGSFEQVYMDISINDKEVTTHTEISPTIFLSVLGCLIPYPDFNQSPRNMYCCQMTKQTMGSSSHTLRYRNDTKMYNITPQSPLVRPAIYDHYRLDEFPLGTNAIVAVISYTGYDMEDAMIINKASAERGFTMGVTKTITVDLKLDESGLPYVGSSNVPFCAYYKTKYEVYKYPEHAYVTDVKLIGSAQKATITFWQRRPFVGDKYANRHGQKGVCSLLWPQESMPFTESGMTPDIIFNPHGYPSRMTIGMMLESMSGKVSALQGEAIDATPFRFSEKDTASDHYGKLMEKYGFNYYGTESMYSGVDGSLMNAEIFTGIVYYIRLRHMVSDKYQVRSTGKVDQVTHQPVKGRRRGGGVRFGEMERDSLLAHGTSFLLQDRLFNCSDKTICTKCGSLLTVFIPHVLLYLTAE-LASVNISLLRETKHDIHRVQRNDPKLHPFALQREYQRALNASKLERVFAKPFIGNMGHGDIVSNLMKHQTKLSIMASGAYDGVIKIWNLTNRKCLRTIQAHNSQVRAMCDSGSNIKTILAKMDHHYEPYLITSGDKVELWEERKEPIRQWW-GADSTQCKFNSVETLSSDRAITLFDMRKPAPLRKVVLTMRSNQLCWNPMEAFKFTVANEDHDLHTFDMRNLAKPLALHKDHTAAVISLDYSPTGAEIVSGSYDKTIRIFHSRDVYYTKRMQRITDVIWSLDAKYIVSASDEMDIRMWRANASEKIGPKETNEALKKKFQHHPEIKRILRHRHLPKHVYKLKRTMLDARKKKLAKKLMMAGKYSDALPHFHEAINNDPNNYLTYFKRATVFLALNRPKSALDDLNKAIELNPNFTSALSQRASLHVKLGNLDEAHIDYERYLQSDPDNNEAKIEKLKEDALRLLEPIYSTQLMKTRANAFERVGEIRRAISDYRAVAKLSVDSAT-YLKIASLCYKLGEVEEALSNVRECLKLDPDHKSCYKPTKKLNKSMLSDCNIQQLPLIYSICRCLSKAGDGLKTCDQAL-DSSVICDKADLLVEKEDLAKLYQESQKLRQRAREGIEKIKKMQKQAKKRDYYKILAGESEINRAYRKLAAQWHPDRH-QDAEA-KKLAQAKFMDIADAKAVLTDPEKRQQYDRGEDPLDPESKFQFKFNFMLVLFETAQGYAFFKLHDDKKLAKVDKLVKTFQKGDDLSNLISLHHFEKFKSTSEAVEGAAALLDSKIGKKLKKAIKKSIVKSLEDELAVADPKLGTKIKDKFDITCVSTNAVQELMSLIRNKVEDLIPEWSNDDEMVMQLGTSHGIGRYKIKFSPDKVDTMIIQAVSLLDDLDKELNNYIMRCREWYGWHFPELSKILPDHMTYVRTILTIGMRSAATDVDLSEVIEEETANKVKEYSEISMGTDIADEDLENIKFLCQNIIELTEYRAQLYDYLRNRMMTIAPNLTILVGELVGARLISHAGSLMNLAKHPASTVQILGAEKALFRALKTKHDTPKYGLIYHAQLVGMSNQQTKGKMSRMLAAKASLATRVDALGDEPTNALGTEHRAKLETRLKMLEDTKLRVVNQYVWPKDRPDIKRRVVISLALLVTGKLISIQAPILFKNAIDCMNVGTDLILGYGAARAGSSLFNELRNSIFAKVASESIRQVAVNVFSHLHRLDLKYHLNRQTGALSRSIDRGSRGINFVLSSIVFNVVPTIFEVSLVSSLLYYKCGQFALVTLGCIGTFAFTRWRTKFRVQMNSAESQAGSKSIDSLINYETVKYFNNEKHELKQYQQASLKTTTSLAALNFGQQAIFSGWIMMLSAETTVGDLVMVNGLLFQLQMPLNFLGTVYREVRQSLIDMQAMFGLMRIKNKPLILNPSIVFDNVNFRNVLNGISFEVKSGKKVAIVGGSGCGKSTIVRLLYRFYDPLSGRVLINGHDIRDVSLRKQIAVVPQDAVLFHDTIKYNIHYGKFEEKVLAVSQLAELHDTIMKWYEAQVGERGLKLSGGEKQRVAIARAILKNSPILIFDEATSSLDSITEHKIMTALQAVENRTSVMIAHRLGTVVGADQILVLENGKIVERGHKELIRSLYSHLWHQQQS??????????????????????????????????????????????????????????????????????????????????????????????????????????????????????????????????????????????????????????????????????????????????????????????????????????????????????????--???????????????????????????????????????????????????????????????????????????????????????????????????????????????????????????????????????????????????????????????????????????????????????????????????????????????????????????????????????????????????????????????????????????????????????????????????????????????????????????????????????????????????????????????????????????????????????????????????????????????????????????????????????????????????????????????????????????????????????????????????????????????????????????????????????????????????????????????????????????????????????????????????????????????????????????????????????VQGSSSDYRIDVSKHDYLETLGVNVKAKNFLVYQGAVESIAMKNPREITSLFEEISHSMDKAEEELFVKKKGITAEKKEAQGEEAKRYQSIRNEYQIDLQLFKLYHIDKEMSRRKKKNAIEEDIKNSRNIDKSIRDELNLNKKRPSYIKAKENAAHIEKKLEMAKKSLTGARKSHANHEDIKELEQDLERVIQEFLIEDVELQEEQRAEYTKLKKKASSMSAKYLKDLDSLEREQKADSDRLEAEVAKQREIEHKIREKEENVRRIEKLTDLSEFKKREREVHAKRELTQELADISSLGDAKVDRSENERQRKKGEIVENLKKIYPGVYDRMLNLCKPIHKRYNIAITKIMGKSMEAIVVDSEKTGRLCIQYLKDQMLEPETFLPLDYIASKPIKERLRNIQSPSNVKLIFDVIKFEPQDIKPAVLFATNNALVCETAADANKVAFELGDGQRYDAVSLDGTFYQKCGFISGGSLELERRARRWDEKEIHSMKHKKERLAEDLKENVKKTRKEGDLMVITSQIQGFESRLRYSKIDEIKVRMDKRAVEIKKIRDLMNGVEDDIFKDFCAQLGVDNIRQYEERQSKASQEKERLLQYESEKNSIQSRLLYER--SKDTSEVEQEDKNLLSAKEAERREMHAIEEEMRKVEQLKNDKISQKTECDKVEELITEKKRALQTIQKEISTIQKNIMTLECKMDSKRADRHAVFMHCKMESIALPLKEGSLQRAYQSDNIKPDFDVLEMQQEIAFRKIQAPNMKADERLDSAKERLRNTDSELNNLRKQAKDVFEAVKRERECFDTVSQRVDSIYKQLTNNPSAQAFLVPENPEEPYLEGINYNCVAPGKRFQPMSNLSGGEKTVAALALLFAIHSFKPAPFFVLDEIDAALDNTNISKVARFIRQRTVSSFQCIVISLKEEFYGHGDALIGVAPDPGDCTISRIFAVDLGVPGPVLIRLKNGIEQNNLVALQYLYDEAFPELTAQFFGILYGE--LHYRHIYANVRCDSYFNYCHLFNFIVSAQPINLQLPHKWMWDIIDEFIYQF--QNCSLLIWNVHSVLNVLHSLIDKSNIIEQLKVVNSGGDPTEAGGEFGNHPLYRMLGYYSLIGLLRLHSLLGDYHRAIRGLENVDLNKQMMVKAVACQTTTYYYVGFAYMMMRRYSDAIRTCTNLLVYLQRSKKTFQQELVEKQTDQMYVMLALCLALHPQRIDESVTTQMQEKFYAKLQRGDVAEFEKCPKFVSAVQLSIFMEEVTQQLVIRSYLKLYKTMSIEKLAGFLLLCFKHKKQSMVLDGELMSGSDVDFYIDRDMIHIADTKVARRYGDYFMRQIHKFEDLYKMGPPDPILGVTEAFKKDTNPKKINLGVGAYRDDNGKPYILPCVKDAEKKVAAANFDHEYLPIGGNAKFCHAAAELAFGADSSVIKDKVNVTVQGLSGTGSLTLGAAFLRDHFPGSKDLYLPSPTWGNHIPLFKKNSFNIKHYRYYDAKTCGFDLKGCLDDISKIPEKSVIVLHACAHNPSGVDPKPQEWELISELVKKRNLFPFFDMAYQGFASGDIDRDAHALRLFIKHGHQVALAQSFAKNMGLYGQRVGAFTLTARDPEEAARLMSQVKIVIRPMYSNPPLHGARIAEAVLTDKALYGQWLKDVKGMADRIISMRKALRSGLTREGSKRDWAHVTDTIGMFCYTGMTADQVTRLWNEFSVYLTKDGRVSIAGITSKNVDYLAHAIHQVTK???????????????????????????????????????????????????????????????????????????????????????????????????????????????????????????????????????????????????????????????????????????????????????????????????????????????????????????????????????????????????????????????????????????????????????????????????????????????????????????????????????????????????????????????????????????????????????????????????????????????????????????????????????????????????????PYPGFEYTGKLRPYPRTGPRKVPDHIKKPDYADHPEGRALSEEDIKESTHIKILNEQEIESMRLVSRFAREVLEEAAKITDVGVTTDEIDRAVHEAAIERDCYPSPLNYYKFPKSCCTSVNEVICHGIPDKRPLENGDIVNIDVTVYHKGFHGDLSETLIVGEPSEQHKKLVQVTWECLQKGIAAVKPGVKYREIGDVIQKHASLHGFSVVKSYCGHGIHRLFHTAPKVPHYAKNKAIGVIRPGHTFTIEPMIAEGSWRDTSWPDDWTAVTVDGLRSAQFEETLLATESGCEILTRRRTQKGKPYFMAS--MLQWMRDQSKRRRLLLSEQLGAQESDSLCQVLGTEDERTTSLADEQLIDTRYEYKGSSTFLKGTQSANPHNDYCQHYVDTGQRPQNFIRDYELHDRFEEYPKLKELIRLKDELIAQTATPPMYLKCDLRQGSLGQLDSKFDVILIDPPLEEYQRTQGVTKTKFWSWDDIMHLEIEEVAAPRSFIFLWCGSSDGLDLGRQCLRKWGFRRCEDICWIKTNKKNGHNKNLEPKAIFQRTKEHCLMGIKGTVRRSTDGNFIHSNVDIDLIISEEPPYGTYEKPDEIFHIIEHFCLGRRRLHLFGRDTTIRPGWLTVGPELTNSNFNSDLYKDYFSGSTDYLTGCTDRIESLRPKSPPPK?????????????????????????????????????????????????????????????????????????????????????????????????????????????????????????????????????????????????????????????????????????????????????????????????????????????????????????????????????????????????????????????????????????????????????????????????????????????????????????????????????????????????????????????????????????????????????????????????????????????????MRTFGDKPSPFQLEEGGELFYIGSEVGNYLRMFRGALYKKYPSIWRRTVSTEERRHISALGYGGHSIATNLTLLKASEVEDIFQGRDEKYKAVSLTTHDLTPRRLDHSRRNAISGVPSPTHHLDAVPCSTPIPKSRLQNKRVKTFPLIYDDLDPASLHRISNVGEVLVPIRLDMEIEGYKLRDTFVWNKNEISISPEQFADVLCDDLELPPQTFVPAIAQSIRQQVEAFTVPSNGDSGTDQRVLIKLNVHVGNISLVDQFEWDLSEKNNTPEQFAMKLCSELGLGGEFVTTVAYSIRGQISWNQKTYAFSEAQLSAIEFPYRSQDEGDQFSPFLETLTDAEMEKKIRDQDRNTRRMRRLAAQ--MGSNVSQLEREIGFPPNEHYFGLVNFGNTCYCNSVLQALYFCMPFRNKILESKAKSKRSKETLLTCLADLFYTIANQKKRTGSYAPKKFINRLRKENEIFDNYMQQDAHEFLNYLLNTIADLLRSDTSWVQEIFQGTLTNETRCLNCESISSKDEDFIDLSVDVEQNTSLTHCLRVFSKTETLDSEHKYYCEKCCSKQEAQKRMRIKKPPKILALHLKRFKYMEAQNRHTKLTSRVVFPLELRLFNDNSTEECEDRLYDLFAVVIHCGFGPNRGHYISIVKSYGFWLLFDDDFVEKLDTANLEDFFGLTNDSQKTSESAYILFYQSRD??????????????????????????????????????????????????????????????????????????????????????????????????????????????????????????????????????????????????????????????????????????????????????????????????????????????????????????????????????????????????????????????????????????????????????????????????????????????-----------------------------------------------------------------------------------------------LPLIPELNVYFNLLILINLIESGRYEEAVKCSDQLMQIVKSQNRRTLDALAAKCYYFHTRCYELTGQLHQIKGFLHSSLRTATLRNDFEGQAVLLNCLLRLYLNYDQYDQASKLVSKSVFPESASNNEWARFLYYLGRIKAIQLEYSDAHKNLLQAIRKAPQNGAIGFKQTVHKLAIIVELLMGEIPDRSLFREPTLRRSLAPYFQLTQAVRSGDLNRFGIVIENYGPKFQADHTFTLIIRLRHNVIKTGMRMISLSYSKIYLADIAKKLNSDDSQDAEYIAAKAIRDGVIEAKINHEGSFLQSKETSDVYCTPEPQAAFHQRIAFCLDIYNQSIKAMRFPLRSYHADAE--------------------------------------------------------------------------------------------------------------------------------------------------------------------------------------------------------------------------------------------------------------------------------------------------------------------------------------------------------------------------------------------------------------------------------------------------------------------------------------------------------------------------------------------------------------------------------------------------------------------------------------------------------------------------NVKVRWKKEFENVDLNLPTLFKAQLYTLTGVPIERQRLMCSGSMIKDDVWATTFMLMGSADKLPEPVEKPKFVEDLSETELAAAMDLPAGLTNLGNTCYMSATIQCLKVVPEFCVLKKFDASVTAALRDLYKSMDSAVVEPVILLRMMHLAIPRFAQRGEGNQQDANECWTELTRILQNFIDEYFSGVLGELKCDESPEESNENFLQLSCFIS-QDVRYLQAGLISRM-KETITKFSPSLNRDASYKVSRISRLPAYLTIQMVRFHYKGNVQTNAKLLRDVKFSMILDVYDLCTNELQEKLAPMRLKYKNADDLYTFEDDGSNNSGFYELRAVLTHKGRSSASGHYVAWIKKQWYKCDDDYVIDSEEILKLSGGGDWHCAYVLLYGPRYLRDLINTKLPKELILKIFSFLDIVSLCRCAQVSKYWNKLALDGDNWQSVNLFDFRVAVQGQVVENLSARCGDFLKRLTLRGCRSVSDSAMQTFSKNCRNLEEILDDCKQLTDETCKSLAEHLNIAS---------------CNQLKHINISNCNKIRPAGIESLAKNCTNLVSFIGTACINDESLKALSQKLKMINLNGCSAITDTGVKYLAENCHQLFYCCLSKCFALTDQALISLGQGCLKTLGLIGCHQLTDHGFQALTKNCKQLQDLDLEDCVLITDLTLYHLTNNCNNLKRLALSHCDLITDEGIKYIGTNYLELDNCIQLTDTAIDHLISCLKRLDIYDNNRISRQATKKLYPQLAIHTYFQASTPRQRYCRCCSIL??????????????????????????????????????????????????????????????????????????????????????????????????????????????????????????????????????????????????????????????????????????????????????????????????????????????????????????????????????????????????????????????????????????????????????????????????????????YLQMAWVERFRPKEFEDIVGNEEAVARLAQFAKEGNMPNIILHGPPGCGKTTVILCMARKILGPNIKEAVLELNASNERGIDVVRNKIKMFAQTKITLEPGKQKLVILDEADAMTEAAQQALRRIIEIYSKTTRFAFACNIFDKIIEPIQSRCAIVRFNRLNDEQLKRKLVDICNLMNVKYDDKGIQAVIYTAQGDMRQAINNLQSTHDGFEEITEDKVFKVCDEPPPIVIAKIIESCLKRRLDEAENTLAHLYSLGYCTEDIVSGMFRVVKSYNMPEFTKLEYLKQIGIAQIRVVQGVNSMLQMKGLISHFCAQAIKSPVYQREHSLVKPYQGSGMTIPNWEFFGSTIVTSNYIRLTQDTQSRQGGIWNVVPWQVEVAFRVHGHGSELFGDGLALWYVKEPPKGGPIFGNRDFTGLTVVLDTYANQNGVHSHGHPYISAMVNNGTQHYDHENDGVNSELAGCECKFRGLEHEARILVQYYNEELIIKTDIENSGIWRDCLSVKGVYLPTHYYFGITSATGDLSDNHDILSIKMRQLSQPKPDDLPRALPNRDKLNDGMSALRKLFLVVCVIAGVVAFLYQSKRNTARKRFYHQSDNMDEIIGRNCAVLDRNHLFLPMSTVSHIPDIKHFGIDPIYQNRTNLLQIHNIALNRAFFYSYILQKAQDAEPGFMYYMLAASADVSANPSVNSSAIYYSPNRAFTPSYNGFFNKTMPLFAPRAYRIDDYNDPYQLKGVSTMNTIAVTDLGAIDSNYTAEVYKINEWYSAWLPDLTKRHDSKPTYGVQISGTNETFVFHGPPGASDEPGPVKWQRPYYDCGRSNKWLVSASVPIADLFPRHTGWRHIELPIHVAASVIEMDFHRLDINQCPASEANGAESNYFADTAKCKRDTTTCEPIHGYGFRRGGYQCRCRPGHRLPKHVRAPYLGELIERASDFEYKQGFGCQKIENLAVKTQNVQTVTGVASRLDINMPGNIAHGKEHQFENQARAALRLSHFISSFLQVVDTNEMFAEFRVPDKPLTRDQVIGEALSTLIGDRQIVGLGVWFDRNQFFAPY--AYRLERNARNFFVLDTVKANIRFNSSGIKYDRYPIQYKVAQLGYWSEPFLDC-GLHNQWLISYASPFFGPDKLRLRVEFKGVVVVLKLSELDVNQCNAFKGTHKCDRKSTRCFPTSGRKFSGGYRCECKQGYEYPFNQPTTYIDGQMMEAPSRFDSLRFLSTSTKAKYGGRFMVTLLPGDGIGPEMMRHVKTVFAIGGIPVDFEEINLDSMNENIEKVEEAITSIKRNGVALKGNIETREHLKYFKSRNVELRTRLNLYVNIVHIKSQPSIETRHKDIDLFLIRQNTEGEYSSIEHETVPGVVSCLKVVTREKSEQIARYAFKFAVENNRKKISCVHKANIMKVSDGLFLNVAREVSKEFPQIEFEDIIIDNCSMQLVSNPWQFDVLLLPNLYGAVLTNIACGLVGGPGLISGANFGDEYAVFETGCRSTGKNIIGKNIANPLAIMNASADLLQYLKLDYHAQLIRNAVNKSLNEARVHTPDLKGQYTTTDVVNFIIDDIRHQL?????????????????????????????????????????????????????????????????????????????????????????????????????????????????????????????????????????????????????????????????????????????????????????????????????????????????????????????????????????????????????????????????????????????????????????????????????????????????????????????????SLEQLREHSVVVADTGDFELISKYKPTDATTNPSLILQAASKPQYAKIIEEAVKLGKGN---LEDTMDLVFVLFGCEILKIIPGRVSTEVDARLSFDVQKSVAKALKLIKLYEERGIKKERILIKLASTWEGIQAAKILENDHQIHCNMTLLFSLVQAVPCAEVGATLISPFVGRIYDYY----QVKKYEPFDDPGVKSVTKIYNYYKKYGYKTVVMGASFRNLDEIKCLAGCDLLTISPSLLEGLNETGLQVARSLDESKAK--DLEKVHYDEAAFRFYMNEDEMAHFKLGEGIRKFVQDQVKLEQMMKAKLGMSISTARPLISVYSEKNEATGSTIALPAVFKAPIRPDVVNFVHQNMAKNHRQPYCVNDQAGHQTSAESWGTGRAVARIPRVRGGGTHRSGQGAFGNMCRGGRMFAPTKTWRRWHRKINVNQKRYAMVSAIAASGVPALVQSKGHVIDSVPELPLVVSDKVQEISKTKQAVSFLKSINAWADVEKVYKSRRMRAGKGKMRNRRRIQRRGPLVIYGNDQGVTRAFRNIPGVDCVPVEKMNLLKLAPGGHVGRFIIWTESAFQALDKLYGTWKQAAKDKKGYNLPMPKMGNTDLTRLIKSEEIRKVLRPAQKKVVRRVRRLNPLRNQKAMLRLNPYAAVLKRNAILTIQKRMKQRLLAKKRGVKVPKYEAKPKKAGVKKPKRLVQGQLFKKILDAVKDLVNEASWDCSPSGMALQAMDTSHVSLVAAQLKSEAFDQYRCDKPIMLGMNLPNFFKFLKCAGNDDVITIRATDECDKVTLLFEDKNATETSEYELKLINLDNEYLGIPDQEYTVDIEMPSAKFSRICRDLNQIGDNVTISCVKDSVRFSISGDLGTGSINLSQNADADKPEDGVTIKMIEPICLSFSLKYLIQFSKAAPLSPRMKLSLKTDAPLVVSYEIGYMRYYLAPKMDEGE??????????????????????????????????????????????????????????????????????????????????????????????????????????????????????????????????????????????????????????????????????????????????????????????????????????????????????????????????????????????????????????????????????????????????????????????????????????????????????????ELEWLLKVDVKTSIKHLILADCAT-----STPTQYNL-TIKVNTTIEGYRITNADINIKLSSKHIIKTCIKNPFCWRLYQIQDANNHLNNAIDLLEAGFESAEEVLQLINDIMNSLLKSRSSLLTPKKSSVEELQHCQNMQSISPALPLDYTISFYVQANNLVCSVYQLSQSNNVK--NAEVSIPFLSDVLILLGLALQVCQQMLDKIQTL--NNLVPTFSPLLKELNPSLSNIQEVITGLDVVMEDVESVM--NAVVT-LLFTFASTLADKSSDKVSLR--VVKNLHDGLQYIAYTAMVKLATKRLPEVFTDVESVKSKYGFEKAQNLYRLLKTSASELASQIMIELLSTYTEENASQAEQDAVTCITSFLKDPNTFLLDHLLALKPVLYLEGKPIFDLLTIFVSEKLQNYIEFYNKNKSFV-DGLGLNHEQNLQKMRLLTFMQMAEGQKEISYETIMAELKIEHNEVEPFVFDVLRTQLVRAKIDQLNKKVLVQSTMHRTFGRPQWEQLRTVLNFAHIEKTIRMFLASLG-TR-RKKTVEQVNEGKHAIDENESRAIVDRSPQFQDLVQTLVNWINDELNDQRII--VKHLEMDLYDGQVLARLVEKLSGTKLDVVDVTQNEDSQRRKLREVLETVNRIFSIKWSVEGIHSRNTVQIIHLLVTMIRHFRPPI--KLPPNVTARVNVHMKQSEKLTEQYDETGMKVEPRDAFDTLFDHAPDKLAMVKRSLCGFVNKHLNKINLEELDPNQFSDGLLLVFLMASLENYFVPLGIFTQP-TDPVADMTGVQTALQHENYVNTSKLHNVNVAFQLMEDADVKNRVRAEDIVNADLKSTLRVLYEIFSKMSHRSKAIIEETEANLRKLINIPDDYSVLFMHGGAKSQFDTVPMNLCSVEYIINGSWSKMAVKEAEKYATYTRQPAYEELQEFDDVTYRYYCDNETIQGVEFPLVCDMTSNFLSRPIDVKKFGVIFAGAQKNCGIAGLVIVIIRNDLIGKMRIVPNVQNYQIMQKDKSLHNTPLTFAIYVALCVKWALERGGLEGMRFSKEKSQILYDLIDQSNGFYSRSRMNVVFLL-----SNKDLEAKFLEESRLFELKGHRSVGGFRASLYNGIELDDVKISYRTRPEWADVEPVMDIKYTPRFEEAFSYFRAMLRDELSERSLALTADCIELQRSNFSVWYFRRRILRKLTHHLENELEFVGKMIEDEPKNYQVWHHRKTIVEWLDKQLTASVLDSKNYHAWQHRQWVIKEFSLWEGELEFTDAQIAMDVQNNSAWNHRYFVVTETKRFWLAEEIRYVHKRIENNESTWSYLRGILSPHLLAFVLDKACDLDQIRSRYWKYIKLKV???????????????????????????????????????????????????????????????????????????????????????????????????????????????????????????????????????????????????????????????????????????????????????????????????????????????????????????????????????ILPGDLVTSHNLMRGHGELISSVAGRVVQINKLISVHAPRARFVGETGDVVVGRIIEVQVGQRRWKVETGARLDSVLILNHINLPGGELRRKTVEDEIMMRSYFKEGDLIVAEVQSTFQDGSLSLHTRSLRYGLVGQGALVRVPPNLVERCKVFINLPQIGVHLILANNGYVWISEYGFDRQTIARMRNCIHIMKTGKIMLNSNSCTKCFEES-IIGKLNK??????????????????????????????????????????????????????????????????????????????????????????????????????????????????????????????????????????????????????????????????????????????????????????????????????????????????????????????????????????????????????????????????????????????????????????????????????????????????????????????????????????????????????????????????????????????????????????????????????????????????????????????????????????????????????????????????????????????????????????????????????????????????????????????????????????????????????????????????????????????????????????????????????????????????????????????????????---MELIEFANKVLKLEMFPYFEIAHVIMVCLSVKSDFGKGAVIFSRRHPLACWLSCMFATFSGTILANFLLNEPIVGAFKNTQQVLLASAVWYLMFYSPFDLVYKLCNFLPFKLLVACMKEVNRCHKIHHGVLYASKIYPSSYFIIVLIGTVKGNGAGLLKVMERMFRGIWQPSTIEFIQPTFATKASIAASILFIVDKKTDFISAPHSLVYFGVVIFLVYFKLSSVLLGINDPFGPFENLFCAIFFGGIWDALEQTIKDKGGKTKSMSSDEGLVRRRVTDNNDDARNIKLSLLEEVFLLGLKDRQGYTSFWNDCISIGLRGCILAELVLRKRITLDKSDRRRTSLSLRKVHVINAEHTGDALLDEALKHIHDTHPPESIHSWIYYLSGETWNPFNLKFQLKNVRERIAKNLVEKGVLSTEKKSFVIFDMTTHPLVNCPIKSKLVRRIQDAVLSDWVSDPHLMDKRLLALIILAYHSDVLENAFGPLSDDDYETAMSRSRTLLDLDMDYESSKPNANEALWAVFAAISKMQQPVKSFKVVLVGDGGTGKTTFVKRHKTGEFEKKYIATLGVEVHPLVFYTKYGPIEFSVWDTAGQEKFGGLRDGYYIQSKAAIIMFDVTSRVTYKNVPNWHRDLVRVCDNIPIVLCGNKVDVKDRKVKAKAIVFHRKKNLQYYDISAKSNYNFEKPFLYLARKLTGDSTLEFVAMPALAPPEVHMDPETIRKLEAEMKDAEIAPLPEDDDDDLVAEYFTPVLKQSKFKQTGVLTPEEFVIAGDHLVHQCPTWSWSSASDKPYLPADKQYLITKSVPCYKRCKDIEEKVILTD-EGWVDTHDDNDDDDEAEDMDKFMEENDTSVNIVATRTYDLNITYDKYYQTPRLWLVGYDENLKPLSIDDMYQDISQDHAKKTVTMENHPHIPA-VMASVHPCRHAEVMKKIISTVEEGGRLYVHSYLIIFLKLVQSVIPTLEYDFTQNFTI---------YQQLQAKLKLVNKKLARPLTLSEKILYSHIDNVETQDIVRGESYLKLRPDRVAMQDATAQMALLQFISSGLPKVSLPTTIHCDHLIEAKLGATKDLSKAKEVNKEVYDFLSSAASKYGIGFWHPGSGIIHQIVFENYAYPGCLIIGTDSHTPNGGGLGGLCIGVGGADAVDAMASLPWELKCPKVIGVHLTGNLNGWASFKDVILKIADILTVKGGTGSIIEYFGPGVDNIACTGMGTICNMGAEIGATTSVFPYNGQMRDYLIATNRSEIAAAADDNTNLLSADKNAKYDKVIELNLDTLEPYVNGPFTPDRGHTISQLGESAKKNNWPLDIKVGLIGSCTNSSYGDMTRAASVARQAIEHGLKAKSSFTVTPGSEQIRATIERDGQAETFRKFGASVLANACGPCIGQWNRQDIKKGDVNTIVTSYNRNFTGRNDANPQTHAFVASPEIVTALAITGRLDFNPLTDELVDSAGKKFKLQPPHGDELPSRGFDPGQDTYQPPAEDGSKVKVNVDPKSERLQLLSPFQKWDGKDLQDLLVLIKVKGKCTTDHISAAGPWLKFRGHLDNISNNMFIGAVNAENGEVNKVKNTLTNEYGSVPDTARYYKSKGLGWVAVGEENYGEGSSREHAALEPRHLGARAIIVKSFARIHETNLKKQGLLPLTFNDPKDYDKVQQTDKIDLVNLKELAPG------MFRNTFQSGLLSILYSVGSQPLQLFETRVKNGMVKRVTDEDIKSLVLEITSSNVSTTYITCPK-ISKSLGITLSHIVLIVKNLDRFFTFEIEIIDDTKAKRRFRASNYQTRTRVKDFICTMPLKLESGWNHINLNLADFTRRAYGTNYVETSRITVNANCRLRRIYFADRQYTDEELPAEFKLYLPVQQLLSAAKYKHPEHTKQDILNACKYIKTLMERYTFPTGVSKELICLDGTIPVTFRYNIPVGIFISDNHPFEAPLCYVRPTRDMTIKTSRHVDGSGRVYLPYLSEWNKNT-SDILSTIQVMQIVFGQMCPVYQK-AKALPNSTTGTITEEHIRLSLLSAVEDRLKYRMKEQIQDEIEVLKKTSNDLNRGKIQLDDMKSRMAQEASLSEELDGQLQEVDPDEVYGPTQPLFKQLLDAFAEENAVVDAIYHTGEGLRKGRISLDVFLKNVRELSRRQFMLRALMRACRAKASLP????????????????????????????????????????????????????????????????????????????????????????????????????????????????????????????????????????????????????????????????????????????????????????????????????????????????????????????????????????????????????????????????????????????????????????????????????????????????????????????????????????????????????????????????????????????????????????????????????????????????????????????????????????????????????????????????????????????????????????????SPMDSLKEWSMSTFKCTKQLINEKRGLCPVTNDSQLQNDIEQLRGNREKLVQMLRFGQQMTDHYQLVRTQKQLHSLMNEMSIKCFTNLVDDFKKNAITLNVAINNGEKLITALNFYCSNLSTLIYKTIEDTLTTVKQFESARLEYDAEKN--SDKVRLRYEQLKQDVQIKMRFLEENTIKVMHKQLLLFNGAFASY-TSGNTAALDTTLKQFCIRFL--LKSDLLIDPDIRVWVFLPIVVITFLVGVLRNYVTILLMSTKKVDLQQIQDSQALIRARLLRENGKYLPKSSFLMRRHFFNNPETGYLTLAKNRPSTQPNPMNDPTMMSDMLKNNLTNMLPTILIGGWINWTFSGFVTTRVPFPLTLRFKPMLQRGIELASLNSSWVSSASWYFLNVFGLRGIYLLVLGENNQADHTRGMQDQMSGAAMSMPTDLKPAFKAEWEMLEIYEHRDTNSV---MAHYKGDANEATRAMHISRRREKAKEELEEKRKKIEGDVKLSTIDNQFSTHTDSVEAQLKSSTVGLVTLDQMRQKQENAVKEREKQLAQKEDGSLKIGLDHGLKKSSIPTKNSKSLSF-YDDTNVRKKPDIDTSLLPDRDREEEERRLREELAAEWRERQQRLKEEEIEITFSYWDGSGHRRVVRMKKGDSIYQFLQACLETLRKDFNELRSVSADQLVYVKEDLIIPHHYTFYDFIVTKARGKSGPLFSFDVHEDVRLVADASVEKDESHAGKVLLRSWYERNKHIFPASRWEPYDPTKTYDKYTISDKRSK??????????????????????????????????????????????????????????????????????????????????????????????????????????????????????????????????????????????????????????????????????????????????????????????????????????????????????????????????????????????CKSTAANVWKSVISVSAAGKKRGRGKGTGRVIAKDFNRGQQIGVGRHKLILPGLNTSVFAAKKPVEIQDLGKNDEFQEKLQAVRNEMNTFRKFRELPIERGFSGRRAHGRHAGQPDDHNETSFDGFDSIVLMLRPIQNMTGVMGRTKSMQALVVAGNKNGLAGFGMASGKDGRAVVRHARNRAAQALVYIPRFEGTVMHDFFSRYYQTTVFVERKPRGHGINAHRVIKAICEMFGITDLYANVEGVTRNQINMTKAFFLGLMNQKSYQDIANEKQLHLVDVREENYFYPQILASPEGVKTDSDIASGENLDFTYYINDGRVKLVTPKRKPFYEGDATWYKHLDRLDYGKNREKTKLILAAKYGSLDVFPHFKAFNRDDT-????????????????????????????????????????????????????????????????????????????????????????????????????????????????????????????????????????????????????????????????????????????????????????????????????????????????????????????????????????????????????????????????????????????????????????????????????????????????????????????????????????????????????????????????????????????????????????????????????????????????????????????????????????????????????????????????????????????????????????????????????????????????????IIVGIIGGTGLDQDSSLLTDETPYGRASDTQAIAGQIEGVDVFIISRHGKNHDVSPSHVNYRANLWTLVKQLNCTHVLVTSACGSLKEHIEPGHIILDQYIDRTRDRSFYVCHVEQRNPIMQQFILEHEGLTCVTIEGPRFSTLAESRLHRSWGCDVVNMTSVPEVQLATELATMYACILLVTDYDCWR-DDGECVSSSGVERMKDLGAKARKIIPGVIRRMLKDSIMMRVLRHEVFDEGCEAACNGPYDNKWSKTMIGYGPEDDHFVLELTYNYSVGSYKLGNDLQYLKISLKDLFDKVLSTEQVSSGESRNFSLKSPDGYRFLIETGRG-QSKNDVTEVCLSCTNLNRSKQYWTELLKMTPAEESSNETVLSYSEQQARLRLCQINTALDHASAYGRIAFACPSADLRGIQAAVEAANEKVLTPFLSLDTPGKATVQVVILADPDGHEICFVGDEGFRELSQVDPEANTLIEKSMADDKSNEWFEKKGKSKSDAKLPPLPRTRDLLHVYGIRAKKNLSQNFLLNQLIRGLVRAAGRVIEVGPGPGNLTRAILEQSPFEVLAVEKDRRFLPLLEQLADSVLPGQLKILLGDALDHDFENIFGPKQLEREWEDVRLIGNLPFSISTPLLIKWLHHISLRNSFWQYGRVPMLLTFQDEVARRICAYERTRLSVMSQNCHVQYGKSFTPAAGVDTGVVRLEPKQPLCVPFKLFEKFNRHLFHHNLNYDRIEHAFDKAQVNPPYMISNKETARLCEEYYKLCLEPALVEYDYRASGYVPIVIDKTGKGERAYDVYSRLLKERIICVMGPIEDNMASSVIAQLLYLQSEHNRQPIHMYINSPGGVVTAGLGIYDVMQYVQPSIATWCVGQACSMASLLLAAGAPGWRHALPNSRIMIHQPSGHASGQATDIQIHAEEILYLKKRLYGIYEKHTKQDYDVIHAHMERDRFMNAEQAKEFGLIDTIIESMP?????????????????????????????????????????????????????????????????????????????????????????????????????????????????????????????????????????????????????????????????????????????????????????????????????????????????????????????????????????????????????????????????????????????????????????????????????????????????????????????????????????????????????????????????????????????????????????????????????????????????????????????????????????????????????????????????????????????????????????????????????????????????IHPIKALQDNYMYLLVTRHAAAVDPVNASAMASAVAVDLKAILTTHHHYDHAHGNSDMLVYGGDNRVQALNKHGD-VIKIGTLIECLATPCHTKGHICYYVVFTGDTLFIAGCGRFFEGSSEQMNQNLLASLPSDTKVYCGHEYTVTNLKFALSVEPQNIIKNKLDWAKREPTVPSTIGEEKKINPFMRLQVKQFTDELEVMTVLRHKKNEFVMNRLFGKTKPAAPGPSLSDIGSNLDKRAEQFDKKIQMLDAELFKYREQMKKMRDGPAKNSVQQKALRVLRQKKQYEQQRENLSQQSFNLDQANFTTQMLIETKGTVDAMKAGVKQMKQEYKNLNIGEIEDLQDDLQDMMADANEVQEALSRSYGVPDVDESELEAELEALGDELQKEDSSFLDMTSYLSSVRSKHTLPDLPYDYNALEPAISAEIMKLHHTKHHATYVNNLNVAEEKLAEALHKGDTTAVVQLQNVIKFNGGGHINHSIFWHNLSPSGGGDPPSELLTLINGSFGSVDNLKQAMSTAAISVQGSGWAWLGYNKDAQKLHVTTTANQDPLQPTHGLVPLLGIDVWEHAYYLQYKNVRPDYVKAIWRVVNWKDVAERLHKARSAEIAHESPKDVPRTGVDIRVQRLEIEKQLKDLEIDCVADYMKEKENIIDLYHRVNSCDQILERLETILCKFQADLGNICQEIISLHEQTVSLNMQLKTKQSVRTKLGQFIEDMTIPQPVIQHIMYTPACDKNFSDHLVILDQKIHFFKEQDFRDALSCNDVHQTLLGLKTKAIYKVREYVLRKIHDCRKYLSNYQVPQTALLKNKFFYQFLLSHERERAREVQTEYLDTMSKVYHSYFKEYIQRLCKLEYDDKPDENDLMASDDQGANIIFNKSSLKNRSTVFTVGTRASVIKEDLEAPLIMPST-AKQDVKYTPEAIFRTVHYALLDNTCREFVFLRDFFMTSDQQTTDLFNSVFAKTLSMIHMHFNDQFKSSYDTIAIFLCLHLVYRYREMARRKKVTVLDPYWDSTVRCLYPRFEKLIQLQINSVRNFNNDKFNNVDTMPHSITRRYAEFASALSSINDTYPDERISQLLSDLQNEVKNFILRVAAVFGQPKEQQIFMINNYDHILSVFKQSNREDSKDIEEIKLQLNKRTQEIVEELLYPHFGSIICFVKDCEVFLERDDQESLRASEKKVGALVEAFNLNWQKALDEISRDILSSFSNFENGNTIQQATMAQLLQYHLRLQKLLENPIMKDSQRKSKLLGLHELMNYVKKYKTNF???????????????????????????????????????????????????????????????????????????????????????????????????????????????????????????????????????????????????????????????????????????????????????MRLTRFLRIPENYHNMPQRYVDRHTTFISKRAPRLPQYTQKIFRYKYDEWRPWQDDFKRNSEMVKVFVEPKFRGDRVEVLKGQDKGKQGVIIHMVKERNWVYVQGLNIKEEPYLIDEEVKLVDPSDLAPTDFEWRYDDQGLLLRVSTRTERVIPIPEAFETVDYVYKEQPKDTPADLVRQVTFEP-EVKTFEMDICETMGIKDERIPYPMYWY????????????????????????????????????????????????????????????????????????????????????????????????????????????????????????????????????????????????????????????????????????????????????????????????????????AYSVLAIVTCYSSDSPVSIRDYLKIPSTVGDAKRIGSLILRYKDDTVFGAYFSTYILLQSFCIPGSIVLSILAGYLFPALLALVIICICSTIGASTFYILIYN-RKKTLLKFLSNLFICVFLLRATPIFPNWTINLCSPLINIPLKPFVWGTFTGVAPLSVIHVWTGRILNDLSNDVNWQSVLMTSLVA-ISIVFLVFVFQTSTNSSQAKLDRIAIVNQDKCKPKRCNQECKRLCPVVKSGKLCVEVTPSDKIAFISEQLCIGCGICVKRCPFGAIEIINLPSNLDKDTTHRYGPNSFKLHRLPTPRPGQVLGLVGTNGIGKSTALKILAGKIKPNLGRWTDPPDWTDILAYFRGSELQNYFTKILENQLKAVIKPQYVDLIPKAFKGTVQEALDKKNETGRLEEFCKLLDLTPVRSRNIDELSGGELQRFAIALLCIQKADVYMFDEPSSYLDVKQRLKAAEAIRSLVTPKNYVIVVEHDLSVLDYLSDFTCCLYGKPSVYGVVTMPFSVREGINIFLDGFVPTENLRFRDTELVFKVAESAQEEIKRMCHYEYPRMTKALGGFKLNIEPGTFTDSEIIVMLGENGTGKTTFIKILAGGLKPDGDSDLPSLNISYKPQKISPKSQKSVKDLLMEKIFDMYRHPQFQTDVLKPLEIERIQDQLVTELSGGELQRVALVLCLGKPADVYLIDEPSAYLDSEQRLVAAKVIKRFLLHSKKTGFIVEHDFIMATYLADRVIVFEGQPGLDAKAKSPQSLLTGMNTFLDMLNITFRRDPNNYRPRINKLNSLKDVDQKRSGNFFFLEE----EDLFGPELDHYEAAEATMRQRDRMKSRRGLETIDNIEDTRGMTNKEWLSQATKNEIRNRFKNLLKTFKEKLRTMELNQQSFELEYILAQEQPALALFLVEAPHQMLDIFNQAAKEVVFSMYPAYGKIAEEIFVRIKDLALAEDIRSLRQLHLNQLIRTQGVINSATTILPQLSLVKYDCLKCKYMLGPFVQQQEIKPGTCPGCQSTGPFAINMEETLYKNYQRLTIQESPGKISAGRVPRAKDVIVLGDLVDSCRPGDEVDLTGIYTNSYDGSLNIATGFPVFTTVIQANSIVRKQSDADVHQIVNISKDPVVERIMASICPSVHGHSNIKRAIALSLFGGCAKNPSGKHRLRGDINILLCGDPGTAKSQFLKYTQTIAPRAVYTTGQGASAVGLTAFVSPVTREWTLEAGALVLADNGVCLIDEFDKMSDRDRTSIHEAMEQQSISVSKAGIVASLQARCAVIAAANPVGGRYNTFNMNVDLTEPIISRFDIICVVRDSVLAKFIVRSHRKSHFIDKDLLRRYIVYARDRFKPKIQMETIAQVYADMRRESAQTNSIPITTRHIESIIRCSEAFAKMHLRNHVDVRMSIKVILESFITQKHQEQKMTLLGEIEEYFDAKDLYQVLGIDKNATSDQIKKAYRKASLKVHPDRVGEKLKEKATKRFQVLSKVHYVLSDEERRRMYDDHGVIDSEGNLETGTDWLDYWRLLFPKVTVKDVDSFFDRYIGSEEEEKDLISIYNKYEGDLDKISDSHIGYDEERT???????????????????????????????????????????????????????????????????????????????????????????????????????????????????????????????????????????????????????????????????????????????????????????????????????????????????????????????????????????????????????????????????????????????????????????????????????????????????????????????????????????????????????????????????????????????????????????????????????????????????????????????????????????????????????????????????????????????????????????????????????????????????????????????????????????????????????????????????????????????????????????????????????????????????????????????????????????????????????????????????????????????????????????????????????????????????????????????????????????????????????????????????????????????????????????????????????????????-----TAHSQDEAGV-----------------EKHAFQAEVSRMMKLIINSLYTNKEIFLRELISNASDALDKIRVLALTDKDTLDSLGELEIRIKADDARGALHVMDTGVGMSKSELITNLGTIAKSGTSEFIKKSLDNPEKVQLNDLIGQFGVGFYSSFLVADKVSVRSKSHQEPFEHVWESNSTEFSVVEDDGSDQLKRGSVVTLHLKDEAKDFLKPDTLRELIKKYSQFINFPIYLWTSKTVTEEVPVDDD-----ESTD-KKDEDATSTTEEEKTKKKTKKVEKTVYDWERINVAKPIWQRKPKDISDEEYDEFYKSITRDHQPPIVRTHFTAEGELTFKSLLFIPKVQPTESFNKYGNKNDNIKLYVKRVFISDDFNDLMPAYMRFIRGVVDSEDLPLNVGRETLQQHKLLKVIKKKLVRKTLDMIKKIDDNKYIDFWKEFGTNLKLGIIEDQNNRNRIAKLLRFHSSKTGPDGWTSLEEYVKNMLPEQEQIFYIAGSSYDEVSQSPFVESVLKKGYEVLYLTDAVDEYTLSNLPDFDGKRFQNVAKEGLTLDKSKKRELYKKALEGKYAKLVSYLQETALKGKVHKVVLSERLSESPAALVATAFGWTGNMERLAKSNAHSKTNDATRDYYLQQKKILEINPHHPVIKELLKRVEADESDERVQEAASMVFAIATVRSGYMLQDLEDFGKKIERYMRTDLHVPLDAPVEEEQ---MAQLKLSHQEKQVLVVREIIETLIEAHEKNEDVDLSKLRNKIASKYATSKVPSLVDIISGVPHEYKPILLPKLRAKPIRSASGIAVIAVMC--------------------GPDSDFEYSTQSYTGFEPASMRAIRARYDPYLQTKGRIEQLQRLGHIVDKVEFIVMGGTFMSLPPDYRDFFIRSLHDCLSGHQSKSVAEAVKMSERSKIKCIGITIETRPDYCLKRHLNDMLDYGCTRLEVGLQSIYEDVAVDTNRGHTVKSVCQSFQLGKDCGFKMVSHIMPNLPNVDLERDLNQFVELFQNPAFRPDGLKVYPTLVIRGTGLYELWKTNRYKSYPPSVLIDLLAQALSLVPPWTRIYRIQRDIPMPLVSAGVEHGNIRELVLARMADYGMKCRDIRTREVGIQEIHHKIRPYNVELIRRDYVANDGWETFLSYEDVEQDILIGLLRLRKCSPGTFRPEFIENTSIIREHVYGSTVPVNTKNPVKFQHQGFGMLLMEEAERIALEEHGSSKISVISGVGTRNYYKKLGYQLDGPYMSK

'Fragariocoptes_setiger' --------------AGVIISAKPGCFVAGADINMLSNCKTETENLKPIVAAIMGDCLGGGAELALACHYR-IAVNNPKTVISFPEVMLGLLPGSGGTQRLPKLIDIPTALTLCLTAKRVRADKAKKMGLVDLVMHPLGPGLYLEEVAAKQLADGQLKVRKRPLMENLARDF-VFNKAKDQVMKMTNGLYPAPLKIIDVIRNGIENKTFAELATTHSKALVGLYFGQVLCKKNRFGTPAKSVAVLGAGLMGAGICQVSIEKGVQTIMKDGLARGQNQIKRRNIQRDTTMAKLFPQTDYSDLVIEAVFEDLALKHKVVKEVEEDDCVFASNTSALPIHMIAQASKQPENFIGMHYFSPVDKMQLLEVITTDKTSDRAASIAVQAGLRQGKVVITVKDGPGFYTTRILSPVLSEAILLLQEGISPKELDVLKSYGFPVGAATLVDEVGVDVASHVSKQVFGQRTEMVGGYKGRKSGRGFFVYRALNDILKKYTREDHQMRLGTRMVNEAVLCLQEG---------------------------------------------------------QDQSTSF-MQAPIILLKEGTESQQGLSQVINNINACQLVAQTIRTTLGPRGMDKLIVDGKGAVTITNDGATILKQLDVVHPVARTLVDIAKSQDSEVGDGTTSVVVLAAEFLKQARPFVEEGVHPQIIINSFRNSLQFVLKKLDEIAVKVNDN-SDEIFMKCAATTLSSKMIAQKKEFFARMVVDAVKSLDGHLPLNMIGIKKVSGGALEDSQLIAGVAFKKTFSYAGFEMQRKKYQNPKIALLNVELELKAERDNAEIRIDNVSEYQSIVDAEWKILYEKLEKIHKSGAKVVLSKLPIGDVATQYFADRDMFCAGRVPEDDLKRTMRACGGSILTTCNDLEDSNLATCELFEESQIGGERYNLFKGCPNAKAVTIIIRGGADQLMEEVDRSLHDAIMVVRRLYKKDSVVAGGGAIEMELAHHLRQHSRSIAGKEQSLAAAVAKAFEIIPRQLCQNAGIDATTILSQLREKHA----------------------------------------------------------------------------------------SQPRLSYKTISSVYGPLLVVDLVRYPQYGEIVQIKLPDGSRRTGQVLEFKGSKAVVQVFEGTSGLDVKNTQCEFSGELLRVPVEEDMLGRVFNGCGKPIDGGDDITPEKYLDINGEPINPASRDYPKEMIQTGISAIDVMNSIARGQKIPIFSAASLPHNEIAAQICRQAGLVRAHDNSDKD-NFAIVFAAIGVTSETARFFRQEFIANGSMENVCLFINLANDPTFERIITPRIALTTAEYLAYECGRHVLVILTDMTSYCDALREISSAREEVPGRRGYPPYMYSDLATIYERAGRVEGRSGSITQIPILTMPNDDITHPIPDTTGFITEGQVYVDKNLHGRKIYPPINVLPSLSRLMKSAIGPGMTRPDHSDVSNQLYACYATAQDVAPMKKVAGEEALTADDLLYLEFMEKFEKGFLAQDMHENRTVFESLDLAWKLLRMFPKQMLRRIPPKVLEEYYPHTRHV--------AFRLLIEEVVSASERAKSRSGSDNFEVVEIFDELSNCLCKVADLSEFVRVGHPHPRFQAAAEHASMAISSLVEKLNTDKRLYQSLKDSLRYS-----TTLDRYVAKLFIFDFEQSGIHLDDDKRKLVLQLNEDILKLGSIFTANSNQPRLVPVSCLPEEIALINGLCADSDNENLREVAYKHYLRVDERQENLLSKLLYARSRLARLCGFKSYAHRAVKESIISKPESIEFLDILNERIRPLAERDYAQMLDLKAWDVPYYSPYFSLTACLNGLNVIFNHLYDIRMQETGELWHNHVNKYAILDTNERLGIIYCDLFERHGKPHQDCHFTIRGGCSRDGTYQEPVVVLMLSLPPSLLTPTMVDNLFHEMGHAMHSMLARTKYQHVTGTRCSTDLAEVPSILMEFFAADPRVLSKFARHYATGDAMPNELMHSWIKSKKIFAASDTQLQVFYAALDQAYH-------------------------------------------------------------------------------------------------------MPSESKKKRDAKKKEAAKARQGGGTINGETNVDKVTAMLERDLELAAQARSCTGVIGVHPRSRDIKVDNLSITFHGHEILTDTKLELNYGRRYGLIGLNGCGKSTLLSVIGRCEIPIQSTIDIYHLTREIPPLDKNALEAVLDVEQERIRLEKLAESLANCEDEESQDQLMDIYERLDDMGADRAKAKAAYILYGLGFDKAMQQKKCKDFSGGWRMRIALARALYLKPHLLLLDEPTNHLDLEACVWLEQELKNYKRILVIISHSQDFLNGVCTNIIHMNKLRLEYYGGNYDAFVRTRLELLEHQMKRYNWEQDQIAHMKDYIARFGHGSAKLARQAQSKEKTLAKMVASGLTDKVVNDKLVEFYFPACGKIPPPVVMIQNISFRYNDKSPWIYKNLELGIDLDSRIALVGPNGCGKSTFLKLLVGEVIPQDGLIRKHSHLRIARYHQHLHEALDLSLSALEYMQKCFPEVKEREEMRRIIGRYGLSGRQQICPMRHLSDGQRCRVVFAWLAFQVPHMLLLDEPTNHLDMETSDALADAINDFEGGLVLVSHDFRLIGQVAKEIWICENSTIRKWEKDIIEYKNHLRSKILKETEFMVLADLGRQITSALRSLGNATIINQQVLDSLLEEIVRALLSADVNIKLVARLRQNVKQVIDFEEMAQGLNKRRMIQMVVFQELVKLVDPGVKPWQPVKACSNVIMFVGLQGSGKTTTCTKMAYYYQRKGWKCALVCADTFRAGAFDQLKQNATKARIPFYGSYDESDPVVIASEGVEKFQNEGFEIIIVDTSGRHMQEASLFEEMLSIQTAVQPDQIIYVMDASIGQACEAQAAAFKSKVDVGAVIVTKLDGHAKGGGALSAVAATQSPIIFIGTGEHIDEFEPFKVKPFVSKLLGMGDLEGLIDKVNELKLEENEELIEKLKHGEFTLRDMYEQFTNIMKMGPFNQLLNMIPGFGADLLRGASEAESMSRLKRLMTIMDSMSDSELDSREGAKLFSKAPTRVARVAQGSGVRQREVQELLAQHTKFAAVVKKMGGIKGLFKGGDMSKNVNPAQMARLNQQVARIMDPRMLAQMGGMNGIQNMMRQLQAQ------------------------------------------------LLFDYSKNLINEDVITSLLELAKSRGVEEKRHAMVTGANINFTENRAVLHVALRNRANRPINVDGKNVMPDVNRVLDQMKAFTESLINGTWVGFTGKKITDVVNIGIGGSDLGPVMVTEALKAYQCGPNVHFVSNIDGTHLHSVIKKLDPETTLFIVASKTFTTQETITNATSAREWFLQAANDSKHVSKHFVALSTNVPKVKKFGISETAIFEFWDWVGGRYSLWSAIGLSIACHIGFDNFVKLLEGAHYMDGHFYHSPLERNLPVLMAMLGVWYINFFNAETHAILPYDQYLHRFAAYFQQGDMESNGKSVTDTGERVNYQTGPIVWGEPGTNGQHAFYQLIHQGTRLIPCDFIAPVKSHNPIRNGLHHTILLANFLAQTEALMMGKTAEEARRELEAKGTPSDTLDKLVPHKVFIGNRPSNSIVVDKVDPFTLGALIAAYEHKIFVQGIVWGINSYDQWGVELGKELAKAIEPELKSSTAVTNHDTSTNMLINYIRDRQQDRSFKDKDKPGQIRQSNMAAAKAVADAVRTSLGPRGMDKMIQGANGDVTITNDGATILKQIQVLHPAAKMLVELSQAQDIDAGDGTTSVVVLAGSLLDASQKLLQKGIHPTIISDAFQEAAKKCRESLEQLAISVDLKDMESLQRVASTSLNSKVVSQHSSLLAPIAVNAVLKIRQTDNVDLRNIKVIKKLGGTLDDTHLIEGLVLDSKFCSDFGSKKVEKAKIGLIQFCISPPKTDMDNQVVITDYTQMDRALRDERNYILNIVKQIKKAGCNVLLVQKSILRDALTDLALHFLGKSKIAVVKDVEREDVDFICKTLGCRPIASLDHFTSDHLVSADLVEEVTSAKYIKITGIQPNPVNKTVNILIRGSNKLVMEEADRSLHDALCVVRCLVKSPFLIPGGGAPEIHLSHHLTQLSHSMTGLHAVCTRAFAEALEIIPYTLAENAGLNPISTITELRQKHASGDKNYGINIRKGTVTDMLGENVLHPLLVATSAVTLASETTRSILKIDDLINTVRMTQRRVSYFWDPDVGNYHYGKHHPMKPHRLSVTHSLIMSYNLHTKMNIYRPYNASIMDMCRFHSDQYIDFLSRVTPSNINEFSKYLTAFNVGEDCPVFEGLYNFCSKYCGASLQGAQQLNNKQCDIAINWSGGLHHAKKFEASGFCYVNDIVIAILELLKHHVRVLYIDIDIHHGDGVQEAFYLTDRVMTVSFHKYGSLFFPGTGDMYETGAESGRFYSVNVPLKEGIDDTNYATVFKPIITDVIQFYQPTAIVLQCGADSLAGDRLGCFNLSIRGHGECVKFVKSFGLPLLVLGGGGYTLRNVSRCWTYETSLLVDEEISNEIPYSEYVQYFSPDFTLLLDKPCGDVTSNHNSRAYLEAIVGMVRENLRCVAHSPSVQMQAIPTDFFKPEE-------------------------MSDDADFMCDDQEEYDLDFSDNSNSEPDVDLENQYYTSKSIKESDPQAALVNFARVLELENGVKGDWGFKALKQMIKINFKLGQYEEMMRRYKQLLTYIRSAVTRNYSEKSINSILDYISTSKQMELLQEFYETTLDALKDAKNDRLWFKTNTKLGKLYFDRGEFQRLSKILKQLHASCRNDDGTDDLKKGTQLLEIYALEIQMYTVQKNNKKLKRLYEQSLHIKSAIPHPMIMGVIRECGGKMHLREGEYEKAHTDFFEAFKNYDESGSPRRSTCLKYLVLANMLMKSGINPFDSQEAKPYKNNPEILAMTNLVSAYQNNDINEFEKILKENRNTIMDDMFIKEHIEDLLRNIRTQVLTKLIRPYTRIHIPFISKELNLDPHEVETLLVSCILDNTIEGRIDQVNQVLELTRQSHNNAKYLALDKLTNQLASLQTTMVEGQIRPISLNPVQILRGDADEEQGEQARLSSFVGAYAVGDLIKSTLGPKGMDKILYGSGRDEGRIEVTNDGATILRAIGVDNPAAKVLVDIAKIQDEEVGDGTTTVAVLASELLKEAEALVSKKFHPQTIITGWRQATQAAKKALEDSAVSDVDDPEKLRAQLMSIAMTTLSSKILSQSKELFAKLCVNAVLRLKGSGNLDSIQIIKKLGGALEDSFLDDGFLLDKTPGNGQPKRIENARILIANTPMDTDKIKVFASRVKVDSVAKVAELEIAEKEKMRDKVNLILSHNINVFVNRQLIYNYPEQLFADANVMAIEHADFEGVERLALVTGGEIVSTFGNPDKVRLGTCELIEEIMIGEDKLLRFSGVPVGEACTIVIRGATQQILEEAQRSIHDALCVLSRVVKDPRIVWGGGCSEMLMANAVAELAKKTSGKHQFAMESFATALRTLPTIIADNGGYDSSQLISELRAAHALGKKKLGFDMYQGCIADMSELNISEALVVKRQALISASEAAEMILRVDNIIKAAPRRRQADHRGMSVELREFETLELLSRLDGHQDLITQACILRNEDGVISISDDKTIRLWLKRDTGSYWPSVCHILPSPATCMHFDHDSRRLFVGLDNGTISEFMISEDYNRINHQRFFPSHQSRVTALLLAPSCRWLLSAGRDKHFHWYCSDSAYRLGSYQCTAPCTSVQFDELSKHVFIGDQHGQITMLELIQSSYKFVKTLGGYQTTIRFLEWDPASQMLFSAGSDHVIICWDIGGRKGTTYELQGHSSQVTALRYAKKSHLVSVGDDCRIISWSMKAQRTETPPWAESDNCQKCQKPFFWNFKVMFDQKTLGIRQHHCRNCGKAVCNDCSLNRTKIPPMGFEHSVRVCDECYAQFSNITCTSLANFYSAKHMITCMDLDESKKILLTTGPDRSIKLWDSSPIL-----------------------------------------------------DRKLVLIKELSQELVGQTIWLRARLHSSRGRGKQAFLVLRQQQYTAQAILRVSEGVTKEMVSFASSIPRESIIDVFGLVKQSPVKIESCSQHDVELELSELYVVSQAKPQLPLLLEDASRSEAESADPGMLTIIVNQDTRLDNR---------------------LFRASLNKRGFIEIHTPKIIMAASEGGANVFEVTYFKRKAYLAQSPQFYKQMAIAADLDRVYTIGAVFRAEDSNTHRHLTEFVGLDLEMAFNYHYREVVDVIAQMFVDIFKGLQERYEHEIKVIARQYPSQKFVFLEPSLILNYTEGVAMLRESGVEIGDEDDLSTPNEKLLGKLVKEKYNTDFYVLDKFPLAVRPFYTMPDAQNPRYSNSYDMFMRGEEIMSGAQRIHDPELLIERAQLHQIDVTKIESYIDAFKYGCAPHAGGGIGLERVTMLFLGLDNIRKTSMFPRDPKRLAP----------------------------MRGRTYKGRLYLEFWCLDGKMMGTFNEYVGEVPIMVKSELCNLNRLTPKQMIERYEDAEEFGGYFIANGNERLIRMLIAQRRNYPLGILRNGWKDAGPMFSEFGVSMRCVGDSGQNMVLHYLTNGTAKLKLFYKGQQLFLPLMLVARALVDHTYFRIFQLFMRGKESNAFYRGCVVSMIRLVKQSINSEQALAYIGEKFRWYSDLEVGEHFIKTSMAIHLPNNEDKLNCLVMMTQKLFALAKVECAIENADNPMFHEIHTSGQIFFTLLIERIDQFLSGLKMCFDHHMLRMLTIVRPLEFLIATGNLQSTTGLGMMQRVGISVMAEKINYLRFVSHFRSVHRGAFFAQMRTTACRKLYPEAWGFLCPVHTPDGTPCGLLNHLTESCIITVTVDGKLIGYVVRDHKVKGGIPNQIEICLIPRTAHPTQYPGVFIFTTMSRMMRPVFHRLEAVEYIGTFEQVYMDICLVEEEATTHRELSETAFLSVLGALIPYPDFNQSPRNMYCCQMTKQTMGQASHTLLYRCDTKMYAVTPQSPLIRPAIYDHYHFDEYPLGTNAIVAVISYTGYDMEDAMIINKSAAERGFTHGIMKTVTVDLKIDRDGLPFIGSMDDPICVYHELKMEFYKSSEPAYVLDVKLLGDLQKVAITLWLRRPFVGDKYANRHGQKGVCSLLWPQENMPFTESGMTPDILFNPHGYPSRMTIGMMLESISGKASALRGEPLDSTPFRFSENSHASEHYGQMLADSGFNFYGTERMYSGTDGNELIADIFIGVVYYIRLRHMVSDKYQVRSTGPIDQITHQPIKGRRRGGGVRFGEMERDSLLAHGASFLLQDRLVNCSDGCLCKHCGSIITVYLPYVMRFLVAE-L-------MRETKNDIHKVFRNDRKLHPHRIEREYQRALNATKLERVFAKPYIGNLGHGDIVNCLMKHQTKLSVIVSGACDGQIKIWNLASRRCVRTIEAHNSVVRSLCDSQSTIKTVIAKMDHHYRPFFVTVGDKVDLWEERNEPLRSWW-GADSSQVKFNPIEATSNDRGITLYDTRKANPMRKIVLEMRSNQVCWNPMEAFVFTAANEDHDLYTFDMRNLNKPLIIHKDHTEAVITLDYSPTGLELVSGSYDKTIRIFRSRDVYHTKRMQKLTDVAWTHDGKYIISASDEMDIRLWRARASEKIGPKNVQETLKKKFANHPEIKRISRHRHVPRHVYKEKREMLDSRKRK???????????????????????????????????????????????????????????????????????????????????????????????????????????????????????????????????????????????????????????????????????????????????????????????????????????????????????????????????????????????????????????????????????????????????????????????????????????????????????????????????????????????????????????????????????????VFVVFKTAQGYAVFKLND-----KVEKLVKYFKKSDKYSSLIELHHFEKFKSTAEAVESATCLIEGKVAKKLKKTIRKCIINDIQDELAVAESKLGTKIKEKLQINCVTSQAVQELMSMIRLKIDDLIPDWSNENNEIMQLGVAHGLGRYKLKFSPDKVDTMIIQAVSLLDDLDKELNNYVMRLREWYGWHFPELCKIITDPTTYVSTIMAIGMRSNAADADLSEVLPEAMQQQVKEYAEISMGTDIADDDLINIKYLCGNILELTKYRSELYEYLKNRMMTIAPNLTVLVGELVGARLISHAGSLMNLAKHPASTVQILGAEKALFRALKTKHDTPKYGLIYHAQLVGQSGTALKGKMSRMLAAKASLATRVDALQEDVDATLGTEHRATLEARLKSLEDGTLRKVSMYIWPKDRPDIKRRVIIALGLMIAGKVISIQAPFMLKHVVDYLN----TNQSYGAARAGSSLFNELRNSVFAKVATDSIRRVAVNVFSHLHCLDLNYHLNRQTGALSKSIDRGSRGINFMLTSLVFNVVPTIFEVALVSSILYYRCGQFAVVTIGCISTFAVTQWRTKFRIQMNKSDQQAGSRSIDSLINYETVKYFNNEKHELKEYQVASLKTSTSLAALNFGQQAIFSACIMVLAAEMSVGDLVMVNGLLFQLSQPLNFLGTVYREVRQSLIDMQSMFGLLNIKSKPLALTPAVVFDNVTFRLVLDSMSFEVPSGKKIAIVGGSGCGKSTIVRLMYRFYDPTGGRVLVNGNDIRDVSLRRQIAVVPQDSVLFNETIEYNIHYGNFDEQVNEASSMAELHDTILRWYDTQVGERGLKLSGGEKQRVAIARAILKNSPILVFDEATSSLDSITEAKIMTALRAVENRTSVRIAHRLGTVADADTILVLEHGKIVERGHNELIGTLYAHLWHQQQNFNVPETQITELDNGMRVATEDSKLETCTVGVWIDAGSRYETPKTNGVAHFLEHMAFKGTSKRSQTELELEIENMGAHVNAYTSREQTVYYAKCLSKDVDKAVEILADILLNSKLGEAEIERERGVILREMQEVEQNLQEVVFDHLHTTAFQGTPLGMTILGPTENINSITRNDLLEYIQTHYKAPRMVLAGAGGIDHKKLVEL----------------------------------------------------------------------------------------------------------------------------------------------------------------------------------------------------------------------------------------------------------------------------------------------------------------------KPTWHAPWKLYRVISGNSGWVHSLAVEPGNEWFASGSRDGLIKIWDLASGRLRLTLTGHISSVRGLAVSDRQPYLFSGADDKMVKCWDLEQNKVVRHYHGHLSGVYTLALHPTLNILVTGGRDSVARVWDMRTKAQIHCFTGHSNTVATVKCQSSEPQIISGSHDTTIRLWDLREARTHVTLTNHKKSVRSIAIHPEQNMFASGATDNIKEWSFPDGTFVQNLSGHESLINSLAINQDNVLVSAADNGSMFFWDWRTGYNFQRLLSRPQPGSIESEAGIFAVTFDQSGSRIITGETDKSIKIYQVEQRLTKHVKKLNARLNVVTGSTSDYRIDVNKQDYLEKLGVNVKARNFLVYQGAVESIAMKNPREITSLFEEISHSMDRSEEELFVKKKGITAEKKEAQGEEAKRYQNTRNEYQIDLQLFKLYHIDKEMARRRKKTAIDDDIKAKRQIDKNIRDELNLNKKRPSYIKAKENASHIEKKLESARKSLAAAQKAHANHEDIKELEQDLERVNKKFLVEDVELQEEQKTEYNRLKKQAASMSAKYLKDLDSLEREQKADSDRHENEVGKQRELDHKLREREENLRRIEKLKDLIDFKQREREIIAKKELSQELNGISNLGDAKVDRHEEDRRRKKSEIVEHLKKIYPGVYDRLLNLCKPIHKRYNIAITKIMGKSMEAIVVDTEKTGRSCIQYLKDQMLEPETFLPLDYIHSKPIKERLRNIQSPPNVKLVFDVIKFEPQDVREAVLFATNNALVCETASDANKVAFELGDNQRYDAVSLDGTFYQKCGFISGGSLELERRARRWDEKEIHNMKHKKEKLAEDLKENVKKTRKEGDLMVITSQITGFESRLRYSKIAEIKDRMDAREVEINKIRSAMNRVEDDIFKDFCIQLGVDNIRQYEERQSKASQERERLLQYESEKNSIQSRLLYER--SKDTLEVEVEQHNLEEAKEAERREMKAIEEEMSKVEQLKNDKISQKTDYDKIEEQVTEKKRSLTTIQKEILAIQKNITNLECRMESKRTDRHAVFVHCKMESINLPLKQGSLQRAHQSDNIRPNFDLLELLAEIAFRKIQAPNMRADERLDSAKERLRETDSELNSLRKQAKEAFESVKQERECFDTVSQRVDSIYKQLTNNPSAQAFLVPENPEEPYLEGINYNCVAPGKRFQPMSNLSGGEKTVAALALLFAIHSYKPAPFFVLDEIDAALDNTNISKVARFIRQRTESAFQCIVISLKEEFYGHGDALIGVAPDPGDCTISRIYAVNLG-----------------------------------------------------------------------------------------------------SLHIWNVHSVLNVLHSLIDKSNVIDQLREYNAGRDPQAVAGVFGSHNIYKMLGYYSMIGLLRLHSLLGDYHQAIKVLENIDLNRQIMVRVLACQTATYYYVGFAYMIMKRYSDAIRTFTNMLAYLHRTKRTFQVELVDKQTDQMYVLLAMCLVLHPQRVDEGVTVVLREKYMARLQRGDINEFETCPKFLSPVQLKVFLEEVTQQLIIRSYLKLYKTMSIEKLANFLLWCFKHKKQSIVLDGELRSGSDVDFYIDKNMIHIADTKVARRYGDYFLRQVHKFDELYRMGPPDPILGVTEAFKRDTNPKKINLGVGAYRDDNGKPFVLPSVKVAEERIFKAGLDHEYLPITGNANFCQAAATLAFGNDSHIITNKLNATVQGLSGTGSLTVGAAFLRDFHNYSKEVYMPAPTWGNHIPLFKRNGFNVKQYRYYDPKTCGFDFVGAMEDLNNMPEKSIVLLHACAHNPSGVDPKAEQWQEISHIIKKKKLFPFFDMAYQGFASGDIDRDAHALRMFIRDGHMVALAQSFAKNMGLYGQRIGAFTLTAKDQQEAERILSQLKIIIRPMYSNPPLHGSRIVETVLTDTNLRQQWLKDVKLMADRIISMRHALRDGLVSEGSTRDWSHVTETIGMFCYTGMNAEQVGRLWNEFSVYLTKDGRVSIAGITSKNVSYLAHSIHSVTK--------------------------------------------FLYVIRLFMLLATPQCLFNFLGLISFNPFPGKVTLKHDDKTRPFICIRVVTRGLFADLIRNNVRRNLQTCIDSGLDNFVIEVVSDRDITLNDSQNVRLLVVPKDYRTSTGALYKARALQYALEDKVSQLNDGDYIVHLDEETLLTENVIYGILNFAKEGKYDSGQGLITYANEEVVNLITTLADCFRVADDLGKIRFQFRAFHRPLFGWKGSFIVNKYTVEKDVSFDHGPDGSIAEDCYFSMVAYKKGYKFQFIEGEMWEKSPFTVADLIKQRTRWLQGIFLVVHSRKIPIVNKFFLAVSLYAWMSVPLITCTLILGPIYPMPEIIWLNSITAFNGVVTMGMFLFGVMISFRIKRVGVSRLFVYLVGTIVILPLYVCIENIVVIWGLFSPKHKFYPWPNYVYTGDLRPYPRSALRTVPPNIQRPDYADNPEGRAISEEAIKDSTQIKCLNDEEIESMRVVCKLAREVLDEAAKVAVVGVTTDEIDRVVHEASIERDCYPSPLNYYKFPKSCCTSVNEVICHGIPDMRPLQDGDIVNVDITVYHKGFHGDLSETLLVGNVAEQYRKLVQVTYECLQKGIEIVKPGVKYREIGDVIQKHASQAGYSVVKSYCGHGINRLFHTAPKVPHYAKNKAIGIIKPGHTFTIEPMISEQSWRDTQWPDQWTAVTVDGKRSAQFEETLLATETGCEILTRRRSNGGQPYFMDYMTLLKMMKGQSKKRRHLLAQQLGISSGDNLSAILGTRVERTTT-------EQTYRYTGSSTFLKGTQSANPHNDYCQHYVDTGQRPQNFIRDIGLHDRFEEYPKLKELIRLKDELIARTATPPMYLKCDLRQFDFRELECKFDVILIEPPLEEYQRTQGVTNTDFWSWDEIMKLRIEEVAAPRSFIFLWCGSSDGLDLGRQCLRHWGFRRCEDICWIKTNATNGHTKNLEPRAIFQRTKEHCLMGIKGTVRRSTDVDFIHANVDIDLIISEEPKYGTCEKPEEIFHIIEHFCLGRRRLHVFSRDTILRPGWLSIGPDLTNSNFKAKLYNSYFNSPQDCLTGCTDRIEALRPKSPPPKSKRILIRVDFNVPMKDGRITNNQRIVGALQTIKFCLENKAKSVVLMSHLGRPDGQVKPEYSLRPVADEVSRLLHRNVTFLCDCVGLDIEEVCKDPQDGSVILLENLRFHVEEEGKGVDEHGNKIKADPKSVEKFRKSLTSLGDIYINDAFGTAHRAHSSMVGINLPHKAAGFLMKAELDYFAKALNNPPRPFVAILGGAKVKDKIQLINNLLDRVNEMIIVGGMAFTFLKVLKGMDIGSSLFDSDGAAIVKDLMDKAAKMNVKMHLPVDFVTGDAFKEDAKVGEATVESGIPDGHMGLDCGKKSMQLFEEPLKRANIILWNGPCGVFEWDAFSHGTKAVMDMVVAATQRGAITIIGGGDTATCAAKFQTESKVSHVSTGGGASLELLEGKELPESKISVRMMRTFGDRPTAFQLEEGGEYFYIGSEVGNYMRMFRGSLYKKYPSLWRRMVSVEERKKISSLGLGAHTIATNVTLLKATEVDEIFAGKDERYKAISISSEPSASRVDRDKRINWAASLPTSSHHLDAVPCSTAIARNRLTHKRVRTFPMIYDDLDPKTLTQIANVTEVLVPIRLDMEIEGHKLRDTFVWNKNEISITPEQFAEILCDDLDYPPQAFVPAIAQSIRQQIEAFPTESILDQQTDQRVLIKLNIHVGNISLVDQFEWDLSEKNCTPEQFALKLCAELGLGGEFVTAIAYSIRGQLSWHQKTYAFSEAPLPALDFPFRPNNDADQYAPFLETLTDAEMEKKIRDQDRNTR----------MGSNASQLEREIGFPQNEHYFGLINFGNTCYCNSVLQALYFCQPFREKVLEYKLKSKRTKETLLTCLADLFHTIANQKKKTGSYAPKKFINRLRKENEVFDNYMQQDAHEFLNYLLNTIADLLRGEKGWVHEIFQGTLTNETRCLNCESMSSKDEDFIDLSVDVEQNTSLTHCLRVFSKTETLGAEHKYYCEKCCSKQEAQKCMRIKKPPMILALHLKRFKYMESQNRHTKLTSRVVFPLELRLFNKTSNSDSDDRLYDLVSVVIHCGFGPNRGHYISIVKSFGFWLLFDDDYVDKIDASSLEDFYGLTNDTQKTSESAYILFYQSRD??????????????????????????????????????????????????????????????????????????????????????????????????????????????????????????????????????????????????????????????????????????????????????????????????????????????????????????????????????????????????????????????????????????????????????????????????????????????-----------------------------------------------------------------------------------------------LPLLPELDVYFNLLVVVNLIDLGRYEIAVRCSNQLMGMIVSQNRRTLDAMAARCYYYHTRCYELIGQLNQIKSFLHSRLRTATLRNDYEGQAVLLNCLLRLYLNCNLYDQAAKLVSKSVFPEAASNNEWARFLYYLGSIKALQLEYSEAHKNLLQAIRKAPQTGAIGFKQTVHKLAIIVELLMGEIPDRSLFREPTLRRSLAPYFQLTQAVRSGDLTRFGVVIDRYGSKFQTDSTFPLIIRLRHNVIKTGVRMINLSYSRIHLADIAKKLKSDFTEDAVFIAGKAIRDGVIEAKINHENGYLQSKETTDVYCTPEPQAAFHQRIAFCLDIYNQSVKAMRFPLKSYNVDPESAEER-------------------??????????????????????????????????????????????????????????????????????????????????????????????????????????????????????????????????????????????????????????????????????????????????????????????????????????????????????????????????????????????????????????????????????????????????????????????????????????????????????????????????????????????????????????????????????????????????????????????????????????????????????????????????????????????????????????????????????????????????????????????????????????????????????????????????????????????????????????????????????????????????????????????????????????????????????????????????????????????????RQHCRW-ATLENVELCIPLVFKAQLFALTGVPTDRQKLMFKGSVIKDDAWGCMFMLMGSNEKLPEPVVKPKFVEDMSEHELATALDMPSGLTNLGNTCYMSATVQCLKTVPALRELRRYQGNVTASLRDLYTSMDSS-VEPVILLSVLQSAIPRFAQKAEGGQQDANECWTELTRMLQNFIDEYFGGTFVEMKCDEAPEESNENFLQLSCFIS-QDIKYMQSGLISRM-KETLTKSSPTLGRDASYKTSRISRLPAYLTVQFVRFFYKGNVQTNAKMLRDVKFSMNLDVYELCTPALQEKLAPMRQKYKEADDHYSFVDDGSNNSGFYELKAVLTHKGRSSSSGHYVAWIRRQWFQCDDDRVVDEEEILKLSGGGDWHCAYVLLYGPKLI---INQVLPRELILKIFSYMDIVTLCRCAQVSKYWNQLALDGDNWQDVSLFDFRAGVKGRVVEYLSSRCGNFLKRLTLRGCRSVTDSSIDIFANNCRNLEEILDDCKQLTDRSCLSLANSLNIASC-EVTDESLIALGTNCKNLQHIDISGCNKITGAGIRALADGCPKLRSFISIACVNNESLQYLASQLRTINLNACSSITDEAVIALSENCDDIVNCCLSKCTNIADQSLIALSQHCLKTLGLIGCNLLTDAGFQALTRGCKYLENLDLEGCVQITDQTLYYLTLNCLKLKRLVLSYCEFITDEGIKHLGASFLELDNCPQLSDVAIGHLANCLKRLDIYDNQMITRQAI-----------------------------GSRGATVCCPRHPTANLIEDHRAGDMICPECGLVVGDRVVDVGSEWRTFSNERGNTDPSRVGAAENPLMGGNDLSTMIGRTGSAGDEVGNPRYSNRRTMSSSDRTLQTAYRDISNMADRLGMPRMLVDRASETFKKVHESKALRGRSNQAIAAASLYIACRQEGVPRTLAEIRSASDVLKRDVGRCFKLIMGLLGTSVDMISTSDYMSRFCSNLGLPTYVQRAACIISERATQRDINAGRSPVSLAAAAIYMASQASDVKKSQKEISDVAGVAEQTIRQAYKAMFPRAGELFPDDFRPHSKMAWVEKFRPKEFKDIIGNKEAISRLEVFSREGNLPNIILSGPPGCGKTTTMLCLARKILGDQMKDAVLELNASNDRGLEVVRNKIKMFAQTKVTLPAGKQKLIILDEADSMTEGAQQALRRIIELYSKTTRFAFACNTFDKMIEPIQSRCAVVRFTRIPDNEIRSKIIEICKMMGVKYDGSGIDALLHTAQGDMRQAINNLQSTYDGFGEVTSDNVFKVCDEPPPIMIKDIINHCLQGKLREAEESLVLLYKRGYCSEDLISSIFRVVKAYKTEEAFIIELFIHYGNTLLTILTVVQSVAVFAIFSTRLPKKILNLMTPKREHTLTKPYQGAGMTIPNWEFFGSTIVTNNYIRLTQDAQSRQGGIWNSMPWQVEVGFRIHGQGTELYGDGFAIWYVRDPPQVGPVFGNQDFTGMGIFFDTYANQQGVHSHGHPYISGMINNGTMHYDHDMDGTHSELAGCECKFRGSDHETRALITYYNDEITIKIDVEGDNEWHDCFKVTGVYLPPYYYFGITAATGDLSDNHDILYVKAAMLSAPKDDDLPKAAPPSERLESGMPALRRFFMIVCIGAAIIAFLYTSQLRNTRKRLYGNQDNFDDIMEKNCNVLDRNSLFLPESSVSHIPDIKQLGIDPTYENRTALLHIHNTALGRAIFFSYLLQKAFDAEAGLMYYYLSTNADVAASRYANASAIYFSPNRAFTPSYNGFFNKTMPLFAPRAFRADDFNDPYQLRGTSTLNSVIEHDHGAIDSNYTTEFYKINEWYGTWLPDMTRRHDSKPTYSVQITGHNETFTFHGPAGASDTPGPVRWTRPYYDCGRSNKWLVAAHTPIADLYPRHTGWRHIELPLYVAVAVVEIDYERLDINQCPLSDGN-PSPNHFHDTAKCVKDTTMCEPISGYGLRRGGYQCRCRPGYRLPRHVKSPYLGEIVERATDDDRRNSFKCEKIDNLAVKTQNVQTLTGLSSRMDASLPGSVAHGKEHQFEIQSRAALRLSHFISSFIQLVDPKEVFAEFRVPDRALTKDQVIGEALSTMMADRSIQGLGVWFDRNQFASP----------------------------------------------------------------------------------------------------------------------------------------------------------------------LKAKYGGRFMVTMLPGDGIGPEMMRHVKTIFKVGGMPVDFEEINLDSMNENIEKVEEAITSIKRNGVAIKGNIETREHSKYFKSRNVELRTRLNLYANVVHIRSQPGVATRHHDLDLVLIRQNTEGEYSSIEHETVTGVVECLKVVTRAKSEQIAHYAFNFAIKHGRKKVTCVHKANIMKLSDGLFLDVARSVAKSYPEIEFEDIIIDNCSMQLVSNPHQFDVLLLPNLYGAVLTNIACGLVGGPGLISGANYGDEYAVFETGSRNTGKTIAGKNIANPIAVFNASADLLQHLKLENYASLMRGAVHKALNESKVHTPDLHGIHTTTDVVNLIIDEIRQQT------MDEYQLNNPPTDTIQSVKFSPHNGRHLLVASWDSAVRLYDVESNIVKARYNHSEPVLDIAYEGSEAFWSGSVDKTVKRYDISTQTATTVGSHTDAVRCVEHIPDLNLMVSGGWDSQLKFWDPRQGKGAALSSQVDKVHTLDVAGHRVLVGTASRRVIIWDARTHKFVQ--RESPLKYQTRCIRAFPDGQGYVLSSIEGRVAVEYLDPDPEVQKQKYAFKCHRNKDEGEMVVIYPVNAVSFHRKHGTFATGGSDGYVNIWDAQRKKRLVQFRKYPTTISSLSFSCDGTMIAIASSYLYENEEINEIPPDAIYIRRVSLEQLRKLSVVVADTGDFELMKKYKPTDATTNPSLILQAAQKEEYSGIIDKALEYGRQNGRSLEATMDYLCVLFGCEILKIIPGRVSTEVDARLSFNTQGSVNKALKLIQLYKDHGVDKERVLIKLASTWEGIQAAKILEQQHGIHCNMTLLFSMAQAIACAEVGATLISPFVGRIYDYY----KVKSYEPFDDPGVKSVTKIYNYYKRHDYKTVVMGASFRNISEIKCLAGCDLLTISPALLGELAEKGVTLNKELDVAKAKSLDLEKRTYDAARFAWEMNEDEMAHFKLAEGIRKFAQDQTRLETIIKAKL-MPTACVRPLVTVYTDKNQKSGKTVKMPAVFSAPIRTDIVQYVHTNIRKNSRQPYSVSKLAGHQTSAESWGTGRAVARIPRVRGGGTHRSGQGAYGNMCRGGRRFAPTRVWRRWHRKVNRNQRRYAVVSAVAATGVPSIVQSRGHRVENIAEFPFVVSDKVQELNKTKQAVKFLRQVRAWKDIERVYKSKRMRAGKGKMRGRRRVKGLGPVIIYSRDSGLTRAFRNIPGVETINVEKLSLLRLAPGGQIGRFCIWTEGAFRKLDRIFGTWTKKSMLKRDYMLPQPTMANTDIARILKSDEIKKAIRPRRSRLPKRCIKRNPLKNIRVMARLNPYAPVLKKVRKIETAHRKAKKIEKAKKLHKAEARKKDKKKQRIVKKDMLKNGQIIKKILDAVKDLVTEATWECSPNGLSLQAMDTSHVSLVTAQLKSEGFDHYRCDKPLNLGMNLPHLTKIIKCAGNDDTITIKAIDECDKITLLFEDKNETETSEYEMKLINLDSEYLGIPEQDSEVEVEMPSSKFARICKDLSSMGEAVTISCVKESVRFQVQGDLGSGSVNLVQKSSADKPDEAVCIKMVNPICLSFSLKYLNHFSKAAPLSPRVKLLLKAEAPLVVSFEIGFIRYYLAPKIEDVQSLVSKVAVLGACGGIGQPLSLLLKTSPMISHLALYDVANTAGVAADLSHINTKAKVSAHQGPAQLADCVKDASVVVIPAGVPRKPGMTRDDLFNTNASIVKDLCKVCAQIAPKAMLAIISNPVNSTLPIASEVYKKMGVYDPLRLFGVTTLDIVRANAFVAELKQLDPNQVNVPVVGGHAGTTIIPLLSQTTPSMQISKEQREPLIKRIQDAGTEVVKAKAGTGSATLSMAFAGARFTFSLLKAMAGENVVECSYVKSDVVGLSYFSTPIVLGKNGQEKNLGLGQINDFEKKMVEDAKAELKASIAKGEEFASK????????????????????????????????????????????????????????????????????????????????????????????????????????????????????????????????????????????????????????????????????????????????????????????????????????????????????????IPTFIGVLNELKPSLNDIKKAVDVCDVVFTEVEPSL--NSVVT-LLFTFCENVLKAPSESSCLR--VMKNLYDGLQYVAYITLVKLANNQLSEVFDSLASVKSKFGVESTQNLYRLLHSSLSKLASQVMIELLGTYTEENASHAKQDAMTCITSFLKDPNTFLMDHLLTLKPVKFLEGEPIHDLLTIFVSEKLQDYLEFYSKNIQVI-ENLGLVHEQNMKKMRLLTFMQMAESKKEISYETICEELQLEHDNVEPFVIDVLRTKLVRAKVDQLGRKVLVQSTMHRTFGRPQWQQLRDILNFTHVEQTIKTFLEYIGSTR-KKKTVEEVQEGKKAIDDNESRIIIDQSPQFKELVTTLANWINDELNDQRIIVSVKHLEHDLYDGQVLGKLVEKLTGTQLDVVEVTQNEDSQRRKLREVLETVNRIFNIKWSVEGIHAKNTVQIIHLLVTMIRHFRAPI--RLPPNVTVRCNVLSKQGEKLTETYNELGMRIEPRDAFDTLFDHAPEKLDVVKRSLCNFVNRHLKKINVEDLDPNQFSDGLLLIFLMASLENYFVPLGIFTTP-TDPIADMNGAQTALQHENYVNTSKLHNVNVALQLMEDADVKQRVRAEDIVNADLKSTLRVLYMIFSKMSHRSKEILTTTEKNLRALMKIPDNYTVLFLQGGAQAQFDSVPMNLCSAEYLVTGIWSSKASKEAQKYVNYERLPTEEEMPLCGCACYRYYCDNETIQGVEFPLVCDMTSNFLSRPVDVSKFGIIFASAQKNCGTSGLAIVIVRDDLIGKMSVTPSTQNYQIMRDNKSNYNTPPVYAIYLALCVKWILDKGGLGAMKISCEKSSLLYHTIDESKGFYSRSRMNVVFLI-GGSKGNAELEKKFLAEAEFHDLKGHRLVGGLRASLYHGVTLDDSK???????????????????????????????????????????????????????????????????????????????????????????????????????????????????????????????????????????????????????????????????????????????????????????????????????????????????????????????????ALVVDRFDELLKFVPYISEFLHKQGITHKIYVMNQVDNLRFNRGALINAGFLVHAGQSDYIVMHDVDILPVNNNLSYAYPENGPFHIASPEYHPQYNYARYIGGILSISNEHFRLVNGFSNRYYGWGLEDDEFFTRLTSKDLRIARPTRLQTNKTNTFIHMHR---RPRDKSRLFNQKEATSKRDRQNEWHVLPHKDDFLYPQSVQSIAFAVSSAFFTGQLFETFWCFQSVALPGDLIIENGYMRGHGELVSSVAGRVERINKLVSVHAPRARFVGETGDVVVGRIIEVQVSQKRWKVETKSRLDSILLLASINLPGGELRRKTAEDELMMRKYFKEGDLIVAEVQSIFQDGALSLHTRSLKYGRVGQGILIKVPPSLIERRKLFHNLNTLGVHLILANNGFVWISEHGFDRRAVARMRNCIEILCFAKIMINDKSCTVCFEES-IVTLSHECNPKHVGVWLLGVSGITAFTVSLGGITRLTKSGLSMVDWHPVLESPPRTTDWNAEFDKYKQYPEYQMRNFDISLDEFKRIWWMEYIHRTFGRVIGISFFVPAAYFAYKGYFKGRTRYVIVALGGLILFQGGLGWYMVKSGLEQPPRVSHLRLASHLGTAFTFFSISFLTALHYLLPNVQLLRYKKLTGSVFVTALSGALVAGLGAGLTYNSYPKMADRWIPTDLFAKPVWRNFLENSTTVQFDHRWLGQITASGCWLWSLRLPLTPRLRVVSNVVMMLCCQLSLGIATLLLYVPKHLAATHQAGALGLLTLSLWLAHELKYVKK-V????????????????????????????????????????????????????????????????????????????????????????????????????????????????????????????????????????????????????????????????????????????????????????????????????????????????????????????????????????????????????????????????????????????????????????????????-----------------------------------------------------------------------------------------------------------------------------------------------------------------------------------------------------------------------------------------------------------------------------------------------------------------------------------------------------MRRRCLSLRKVIVKNNAKTGEVLLDETLRHISTTEPPLSVQAWIEYLSGETWNPLKLKYQLKNVRERLAKNLVEKKVLTTDKTNWVIFDMTTHPLVNSTIKNKLIRKVQDALLRNWINIPQRMDKRILSLLLLAHYSDVLENAFGPLSDEEYETVVNRLNTLTSLDMEAESQKPDTNESIWAVFSYFNKMQPPIAKYKCVLVGDGGTGKTTFVKRHKTGEFEKKYIATLGVEVHPLVFFTRYGPIEFSVWDTAGQEKFGGLRDGYYIQSQAAIIMFDVTSRVTYKNVPNWHRDLVRVCDNIPIVLCGNKVDVKDRKVKAKAIVFHRKKNLQYYDISAKSNYNFEKPFLYLARKLMGDASLEFVAMPALQPPEITMDPQWTARLEQEMQQAQDVPLPDDDDEDLVAEYFTPVLRSSKFKQTGVLTPEEFVAAGDHLIHHCPTWTWSSSSEKSYLPSDKQYLVTKSVPCYKRCKDIDEKVIMTDEEGWVDTFDYDDDDDEAMDMDKFLEDHDDAVNIIATRVYDLNITYDKYYQTPRLWLVGYDEHHKPLTIEKMYEDISQDHRKKTVTMENHPHIGR-VMASVHPCRHAEVMKRLISTVEDGGGLQVHQYLIVFLKFVQSVIPTIEYDYTQNFTIISRYDPVMPYGKLQNNLAQVRKKLDRPLTLAEKILCPHLDDIKNQDIKRGESYLRLRPDRVAMQDATAQMAILQFISSGLPKVCVPSSIHCDHLIEAKSGASSDLSKAKEVNKEVYEFLRTAAAKYGIGFWHPGSGIIHQIIFENYAFPGCLIIGTDSHTPNGGGLGGLCIGVGGADAVDVMANLPWEVKCPNVIGVHLTGQLNGWASFKDVILKVAGILTVKGGTGAIIEYFGPGVDHISCTGMGTICNMGAEIGATTSVFPYNGMMRDYLVATGRQAIADMADENLSLLTPDKNAKYDQVIEINLSTLEPHINGPFTPDLATPISKLAEAAKKNNWPMDIKVGLIGSCTNSSYGDMNRSVAVAKQAIEHGLKTKSAFTVTPGSEQIRATIERDGQADTLRKFGAMVLANACGPCIGQWNRQDTKKGEVNTIVTSYNRNFTGRNDANPKTHAFITSPEMVTALSIVGRLDFNPLTDHLTGAKGEKFKLEPPTGEELPSRGFDPGQDTYQPPAEDGSNVTVNVDPASNRLQLLQPFDKWDGRDLTNMAILIKVKGKCTTDHISMAGPWLKFRGHLDNISNNMFIGAVNAENGEVNKVRNQLTKEFGSVPDTARYYKSQGIKWVAIGEENYGEGQ------------------------------------------------------------------------MFRNTFQSGFLSILYSIGSQPLQLYDTKVRNGSIKRITDDEIKSLVIEITSSNVSTTFITCPKGLERNLGIKLPIFVMIMKGLNRYFTFEIEILDDTKTKRRFRASNFQSETKVRDNICTMPLRLEHGWNQITLNLPDYCRRAYCTNYVETVRLTVNANCKLRRIYFCDKVYPEEELPAEFKLCLPVTRLLQHAKYRQIEHTRKDILNAIKYYRTLLEKYTFPDGVSKELICLDGTIPVTFRYNIPVGIWLSDSHPFVAPICYVRPTRDMTIKQSRHVDGSGRIYLPYLSDWNCAT-SDILSTIQVMQIVFGQTPPVYQT-QRPLP--NTGTITEEHIRASLLSAVGDKVKARLREQIQAEIEVLKKTSNDLNRGKMQLDEMETRMAKEVDLNDSLDAQLQEIDVDNIYGPTQPLYRQLLNAFAEENAVVDAIYYTGEGLRKGVISLDVFLKNVRELSRRQFMLRALMQACRAKANLP-----------------------------------------------------------------------------------------------------------------------------------------------------------------------------------------------------------------------------------------------------MASRLSQKKKVVASGMFKAEIDEFLRRELAEDGYSGVEIRKNASRTEIIISATRTQQVLGDKSRRIRELTSVVSKRFNCEDGTVEMYAEKVNDRGLCAVAQCESLRYKLVGGLAVRRACYGVLRYIIESGAKGCEVVVSGKLRGQRAKSMKFVDGYMIHSGEPTNHYVDTAVRHVLLRQGVLGIKVKIMLPNDPTGKQGPKKPLPDTIHVIEPKEEVMPIVPSASNVDGMVSPMDSLKEWSVSTFKCTKQRLEEKLGKCSRTFDGPLEAEIERLRENQRKYTMMLNTARSMSIQYSLVHTQRQLCELMNEMALKYTANLSEDFRKNALVLSIAAKNGEKLCQALSFFCANLSTLVHKTIEDTLFTIRAFEAARLEYDAERN--SDKLKSRYEQLREDVLIKIRFLDENKFKVMHKQLVLFNSAFASY-TSGNTAALEAALKQFKI-----MSDEATATADIRLWVFLPIVIITFLIGVLRNYVTILLMSTKKLELQQVQDSQALIRARLLRENGKYLPKNSYLMRRHFFNS-EHGYLTVAKNRPSVQPNPMTDPSMMTDMLKGNLTNMLPTILIGGWINWTFSGFVTTRVPFPLTLRFKPMLQRGIELASLNSSWVSSASWYFLNVFGLRGIYTLVLGENNSADTTRMMQDQMSGAALSMPADPKPAFKSEWEMLEIHEHHFAL-----MNSSTSGPSSSRRCKSRDAKRRRA-SQTETLKKKIEEETKVADIGGRFATHHDAIEAHLRASTVGLVTLDEMRIRQEEAAKERAQQLVQLQEEKNARPSVDPKELQEKHRRQRQALSFQFDDKKMLKNPDVDTSFLPDREREEEERRLREELRLQWQERQEKLKEEEIEITFSYWDGSGHRRVVKMKKGHSIYQFLQKCLEQLRREFNELRSISADQLMYVKEDLIIPHRYTFYDFIVTKARGKSGPLFSFDVHDDVRLKTDATVEKDESHAGKVLLRSWYERNKHIFPASRWEAYDPTKTYDKYTISDKKGRVPTVPESVLKKRQLRKTIKEAQRVKAQKRQKERRVKHTKIFKRAEKYVKEYRLRERDQIRLKRQAKKHGNFYVPPEPKLAFVIRIKGINGVSPKPRKVLQLLRLRQINNGVFVKLNKATVNMLRIAEPFITWGYPSLKMVRELVYKRGFGRLNGQRVPLTSNEYIEKRLKRYDIICMEDLIHEIYTVGPHFKQANNFLWHFKLNNPRHGWNKKTTHYVEGGDAGNREDKINDLLKKMVCRGSAEQLWKAVTGVSNAGKKRGRGRGAGRRQVKDFNRGQMIGVGRKTLIMPGLNAPVVKAKQMVEPQLVGENTEFRDNLSKMRNEMNIFKKYRQHPMERGWSGNKAAGKHMGPPDPVAGTPFENFESTVLMLRPLVMMRGPLGRTKEMNALVVTGNGNGLAGFSTSTAKDGRAAVRNARNRAGQALVYIERGDGTVLHDFFSRYYYTTIFVERKPKGYGIKAHRVIKAICEAFGITDLYAKCEGASDNTINITKAFFLGLMNQRPYQDMADEKKLHLVELREENNYYPRILASPRDARTERDIAPGEMLDFTYFIYNGKIRQVTRKNPPLYVGSAGWQVHLNRMDFVKNREKTKLALAAKYDDFEVFPGFKAIKRPEQA-----QSRMAPKTRVLVLCSRGVTHQDRHLMLDMRKLIPHSKKECKFD--KKDLNELSIIDNCDKCIYFENRKGKLLFMWASNVTGGPTVKFLVQGVHTMSDLKFVGNCLKGSRPILTFDSNFDKLPLIKELFIQIFNVPFKHPKSQPFVDRVVTFSYLDNHIWFRNYQIIDEAEISLAEIGPR--------------------------------------------------------????????????????????????????????????????????????????????????????????????????????????????????????????????????????????????????????????????????????????????????????????????????????????????????????????????????????????????????????????????????????????????????????????VIVGILGGTGLDQDSSILSDETPYGRASDTQAISGVIEGVKVYILARHGKSHDRSPSHVNYRANLWTLVQQLNCTHILVTSACGSLQEHIEPGHAILDQYIDRTRDRSFFVCHIAQGRPIMRDILIEHKDLCAVTIEGPRFSTLAESLLHKSWGCHVVNMTSVPEVQLAAELGVFYGCLLLITDYDCWK-EGEECVSAEMVDRMKSLRSTAVKIIPRAIKKIVKEAMMMTVLRHEVFDSGCEAACNGPYDNKWSKTMIGYGPEDDYFVLELTYNYSVGKYNLGNDINYLKIKAADLFNKIKESEE-SQESDETLELVSPDGYKFIVEQLEE-GKKTDVTQVCLSCIDLNKSKNYWINLLKCELYSESERELVFGYSDKQTSLKLIKIDSELNHATAYGRIAFSCPASELKSIEALVADNKHTILTPYISLDTPGKASVEVVILADPDGHEICFVGDEGFRELSKVDPDANKLIDQSIADDKSAEWFAKKKKFKKDA????????????????????????????????????????????????????????????????????????????????????????????????????????????????????????????????????????????????????????????????????????????????????????????????????????????????????????????????????????????????????????????????????????????????????GTIPIVIEQTGRGERAYDIYSRLLKERIICVMGPIDDHLSSLVVAQLLFLQSESSKKPIHMYINSPGGLVTAGLGIYDTMQYILPPVATWCVGQACSMASLLLAAGTPNMRHSLPNSRIMIHQPSGAASGQATDIQIHAEEILYLKKRLNGIYEKHTKQPLEKIEAFMERDKFMNTEQAKEFGLIDVIVERPP????????????????????????????????????????????????????????????????????????????????????????????????????????????????????????????????????????????????????????????????????????????????????????????????????????????????????????????????????FFRIVKDLKHGTSITLLPSEPALVEGDKTLYIRGYTGNKLTTNVMKDIYSLQKPLSSFLNRKNPFDDASTIEYLQKNESPLFMFSAHSKKRPNNLVMGRTFGGQILDMIELGIDSYKSISEFKNLKISLGSKPVLVFAGEAFEDMIRIKNLLNDFFCGTSIRVAGIEHTIQFIAHGIHMRVYLVEQKKTGSSSKSIVELTEMGPRLELSVRRTRIASFDHFKLACKQQPKVKSKKTRNVSKDAFGATRGKLHMQRQALSLHHLEVVSLSIHPIKALQDNYMYLLVSREAAAVDPVNASAMTDAIQADLKAILTTHHHYDHANGNAELLVYGGDARVQALTKHGD-KIKVGKIITCLSTPCHTKGHICYYVVFTGDTLFIAGCGRFFEGTADQMNRNLLGNLPDETLVYCGHEYTVNNLKFAIHVEPRNNIKRKLDWAEREPTVPSTIGEEKKINPFMRLTVKRFTDENEVMSVLRVKKNQFHMNRLFGRSKPTAPGPSILDVIQSVDQRVEQFDKKIAMLDKELLKYREQMNKMRDGPGKQSVQQKAIRVLKQKKMYESQRENLIQQSFNMEQTNFATQMLKETKTTVDAMRAGVKQMKQEYKNVNIDDIESVQDDLEEMLADANEVQEVLGRTYGVPDVDESELEAELEALGEELQQDDTSFLDL-GTISGVRAKHSLPDLPYDYNALEPTISAEIMKLHHGKHHAAYVNNLNIAEEKLSEAMQKNNVSQIIELQKAIVFNGGGHVNHSIFWKNLSPSGGGDPSGALLNAINSSFASVDDMRQRVSSTAVALQGAGWAWLAYCPQTKKLTVRGMPNQDPLHPLTGLVPLFGIDVWEHAYYLQYKNARPDYVKAIWNIVNWKDVEERFIQAQEAPIKQDLPSDIPSLKQELETRRQNIEKKLKHVEHACIADYMKEGNNISDLHQRVICCDQILERLETMLCKFQADLGNICQDILSLQDQSVSLSTQLQNKRAVREKLGQFIDDMTIPQPVINHIMNAPTNEALFMEHLQILDQKINFFKEQDFRDAKACNDIHETLMSLKSRAVSKVREYILKKIQGCKKCLSNYQIPQNSLLKNKFFYRFLLTHEREKAREIQAEYVDTMSKVYFSYFKEYLARISKLDYDDKPDEHDLMGGDDQSNLTLFSKTSIKNRSTVFSMGQRASVINTDLEAPLILPTMQQKSDTKYPSECLFRSIQYAIVDNACREYQFLCEFFMVSDGQAADLFHSVFGKTLAIVHAYVVEQFKMSYDTIAMFLCLHVIYRYRRLALKQNVPVLESYWDSLVKCLWPRFDKVFRMHIDSVKNCDPQRIGPVDTLPHPVTRRYAEYACSMAAVNNTFPDERVMFLLSSLQNEVKNFILRTAAIFVHPKEQLIFMINNYDHILSVFKRAVKEDSKDIEDIKLLISKRTQEIVEELLYPHFGSIICFVKDSEVYLERNDQESLKRNEPKVTTLVKSFNSDWRKALDEISHEIMGSFSNFENGNNIQQAVLTQIIQYYHRLQKIVALSPFKNNPVRNELIDTHQLMVDLKKYKTNFALMQTIDAHAEECFFERAEAGTKLGFTFEVIDGGFLDIDIHIRDPEGRILHQEERASSGKYTIEATTTGAYDYCFSNKMSTMTPKVVMFSIEKSDGTH---SKPGDAEHAKLQSMVQTLVYSGVSVKRELEYMAVRDRIHRRINEETNSRVLTWSLFEFVLFLTASVAQVVYLKRFFEVKRVIMRLTQLLALPPSYHNMPQRYVDRNTKFIEYRAPKLPHYVRKVIKYRYDIWRPWQREFQENRAGSKIYVEPIFRGDRVELMTGPDKGKQGYINYIVKQRNWVCVEGLNLEEKPLLINHEVKLVDPTDLLPTDIEWRFDDQGKRVRVSSRTQRVIPIPRAFETIDYVYRDQPKDTDADEAAKITFKP-RAMTFEMEICEQVGIKDDRVPYPMYWYSRNKIDLALFFTRQCTLLFTIYYLVGVTNNHYKRALLANAATSALRMHQRMPTVQLSRQFLVNLMHEDSAHYLFFSVIF-LPVS-LALLPIALFSMLNTMTIMDKHGAFIE---KQQSILQTIALCEIVLQLVCVVGVLSRLFLVTPVLYYRFLLLRYESRRNGHTRLFNREHNTHTHTFLWKLFETMIPKIISVSVVSQ????????????????????????????????????????????????????????????????????????????????????????????????????????????????????????????????????????????????????????????????????????????????????????????????????????????LAGKDKLDRIAIVNNDRCKPKRCNQECKRSCPIVKSGKLCIEVTTNDKIARISEELCIGCGICIKRCPFEAISIINLPSNLEKDTTHRYGPNSFKLHRLPIPRPGQVLGLVGTNGIGKSTALKILAGKLKPNLGRFSDPPDWTDILAHFRGSELQNYFTKILEDELKAVIKPQYVDVIPKALKGTVQANLDRKNDTGRLKEICDILDLNEIRSREVDQLSGGELQRFAIALVCIQNADVYMFDEPSSYLDVKQRLQAAQAIRSLIAPEKYVIVVEHDLSVLDYLSDFTCCLYGVPSAYGVVTMPFSVREGINIFLDGFVPTENLRFRDVALVFKVAESAQEEIKRLCHYEYPRMDKGLGGFHLDIEPGTFTDSEIIVMLGENGTGKTTFIRMLAGALKPDGDSELPTLNISYKPQKISPKSQKTVKELLMEKISDLFRHPQFQTDVVKPLQIDQILDQNVADLSGGELQRVALVLCLGKPADVYLIDEPSAYLDSEQRLVAAKVIKRFLLHSKKTGFVVEHDFIMATYLADRVIVFEGEPGVEAKACSPQSLLTGMNKFLSMLHITFRRDPNNFRPRINKMNSLKDVDQKRSGNFFFLEEEKSVHSLMKPPLKTVDAVEAELRERDRGRLRRGLETIDNLEDTRGMSNKEWVSQATRNEIKNRFKHLLKTFREKLRAMEQNQQSFELEYILAREQQALALFLPEVPHQMLELFNSASKEVVLSIYPNYTKIAPEIFVRIKDLAITDEIRSLRQLHLSQLIRTEGVVTSSTSIIPQLSLIKFDCLKCKYVLGPFIQTQEVKPGTCPGCQSLGPFAINMEETIFKNYQRLTTQESPGRISAGRVPRAKDVIVLGDLCDSCRPGDEIDLTGIYSNTYDGSLNIANGFPVFSTVIMANNIIRKEVDEDISRIVNLSKDPIFERIMASICPSVYGHKPVKRAIALALFGGVAKTQE-KHRVRGDINVLLCGDPGTAKSQFLKYTSKIAPRAVYTTGQGASAVGLTAYVSPVTREWTLEAGALVLADNGVCLIDEFDKMSDRDRTSIHEAMEQQTISVSKAGIVASLKARCSVIAAANPIGGRYNTFSQNVELTEPIISRFDIICVVRDQLLTKFVVRSHIKSHPIRQDLLRKYIVYARDKIKPVLQQDQIAKVYADMRRESAMTNSIPITARHIESIIRCSEAYARMHLRNYVDVRMAIKVTLESFVTQRAMVRKMTVLEDIENAFGKSNLYEVLGIDKHASNEQIKKAYRRMSLKVHPDRVSEEKKVEFTKKFQILAQVHYVLTDDERRKMYDEHGIIMNEDSLSGEADWSQYWRVLFPKVTKKDIESFIQNYQGSQDEKDDLSQLYNRYEGDMDKISQCHIAFEEDRT????????????????????????????????????????????????????????????????????????????????????????????????????????????????????????????????????????????????????????????????????????????????????????????????????????????--------------------------------------------------------------------------------------VPKLDKLNGIREESFRYSQLCTARDNFKHIFDVPANVATTRQYIREGKLLMAHHLSELENSRSALLYEIHRHPKSSSSDRTTLKHYFSEVDKLSEELEKQILNIMSRALNTVRQDPHTLVSALRIIEREEKFSIKRQAAT-GFMPQGRPKLLRKKMFENIEENVGERLSGNQFEDRTNNKLWLARHLEIIRLLILEDLRVVKKGFVQCFPPSYNIAHLLKLYHRCLRTHLQEL-AHLEGNEYVTLLNWVKAYPGPELLGHPDLDIDLPLLSDTLNELTNAYFVTIEKNYTEWMGNTINDTRDWPETDDSGHYQTTTPVFIFQMIDQHLQVAKSFTLVNRVLALSMNQLAIFAKKYREAIEKYHFTDRQFTPYMVAITNNCSAFS-FEALRDDSIDYLLQELFLDVDKIATIGTENICLTLEDYFQDYLQNQLAKGYIRAIL--QRKITLRDYSQRKDFCKREVLKASPFDALKSLAGCLELKDSSLLSLEVSG-----QALSQEEDG------------------EKHEFQAEVTRMMKLIINSLYTNKEIFLRELISNASDALDKIRVLALTSKEALDSGSELEIRIKADDTTNTLHITDTGIGMTRQELINNLGTIAKSGTSEFLKQNLESSDQVQLNDLIGQFGVGFYSSFLVADKVVVSSKANDDPERHLWISNSSAYTVAVDDSEDKLKRGTTVTLYLKEEAKDFLKQDTLQELIKKYSQFINFPIYLWTSKTVQEEVPVEGD-----DEEDVEKNDDATVEEEEQNKKAKTKKVDKTVWEWKRINVAKPIWQRKPIEVTENEYNEFYKSITRDYQNPLARTHFTAEGELTFKSLLFVPKQQPSETFNKYGTKSDNIKLYVRRVFISDEFNDLMPNYLSFVRGVVDSDDLPLNVGRETLQQHKLLKVIKKKLVRKTLDMIKKIDESKYTEFWKEYSTNLKLGVIEDQSNRNRIAKLLRFHSSKTGADKWTSLSDYVKDMQSDQEQIYYIAGSSFDELSQSPFVESALKRGYEVLYLTDAVDEYCLSNLPEFDGKKFQNVAKEGLVLGKSKS-ESQKKAIESKFKPLVGWLKGTALKDKVQNVVLSERLSESPAALVAPTFGWTGNMERLAKSNAHSKSKDMTRDYYLSQKKILEINPHHPIIKELLKRVALDEGDSKARQTAELLFETATIRSGYMLQDTEEFGKRVEKLMRVNLDVPEDAPVEEET--GAEGYTMSAQQNTVLTIGEIIKELIAAHERNEDVNLTKLRFHVARKYKLAKFPRLIDIISAVPPQYRNILLPKLRAKPIRSASGIAVIAVMCKPHRCPHINYTGNICVYCPGGPDSDFEYSTQSYTGYEPTSMRAIRARYDPYLQSQGRIEQLQQLGHTVDKVEFIVMGGTFMSLPADYRDYFIRNLHDSLSGHHSQSVDEAVRMSEKSRTKCIGITIETRPDYCLNKHISDMLAYGCTRLEIGLQSIYEDVAVDTNRGHTVRSVCQSFQLGKDSGFKIVSHIMPNLPNVDLERDLEQFVELFANPAYRPDGLKIYPTLVIRGTGLYELWKTGRYKSYPPSTLIDLIAQILSLVPPWTRIYRIQRDIPMPLVSSGVEHGNIREMVLARMGDLGLKCRDVRTREVGIQEIHHKIRPYNIELIRRDYTANDGWETFLTYEDVEQDILIGLLRLRLCSIHTFRPELMQQTSIVREHVYGSTVPISTKDPTKFQHQGFGMLLMEEAERIAREEHGSQKLAVISGVGTRNYYRKMGYELEGPYMTK
[truncated: 549,809 more chars]
