# Supplementary material for: Symbiotic bacteria of the gall-inducing mite Fragariocoptes setiger (Eriophyoidea) and phylogenomic resolution of the eriophyoid position among Acari
Source: Sci Rep. 2022 Mar 9;12:3811. doi: 10.1038/s41598-022-07535-3 (PMC8907322; doi:10.1038/s41598-022-07535-3)
Supplement: Supplementary file 7 — Supplementary Table S1. [file 41598_2022_7535_MOESM7_ESM.docx]

Supplementary Table S1. Quality assessment of the mite metatranscriptome (soft filtered contigs) vs mite 'clean' genome in rnaQUAST.

| Metrics | RNA soft filtered contigs |
| --- | --- |
| Genes | 3,171 |
| Avg. number of exons per isoform | 2.863 |
| Transcripts | 145,220 |
| Transcripts > 500 bp | 31,089 |
| Transcripts > 1000 bp | 12,133 |
| Aligned | 22,669 |
| Uniquely aligned | 20,563 |
| Multiply aligned | 62 |
| Unaligned | 122,551 |
| Avg. aligned fraction | 0.97 |
| Avg. alignment length | 1084.0 |
| Avg. mismatches per transcript | 2.015 |
| Misassemblies | 573 |
| Database coverage | 0.826 |
| 50%-assembled genes | 2,161 |
| 95%-assembled genes | 1,355 |
| 50%-covered genes | 2,464 |
| 95%-covered genes | 1,953 |
| 50%-assembled isoforms | 2,161 |
| 95%-assembled isoforms | 1,355 |
| 50%-covered isoforms | 2,464 |
| 95%-covered isoforms | 1,953 |
| Mean isoform coverage | 0.898 |
| Mean isoform assembly | 0.779 |
| 50%-matched | 4,419 |
| 95%-matched | 2,123 |
| Unannotated | 13,843 |
| Mean fraction of transcript matched | 0.21 |
